# Encorafenib, cetuximab and chemotherapy in BRAF-mutant colorectal cancer: a randomized phase 3 trial

**DOI:** 10.1038/s41591-024-03443-3

**Published:** 2025-01-25

**Authors:** Scott Kopetz, Takayuki Yoshino, Eric Van Cutsem, Cathy Eng, Tae Won Kim, Harpreet Singh Wasan, Jayesh Desai, Fortunato Ciardiello, Rona Yaeger, Timothy S. Maughan, Elena Beyzarov, Xiaoxi Zhang, Graham Ferrier, Xiaosong Zhang, Josep Tabernero

**Affiliations:** 1 University of Texas MD Anderson Cancer Center, Houston, TX USA; 2 National Cancer Center Hospital East, Kashiwa, Japan; 3 University Hospitals Gasthuisberg Leuven and KU Leuven, Leuven, Belgium; 4 Vanderbilt-Ingram Cancer Center, Nashville, TN USA; 5 Asan Medical Center, University of Ulsan College of Medicine, Seoul, South Korea; 6 Hammersmith Hospital, Division of Cancer, Imperial College London, London, UK; 7 Peter MacCallum Cancer Centre and the University of Melbourne, Melbourne, VIC Australia; 8 University of Campania Luigi Vanvitelli, Naples, Italy; 9 Memorial Sloan Kettering Cancer Center, New York, NY USA; 10 University of Liverpool, Liverpool, UK; 11 Pfizer, Inc., New York, NY USA; 12 Pfizer, Inc., South San Francisco, CA USA; 13 Vall d’Hebron Hospital Campus and Vall d’Hebron Institute of Oncology (VHIO), University of Vic – Central University of Catalonia, Barcelona, Spain

**Keywords:** Gastrointestinal cancer, Colorectal cancer

## Abstract

Encorafenib + cetuximab (EC) is approved for previously treated BRAF V600E-mutant metastatic colorectal cancer (mCRC) based on the BEACON phase 3 study. Historically, first-line treatment of BRAF V600E-mutant mCRC with chemotherapy regimens has had limited efficacy. The phase 3 BREAKWATER study investigated EC+mFOLFOX6 versus standard of care (SOC) in patients with previously untreated BRAF V600E mCRC. The dual primary endpoint of progression-free survival is event driven; data were not mature at data cutoff. BREAKWATER met the other dual primary endpoint of objective response rate, demonstrating significant and clinically relevant improvement in objective response rate (EC+mFOLFOX6: 60.9%; SOC: 40.0%; odds ratio, 2.443; 95% confidence interval (CI): 1.403–4.253; 99.8% CI: 1.019–5.855; one-sided *P* = 0.0008). Median duration of response was 13.9 versus 11.1 months. At this first interim analysis of overall survival, the hazard ratio was 0.47 (95% CI: 0.318–0.691; repeated CI: 0.166–1.322). Serious adverse event rates were 37.7% versus 34.6%. The safety profiles were consistent with those known for each agent. BREAKWATER demonstrated a significantly improved response rate that was durable for first-line EC+mFOLFOX6 versus SOC in patients with BRAF V600E mCRC. ClinicalTrials.gov identifier: NCT04607421.

## Abstract (web summary)

As presented at the 2025 ASCO GI Cancers Symposium: in the phase 3 BREAKWATER trial, patients with previously untreated BRAF V600E metastatic colorectal cancer received the BRAF inhibitor encorafenib, the anti-EGFR monoclonal antibody cetuximab and chemotherapy mFOLFOX6 versus investigator’s choice of chemotherapy with or without bevacizumab, leading to an improved objective response rate, with the dual primary endpoint of progression free survival still maturing.

## Main

*BRAF* V600E mutations occur in 8–12% of metastatic colorectal cancers (mCRCs)^1,2^; the presence of these mutations has emerged as a distinct subtype that is characterized by poor prognosis compared with wild-type disease and resistance to standard chemotherapy regimens^1,2^. *BRAF* V600E mutations are found in multiple tumor types, and BRAF inhibitors in combination with MEK inhibitors are part of the standard of care (SOC) in BRAF-mutant melanoma and non-small cell lung cancer^3,4^.

Encorafenib is a highly selective, ATP-competitive small-molecule BRAF inhibitor with anti-proliferative and apoptotic activity in tumor cells expressing *BRAF* V600E and has prolonged pharmacodynamic activity compared with other approved BRAF inhibitors^5,6^. BRAF V600E inhibition causes rapid pathway feedback reactivation through the epidermal growth factor receptor (EGFR)^7,8^; previous clinical trials targeting BRAF simultaneously with EGFR inhibition have shown the value of this combination in targeting MAPK signaling^9,10^. Encorafenib plus cetuximab, an anti-EGFR monoclonal antibody, is approved for previously treated BRAF V600E-mutant mCRC based on results from the BEACON study^11^. Median overall survival was 8.4 months, the objective response rate was 20%, median progression-free survival was 4.2 months and no new safety signals were observed with encorafenib plus cetuximab^9^.

Despite this promising option of targeted treatments in the second and later lines as demonstrated in the BEACON study, first-line chemotherapies with or without a biologic agent (eg, bevacizumab) have had limited efficacy for BRAF V600E-mutant mCRC^12^. Furthermore, the addition of bevacizumab with doublet and triplet chemotherapy has been debated due to tolerability concerns^13^. There are currently no first-line activation pathway-targeted treatments indicated for patients with BRAF V600E-mutant mCRC; therefore, a treatment that can demonstrate improved efficacy in the first-line setting is needed given the poor prognosis compared with BRAF wild-type mCRC.

BREAKWATER (NCT04607421) is a phase 3 study evaluating encorafenib plus cetuximab with or without standard chemotherapy (oxaliplatin, leucovorin and 5-FU (mFOLFOX6) (EC±mFOLFOX6) versus SOC, investigator’s choice of chemotherapy (mFOLFOX6; irinotecan, oxaliplatin, leucovorin and 5-FU (FOLFOXIRI) or oxaliplatin and capecitabine (CAPOX)) with or without bevacizumab for the first-line treatment of patients with BRAF V600E-mutabt mCRC. Data from the safety lead-in portion demonstrated encouraging response rates and progression-free survival of encorafenib and cetuximab with chemotherapy (mFOLFOX6 or irinotecan, leucovorin and 5-FU (FOLFIRI))^14^.

Reported here are one of the dual primary endpoints, objective response rate and the first interim analysis of overall survival, duration of response, time to response and safety in the EC+mFOLFOX6 and SOC arms from the phase 3 portion. The second dual primary endpoint, progression-free survival, is event driven; the required number of events needed for analysis had not yet been achieved at the time of writing and will be reported later. Additional planned secondary endpoints not reported in this paper are progression after next line of therapy, patient-reported outcomes, pharmacokinetics and biomarker endpoints. An interactive infographic is available at https://www.breakwaterphase3-infographic.com/.

## Results

### Patients

**Fig. 1:**
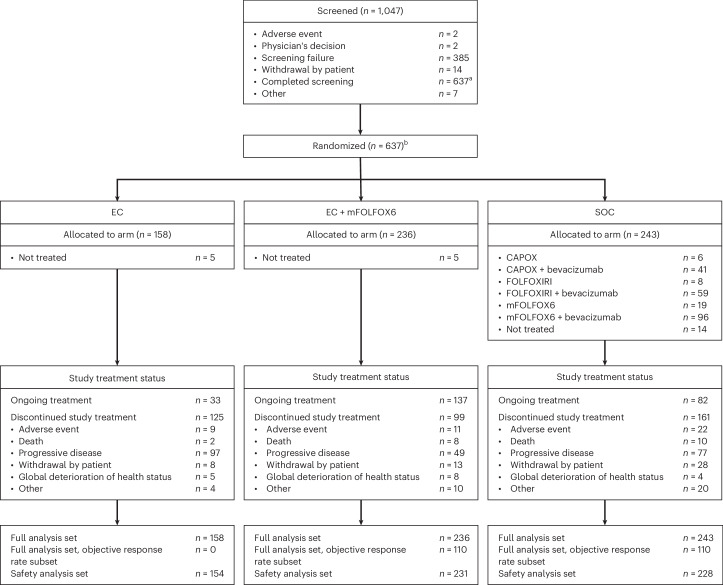
Patient disposition. CAPOX, oxaliplatin and capecitabine; EC, encorafenib and cetuximab; mFOLFOX6, oxaliplatin, leucovorin and 5-FU; FOLFOXIRI, irinotecan, oxaliplatin, leucovorin and 5-FU; mCRC, metastatic colorectal cancer; SOC, standard of care. ^a^One participant who was randomized to the EC+mFOLFOX6 arm (but never treated) was inadvertently entered as withdrawal by subject on the screening case report form page. ^b^Following closure of the EC arm, randomization was 1:1 to the EC+mFOLFOX6 and SOC arms.

Patients were enrolled between 16 November 2021 and 22 December 2023 in the phase 3 portion of the study. Eligible patients had previously untreated BRAF V600E-mutant mCRC in the metastatic setting, measurable disease per Response Evaluation Criteria in Solid Tumors (RECIST) version 1.1 and Eastern Cooperative Oncology Group performance status 0-1. Data reported here are for the EC+mFOLFOX6 and SOC arms; data from the EC arm will be reported at a later date. Patient disposition is shown in Fig. 1; 236 patients were randomized to the EC+mFOLFOX6 arm and 243 were randomized to the SOC arm in the phase 3 portion of the study. At data cutoff (22 December 2023), study treatment was ongoing in 137 patients in the EC+mFOLFOX6 arm and 82 in the SOC arm. A summary of important protocol deviations is reported in Supplementary Table 1.

Baseline demographics and disease characteristics were similar across arms (Table 1). The median age was 61 years, 49.5% of patients were female and 42.0% of patients had an Eastern Cooperative Oncology Group performance status of 1. The majority of patients had tumors that were on the right (61.0%), and most were microsatellite stable/proficient mismatch repair (95.2%).

**Table 1:** Baseline demographics and disease characteristics

	EC + mFOLFOX6 (*n* = 236)	SOC (*n* = 243)	Total (*n* = 479)
Median age (range), years	60.0 (24–81)	62.0 (28–84)	61.0 (24–84)
Male, *n* (%)	123 (52.1)	119 (49.0)	242 (50.5)
Female, *n* (%)	113 (47.9)	124 (51.0)	237 (49.5)
**Race,** ***n*** **(%)**			
White	141 (59.7)	144 (59.3)	285 (59.5)
Asian	88 (37.3)	91 (37.4)	179 (37.4)
Multiracial	0	2 (0.8)	2 (0.4)
Black or African American	0	1 (0.4)	1 (0.2)
Not reported	7 (3.0)	5 (2.1)	12 (2.5)
**Body site,** ***n*** **(%)**			
Colon	191 (80.9)	192 (79.0)	383 (80.0)
Rectum	24 (10.2)	27 (11.1)	51 (10.6)
Cecum	21 (8.9)	24 (9.9)	45 (9.4)
**Side of tumor,** ***n*** **(%)**			
Left	89 (37.7)	98 (40.3)	187 (39.0)
Right	147 (62.3)	145 (59.7)	292 (61.0)
**Stage at initial diagnosis,** ***n*** **(%)**			
I	3 (1.3)	2 (0.8)	5 (1.0)
II	13 (5.5)	10 (4.1)	23 (4.8)
III	37 (15.7)	43 (17.7)	80 (16.7)
IV	183 (77.5)	188 (77.4)	371 (77.5)
**Primary tumor resection,** ***n*** **(%)**			
Complete	116 (49.2)	105 (43.2)	221 (46.1)
Partial	14 (5.9)	13 (5.3)	27 (5.6)
None	106 (44.9)	125 (51.4)	231 (48.2)
**No. of organs involved,** ***n*** **(%)**^a^			
≤2	122 (51.7)	129 (53.1)	251 (52.4)
≥3	114 (48.3)	114 (46.9)	228 (47.6)
Liver metastases, *n* (%)^a^	144 (61.0)	156 (64.2)	300 (62.6)
**Eastern Cooperative Oncology Group performance status,** ***n*** **(%)**			
0	129 (54.7)	131 (53.9)	260 (54.3)
1	103 (43.6)	98 (40.3)	201 (42.0)
Missing	4 (1.7)	14 (5.8)	18 (3.8)
**Central BRAF V600E status (tumor tissue),** ***n*** **(%)**			
Detected	226 (95.8)	224 (92.2)	450 (93.9)
Indeterminate	0	1 (0.4)	1 (0.2)
Not detected	4 (1.7)	2 (0.8)	6 (1.3)
Not available	6 (2.5)	16 (6.6)	22 (4.6)
**Local microsatellite instability/mismatch repair deficiency status,** ***n*** **(%)**			
High microsatellite instability/mismatch repair deficiency	1 (0.4)	0	1 (0.2)
Microsatellite stable/proficient mismatch repair	229 (97.0)	227 (93.4)	456 (95.2)
Not available	6 (2.5)	16 (6.6)	22 (4.6)
**Carcinoembryonic antigen at baseline,** ***n*** **(%)**			
≤5 μg liter^-1^	65 (27.5)	63 (25.9)	128 (26.7)
>5 μg liter^-1^	166 (70.3)	163 (67.1)	329 (68.7)
Missing	5 (2.1)	17 (7.0)	22 (4.6)
**C-reactive protein at baseline,** ***n*** **(%)**			
≤10 mg liter^-1^	125 (53.0)	119 (49.0)	244 (50.9)
>10 mg liter^-1^	105 (44.5)	107 (44.0)	212 (44.3)
Missing	6 (2.5)	17 (7.0)	23 (4.8)

EC+mFOLFOX6, encorafenib and cetuximab plus oxaliplatin, leucovorin and 5-FU.

^a^Number of organs and presence of liver metastases are based on blinded independent central review data for the phase 3 portion of the study.

### Treatment

The median duration of treatment was 28.1 weeks (range: 1.3–107.4) in the EC+mFOLFOX6 arm and 20.4 weeks (range: 1.1–98.3) in the SOC arm (Extended Data Table 1). Median duration of treatment and relative dose intensities for each drug in each arm are reported in Extended Data Table 1 and Extended Data Table 2.

### Efficacy

**Fig. 2:**
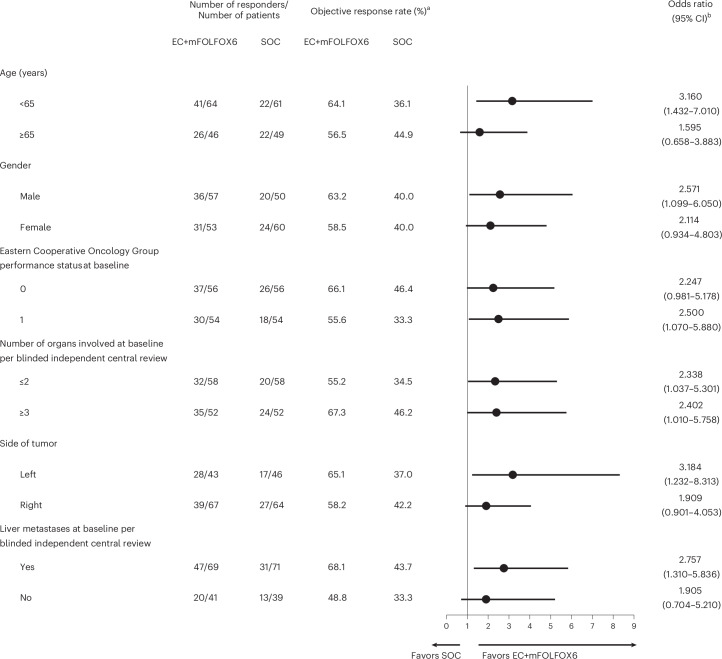
Subgroup analyses of confirmed objective response rate by blinded independent central review. Odds ratios (center) are presented with 95% CI (error bars). EC+mFOLFOX6, encorafenib and cetuximab plus oxaliplatin, leucovorin and 5-FU. ^a^Percentages were calculated based on the number of participants in the objective response rate subset of all randomized patients in each treatment group. ^b^Objective response rate calculated based on the number of participants in the objective response rate subset of all randomized patients within each treatment group and subgroup. The odds ratio was estimated using the Mantel–Haenszel method. The exact CI was calculated.

In the objective response rate subset of all randomized patients, the dual primary endpoint of confirmed objective response rate by blinded independent central review was met (60.9% (95% confidence interval (CI): 51.6–69.5) versus 40.0% (95% CI: 31.3–49.3) in the EC+mFOLFOX6 and SOC arm, respectively; odds ratio = 2.443 (95% CI: 1.403–4.253; 99.8% CI: 1.019–5.855), one-sided *P* = 0.0008) (Table 2). Predefined subgroup analyses of objective response rate showed consistency in results (Fig. 2). The median time to response by blinded independent central review was 7.1 weeks (range: 5.7–53.7) versus 7.3 weeks (range: 5.4–48.0), respectively (Table 2). The median duration of response was 13.9 months (95% CI: 8.5-not estimable) versus 11.1 months (95% CI: 6.7–12.7), respectively (Table 2). The proportion of patients with a duration of response of ≥6 months was 68.7% and 34.1%, respectively, and the proportion of patients with a duration of response of ≥12 months was 22.4% and 11.4%, respectively (Table 2). Data by investigator assessment also showed consistent treatment effects (Extended Data Table 3).

**Table 2:** Confirmed objective response rate, time to treatment and duration of response by blinded independent central review

	EC + mFOLFOX6 (*n* = 110)	SOC (*n* = 110)
**Confirmed best overall response,** ***n*** **(%)**		
Complete response	3 (2.7)	2 (1.8)
Partial response	64 (58.2)	42 (38.2)
Stable disease	31 (28.2)	34 (30.9)
Non-complete response/non-progressive disease	3 (2.7)	4 (3.6)
Progressive disease	3 (2.7)	9 (8.2)
Not evaluable	6 (5.5)	19 (17.3)
Confirmed objective response rate (95% CI), %^a^	60.9 (51.6–69.5)	40.0 (31.3–49.3)
Odds ratio (95% CI; 99.8% CI)^b^	2.443 (1.403–4.253; 1.019–5.855)	
One-sided *P* value	0.0008	
	*n* = 67	*n* = 44
Median time to response (range), weeks	7.1 (5.7–53.7)	7.3 (5.4–48.0)
Estimated median duration of response (range), months	13.9 (8.5–NE)	11.1 (6.7–12.7)
Patients with a duration of response of ≥ 6 months, *n* (%)	46 (68.7)	15 (34.1)
Patients with a duration of response of ≥ 12 months, *n* (%)	15 (22.4)	5 (11.4)

CI, confidence interval; EC+mFOLFOX6, encorafenib and cetuximab plus oxaliplatin, leucovorin and 5-FU; NE, not estimable.

^a^Defined as complete response or partial response according to RECIST 1.1 recorded from the date of randomization until the date of the first documentation of progression of disease, death or start of subsequent anticancer therapy; both complete response and partial response must be confirmed by repeat assessments performed no less than 4 weeks after the criteria for response are first met.

^b^Asymptotic CI used.

**Fig. 3:**
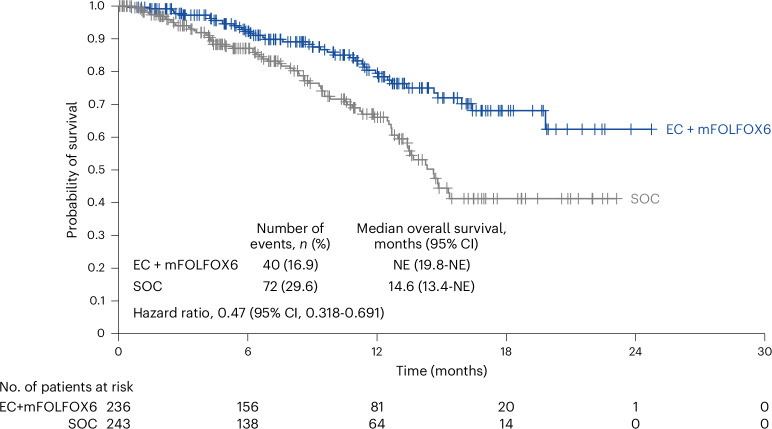
Overall survival. Hazard ratio repeated CI: 0.166–1.322.

Upon achieving the dual primary endpoint of objective response rate, the key secondary endpoint of overall survival was formally tested in all randomized patients following the prespecified plan with one-sided alpha of 0.000000083, calculated as a portion of the nominal one-sided alpha of 0.001 based on the observed number of deaths (40 (16.9%) deaths in the EC+mFOLFOX6 arm; 72 (29.6%) deaths in the SOC arm). The median overall survival follow-up was 10.3 months (95% CI: 8.6–11.6) in the EC+mFOLFOX6 arm and 9.8 months (95% CI: 7.5–11.3) in the SOC arm. At this interim analysis of overall survival, the overall survival hazard ratio was 0.47 (95% CI: 0.318–0.691; repeated CI: 0.166–1.322)^15^; statistical significance was not achieved at this time (*P* = 0.0000454, one-sided alpha of 0.000000083). The median overall survival was not estimable (95% CI: 19.8 to not estimable) versus 14.6 months (95% CI: 13.4-not estimable), respectively (Fig. 3). The landmark overall survival rates were 92.3% versus 87.1% at 6 months and 79.5% versus 66.1% at 12 months.

### Subsequent systemic anticancer treatments

Approximately half of the patients who discontinued study treatment received subsequent systemic anticancer treatment by the data cutoff. The majority of patients in the EC+mFOLFOX6 arm received subsequent chemotherapies, especially FOLFIRI-based combination. The majority of patients from the SOC arm received BRAF inhibitor-based subsequent therapies (Extended Data Table 4).

### Safety

A safety summary is reported in Table 3 and Extended Data Table 5. Treatment-emergent adverse events occurred in 99.6% versus 97.8% of patients in the EC+mFOLFOX6 arm versus in the SOC arm, respectively. Similar rates of treatment-related adverse events were reported (Extended Data Table 5). The most frequent (≥30% of patients based on the EC+mFOLFOX6 arm) treatment-emergent adverse events were nausea (51.1% in the EC+mFOLFOX6 arm versus 48.2% in the SOC arm), anemia (36.4% versus 22.8%, respectively), diarrhea (34.2% versus 46.9%, respectively), decreased appetite (33.3% versus 25.0%, respectively), vomiting (33.3% versus 21.1%, respectively) and neutrophil count decreased (32.0% versus 28.1%, respectively) (Table 3).

**Table 3:** Most common all-causality treatment-emergent adverse events (≥10% of patients in any arm) by preferred term

	EC + mFOLFOX6 (*n* = 231)		SOC (*n* = 228)	
	Any grade	Grade ≥3	Any grade	Grade ≥3
Any adverse event	230 (99.6)	181 (78.4)	223 (97.8)	149 (65.4)
Nausea	118 (51.1)	6 (2.6)	110 (48.2)	7 (3.1)
Anemia	84 (36.4)	25 (10.8)	52 (22.8)	8 (3.5)
Diarrhea	79 (34.2)	3 (1.3)	107 (46.9)	8 (3.5)
Neutrophil count decreased	74 (32.0)	42 (18.2)	64 (28.1)	38 (16.7)
Decreased appetite	77 (33.3)	5 (2.2)	57 (25.0)	3 (1.3)
Vomiting	77 (33.3)	8 (3.5)	48 (21.1)	5 (2.2)
Asthenia	62 (26.8)	10 (4.3)	33 (14.5)	3 (1.3)
Pyrexia	60 (26.0)	4 (1.7)	31 (13.6)	1 (0.4)
Peripheral sensory neuropathy	57 (24.7)	13 (5.6)	49 (21.5)	5 (2.2)
Rash	57 (24.7)	2 (0.9)	6 (2.6)	0
Fatigue	56 (24.2)	6 (2.6)	57 (25.0)	6 (2.6)
Neuropathy peripheral	54 (23.4)	16 (6.9)	48 (21.1)	6 (2.6)
Arthralgia	51 (22.1)	2 (0.9)	8 (3.5)	0
Neutropenia	51 (22.1)	34 (14.7)	51 (22.4)	21 (9.2)
Alopecia	49 (21.2)	0	23 (10.1)	0
Constipation	47 (20.3)	1 (0.4)	44 (19.3)	1 (0.4)
Platelet count decreased	46 (19.9)	3 (1.3)	28 (12.3)	4 (1.8)
White blood cell count decreased	42 (18.2)	13 (5.6)	32 (14.0)	8 (3.5)
Lipase increased	46 (19.9)	34 (14.7)	22 (9.6)	12 (5.3)
Weight decreased	40 (17.3)	2 (0.9)	19 (8.3)	0
Skin hyperpigmentation	39 (16.9)	0	5 (2.2)	0
Abdominal pain	38 (16.5)	7 (3.0)	47 (20.6)	3 (1.3)
Dermatitis acneiform	35 (15.2)	2 (0.9)	1 (0.4)	0
Hypokalemia	30 (13.0)	4 (1.7)	22 (9.6)	7 (3.1)
Aspartate aminotransferase increased	29 (12.6)	2 (0.9)	25 (11.0)	3 (1.3)
Dry skin	29 (12.6)	0	8 (3.5)	0
Headache	29 (12.6)	1 (0.4)	17 (7.5)	0
Mucosal inflammation	29 (12.6)	4 (1.7)	22 (9.6)	1 (0.4)
Paresthesia	28 (12.1)	6 (2.6)	18 (7.9)	3 (1.3)
Dysgeusia	27 (11.7)	0	31 (13.6)	0
Epistaxis	27 (11.7)	0	28 (12.3)	0
Hypomagnesemia	27 (11.7)	2 (0.9)	9 (3.9)	1 (0.4)
Stomatitis	27 (11.7)	4 (1.7)	32 (14.0)	3 (1.3)
Alanine aminotransferase increased	26 (11.3)	3 (1.3)	22 (9.6)	3 (1.3)
Myalgia	26 (11.3)	0	9 (3.9)	0
Thrombocytopenia	26 (11.3)	0	18 (7.9)	0
Neurotoxicity	25 (10.8)	11 (4.8)	18 (7.9)	0
Palmar-plantar erythrodysesthesia syndrome	25 (10.8)	3 (1.3)	18 (7.9)	2 (0.9)
Pruritus	24 (10.4)	0	4 (1.8)	0
Hypoalbuminemia	23 (10.0)	1 (0.4)	13 (5.7)	0
Insomnia	23 (10.0)	0	13 (5.7)	0

Grade 3/4 adverse events occurred in 74.0% of patients in the EC+mFOLFOX6 arm versus 61.0% in the SOC arm; grade 3/4 treatment-related adverse events occurred in 69.7% versus 53.9% of patients, respectively (Extended Data Table 5).

Overall, there were 38 (16.5%) deaths in the EC+mFOLFOX6 arm and 69 (30.3%) deaths in the SOC arm; the disease under study was the most common cause (35 [15.2%] deaths in the EC+mFOLFOX6 arm versus 60 (26.3%) deaths in the SOC arm, respectively). Grade 5 (fatal) adverse events occurred in 4.3% versus 4.4% of patients, respectively; grade 5 treatment-related adverse events occurred in 0% versus 0.4% of patients, respectively (Extended Data Table 5).

Serious treatment-emergent adverse events occurred in 37.7% versus 34.6% of patients in the EC+mFOLFOX6 versus SOC arms, respectively (Extended Data Table 6). The most common serious adverse events are reported in Extended Data Table 6. Serious treatment-related adverse events occurred in 18.2% versus 19.3% of patients, respectively (Extended Data Table 6).

Adverse events leading to permanent discontinuation of any study intervention occurred in 20.8% versus 14.9% of patients in the EC+mFOLFOX6 versus SOC arms, respectively. Adverse events leading to dose reduction of any study intervention occurred in 61.0% versus 47.8% of patients, respectively. Permanent discontinuation of chemotherapy with or without bevacizumab (as appropriate for the treatment group) due to adverse event was reported in 15.6% of patients in the EC+mFOLFOX6 arm and 14.9% of patients in the SOC arm; dose reduction of any of these interventions were reported in 55.8% and 47.8%, respectively (Extended Data Table 5).

## Discussion

BREAKWATER has met one of its dual primary endpoints, objective response rate, demonstrating a statistically significant and clinically relevant benefit in objective response rate by blinded independent central review with EC+mFOLFOX6 versus SOC. At the time of this analysis, data also showed a point estimate of overall survival hazard ratio of 0.47 for EC+mFOLFOX6 versus SOC; however, 16.9% of patients in the EC+mFOLFOX6 arm and 29.6% of patients in the SOC arm had an event at data cutoff for this first interim analysis and did not achieve the prespecified statistical significance. BREAKWATER is ongoing and once the required number of events specified in the protocol have occurred, the primary analysis of progression-free survival, the other dual primary endpoint, will be conducted and subsequently reported.

Investigator-assessed objective response rates were consistent with the objective response rates by blinded independent central review. Secondary endpoints showed the response to EC+mFOLFOX6 was rapid and durable. The percentage of patients with a duration of response beyond 6 or 12 months approximately doubled in the EC+mFOLFOX6 arm compared with the SOC arm. These early overall survival data showed a clear separation between the arms in the Kaplan-Meier curves, despite the number of deaths at data cutoff and data were not statistically significant at this first interim analysis. Follow-up is ongoing, with planned additional interim and final analysis. The subsequent system anticancer treatments reported in the study are similar to the current real-world practice. The majority of the patients in the SOC arm received a BRAF inhibitor-based subsequent anticancer treatment. Thus, the observed difference in overall survival is evaluated against a valid current SOC.

Subgroup analyses of objective response rates by blinded independent central review showed the clinical benefit of EC+mFOLFOX6 was seen across all key clinical subgroups; notably, clinical benefit was observed regardless of presence of liver metastases.

Trials of chemotherapy plus cetuximab or bevacizumab, BRAF inhibitor monotherapy, BRAF inhibitors with MEK inhibitors, and BRAF inhibitors with chemotherapy have shown limited benefit over the current SOC for patients with BRAF V600E-mutant mCRC^7,10,12,16–23^. Our data highlight the importance of combining dual targeted therapy (encorafenib and cetuximab) with chemotherapy in BRAF V600E-mutant CRC to improve patient outcomes in the first-line setting. It is currently unknown what specific mechanisms are responsible for the observed clinical benefit of EC and chemotherapy compared with chemotherapy alone. The combination of cytotoxic chemotherapy, which has a nonselective antitumor effect, and targeted therapy may overcome intratumor heterogeneity through an additive effect, targeting different cell populations, and ultimately improving clinical outcomes. Ongoing exploratory analyses may provide further insights into predictive biomarkers for this combination therapy.

Safety data showed that EC+mFOLFOX6 was generally tolerable, with a safety profile consistent with that known for each agent. Patients in the EC+mFOLFOX6 arm had a longer duration of treatment and maintained high relative dose intensities. The addition of EC to chemotherapy was generally tolerable without significant increase in chemotherapy dose reduction or discontinuation.

Recent data suggest that combining chemotherapy with targeted therapy may prevent the emergence of resistance alterations and allow for prolonged antitumor efficacy, these preclinical data support the rationale for the BREAKWATER study to combine EC with chemotherapy in BRAF V600E-mutant mCRC^24,25^. The long duration of response achieved in the BREAKWATER trial with EC+mFOLFOX6 suggests the potential for prolonged effect of the combination. Despite these promising data, there is a further need to characterize the mechanisms of resistance to help improve outcomes in patients who ultimately progress on treatment. A retrospective, exploratory, clinical and molecular analysis of the BEACON study characterized potential biological determinants underlying response and acquired resistance to BRAF-targeted therapy, with or without MEK inhibition, in BRAF V600E-mutant mCRC^26^. Future biomarker analyses of the BREAKWATER study may shed light on the resistance mechanism by comparing chemotherapy plus targeted therapy versus chemotherapy or targeted therapy alone.

Based on the results from the phase 2 ANCHOR study of encorafenib, cetuximab and binimetinib^27^, it was suggested that the likelihood to demonstrate superiority in the EC arm versus the SOC arm was relatively low. This low likelihood, together with the fact that the majority of patients with BRAF V600E-mutant mCRC require an intensive first-line regimen to control the aggressive tumor growth, supports the investigation of EC+mFOLFOX6, and led to the early closure of EC arm enrollment.

BREAKWATER excluded patients with MSI-H or dMMR tumors unless ineligible to receive immune checkpoint inhibitors due to a preexisting medical condition. The programmed death 1 inhibitor pembrolizumab has shown clinical benefit as a first-line therapy for MSI-H or dMMR mCRC^28^. SEAMARK is an ongoing phase 2 study evaluating first-line EC with pembrolizumab versus pembrolizumab alone in patients with BRAF V600E-mutant and MSI-H/dMMR mCRC^29^.

Furthermore, the phase 3 portion of this study only investigated EC in combination with mFOLFOX6. As previously mentioned, the safety lead-in portion evaluated EC plus mFOLFOX6 or FOLFIRI in a small number of patients and showed encouraging results^14,30^. BREAKWATER is further evaluating EC plus FOLFIRI versus FOLFIRI with or without bevacizumab in the ongoing cohort 3 portion.

BREAKWATER showed substantially improved clinical benefit with EC+mFOLFOX6 as a first-line treatment for patients with BRAF V600E-mutant mCRC. These encouraging data support this regimen to potentially become the new SOC in BRAF V600E-mutant mCRC; prespecified analyses of mature progression-free survival and overall survival data are planned.

## Methods

### Trial oversight

BREAKWATER enrolled in 28 countries. It was designed and overseen by a steering committee, representatives of the sponsor, and an independent data monitoring committee. BREAKWATER was supported by Pfizer, Inc. Informed consent from patients was obtained prior to enrollment. The protocol, including amendments and was approved by the relevant ethics committee/institutional review board at each site (See Supplementary Information). BREAKWATER was performed in accordance with consensus ethical principles derived from international guidelines, including the Declaration of Helsinki and CIOMS International Ethical Guidelines, applicable International Conference on Harmonization Good Clinical Practice guidelines, and applicable laws and regulations, including applicable privacy laws. The listing of investigators who conducted the study is provided in the Supplementary Information. Data collection and analysis were performed by the sponsor in collaboration with the authors. The authors had access to the study data. The authors, in collaboration with the sponsor, made the decision to submit the results for publication. The first draft of the manuscript was developed using third-party medical writing support, provided by the sponsor, in collaboration with the authors. The authors assume responsibility for the accuracy and completeness of the data and analyses and for the fidelity of the trial and this report to the protocol.

### Patients

BREAKWATER enrolled patients who were at least 16 years of age (where permitted locally), with histologically or cytologically confirmed colorectal adenocarcinoma that had evidence of Stage IV metastatic disease, measurable disease (RECIST 1.1)^31^ and presence of a *BRAF* V600E mutation assessed by local or central laboratory testing. *BRAF* V600E mutation status was confirmed retrospectively by the central laboratory using tumor tissue collected within 2 years prior to study enrollment if not done at screening. Patients were eligible if they had not received prior systemic treatment for metastatic disease (prior [neo]adjuvant therapy was considered to be metastatic treatment if relapse or metastasis <6 months from the end of [neo]adjuvant therapy) and were ineligible if they previously received any selective BRAF inhibitor or any EGFR inhibitor. Eligible patients had an Eastern Cooperative Oncology Group performance status of 0 or 1, and adequate bone marrow, hepatic, and renal function. Patients with symptomatic brain metastases, microsatellite instability-high/mismatch repair deficient tumors (MSI-H/dMMR; unless ineligible to receive immune checkpoint inhibitors due to a preexisting medical condition), or a *RAS* mutation were excluded.

### Trial design and treatment

Patients were randomized 1:1:1 to the EC arm (encorafenib 300 mg orally once daily; cetuximab 500 mg/m^2^ intravenously once every 2 weeks), EC+mFOLFOX6 arm (encorafenib 300 mg orally once daily; cetuximab 500 mg/m^2^ intravenously once every 2 weeks; oxaliplatin 85 mg/m^2^ intravenously, leucovorin 400 mg/m^2^ intravenously, and 5-FU 400 mg/m^2^ intravenous bolus, then 5-FU 2400 mg/m^2^ continuous intravenous infusion over 46–48 h, all once every 2 weeks (mFOLFOX6; 28-day cycle)) or investigator’s choice SOC arm (mFOLFOX6 with or without bevacizumab (per prescribing instructions); irinotecan 165 mg/m^2^ intravenously, oxaliplatin 85 mg/m^2^ intravenously, leucovorin 400 mg/m^2^ intravenously and 5-FU 2,400 or 3,200 mg/m^2^ continuous intravenous infusion over 46–48 h, all once every 2 weeks (FOLFOXIRI; 28-day cycle) with or without bevacizumab (per prescribing instructions); oxaliplatin 130 mg/m^2^ intravenously once every 3 weeks (21-day cycle) and capecitabine 1,000 mg/m^2^ orally twice daily (days 1–14) (CAPOX) with or without bevacizumab (per prescribing instructions)). Following a protocol amendment, enrollment to the EC arm was stopped and patients were randomized 1:1 to the EC+mFOLFOX6 or SOC arms.

Randomization stratification factors were Eastern Cooperative Oncology Group performance status (0 versus 1) and region (US/Canada versus Europe versus Rest of World). Randomization was completed by Interactive Response Technology; sites contacted the Interactive Response Technology prior to the start of study intervention administration for each patient, and sites recorded the study intervention assignment on the applicable case report form required.

### Endpoints

The dual primary endpoints are objective response rate and progression-free survival by blinded independent central review between the EC+mFOLFOX6 and SOC arms, to be evaluated independently. Objective response rate is defined as confirmed complete response or partial response according to RECIST 1.1 (ref. ^31^) recorded from randomization until the date of the first documentation of progression of disease, death or start of subsequent anticancer therapy; both complete response and partial response must be confirmed by repeat assessments performed no less than 4 weeks after the criteria for response are first met. Progression-free survival is defined as the time from the date of randomization to the earliest documented disease progression per RECIST 1.1 (ref. ^31^) or death due to any cause.

The key secondary endpoint is overall survival between the EC+mFOLFOX6 and SOC arms, defined as the time from the date of randomization to death due to any cause. Other secondary endpoints include time to response, duration of response, progression after next line of therapy, patient-reported outcomes, pharmacokinetics, safety, and biomarker endpoints.

Adverse events were coded using Medical Dictionary for Regulatory Activities v26.1 (ref. ^32^), and severity of adverse events was graded using National Cancer Institute Common Terminology Criteria for Adverse Events v4.03 (ref. ^33^).

### Statistical analysis

The statistical analysis plan includes a detailed methodology for the statistical analyses of the data collected in this study. The sample size of 235 patients per arm was determined based on statistical assumptions for progression-free survival analysis. An overall one-sided alpha of 0.024 was unequally divided between the two dual primary endpoints.

One of the dual primary endpoints of objective response rate by blinded independent central review was analyzed in the objective response rate subset, comprised of the first 110 patients randomized in the EC+mFOLFOX6 arm and the SOC arm respectively. This sample size of 220 patients provided 90% power to test the odds ratio at a one-sided alpha of 0.001, assuming an objective response rate by blinded central review of 35% and 65% for the EC+mFOLFOX6 and SOC arms, respectively. Objective response rate was calculated along with the corresponding two-sided 95% Wilson score CI. The treatment effect between arms was measured using an odds ratio stratified by baseline stratification factors and its 99.8% and 95% CI and tested using a stratified Cochran–Mantel–Haenszel statistics at the one-sided alpha of 0.001.

Following a prespecified hierarchical testing procedure to control the family-wise type I error rate, an interim analysis of the key secondary endpoint of overall survival on all randomized patients would only be conducted if the dual primary endpoint of objective response by blinded independent central review is achieved, using a portion of the nominal one-sided alpha of 0.001. The treatment effect of overall survival was evaluated using a Cox proportional hazards model stratified by baseline stratification factors. The hazard ratio and its corresponding 95% CI were reported.

The other dual primary endpoint of progression-free survival was allocated one-sided alpha of 0.023 and will be analyzed once the required number of events has been observed.

Statistical analyses were performed using SAS version 9.4 or higher.

### Reporting summary

Further information on research design is available in the Nature Portfolio Reporting Summary linked to this article.

## Online content

Any methods, additional references, Nature Portfolio reporting summaries, source data, extended data, supplementary information, acknowledgements, peer review information; details of author contributions and competing interests; and statements of data and code availability are available at 10.1038/s41591-024-03443-3.

## Supplementary information

Supplementary Information Supplementary Table 1. List of investigators, protocol and statistical analysis plan.

Reporting Summary

## Figures and Tables

**Extended Data Table 1:**
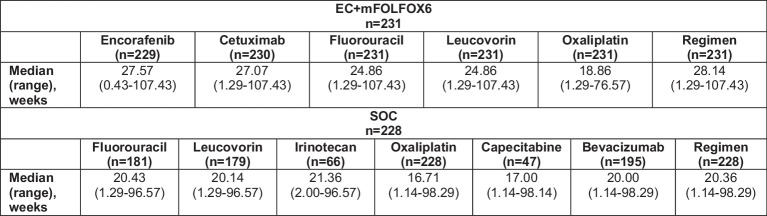
Median duration of treatment

EC+mFOLFOX6, encorafenib and cetuximab plus oxaliplatin, leucovorin, and 5-FU; SOC, standard of care.

**Extended Data Table 2:**
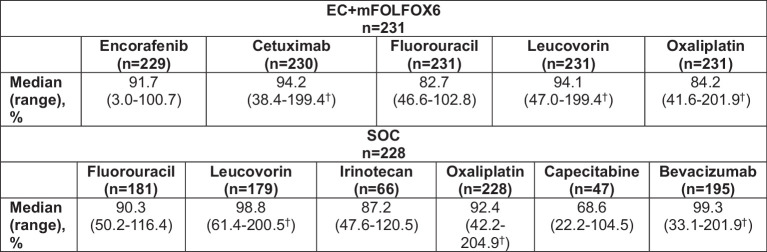
Relative dose intensity

EC+mFOLFOX6, encorafenib and cetuximab plus oxaliplatin, leucovorin, and 5-FU; SOC, standard of care. Relative dose intensity = 100 × (cumulative dose ÷ planned cumulative dose). Planned cumulative is sum of all doses assuming all doses are administered at the protocol-specified dose. ^†^ Patients who received only 2 doses and whose second dose was received prior to the planned date (still within protocol allowed window) leading to only 1 planned dose in the calculation.

**Extended Data Table 3:**
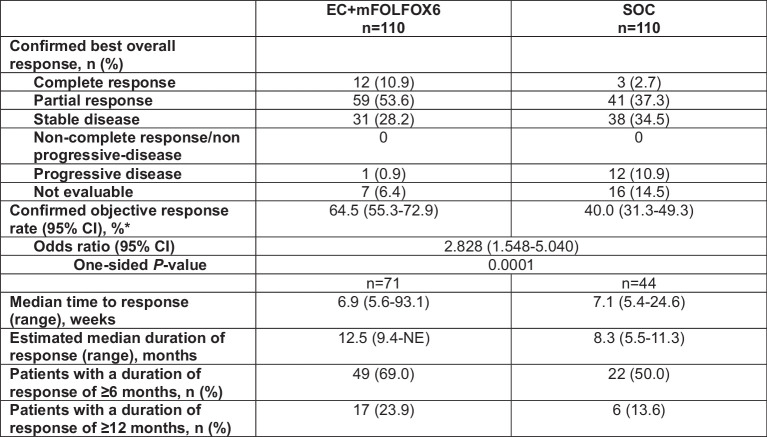
Confirmed objective response rate, time to treatment, and duration of response by investigator assessment

* Defined as complete response or partial response according to RECIST 1.1 recorded from the date of randomization until the date of the first documentation of progression of disease, death, or start of subsequent anticancer therapy; both complete response and partial response must be confirmed by repeat assessments performed no less than 4 weeks after the criteria for response are first met.

**Extended Data Table 4:**
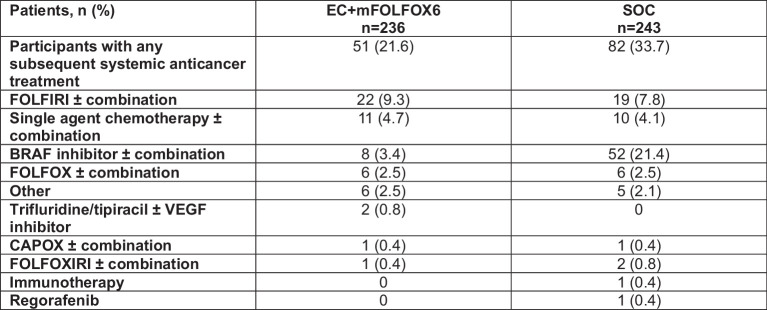
Subsequent systemic anticancer treatments by drug category

Classified using the WHODD GLOBALB3 MAR2023 coding dictionary.

**Extended Data Table 5:**
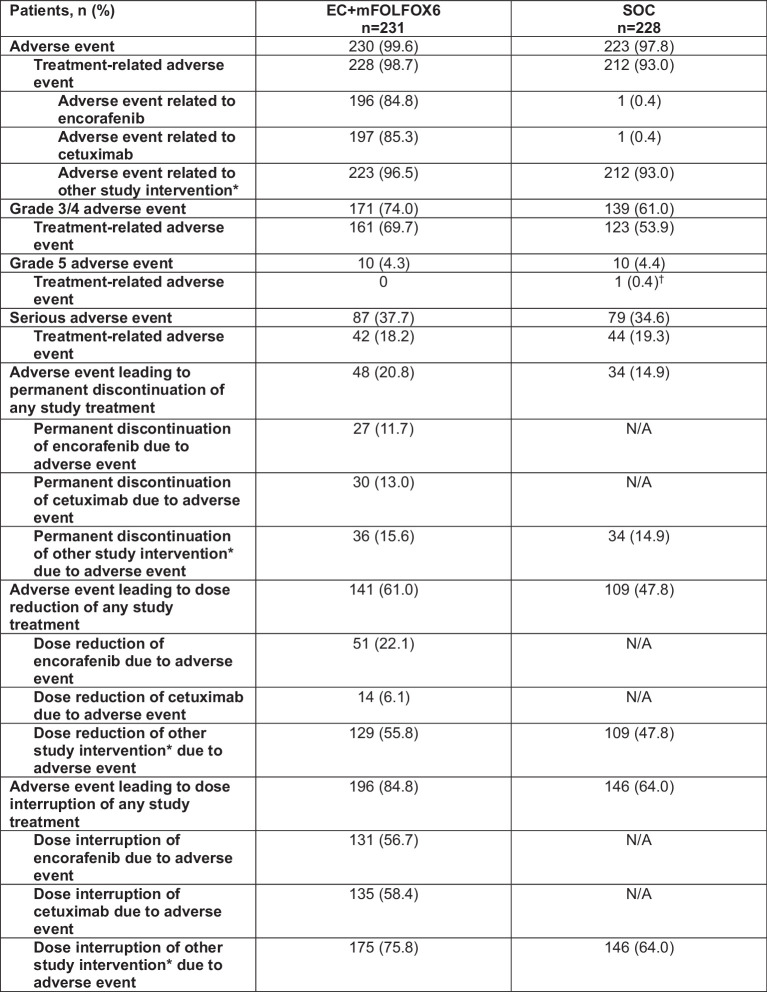
Safety summary

EC+mFOLFOX6, encorafenib and cetuximab plus oxaliplatin, leucovorin, and 5-FU; N/A, not applicable; SOC, standard of care. * Other study intervention includes: irinotecan, oxaliplatin, leucovorin or levo-leucovorin, fluorouracil (bolus or infusion), capecitabine, and bevacizumab (as appropriate for the treatment group). ^†^ Sepsis (preferred term).

**Extended Data Table 6:**
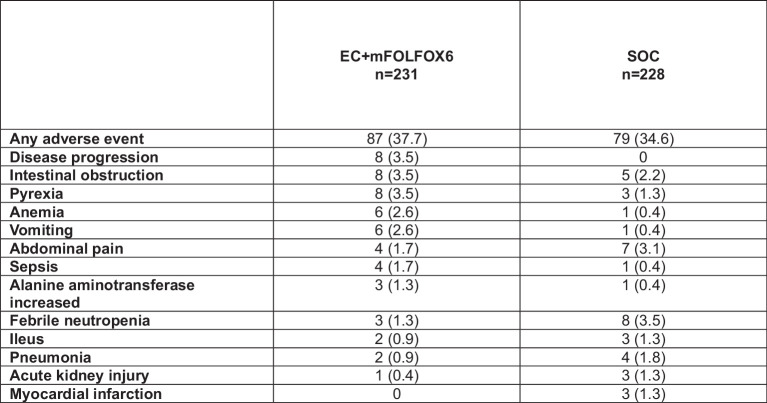
Most common serious all-causality treatment-emergent adverse events (≥1% of patients in any arm) by preferred term

EC+mFOLFOX6, encorafenib and cetuximab + oxaliplatin, leucovorin, and 5-FU; SOC, standard of care.

## Data Availability

The analyses in this paper were based on a data cutoff of 22 December 2023. Upon reasonable request and subject to review, Pfizer will provide the data that support the findings of this study. Subject to certain criteria, conditions and exceptions, Pfizer may also provide access to the related individual deidentified participant data from Pfizer-sponsored global interventional clinical studies conducted for medicines, vaccines and medical devices (1) for indications that have been approved in the United States and/or European Union or (2) in programs that have been terminated (that is, development for all indications has been discontinued). Pfizer will also consider requests for the protocol, data dictionary and statistical analysis plan. See https://www.pfizer.com/science/clinical-trials/trial-data-and-results for more information. Data may be requested from Pfizer trials 24 months after study completion. The deidentified participant data will be made available to researchers whose proposals meet the research criteria and other conditions, and for which an exception does not apply, via a secure portal. To gain access, data requestors must enter into a data access agreement with Pfizer.
