# Supplementary material for: Encorafenib, cetuximab and chemotherapy in BRAF-mutant colorectal cancer: a randomized phase 3 trial
Source: Nat Med. 2025 Jan 25;31(3):901–8. doi: 10.1038/s41591-024-03443-3 (PMC11922750; doi:10.1038/s41591-024-03443-3)
Supplement: Supplementary file 1 — Supplementary Table 1. List of investigators, protocol and statistical analysis plan. [file 41591_2024_3443_MOESM1_ESM.pdf]

nature medicine

Supplementary information https://doi.org/10.1038/s41591-024-03443-3

# Encorafenib, cetuximab and chemotherapy in BRAF-mutant colorectal cancer: a randomized phase 3 trial

In the format provided by the authors and unedited

## SUPPLEMENTARY INFORMATION

**TABLE OF CONTENTS**


| | |
|---|---|
| Table S1. Summary of Important Protocol Deviations | Page 2 |
| Listing of investigators | Page 3 |
| Listing of institutional review board or independent ethics committee | Page 25 |
| Protocol and statistical analysis plan | Page 44 |

**Table S1. Summary of Important Protocol Deviations**

| n (%) | EC+mFOLFOX6 (n=236) | SOC (n=243) |
|---|---|---|
| **Number of participants with at least one important protocol deviation** | 67 (28.4) | 60 (24.7) |
| **Concomitant medications** | 13 (5.5) | 2 (0.8) |
| **Inclusion/exclusion** | 13 (5.5) | 10 (4.1) |
| **Informed consent** | 6 (2.5) | 11 (4.5) |
| **Investigational product** | 20 (8.5) | 15 (6.2) |
| **Procedures/tests** | 9 (3.8) | 15 (6.2) |
| **Protocol specific discontinuation criteria** | 1 (0.4) | 0 |
| **Randomization** | 3 (1.3) | 1 (0.4) |
| **Safety reporting** | 12 (5.1) | 16 (6.6) |
| **Visit schedule** | 1 (0.4) | 0 |

**Listing of investigators**

<u>Argentina</u>

Martin Angel

Maria Baiud

Santiago Bella

Nicolas Castagneris

Matías Cortés

Diego Espinosa

Carlos Ferretti

Juan Manuel O Connor

Victor Pastrana

Yanina Pfluger

Ruben Segovia

Jeremias Sierra

Andrea Soria

Natalia Vega

Federico Waisberg

Melina Winocur

Juan Zarba

<u>Australia</u>

Anas Alawawdeh

Faria Arefin

Surein Arulananda

Nathan Bain

Catherine Berman

Amy Body

Matthew Burge

David Chan

Mei Mei Chan

Geoff Chong

Wei Chua

Michael Cilento

Stephen Clarke

Ciara Conduit

Elizabeth Connolly

Madhawa De Silva

Laura Depauw

Jayesh Desai

Connie Diakos

Melissa Eastgate

Michael Fernando

Tiffany Foo

Sophia Frentzas

Chun Gan

Barbara Geerinckx

Aisha Ghaus

Sanjeev Gill

Aaron Hansen

Marion Harris

Eliza Hawkes

Andrew Haydon

Alison Hiong

Nadia Hitchen

Haakan Jakobsson

Anadil Javaid

Michael Jefford

Helen Ke

Richard Kelly

Andrea Knox

Marissa Lam

David Lau

Sheau Wen Lok

Caroline Lum

Lee-Jen Luu

Kate Mahon

Sarah Maloney

Benjamin Markman

Jared Mathai

James McCracken-Skeggs

Michael Michael

William Mullally

Felicity Murphy

Mahendra Naidoo

Mark Nalder

Robert Needleman

Gary Ng

Weng Ng

Mike Nguyen

Kenneth O'Byrne

Christian Orlowski

Nick Pavlakis

Ashley Pheely

Oliver Piercey

Timothy Price

Ajay Raghunath

Rachel Raju

Hayley Roberts

Kate Roberts

Aflah Roohullah

Tahlia Scheinberg

Eva Segelov
Annabel Smith
Andrew Strickland
Sarah Sutherland
Lok-man Tam
Joanne Tang
Niall Tebbutt
Jeanne Tie
Yat Hang To
Yoko Tomita
Amanda Townsend
Avraham Travers
Iris Tung
Cassie Turner
Anthony Uccellini
Imogen Walpole
Sarah Williams
Edbert Wong
Hui-Li Wong
David Wyld
Kyung Ha (Kathy) You
John Zalcberg
Farzana Zaman

Belgium

Antoon Billiet
Nathalie Blockx
Ana-Maria Bucalau
Jean-Luc Canon
Javier Carrasco
Alix Colassin
Joëlle Collignon
Francois Dall'Armellina
Astrid De Cuyper
Jeroen Dekervel
Megane Deliège
Anne Demols
Chloé Denis
Alix Devaux
France Gay
Marco Gizzi
Elodie Gonne
Brigitte Honhon
Hassan Kalantari
Molka Kammoun
Dominique Korman
Virginie Labille
Marie Lecocq
Sarah Lefevre
Catherine Loly
Christophe Lonchay
Laurence Lousberg
Aurélie Louvet
Laura Mans
Nathalie Marchal
Sacha Mignon
Anne-Catherine Moreau
Aurélie Poncin
Charles Pottier
Gertjan Rasschaert
Anne Sacre
Dirk Schrijvers
David Schroder
Christophe Severi
Isabelle Sinapi
Sabine Tejpar
Wesley Teurfs
Florence Troisfontaine
Eric Van Cutsem
Marc Van Den Eynde
Frank Van Fraeyenhove
Filip Van Herpe
Jean-Luc Van Laethem
Simon Van Wambeke
An Vandebroek
Alexander Vanden Bulcke
Muriel Vandervaeren
Philippe Vergauwe
Joanna Vermeij
Vincent Verschaeve
Gontran Verset
Chris Verslype
Manon Wick

Brazil

Kathia Abdalla
Maira Abreu
Luciana Alban
Gabriela Andrade
Camila Araújo
Paula Bagatini
Giovanni Bariani
Juliana Bonometto
Giuliano Borges
Arinilda Campos Bragagnoli
Maria Ignez Braghiroli
Erika Brandao
Leandro Brust
Allyne Carneiro Cagnacci
Ana Suellen Barroso Carneiro
Élvis Cassol
Juliano Ce Coelho

Leticia Cericato

Fernanda Correa

Leticia Costa

Daniel Cubero

Carini Dagnoni

Cristiano De Assis Assis Assis

Sergio Jobim De Azevedo

Aline De Barroso

Gilberto De Castro Junior

Murilo De Mello

Lucas Di Bello Zanini

Jose Dos Zanini Junior

Marcelo Drude

Jamille Dutra

Marilia e Silva

Grazielle Felippe

Camila Ferrari

Carla Ferreira

Leonardo Fonseca

Larissa Furlan

Livia Gravina

João Vitor Gregório

João Daniel Guedes

Daniel Kischinhevsky

Victor Marcondes Lopes Dos Santos

Suelen Martins

Dayana Mendes Ribeiro

Ana Verena Miranda

Camila Moniz

Daniela Nebuloni Nagy

Thamires Oliveira Silva

Monica Padoan

Rodrigo Pereira

Gustavo Pinto

Taymara Raizer

Sandro Reichow

Marcos Renni

Breno Rocha

Pedro Rubini Liedke

Patricia Santi

Florinda Santos

Cintia Schroeder

Rafael Seewald

João Soler

Juliana Souza

Marina Topanotti

Bruno Cezar Uchôa Júnior

Claudia Vaz de Melo Sette

Carolina Victor

Lucas Henrique Vidoto

Alana Visentainer

Bulgaria

Mohamed Alhalabi

Marin Angelov

Jeliazko Arabadjiev

Tatyana Chalakova

Aynura Changalova

Ivan Donev

Sonya Draganova

Nadzhie Emin

Radostina Gencheva

Aneliya Hubenova

Bozhidar Iliev

Rumyana Ilieva

Anika Ivanova

Teodora Karanikolova

Ahmed Kontilev

Petya Kraleva

Vasilena Kumanova-Chocheva

Dimo Manov

Velko Minchev

Snezhina Nedeva

Mila Petrova

Stefka Petrova

Bonka Popova

Rossitza Ruseva

Tsvetan Tatarov

Margarita Taushanova-Hadzhieva

Stefan Todorov

Antoaneta Tomova

Georgi Zhbantov

Canada

Omar Abdelsalam

Nathalie Aucoin

Gerald Batist

Scott Berry

Alain Bestavros

Jim Biagi

Christopher Booth

Daniel Breadner

Najwa Buhlaiga

Kelvin Chan

Neil Chua

Reginald Comeau

Sabrina Demers

Scot Dowden

Emmanuelle Dugas-Bourdages

Jacob Easaw

Karin Hahn

Nazik Hammad

Hatim Karachiwala

Hatim Karachiwala

Safiya Karim

Elie Kassouf

Petr Kavan

Karen King

Sheryl Koski

Vanessa Krause

Carole Lapointe

Catherine Lavoie

Richard Lee-Ying

John Lenehan

Anthony Lott

Sasha Lupichuk

Kim Ma

Ashley Marton

Jose Monzon

Karen Mulder

Veera Panuganty

Ashley Paul

Sheron Perera

Julie Price Hiller

Michael Raphael

Jennifer Rauw

Michael Sanatani

Michael Sawyer

Jennifer Spratlin

Paul Stewart

Danielle Talbot

Vincent Tam

Patricia Tang

Anna Tomiak

Christine Tremblay

Elena Tsvetkova

Mark Vincent

Stephen Welch

Xiaofu Zhu

China

Yue Cai

Xinyi Cai

Yanming Cao

Yanshuo Cao

Wuteng Cao

Jinjia Chang

Chunxiao Chang

Zhiyu Chen

Dianke Chen

Hua Chen

Xingyue Chen

Xianshuo Cheng

Jie Da

Yanhong Deng

Juan Ding

Honghua Ding

Jing Ding

Jianhua Dong

Bin Du

Xingyu Feng

Huiqun Fu

Xinyan Gao

Pin Gao

Yi Gao

Yawen Gao

Qirong Geng

Shilei Geng

Hongli Gu

Sijia He

Peifeng Hou

Hanguang Hu

Huabin Hu

Xueqing Hu

Jing Hu

Chenghui Huang

Mingzhu Huang

Le Huang

Yingying Huang

Yu Huang

Jinling Jiang

Tao Jiang

Zhichao Jiang

Li Jiang

Long Jin

Qiao Jin

Yongdong Jin

Xuan Jin

Jinhuo Lai

Yanqin Lan

Shuangyi Lei

Jian Li

Wenhua Li

Xiang Li

Fangqian Li

Shanshan Li

Weiwei Li

Qi LI

Yunfeng Li

Qiang Li

Ming Li
Xin Li
Yongqiang Li
Qian Li
Yan Li
Yongsheng Li
LuChun Li
Yong Li
Guanzhong Liang
Sina Liao
Xiaoli Liao
Xiaoyan Lin
Mengxin Lin
Yan Lin
Jiayu Ling
Jing Liu
Chang Liu
Qing Liu
Chuan Liu
Ping Liu
Xianling Liu
Bin Liu
Qin Liu
Ying Liu
Song Liu
Jianlin Long
Ming Lu
Jin Lu
Min Luo
Fei Lv
Zejian Lv
Ning Ma
Kai Mei
Zuoxing Niu
Yuliang Pan
Xi Pan
Zhangchi Pan
Dingguo Pan
Bin Peng
Honghua Peng
Zhi Peng
Wanren Peng
Xiaoping Qian
Yu Qiao
Hong Qiu
Lei Qiu
Chengfang Shangguan
Lin Shen
Songfei Shen
Feifei Shen
Tao Shen
Min Shi
Chunmei Shi
Zewen Song
Yongkun Sun
Meili Sun
Guoping Sun
Xueyuan Tang
Qingnan Tang
Youqun Tang
Yuanyuan Tang
Yan Teng
Qi Wang
Xicheng Wang
Zhenghang Wang
Chenchen Wang
Yao Wang
Xinli Wang
Han Wang
Huaqing Wang
Jingjue Wang
Fan Wang
Shuhang Wang
Wei Wang
Xiaoyun Wang
Ying Wang
Chunmei Wang
Ting Wang
Wen Wei
Shanshan Weng
Xiaojiao Weng
Bin Wu
Riping Wu
Tao Wu
Shikai Wu
Zhijuan Wu
Cuifeng Xia
Xiaoyu Xie
Mingzhi Xie
Guiyuan Xie
Wei Xiong
Shuanglong Xiong
Ting Xu
Zhibin Yang
Quan Yang
Guangyu Yao
Xiang Yu
Hongen Yu
Kun Yu
Ying Yuan
Hong Yuan
Xianglin Yuan

Dandan Yuan
Xianghua Zeng
Xiaohui Zhai
Xi Zhang
Jun Zhang
Zhen Zhang
Yi Zhang
Jun Zhang
Huan Zhang
Xiaotian Zhang
Jieyun Zhang
Mingsheng Zhang
Yan Zhang
Jianwei Zhang
Haiyan Zhang
Shuai Zhang
Xuan Zhang
Hongtao Zhang
Guodong Zhang
Wen Zhang
Yinjie Zhang
Li Zhang
Jinyan Zhang
Yumei Zhang
Nan Zhang
Ying Zhang
Qun Zhang
Liqin Zhao
Yunbo Zhao
Jianwei Zheng
Dongta Zhong
Lixing Zhong
Miner Zhong
Fei Zhou
Aiping Zhou
Jianwei Zhou
Yuxing Zhu
Xishan Zhu
Yanyan Zhu
Jianling Zou
Jiayun Zou

Czech Republic

Katarina Adamova
Lenka Balazova
Tomas Buchler
Barbara Donocikova
Michal Eid
Petra Beran Holeckova
Stanislav John
Hana Kalabova
Jindrich Kopecky
Ivana Kosikova
Zdenek Kral
Eugen Kubala
Ondrej Kubecek
Zdenek Linke
Bohuslav Melichar
Lenka Ostrizkova
Pavel Pacas
Jana Prausova
Peter Priester
Martina Spisarova
Hana Studentova
Anezka Zemankova
Lucie Zitnanska
Kamila Zvackova

Denmark

Rana Bahij
Signe Christensen
Lise Eckhoff
Mathilde Ejlsmark
Torben Hansen
Birgitte Havelund
Jon Henriksen
Helle Jensen
Lars Henrik Jensen
Annette Kodahl
Merete Krogh
Morten Ladekarl
Jim Larsen
Thea Mattsson
Rikke Oksen
Rene Olesen
Lone Petersen
Per Pfeiffer
Laurids Poulsen
Camilla Qvortrup
Lisbeth Riber
Line Tarpgaard
Jakob Vasehus Schou
Lise Ventzel
Mette Yilmaz

Finland

Tuomo Alanko
Päivi Halonen
Eetu Heerva
Antti Hosio
Riikka Huuhtanen
Raija Kallio
Justina Kaukola
Kaisa Lehtomäki
Siru Makela

Olga Maslennikova

Riina Ollikainen

Pia Osterlund

Niina Paunu

Kalevi Pulkkanen

Raija Ristamaki

Veera Salminen

Tapio Salminen

Jorma Sormunen

Sanni Tulokas

Heli Vajavaara

Germany

Salah-Eddin Al-Batran

Tiago Almeida Valadas de Castro

Torben Altmann

Stefan Böck

Yasemin Dogan Akyildiz

Dorothea Fasslrinner

Gunnar Folprecht

Dirk Forstmeyer

Eray Goekkurt

Thorsten Goetze

Michael Haas

Timorshah Habibzada

Ulrich Hacker

Sophia Heinrich

Meinolf Karthaus

Jakob Kather

Benjamin Kobitzsch

Claus-Henning Koehne

Anke Kroecher

Anette Kruempelmann

Kristina Lerch

Kristina Lerch

Cornelia Link-Rachner

Florian Lordick

Andrea Mayer

Marika Mende

Anke Mütherig

Peter Reichardt

Annette Reichardt

Stephan Richter

Anna Saborowski

Harald Schaefer

Ulrike Schneider

Ingo Schwaner

Alexander Stein

Gertraud Stocker

Ingo Tamm

Ruth Thole

Tina Thomas

Karolin Trautmann-Grill

Ulrike Ubbelohde

Benjamin Unger

Nils Utz

Janna-Lisa Velthaus

Arndt Vogel

Martin Wermke

Elisabeth Wetzel

Thomas Wirth

Nicolas-Stephan Ziegenhagen

Josip Zovko

India

Prabhat Bhargava

Sumit Goyal

Sachin Hingmire

Linu Hooda

Sandeep Jasuja

Dhananjay Kelkar

Rahul Kulkarni

Aditi Mittal

Viraj Nevrekar

Vikas Ostwal

Anant Ramaswamy

Nirmal Raut

Sujay Srinivas

Kailash Surnare

Shivashankara Swamy Mathighatta Shivarudraiah

Tushar Vishvasrao Patil

Italy

Massimo Aglietta

Alessio Amatu

Alicia Garcia Arias

Maria Banzi

Chiara Baratelli

Katia Bencardino

Lavinia Benini

Francesca Bergamo

Raffaella Bitossi

Erica Francesca Bonazzina

Maria Pia Brizzi

Delia Campanella

Giovanni Cappello

Francesco Caputo

Chiara Alessandra Cella

Davide Ciardiello

Davide Ciardiello

Tiziana Cipani

Angela Damato
Francesca Daniel
Silvia Fanello
Elisabetta Fenocchio
Renato Ferraris
Renato Ferraris
Lorenzo Gervaso
Laura Giannetta
Francesco Iachetta
Rossana Intini
Eleonora Lai
Mario Larocca
Tiziana Latiano
Michela Libertini
Sara Lonardi
Evaristo Maiello
Chiara Manai
Erika Martinelli
Giulia Martini
Luca Mazzarella
Maria Morritti
Sabina Murgioni
Luciano Nanni
Stefania Napolitano
Floriana Nappo
Silvia Novello
Adele Orlando
Oliviero Ostellino
Michele Panebianco
Carmine Pinto
Elio Pizzutilo
Alessandra Prete
Andrea Pretta
Valeria Pusceddu
Marco Puzzoni
Patrizia Racca
Mario Domenico Rizzato
Maria Grazia Rodriquenz
Andrea Sartore-Bianchi
Giorgio Scagliotti
Mario Scartozzi
Ilaria Schiavetto
Salvatore Siena
Francesca Spada
Dario Spanu
Francesco Spina
Federica Tosi
Teresa Troiani
Maria Zampino
Alberto Zaniboni
Pina Ziranu

<u>Japan</u>

Masaru Abe
Yumi Amano
Yusuke Amanuma
Masashi Ando
Yu Aoki
Hiroyuki Arai
Kohei Arimizu
Akinori Asagi
Masahiro Asari
Masako Asayama
Takahito Awatsu
Hideaki Bando
Masashi Bungo
Keigo Chida
Akihiko Chida
Keisho Chin
Katsuya Deguchi
Tadamichi Denda
Toshihiko Doi
Ayako Doi
Taito Esaki
Yoji Fuchizaki
Takatsugu Fujii
Fumie Fujisawa
Koshiro Fukuda
Tsuyoshi Fukumoto
Shota Fukuoka
Kunihiro Fushiki
Masahiro Goto
Kana Hagihara
Atsushi Hamabe
Tetsuya Hamaguchi
Yasuo Hamamoto
Satoshi Hamauchi
Fumiyasu Hanamura
Hiroki Hara
Shuichiro Hara
Kentaro Harada
Kazuaki Harada
Naotsugu Haraguchi
Hiroko Hasegawa
Tsuyoshi Hata
Hideyuki Hayashi
Hidetoshi Hayashi
Kaori Hino
Hidekazu Hirano
Yasumitsu Hirano
Kenro Hirata
Esuteru Hirokawa
Shuichi Hironaka

Toshiharu Hirose
Kazunori Honda
Yoshiki Horie
Sara Horie
Yosuke Horita
Waki Hosoda
Takashi Ichimura
Arika Ida
Kenta Iguchi
Go Ikeda
Takako Ikegami
Suzuka Imanishi
Hiroshi Imazeki
Yoshitaka Inaba
Chiaki Inagaki
Tomonori Inoue
Koichi Ishida
Mikiya Ishihara
Genichiro Ishii
Takahiro Ishii
Shintaro Ishikawa
Yasunobu Ishizuka
Kohsuke Isomoto
Nobuhito Ito
Ken Ito
Satoru Iwasa
Tsutomu Iwasa
Naoki Izawa
Amane Jubashi
Toru Kadono
Shigenori Kadowaki
Hayato Kaida
Takeshi Kajiwara
Daisaku Kamiimabeppu

Tomoyasu Kamiya
Shiho Kaneko
Hiroaki Kanemura
Ken Kato
Takeshi Kato
Kenji Kawai
Takeshi Kawakami
Hisato Kawakami
Yasuyuki Kawamoto
Yusuke Kawanaka
Akihito Kawazoe
Shotaro Kishimoto
Masatoshi Kitakaze
Saya Kitashiro
Akinori Kobayashi
Hiroyuki Kodama
Kunishige Koga
Shigehiro Koganemaru
Motohiro Kojima
Takashi Kojima
Yoshiki Kojitani
Yoshito Komatsu
Masato Komoda
Azusa Komori
Daisuke Kotani
Hiroshi Kotani
Yasutoshi Kuboki
Yohei Kubota
Toshihiro Kudo
Ryosuke Kumanishi
Yosuke Kumekawa
Takashi Kurosaki
Toshiki Masuishi
Yuki Matsubara

Chu Matsuda
Takashi Matsumura
Hiroya Matsuoka
Tomohiro Matsushima
Yoshiaki Mihara
Yo Mikayama
Keiko Minashi
Saori Mishima
Seiichiro Mitani
Yu Miyashita
Masaaki Miyo
Norikatsu Miyoshi
Takuro Mizukami
Masashi Morimachi
Yuichi Motoyama
Shuichi Murata
Kozue Murayama
Kei Muro
Yusuke Nagata
Kazuhiko Nakagawa
Hiromichi Nakajima
Yoshiaki Nakamura
Takeaki Nakamura
Akio Nakasya
Akinobu Nakata
Izuma Nakayama
Tomohiro Nakayama
Taiko Nakazawa
Shigeki Nanjo
Yukiya Narita
Masanori Nasu
Naohiro Nishida
Norifumi Nishide
Hirotaka Nishihara

Tomohiro Nishijima
Ari Nishimura
Junichi Nishimura
Tomohiro Nishina
Minako Nishio
Akihiro Nishiyama
Kazuki Nozawa
Suguru Nukada
Koji Numata
Takatsugu Ogata
Takashi Ogimi
Mariko Ogura
Yoshinori Ohno
Ukyo Okazaki
Naoto Okazaki
Natsuko Okita
Yuta Okumura
Masaki Okura
Sachiyo Onishi
Yukari Ono
Yusuke Onozawa
Akira Ooki
Mao Osaki
Kotoe Oshima
Hiroki Osumi
Tomoyuki Otsuka
Toru Otsuru
Masato Ozaka
Yuki Saito
Rika Saito
Hiroyuki Sakaguchi
Chihiro Sakaguchi
Daisuke Sakai
Tomoki Sakakida

Yu Sakano
Megumi Sasaki
Seiya Sato
Shigeki Sato
Chihiro Sato
Taroh Satoh
Yoshihiko Segawa
Fukutaro Shimamoto
Satoshi Shimizu
Satoko Shimizu
Keitaro Shimozaki
Eiji Shinozaki
Manabu Shiozawa
Hiromichi Shirasu
Kohei Shitara
Hirokazu Shoji
Eiichiro So
Toshinori Sueda
Naotoshi Sugimoto
Yu Sunakawa
Satoshi Suzuki
Chiaki Suzuki
Yuko Suzuki
Shinichiro Suzuki
Masahiro Tajika
Akihiro Takagi
Takayuki Takahama
Daisuke Takahari
Hidekazu Takahashi
Naoki Takahashi
Yusuke Takahashi
Toshiaki Takakura
Atsuo Takashima
Hiroyuki Takeda

Shogo Takei
Norihiko Takemoto
Shinji Takeuchi
Masaki Takinami
Tsutomu Tanaka
Kaoru Tanaka
Mikako Tanba
Hiroya Taniguchi
Hiroya Taniguchi
Junko Tanizaki
Koki Terakawa
Tetsuji Terazawa
Akiko Todaka
Kazuhiro Togasaki
Takuya Tokunaga
Shinji Tokuyama
Kai Tsugaru
Takahiro Tsushima
Yoshiyasu Uchida
Mamoru Uchiyama
Shohei Udagawa
Mamoru Uemura
Kumiko Umemoto
Masahiro Umezu
Shinpei Ushiyama
Munehiro Wakabayashi
Takeru Wakatsuki
Ryosuke Watanabe
Satomi Watanabe
Toshinari Yagi
Keisaku Yamada
Toshifumi Yamaguchi
Kensei Yamaguchi
Toshifumi Yamaguchi

Takahiro Yamamura

Eiki Yamasaki

Keishi Yamashita

Kentaro Yamazaki

Kiminori Yanagisawa

Hirofumi Yasui

Masayoshi Yasui

Tomoyuki Yokose

Tomoya Yokota

Kimio Yonesaka

Takeshi Yoshida

Takako Yoshii

Ayumu Yoshikawa

Megumi Yoshimatsu

Takayuki Yoshino

Koichiro Yoshino

Hiroki Yukami

Hiroki Yukami

Satoshi Yuki

Republic of Korea

Jihong Bae

Joong Bae Ahn

Seung-Hoon Beom

Dong Bok Shin

Yongjun Cha

Songji Choi

Jeong Eun Kim

Jong Gwang Kim

Sae-Won Han

Myung Han Hyun

Sang Hee Cho

Sung Hee Lim

Jin Ho Baek

Yeul Hong Kim

Jun-Eul Hwang

Ja Hyun Yeo

Seok Jae Huh

Lee Jeeyun

Hyehyun Jeong

Hyun Jeong Shim

Sun Jin Sym

Ik Joo Chung

Sang Joon Shin

Minsu Kang

Moon Ki Choi

Won Ki Kang

Hyunwook Kim

Jinyong Kim

Jwahoon Kim

Tae-Yong Kim

Woo Kyun Bae

Dae-Won Lee

Jiwon Lee

Kyung-Hun Lee

Soohyeon Lee

Yongho Noh

Do-Youn Oh

Joon Oh Park

Changhee Park

Joo-Hwan Park

Taekeun Park

Woochan Park

Yeonsoo Park

Ah Reum Lim

Hye Ryeon Kim

Yong Sang Hong

Han Sang Kim

Jeongmin Seo

Junghoon Shin

Young Suk Park

Ye Sul Jeung

Seung Tae Kim

Tae Won Kim

Byung Woog Kang

Jieun Yang

Ji Yeon Baek

Ho Yeong Lim

Hanbaek Yi

Jung Yong Hong

Sung Yong Oh

Jeesun Yoon

Jeonghwan Youk

Sun Young Kim

Mexico

Sergio Buenaventura Cisneros

Roberto Castro Zarate

Nevid Ferrel

Aurora Hernández Castillo

Jose Luis Martinez Lira

Roberto Romero

Netherlands

Annemieke Cats

Myriam Chalabi

Geert Cirkel

Geert.Jan Creemers

Corine De Graaf

Anne-Joy De Graan

Judith De Vos

Joeri Douma

Boudewijn Dullens

Mira Franken

Marnix Geukes Foppen

Ruben Goedegebuure

Kaitlyn Goey

Cecile Grootscholten

Wesley Hartman

Helgi Helgason

Robert Jan Kwakman

Frank Jeurissen

Miriam Koopman

Laetitia Lamberts

Loes Latten

Lieke Razenberg

Jeanine Roodhart

Marijke Ros

Dirkje Sommeijer

Mirte Streppel

Anna Thijs

Jansen Tonneke Beijers

Liselot Valkenburg-van Iersel

Nico Van Blijderveen

Irene Van Hellemond

Loes Verhoeven

Jeroen Vermeulen

Kathelijn Versteeg

Marieke Vollebergh

Birgitte Vriens

New Zealand

Laura Camburn

Matthew Cross

Dragan  Damianovich

Jonathan Davis

Sanjeev Deva

Claire Harris

William Harris

Michelle Head

Nadia Hitchen

Siti Aisyah Haji Ibrahim

Weng John Mak

Anna Manning

Angela Mweempwa

Richard North

William Thompson

Jane Yeojeong So

Norway

Elin Aamdal

Kristine Aasebø

Nikolai Andresen

Maria Birkenes

Helene Eikenes

Kjersti Elvestad Hestetun

Ida Faksvåg Andersen

Herish Garresori

Geir Hjortland

Eva Hofsli

Marie Johnsen

Janne Kjersem

Are Kristensen

Tormod Kyrre Guren

Inger Løes

Camilla Norbakk Sævereide

Marta Nyakas

Jan-Åge Olsen

Sara Ribe

Andreas Rossevold

Lone Scibstad

Jens-Erik Slagsvold

Jørgen Smeby

Tor Magnus Smedman

Knut Smeland

Halfdan Sorbye

Linn Tetlie

Maria Thomsen

Poland

Lukasz Glogowski

Magdalena Jakubowska

Boguslawa Karaszewska

Joanna Kiszka

Dawid Kostiukow

Katarzyna Nazim-Kociorska

Monika Nowaczyk

Ewa Nowakowska-Zajdel

Pawel Perkowski

Dariusz Sawka

Pawel Staszewski

Pawel Tran Dinh

Joanna Trella

Piotr Witasik

Andrzej Witkos

Joanna Wojcik-Tomaszewska

Artur Zielinski

Russia

Polina Balabanova

Olga Berseneva

Dmitriy Boiko

Ekaterina Bolotskaya

Antonina Cheremnikh

Daria Eliseeva

Sergei Evstifeev
Natalya Fadeeva
Anastasia Fatyanova
Alexey Fedoseev
Vitaliy Fokin
Jaroslav Galahov
Irina Grebtsova
Anastasia Gritsenko
Airat Imaev
Nursultan Ismanbaev
Almaz Iunusov
Artem Ivashin
Nikolay Kislov
Maxim Lavrentyev
Viktoria Lemekhova
Oleg Lipatov
Natalya Lozovaya
Alexander Muravyov
Mahabbat Murzalina
Svetlana Odintsova
Lianna Pavlova
Konstantin Penkov
Natalya Pinchuk
Albert Pirmagomedov
Vladislav Pisarev
Irina Radyukova
Natalia Rats
Irina Rozhkova
Anastasiya Rusanova
Marina Sekacheva
Vadim Shirinkin
Marina Silkina
Pavel Skopin
Natalia Smirnova
Elena Sotskova
Zaur Sultanov
Maria Sviridenko
Andrei Ulitin
Magaripa Urtenova
Aleksey Vasiliev
Alexandr Vasiliev
Georgy Vesnushkin
Felix Zainullin

Slovakia

Tatiana Albertova
Igor Andrasina
Katarina Bahnova
Juraj Beniak
Andrea Cipkova
Miriam Hancinova
Matej Hrncar
Marian Kakalejcik
Lenka Medvecova
Zuzana Minarikova
Natalia Pazderova
Eleonora Pihurikova
Stefan Porsok
Lenka Sako
Tomas Salek
Jan Slopovsky
Benjamin Spanik
Anna Svidranova
Vanda Usakova
Eva Zomborska
Peter Zuzák

South Africa

Panayiotis Anastasiades
Sakina Bawa
Khabane Chabane
Corlia Coetzee
Graham Cohen
John Crockett
Garth Davids
Georgia Demetriou
Louis Dupper
Courtney Farland
Doné Fourie
Jill Harris
Gregory Hart
Allana Hemus
Ryno Holtshousen
Natalie Irwin
Conrad Jacobs
Natalie Jonas
Canzonette Kempen
Jorn Malan
Jean-Marc Maurel
Yusuf Mayet
Georgina McAdam
Samuel Molepo
Shun Moodley
Johannes Raats
Paul Ruff
Elizabeth Schoeman
Karishma Singh
Coenraad Slabber
Judy Sparg
Dineo Tshabalala
Ananda Vorster
Ashraf Wadee
Cathryn Walton

Spain

Marina Meri  Abad

Jorge Adeva Alfonso

Iker Aguilar Caballero

Vicente Alonso-Orduña

Maria Angeles Vaz Salgado

Beatriz Anton Pascual

Elena Asensio Martinez

Iosune Baraibar Argota

Susana Barriendos Sanz

Carmen Blanco Abad

Elena Brozos-Vazquez

Pilar Aitana Calvo Ferrandiz

Sonia Candamio Folgar

Alfredo Carrato Mena

Juan Catoya Villa

Elena Corral de la Fuente

Belén De Frutos Gonzalez

Elena Elez Fernandez

Paula Espinosa Olarte

Maria Pilar Felices

Amaya Belén Fernández Díaz

Mar Fuentes Losada

Raquel Fuentes Mateos

Inmaculada Gallego Jimenez

Javier Gallego Plazas

Maria Rosa Gallego Sanchez

Valentina Gambardella

Pilar Garcia Alfonso

Rocio Garcia Carbonero

Lucio Ghiglione

Mireia Gil

Paula Gomila Pons

Reyes Gonzalez Exposito

Nerea Gonzalez Garcia

Cristina Gravalos Castro

Carmen Guillen Ponce

Ana Herrero Ibanez

Maria Huerta Alvaro

Alberto Indacochea Cusirramos

Maria Luisa Limon Miron

Miriam Lobo de Mena

Federico Longo Muñoz

Victoria López Gómez

Amelia Lopez Ladron

Rafael López López

Inigo Martinez Delfrade

Mercedes Martínez Villacampa

Joan Maurel Santasusana

Andrea Modrego Sanchez

Reinaldo Moreno Zambrano

Blanca Moron Garcia

Blanca Moron Garcia

Andres Jesús Munoz Martin

Helena Oliveres

Mayra Orrillo Sarmiento

Laura Ortega Moran

Martin Perez Martelo

David Pesantez Coronel

Eduardo Polo Marques

Pablo Reguera Puertas

Maria Reyes Ferreiro Monteagudo

Maria Riesco Martinez

Luis Robles Díaz

Desamparados Roda

Mercedes Rodriguez Garrote

Jose Ramon Rodríguez Mowbray

Francisco Javier Ros Montana

Susana Rosello Keranen

Jose Carlos Ruffinelli Rodriguez

Juan Ruiz Banobre

Maria Jose Safont Aguilera

Francesc Salva Ballabrera

Maria San Roman Gil

Cristina Santos

Lucas Sanz Monge

Nadia Saoudi Gonzalez

Juan Jose Serrano Domingo

Gemma Soler

Javier Soto Alsar

Josep Tabernero Caturla

Maria Tobeña Puyal

Gabriela Torres Pérez-Solero

Manuel Valladares Ayerbes

Vanesa Varela

Francisca Vazquez

Rocio Velasco

Alba Viala Monleon

Yolanda Vidal

Maria Luisa Villamayor Delgado

Sweden

Sandra Andersson

Daniel Brattstrom

Nina Fanni

Jan-Erik Frodin

Sofia Heyman

Mia Karlberg

Henning Karlsson

Tanweera Khan

Leif Klint

Paulina Krywda

Birgitta Lindh

Ingrid Ljuslinder

Ylva Naeser

Marjut Niinivirta

Peter Nygren

Mats Perman

Cecilia Remling

Johanna Sand

Carl-Henrik Shah

Gustav Silander

Taiwan

Ching-Fu Chang

Dwan-Ying Chang

Sheng-Chi Chang

Yee Chao

Chao-Hsun Chen

Hung-Chang Chen

Jen-Shi Chen

Kuo-Hsing Chen

Ming-Huang Chen

Po-Chuan Chen

Shang-Hung Chen

Shang-Wen Chen

Yen-Hsun Chen

Yi-Chang Chen

Ann-Lii Cheng

Wen-Chi Chou

Jhe-Cyuan Guo

Sheng-Yen Hsiao

Ming-Hao Hsieh

Chih-Hung Hsu

Chiun Hsu

Hung-Chih Hsu

Ching-Wen Huang

Ta-Chen Huang

Wen-Kuan Huang

Wen-Tsung Huang

Ying-Tzu Huang

Yi-Ping Hung

Wan-Chen Kao

Tao-Wei Ke

Li-Jen Kuo

Kuan-Der Lee

Rheun-Chuan Lee

Yi-Hsin Liang

Been-Ren Lin

Bo-Wen Lin

Che-Hung Lin

Cheng-Yao Lin

Chien-Liang Lin

Peng-Chan Lin

Zhong-Zhe Lin

Chien-An Liu

Li-Chun Lu

Meng-Ting Peng

Yu-Yun Shao

Ying-Chun Shen

Wei-Chih Su

Yung-Yeh Su

Hao-Wei Teng

Hsiang-Lin Tsai

Yuan-Yao Tsai

Chao-Jung Tsao

Huey-En Tzeng

Hwei-Ming Wang

Jaw Yuan Wang

Po-Li Wei

Tsai Sheng Yang

Kun-Huei Yeh

Yu-Min Yeh

Chueh-Chuan Yen

Jui-Hung Tsai

Ukraine

Mokhammad Abo Gali

Hryhoriy Adamchuk

Igor Bondarenko

Lina Bondarenko

Oleksii Datsenko

Olena Datsenko

Kateryna Denysenko

Alisa Dubnytska

Oleksandr Fedoryaka

Viktor Fomenko

Svitlana Glushchenko

Olexandr Goloborodko

Volodymyr Holotiuk

Ievgen Ilin

Ruslan Khmyzov

Anna Kryzhanivska

Dmytro Lutsenko
Yuliia Mikhieienko
Yurii Mohylnyi
Olga Nepomyashcha
Alla Nikitenko
Yurii Partykevych
Alona Perehinets
Serhii Posokhov
Volodymyr Pyrogovskyy
Dinara Ryspayeva
Yaroslav Shestakov
Denys Skoryi
Vasyl Skrypko
Olha Soloshenko
Bogdan Sorokin
Viktoriia Stavniichuk
Iryna Stepanovych
Svitlana Tsupikova
Daria Tsvietaieva-Berest
Nataliia Urzhumova
Konstiantyn Vorona
Oleg Yakovenko

United Kingdom

Mohammed Alhilali
Avani Athauda
Shobhit Baijal
Ian Chau
David Church
Veronica Crespi
Michelle Cunnell
David Cunningham
Rebecca Finn
Caroline Yean Kit Fong
Ashram Gautam
Ehsan Ghorani
Anderley Gordon
Ahmed Hashmi
Solat Hasnain
Lauren Jones
Laura Kennedy
Rachel Kerr
Valentinos Kounnis
Racha Kussaibati
David Lau
Mo Linh Le
Iris Levink
Martin Little
Abdul Mian
Alex Mitchell
Nabeel Naban
Alexandra Ostler
Jeffrey Rubasingham
Timothy Simmons
Maninder Singh
Shivan Sivakumar
James Stewart
Nicola Valeri
Harpreet Wasan
Alex Watson
Zin Win
Victoria Woodcock

United States

Ivy Abraham
Laith Abushahin
Kenneth Adler
Raluca Agafitei
Rajiv Agarwal
Daniel Ahn
Diogenes Alayon
Lauren Albertine
Steven Alberts
Sandra Algaze
Roba Alhasan
Andrea Amico
Harshad Amin
Manik Amin
Victoria Amran
Lalitha Anand
Brittany Andress
Kylee Andrews
Leah Anton
Santiago Aparo
Arlyn Apollo
Jeneth Aquino
Olivia Aranha
Allen Ardestani
Karim Arnaout
Geri Arnell
Niyati Asher
Steven Attia
Louis Avvento
Georges Azzi
Hani Babiker
Nusayba Bagegni
Ellen Ball
Jacintha Ballingall
Victoria Barrett
Jacqueline Barrientos
Afsaneh Barzi
Ramakrishna Battini
Scott Baum
Tanios Bekaii-Saab

Tanios Bekaii-Saab
Karen Belleh
Sonia Benn
Jordan Berlin
Arnold Blaustein
Morgan Bonds
Ashton Boyle
Christopher Bozarth
Mary Brady
Robin Brenner
Vanessa Brink
Julia Brockway-Marchello
Evelyn Brosnan
Anna Brown
Angela Bryant
Diane Buchbarker
Claudia Calderon
Dana Cardin
Teresa Carpenter
Lucia Carranza
Andrea Cercek
Gerard Chaaya
Majd Chahin
Seungjean Chai
Sakti Chakrabarti
Sree Chalasani
Joseph Chao
Salman Chaudhry
Denaly Chen
Grace Chen
Roy Chen
Elena Chiorean
Simona Chivu
Lydia Chow

Nicole Chrisman
David Christianson
David Chu
Diane Chun
RaYoung Chung
Vincent Chung
Robert Chun-Hao Hsu
Kristen Ciombor
Patrick Cobb
Janet Cogswell
Stacey Cohen
Katrina Columna
Samantha Conlin
Louise Connell
Dana Connor
Deborah Conroy
Carol Covarrubias
Andrew Coveler
Elizabeth Cruz
Oxana Crysler
Mike Cusnir
George Cyriac
Noshir DaCosta
Satya Das
Nageshwara Dasari
Emily Davidson
Keith Davidson
Molly Davis
Shemeka Davis
David de la Torre-Dorado
Fernando De Zarraga
Allison Dekker
Dustin Deming
William DeRosa

Amishi Desai
Avni Desai
Christina DiBenedetto
Erin Diguglielmo
Stephen Dong
James Drechsler
David Drew
Kathleen Dronkowski
Pamela Drullinsky
Lingling Du
Emily Dunaway
Gary Dunn
David Eagle
Rachel Eiring
David Eisner
Ahmad El-Far
Rifat Elkhatib
Anthony El-Khoueiry
Tarek Elrafei
Patricia Emfinger
Cathy Eng
Thomas Enzler
Bassam Estfan
Nkiruka Ezenwajiaku
Jamie Faber
Marwan Fakih
Lorena Farrell
Moon Fenton
Eduardo Fernandez-Hernandez
Lynn Feun
Troy Fiddler
Victoria Finland
Gene Finley

Phyllis Fiveash
Elizabeth Fleming
Casey Fletcher
Michael Foote
Christopher Foss
Missy Foster
Benjamin Freeman
David Friedland
Laura Friedman
Monica Fukala
Sharon Funkhouser
David Gallinson
Karuna Ganesh
Stephanie Gebhardt
Ben George
Jerry George
Roshini George
Sagila George
Michael Gibson
Upama Giri
Oleg Gligich
Laura Goff
Ronald Goldberg
Zoe Goldberg
Jun Gong
Nohelia Gonzalez
Ragisha Gopalakrishnan
Vikram Gorantla
Constance Gore
Margaret Ann Gosnell
Jeffrey Green
Heather Greene
Jenna Gregory
Patrick Grierson
Jennifer Griffith
Roxanne Griswold
Axel Grothey
Steven Gruenstein
Julie Gruszczynski
Ping Gu
Adriana Guigova
Sarada Gurubhagavatula
Karen Haiber
Gregory Haidemenos
Thorvardur Halfdanarson
Angela Hamilton
Diana Hanna
Lindsay Hannan
James Harding
Amy Harkness
Leonard Harris
William Harris
Mindy Hartgers
Amy Hartman
Hassan Hatoum
Deborah Hawkins
John Hays
Aiwu He
Jaclyn Hechtman
Nicole Heinz
Andrew Hendifar
Lindsay Herron
Thatcher Heumann
Haley Hill
Evthokia Hobbs
Timothy Hobday
David Hoffman
Emily Hoffmann
Lauren Holbrook
Ellen Hollywood
Peter Hosein
Mindy Hsiao
Ronan Hsieh
Dennis Hsu
Johann Hsu
Joleen Hubbard
Ryan Huey
Nooreen Hussain
Jimmy Hwang
Elena Ignatiev
David Ilson
Samantha Inabinett
Syma Iqbal
Alyssa Iwano
Siddharth Iyengar
Amy Izzett
Regina Jablonski
Jerry Jaboin
Ajay Jain
Sanjeev Jain
Amjad Jalil
Jessica James
Aminah Jatoi
Priya Jayachandran
Ning Jin
Zhaohui Jin
Mazen Jizzini
Jacob Jochum
Tanner Johanns
Benny Johnson
Robert Johnson
Valerie Johnson

Jeremy Jones
Makenzie Jones
Smita Joshi
Karen Julien
Kunal Kadakia
Suneel Kamath
Mandana Kamgar
Lionel Kankeu Fonkoua
Erica Kaufmann
Judith Kaur
Omar Kayaleh
Bryan Kee
Bethany Kelley
David Kelsen
Nancy Kemeny
Hagen Kennecke
Maureen Kennedy
Charlean Ketchens
Alok Khorana
Taline khoukaz
Moh'd Khushman
Shandell Kidd
Eileen Kilgus-Reid
Young-Wook Kim
Hedy Kindler
Gentry King
Rachel Kitson
William Klein
Eric Knoche
Swapna Kochuveettil
Irina Konzelmann
Scott Kopetz
Mark Kozloff
Marian Krasowski
Jeremy Kratz
John Krauss
Anuja Kriplani
Smitha Krishnamurthi
Anuradha Krishnamurthy
Kimberly Kruczek
Bahar Laderian
Oscar Lahoud
Kimberly Laino
Raylene Langish
Hugo Lara-Martinez
Mary Larsen
Jade Law
Michael Lee
Nara Lee
Heinz-Josef Lenz
Beth Levine
Grant Lewis
Daneng Li
Jia Li
Rui Li
Chih-Yi Liao
Joseph Light
Louise Ligresti
Dean Lim
Kian-Huat Lim
William Lipera
Noelle LoConte
Gregory Long
Jeremy Lorber
Yanyan Lou
Nicole Loving
Yi-Tsung Lu
Sam Lubner
Janet Luong
Barry Luskey
Aleya Lyn
Michelle Mackenzie
Amit Mahipal
Porshia Mahoro
Ofer Maimran
Umair Majeed
Pannaga Malalur
Monica Malhotra
Ashish Manne
Rami Manochakian
Sherie Mar-Chaim
Jessica Marchisotto
Stanley Marks
Margaret Marriott
John Marshall
Michael Martin
Anastasia Martynova
Gena Mathew
Khalid Matin
Marc Matrana
Sarah McCaffrey
Fransen McGinley
Deaglan McHugh
Evan McKiernan
Carrie McKiernon
Julian McLain
Michael McNamara
Robert McWilliams
Jose Melo
Vincent Mendiola
Brittany Mezquita
Sarah Miller

Lisa Mills
Vinay Minocha
Jessica Mitchell
Jocelyn Mitchell
Arjun Mittra
Jonathan Mizrahi
Kabir Mody
Casey Moffa
Tabraiz Mohammed
Kerri Mola-Rudd
Parisa Momtaz
Dulabh Monga
Steven Montana
Maria Morelli
Alvaro Moreno-Aspitia
Katherine Morris
Van Morris
Ashley Morton
Christina Mraz
Lynne Muir
Reetu Mukherji
Daniel Mulkerin
Julie Murumba
Laura Musselwhite
Jennifer Myers
Erin Myklebust
Neil Nagovski
Kanika Nair
Azadeh Namakydoust
Sunil Narula
Katherine Navo
Shahid Nawaz
Reza Nazemzadeh
Anu Neerukonda

Kenneth Ng
Vincent Nguyen
Jodi Nickel
Ryan Nipp
Marcus Noel
Anne Noonan
Jaclyn Norris
Marcus Norris
Yelda Nouri
Elise Novo
Amory Novoselac
Kelli Nugent
Maliha Nusrat
Nneka Nwachukwu
Suhail Obaji
Chika Obele
Meghan O'Brien
Kelly O'Connor
Stephanie Oetting
Peter Oppelt
Mary Orians
Arsen Osipov
Elizabeth Ott
Michael Overman
Colette Owens
Jonathan Pai
Arnel Pallera
Manjari Pandey
Gail Panton
Brenda Panzera
Steven Papish
Haeseong Park
MiHee Park
Viktoriya Paroder

Christine Parseghian
Bhaumik Patel
Monica Patel
Ronak Patel
Samir Patel
Asit Paul
Katrina Pedersen
Daniel Petro
Roshni Philip
Liza Piazzaro
Erin Pierce
Agustin Pimentel
Natasha Pinheiro
Victoria Pinkava
Catherine Pires
Henry Pitot
Andrew Poklepovic
Blase Polite
Gairta Porroga
Jason Porter
Bruce Porterfield
David Portnoy
Daniel Powell
Isabel Preeshagul
Sippy Punn
Christine Racette
Lisa Rader
Kanwal Raghav
Karen Ragusa
Shafia Rahman
Moses Raj
Nitya Raj
Richard Ralls
Murtuza Rampurwala

Devika Rao
Thomas Ratliff
Shayan Rayani
Timothy Rearden
Jennifer Reeder
Rabia Rehman
Diane Reidy-Lagunes
Stacy Reschke
Avi Retter
John Rhee
Sylvia Richey
Caron Rigden
Eva Ritzmann
Janene Roberson-Marshall
Lester Robertson
Mary Rockwell
Darwin Roman
Kelley Rone
Yolanda Roper
Varun Roy
Sameek Roychowdhury
Tariq Sabir
Meena Sadaps
Rachael Safyan
Lisa Salem
Mohamed Salem
John Salmon
Leonard Saltz
Joel Saltzman
Ashley Sandridge
Suma Satti
Cynthia Savaiano
Kevin Scher
Juan Schvartzman
Michael Schwartz
Lee Schwartzberg
Lisa Scofield
Joshua Scott
Michal Segal
Neil Segal
Zdenka Segota
Leonard Seigel
Neil Shah
Shannon Shah
Sridhar Shankar
Veena Shankaran
Marc Shapiro
Deepali Sharma
Marina Shcherba
John Shen
Ardaman Shergill
Jane Sherman
Aditya Shreenivas
Imad Shureiqi
Aron Simkins
Frank Sinicrope
Jenna Sinopoli
Karleung Siu
Emily Skotte
Kathryn Slane
Anna Smith
Bradley Somer
Bradley Somer
Mohamad Sonbol
Tina Soo
Jennifer Spann
Alison Spellman
Deepika Sriram
Zsofia Stadler
Jason Starr
Sunny Stinson
Charlee Striebinger
Ryan Sugarman
Rama Suresh
Courtney Susser
Leslie Swanson
Gurmohan Syali
Jason Tache
Benjamin Tan
Winston Tan
Kurt Tauer
Mark Taylor
Yeshanew Teklie
Brian Thomas
Gary Thomas
Jacob Thomas
James Thomas
Roby Thomas
Sajeve Thomas
Gary Tian
Justin Tiulim
Angelica Tolentino
Alfredo Torres
Janet Torres
Joanna Tortora
Tashani Townsend
Lan Tran
Nguyen Tran
Nikolaos Trikalinos
Tiffany Troso-Sandoval
Ruby Turner

Nataliya Uboha
Antonio Ucar
Mikka Uehara
Susanna Ulahannan
Rebecca Umayam
Elizabeth Upchurch
Gina Vaccaro
Hetal Vachhani
Daniel Vaena
Pamela Vaiskauskas
Efsevia Vakiani
Beth Vanden Hoek
Ari Vanderwalde
Anna Varghese
Christine Veenstra
Carmel Verrier
Gregory Vidal
Joy Vlamakis
Fabio Volterra
Kandice Walker
Deborah Wallace
James Wallace
Lan Wang
Rui Wang
Leslie Washburn
Gloria Wasilewski
Jaime Watkins
Michael Wax
Wen Wee Ma
Mary Weeg
Benjamin Weinberg
Louis Weiner
Saradasri Wellikoff
Neil West
Katherine Wherity
Stephen White
Julie Whitworth
Christen Wiedower
Eric Wiedower
Jenna Wilke
Anneleis Willett
Toya Williams
Jason Willis
Meredith Winkelhake
Michelle Wisniewski
Crystal Wolf
Robert Wolff
Elizabeth Won
Alexandra Wong
Kit Wong
Neilee Wood
Kathryn Wright
Christina Wu
Natasha Wylie
Hao Xie
Rona Yaeger
Jessica Yang
Harry Yoon
Irene Yu
Shi Yu
Mark Zalupski
Alice Zervoudakis
David Zhen
Mojun Zhu
Alexander Zuhoski
Richard Zuniga

## Listing of institutional review board or independent ethics committee

| Study Site Number | Independent Ethics Committee or Institutional Review Board Address(es) |
|---|---|
| 1057 | Comité de Ética en Investigación Clínica (CEIC)<br>Paraná 755, Recoleta, Comuna 2, 6 "A" y "B"<br>Ciudad Autónoma de Buenos Aires, CIUDAD AUTÓNOMA DE BUENOS AIRES C1017 AAO<br>ARGENTINA |
| 1149 | Comite Institucional de Etica de la Investigacion en Salud de la Clinica Universitaria Reina Fabiola (CIEIS)<br>Jacinto Ríos 554,Piso 8, oficina 3<br>Cordoba, CORDOBA 5004<br>ARGENTINA<br><br>Consejo de Evaluación Ética de Investigación en Salud - CoEIS<br>Avenida Velez Sarsfield 2311<br>Córdoba, CÓRDOBA 5000<br>ARGENTINA |
| 1185 | Comité Institucional de Ética de Investigación en Salud<br>Av. Naciones Unidas 346, Barrio Parque Velez Sarsfield, Hospital Privado - Centro Médico de<br>Córdoba S.A.<br>Córdoba, CORDOBA X5016KEH<br><br>ARGENTINA<br>Consejo de Evaluación Ética de Investigación en Salud - CoEIS<br>Avenida Velez Sarsfield 2311<br>Córdoba, CÓRDOBA 5000<br>ARGENTINA |
| 1319 | Comité de Ética de Investigaciones del Instituto Alexander Fleming<br>Crámer 1180,2do Piso<br>Ciudad Autónoma de Buenos Aires, CIUDAD AUTÓNOMA DE BUENOS AIRES 1426<br>ARGENTINA |
| 1104 | Peter MacCallum Cancer Centre Ethics Committee<br>305 Grattan Street<br>Melbourne, VICTORIA 3000<br>AUSTRALIA |
| 1138 | Peter MacCallum Cancer Centre Ethics Committee<br>305 Grattan Street<br>Melbourne, VICTORIA 3000<br>AUSTRALIA |
| 1178 | Peter MacCallum Cancer Centre Ethics Committee<br>305 Grattan Street<br>Melbourne, VICTORIA 3000<br>AUSTRALIA<br><br>Peter MacCallum Cancer Centre Ethics Committee<br>Level 4, 305 Grattan Street<br>Melbourne, VICTORIA 3000<br>AUSTRALIA |
| 1232 | Peter MacCallum Cancer Centre Ethics Committee<br>305 Grattan Street<br>Melbourne, VICTORIA 3000<br>AUSTRALIA |
| 1233 | Peter MacCallum Cancer Centre Human Research Ethics Committee<br>305 Grattan St<br>Melbourne, VICTORIA 3000<br>AUSTRALIA |
| 1234 | Peter MacCallum Cancer Centre Ethics Committee<br>Level 4, 305 Grattan Street<br>Melbourne, VICTORIA 3000<br>AUSTRALIA |
| 1240 | Peter MacCallum Cancer Centre Ethics Committee (Inactive IRB/IEC)<br>Level 4, 305 Grattan Street<br>Melbourne, VICTORIA 3000 |

| | |
|---|---|
| | AUSTRALIA<br><br>Peter MacCallum Cancer Centre Ethics Committee<br>305 Grattan Street<br>Mebourne, VICTORIA 3000<br>AUSTRALIA |
| 1269 | Peter MacCallum Cancer Centre Ethics Committee<br>305 Grattan Street<br>Mebourne, VICTORIA 3000<br>AUSTRALIA |
| 1271 | Peter MacCallum Cancer Centre Ethics Committee<br>305 Grattan Street<br>Melbourne, VICTORIA 3000<br>AUSTRALIA |
| 1358 | Peter MacCallum Cancer Centre Ethics Committee<br>305 Grattan Street<br>Melbourne, VICTORIA 3000<br>AUSTRALIA |
| 1121 | CHU UCL Namur site Godinne<br>Av Docteur G Therasse 1<br>Yvoir, 5530<br>BELGIUM |
| 1179 | CHU UCL Namur site Godinne<br>Av Docteur G Therasse 1<br>Yvoir, 5530<br>BELGIUM |
| 1203 | CHU UCL Namur site Godinne<br>Av Docteur G Therasse 1<br>Yvoir, 5530<br>BELGIUM |
| 1221 | CHU UCL Namur site Godinne<br>Av Docteur G Therasse 1<br>Yvoir, 5530<br>BELGIUM |
| 1265 | CHU UCL Namur site Godinne<br>Avenue du Docteur Gaston Therasse 1<br>Yvoir, 5530<br>BELGIUM |
| 1289 | CHU UCL Namur - Ethic committee<br>Av. Gaston Therasse 1<br>Yvoir, NAMUR 5530<br>BELGIUM |
| 1317 | CHU UCL Namur - Ethic committee<br>Av. Gaston Therasse 1<br>Yvoir, NAMUR 5530<br>BELGIUM |
| 1338 | CHU UCL Namur - Ethic committee<br>Av. Gaston Therasse 1<br>Yvoir, NAMUR 5530<br>BELGIUM |
| 1176 | Comitê de Ética e Pesquisa em Seres Humanos da Faculdade de Medicina de São José do Rio Preto<br>Centro Integrado de Pesquisa<br>5416 Avenida Brigadeiro Faria Lima,Vila Sao Manoel, São José do Rio Preto<br>São José do Rio Preto, SÃO PAULO 15090-000<br>BRAZIL |
| 1231 | Comissão Nacional de Ética em Pesquisa - CONEP<br>SRTV 701, Via W 5 Norte, lote D, Edificio PO 700, 3° andar - Asa Norte<br>Brasilia, DF 70719-040<br>BRAZIL |
| 1235 | Comissão Nacional de Ética em Pesquisa - CONEP<br>SRTVN 701, Via W 5 Norte, lote D, Edifício PO 700, 3° andar- Asa Norte<br>Brasília, DF 70719-040<br>BRAZIL<br><br>Comitê de Ética em Pesquisa da Universidade do Vale do Taquari - Univates |

| | |
|---|---|
| | Rua Avelino Tallini, 171 - Sala 309 - Prédio 01, Bairro Universitário<br>Lajeado, RS 95914-014<br>BRAZIL |
| 1337 | Comissão Nacional de Ética em Pesquisa (CONEP)<br>SRTVN 701, Via W 5 Norte, Lote D, Edificio PO 700, 3° andar, Asa Norte<br>Brasilia, DF 70719-040<br>BRAZIL<br><br>Hospital das Clínicas da Faculdade de Medicina da Universidade de São Paulo-HCFMUSP<br>Rua Dr. Ovídio Pires de Campos, 225 ‐ 5$^{o}$ andar ‐ Prédio da Administração<br>São Paulo, SÃO PAULO 05403-010<br>BRAZIL |
| 1345 | Comissão Nacional de Ética em Pesquisa (CONEP)<br>SRTVN 701, Via W 5 Norte, Lote D, Edificio PO 700, 3° andar, Asa Norte<br>Brasilia, DF 70719-040<br>BRAZIL<br><br>Comitê de Ética em Pesquisa do Centro Universitário Faculdade de Medicina do ABC - FMABC<br>Avenida Lauro Gomes, 2000 ‐ Prédio CEPES, 1$^{o}$ andar, salas 63 e 64 ‐ Vila Sacadura Cabral<br>Santo André, SÃO PAULO 09060-870<br>BRAZIL |
| 1347 | Comitê de Ética em Pesquisa do Hospital de Clínicas de Porto Alegre<br>Av. Protásio Alves, 211 - Portão 4 - Rio Branco - Porto Alegre/RS,, Santa Cecilia, Porto Alegre, 5$^{o}$<br>andar do Bloco C<br>Porto Alegre, RIO GRANDE DO SUL 90410-000<br>BRAZIL<br><br>Comitê de Ética em Pesquisa do Hospital de Clínicas de Porto Alegre (Inactive IRB/IEC)<br>Unidade de Pesquisa Clínica em Oncologia do Hospi<br>2350 Rua Ramiro Barcelos, Santa Cecilia, Porto Alegre, 2nd floor<br>Porto Alegre, RIO GRANDE DO SUL 90035-903<br>BRAZIL<br><br>Conep - Comissao Nacional De Etica E Pesquisa<br>701, Asa Norte, SGAN, Brasília, Edificio PO 700, 3 andar<br>Brasilia, DISTRITO FEDERAL 70755-540<br>BRAZIL |
| 1362 | Comissão Nacional de Ética em Pesquisa - CONEP<br>SRTVN 701 Via W 5 Norte, Lote D, Edificio PO700 3o Andar Asa Norte<br>Brasilia, DF 70719-040<br>BRAZIL<br><br>Hospital do Cancer de Barretos - Fundacao Pio XII<br>Rua Antenor Duarte Vilela, 1331 - Bairro Dr. Paulo Prata, Instituto de Ensino e Pesquisa - sala<br>CEP s/n<br>Barretos, SP 14784-400<br>BRAZIL |
| 1365 | Comissão Nacional de Ética em Pesquisa - CONEP<br>SRTVN 701, Via W 5 Norte, lote D, Edifício PO 700, 3$^{o}$ andar- Asa Norte<br>Brasília, DF 70719-040<br>BRAZIL<br><br>Instituto Nacional de Cancer José Alencar Gomes da Silva - INCA<br>Rua do Resende 128, 2 andar, Sala 204<br>Rio de Janeiro, RIO DE JANEIRO 20231-092<br>BRAZIL |
| 1379 | Comissão Nacional de Ética em Pesquisa - CONEP<br>SRTVN 701 Via W 5 Norte, Lote D, Edificio PO700 3o Andar Asa Norte<br>Brasilia, DF 70719-040<br>BRAZIL |

| | |
|---|---|
| | Comitê de ética em Pesquisa da Universidade Regional de Blumenau - FURB<br>Rua Antônio da Veiga, 140, Itoupava Seca, Campus 1, Bloco I, Sala 506, Bairro Victor Konder<br>Blumenau, SANTA CATARINA 89.030-903<br>BRAZIL |
| 1165 | Ethics Committee for Clinical Trials<br>8, Damyan Gruev Str.<br>Sofia, SOFIA 1303<br>BULGARIA |
| 1274 | Ethics Committee for Clinical Trials<br>8, Damyan Gruev Str.<br>Sofia, SOFIA 1303<br>BULGARIA |
| 1275 | Ethics Committee for Clinical Trials<br>8, Damyan Gruev Str.<br>Sofia, SOFIA 1303<br>BULGARIA |
| 1276 | Ethics Committee for Clinical Trials<br>8, Damyan Gruev Str.<br>Sofia, SOFIA 1303<br>BULGARIA |
| 1277 | Ethics Committee for Clinical Trials<br>8, Damyan Gruev Str.<br>Sofia, SOFIA 1303<br>BULGARIA |
| 1278 | Ethics Committee for Clinical Trials<br>8, Damyan Gruev Str.<br>Sofia, SOFIA 1303<br>BULGARIA |
| 1279 | Ethics Committee for Clinical Trials<br>8, Damyan Gruev Str.<br>Sofia, SOFIA 1303<br>BULGARIA |
| 1132 | CIUSSS West-Central-Montreal-Research-Ethics-Board<br>3755 Cote Ste Catherine RD,E-670<br>Montreal, QUEBEC H3T 1E2<br>CANADA<br><br>Jewish General Hospital (Inactive IRB/IEC)<br>3755 Cote Ste Catherine RD,E-670<br>Montreal, QUEBEC H3T 1E2<br>CANADA |
| 1322 | Health Research Ethics Board of Alberta (HREBA) - Cancer Committee<br>1500 - 10104 103 Avenue NW<br>Edmonton, ALBERTA T5J 0H8<br>CANADA |
| 1323 | Health Research Ethics Board of Alberta Cancer Committee<br>1500, 10104 - 103 Avenue NW<br>Edmonton, ALBERTA T5J 0H8<br>CANADA |
| 1326 | CIUSSS West- Central Montreal Research Ethics Board<br>3755 Chemin de la Côte-Sainte-Catherine,Côte-Des-Neiges–Notre-Dame-De-Grâce, Communauté-Urbaine-de-Montréal<br>Montréal, QUEBEC H3T 1E2<br>CANADA |
| 1332 | OCREB<br>MaRS Centre - Suite 510, 661 University Avenue<br>Toronto, ON M5G 0A3<br>CANADA |
| 1346 | Ontario Cancer Research Ethics Board<br>661 University Avenue,Suite 510<br>Toronto, ONTARIO M5G 0A3<br>CANADA |
| 1349 | OCREB<br>MaRS Centre - Suite 510, 661 University Avenue<br>Toronto, ON M5G 0A3 |

| | |
|---|---|
| | CANADA |
| 1098 | Human Ethics Committee of the Second Affiliated Hospital of College of Medicine, Zhejiang<br>University<br>No.88 Jiefang Road<br>Hangzhou, 310009<br>CHINA |
| 1130 | Ethics Committee of The Third Xiangya Hospital of Central South University<br>No.138 Tongzipo Road, Yuelu District<br>Changsha, HUNAN 410013<br>CHINA |
| 1136 | Ethics Committee of Ruijin Hospital Affiliated to Shanghai Jiaotong University School of Medicine<br>No.197 Ruijin Er Road<br>Shanghai, SHANGHAI 200025<br>CHINA |
| 1139 | Medical Ethics Committee of Beijing Cancer Hospital<br>No.81 Fu-cheng Road, Haidian District<br>Beijing, BEIJING 100142<br>CHINA |
| 1154 | Medical Ethics Committee of Fudan University Shanghai Cancer Center<br>No. 270, Dong'an Road, Xuhui District<br>Shanghai, SHANGHAI 200030<br>CHINA |
| 1169 | Ethics Committee of Fujian Medical University Union Hospital<br>No. 29, Xinquan Road<br>Fuzhou, FUJIAN 350001<br>CHINA |
| 1195 | Ethics Committee of Tianjin Union Medical Center<br>No. 190, Jieyuan Road, Hongqiao District<br>Tianjin, 300000<br>CHINA |
| 1198 | Clinical Trial Ethics Committee of Huazhong University of Science and Technology<br>No.13 Hangkong Road<br>Wuhan, HUBEI 430030<br>CHINA |
| 1236 | Ethics Committee of The Sixth Affiliated Hospital, Sun Yat-sen University<br>No.26 Yuan Cun Er Heng Lu<br>Guangzhou, GUANGDONG 510655<br>CHINA |
| 1242 | Ethics Committee of Human Trial of Shanghai General Hospital<br>No.100 Haining Road<br>Shanghai, SHANGHAI 200080<br>CHINA |
| 1258 | Ethics Committee of Beijing Hospital<br>No. 1 Dongdan Dahua road Dongcheng district<br>Beijing, BEIJING 100730<br>CHINA |
| 1259 | Ethics Committee of Yunnan Cancer Hospital<br>No.519 Kunzhou Road, Xishan District<br>Kunming, YUNNAN 650118<br>CHINA |
| 1260 | Ethics Committee of the Shandong First Medical University affiliated oncology hospital<br>No. 440 Jiyan Road, Huaiyin Distric<br>Jinan, SHANDONG 250117<br>CHINA |
| 1290 | Ethics Committee of Cancer Hospital Chinese Academy of Medical Sciences<br>No. 17 Panjiayuan Nan Li, Chaoyang District,<br>Beijing, 100021<br>CHINA |
| 1387 | Ethics Committee of Jinan Central Hospital<br>No. 105, Jiefang Road<br>Jinan, SHANDONG 250013<br>CHINA |
| 1388 | Ethics Committee of Sichuan Province Cancer Hospital<br>55 Siduan People's South Road |

| | |
|---|---|
| | Chengdu, SICHUAN 610041<br>CHINA |
| 1392 | Ethics Committee of Affiliated Tumor Hospital of Guangxi Medical University<br>No.71 Hedi Road<br>Nanning, GUANGXI 530021<br>CHINA |
| 1416 | Peking University First Hospital Biological and Medical Research Ethics Committee<br>Room 308, Administrative South Building,No.8 Xishiku Street, Xicheng District<br>Beijing, 100034<br>CHINA |
| 1417 | Ethics Committee of Clinical Medical Research of First Affiliated Hospital of Anhui Medical Univ<br>218 Jixi Road, Shushan District<br>Hefei, ANHUI 230022<br>CHINA |
| 1420 | Drugs (Devices) Clinical trials of Ethics Committee of Henan Provincial People's Hospital<br>No.7 Weiwu Road, Jinshui District<br>Zhengzhou, HENAN 450003<br>CHINA |
| 1423 | Medical Ethics Committee of The Second Xiangya Hospital of Central South University<br>No.139 Renmin Middle Road<br>Changsha, HUNAN 410011<br>CHINA |
| 1428 | Ethics Committee of Shengjing Hospital of China Medical University<br>No. 36 Sanhao Street, Heping District<br>Shenyang, LIAONING 110004<br>CHINA |
| 1434 | EC of Nanjing Drum Tower Hospital The Affiliated Hospital of Nanjing University Medical School<br>No.321 Zhongshan Road<br>Nanjing, JIANGSU 210008<br>CHINA |
| 1440 | Ethics Committee of Chongqing University Cancer Hospital<br>181 Hanyu Road, Shapingba District<br>Chongqing, CHONGQING 400030<br>CHINA |
| 1458 | Ethics Committee of Guangdong Provincial People's Hospital<br>23F,Haiyin Center, No. 98 Donghua South Road, Yuexiu District<br>Guangzhou, GUANGDONG 510080<br>CHINA |
| 1018 | Eticka komise Fakultni nemocnice Hradec Kralove<br>Sokolska 581<br>Hradec Kralove, 500 05<br>CZECH REPUBLIC<br><br>Eticka komise Fakultni nemocnice Olomouc a LF UP v Olomouci<br>I.P. Pavlova 185/6<br>Olomouc, 779 00<br>CZECH REPUBLIC |
| 1028 | Eticka komise Fakultni nemocnice Olomouc a LF UP v Olomouci<br>I.P. Pavlova 185/6<br>Olomouc, 779 00<br>CZECH REPUBLIC<br><br>Eticka komise IKEM a FTN<br>Fakultni Thomayerova nemocnice<br>Videnska 800<br>Praha 4 - Krc, 140 59<br>CZECH REPUBLIC |
| 1032 | Eticka komise Fakultni nemocnice Brno<br>Jihlavska 20<br>Brno, 625 00<br>CZECH REPUBLIC<br><br>Eticka komise Fakultni nemocnice Olomouc a LF UP v Olomouci<br>I.P. Pavlova 185/6 |

| | |
|---|---|
| | Olomouc, 779 00<br>CZECH REPUBLIC |
| 1123 | Eticka komise Fakultni nemocnice Olomouc a LF UP v Olomouci<br>I.P. Pavlova 185/6<br>Olomouc, 779 00<br>CZECH REPUBLIC<br><br>Eticka komise Fakultni nemocnice v Motole<br>V Uvalu 84<br>Praha 5, 150 06<br>CZECH REPUBLIC |
| 1127 | Eticka komise Fakultni nemocnice Olomouc a LF UP v Olomouci<br>Zdravotniku 248/7,Onkologicka klinika<br>Olomouc, OLOMOUC 779 00<br>CZECH REPUBLIC |
| 1186 | Eticka komise Fakultni nemocnice Bulovka<br>Budinova 2<br>Praha 8, 180 81<br>CZECH REPUBLIC<br><br>Eticka komise Fakultni nemocnice Bulovka<br>Budinova 67/2<br>Praha 8 - Liben, 180 81<br>CZECH REPUBLIC<br><br>Eticka komise Fakultni nemocnice Olomouc a LF UP v Olomouci<br>I.P. Pavlova 185/6<br>Olomouc, 779 00<br>CZECH REPUBLIC |
| 1067 | De Videnskabsetiske Komiteer for Region Syddanmark<br>Damhaven 12,Regionshuset<br>Vejle, N/A 7100<br>DENMARK |
| 1094 | De Videnskabsetiske Komiteer for Region Syddanmark<br>Damhaven 12,Regionshuset<br>Vejle, N/A 7100<br>DENMARK<br><br>De Videnskabsetiske Komitéer for Region Syddanmark<br>Regionshuset, Damhaven 12<br>Vejle, 7100<br>DENMARK |
| 1307 | De Videnskabsetiske Komitéer for Region Syddanmark<br>Regionshuset, Damhaven 12<br>Vejle, 7100<br>DENMARK |
| 1341 | De Videnskabsetiske Komitéer for Region Syddanmark<br>Regionshuset, Damhaven 12<br>Vejle, 7100<br>DENMARK |
| 1344 | De Videnskabsetiske Komitéer for Region Syddanmark<br>Regionshuset, Damhaven 12<br>Vejle, 7100<br>DENMARK |
| 1099 | Valtakunnallinen lääketieteellinen tutkimuseettinen toimikunta TUKIJA<br>Valvira, Ratapihantie 9<br>Helsinki, 00520<br>FINLAND |
| 1207 | Valtakunnallinen lääketieteellinen tutkimuseettinen toimikunta TUKIJA<br>Valvira, Ratapihantie 9<br>Helsinki, 00520<br>FINLAND |
| 1246 | Valtakunnallinen lääketieteellinen tutkimuseettinen toimikunta TUKIJA<br>Valvira, Ratapihantie 9<br>Helsinki, 00520<br>FINLAND |

| | |
|---|---|
| 1298 | Valtakunnallinen lääketieteellinen tutkimuseettinen toimikunta TUKIJA<br>Valvira, Ratapihantie 9<br>Helsinki, 00520<br>FINLAND |
| 1299 | Valtakunnallinen lääketieteellinen tutkimuseettinen toimikunta TUKIJA<br>Valvira, Ratapihantie 9<br>Helsinki, 00520<br>FINLAND |
| 1366 | Valtakunnallinen lääketieteellinen tutkimuseettinen toimikunta TUKIJA<br>Valvira, Ratapihantie 9<br>Helsinki, 00520<br>FINLAND |
| 1031 | Carl von Ossietzky Universität Oldenburg, Medizinische Ethikkommission<br>Ammerländer Heerstrasse 114-118<br>Oldenburg, NIEDERSACHSEN 26129<br>GERMANY |
| 1061 | Carl von Ossietzky Universität Oldenburg<br>Medizinische Ethikkommission<br>Ammerländer Heerstrasse 114-118<br>Oldenburg, 26129<br>GERMANY |
| 1075 | Carl von Ossietzky Universität Oldenburg<br>Medizinische Ethikkommission<br>Ammerländer Heerstrasse 114-118<br>Oldenburg, 26129<br>GERMANY |
| 1126 | Carl von Ossietzky Universität Oldenburg<br>Medizinische Ethikkommission<br>Ammerländer Heerstrasse 114-118<br>Oldenburg, 26129<br>GERMANY<br><br>Ethik-Kommission der Ärztekammer Hamburg<br>Weidestraße 122 b<br>Hamburg, HAMBURG 22083<br>GERMANY |
| 1131 | Carl von Ossietzky Universität Oldenburg<br>Medizinische Ethikkommission<br>Ammerländer Heerstrasse 114-118<br>Oldenburg, 26129<br>GERMANY |
| 1159 | Carl von Ossietzky Universität Oldenburg, Medizinische Ethikkommission<br>Ammerländer Heerstrasse 114-118<br>Oldenburg, NIEDERSACHSEN 26129<br>GERMANY |
| 1177 | Carl von Ossietzky Universität Oldenburg<br>Medizinische Ethikkommission<br>Ammerländer Heerstrasse 114-118<br>Oldenburg, 26129<br>GERMANY |
| 1272 | Carl von Ossietzky Universität Oldenburg<br>Medizinische Ethikkommission<br>Ammerländer Heerstrasse 114-118<br>Oldenburg, 26129<br>GERMANY |
| 1364 | Carl von Ossietzky Universität Oldenburg, Medizinische Ethikkommission<br>Ammerländer Heerstrasse 114-118<br>Oldenburg, NIEDERSACHSEN 26129<br>GERMANY |
| 1103 | Institutional Ethics Committee<br>Dr. Ernest Borges road,3rd Floor, Main Building, Tata Memorial Hospital<br>Mumbai, MAHARASHTRA 400012<br>INDIA |
| 1160 | Institutional Review Board Rajiv Gandhi Cancer Institute and Research Centre<br>Sector 5, Rohini<br>New Delhi, DELHI 110085 |

| | |
|---|---|
| | INDIA |
| 1229 | The Ethics Committee S.M.S. Medical College and Attached Hospitals<br>JLN Marg<br>Jaipur, RAJASTHAN 302004<br>INDIA |
| 1300 | Sahyadri Hospitals Limited Ethics Committee<br>Sahyadri Clinical Research & Development Center (A Unit of Sahyadri Hospitals Ltd.)<br>Karve Road,33/34 B, Makarand Bhave Path<br>Pune, MAHARASHTRA 411004<br>INDIA |
| 1306 | Institutional Ethics Committee (IEC)<br>Deenanath Mangeshkar Hospital & Research Centre<br>Erandwane<br>Pune, MAHARASHTRA 411 004<br>INDIA |
| 1331 | Bhaktivedanta Hospital Ethics Committee<br>Bhaktivedanta Swami Marg, Mira Road, Thane, 3rd floor HEC Office, Bhaktivedanta Hospital &<br>Research Institute, Srishti complex<br>Mira Bhayandar, MAHARASHTRA 401107<br>INDIA |
| 1026 | Comitato Etico Area Nord Veneto<br>Via Gattamelata, 64<br>Padua, VENETO 35128<br>ITALY |
| 1029 | Comitato Etico Area Nord Veneto<br>Via Gattamelata, 64<br>Padua, VENETO 35128<br>ITALY |
| 1051 | Comitato Etico Area Nord Veneto<br>Via Gattamelata, 64<br>Padua, VENETO 35128<br>ITALY |
| 1054 | Comitato Etico Area Nord Veneto<br>Via Gattamelata, 64<br>Padua, VENETO 35128<br>ITALY |
| 1143 | Comitato Etico Area Nord Veneto<br>Via Gattamelata, 64<br>Padua, VENETO 35128<br>ITALY |
| 1163 | Comitato Etico Area Nord Veneto<br>Via Gattamelata, 64<br>Padua, VENETO 35128<br>ITALY |
| 1226 | Comitato Etico Area Nord Veneto<br>Via Gattamelata, 64<br>Padua, VENETO 35128<br>ITALY |
| 1253 | Comitato Etico Area Nord Veneto<br>Via Gattamelata, 64<br>Padua, VENETO 35128<br>ITALY |
| 1264 | Comitato Etico Area Nord Veneto<br>Via Gattamelata, 64<br>Padua, VENETO 35128<br>ITALY |
| 1348 | Comitato Etico Area Nord Veneto<br>Via Gattamelata, 64<br>Padua, VENETO 35128<br>ITALY |
| 1382 | National Cancer Center IRB<br>5-1-1, Tsukiji<br>Chuo-ku, TOKYO 104-0045<br>JAPAN |
| 1383 | Aichi Cancer Center Hospital IRB |

| | |
|---|---|
| | Kanokoden, Chikusa-ku, 1-1<br>Nagoya, AICHI 464-8681<br>JAPAN |
| 1384 | Shizuoka Cancer Center IRB<br>1007 Shimonagakubo, Nagaizumi-cho<br>Sunto-gun, SHIZUOKA 411-8777<br>JAPAN |
| 1394 | Kanazawa University Hospital IRB<br>13-1, Takaramachi<br>Kanazawa, ISHIKAWA 920-8641<br>JAPAN |
| 1395 | National Cancer Center Hospital IRB<br>5-1-1 Tsukiji, Chuo-ku<br>Tokyo, 104-0045<br>JAPAN |
| 1396 | Kanagawa Cancer Center IRB<br>2-3-2 Nakao, Asahi-ku<br>Yokohama, KANAGAWA 241-8515<br>JAPAN |
| 1397 | Osaka University Hospital Institutional Review Board<br>2-15 Yamadaoka<br>Suita, OSAKA 565-0871<br>JAPAN |
| 1398 | National Hospital Organization Shikoku Cancer Center Institutional Review Board<br>160 Kou Minamiumenomoto-machi<br>Matsuyama, EHIME 7910280<br>JAPAN |
| 1399 | Japanese Foundation for Cancer Research Institutional Review Board<br>3-8-31 Ariake<br>Koto-ku, TOKYO 135-8550<br>JAPAN |
| 1400 | Osaka Prefectural Hospital Organization Osaka International Cancer Institute IRB<br>3-1-69 Otemae, Chuo-ku<br>Osaka-shi, OSAKA 541-8567<br>JAPAN |
| 1401 | Saitama Prefectural Cancer Center Institutional Review Board<br>780 Oaza Komuro, Inamachi<br>Kita-adachi-gun, SAITAMA 362-0806<br>JAPAN |
| 1402 | National Hospital Organization Osaka National Hospital Institutional Review Board<br>2-1-14, Hoenzaka, Chuo-ku<br>Osaka, OSAKA 540-0006<br>JAPAN |
| 1403 | St. Marianna University Group institutional Review Board<br>2-16-1 sugao miyamaeku<br>kawasaki-shi, KANAGAWA 2168511<br>JAPAN |
| 1404 | national Hospital organization Kyushu Cancer Center Institutional Review Board<br>3-1-1,Notame Minamiku<br>Fukuoka, FUKUOKA 8111395<br>JAPAN |
| 1410 | Keio University Hospital IRB<br>35, Shinano-machi<br>Shinjuku-ku, TOKYO 160-8582<br>JAPAN |
| 1411 | Saitama Medical University International Medical Center Institutional Review Board<br>1397-1 Yamane, NA<br>Hidaka, SAITAMA 350-1298<br>JAPAN |
| 1412 | chiba cancer center Institutional Review Board<br>666-2 Nitona-cho,Chuo-Ku<br>Chiba City, CHIBA 2608717<br>JAPAN |
| 1413 | Osaka Medical and Pharmaceutical University Hospital Institutional Review Board<br>2-7, Daigaku-machi<br>Takatsuki-shi, OSAKA 569-8686 |

| | |
|---|---|
| | JAPAN |
| 1414 | Hokkaido University Hospital Institutional Review Board<br>Kita 14, Nishi 5, Kita-ku<br>Sapporo, HOKKAIDO 060-8648<br>JAPAN |
| 1415 | Kindai University Hospital Institutional Review Board<br>377-2 Ohnohigashi<br>Osakasayama, OSAKA 589-8511<br>JAPAN |
| 1296 | IRB of Asan Medical Center<br>88, Olympic-Ro 43-gil, Songpa-gu<br>Seoul, 05505<br>KOREA, REPUBLIC OF |
| 1318 | Samsung Medical Center IRB<br>81 Irwon-Ro, Gangnam-Gu<br>Seoul, 06351<br>KOREA, REPUBLIC OF |
| 1351 | National Cancer Center IRB<br>323 Ilsan-ro, Ilsandong-gu<br>Goyang-si, GYEONGGIDO 10408<br>KOREA, REPUBLIC OF |
| 1352 | Korea University Anam Hospital - IRB<br>73 Goryeodae-ro, Seongbuk-gu<br>Seoul, 02841<br>KOREA, REPUBLIC OF |
| 1355 | Dong-A University Hospital IRB<br>26 Daesingongwon-ro, Seo-gu<br>Busan, 49201<br>KOREA, REPUBLIC OF |
| 1376 | Chonnam National University Hwasun Hospital IRB<br>322 Seoyang-ro, Hwasun-eup, Hwansun-gun<br>Jeonnam, 58128<br>KOREA, REPUBLIC OF |
| 1405 | Gachon University Gil Medical Center IRB<br>21, Namdong-daero 774 beon-gil, Namdong-gu<br>Incheon, 21565<br>KOREA, REPUBLIC OF |
| 1407 | Severance Hospital IRB<br>50-1 Yonsei-ro, Seodaemun-gu<br>Seoul, 03722<br>KOREA, REPUBLIC OF |
| 1443 | Seoul National University Hospital IRB<br>101 Daehak-ro, Jongno-gu<br>Seoul, 03080<br>KOREA, REPUBLIC OF |
| 1444 | gpook National University Chilgok Hospital Institutional Review Board<br>807 Hoguk-ro, Buk-gu<br>Daegu, 41404<br>KOREA, REPUBLIC OF |
| 1141 | Investigación Biomédica para el Desarrollo de Fármacos<br>3352 Calle Volcán Popocatépetl,Colli Urbano<br>Zapopan, JALISCO 45070<br>MEXICO |
| 1150 | Accelerium, S. de R.L. de C.V.<br>Calle Modesto Arreola 917 Oriente, Centro<br>Monterrey, NUEVO LEON 64000<br>MEXICO |
| 1011 | METC Brabant<br>Dr. Deelenlaan 9<br>Tilburg, 5000 LC<br>NETHERLANDS |
| 1120 | METC Brabant<br>Dr. Deelenlaan 9<br>Tilburg, 5000 LC<br>NETHERLANDS |
| 1230 | METC Brabant |

| | |
|---|---|
| | Dr. Deelenlaan 9<br>Tilburg, 5000 LC<br>NETHERLANDS |
| 1256 | METC Brabant<br>Dr. Deelenlaan 9<br>Tilburg, 5000 LC<br>NETHERLANDS |
| 1316 | METC Brabant<br>Dr. Deelenlaan 9, Gebouw Hasseltveste<br>Tilburg, 5000 LC<br>NETHERLANDS |
| 1353 | METC Brabant<br>Hasseltveste Dr. Deelenlaan 9<br>Tilburg, NOORD-BRABANT 5042 AD<br>NETHERLANDS |
| 1125 | Health and Disability Ethics Committees<br>Ministry of Health<br>133 Molesworth Street,PO Box 5013<br>Wellington, 6011<br>NEW ZEALAND |
| 1202 | Health and Disability Ethics Committees<br>Ministry of Health<br>133 Molesworth Street,PO Box 5013<br>Wellington, 6011<br>NEW ZEALAND |
| 1055 | REC South-East B<br>Gullhaugveien 1-3<br>Oslo, 0484<br>NORWAY |
| 1059 | REC South-East B<br>Gullhaugveien 1-3<br>Oslo, 0484<br>NORWAY |
| 1060 | REC South-East B<br>Gullhaugveien 1-3<br>Oslo, 0484<br>NORWAY |
| 1117 | REC South-East B<br>Gullhaugveien 1-3<br>Oslo, 0484<br>NORWAY |
| 1291 | REC South-East B<br>Gullhaugveien 1-3<br>Oslo, 0484<br>NORWAY |
| 1135 | Komisja Bioetyczna przy Okręgowej Izbie Lekarskiej w Gdańsku<br>ul. Śniadeckich 33<br>Gdańsk, 80-204<br>POLAND |
| 1335 | Komisja Bioetyczna przy Okręgowej Izbie Lekarskiej w Gdańsku<br>ul. Śniadeckich 33<br>Gdańsk, 80-204<br>POLAND |
| 1363 | Komisja Bioetyczna przy Okręgowej Izbie Lekarskiej w Gdańsku<br>ul. Śniadeckich 33<br>Gdańsk, 80-204<br>POLAND |
| 1372 | Komisja Bioetyczna przy Okręgowej Izbie Lekarskiej w Gdańsku<br>ul. Śniadeckich 33<br>Gdańsk, 80-204<br>POLAND |
| 1109 | LEC at Kaluga Regional Clinical Oncology Center<br>2, Vishnevskogo str.<br>Kaluga, 248007<br>RUSSIAN FEDERATION |
| 1111 | Independent Ethics Committee LLC "Komanda" |

| | |
|---|---|
| | bld.2/A, 19, Frunze str.<br>Saint-Petersburg, 196135<br>RUSSIAN FEDERATION |
| 1112 | Independent Interdisciplinary Committee at Clinical Trials Ethical Expertise<br>Leningradskiy Prospekt, 51<br>Moscow, 125458<br>RUSSIAN FEDERATION |
| 1113 | Ethics Committee at the ANO Development of Research Studies in Medicine<br>ul. Instrumentalnaya, d. 3, liter K, chast. Pom. 15N, komn. 93<br>Saint Petersburg, 197022<br>RUSSIAN FEDERATION |
| 1114 | Ethics Committee LLC ¨Komanda¨<br>bld.2/A, 19, Frunze str.<br>Saint-Petersburg,, 196135<br>RUSSIAN FEDERATION |
| 1115 | Interdisciplinary Ethics Committee of Omsk region<br>43, Kuibysheva street, office 632<br>Omsk, 644070<br>RUSSIAN FEDERATION |
| 1116 | LEC at Federal State Budgetary Educational Institution of Higher Education “National Research Ogarev Mordovia State University” (MRSU), Medical Institute<br>26a, Ulyanova str.<br>Saransk, REPUBLIC OF MORDOVIA 430032<br>RUSSIAN FEDERATION |
| 1183 | LEC at Republican Clinical Oncology Dispensary of the Ministry of Health of Bashkortostan Republic<br>Prospekt Oktyabrya 73/1<br>Ufa, 450054<br>RUSSIAN FEDERATION |
| 1281 | Local Ethics Committee of State Budgetary Healthcare Institution “City Clinical Hospital #1”<br>7, Golovko street<br>Nalchik, KABARDINO-BALKARIAN REPUBLIC 360003<br>RUSSIAN FEDERATION |
| 1282 | Local Ethics Committee of SBHI "Orenburg Regional Clinical Oncological Dispensary"<br>11, Gagarina prospect<br>Orenburg, 460021<br>RUSSIAN FEDERATION |
| 1312 | Local Ethics Committee at FSAEI HE I.M Sechenov First MSMU MoH Russia (Sechenovskiy<br>University)<br>ul. Trubetskaya, d. 8<br>Moscow, 119991<br>RUSSIAN FEDERATION |
| 1313 | Ethics Committee at SBIH "Chelyabinsk Regional Clinical Centre of Oncology and Nuclear Medicine"<br>42, Blukhera str.<br>Chelyabinsk, 454087<br>RUSSIAN FEDERATION |
| 1108 | Eticka komisia Fakultna nemocnica s poliklinikou F.D. Roosevelta<br>Nam. L. Svobodu 1<br>Banska Bystrica, 975 17<br>SLOVAKIA<br><br>Eticka komisia Narodny onkologicky ustav<br>Klenova 1<br>Bratislava, BRATISLAVSKÝ KRAJ 833 10<br>SLOVAKIA |
| 1147 | Eticka komisia Fakultna nemocnica s poliklinikou F.D. Roosevelta<br>Nam. L. Svobodu 1<br>Banska Bystrica, 975 17<br>SLOVAKIA<br><br>Eticka komisia, Vychodoslovensky onkologicky ustav, a.s.<br>Rastislavova 43<br>Kosice, 041 91 |

| | |
|---|---|
| | SLOVAKIA |
| 1224 | Eticka komisia Fakultna nemocnica s poliklinikou F.D. Roosevelta Banska Bystrica<br>Nám. L. Svobodu 1<br>Banska Bystrica, 975 17<br>SLOVAKIA<br><br>Eticka komisia Onkologicky ustav sv. Alzbety, s.r.o.<br>Heydukova 10<br>Bratislava, 812 50<br>SLOVAKIA |
| 1255 | Eticka komisia Fakultna nemocnica s poliklinikou F.D. Roosevelta Banska Bystrica<br>Námestie L. Svobodu 1<br>Banská Bystrica, 975 17<br>SLOVAKIA<br><br>Eticka komisia Urad Presovskeho samospravneho kraja<br>Namestie Mieru 2<br>Presov, 080 01<br>SLOVAKIA |
| 1328 | Eticka komisia Fakultna nemocnica s poliklinikou F.D. Roosevelta<br>Nam. L. Svobodu 1<br>Banska Bystrica, 975 17<br>SLOVAKIA |
| 1173 | Pharma Ethics<br>123 Amkor Road, Lyttelton Manor Ext 3<br>Centurion, GAUTENG 0157<br>SOUTH AFRICA |
| 1174 | Pharma Ethics<br>123 Amkor Road, Lyttelton Manor Ext 3<br>Centurion, GAUTENG 0157<br>SOUTH AFRICA |
| 1270 | Pharma Ethics<br>123 Amkor Road, Lyttelton Manor Ext 3<br>Centurion, GAUTENG 0157<br>SOUTH AFRICA |
| 1287 | Pharma Ethics<br>123 Amkor Road, Lyttelton Manor Ext 3<br>Centurion, GAUTENG 0157<br>SOUTH AFRICA |
| 1308 | University of the Witwatersrand Human Research Ethics Committee: (Medical)<br>Private Bag x2600,Suite 189<br>Houghton, JOHANNESBURG 2041<br>SOUTH AFRICA |
| 1013 | Comité de Ética de Investigación Clínica del Hospital Universitario 12 de Octubre<br>Av. de Cordoba s/n<br>Madrid, MADRID 28041<br>SPAIN |
| 1019 | Comité de Ética de Investigación Clínica del Hospital Universitario 12 de Octubre<br>Av. de Cordoba s/n<br>Madrid, MADRID 28041<br>SPAIN |
| 1033 | Comité de Ética de Investigación con medicamentos del Hospital Universitario 12 de Octubre<br>Av. de Cordoba s/n, Centro de Actividades Ambulatorias, Bloque D, Planta 6<br>Madrid, 28041<br>SPAIN |
| 1034 | Comité de Ética de Investigación con medicamentos del Hospital Universitario 12 de Octubre<br>Av. de Cordoba s/n, Centro de Actividades Ambulatorias, Bloque D, Planta 6<br>Madrid, 28041<br>SPAIN |
| 1035 | Comité de Ética de Investigación Clínica del Hospital Universitario 12 de Octubre<br>Av. de Cordoba s/n<br>Madrid, MADRID 28041<br>SPAIN |
| 1036 | Comité de Ética de Investigación Clínica del Hospital Universitario 12 de Octubre |

| | |
|---|---|
| | Av. de Cordoba s/n<br>Madrid, MADRID 28041<br>SPAIN |
| 1037 | Comité de Ética de Investigación con medicamentos del Hospital Universitario 12 de Octubre<br>Av. de Cordoba s/n, Centro de Actividades Ambulatoria, Bloque D, Planta 6<br>Madrid, 28041<br>SPAIN |
| 1038 | Comité de Ética de Investigación Clínica del Hospital Universitario 12 de Octubre<br>Av. de Cordoba s/n<br>Madrid, MADRID 28041<br>SPAIN |
| 1144 | Comité de Ética de Investigación Clínica del Hospital Universitario 12 de Octubre<br>Av. de Cordoba s/n<br>Madrid, MADRID 28041<br>SPAIN |
| 1148 | Comité de Ética de Investigación Clínica del Hospital Universitario 12 de Octubre<br>Av. de Cordoba s/n<br>Madrid, MADRID 28041<br>SPAIN |
| 1171 | Comité de Ética de Investigación Clínica del Hospital Universitario 12 de Octubre<br>Av. de Cordoba s/n<br>Madrid, MADRID 28041<br>SPAIN |
| 1286 | Comité de Ética de Investigación Clínica del Hospital Universitario 12 de Octubre<br>Av. de Cordoba s/n<br>Madrid, MADRID 28041<br>SPAIN |
| 1153 | Etikprövningsmyndigheten<br>Box 2110<br>Uppsala, 750 02<br>SWEDEN |
| 1187 | Etikprövningsmyndigheten<br>Box 2110<br>Uppsala, 750 02<br>SWEDEN |
| 1237 | Etikprövningsmyndigheten<br>Box 2110<br>Uppsala, 750 02<br>SWEDEN |
| 1371 | Etikprövningsmyndigheten<br>Box 2110<br>Uppsala, 750 02<br>SWEDEN |
| 1063 | Institutional Review Board of Taipei Veterans General Hospital<br>201, Shih-Pai Road, Section 2,<br>Taipei, 11217<br>TAIWAN |
| 1072 | Research Ethics Committee China Medical University & Hospital<br>No.2 Yude Road<br>Taichung, TAICHUNG 40447<br>TAIWAN |
| 1088 | Institutional Review Board of Kaohsiung Medical University Chung-Ho Memorial Hospital<br>No. 100, Tzyou 1st Road, Kaohsiung 807, Taiwan<br>Kaohsiung, KAOHSIUNG 807<br>TAIWAN |
| 1137 | National Taiwan University Hospital Research Ethics Committee<br>7, Chung-Shan South Road<br>Taipei, 100<br>TAIWAN |
| 1156 | Chang Gung Medical Foundation Institutional Review Board<br>199, TUNG HWA NORTH ROAD<br>Taipei, 10507<br>TAIWAN |
| 1158 | TMU-Joint Institutional Review Board<br>No.172-1, Sec. 2, Keelung Rd.,17F. |

| | |
|---|---|
| | Taipei, TAIPEI 10675<br>TAIWAN |
| 1175 | Chi Mei Medical Center Institutional Review Board<br>No. 901, Zhonghua Road, Yongkang District<br>Tainan, TAINAN 71004<br>TAIWAN |
| 1374 | Institutional Review Board National Cheng Kung University Hospital<br>138 Sheng-Li Rd.,<br>Tainan, 704<br>TAIWAN |
| 1062 | EC at Municipal non-profit enterprise "Zaporizhzhia Regional Antitumor Center" ZRC<br>177a Kulturna str.<br>Zaporizhzhia, 69040<br>UKRAINE |
| 1066 | Ethics Commission of Communal Non-Profit Enterprise "Regional Center of Oncology"<br>4 Lisoparkivska Street<br>Kharkiv, 61070<br>UKRAINE |
| 1070 | EC at MNPE "Prykarpatski Clinical Oncological Center" of Ivano-Frankivsk Regional Council<br>17 Medychna Str.<br>Ivano-Frankivsk, 76018<br>UKRAINE |
| 1076 | Ethics Commission at MNE City Clinical Hospital #4 of Dnipro City Council<br>31 Blyzhnia str.<br>Dnipro, 49102<br>UKRAINE |
| 1077 | EC at Communal enterprise "Kryvyi Rih Oncology Dispensary" of Dnipropetrovsk Regional Council<br>41, Dniprovske shose<br>Kryvyi Rih, 50048<br>UKRAINE |
| 1089 | Ethics Committee at "MedX-ray International Group", LLC LISOD - Israeli Oncological Hospital<br>vul. A. Malyshka, bud. 27<br>Pliuty village, KYIV REGION, OBUKHIV DISTRICT 08720<br>UKRAINE |
| 1105 | Ethics Commission at the Municipal Institution of Kyiv Regional Council<br>vul Bahhovutivska 1, Kyiv Regional Clinical Hospital<br>Kyiv-107, 04106<br>UKRAINE |
| 1314 | Ethics Committee at MNP enterprise "CCH #2 named after O.O.Shalimov" of Kharkiv City Council<br>197 Moskovskyi avenue<br>Kharkiv, 61037<br>UKRAINE |
| 1239 | East of England - Essex Research Ethics Committee<br>The Old Chapel,Royal Standard Place<br>Nottingham, NOTTINGHAM NG1 6FS<br>UNITED KINGDOM |
| 1301 | East of England - Essex Research Ethics Committee<br>The Old Chapel,Royal Standard Place<br>Nottingham, NOTTINGHAM NG1 6FS<br>UNITED KINGDOM |
| 1343 | East of England - Essex Research Ethics Committee<br>The Old Chapel,Royal Standard Place<br>Nottingham, NOTTINGHAM NG1 6FS<br>UNITED KINGDOM |
| 1359 | East of England - Essex Research Ethics Committee<br>The Old Chapel,Royal Standard Place<br>Nottingham, NOTTINGHAM NG1 6FS<br>UNITED KINGDOM |
| 1445 | East of England - Essex Research Ethics Committee<br>The Old Chapel,Royal Standard Place<br>Nottingham, NOTTINGHAM NG1 6FS<br>UNITED KINGDOM |

| | |
|---|---|
| 1455 | East of England - Essex Research Ethics Committee<br>The Old Chapel,Royal Standard Place<br>Nottingham, NOTTINGHAM NG1 6FS<br>UNITED KINGDOM |
| 1001 | The University of Texas MD Anderson Cancer Center Institutional Review Board<br>7007 Bertner Avenue, Unit 1637<br>Houston, TEXAS 77030-4009<br>UNITED STATES |
| 1002 | WCG IRB<br>1019 39th Avenue SE,Suite 120<br>Puyallup, WASHINGTON 98374<br>UNITED STATES |
| 1003 | Memorial Sloan Kettering Cancer Center Institutional Review Board/Privacy Board<br>1275 York Avenue<br>New York, 10065<br>UNITED STATES<br><br>WCG IRB<br>1019 39th Avenue SE,Suite 120<br>Puyallup, WASHINGTON 98374<br>UNITED STATES |
| 1004 | WCG IRB (Inactive IRB/IEC)<br>1019 39th Ave SE,Suite 120<br>Puyallup, WA 98374<br>UNITED STATES<br><br>WCG IRB<br>212 Carnegie Center, Suite 301<br>Princeton, NEW JERSEY 08540<br>UNITED STATES |
| 1005 | University of Southern California (USC) Institutional Review Board<br>3720 S. Flower Street,Suite 325<br>Los Angeles, CALIFORNIA 90089<br>UNITED STATES |
| 1006 | WCG Institutional Review Board<br>1019 39th Ave SE,Ste 120<br>PuyalluP, WA 98374-2115<br>UNITED STATES |
| 1007 | WCGIRB<br>1019 39th Avenue SE,Suite 120<br>Puyallup, WASHINGTON 98374<br>UNITED STATES |
| 1009 | Medical College of Wisconsin and Froedtert Hospital Institutional Review Board<br>8701 Watertown Plank Road<br>Milwaukee, WISCONSIN 53226<br>UNITED STATES |
| 1015 | WCG IRB<br>1019 39th Avenue SE,Suite 120<br>Puyallup, WASHINGTON 98374<br>UNITED STATES |
| 1020 | WCG IRB<br>1019 39th Avenue SE,Suite 120<br>Puyallup, WASHINGTON 98374<br>UNITED STATES |
| 1021 | WCG IRB (Inactive IRB/IEC)<br>1019 39th Avenue Southeast,Suite 120<br>Puyallup, WASHINGTON 98374<br>UNITED STATES<br><br>WCG IRB<br>212 Carnegie Center,Suite 301<br>Princeton, NEW JERSEY 08540<br>UNITED STATES |
| 1025 | Advarra<br>6100 Merriweather Dr.,Suite 600<br>Columbia, MARYLAND 21044 |

| | |
|---|---|
| | UNITED STATES |
| 1027 | Mayo Clinic Institutional Review Board<br>200 First Street SW,201 Building, Room 4-60<br>Rochester, MINNESOTA 55905<br>UNITED STATES |
| 1041 | WCG IRB<br>1019 39th Avenue SE,Suite 120<br>Puyallup, WASHINGTON 98374<br>UNITED STATES |
| 1045 | WCG IRB<br>1019 39th Avenue SE,Suite 120<br>Puyallup, WASHINGTON 98374<br>UNITED STATES |
| 1046 | WCG IRB<br>1019 39th Avenue SE,Suite 120<br>Puyallup, WASHINGTON 98374<br>UNITED STATES |
| 1047 | WCG IRB<br>1019 39th Avenue SE,Suite 120<br>Puyallup, WASHINGTON 98374<br>UNITED STATES |
| 1048 | WCG IRB<br>1019 39th Avenue SE,Suite 120<br>Puyallup, WASHINGTON 98374<br>UNITED STATES |
| 1049 | WCG IRB (Inactive IRB/IEC)<br>1019 39th Avenue Southeast,Suite 120<br>Puyallup, WASHINGTON 98374<br>UNITED STATES<br><br>WCG IRB<br>212 Carnegie Center,Suite 301<br>Princeton, NEW JERSEY 08540<br>UNITED STATES |
| 1050 | ADVARRA IRB<br>6100 Merriweather Dr,Suite 600<br>Columbia, MARYLAND 21044<br>UNITED STATES |
| 1056 | WCGIRB<br>1019 39th Ave SE,Ste 120<br>Puyallup, WA 98374<br>UNITED STATES |
| 1073 | Cleveland Clinic Foundation Institutional Review Board<br>9500 Euclid Ave<br>Cleveland, OH 44195<br>UNITED STATES |
| 1101 | St. Joseph's/Candler IRB<br>5354 Reynolds Street, Suite 328<br>Savannah, GEORGIA 31405<br>UNITED STATES |
| 1102 | WCG IRB<br>1019 39th Avenue SE,Suite 120<br>Puyallup, WASHINGTON 98374<br>UNITED STATES |
| 1155 | University of Miami Human Subject Research Office (HSRO)- Institutional Review Board<br>Gables One Tower, 1320 S Dixie Highway, #650<br>Coral Gables, FLORIDA 33146<br>UNITED STATES |
| 1168 | Vanderbilt University Institutional Review Board<br>3319 West End Avenue, Suite 600<br>Nashville, TN 37203<br>UNITED STATES |
| 1201 | Cedars-Sinai Medical Center; Office of Research Compliance and Quality Improvement<br>6500 Wilshire Blvd.,Ste 1800<br>Los Angeles, CA 90048<br>UNITED STATES |

| | |
|---|---|
| 1216 | Ochsner Clinic Foundation Institutional Review Board<br>1514 Jefferson Highway<br>New Orleans, LOUISIANA 70121<br>UNITED STATES |
| 1266 | Mayo Clinic Institutional Review Board<br>200 First Street Southwest<br>Rochester, MINNESOTA 55905<br>UNITED STATES |
| 1321 | WCG IRB<br>1019 39th Avenue SE, Suite 120<br>Puyallup, WASHINGTON 98374-2115<br>UNITED STATES |
| 1340 | Mayo Clinic Institutional Review Board<br>200 First St SW<br>Rochester, MN 55905<br>UNITED STATES |
| 1370 | WCG IRB<br>1019 39th Avenue SE,Suite 120<br>Puyallup, WASHINGTON 98374<br>UNITED STATES |
| 1373 | BSD IRB, The University of Chicago Biological Sciences Division/University of Chicago Medical<br>Center<br>5841 S Maryland Avenue, MC7132, I-625<br>Chicago, ILLINOIS 60637<br>UNITED STATES |
| 1377 | WCG IRB<br>1019 39th Avenue SE,Suite 120<br>Puyallup, WASHINGTON 98374<br>UNITED STATES |
| 1380 | WCG IRB<br>1019 39th Avenue Southeast,Suite 120<br>Puyallup, WASHINGTON 98374<br>UNITED STATES |
| 1406 | WCG IRB<br>1019 39th Avenue SE,Suite 120<br>Puyallup, WASHINGTON 98374<br>UNITED STATES |
| 1452 | WCG IRB<br>1019 39th Avenue SE,Suite 120<br>Puyallup, WASHINGTON 98374<br>UNITED STATES |

This supplement contains the following items:

1. Original protocol
2. Final protocol
   *The protocol summary of changes is included as Appendix 13 within the final protocol*
3. Original statistical analysis plan
4. Final statistical analysis plan
   *The statistical analysis plan summary of changes is included as Table 1 within the final statistical analysis plan*

# AN OPEN-LABEL, MULTICENTER, RANDOMIZED PHASE 3 STUDY OF FIRST-LINE ENCORAFENIB PLUS CETUXIMAB WITH OR WITHOUT CHEMOTHERAPY VERSUS STANDARD OF CARE THERAPY WITH A SAFETY LEAD-IN OF ENCORAFENIB AND CETUXIMAB PLUS CHEMOTHERAPY IN PARTICIPANTS WITH METASTATIC BRAF V600E-MUTANT COLORECTAL CANCER

| | |
|---|---|
| **Study Intervention Number:** | PF-07263896; LGX818 |
| **Study Intervention Name:** | Encorafenib |
| **US IND Number:** | 115298 |
| **EudraCT Number:** | 2020-001288-99 |
| **Protocol Number:** | C4221015 |
| **Phase:** | 3 |

**SHORT TITLE:** The BREAKWATER Study (***BR****AF* V600E-mutant colorectal cancer study evaluating **E**ncor**A**fenib ta**K**en **W**ith cetuxim**A**b plus or minus chemo**T**h**ER**apy)

This document and accompanying materials contain confidential information belonging to Pfizer. Except as otherwise agreed to in writing, by accepting or reviewing these documents, you agree to hold this information in confidence and not copy or disclose it to others (except where required by applicable law) or use it for unauthorized purposes. In the event of any actual or suspected breach of this obligation, Pfizer must be promptly notified.

090177e194745c57\Approved\Approved On: 22-Jul-2020 00:48 (GMT)

## Protocol Amendment Summary of Changes Table

| **Document History** | | |
|---|---|---|
| **Document** | **Version Date** | **Summary and Rationale for Changes** |
| Original protocol | 15 July 2020 | N/A |

090177e194745c57\Approved\Approved On: 22-Jul-2020 00:48 (GMT)

## TABLE OF CONTENTS

LIST OF TABLES ....................10
LIST OF FIGURES ....................11
1. PROTOCOL SUMMARY ....................12
1.1. Synopsis ....................12
1.2. Schema ....................16
1.3. Schedule of Activities ....................17
2. INTRODUCTION ....................42
2.1. Study Rationale ....................42
2.2. Background ....................42
2.2.1. *BRAF* V600E-Mutant Metastatic Colorectal Cancer ....................42
2.2.2. Clinical Overview ....................43
2.2.2.1. Current Recommendations for Treatment of BRAF V600E-mutant mCRC ....................43
2.2.2.2. Encorafenib + Cetuximab for the Treatment of BRAF V600E-mutant mCRC ....................43
2.3. Benefit/Risk Assessment ....................45
2.3.1. Risk Assessment ....................47
2.3.2. Benefit Assessment ....................51
2.3.3. Overall Benefit/Risk Conclusion ....................51
3. OBJECTIVES, ESTIMANDS, AND ENDPOINTS ....................51
4. STUDY DESIGN ....................53
4.1. Overall Design ....................53
4.2. Scientific Rationale for Study Design ....................54
4.2.1. Safety Lead-in ....................54
4.2.2. Assessment of Potential DDI with Encorafenib ....................54
4.2.3. Investigator's Choice of Control Regimen ....................55
4.2.4. Progression-Free Survival as Primary Endpoint ....................55
4.2.5. Biomarkers ....................56
4.2.6. Use of Contraceptives ....................57
4.3. Justification for Dose ....................57
4.4. End of Study Definition ....................57
5. STUDY POPULATION ....................57



090177e194745c57\Approved\Approved On: 22-Jul-2020 00:48 (GMT)

5.1. Inclusion Criteria ....58
5.1.1. Molecular Prescreening Inclusion Criteria ....58
5.1.2. Screening Inclusion Criteria ....59
5.2. Exclusion Criteria ....61
5.2.1. Molecular Prescreening Exclusion Criteria ....61
5.2.2. Screening Exclusion Criteria ....62
5.3. Lifestyle Considerations ....64
5.3.1. Contraception ....64
5.3.2. Meals and Dietary Restrictions ....64
5.4. Screen Failures ....64
6. STUDY INTERVENTION ....65
6.1. Study Interventions Administered ....66
6.1.1. Safety Lead-in ....67
6.1.1.1. Dose Limiting Toxicity Definition ....68
6.1.2. Treatment Regimens ....70
6.1.3. Administration ....73
6.1.3.1. Administration of Encorafenib ....73
6.1.3.2. Administration of Capecitabine, Cetuximab, 5-Fluorouracil, Irinotecan, Leucovorin, Oxaliplatin and Bevacizumab ....74
6.2. Preparation/Handling/Storage/Accountability ....74
6.2.1. Preparation and Dispensing ....75
6.3. Measures to Minimize Bias: Randomization and Blinding ....75
6.3.1. Allocation to Study Intervention ....75
6.4. Study Intervention Compliance ....76
6.5. Concomitant Therapy ....76
6.5.1. Permitted Concomitant Medications/Therapies ....76
6.5.1.1. Surgical Resection ....77
6.5.1.2. CYP and UGT Substrates and Inhibitors ....77
6.5.1.3. Transporter Substrates and Inhibitors ....78
6.5.1.4. Drugs with a Conditional or Possible Risk to Prolong the QT Interval and/or Induce Torsade de Pointes ....78
6.5.2. Prohibited Concomitant Therapy ....78

090177e194745c57\Approved\Approved On: 22-Jul-2020 00:48 (GMT)

6.5.3. Premedication and Concurrent Medication ....79
6.5.3.1. Premedication for Cetuximab Administration ....79
6.5.3.2. Loperamide ....79
6.5.3.3. Antibiotics ....79
6.5.3.4. Atropine ....80
6.6. Dose Modification ....80
6.6.1. Dose Modifications for Encorafenib ....81
6.6.2. Dose Modifications for Cetuximab ....84
6.6.3. Dose Modifications for Oxaliplatin, Irinotecan and 5-FU as Part of mFOLFOX6, FOLFIRI, and FOLFOXIRI Regimens ....85
6.6.4. Dose Modifications for Capecitabine ....90
6.6.5. Dose Modifications for Bevacizumab ....91
6.6.6. Guidance For Administration of Study Intervention in Participants with SARS-CoV-2 Infection ....94
6.7. Intervention After the End of the Study ....94
7. DISCONTINUATION OF STUDY INTERVENTION AND PARTICIPANT DISCONTINUATION/WITHDRAWAL ....95
7.1. Discontinuation of Study Intervention ....95
7.2. Participant Discontinuation/Withdrawal From the Study ....96
7.2.1. Withdrawal of Consent/Assent ....97
7.3. Lost to Follow-up ....97
8. STUDY ASSESSMENTS AND PROCEDURES ....98
8.1. Efficacy Assessments ....99
8.1.1. Tumor Response Assessments ....99
8.1.1.1. BICR Evaluation of Imaging Data ....100
8.1.2. Patient-reported Outcomes ....102
8.2. Safety Assessments ....103
8.2.1. Participant Demographics and Other Baseline Characteristics ....104
8.2.2. Disease Characteristics and Treatment History ....104
8.2.3. Physical Examinations ....104
8.2.4. Body Surface Area ....104
8.2.5. Dermatological Examinations ....105
8.2.6. Vital Signs ....105

090177e194745c57\Approved\Approved On: 22-Jul-2020 00:48 (GMT)

8.2.7. Electrocardiograms ....105
8.2.8. Clinical Safety Laboratory Assessments ....106
8.2.9. Pregnancy Testing ....107
8.2.10. ECOG Performance Status ....107
8.3. Adverse Events and Serious Adverse Events ....107
8.3.1. Time Period and Frequency for Collecting AE and SAE Information ....108
8.3.1.1. Reporting SAEs to Pfizer Safety ....108
8.3.1.2. Recording Nonserious AEs and SAEs on the CRF ....109
8.3.2. Method of Detecting AEs and SAEs ....109
8.3.3. Follow-up of AEs and SAEs ....109
8.3.4. Regulatory Reporting Requirements for SAEs ....109
8.3.5. Exposure During Pregnancy or Breastfeeding, and Occupational Exposure ....110
8.3.5.1. Exposure During Pregnancy ....110
8.3.5.2. Exposure During Breastfeeding ....111
8.3.5.3. Occupational Exposure ....112
8.3.6. Cardiovascular and Death Events ....112
8.3.7. Disease-Related Events and/or Disease-Related Outcomes Not Qualifying as AEs or SAEs ....112
8.3.8. Adverse Events of Special Interest ....113
8.3.8.1. Lack of Efficacy ....113
8.3.9. Medical Device Deficiencies ....113
8.3.10. Medication Errors ....113
8.4. Treatment of Overdose ....114
8.5. Pharmacokinetics ....114
8.5.1. Pharmacokinetics for Participants in China ....117
8.6. Pharmacodynamics ....117
8.7. Genetics ....118
8.7.1. Specified Genetics ....118
8.7.2. *BRAF* Testing ....118
8.7.3. Molecular Prescreening ....119
8.7.4. Screening ....120
8.7.5. Companion Diagnostic for Eligibility ....120



090177e194745c57\Approved\Approved On: 22-Jul-2020 00:48 (GMT)

8.7.6. Companion Diagnostic Assay Development ..........120
8.7.7. Banked Biospecimens for Genetics ..........121
8.8. Biomarkers ..........121
8.8.1. CRP and CEA assessments ..........122
8.8.2. Tumor Tissue Assessments ..........122
8.8.3. Circulating Free DNA (cfDNA) ..........123
8.8.4. Specified Gene Expression (RNA) Research ..........123
8.8.5. Specified Protein Research ..........123
8.8.6. Specified Metabolomic Research ..........124
8.9. Immunogenicity Assessments ..........124
8.10. Health Economics ..........124
9. STATISTICAL CONSIDERATIONS ..........124
9.1. Estimands and Statistical Hypotheses ..........124
9.1.1. Estimands ..........124
9.1.2. Statistical Hypotheses ..........128
9.2. Sample Size Determination ..........128
9.3. Analysis Sets ..........131
9.4. Statistical Analyses ..........132
9.4.1. General Considerations ..........132
9.4.1.1. Statistical Testing Strategy ..........132
9.4.1.2. Pooling of Centers ..........134
9.4.1.3. Stratification Factors ..........134
9.4.1.4. Definition of Baseline ..........135
9.4.1.5. Definition of On-treatment Period ..........135
9.4.2. Primary Endpoints ..........135
9.4.3. Secondary Endpoints ..........137
9.4.3.1. OS ..........137
9.4.3.2. Objective Response Rate ..........138
9.4.3.3. Duration of Response ..........138
9.4.3.4. Time to Response ..........139
9.4.3.5. PFS (Safety Lead-in) ..........139
9.4.3.6. PFS2 ..........139



090177e194745c57\Approved\Approved On: 22-Jul-2020 00:48 (GMT)

9.4.3.7. Patient Reported Outcomes ....................................................139
9.4.3.8. Pharmacokinetic Analyses ....................................................141
9.4.3.9. Exposure-Response of Encorafenib ........................................141
9.4.3.10. Biomarker Analyses ...........................................................141
9.4.4. Tertiary/Exploratory Endpoints ...............................................141
9.4.5. Safety Analyses ......................................................................142
9.4.5.1. Adverse Events .....................................................................142
9.4.5.2. Clinical Laboratory Results ...................................................142
9.4.5.3. Electrocardiogram Analyses ..................................................142
9.4.6. Other Analyses .......................................................................142
9.4.6.1. Subgroup Analyses ...............................................................143
9.5. Interim Analyses ........................................................................143
9.5.1. Interim Futility Analysis for PFS ..............................................143
9.5.2. Interim Efficacy Analysis for OS ...............................................143
9.6. Data Monitoring Committee or Other Independent Oversight Committee..........144
10. SUPPORTING DOCUMENTATION AND OPERATIONAL CONSIDERATIONS ..........145
10.1. Appendix 1: Regulatory, Ethical, and Study Oversight Considerations ............145
10.1.1. Regulatory and Ethical Considerations ....................................145
10.1.1.1. Reporting of Safety Issues and Serious Breaches of the Protocol or ICH GCP ..........145
10.1.2. Financial Disclosure ................................................................146
10.1.3. Informed Consent/Assent Process ............................................146
10.1.4. Data Protection .......................................................................147
10.1.5. Dissemination of Clinical Study Data .......................................148
10.1.6. Data Quality Assurance ...........................................................149
10.1.7. Source Documents ...................................................................150
10.1.8. Study and Site Start and Closure .............................................150
10.1.9. Publication Policy ...................................................................151
10.1.10. Sponsor's Qualified Medical Personnel ...................................152
10.2. Appendix 2: Clinical Laboratory Tests ...........................................153
10.3. Appendix 3: Adverse Events: Definitions and Procedures for Recording, Evaluating, Follow-up, and Reporting ..........154



090177e194745c57\Approved\Approved On: 22-Jul-2020 00:48 (GMT)

10.3.1. Definition of AE .....154
10.3.2. Definition of SAE .....155
10.3.3. Recording/Reporting and Follow-up of AEs and/or SAEs.....157
10.3.4. Reporting of SAEs.....160
10.4. Appendix 4: Contraceptive Guidance .....162
10.4.1. Male Participant Reproductive Inclusion Criteria .....162
10.4.2. Female Participant Reproductive Inclusion Criteria.....162
10.4.3. Woman of Childbearing Potential .....163
10.4.4. Contraception Methods.....164
10.5. Appendix 5: Genetics.....166
10.6. Appendix 6: Liver Safety: Suggested Actions and Follow-up Assessments .....167
10.7. Appendix 7: ECG Findings of Potential Clinical Concern .....169
10.8. Appendix 8: Country-Specific Requirements .....171
10.8.1. France Contract Unique.....171
10.9. Appendix 9: RECIST 1.1 .....172
10.10. Appendix 10: Patient Global Impression of Severity and Patient Global Impression of Change.....176
10.11. Appendix 11: Abbreviations .....177
REFERENCES .....183

090177e194745c57\Approved\Approved On: 22-Jul-2020 00:48 (GMT)

## LIST OF TABLES

Table 1. Schedule of Activities (Molecular Prescreening and Screening) ............17
Table 2. Schedule of Activities (Safety Lead-in) ................................................20
Table 3. Schedule of Activities (Arm A and Arm B) ...........................................26
Table 4. Schedule of Activities (Control Arm [Arm C]: mFOLFOX6, FOLFIRI or FOLFOXIRI ± Bevacizumab)............................................31
Table 5. Schedule of Activities (Control Arm [Arm C]: CAPOX ± Bevacizumab) ...........................................................................................37
Table 6. Efficacy of Standard of Care First-Line Therapies in Patients with *BRAF*-mutant mCRC ...................................................................................43
Table 7. Study ARRAY-818-302 (EC Arm and Control Arm): Efficacy Results..........................................................................................................44
Table 8. Study ARRAY-818-302 (EC Arm and Control Arm): Adverse Events Regardless of Causality Reported in ⩾15% of EC Patients ........45
Table 9. DLT Criteria .............................................................................................68
Table 10. Treatment Regimens (Safety Lead-in)....................................................70
Table 11. Treatment Regimen (Phase 3, Arm A) ...................................................70
Table 12. Treatment Regimens (Phase 3, Arm B)...................................................71
Table 13. Treatment Regimens (Phase 3, Control Arm [Arm C])...........................72
Table 14. Recommended Encorafenib Dose Reductions.........................................81
Table 15. Recommended Encorafenib Dose Modifications ....................................81
Table 16. Recommended Cetuximab Dose Reductions...........................................84
Table 17. Recommended Dose Modifications for Cetuximab related Adverse Events ...............................................................................................84
Table 18. Recommended Dose Reductions for Oxaliplatin, Irinotecan and 5-FU as Part of mFOLFOX6 and FOLFIRI Q2W ......................................86
Table 19. Recommended Dose Reductions for Oxaliplatin, Irinotecan and 5-FU as Part of FOLFOXIRI Q2W...........................................................86
Table 20. Recommended Oxaliplatin, Irinotecan and 5-FU Dose Modifications....................................................................................86
Table 21. Recommended Capecitabine Dose Reductions ......................................91
Table 22. Recommended Capecitabine Dose Modifications...................................91
Table 23. Recommended Bevacizumab Dose Modifications..................................92
Table 24. Eastern Cooperative Oncology Group (ECOG) Performance Status Scale...........................................................................................107



090177e194745c57\Approved\Approved On: 22-Jul-2020 00:48 (GMT)

Table 25. PK Sampling Schedule for Cohort 1 (EC + FOLFIRI) in SLI ...............115
Table 26. PK Sampling Schedule for Cohort 2 (EC + mFOLFOX6) in SLI..........116
Table 27. PFS Outcome and Event Dates – Primary Analysis...............................127
Table 28. PFS Outcome and Event Dates – Supportive Analysis ..........................127
Table 29. Characteristics of DLT Probability During the DLT Evaluation Period....................................................................................................129
Table 30. Probability of Observing ≥1 Instance of Toxicity at 10% and 15% Incidence Rate ..........................................................................................129
Table 31. OS – Summary of Planned Efficacy Boundaries....................................144
Table 32. Protocol-Required Safety Laboratory Assessments ...............................153

## LIST OF FIGURES

Figure 1: *BRAF* Testing.........................................................................................119
Figure 2: Graphical Gatekeeping Multiple Testing Strategy .................................134

090177e194745c57\Approved\Approved On: 22-Jul-2020 00:48 (GMT)

# 1. PROTOCOL SUMMARY

## 1.1. Synopsis

### Rationale

The purpose of the study is to evaluate whether encorafenib plus cetuximab (EC), alone or in combination with chemotherapy, can improve clinical outcomes relative to current standard of care chemotherapy in participants with previously untreated *BRAF* V600E-mutant mCRC. Since encorafenib has not previously been combined with chemotherapy, the tolerability and PK of EC in combination with mFOLFOX6 and in combination with FOLFIRI will be evaluated in separate cohorts in the SLI portion of the trial in order to identify which chemotherapy combination is to be used in the Phase 3 portion of the study.

### Objectives, Estimands, and Endpoints

| Safety Lead-in | |
|---|---|
| **Objectives** | **Endpoints** |
| **Primary** | |
| • To determine the safety and tolerability of EC + mFOLFOX6 and EC + FOLFIRI | • Incidence of DLTs |
| **Secondary** | |
| • To assess the overall safety and tolerability of EC + mFOLFOX6 and EC + FOLFIRI | • Incidence and severity of AEs graded according to the NCI CTCAE v4.03 and changes in clinical laboratory parameters, vital signs and ECGs<br>• Incidence of dose interruptions, dose modifications and discontinuations due to AEs |
| • To estimate the efficacy of EC + mFOLFOX6 and EC + FOLFIRI | • ORR by Investigator, defined as the proportion of participants who have achieved a confirmed BOR (CR or PR) per RECIST v1.1<br>• DOR by Investigator, defined as the time from the date of first radiographic evidence of response (CR or PR) to the earliest documented disease progression per RECIST v1.1, or death due to any cause<br>• PFS by Investigator, defined as the time from first dose to the earliest documented disease progression per RECIST v1.1, or death due to any cause<br>• TTR by Investigator, defined as the time from first dose to first radiographic evidence of response (CR or PR) per RECIST v1.1 |
| • To compare the efficacy of EC + mFOLFOX6 and EC + FOLFIRI | • OS defined as the time from first dose to death due to any cause |
| • To characterize the PK of encorafenib, irinotecan, oxaliplatin and relevant metabolites | • PK parameters of encorafenib, irinotecan, oxaliplatin and relevant metabolites |

090177e194745c57\Approved\Approved On: 22-Jul-2020 00:48 (GMT)

| | |
|---|---|
| • To assess drug-drug interaction of encorafenib with irinotecan or oxaliplatin | • Changes in exposures of irinotecan and its metabolite (SN-38) on Cycle 1 Day 15 compared to Cycle 1 Day 1 in Cohort 1 (EC + FOLFIRI)<br>• Changes in exposures of oxaliplatin on Cycle 1 Day 15 compared to Cycle 1 Day 1 in Cohort 2 (EC + mFOLFOX6) |
| **Tertiary/Exploratory** | |
| • To understand the relationship between the therapeutic intervention(s) being studied and the biology of the participant's disease | • Measurements of biomarkers, consisting of DNA, RNA, protein or defined cell types, resulting from analyses of peripheral blood and/or tumor tissue biospecimen obtained at baseline, on treatment and/or at end-of-study |
| **Randomized Phase 3** | |
| **Objectives** | **Endpoints** |
| **Primary** | |
| • To compare the efficacy of EC (Arm A) vs SOC (Control Arm [Arm C]) as measured by PFS<br>• To compare the efficacy EC + mFOLFOX6 or EC + FOLFIRI (Arm B) vs the Control Arm as measured by PFS | • PFS by BICR, defined as the time from the date of randomization to the earliest documented disease progression per RECIST v1.1, or death due to any cause |
| **Key Secondary** | |
| • To compare the efficacy of Arm A vs the Control Arm as measured by OS<br>• To compare the efficacy of Arm B vs the Control Arm as measured by OS | • OS, defined as the time from the date of randomization to death due to any cause |
| **Secondary** | |
| • To compare the efficacy of Arm A vs the Control Arm as measured by ORR, DOR, PFS, PFS2 and TTR<br>• To compare the efficacy of Arm B vs the Control Arm as measured by ORR, DOR, PFS, PFS2 and TTR<br>• To compare the efficacy of Arm A vs Arm B as measured by OS, PFS, PFS2, ORR, DOR and TTR | • ORR by BICR and by Investigator<br>• DOR by BICR and by Investigator<br>• PFS by BICR<br>• OS<br>• PFS by Investigator<br>• TTR (by BICR and by Investigator), defined as the time from the date of randomization to first radiographic evidence of response (CR or PR) per RECIST v1.1<br>• PFS2, defined as the time from the date of randomization to the second objective disease progression per RECIST v1.1, or death from any cause, whichever occurs first |
| • To determine the safety and tolerability of EC<br>• To determine the safety and tolerability of EC + mFOLFOX6 or EC + FOLFIRI | • Incidence and severity of AEs graded according to the NCI CTCAE v4.03 and changes in clinical laboratory parameters, vital signs, and ECGs |
| • To compare the PRO measures | • PROs as measured by the EORTC QLQ-C30, EQ-5D-5L, PGIS and PGIC. |



090177e194745c57\Approved\Approved On: 22-Jul-2020 00:48 (GMT)

| | |
|---|---|
| • To evaluate trough concentrations of encorafenib and its metabolite LHY746 in Arm A and Arm B | • Trough plasma concentrations of encorafenib and the metabolite LHY746 in Arm A and Arm B |
| • To characterize the PK of encorafenib and its metabolite LHY746 in participants randomized in China (Arm A) | • PK parameters of encorafenib and its metabolite LHY746 |
| • To confirm the MSI status in tumor tissue | • Summarize MSI-status as determined by retrospective central testing of baseline tumor tissue |
| • To determine the correlation between cfDNA genetic alterations and clinical outcome | • *BRAF* V600E variant allele fraction (VAF) and/or overall mean VAF from cfDNA analysis of plasma samples collected at baseline and on treatment |
| **Tertiary/Exploratory** | |
| • To understand the relationship between the therapeutic intervention(s) being studied and the biology of the participant's disease | • Measurements of biomarkers, consisting of DNA, RNA, protein or defined cell types, resulting from analyses of peripheral blood and/or tumor tissue biospecimen obtained at baseline, on treatment and/or at end-of-study |

**Estimands**

The primary estimand in the SLI is the DLT rate estimated based on data from DLT-evaluable participants during the DLT-evaluation period, which is the first 28 days after the first dose of study intervention in the SLI. The number and proportion of participants experiencing DLTs during the DLT-evaluation period will be summarized for Cohort 1 (EC + FOLFIRI) and Cohort 2 (EC +mFOLFOX6), separately.

The primary estimand in the Phase 3 portion of the study is the treatment effect in PFS by BICR, as measured by the hazard ratio. PFS is defined as the time from the date of randomization to the earliest documented disease progression per RECIST v1.1, or death due to any cause. The hypothetical strategy will be applied for the intercurrent events. PFS will be compared between Arm A and the Control Arm (Arm C), and between Arm B and the Control Arm using a 1-sided stratified log-rank test.

A complete list of estimands are defined in Section 9.1.1.

**Overall Design**

This is an open-label, multicenter, randomized Phase 3 study of EC with or without chemotherapy versus standard of care chemotherapy in participants with previously untreated *BRAF* V600E-mutant mCRC. Prior to the Phase 3 portion, a SLI will be conducted at a limited number of sites to evaluate the safety/tolerability and PK of EC in combination with either mFOLFOX6 or FOLFIRI. The primary objectives in the Phase 3 portion of the study are to compare the efficacy, as measured by the primary endpoint of PFS by BICR, of Arm A versus the Control Arm and of Arm B versus the Control Arm. The study will be considered positive (ie, demonstrated evidence of effectiveness) if at least one of the hypothesis tests of the primary endpoint is statistically significant.



090177e194745c57\Approved\Approved On: 22-Jul-2020 00:48 (GMT)

**Number of Participants**

Approximately 60 participants will be enrolled in the SLI portion (up to 30 per regimen). The results of the SLI will inform which chemotherapy regimen is used in Arm B of the Phase 3 portion of the study. In the Phase 3 portion, approximately 870 participants will be randomized 1:1:1 to receive EC (Arm A), EC + chemotherapy (Arm B) or SOC chemotherapy (Control Arm), with approximately 290 participants per arm. The determination of sample sizes is detailed in Section 9.2.

**Intervention Groups and Duration**

Participants in the SLI will receive either encorafenib (300 mg QD) + cetuximab (500 mg/$m^2$ Q2W) + mFOLFOX6 or encorafenib (300 mg QD) + cetuximab (500 mg/$m^2$ Q2W) + FOLFIRI. Participants randomized to Arm A of Phase 3 will receive encorafenib (300 mg QD) + cetuximab (500 mg/$m^2$ Q2W). Based on the results of the SLI, participants randomized to Arm B of Phase 3 will receive either encorafenib (300 mg QD) + cetuximab (500 mg/$m^2$ Q2W) + mFOLFOX6 or encorafenib (300 mg QD) + cetuximab (500 mg/$m^2$ Q2W) + FOLFIRI. If neither SLI regimen is tolerated, Phase 3 will proceed without Arm B.

The regimens to be used in the Control Arm will depend on which regimen is used in Arm B. If mFOLFOX6 is used in Arm B, the Control Arm will consist of Investigator's choice of mFOLFOX6 ± bevacizumab, FOLFOXIRI ± bevacizumab or CAPOX ± bevacizumab. If FOLFIRI is used in Arm B, the Control Arm will consist of Investigator's choice of FOLFIRI ± bevacizumab or FOLFOXIRI ± bevacizumab. If Phase 3 proceeds without Arm B, the Control Arm will consist of Investigator's choice of mFOLFOX6 ± bevacizumab, FOLFIRI ± bevacizumab, FOLFOXIRI ± bevacizumab or CAPOX ± bevacizumab.

Participants will receive study intervention until disease progression (confirmed by BICR in Phase 3 portion), withdrawal of consent/assent, lost to follow-up, death or unacceptable toxicity. After discontinuation of study intervention, participants will be followed for survival, until withdrawal of consent/assent, the participant is lost to follow-up, death or defined End of Study.

**Data Monitoring Committee or Other Independent Oversight Committee**

This study will use an E-DMC to review cumulative safety data during the study conduct as well as to review the interim futility analysis on the PFS data. The E-DMC is independent of the study team and includes only external members.

All imaging data acquired in the Phase 3 portion of the study for efficacy purposes (eg, CT/MRI scans) will be transmitted to an imaging vendor for BICR review. Imaging data acquired in the SLI portion of the study may also be transmitted to an imaging vendor for BICR review. These images will be read by readers who are blinded to treatment assignment and to other clinical data as specified in the BICR Charter.

090177e194745c57\Approved\Approved On: 22-Jul-2020 00:48 (GMT)

090177e194745c57\Approved\Approved On: 22-Jul-2020 00:48 (GMT)

## 1.2. Schema

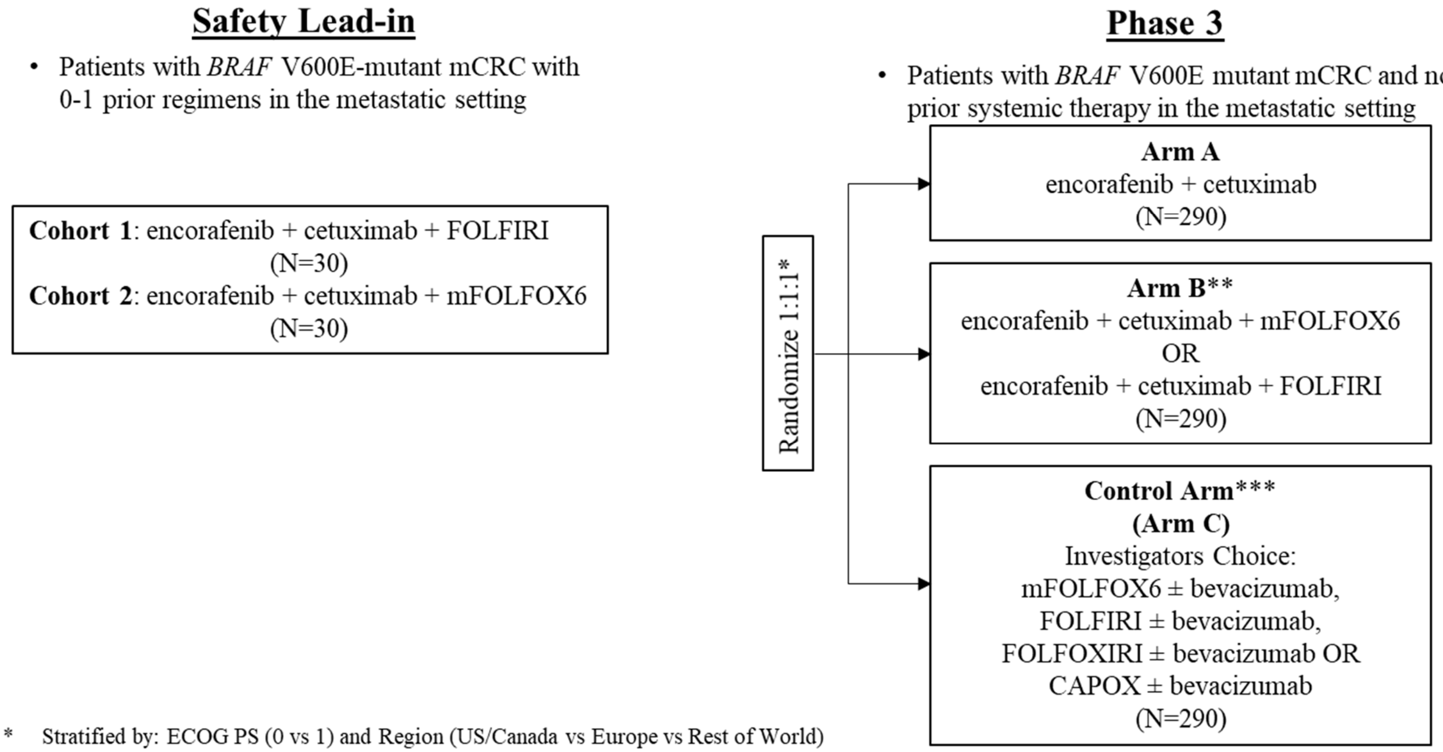


* Stratified by: ECOG PS (0 vs 1) and Region (US/Canada vs Europe vs Rest of World)

** Use of mFOLFOX6 or FOLFIRI in Arm B will depend on results of the SLI portion of the study. If neither SLI regimen is tolerated, Phase 3 will proceed without Arm B.

*** If mFOLFOX6 is used in Arm B, the Control Arm will consist of investigator's choice of mFOLFOX6 ± bevacizumab, FOLFOXIRI ± bevacizumab or CAPOX ± bevacizumab.
If FOLFIRI is used in Arm B, the Control Arm will consist of investigator's choice of FOLFIRI ± bevacizumab or FOLFOXIRI ± bevacizumab.
If Phase 3 proceeds without Arm B, the Control Arm will consist of investigator's choice of mFOLFOX6 ± bevacizumab, FOLFIRI ± bevacizumab, FOLFOXIRI ± bevacizumab or CAPOX ± bevacizumab.

090177e194745c57\Approved\Approved On: 22-Jul-2020 00:48 (GMT)

## 1.3. Schedule of Activities

The SoA table provides an overview of the protocol visits and procedures. Refer to the STUDY ASSESSMENTS AND PROCEDURES section of the protocol for detailed information on each procedure and assessment required for compliance with the protocol.

The investigator may schedule visits (unplanned visits) in addition to those listed in the SoA table, in order to conduct evaluations or assessments required to protect the well-being of the participant.

**Table 1. Schedule of Activities (Molecular Prescreening and Screening)**

| **Evaluation/Window**<br>Abbreviations used in this table may be found in Appendix 11. | **Prescreening**<br>**Any Time Before Screening** | **Screening**<br>**Day -28 to -1** | **Randomization** | **Notes/Protocol Section** |
|---|---|---|---|---|
| **Administrative Procedures** | | | | |
| Informed consent/assent | X | | | See Section 10.1.3 |
| Molecular Prescreening inclusion/exclusion criteria | X | | | See Section 5.1.1 and Section 5.2.1 |
| Contact IRT | X | | X | All participants will be registered for the study using the IRT after informed consent/assent is obtained. Phase 3 participants will be randomized via IRT upon completion of Screening.<br>Phase 3 participants must receive their first dose of study intervention within 5 days of randomization.<br>See Section 6.3.1 |
| Demography | X | | | See Section 8.2.1 |
| Screening inclusion/exclusion criteria | | X | | See Section 5.1.2 and Section 5.2.2 |
| Medical history | | X | | See Section 8.2.1 |
| Prior antineoplastic therapy/concomitant medication | | X | | See Section 6.5 |
| ECOG PS | | X | | See Section 8.2.10 |

090177e194745c57\Approved\Approved On: 22-Jul-2020 00:48 (GMT)

**Table 1. Schedule of Activities (Molecular Prescreening and Screening)**

| **Evaluation/Window** Abbreviations used in this table may be found in Appendix 11. | **Prescreening** **Any Time Before Screening** | **Screening** **Day -28 to -1** | **Randomization** | **Notes/Protocol Section** |
|---|---|---|---|---|
| Verification of eligibility criteria | | X | | See Section 5 |
| **Clinical Procedures/Assessments** | | | | |
| Tumor tissue sample for *BRAF* V600E mutation testing | X | | | Central laboratory test for *BRAF* V600E mutation and other molecular markers. See Section 8.7.2 and Section 8.8.2 |
| Tumor radiographic assessment (CT, MRI) | | X | | Must be performed prior to enrollment for participants in the SLI and prior to randomization for participants in the Phase 3 portion. See Section 8.1.1 |
| Physical exam | | X | | See Section 8.2.3 |
| Height/Weight | | X | | See Section 8.2.3 |
| Vital signs | | X | | See Section 8.2.6 |
| Triplicate 12-lead ECG | | X | | See Section 8.2.7 |
| Adverse event assessment | | X | | See Section 8.3 and Appendix 3 |
| Dermatologic exam | | X | | See Section 8.2.5 |
| **Laboratory Assessments** | | | | |
| Hematology | | X | | See Section 8.2.8 and Table 32 |
| Chemistry | | X | | See Section 8.2.8 and Table 32 |
| Coagulation | | X | | See Section 8.2.8 and Table 32 |
| Full Urinalysis | | X | | See Section 8.2.8 and Table 32 |
| HBV and HCV serology; HIV where mandated | | X | | See Section 5.2.2 and Table 32 |
| Serum pregnancy test | | X | | See Section 8.2.9 and Table 32 |

090177e194745c57\Approved\Approved On: 22-Jul-2020 00:48 (GMT)

**Table 1. Schedule of Activities (Molecular Prescreening and Screening)**

| **Evaluation/Window** Abbreviations used in this table may be found in Appendix 11. | **Prescreening** | **Screening** | **Randomization** | **Notes/Protocol Section** |
|---|---|---|---|---|
| | **Any Time Before Screening** | **Day -28 to -1** | | |
| FSH | | X | | See Section 10.4.3 and Table 32 |
| Blood sample for CRP | | X | | See Section 8.8.1 |
| Blood sample for CEA | | X | | See Section 8.8.1 |
| Blood sample for *BRAF* CDx | | X | | Collect 20 mL of blood optimized for plasma preparation.<br>See Section 8.7.6 |

090177e194745c57\Approved\Approved On: 22-Jul-2020 00:48 (GMT)

## Table 2. Schedule of Activities (Safety Lead-in)

| **Evaluation** Abbreviations used in this table may be found in Appendix 11. | **Treatment Period (Each 28 Day Cycle)** | | | | **Post-Treatment Period** | | | **Notes/Protocol Section** |
|---|---|---|---|---|---|---|---|---|
| | **Day 1** | **Day 3** | **Day 15** | **Day 17** | **End-of-Treatment Visit** | **Follow-up Visit** | **Long Term Follow-up** | |
| **Window** | **± 3D** | **± 3D** | **± 3D** | **± 3D** | **± 3D** | **28 Days ± 7D** | **Q12W ± 7D** | |
| **Administrative Procedures** | | | | | | | | |
| Verify Screening inclusion/exclusion criteria | Cycle 1 | | | | | | | Must occur prior to first dose.<br>See Section 5.1.2 and Section 5.2.2 |
| Verify demography; medical history from Screening to Day 1 | Cycle 1 | | | | | | | See Section 8.2.1 and Section 8.2.2 |
| Concomitant medication/therapies | Assess continuously | | | | | | | See Section 6.5 |
| ECOG PS | Cycle 1 | | | | | | | Does not need to be repeated if performed within 72 hours prior to Cycle 1 Day 1 (ie, first day of dosing).<br>See Section 8.2.10 |
| Document survival status | | | | | | X | X | After discontinuation of study intervention, participants will be followed approximately every 3 months until withdrawal of consent, the participant is lost to follow-up, death or defined End of Study.<br>See Section 7.1 |
| Document subsequent therapies and dates of progression | | | | | Assess continuously | | | See Section 7.1 |

090177e194745c57\Approved\Approved On: 22-Jul-2020 00:48 (GMT)

**Table 2. Schedule of Activities (Safety Lead-in)**

| **Evaluation** Abbreviations used in this table may be found in Appendix 11. | **Treatment Period (Each 28 Day Cycle)** | | | | **Post-Treatment Period** | | | **Notes/Protocol Section** |
|---|---|---|---|---|---|---|---|---|
| | **Day 1** | **Day 3** | **Day 15** | **Day 17** | **End-of-Treatment Visit** | **Follow-up Visit** | **Long Term Follow-up** | |
| **Window** | **± 3D** | **± 3D** | **± 3D** | **± 3D** | **± 3D** | **28 Days ± 7D** | **Q12W ± 7D** | |
| Dispense encorafenib (with diary) | X | | | | | | | Encorafenib dosing will not begin until Cycle 1 Day 3 for participants receiving FOLFIRI<br>See Section 6.1.3.1 |
| Cetuximab IV infusion | X | | X | | | | | See Section 6.1.3.2 |
| mFOLFOX6 or FOLFIRI | X | | X | | | | | See Section 6.1.3.2 |
| Assess study intervention compliance | X | | | | X | | | See Section 6.4 |
| **Clinical Procedures/Assessments** | | | | | | | | |
| Physical exam | X | | | | X | X | | Does not need to be repeated if performed within 72 hours prior to Cycle 1 Day 1 (ie, first day of dosing).<br>See Section 8.2.3 |
| Weight | X | | | | | | | Does not need to be repeated if performed within 72 hours prior to Cycle 1 Day 1 (ie, first day of dosing).<br>See Section 8.2.3 |
| BSA | X | | | | | | | Does not need to be repeated if performed within 72 hours prior to Cycle 1 Day 1 (ie, first day of dosing).<br>See Section 8.2.4 |

090177e194745c57\Approved\Approved On: 22-Jul-2020 00:48 (GMT)

## Table 2. Schedule of Activities (Safety Lead-in)

| **Evaluation** Abbreviations used in this table may be found in Appendix 11. | **Treatment Period (Each 28 Day Cycle)** | | | | **Post-Treatment Period** | | | **Notes/Protocol Section** |
|---|---|---|---|---|---|---|---|---|
| | **Day 1** | **Day 3** | **Day 15** | **Day 17** | **End-of-Treatment Visit** | **Follow-up Visit** | **Long Term Follow-up** | |
| **Window** | **± 3D** | **± 3D** | **± 3D** | **± 3D** | **± 3D** | **28 Days ± 7D** | **Q12W ± 7D** | |
| Vital signs | X | | X | | X | X | | See Section 8.2.6 |
| Triplicate 12-lead ECG | Cycle 1 | | | | | | | Predose only.<br>See Section 8.2.7 |
| Single 12-lead ECG | Cycles 2+ | | | | X | X | | Predose only. Perform continuous ECGs if QTc abnormality.<br>See Section 8.2.7 |
| Tumor radiographic assessment (CT, MRI) | Q6W for the first 18 months after the date of first dose then Q8W thereafter or until BICR-confirmed PD, withdrawal of consent/assent, initiation of subsequent anticancer therapy, participant is lost to follow-up, death or defined End of Study. | | | | | | | The first post-baseline scan must be performed 42 to 48 days from the date of first dose.<br>See Section 8.1.1 |
| Dermatologic exam | X (Cycles 1, 3, 5…) | | | | X | X | X[a] | Performed every 8 weeks from Cycle 1 Day 1 (ie, on Day 1 of Cycles 3, 5, 7…).<br>[a] Following the 28-day follow-up, it is recommended participants be monitored with dermatological examinations for cutaneous secondary malignancies approximately every 2 months for up to 6 months after the last encorafenib dose or until initiation of another antineoplastic therapy. Clinically significant findings that are considered related to study intervention should be reported as SAEs.<br>See Section 8.2.5 |

090177e194745c57\Approved\Approved On: 22-Jul-2020 00:48 (GMT)

## Table 2. Schedule of Activities (Safety Lead-in)

| **Evaluation**<br>Abbreviations used in this table may be found in Appendix 11. | **Treatment Period (Each 28 Day Cycle)** | | | | **Post-Treatment Period** | | | **Notes/Protocol Section** |
|---|---|---|---|---|---|---|---|---|
| | **Day 1** | **Day 3** | **Day 15** | **Day 17** | **End-of-Treatment Visit** | **Follow-up Visit** | **Long Term Follow-up** | |
| **Window** | **± 3D** | **± 3D** | **± 3D** | **± 3D** | **± 3D** | **28 Days ± 7D** | **Q12W ± 7D** | |
| Adverse event assessment | Assess continuously | | | | | | | See Section 8.3 and Appendix 3 |
| **Laboratory Assessments** | | | | | | | | |
| Hematology | X | | X | | X | X | | Does not need to be repeated if performed within 72 hours prior to Cycle 1 Day 1 (ie, first day of dosing).<br>See Section 8.2.8 and Table 32 |
| Chemistry | X | | X | | X | X | | Does not need to be repeated if performed within 72 hours prior to Cycle 1 Day 1 (ie, first day of dosing).<br>See Section 8.2.8 and Table 32 |
| Coagulation | X | | X | | X | X | | Does not need to be repeated if performed within 72 hours prior to Cycle 1 Day 1 (ie, first day of dosing).<br>See Section 8.2.8 and Table 32 |
| Urinalysis | X | | | | X | X | | Does not need to be repeated if performed within 72 hours prior to Cycle 1 Day 1 (ie, first day of dosing).<br>Full urinalysis on Cycle 1 Day 1, only urine protein thereafter.<br>See Section 8.2.8 and Table 32 |

090177e194745c57\Approved\Approved On: 22-Jul-2020 00:48 (GMT)

**Table 2. Schedule of Activities (Safety Lead-in)**

| **Evaluation**<br>Abbreviations used in this table may be found in Appendix 11. | **Treatment Period (Each 28 Day Cycle)** | | | | **Post-Treatment Period** | | | **Notes/Protocol Section** |
|---|---|---|---|---|---|---|---|---|
| | **Day 1** | **Day 3** | **Day 15** | **Day 17** | **End-of-Treatment Visit** | **Follow-up Visit** | **Long Term Follow-up** | |
| **Window** | **± 3D** | **± 3D** | **± 3D** | **± 3D** | **± 3D** | **28 Days ± 7D** | **Q12W ± 7D** | |
| Urine pregnancy test | X | | | | X | | | Does not need to be repeated if performed within 72 hours prior to Cycle 1 Day 1 (ie, first day of dosing).<br>See Section 8.2.9 and Table 32 |
| Blood sample for PK | Cycles 1-6 | Cycle 1 | Cycle 1 | Cycle 1 | | | | Days 1 and 15 of Cycle 1 of Cohort 1 (EC + FOLFIRI): predose, 0.75, 1.5, 2.5, 3.5, 5.5 and 7.5 h post-dosing<br>Days 1 and 15 of Cycle 1 of Cohort 2 (EC + mFOLFOX6): predose, 1, 2, 3, 4, 6 and 8 h post-dosing<br>All other timepoints: Predose only<br>See Section 8.5 |
| Blood sample for CRP | X | | | | X | | | See Section 8.8.1 |
| Blood sample for CEA | X | | | | X | | | See Section 8.8.1 |
| Blood sample for cfDNA | Cycles 1,7 | | Cycles 1, 2 | | X | | | Collect 20 mL of blood optimized for plasma preparation at indicated visits (all pre-dose).<br>See Section 8.8.3 |
| Blood sample for protein biomarkers | Cycles 1, 2 | | Cycles 1, 2 | | X | | | Collect 10 mL of whole blood for processing into serum at indicated visits (all pre-dose).<br>See Section 8.8.5 |

090177e194745c57\Approved\Approved On: 22-Jul-2020 00:48 (GMT)

**Table 2. Schedule of Activities (Safety Lead-in)**

| **Evaluation** Abbreviations used in this table may be found in Appendix 11. | **Treatment Period (Each 28 Day Cycle)** | | | | **Post-Treatment Period** | | | **Notes/Protocol Section** |
|---|---|---|---|---|---|---|---|---|
| | **Day 1** | **Day 3** | **Day 15** | **Day 17** | **End-of-Treatment Visit** | **Follow-up Visit** | **Long Term Follow-up** | |
| **Window** | **± 3D** | **± 3D** | **± 3D** | **± 3D** | **± 3D** | **28 Days ± 7D** | **Q12W ± 7D** | |
| Blood sample for germline comparator | X | | | | | | | Collect a 4 mL blood biospecimen prior to first dose of study intervention.<br>See Section 8.8.2 |
| Blood sample for banked biospecimen | X | | | | | | | Unless prohibited by local regulations or ethics committee decision, collect a blood sample (4 mL) optimized for genomic analysis before the first dose of study intervention.<br>If not collected on the designated collection day, collect at the next available time point when biospecimens are being collected in conjunction with a participant visit.<br>See Section 8.7.7 |
| FFPE Tumor Tissue | | | | | X<br>(optional) | | | See Section 8.8.2 and Section 8.8.4 |

090177e194745c57\Approved\Approved On: 22-Jul-2020 00:48 (GMT)

**Table 3. Schedule of Activities (Arm A and Arm B)**

| **Evaluation** Abbreviations used in this table may be found in Appendix 11. | **Treatment Period (Each 28 Day Cycle)** | | **Post-Treatment Period** | | | **Notes/Protocol Section** |
|---|---|---|---|---|---|---|
| | **Day 1** | **Day 15** | **End-of-Treatment Visit** | **Follow-up Visit** | **Long Term Follow-up** | |
| **Window** | **± 3D** | **± 3D** | **± 3D** | **28 Days ± 7D** | **Q12W± 7D** | |
| **Administrative Procedures** | | | | | | |
| Verify Screening inclusion/exclusion criteria | Cycle 1 | | | | | Must occur prior to first dose. See Section 5.1.2 and Section 5.2.2 |
| Verify demography; medical history from Screening to Day 1 | Cycle 1 | | | | | See Section 8.2.1 and Section 8.2.2 |
| Concomitant medication/therapies | Assess continuously | | | | | See Section 6.5 |
| EORTC QLQ-C30, PGIS, PGIC and EQ-5D-5L | Q6W for the first 18 months after the date of randomization then Q8W thereafter or until End of Treatment | | X | X | | While on treatment, PRO assessments should be done with the same frequency as the tumor radiographic visits. PGIC will not be performed at baseline. See Section 8.1.2 |
| ECOG PS | Cycle 1 | | | | | Does not need to be repeated if performed within 72 hours prior to Cycle 1 Day 1 (ie, first day of dosing). See Section 8.2.10 |
| Document survival status | | | | X | X | After discontinuation of study intervention, participants will be followed approximately every 3 months, until withdrawal of consent/assent, the participant is lost to follow-up, death or defined End of Study See Section 7.1 |

090177e194745c57\Approved\Approved On: 22-Jul-2020 00:48 (GMT)

**Table 3. Schedule of Activities (Arm A and Arm B)**

| **Evaluation** Abbreviations used in this table may be found in Appendix 11. | **Treatment Period (Each 28 Day Cycle)** | | **Post-Treatment Period** | | | **Notes/Protocol Section** |
|---|---|---|---|---|---|---|
| | **Day 1** | **Day 15** | **End-of-Treatment Visit** | **Follow-up Visit** | **Long Term Follow-up** | |
| **Window** | **± 3D** | **± 3D** | **± 3D** | **28 Days ± 7D** | **Q12W± 7D** | |
| Document subsequent therapies and dates of progression | | | Assess continuously | | | See Section 7.1 |
| Dispense encorafenib (with diary) | X | | | | | If the decision is made to include FOLFIRI in Arm B, encorafenib dosing will not begin until Cycle 1 Day 3 for participants receiving FOLFIRI unless results from the SLI suggest a more optimal timing.<br>See Section 6.1.3.1 |
| Cetuximab IV infusion | X | X | | | | See Section 6.1.3.2 |
| For Arm B Only: mFOLFOX6 OR FOLFIRI | X | X | | | | See Section 6.1.3.2 |
| Assess study intervention compliance | X | | X | | | See Section 6.4 |
| **Clinical Procedures/Assessments** | | | | | | |
| Physical exam | X | | X | X | | Does not need to be repeated if performed within 72 hours prior to Cycle 1 Day 1 (ie, first day of dosing).<br>See Section 8.2.3 |
| Weight | X | | | | | Does not need to be repeated if performed within 72 hours prior to Cycle 1 Day 1 (ie, first day of dosing).<br>See Section 8.2.3 |
| Height | X | | | | | Height to be measured in participants <18 years old.<br>See Section 8.2.3 |

090177e194745c57\Approved\Approved On: 22-Jul-2020 00:48 (GMT)

**Table 3. Schedule of Activities (Arm A and Arm B)**

| Evaluation<br>Abbreviations used in this table may be found in Appendix 11. | Treatment Period (Each 28 Day Cycle) | | Post-Treatment Period | | | Notes/Protocol Section |
|---|---|---|---|---|---|---|
| | Day 1 | Day 15 | End-of-Treatment Visit | Follow-up Visit | Long Term Follow-up | |
| **Window** | **± 3D** | **± 3D** | **± 3D** | **28 Days ± 7D** | **Q12W± 7D** | |
| BSA | X | | | | | Does not need to be repeated if performed within 72 hours prior to Cycle 1 Day 1 (ie, first day of dosing).<br>See Section 8.2.4 |
| Vital signs | X | X | X | X | | See Section 8.2.6 |
| Triplicate 12-lead ECG | Cycle 1 | | | | | Predose only.<br>See Section 8.2.7 |
| Single 12-lead ECG | Cycles 2+ | | X | X | | Predose only. Perform continuous ECGs if QTc abnormality.<br>See Section 8.2.7 |
| Tumor radiographic assessment (CT, MRI) | Q6W for the first 18 months after the date of randomization then Q8W thereafter or until BICR-confirmed PD, withdrawal of consent/assent, initiation of subsequent anticancer therapy, participant is lost to follow-up, death or defined End of Study. | | | | | The first post-baseline scan must be performed 42 to 48 days from the date of randomization.<br>See Section 8.1.1 |
| Dermatologic exam | X<br>(Cycles 1, 3, 5…) | | X | X | | Performed every 8 weeks from Cycle 1 Day 1 (ie, on Day 1 of Cycles 3, 5, 7…).<br>Following the 28-day follow up, it is recommended participants be monitored with dermatological examinations for cutaneous secondary malignancies approximately every 2 months for up to 6 months after the last encorafenib dose or until initiation of another antineoplastic therapy. Clinically significant findings that are considered related to study intervention should be reported as SAEs.<br>See Section 8.2.5 |

090177e194745c57\Approved\Approved On: 22-Jul-2020 00:48 (GMT)

**Table 3. Schedule of Activities (Arm A and Arm B)**

| **Evaluation** Abbreviations used in this table may be found in Appendix 11. | **Treatment Period (Each 28 Day Cycle)** | | **Post-Treatment Period** | | | **Notes/Protocol Section** |
|---|---|---|---|---|---|---|
| | **Day 1** | **Day 15** | **End-of-Treatment Visit** | **Follow-up Visit** | **Long Term Follow-up** | |
| **Window** | **± 3D** | **± 3D** | **± 3D** | **28 Days ± 7D** | **Q12W± 7D** | |
| Adverse event assessment | Assess continuously | | | | | See Section 8.3 and Appendix 3 |
| **Laboratory Assessments** | | | | | | |
| Hematology | X | X | X | X | | Does not need to be repeated if performed within 72 hours prior to Cycle 1 Day 1 (ie, first day of dosing). See Section 8.2.8 and Table 32 |
| Chemistry | X | X | X | X | | Does not need to be repeated if performed within 72 hours prior to Cycle 1 Day 1 (ie, first day of dosing). See Section 8.2.8 and Table 32 |
| Coagulation | X | X | X | X | | Does not need to be repeated if performed within 72 hours prior to Cycle 1 Day 1 (ie, first day of dosing). See Section 8.2.8 and Table 32 |
| Urinalysis | X | | X | X | | Does not need to be repeated if performed within 72 hours prior to Cycle 1 Day 1 (ie, first day of dosing). Full urinalysis on Cycle 1 Day 1, only urine protein thereafter. See Section 8.2.8 and Table 32 |
| Urine pregnancy test | X | | X | | | Does not need to be repeated if performed within 72 hours prior to Cycle 1 Day 1 (ie, first day of dosing). See Section 8.2.9 and Table 32 |
| Blood sample for PK | Cycles 1-6 | | | | | Day 1: Predose See Section 8.5 For participants in China, see Section 8.5.1 |
| Blood sample for CRP | X | | X | | | See Section 8.8.1 |

090177e194745c57\Approved\Approved On: 22-Jul-2020 00:48 (GMT)

**Table 3. Schedule of Activities (Arm A and Arm B)**

| **Evaluation** Abbreviations used in this table may be found in Appendix 11. | **Treatment Period (Each 28 Day Cycle)** | | **Post-Treatment Period** | | | **Notes/Protocol Section** |
|---|---|---|---|---|---|---|
| | **Day 1** | **Day 15** | **End-of-Treatment Visit** | **Follow-up Visit** | **Long Term Follow-up** | |
| **Window** | **± 3D** | **± 3D** | **± 3D** | **28 Days ± 7D** | **Q12W± 7D** | |
| Blood sample for CEA | X | | X | | | See Section 8.8.1 |
| Blood sample for cfDNA | Cycles 1,7 | Cycles 1, 2 | X | | | Collect 20 mL of blood optimized for plasma preparation at indicated visits (all pre-dose). See Section 8.8.3 |
| Blood sample for protein biomarkers | Cycles 1, 2 | Cycles 1, 2 | X | | | Collect 10 mL of whole blood for processing into serum at indicated visits (all pre-dose). See Section 8.8.5 |
| Blood sample for germline comparator | X | | | | | Collect a 4 mL blood biospecimen prior to first dose of study intervention. See Section 8.8.2 |
| Blood sample for banked biospecimen | X | | | | | Unless prohibited by local regulations or ethics committee decision, collect a blood sample (4 mL) optimized for genomic analysis before the first dose of study intervention. If not collected on the designated collection day, collect at the next available time point when biospecimens are being collected in conjunction with a participant visit. See Section 8.7.7 |
| FFPE Tumor Tissue | | | X (optional) | | | See Section 8.8.2 and Section 8.8.4 |

**Table 4. Schedule of Activities (Control Arm [Arm C]: mFOLFOX6, FOLFIRI or FOLFOXIRI ± Bevacizumab)**

| **Evaluation** Abbreviations used in this table may be found in Appendix 11. | **Treatment Period (Each 28 Day Cycle)** | | **Post-Treatment Period** | | | **Notes/Protocol Section** |
|---|---|---|---|---|---|---|
| | **Day 1** | **Day 15** | **End-of-Treatment Visit** | **Follow-up Visit** | **Long Term Follow-up** | |
| **Window** | **± 3D** | **± 3D** | **± 3D** | **28 Days ± 7D** | **Q12W ± 7D** | |
| **Administrative Procedures** | | | | | | |
| Verify inclusion/exclusion criteria | Cycle 1 | | | | | Must occur prior to first dose.<br>See Section 5.1.2 and Section 5.2.2 |
| Verify demography; medical history from Screening to Day 1 | Cycle 1 | | | | | See Section 8.2.1 and Section 8.2.2 |
| Concomitant medication/ therapies | Assess continuously | | | | | See Section 6.5 |
| EORTC QLQ-C30, PGIS, PGIC and EQ-5D-5L | Q6W for the first 18 months after the date of randomization then Q8W thereafter or until End of Treatment | | X | X | | While on treatment, PRO assessments should be done with the same frequency as the tumor radiographic visits.<br>PGIC will not be performed at baseline.<br>See Section 8.1.2 |
| ECOG PS | Cycle 1 | | | | | Does not need to be repeated if performed within 72 hours prior to Cycle 1 Day 1 (ie, first day of dosing).<br>See Section 8.2.10 |



090177e194745c57\Approved\Approved On: 22-Jul-2020 00:48 (GMT)

090177e194745c57\Approved\Approved On: 22-Jul-2020 00:48 (GMT)

**Table 4. Schedule of Activities (Control Arm [Arm C]: mFOLFOX6, FOLFIRI or FOLFOXIRI ± Bevacizumab)**

| **Evaluation** Abbreviations used in this table may be found in Appendix 11. | **Treatment Period (Each 28 Day Cycle)** | | **Post-Treatment Period** | | | **Notes/Protocol Section** |
|---|---|---|---|---|---|---|
| | **Day 1** | **Day 15** | **End-of-Treatment Visit** | **Follow-up Visit** | **Long Term Follow-up** | |
| **Window** | **± 3D** | **± 3D** | **± 3D** | **28 Days ± 7D** | **Q12W ± 7D** | |
| Document survival status | | | | X | X | After discontinuation of study intervention, participants will be followed approximately every 3 months until withdrawal of consent/assent, the participant is lost to follow-up, death or defined End of Study<br>See Section 7.1 |
| Document subsequent therapies and dates of progression | | | Assess continuously | | | See Section 7.1 |
| mFOLFOX6, FOLFIRI, FOLFOXIRI administration | X | X | | | | Choice of treatment regimen is at the participant/treating investigator's discretion but must be declared prior to randomization and must not be changed during the course of the participant's treatment.<br>See Section 6.1.3.2 |
| Bevacizumab administration (optional) | X | | | | | If the investigator decides to administer bevacizumab (Zirabev™ or Avastin®), Zirabev™ must be administered where approved. Where Zirabev™ is not approved, Avastin® may be administered. The decision to use bevacizumab must be made prior to randomization.<br>See Section 6.1.2 and Section 6.1.3.2 |
| Assess study intervention compliance | X | | X | | | See Section 6.4 |

090177e194745c57\Approved\Approved On: 22-Jul-2020 00:48 (GMT)

**Table 4. Schedule of Activities (Control Arm [Arm C]: mFOLFOX6, FOLFIRI or FOLFOXIRI ± Bevacizumab)**

| **Evaluation** Abbreviations used in this table may be found in Appendix 11. | **Treatment Period (Each 28 Day Cycle)** | | **Post-Treatment Period** | | | **Notes/Protocol Section** |
|---|---|---|---|---|---|---|
| | **Day 1** | **Day 15** | **End-of-Treatment Visit** | **Follow-up Visit** | **Long Term Follow-up** | |
| **Window** | **± 3D** | **± 3D** | **± 3D** | **28 Days ± 7D** | **Q12W ± 7D** | |
| **Clinical Procedures/Assessments** | | | | | | |
| Physical exam | X | | X | X | | Does not need to be repeated if performed within 72 hours prior to Cycle 1 Day 1 (ie, first day of dosing). See Section 8.2.3 |
| Weight | X | | | | | Does not need to be repeated if performed within 72 hours prior to Cycle 1 Day 1 (ie, first day of dosing). See Section 8.2.3 |
| Height | X | | | | | Height to be measured in participants <18 years old. See Section 8.2.3 |
| BSA | X | | | | | Does not need to be repeated if performed within 72 hours prior to Cycle 1 Day 1 (ie, first day of dosing). See Section 8.2.4 |
| Vital signs | X | X | X | X | | See Section 8.2.6 |
| Triplicate 12-lead ECG | Cycle 1 | | | | | Predose only. See Section 8.2.7 |
| Single 12-lead ECG | Cycles 2+ | | X | X | | Predose only. Perform continuous ECGs if QTc abnormality. See Section 8.2.7 |

090177e194745c57\Approved\Approved On: 22-Jul-2020 00:48 (GMT)

**Table 4. Schedule of Activities (Control Arm [Arm C]: mFOLFOX6, FOLFIRI or FOLFOXIRI ± Bevacizumab)**

| **Evaluation** Abbreviations used in this table may be found in Appendix 11. | **Treatment Period (Each 28 Day Cycle)** | | **Post-Treatment Period** | | | **Notes/Protocol Section** |
|---|---|---|---|---|---|---|
| | **Day 1** | **Day 15** | **End-of-Treatment Visit** | **Follow-up Visit** | **Long Term Follow-up** | |
| **Window** | **± 3D** | **± 3D** | **± 3D** | **28 Days ± 7D** | **Q12W ± 7D** | |
| Tumor radiographic assessment (CT, MRI) | Q6W for the first 18 months after the date of randomization then Q8W thereafter or until BICR-confirmed PD, withdrawal of consent/assent, initiation of subsequent anticancer therapy, participant is lost to follow-up, death or defined End of Study. | | | | | The first post-baseline scan must be performed 42 to 48 days from the date of randomization.<br>See Section 8.1.1 |
| Adverse event assessment | Assess continuously | | | | | See Section 8.3 and Appendix 3 |
| **Laboratory Assessments** | | | | | | |
| Hematology | X | X | X | X | | Does not need to be repeated if performed within 72 hours prior to Cycle 1 Day 1 (ie, first day of dosing).<br>See Section 8.2.8 and Table 32 |
| Chemistry | X | X | X | X | | Does not need to be repeated if performed within 72 hours prior to Cycle 1 Day 1 (ie, first day of dosing).<br>See Section 8.2.8 and Table 32 |
| Coagulation | X | X | X | X | | Does not need to be repeated if performed within 72 hours prior to Cycle 1 Day 1 (ie, first day of dosing).<br>See Section 8.2.8 and Table 32 |
| Urinalysis | X | | X | X | | Does not need to be repeated if performed within 72 hours prior to Cycle 1 Day 1 (ie, first day of dosing).<br>Full urinalysis on Cycle 1 Day 1, only urine protein thereafter.<br>See Section 8.2.8 and Table 32 |

090177e194745c57\Approved\Approved On: 22-Jul-2020 00:48 (GMT)

**Table 4. Schedule of Activities (Control Arm [Arm C]: mFOLFOX6, FOLFIRI or FOLFOXIRI ± Bevacizumab)**

| **Evaluation** Abbreviations used in this table may be found in Appendix 11. | **Treatment Period (Each 28 Day Cycle)** | | **Post-Treatment Period** | | | **Notes/Protocol Section** |
|---|---|---|---|---|---|---|
| | **Day 1** | **Day 15** | **End-of-Treatment Visit** | **Follow-up Visit** | **Long Term Follow-up** | |
| **Window** | **± 3D** | **± 3D** | **± 3D** | **28 Days ± 7D** | **Q12W ± 7D** | |
| Urine pregnancy test | X | | X | | | Does not need to be repeated if performed within 72 hours prior to Cycle 1 Day 1 (ie, first day of dosing). See Section 8.2.9 and Table 32 |
| Blood samples for CRP | X | | X | | | See Section 8.8.1 |
| Blood sample for CEA | X | | X | | | See Section 8.8.1 |
| Blood sample for cfDNA | Cycles 1,7 | Cycles 1, 2 | X | | | Collect 20 mL of blood optimized for plasma preparation at indicated visits (all pre-dose). See Section 8.8.3 |
| Blood sample for protein biomarkers | Cycles 1, 2 | Cycles 1, 2 | X | | | Collect 10 mL of whole blood for processing into serum at indicated visits (all pre-dose). See Section 8.8.5 |
| Blood sample for germline comparator | X | | | | | Collect a 4 mL blood biospecimen prior to first dose of study intervention. See Section 8.8.2 |

**Table 4. Schedule of Activities (Control Arm [Arm C]: mFOLFOX6, FOLFIRI or FOLFOXIRI ± Bevacizumab)**

| **Evaluation** Abbreviations used in this table may be found in Appendix 11. | **Treatment Period (Each 28 Day Cycle)** | | **Post-Treatment Period** | | | **Notes/Protocol Section** |
|---|---|---|---|---|---|---|
| | **Day 1** | **Day 15** | **End-of-Treatment Visit** | **Follow-up Visit** | **Long Term Follow-up** | |
| **Window** | **± 3D** | **± 3D** | **± 3D** | **28 Days ± 7D** | **Q12W ± 7D** | |
| Blood sample for banked biospecimen | X | | | | | Unless prohibited by local regulations or ethics committee decision, collect a blood sample (4 mL) optimized for genomic analysis before the first dose of study intervention.<br>If not collected on the designated collection day, collect at the next available time point when biospecimens are being collected in conjunction with a participant visit.<br>See Section 8.7.7 |
| FFPE Tumor Tissue | | | X (optional) | | | See Section 8.8.2 and Section 8.8.4 |



090177e194745c57\Approved\Approved On: 22-Jul-2020 00:48 (GMT)

090177e194745c57\Approved\Approved On: 22-Jul-2020 00:48 (GMT)

**Table 5. Schedule of Activities (Control Arm [Arm C]: CAPOX ± Bevacizumab)**

| **Evaluation/Window** Abbreviations used in this table may be found in Appendix 11. | **Treatment Period (Each 21 Day Cycle)** | **Post-Treatment Period** | | | **Notes/Protocol Section** |
|---|---|---|---|---|---|
| | **Day 1** | **End-of-Treatment Visit** | **Follow-up Visit** | **Long Term Follow-up** | |
| **Window** | **± 3D** | **± 3D** | **28 Days ± 7D** | **Q12W ± 7D** | |
| **Administrative Procedures** | | | | | |
| Verify inclusion/ exclusion criteria | X | | | | Must occur prior to first dose.<br>See Section 5.1.2 and Section 5.2.2 |
| Verify demography; medical history from Screening to Day 1 | X | | | | See Section 8.2.1 and Section 8.2.2 |
| Concomitant medication/therapies | Assess continuously | | | | See Section 6.5 |
| EORTC QLQ-C30, PGIS, PGIC and EQ-5D-5L | Q6W for the first 18 months after the date of randomization then Q8W thereafter or until End of Treatment | X | X | | While on treatment, PRO assessments should be done with the same frequency as the tumor radiographic visits.<br>PGIC will not be performed at baseline.<br>See Section 8.1.2 |
| ECOG PS | Cycle 1 | | | | Does not need to be repeated if performed within 72 hours prior to Cycle 1 Day 1 (ie, first day of dosing).<br>See Section 8.2.10 |
| Document survival status | | | X | X | After discontinuation of study intervention, participants will be followed approximately every 3 months until withdrawal of consent/assent, the participant is lost to follow-up, death or defined End of Study.<br>See Section 7.1 |

090177e194745c57\Approved\Approved On: 22-Jul-2020 00:48 (GMT)

**Table 5. Schedule of Activities (Control Arm [Arm C]: CAPOX ± Bevacizumab)**

| **Evaluation/Window** Abbreviations used in this table may be found in Appendix 11. | **Treatment Period (Each 21 Day Cycle)** | **Post-Treatment Period** | | | **Notes/Protocol Section** |
|---|---|---|---|---|---|
| | **Day 1** | **End-of-Treatment Visit** | **Follow-up Visit** | **Long Term Follow-up** | |
| **Window** | **± 3D** | **± 3D** | **28 Days ± 7D** | **Q12W ± 7D** | |
| Document subsequent therapies and dates of progression | | Assess continuously | | | See Section 7.1 |
| Dispense capecitabine (with diary) | X | | | | Choice of treatment regimen is at the participant/treating Investigator's discretion but must be declared prior to randomization and must not be changed during the course of the participant's treatment.<br>See Section 6.1.3.2 |
| Oxaliplatin administration | X | | | | See Section 6.1.3.2 |
| Bevacizumab administration (optional) | X | | | | If the investigator decides to administer bevacizumab (Zirabev™ or Avastin®), Zirabev™ must be administered where approved. Where Zirabev™ is not approved, Avastin® may be administered. The decision to use bevacizumab must be made prior to randomization.<br>See Section 6.1.2 and 6.1.3.2 |
| Assess study intervention compliance | X | X | | | See Section 6.4 |
| **Clinical Procedures/Assessments** | | | | | |
| Physical exam | X | X | X | | Does not need to be repeated if performed within 72 hours prior to Cycle 1 Day 1 (ie, first day of dosing).<br>See Section 8.2.3 |

090177e194745c57\Approved\Approved On: 22-Jul-2020 00:48 (GMT)

**Table 5. Schedule of Activities (Control Arm [Arm C]: CAPOX ± Bevacizumab)**

| **Evaluation/Window** Abbreviations used in this table may be found in Appendix 11. | **Treatment Period (Each 21 Day Cycle)** | **Post-Treatment Period** | | | **Notes/Protocol Section** |
|---|---|---|---|---|---|
| | **Day 1** | **End-of-Treatment Visit** | **Follow-up Visit** | **Long Term Follow-up** | |
| **Window** | **± 3D** | **± 3D** | **28 Days ± 7D** | **Q12W ± 7D** | |
| Weight | X | | | | Does not need to be repeated if performed within 72 hours prior to Cycle 1 Day 1 (ie, first day of dosing). See Section 8.2.3 |
| Height | X | | | | Height to be measured in participants <18 years old. See Section 8.2.3 |
| BSA | X | | | | Does not need to be repeated if performed within 72 hours prior to Cycle 1 Day 1 (ie, first day of dosing). See Section 8.2.4 |
| Vital signs | X | X | X | | See Section 8.2.6 |
| Triplicate 12-lead ECG | Cycle 1 | | | | Predose only. See Section 8.2.7 |
| Single 12-lead ECG | Cycles 2+ | X | X | | Predose only. Perform continuous ECGs if QTc abnormality. See Section 8.2.7 |
| Tumor radiographic assessment | Q6W for the first 18 months after the date of randomization then Q8W thereafter or until BICR-confirmed PD, withdrawal of consent/assent, initiation of subsequent anticancer therapy, participant is lost to follow-up, death or defined End of Study. | | | | The first post-baseline scan must be performed 42 to 48 days from the date of randomization. See Section 8.1.1 |
| Adverse event assessment | Assess continuously | | | | See Section 8.3 and Appendix 3 |

090177e194745c57\Approved\Approved On: 22-Jul-2020 00:48 (GMT)

**Table 5. Schedule of Activities (Control Arm [Arm C]: CAPOX ± Bevacizumab)**

| **Evaluation/Window** Abbreviations used in this table may be found in Appendix 11. | **Treatment Period (Each 21 Day Cycle)** | **Post-Treatment Period** | | | **Notes/Protocol Section** |
|---|---|---|---|---|---|
| | **Day 1** | **End-of-Treatment Visit** | **Follow-up Visit** | **Long Term Follow-up** | |
| **Window** | **± 3D** | **± 3D** | **28 Days ± 7D** | **Q12W ± 7D** | |
| **Laboratory Assessments** | | | | | |
| Hematology | X | X | X | | Does not need to be repeated if performed within 72 hours prior to Cycle 1 Day 1 (ie, first day of dosing). See Section 8.2.8 and Table 32 |
| Chemistry | X | X | X | | Does not need to be repeated if performed within 72 hours prior to Cycle 1 Day 1 (ie, first day of dosing). See Section 8.2.8 and Table 32 |
| Coagulation | X | X | X | | Does not need to be repeated if performed within 72 hours prior to Cycle 1 Day 1 (ie, first day of dosing). See Section 8.2.8 and Table 32 |
| Urinalysis | X | X | X | | Does not need to be repeated if performed within 72 hours prior to Cycle 1 Day 1 (ie, first day of dosing). Full urinalysis on Cycle 1 Day 1, only urine protein thereafter. See Section 8.2.8 and Table 32 |
| Urine pregnancy test | X | X | | | Does not need to be repeated if performed within 72 hours prior to Cycle 1 Day 1 (ie, first day of dosing). See Section 8.2.9 and Table 32 |
| Blood sample for CRP | X | X | | | See Section 8.8.1 |
| Blood sample for CEA | X | X | | | See Section 8.8.1 |

090177e194745c57\Approved\Approved On: 22-Jul-2020 00:48 (GMT)

**Table 5. Schedule of Activities (Control Arm [Arm C]: CAPOX ± Bevacizumab)**

| **Evaluation/Window** Abbreviations used in this table may be found in Appendix 11. | **Treatment Period (Each 21 Day Cycle)** | **Post-Treatment Period** | | | **Notes/Protocol Section** |
|---|---|---|---|---|---|
| | **Day 1** | **End-of-Treatment Visit** | **Follow-up Visit** | **Long Term Follow-up** | |
| **Window** | **± 3D** | **± 3D** | **28 Days ± 7D** | **Q12W ± 7D** | |
| Blood sample for cfDNA | Cycles 1-3, 9 | X | | | Collect 20 mL of blood optimized for plasma preparation at indicated visits (all pre-dose).<br>See Section 8.8.3 |
| Blood sample for protein biomarkers | Cycles 1-3 | X | | | Collect 10 mL of whole blood for processing into serum at indicated visits (all pre-dose).<br>See Section 8.8.5 |
| Blood sample for germline comparator | X | | | | Collect a 4 mL blood biospecimen prior to first dose of study intervention.<br>See Section 8.8.2 |
| Blood sample for banked biospecimen | X | | | | Unless prohibited by local regulations or ethics committee decision, collect a blood sample (4 mL) optimized for genomic analysis before the first dose of study intervention.<br>If not collected on the designated collection day, collect at the next available time point when biospecimens are being collected in conjunction with a participant visit.<br>See Section 8.7.7 |
| FFPE tumor tissue | | X (optional) | | | See Section 8.8.2 and Section 8.8.4 |

090177e194745c57\Approved\Approved On: 22-Jul-2020 00:48 (GMT)

# 2. INTRODUCTION

Encorafenib (BRAFTOVI®, also known as PF-07263896, LGX818 or ONO-7702) is being investigated in this study in combination with cetuximab with or without mFOLFOX6 or FOLFIRI in participants with previously untreated metastatic *BRAF* V600E-mutant CRC.

## 2.1. Study Rationale

The purpose of the study is to evaluate whether encorafenib plus cetuximab (EC), alone or in combination with chemotherapy, can improve clinical outcomes relative to current standard-of-care chemotherapy in participants with previously untreated *BRAF* V600E-mutant mCRC. Since encorafenib has not previously been combined with chemotherapy, the tolerability and PK of EC in combination with mFOLFOX6 and in combination with FOLFIRI will be evaluated in separate cohorts in the safety lead-in portion of the trial in order to identify which chemotherapy combination is to be used in the Phase 3 portion of the study.

## 2.2. Background

### 2.2.1. *BRAF* V600E-Mutant Metastatic Colorectal Cancer

Metastatic colorectal cancer continues to be a life-threatening condition. Globally, colorectal cancer is the third most commonly diagnosed cancer in males and the second in females, with 1.8 million new cases and almost 861,000 deaths in 2018 according to the WHO.[1] In the US, according to the SEER database, the estimated number of new CRC cases for 2019 was 145,600 resulting in an estimated 51,000 deaths.[2] In Japan, CRC is the second most common cancer in women with over 150,000 new cases in 2017; there were over 50,000 deaths due to CRC and it was the leading cause of cancer death in women and third most common cause of cancer death in men in 2018.[3] In the WHO European Region, CRC is the most common tumor type, with 471,000 new cases each year and a mean mortality rate of 28.2 per 100,000 population.[4] Approximately 25% of patients present with metastases and 50% of patients eventually develop metastatic disease. [5]

*BRAF* mutations occur in approximately 10% to 15% of CRC, with a broad range of estimates ranging from as low as 5% to as high as 21%. These mutations primarily (>95%) occur at the V600E codon (consisting of a valine to glutamic acid substitution), are essentially mutually exclusive with *RAS* mutations,[6-11] and lead to constitutive activation of BRAF kinase and sustained RAS/RAF/MEK/ERK pathway signaling, resulting in increased cell proliferation and survival.[12]

*BRAF* mutations are associated with a poor prognosis.[13-17] Retrospective analyses of Phase 3 studies using standard regimens revealed patients with *BRAF*-mutant mCRC achieved mPFS of approximately 7 months and mOS of approximately 12 months compared to approximately 10 months PFS and 25 months OS in patients with *BRAF*-wt tumors.[17-23]

Previous studies have demonstrated an association between the presence of MSI-H or dMMR and presence of *BRAF* mutations in CRC tumors. Approximately 15-30% of *BRAF*-mutant mCRC tumors were also identified as MSI-H/dMMR.[16, 17, 24, 25] Immune checkpoint

inhibitors have been shown to be active in patients with previously treated MSI-H/dMMR mCRC[24, 26] and more recently in previously untreated patients with mCRC as well.[27]

### 2.2.2. Clinical Overview

#### 2.2.2.1. Current Recommendations for Treatment of BRAF V600E-mutant mCRC

Currently, no treatments are indicated specifically for patients with *BRAF* V600E-mutant mCRC in the first-line setting and thus patients receive systemic therapy that is recommended for mCRC in general. The common regimens used in patients with mCRC include mFOLFOX6 (oxaliplatin, leucovorin and fluorouracil [5-FU]), FOLFIRI (irinotecan, leucovorin and 5-FU), FOLFOXIRI (irinotecan, oxaliplatin, leucovorin and 5-FU) and CAPOX (oxaliplatin and capecitabine) each with or without bevacizumab.[28] Retrospective subgroup analyses of Phase 2 and 3 studies in previously untreated patients with mCRC patients have shown that all of the standard regimens have similar efficacy in patients with *BRAF*-mutant tumors (Table 6).

**Table 6. Efficacy of Standard of Care First-Line Therapies in Patients with *BRAF*-mutant mCRC**

| Regimen | *BRAF*-mutant n (%) | Median PFS (Months) | Median OS (Months) |
|---|---|---|---|
| FOLFOX4[18] | 25 (12.5) | 3.8 | NR |
| FOLFOX4 + cetuximab[18] | 27 (14.0) | 2.0 | NR |
| FOLFIRI + bevacizumab[23] | 12 (4.7) | 5.5 | 10.7 |
| FOLFOXIRI + bevacizumab[23] | 16 (6.3) | 7.5 | 19.0 |
| FOLFIRI or FOLFOX-4 (pooled)[19] | 38 (8.5) | 3.7 | 9.9 |
| FOLFIRI or FOLFOX-4 (pooled) + cetuximab[19] | 32 (8.0) | 7.1 | 14.1 |
| FOLFIRI + cetuximab[22] | 23 (7.7) | 6.6 | 12.3 |
| FOLFIRI + bevacizumab[22] | 25 (8.5) | 6.6 | 13.7 |
| CAIRO: capecitabine + irinotecan + oxaliplatin (sequential vs combination)[17]<br>CAIRO2: capecitabine + oxaliplatin + bevacizumab ± cetuximab[17]<br>COIN: capecitabine (or 5-FU + leucovorin) + oxaliplatin ± cetuximab[17]<br>FOCUS: 5-FU + leucovorin + irinotecan vs 5-FU + leucovorin ± irinotecan + oxaliplatin[17] | 250 (8.2) | 6.2 | 11.4 |
| CAPOX + bevacizumab in induction + capecitabine + bevacizumab[21] | 30 (5.4) | 9.5 | 15.8 |

#### 2.2.2.2. Encorafenib + Cetuximab for the Treatment of BRAF V600E-mutant mCRC

Encorafenib is a highly selective ATP-competitive small-molecule RAF kinase inhibitor, with the greatest sensitivity observed in melanoma and CRC tumor cell lineages expressing



090177e194745c57\Approved\Approved On: 22-Jul-2020 00:48 (GMT)

*BRAF* V600E.[29] Encorafenib in combination with binimetinib (a potent and selective allosteric, ATP-uncompetitive inhibitor of MEK1/2) has received marketing approval in several jurisdictions for the treatment of patients with unresectable or metastatic melanoma with a *BRAF* V600E or V600K mutation.

Study ARRAY-818-302 (BEACON CRC; NCT02928224) is a Phase 3 trial in patients with *BRAF* V600E-mutant mCRC whose disease had progressed following 1 or 2 prior systemic therapies. Patients in the study were randomized 1:1:1 to receive either EC, EC + binimetinib, or Control (irinotecan + cetuximab or FOLFIRI + cetuximab). The dosage of encorafenib in all patients was 300 mg QD PO. The dosage of cetuximab in all patients was 400 mg/m$^2$ IV for the first dose followed by 250 mg/m$^2$ IV weekly.

In April 2020, based on the results of the Study ARRAY-818-302, the EC regimen received marketing approval in the US for the treatment of adult patients with mCRC with a *BRAF* V600E mutation, as detected by an FDA-approved test, after prior therapy.[30] In June 2020, the EC regimen received approval from the European Commission for the same indication.

### Efficacy

A total of 220 patients were randomized to the EC arm and 221 to the Control Arm. The EC regimen demonstrated statistically significant improvement in OS, ORR, and PFS compared to the Control Arm as shown in Table 7. The addition of binimetinib to EC did not improve efficacy (ORR, PFS, or OS) significantly compared with EC.[31]

**Table 7. Study ARRAY-818-302 (EC Arm and Control Arm): Efficacy Results**

| | **EC**<br>**N = 220** | **Irinotecan + Cetuximab or FOLFIRI + Cetuximab**<br>**N = 221** |
|---|---|---|
| Overall Survival | | |
| Median OS, months (95% CI) | 8.4 (7.5, 11.0) | 5.4 (4.8, 6.6) |
| HR (95% CI) | 0.60 (0.45, 0.79) | |
| p-value | <0.001 | |
| Overall Response Rate (per BICR) | | |
| ORR (95% CI) | 20% (13%, 29%) | 2% (0%, 7%) |
| CR | 5% | 0% |
| PR | 15% | 2% |
| Progression Free Survival (per BICR) | | |
| Median PFS, months (95% CI) | 4.2 (3.7, 5.4) | 1.5 (1.5, 1.7) |
| HR (95% CI) | 0.40 (0.31, 0.52) | |
| *P*-value | <0.001 | |

090177e194745c57\Approved\Approved On: 22-Jul-2020 00:48 (GMT)

**Safety**

In study ARRAY-818-302, the safety of the EC regimen was evaluated in 216 patients. The most common adverse events in the EC treated patients were gastrointestinal-related and skin-related events, including nausea, diarrhea, and acneiform dermatitis (Table 8). Most of these toxicities were generally reversible and manageable by supportive medical care, dose modifications or discontinuations. Other important adverse events occurring in <15% of patients in the EC arm were melanocytic nevi (14%), and myalgia (13%).[31]

**Table 8. Study ARRAY-818-302 (EC Arm and Control Arm): Adverse Events Regardless of Causality Reported in ≥15% of EC Patients**

| | EC (N = 216) | | Irinotecan + Cetuximab or FOLFIRI + Cetuximab (N = 193) | |
|---|---|---|---|---|
| | **Any Grade n (%)** | **Grade ≥3 n (%)** | **Any Grade n (%)** | **Grade ≥3 n (%)** |
| Any adverse event | 212 (98) | 108 (50) | 188 (97) | 117 (61) |
| Nausea | 74 (34) | 1 (<1) | 80 (41) | 2 (1) |
| Diarrhea | 72 (33) | 4 (2) | 93 (48) | 19 (10) |
| Fatigue | 65 (30) | 9 (4) | 53 (27) | 8 (4) |
| Acneiform dermatitis | 63 (29) | 1 (<1) | 76 (39) | 5 (3) |
| Decreased appetite | 58 (27) | 3 (1) | 52 (27) | 6 (3) |
| Abdominal pain | 49 (23) | 5 (2) | 48 (25) | 9 (5) |
| Vomiting | 46 (21) | 3 (1) | 56 (29) | 5 (3) |
| Asthenia | 46 (21) | 7 (3) | 49 (25) | 9 (5) |
| Headache | 42 (19) | 0 (0) | 5 (3) | 0 (0) |
| Arthralgia | 41 (19) | 2 (1) | 1 (1) | 0 (0) |
| Pyrexia | 35 (16) | 2 (1) | 27 (14) | 1 (1) |
| Constipation | 33 (15) | 0 (0) | 35 (18) | 2 (1) |

The most common laboratory abnormalities (≥2%, Grade ≥3) in patients treated in the EC arm were elevated bilirubin (2%), elevated creatinine (2%), and decreased hemoglobin (4%).[31]

## 2.3. Benefit/Risk Assessment

More detailed information about the known and expected benefits and risks and reasonably expected AEs of encorafenib may be found in the Investigator's Brochure, which is the SRSD for this study. The SRSDs for capecitabine and cetuximab are the respective EU SmPCs. The SRSDs for 5-FU, irinotecan, leucovorin, levoleucovorin, and oxaliplatin are the respective SmPCs.

In the Control Arm of Phase 3, investigators may choose to administer an anti-VEGF agent. If this decision is made to use an anti-VEGF agent, Zirabev™ must be administered where approved. In regions where Zirabev™ is not approved, participants will receive Avastin ® .



090177e194745c57\Approved\Approved On: 22-Jul-2020 00:48 (GMT)

Hereafter, Zirabev™ and Avastin® are collectively referred to as bevacizumab. The SRSD for these agents is the Avastin® EU SmPC.

Detailed information about the benefits and risks of each study intervention, when administered as a single agent, can be found in the respective SRSDs. Potential risks of DDIs and synergistic toxicities are outlined in Section 2.3.1.

090177e194745c57\Approved\Approved On: 22-Jul-2020 00:48 (GMT)

090177e194745c57\Approved\Approved On: 22-Jul-2020 00:48 (GMT)

### 2.3.1. Risk Assessment

| Potential Risk of Clinical Significance | Summary of Data/Rationale for Risk | Mitigation Strategy |
|---|---|---|
| **Study Intervention: Treatment Regimens** | | |
| Potential Risk of Drug-Drug Interaction | | |
| Over-exposure of irinotecan/SN-38 due to CYP3A4 inhibition by encorafenib | Encorafenib is a reversible inhibitor of UGT1A1 and CYP3A, and a time-dependent inhibitor of CYP3A4 at clinically relevant plasma concentrations. Irinotecan and its active metabolite, SN-38, are metabolized via CYP3A4 and UGT1A1.<br>Coadministration of irinotecan with inhibitors of CYP3A4 may increase systemic exposure to irinotecan or SN-38.<br>Concomitant administration of Encorafenib with irinotecan may result in increased toxicity of irinotecan (see Section 4.2.2). | Patients with reduced UGT1A1 enzyme activity (Gilbert's syndrome and known homozygous UGT1A1*28/*28 or UGT1A1*6/*6 genotypes) or double heterozygous UGT1A1*6/*28 genotype are excluded from this study (see Exclusion Criteria 6 for details).<br>Encorafenib will be initiated on Cycle 1 Day 3, 48 hours after irinotecan infusion to avoid potential over-exposure of irinotecan and SN-38. While on study, if encorafenib is interrupted for ≥4 consecutive days, it will be restarted ≥2 days prior to the next planned irinotecan infusion or ≥2 days after the next irinotecan infusion. |
| Potential Risks of Synergistic Toxicities | | |
| QT prolongation | In the single-agent encorafenib study CLGX818X2101, an increase of 12.2 milliseconds in QTcF was predicted following 450 mg encorafenib.<br>In the pooled encorafenib single-agent population, the incidence of new QTc prolongation >500 ms or change in QTc >60 ms from baseline (Grade 3/4), was 1.0% (3/301). There was 1 serious Grade 3 event of QT prolongation, which resolved after permanent discontinuation of encorafenib. There were no AEs of Torsades de pointes or ventricular arrhythmias in patients in the encorafenib single-agent pool.<br>In study ARRAY-818-302, 7 (3.2%) patients in the Doublet arm (encorafenib + cetuximab) had a newly occurring QTcF > 500 ms and increases from baseline of >60 ms were observed in 19 (8.8%) patients. There were no AEs of torsades de pointes, ventricular arrhythmias, pre-syncope, syncope, sudden death or cardiac | Risk for QT prolongation is appropriately described in the encorafenib IB.<br>Administration with medicinal products with a known potential to prolong QT/QTc should be avoided, where possible.<br>Participants at high risk of QT prolongation are excluded from the study as outlined in Section 5.2.2.<br>Monitoring and dose adjustments for QT prolongation are outlined in Table 15. |

090177e194745c57\Approved\Approved On: 22-Jul-2020 00:48 (GMT)

| Potential Risk of Clinical Significance | Summary of Data/Rationale for Risk | Mitigation Strategy |
|---|---|---|
| **Study Intervention: Treatment Regimens** | | |
| | arrest reported contemporaneously with ECG QTcF prolongation > 500 ms.<br>The CredibleMeds® database (https://www.crediblemeds.org/) lists oxaliplatin as having a known risk of TdP cardiac arrhythmia and 5-FU as having a potential risk of TdP. Both are listed as drugs to avoid in patients with congenital LQTS. | |
| Peripheral Neuropathy | AEs related to peripheral neuropathies may potentially be more severe and/or more frequent in patients receiving combination treatment regimens with encorafenib and oxaliplatin.<br>In study ARRAY-818-302, 26 (12%) patients in the Doublet arm (encorafenib + cetuximab) had a peripheral neuropathy AE of any Grade and 3 (1.4%) patients had a Grade 3+ event. Most of the 26 AEs were PTs for sensory neuropathies. Almost all patients (95.5%) had received prior oxaliplatin. In the Phase 3 study of binimetinib + encorafenib versus vemurafenib in patients with BRAF-mutant melanoma (study CMEK162B2301), the rates of peripheral neuropathy were 12% and 13%, respectively.<br>According to the oxaliplatin SmPC, symptoms of dysaesthesia/paraesthesia occur in up to 95% of patients and are most often reversible, although about 3% of patients present with persisting paraesthesias of moderate severity up to 3 years after discontinuation of treatment. Approximately 0.5% of patients have decreased functional activities that are unresolved after discontinuation. | Dose modifications for oxaliplatin in the setting of neurotoxicity are described in Table 20.<br>Nervous system disorders including peripheral neuropathy are described in the IB and considered identified risks for encorafenib. Dose modification for encorafenib in the setting of peripheral neuropathy AEs are described in Table 15. |
| Gastrointestinal Toxicities | AEs related to GI toxicities may potentially be more severe and/or more frequent in patients receiving combination treatment regimens with encorafenib + cetuximab concomitantly with mFOLFOX6 or FOLFIRI.<br>In ARRAY-818-302, diarrhea, nausea, and vomiting occurred in 21-34% of the patients in the Doublet arm (encorafenib + cetuximab). Seventy-two (33%) patients had diarrhea ADRs (4 patients -Grade 3+), 74 (34%) patients had nausea ADRs (1 patient - Grade 3+), and 46 (21%) had vomiting ADRs (3 Grade 3+).<br>Nausea and vomiting are common (≥40%) in patients receiving oxaliplatin. In patients treated with irinotecan in combination with 5-FU on a Q2W regimen, Grade 3-4 diarrhea occurs in in approximately 14% of patients. 5-FU is also known to | GI toxicities including diarrhea, nausea, and vomiting are known ADRs for encorafenib in combination with cetuximab and described in the encorafenib US and EU product labels.<br>Dose modifications for AEs of GI toxicity are described for the various treatment regimens in Section 6.5.3. |

090177e194745c57\Approved\Approved On: 22-Jul-2020 00:48 (GMT)

| Potential Risk of Clinical Significance | Summary of Data/Rationale for Risk | Mitigation Strategy |
|---|---|---|
| **Study Intervention: Treatment Regimens** | | |
| | cause severe diarrhea requiring electrolyte replacement and antidiarrheal therapy. | |
| **Study Procedures** | | |
| CT or MRI procedures for tumor assessments. | CT scans expose the participant to a small dose of radiation. The contrast dye used with the CT scan may cause pain or burning when injected. The dye may worsen kidney function for those with kidney disease or are dehydrated. The dye may cause an allergic reaction.<br>MRI risks include possible reactions to metals due to magnets, loud noises from the machine causing hearing issues, increase in body temperature during long MRIs, and claustrophobia.<br>Participants in this study will be subjected to more frequent (every 6 weeks) CT/MRI scans than is typical for mCRC patients (every 8 weeks) for the first 18 months of the study. Based on this scanning schedule, a patient would receive 3 additional scans in the 18 month period compared with the typical scanning regimen. | Only experienced professionals will conduct the CT or MRI procedures. The participant will be asked about allergies to the contrast dye. Medical history will be reviewed for kidney disease, and information will be provided to minimize dehydration. If possible, an open MRI can be used for those experiencing claustrophobia. The participant may be offered earplugs and/or headphones to minimize the noise. |
| **Other** | | |
| *BRAF* testing | Patients will be eligible for the study based on identification of a *BRAF* V600E mutation. Inaccurate *BRAF* testing (ie false positive or false negative) may lead to inappropriate enrollment to or exclusion from this study. A participant with a false negative result may be excluded from receiving potentially beneficial study intervention. Alternatively, a false positive result may lead a participant to receive study intervention, which may be inappropriate given the tumor mutation status. | In cases where there is discordance between the local assay and central laboratory results, or if the central laboratory is not able to confirm presence of a *BRAF* V600E mutation due to inadequate or poor sample condition within 30 days of initiating study therapy, participants may only continue treatment if there is no clinical indication of deterioration or disease progression and the investigator determines that the participant is deriving benefit. In such instances, participants must be informed that the *BRAF* mutation status is unconfirmed and must acknowledge on the ICDs that includes this information and describes alternative treatment options. |

| Potential Risk of Clinical Significance | Summary of Data/Rationale for Risk | Mitigation Strategy |
|---|---|---|
| **Study Intervention: Treatment Regimens** | | |

***Note***: The pooled encorafenib single-agent population data set was derived from 4 clinical studies (CLGX818X2103 [Phase 1b/2], CMEK162B2301 Part 1 [Phase 3], CMEK162X2102 [Phase 2], CLGX818AUS03 [Phase 2]) with a total of 301 total patients.

090177e194745c57\Approved\Approved On: 22-Jul-2020 00:48 (GMT)

### 2.3.2. Benefit Assessment

The EC regimen is approved in several countries for the treatment of patients with *BRAF* V600E-mutant mCRC that has progressed after 1 or 2 prior regimens (Section 2.2.2). Based on the activity observed in previously treated patients, there is the potential for the EC regimen to be efficacious in the first-line metastatic setting in patients with *BRAF* V600E-mutant mCRC. Furthermore, based on its demonstrated activity in patients with *BRAF* V600E-mutant mCRC, there is the potential for additional benefits to patients when EC is added to chemotherapy regimens that are the current SOC.

### 2.3.3. Overall Benefit/Risk Conclusion

Considering the measures taken to minimize risk to study participants, the potential risks identified in association with the EC regimen with or without mFOLFOX6 or FOLFIRI are justified by the anticipated benefits that may be afforded to participants with *BRAF* V600E-mutant mCRC.

## 3. OBJECTIVES, ESTIMANDS, AND ENDPOINTS

| **Safety Lead-in** | |
|---|---|
| **Objectives** | **Endpoints** |
| **Primary** | |
| • To determine the safety and tolerability of EC + mFOLFOX6 and EC + FOLFIRI | • Incidence of DLTs |
| **Secondary** | |
| • To assess the overall safety and tolerability of EC + mFOLFOX6 and EC + FOLFIRI | • Incidence and severity of AEs graded according to the NCI CTCAE v4.03 and changes in clinical laboratory parameters, vital signs and ECGs<br>• Incidence of dose interruptions, dose modifications and discontinuations due to AEs |
| • To estimate the efficacy of EC + mFOLFOX6 and EC + FOLFIRI | • ORR by Investigator, defined as the proportion of participants who have achieved a confirmed BOR (CR or PR) per RECIST v1.1<br>• DOR by Investigator, defined as the time from the date of first radiographic evidence of response (CR or PR) to the earliest documented disease progression per RECIST v1.1, or death due to any cause<br>• PFS by Investigator, defined as the time from the first dose to the earliest documented disease progression per RECIST v1.1, or death due to any cause<br>• TTR by Investigator, defined as the time from first dose to first radiographic evidence of response (CR or PR) per RECIST v1.1 |
| • To estimate the efficacy of EC + mFOLFOX6 and EC + FOLFIRI | • OS defined as the time from the first dose to death due to any cause |
| • To characterize the PK of encorafenib, irinotecan, oxaliplatin and relevant metabolites | • PK parameters of encorafenib, irinotecan, oxaliplatin and relevant metabolites |



090177e194745c57\Approved\Approved On: 22-Jul-2020 00:48 (GMT)

| | |
|---|---|
| • To assess drug-drug interaction of encorafenib with irinotecan or oxaliplatin | • Changes in exposures of irinotecan and its metabolite (SN-38) on Cycle 1 Day 15 compared to Cycle 1 Day 1 in Cohort 1 (EC + FOLFIRI)<br>• Changes in exposures of oxaliplatin on Cycle 1 Day 15 compared to Cycle 1 Day 1 in Cohort 2 (EC + mFOLFOX6) |
| **Tertiary/Exploratory** | |
| • To understand the relationship between the therapeutic intervention(s) being studied and the biology of the participant's disease | • Measurements of biomarkers, consisting of DNA, RNA, protein or defined cell types, resulting from analyses of peripheral blood and/or tumor tissue biospecimen obtained at baseline, on treatment and/or at end-of-study |
| **Randomized Phase 3** | |
| **Objectives** | **Endpoints** |
| **Primary** | |
| • To compare the efficacy of EC (Arm A) vs SOC (Control Arm [Arm C]) as measured by PFS<br>• To compare the efficacy EC + mFOLFOX6 or EC + FOLFIRI (Arm B) vs the Control Arm as measured by PFS | • PFS by BICR, defined as the time from the date of randomization to the earliest documented disease progression per RECIST v1.1, or death due to any cause |
| **Key Secondary** | |
| • To compare the efficacy of Arm A vs the Control Arm as measured by OS<br>• To compare the efficacy of Arm B vs the Control Arm as measured by OS | • OS, defined as the time from the date of randomization to death due to any cause |
| **Secondary** | |
| • To compare the efficacy of Arm A vs the Control Arm as measured by ORR, DOR, PFS, PFS2 and TTR<br>• To compare the efficacy of Arm B vs the Control Arm as measured by ORR, DOR, PFS, PFS2 and TTR<br>• To compare the efficacy of Arm A vs Arm B as measured by OS, PFS, PFS2, ORR, DOR and TTR | • ORR by BICR and by Investigator<br>• DOR by BICR and by Investigator<br>• PFS by BICR<br>• OS<br>• PFS by Investigator<br>• TTR (by BICR and by Investigator), defined as the time from the date of randomization to first radiographic evidence of response (CR or PR) per RECIST v1.1<br>• PFS2, defined as the time from the date of randomization to the second objective disease progression per RECIST v1.1, or death from any cause, whichever occurs first |
| • To determine the safety and tolerability of EC<br>• To determine the safety and tolerability of EC + mFOLFOX6 or EC + FOLFIRI | • Incidence and severity of AEs graded according to the NCI CTCAE v4.03 and changes in clinical laboratory parameters, vital signs, and ECGs |
| • To compare the PRO measures | • PROs as measured by the EORTC QLQ-C30, EQ-5D-5L, PGIS and PGIC. |

090177e194745c57\Approved\Approved On: 22-Jul-2020 00:48 (GMT)

| | |
|---|---|
| • To evaluate trough concentrations of encorafenib and its metabolite LHY746 in Arm A and Arm B | • Trough plasma concentrations of encorafenib and the metabolite LHY746 in Arm A and Arm B |
| • To characterize the PK of encorafenib and its metabolite LHY746 in participants randomized in China (Arm A) | • PK parameters of encorafenib and its metabolite LHY746 |
| • To confirm the MSI-status in tumor tissue | • Summarize MSI-status as determined by retrospective central testing of baseline tumor tissue |
| • To determine the correlation between cfDNA genetic alterations and clinical outcome | • *BRAF* V600E variant allele fraction (VAF) and/or overall mean VAF from cfDNA analysis of plasma samples collected at baseline and on treatment |
| **Tertiary/Exploratory** | |
| • To understand the relationship between the therapeutic intervention(s) being studied and the biology of the participant's disease | • Measurements of biomarkers, consisting of DNA, RNA, protein or defined cell types, resulting from analyses of peripheral blood and/or tumor tissue biospecimen obtained at baseline, on treatment and/or at end-of-study |

**Estimands**

The primary estimand in the SLI is the DLT rate estimated based on data from DLT-evaluable participants during the DLT-evaluation period, which is the first 28 days after the first dose of study intervention in the SLI. The number and proportion of participants experiencing DLTs during the DLT-evaluation period will be summarized for Cohort 1 (EC + FOLFIRI) and Cohort 2 (EC + mFOLFOX6), separately.

The primary estimand in the Phase 3 portion of the study is the treatment effect in PFS by BICR, as measured by the hazard ratio. PFS is defined as the time from date of randomization to the earliest documented disease progression per RECIST v1.1, or death due to any cause. The hypothetical strategy will be applied for the intercurrent events. PFS will be compared between Arm A and the Control Arm, and between Arm B and the Control Arm using a 1-sided stratified log-rank test.

A complete list of estimands are defined in Section 9.1.1.

# 4. STUDY DESIGN

## 4.1. Overall Design

This is an open-label, multicenter, randomized Phase 3 study of EC with or without chemotherapy versus standard of care chemotherapy in participants with previously untreated *BRAF* V600E-mutant mCRC. Prior to the Phase 3 portion, a SLI will be conducted at a limited number of sites to evaluate the safety/tolerability and PK of EC in combination with either mFOLFOX6 or FOLFIRI. Approximately 60 participants will be enrolled in the SLI portion (up to 30 per regimen). The results of the SLI will inform which chemotherapy regimen is used in Arm B of the Phase 3 portion of the study. In the Phase 3 portion, approximately 870 participants will be randomized 1:1:1 to receive EC (Arm A), EC +



090177e194745c57\Approved\Approved On: 22-Jul-2020 00:48 (GMT)

chemotherapy (Arm B) or SOC chemotherapy (Control Arm), with approximately 290 participants per arm. The primary objectives in the Phase 3 portion of the study are to compare the efficacy, as measured by the primary endpoint of PFS by BICR, of Arm A versus the Control Arm and of Arm B versus the Control Arm. The study will be considered positive (ie, demonstrated evidence of effectiveness) if at least one of the hypothesis tests of the primary endpoint is statistically significant.

## 4.2. Scientific Rationale for Study Design

### 4.2.1. Safety Lead-in

Since the EC regimen has not previously been combined with cytotoxic chemotherapy, a SLI will be conducted prior to the Phase 3 portion of the study to evaluate the safety/tolerability and PK of EC + mFOLFOX6 and EC + FOLFIRI. The results of the SLI will inform which chemotherapy regimen is used in Arm B of the Phase 3 portion of the study (see Section 6.1.1 for detailed description of SLI). The most frequently reported AEs (>25% of patients) among patients treated with EC in the BEACON CRC trial were nausea, diarrhea, fatigue, dermatitis acneiform, and decreased appetite. The potential overlapping toxicities between EC and the chemotherapy regimens are primarily GI toxicities that are managed routinely by treating physicians. Therefore, the safety and tolerability of the combinations will be assessed using the standard doses of all agents.

The SLI will enroll up to 30 participants per cohort to fully assess the safety/tolerability and PK, including the potential for DDI, of EC + mFOLFOX6 and EC + FOLFIRI. For each cohort, the probability of observing at least one instance of a toxicity in 30 participants is >95% when the true toxicity incidence rate is 10%. The decision to initiate Phase 3 will require approval from the study SC and the E-DMC. The E-DMC will also review safety data to confirm the tolerability of the EC + chemotherapy combination after the first 30 participants have been randomized and treated for at least 1 cycle in the Phase 3 portion of the study.

### 4.2.2. Assessment of Potential DDI with Encorafenib

Irinotecan is metabolized to SN-38, which is 100-1,000 times more potent than the parent. The exposures of both irinotecan and SN-38 have been shown to correlate with the efficacy and toxicity (eg, neutropenia and diarrhea) of irinotecan treatment.[32] SN-38 is conjugated by the enzyme UGT1A1 to form an inactive metabolite, SN-38G, which is readily eliminated.

An alternative pathway of irinotecan metabolism is CYP3A4-mediated oxidation, which produces inactive metabolites. Thus, the PK of irinotecan and SN-38 can be altered when co-administered with CYP3A4 inducers or inhibitors.[33] Therefore, strong CYP3A4 inducers or inhibitors should not be co-administered with irinotecan unless there are no therapeutic alternatives.[34]

Encorafenib is a reversible inhibitor of UGT1A1, CYP3A4, and other CYP450 enzymes, and is a time-dependent inhibitor of CYP3A4 at clinically relevant plasma concentrations. Encorafenib also induced CYP3A4 and other CYP450 enzymes at clinically relevant plasma concentrations. Furthermore, encorafenib inhibits multiple transporters that are also involved



090177e194745c57\Approved\Approved On: 22-Jul-2020 00:48 (GMT)

in disposition and elimination of irinotecan and its metabolites (SN-38 and SN-38G).[35, 36] Therefore, when co-administrated with FOLFIRI, encorafenib has the potential to alter the PK of irinotecan or SN-38 via changes in enzyme activities of CYP3A4, UGT1A1 and various transporters. The metabolism of oxaliplatin, 5-FU, and leucovorin is not mediated by CYP450 enzymes, and the risk of CYP450-mediated DDI of encorafenib with these agents is expected to be low.[37-39]

To avoid the potential increases in the rate of AEs due to higher exposure of irinotecan or SN-38 at the initiation of combination treatment, encorafenib QD dosing will be initiated on Cycle 1 Day 3, 48 hours (≥4 times the half-life of irinotecan) after the first dose of irinotecan.[34] By Cycle 1 Day 3, the plasma irinotecan concentration is expected to be reduced by >90% of the peak concentration, and thus any encorafenib-mediated effects on CYP3A4, UGT1A1 and/or transporters will have a minimal effect on the exposure of irinotecan and SN-38. If encorafenib is interrupted for ≥4 consecutive days, in order to minimize the potential for DDIs, encorafenib should be restarted ≥2 days prior to the next planned irinotecan infusion or ≥2 days after the next irinotecan infusion.

### 4.2.3. Investigator's Choice of Control Regimen

Treatment of investigator's choice was chosen as the comparator in the Phase 3 portion of the study because a number of therapeutic options are available as initial therapy for mCRC, allowing selection of the regimen believed to potentially confer the greatest clinical benefit to a given participant. Currently, no treatments are indicated specifically for patients with *BRAF* V600E-mutant mCRC in the first-line setting and there is no single SOC regimen established in this indication (see Table 6).

Clinical guidelines established by ASCO, ESMO, and JSMO suggest that mFOLFOX6, FOLFIRI, or CAPOX are all acceptable therapeutic options and should be combined with bevacizumab where possible for initial treatment of patients with mCRC irrespective of tumor *BRAF* status.[28, 40, 41] The more intensive combination of FOLFOXIRI + bevacizumab was also shown to be active based on retrospective analyses of clinical trial data although more recent data from a meta-analysis suggests that the triple combination of 5-FU, oxaliplatin, and irinotecan (with bevacizumab) has similar efficacy as doublet chemotherapy and bevacizumab in patients with *BRAF* V600E-mutant mCRC.[42] Since the FOLFOXIRI plus bevacizumab regimen is associated with greater toxicity, it is only recommended for patients who are defined as being fit and highly motivated in the ESMO guidelines or those with excellent performance status.

### 4.2.4. Progression-Free Survival as Primary Endpoint

The primary endpoint will be PFS by BICR, and OS will be the key secondary endpoint. Given the study design and nature of the disease, it is likely that at the time of the primary endpoint (ie, PFS) analysis, OS may still be immature, and significance may not have been observed. PFS as a primary endpoint with OS as a key secondary endpoint is commonly used in oncology trials including trials in first-line mCRC.[23, 43]

090177e194745c57\Approved\Approved On: 22-Jul-2020 00:48 (GMT)

According to the Dec 2018 FDA guidance on 'Clinical Trial Endpoints for the Approval of Cancer Drugs and Biologics,' a large improvement in PFS can be used to support traditional approval in select indications.[44] The EMA CHMP Guideline on the 'Evaluation of Anticancer Medicinal Products in Man' states that OS and PFS are acceptable endpoints, and recommends that if PFS is selected as the primary endpoint, OS should be used as a secondary endpoint.[45]

This is an open-label study, therefore the primary endpoint of PFS in the Phase 3 portion will be determined by BICR to minimize evaluation bias. An E-DMC will review data throughout the trial. An E-DMC Charter that outlines E-DMC membership, the data that will be reviewed and the timing and frequency of the reviews will be established. Because the assessment of PFS for determination of the primary endpoint will be based on a BICR, disease progression will be confirmed by expedited central review to minimize informative censoring.

### 4.2.5. Biomarkers

The biomarker analyses applied to pre-treatment, on-treatment (blood only) and end of treatment tumor and/or blood biospecimens are intended to evaluate established and candidate prognostic/predictive biomarkers that may identify participants who preferentially benefit from the study treatments and to evaluate mechanisms of action and/or resistance for each of the combinations being assessed.

*BRAF* and *KRAS* mutations are associated with poor prognosis in advanced CRC and are almost always mutually exclusive.[10, 46, 47] The predominant (>95%) *BRAF* mutation observed in CRC patients is *BRAF* V600E, which leads to constitutive activation of BRAF kinase and sustained RAS/RAF/MEK/ERK pathway signaling, resulting in increased cell proliferation and survival.[17] In mCRC, approximately 5% of patients are MSI-H, compared to 15% in primary CRC.[17, 48] Approximately 15-30% of *BRAF*-mutant mCRC tumors were also identified as MSI-H/dMMR.[16, 17, 24, 25] The presence of a *BRAF* V600 mutation appears to be a poor prognostic factor independent of MSI status in mCRC,[16, 17] which differs from the MSS-dependent poor prognostic value of *BRAF* V600 in primary CRC.[49] In this study, MSI status will be confirmed by retrospective central testing in tumor tissue as a secondary endpoint.

Detection of *BRAF* V600E in ctDNA is concordant with tumor tissue in CRC patients.[50] A decrease in *BRAF* V600E ctDNA levels predicted improved radiographic responses to vemurafenib + cetuximab + irinotecan[51] and was significantly greater in responders compared to non-responders in *BRAF* mutant CRC patients treated with dabrafenib + panitumumab ± trametinib.[52] Correlating *BRAF* V600E VAF and overall mean VAF from plasma samples collected at baseline and on treatment will be a secondary endpoint in this study.

Unless prohibited by local regulations or ethics committee decision, additional blood samples will be collected for various exploratory biomarker analyses as described in Section 8. Banked Biospecimens will be collected and stored for further analyses which may, for example, provide greater understanding of the study intervention.



090177e194745c57\Approved\Approved On: 22-Jul-2020 00:48 (GMT)

### 4.2.6. Use of Contraceptives

The study interventions being administered are known to cause risk for severe manifestations of developmental toxicity in humans or suspected on the basis of the intended pharmacology. Therefore, the use of a highly effective method of contraception is required (see Appendix 4).

## 4.3. Justification for Dose

In order to optimize the potential for benefit, while limiting the potential for toxicity, the starting encorafenib dose in the current study is 300 mg QD, corresponding to its single-agent RP2D. This dose of encorafenib was administered in combination with cetuximab in Study ARRAY-818-302 (BEACON CRC) and was shown to be both effective and tolerable in participants with previously treated *BRAF* V600E-mutant mCRC. Based on these results, the approved dose of encorafenib for this indication is 300 mg QD when given in combination with cetuximab.[30] Further details are available in the encorafenib SRSD.

The dose regimens selected for capecitabine, 5-FU, irinotecan, leucovorin, oxaliplatin, and bevacizumab are consistent with the respective SRSDs.

The cetuximab regimen approved globally is 400 $mg/m^2$ first-dose followed by 250 $mg/m^2$ IV weekly. Because many of the cytotoxic doublets used in mCRC are administered biweekly, a biweekly cetuximab regimen has also been tested. In a Phase 1 dose-escalation study in patients with mCRC, doses of cetuximab 400 to 700 $mg/m^2$ every 2 weeks were evaluated. The MTD was not identified and 500 $mg/m^2$ was identified as the dose for further development based on its PK parameters being most similar to the weekly regimen.[53] A Phase 2 study in patients with previously treated mCRC treated with biweekly cetuximab 500 $mg/m^2$ in combination with irinotecan demonstrated similar efficacy and tolerability as historical data with similar regimens using weekly cetuximab.[54] The use of the biweekly regimen is fairly common in current practice and is included as an acceptable regimen in the NCCN guidelines.[28] Allowing use of this regimen will provide greater flexibility and perhaps enhance compliance and recruitment within the study in which participants are expected to remain on treatment for approximately 10 months.

## 4.4. End of Study Definition

End of study will be defined as the point when all participants have been followed for at least 1 year after the randomization date of the last participant enrolled in the Phase 3 portion of the study or at least 80% of participants have an OS event (or are lost to follow-up), whichever occurs later. Any participants still receiving study intervention at the end of the study will be allowed to continue at the discretion of the Investigator and Sponsor and as long as none of the study intervention discontinuation criteria are met (see Section 7.1).

# 5. STUDY POPULATION

This study can fulfill its objectives only if appropriate participants are enrolled. The following eligibility criteria are designed to select participants for whom participation in the study is considered appropriate. All relevant medical and nonmedical conditions should be



090177e194745c57\Approved\Approved On: 22-Jul-2020 00:48 (GMT)

taken into consideration when deciding whether a particular participant is suitable for this protocol.

Prospective approval of protocol deviations to recruitment and enrollment criteria, also known as protocol waivers or exemptions, is not permitted.

Pfizer or designee will review select eligibility criteria verified by the investigator or qualified designee to confirm that participants meet study eligibility criteria before they are enrolled into the study. The enrollment approval process will be initiated for a participant after an informed consent/assent document has been signed and the investigator or qualified designee has assessed the participant as eligible. The enrollment approval will be based on review of CRF/system data.

## 5.1. Inclusion Criteria

Participants are eligible to be included in the study only if all of the following criteria apply:

### 5.1.1. Molecular Prescreening Inclusion Criteria

**Age and Sex:**

1. **SLI**: Male or female participants age ≥18 years at the time of informed consent.
   **Phase 3**: Male or female participants age ≥16 years at the time of informed consent/assent.

   Refer to Appendix 4 for reproductive criteria for male (Section 10.4.1) and female (Section 10.4.2) participants.

**Weight:**

2. Body weight ≥40 kg.

**Type of Participant and Disease Characteristics:**

3. Participants with histologically or cytologically confirmed colorectal adenocarcinoma.
4. Participants with evidence of metastatic disease.
5. Able to provide a sufficient amount of representative tumor specimen for central testing of *BRAF* V600E mutation status.
   ***Note***: Tumor sample must be in an FFPE block, newly collected fixed biopsy sample, or a minimum of 15 unstained slides of analyzable tissue. Participants with fewer than 15 slides of analyzable tissue may be considered eligible if the Sponsor determines that the slides are sufficient for central testing of *BRAF* V600E mutation status.

**Informed Consent/Assent:**

6. Capable of giving signed informed consent/assent as described in Appendix 1, which includes compliance with the requirements and restrictions listed in the ICD and in this protocol.
   ***Note***: Participants ≥16 years old that are under guardianship may participate with the consent of their legally authorized guardian if permitted by local regulations. When



090177e194745c57\Approved\Approved On: 22-Jul-2020 00:48 (GMT)

appropriate, adolescent participants will be included in all discussions (see Section 10.1.3).

### 5.1.2. Screening Inclusion Criteria

**Type of Participant and Disease Characteristics:**

7. Participants who have met all Molecular Prescreening inclusion criteria.
8. Participants who are willing and able to comply with all scheduled visits, treatment plan, laboratory tests, lifestyle considerations, and other study procedures.
9. Presence of a *BRAF* V600E mutation in tumor tissue or blood (eg, ctDNA genetic testing). The following are acceptable:
   a. Local laboratory assay (PCR or NGS-based only) performed at any time prior to Screening using either tumor tissue or blood.
   b. Central laboratory assay performed during the Screening period using tumor tissue alone (not blood).

   ***Note***: For participants enrolled on the basis of a local *BRAF* mutation assay, tumor samples must be submitted to the central laboratory for *BRAF* testing as soon as possible following signing of the ICD. The *BRAF* status must be confirmed no later than 30 days following first dose of study intervention.
10. The Investigator must obtain prior to Cycle 1 Day 1 (SLI) or date of randomization (Phase 3) adequate tumor tissue (primary or metastatic, archival or newly obtained) for submission to a central laboratory for confirmation of *BRAF* V600E (FFPE block or minimum of 15 unstained slides containing recently cut, 4-5 µm sections on positively charged slides). Participants with fewer than 15 slides of analyzable tissue may be considered eligible if the Sponsor determines that the slides are sufficient for central testing of *BRAF* V600E mutation status.
    ***Note***: Once *BRAF* V600E mutation status is determined by the central laboratory (tumor tissue), the results will be considered definitive for eligibility. No repeat testing will be performed.
    ***Note***: Lack of *BRAF* V600E confirmation by the central laboratory may be due to discordance between the local assay and central laboratory results (potential false positive local assay results), or due to inadequate or poor sample condition for central testing (indeterminate results). If at any time in the study there is lack of *BRAF* V600E confirmation in a total of 6% of the total planned enrollment of the randomized portion of the trial (52 participants) or a discordance between the local assay and the central laboratory of 3% of the total planned enrollment (26 participants), all subsequent participants will be required to have *BRAF* V600E determined by the central laboratory for treatment (ie, local *BRAF* testing will no longer be accepted for trial eligibility).
    ***Note***: Participants whose sample is determined to be inadequate or who have an indeterminate result on central testing may have additional tumor samples submitted for testing.

090177e194745c57\Approved\Approved On: 22-Jul-2020 00:48 (GMT)

11. Participants who have received ≤1 (SLI) or no (Phase 3) prior systemic regimens for metastatic disease.
    ***Note***: SLI participants who have received prior oxaliplatin-based regimens in first-line treatment will be enrolled to the FOLFIRI combination cohort (Cohort 1) and participants who have received prior irinotecan in first-line treatment will be enrolled in the mFOLFOX6 combination cohort (Cohort 2).
    ***Note***: Participants with early stage disease (eg, Stages I-III) treated with surgery followed by chemotherapy (eg, treatment in the adjuvant setting) who present with new lesions or evidence of disease recurrence during or within 6 months of the last dose of chemotherapy would be considered as having received 1 prior systemic therapy in the metastatic setting.
    ***Note***: Participants who have received prior systemic neoadjuvant therapy with or without radiation, and present with new lesions or evidence of disease recurrence, during or within 6 months of the last dose of therapy would be considered as receiving first-line therapy in the metastatic setting.
12. ECOG performance status of 0 or 1.
13. Measurable disease in the Phase 3 portion of the study and measurable or non-measurable but evaluable disease per RECIST, v1.1 in the SLI, as assessed by Investigator and evidenced by available baseline scans.
    ***Note***: Baseline scan is defined as the last scan prior to the date of first dose (SLI) or the date of randomization (Phase 3) (Section 9.4.1)
    ***Note***: Baseline scans will be required to be submitted to a central radiology vendor to be assessed by the BICR.
14. Adequate bone marrow function characterized by the following at screening:
    a. ANC ≥1.5 × $10^9$/L;
    b. Platelets ≥100 × $10^9$/L;
    c. Hemoglobin ≥9.0 g/dL (with or without blood transfusions).
15. Adequate hepatic and renal function characterized by the following at screening:
    a. Serum total bilirubin ≤1.5 x ULN and <2 mg/dL.
       ***Note***: Total bilirubin >1.5 x ULN is allowed if direct (conjugated) ≤1.5 x ULN and indirect (unconjugated) bilirubin is ≤4.25 x ULN.
       ***Note***: Participants with hyperbilirubinemia due to non-hepatic cause (eg, hemolysis, hematoma) may be enrolled following discussion and agreement with the medical monitor.
    b. ALT and AST ≤2.5 × ULN, or ≤5 × ULN in the presence of liver metastases.
    c. Adequate renal function defined by an estimated creatinine clearance ≥50 mL/min according to the Cockcroft Gault formula or by 24-hour urine collection for creatinine clearance, or according to local institutional standard method.

090177e194745c57\Approved\Approved On: 22-Jul-2020 00:48 (GMT)

d. Adequate electrolytes, defined as serum potassium and magnesium levels within institutional normal limits.
   ***Note***: Replacement treatment to achieve adequate electrolytes will be allowed.

16. Able to swallow, retain, and absorb oral medications.

## 5.2. Exclusion Criteria

Participants are excluded from the study if any of the following criteria apply:

### 5.2.1. Molecular Prescreening Exclusion Criteria

**Medical Conditions:**

1. Other medical or psychiatric condition including recent (within the past year) or active suicidal ideation/behavior or laboratory abnormality that may increase the risk of study participation or, in the investigator's judgment, make the participant inappropriate for the study.
2. Known history of acute or chronic pancreatitis.
3. Leptomeningeal disease.
4. History of chronic inflammatory bowel disease requiring medical intervention (immunomodulatory or immunosuppressive medications or surgery) ≤12 months prior to randomization.
5. Known DPD deficiency.
6. Gilbert's syndrome or known homozygous UGT1A1*28/*28 or UGT1A1*6/*6 genotypes or double heterozygous UGT1A1*6/*28 genotype:
   a. **SLI**: Participants with documented Gilbert's syndrome or known homozygous UGT1A1*28/*28 or UGT1A1*6/*6 genotypes or double heterozygous UGT1A1*6/*28 genotype will be excluded from Cohort 1 (EC + FOLFIRI) of the SLI.
   b. **Phase 3**: If the decision is made to include FOLFIRI in Arm B, all participants with documented Gilbert's syndrome or known homozygous UGT1A1*28/*28 or UGT1A1*6/*6 genotypes or double heterozygous UGT1A1*6/*28 genotype will be excluded from the Phase 3 portion.
      If the decision is made to include mFOLFOX6 in Arm B, participants with documented Gilbert's syndrome or known homozygous UGT1A1*28/*28 or UGT1A1*6/*6 genotypes or double heterozygous UGT1A1*6/*28 genotype may be enrolled, but may not receive FOLFOXIRI if randomized to the Control Arm.

**Other Exclusions:**

7. Investigator site staff or Pfizer employees directly involved in the conduct of the study, site staff otherwise supervised by the investigator, and their respective family members.
8. Known RAS-mutant colorectal adenocarcinoma.



090177e194745c57\Approved\Approved On: 22-Jul-2020 00:48 (GMT)

9. Locally confirmed dMMR or MSI-H colorectal carcinoma unless participant is unable to receive immune checkpoint inhibitors due to a pre-existing medical condition.

### 5.2.2. Screening Exclusion Criteria

**Medical Conditions:**

10. Impaired gastrointestinal function (eg, uncontrolled nausea, vomiting or diarrhea, malabsorption syndrome, small bowel resection) or disease which may significantly alter the absorption of oral study intervention or recent changes in bowel function suggesting current or impending bowel obstruction.
11. Clinically significant cardiovascular diseases, including any of the following:
    a. History of acute myocardial infarction, acute coronary syndromes (including unstable angina, coronary artery bypass graft, coronary angioplasty or stenting) ≤6 months prior to randomization;
    b. Congestive heart failure requiring treatment (New York Heart Association Grade ≥2);
    c. History or presence of clinically significant cardiac arrhythmias (including uncontrolled atrial fibrillation or uncontrolled paroxysmal supraventricular tachycardia);
    d. History of thromboembolic or cerebrovascular events ≤12 weeks prior to randomization. Examples include transient ischemic attacks, cerebrovascular accidents, hemodynamically significant (ie, massive or sub-massive) deep vein thrombosis or pulmonary emboli.
    ***Note***: Participants with either deep vein thrombosis or pulmonary emboli that do not result in hemodynamic instability are allowed to enroll as long as they are on a stable dose of anticoagulants for at least 4 weeks.
    ***Note***: Participants with thromboembolic events related to indwelling catheters (including PICC lines) or other procedures may be enrolled.
    e. Triplicate average QTcF interval ≥480 ms or a history of prolonged QT syndrome.
    f. Congenital LQTS.
12. Evidence of active noninfectious pneumonitis.
13. Evidence of active and uncontrolled bacterial or viral infection, with certain exceptions, as noted below, for chronic infection with HIV, hepatitis B or hepatitis C (please see below), within 2 weeks prior to start of study intervention.
14. Participants positive for HIV are ineligible unless they meet all of the following:
    a. A stable regimen of highly active anti-retroviral therapy that is not contraindicated (see Section 6.5);
    b. No requirement for concurrent antibiotics or antifungal agents for the prevention of opportunistic infections;

090177e194745c57\Approved\Approved On: 22-Jul-2020 00:48 (GMT)

   c. A CD4 count >250 cells/mcL, and an undetectable HIV viral load on standard PCR-based tests.

15. Active hepatitis B or hepatitis C infection

   a. Active HBV is defined as any of the following:

      - HBsAg(+), HBV DNA >200 IU/mL (105 copies/mL);
      - HBsAg(+), HBV DNA ≤200 IU/mL and persistent or intermittent elevation of ALT/AST and/or liver biopsy showing chronic hepatitis with moderate or severe necroinflammation.

      ***Note***: Participants who are HBsAg(-), HBcAb(+) are eligible and should be monitored/treated as per local standard of care.

   b. Active HCV is defined as:

      - HCV antibody positive; AND
      - Presence of HCV RNA.

16. Concurrent or previous other malignancy within 2 years of study entry, except curatively treated basal or squamous cell skin cancer, prostate intraepithelial neoplasm, carcinoma in-situ of the cervix, Bowen's disease or prostate cancer with a Gleason score ≤6. Participants with other curatively treated malignancies with low risk of recurrence not listed may also be considered eligible after review and approval by the medical monitor.

17. Residual CTCAE ≥Grade 2 toxicity from any prior anticancer therapy, with the exception of Grade 2 alopecia or Grade 2 neuropathy.

**Prior/Concomitant Therapy:**

18. **SLI**: Prior systemic regimen containing both oxaliplatin and irinotecan (eg, FOLFOXIRI).

19. Previous treatment with any selective BRAF inhibitor (eg, encorafenib, dabrafenib, vemurafenib, XL281/BMS-908662) or any EGFR inhibitor (eg, cetuximab, panitumumab) prior to screening.

20. Use of any prohibited medication (including herbal medication), supplement or food that is a moderate or strong inhibitor or inducer of CYP3A4/5 ≤1 week prior to the start of study intervention (Section 6.5.1.2).

21. Major surgery (eg, inpatient procedure with regional or general anesthesia) or completion of radiation therapy ≤4 weeks prior to randomization.

**Prior/Concurrent Clinical Study Experience:**

22. Previous administration with an investigational drug within 30 days (or as determined by the local requirement) or 5 half-lives preceding the first dose of study intervention used in this study (whichever is longer).



090177e194745c57\Approved\Approved On: 22-Jul-2020 00:48 (GMT)

**Other Exclusions:**

23. Has met any of the Molecular Prescreening exclusion criteria.
24. Has documented clinical disease progression (eg, worsening of performance status, clinical symptoms, or clinically significant laboratory parameters demonstrating worsening of disease) or radiographic disease progression during the Screening period.
25. Symptomatic brain metastasis.
 ***Note***: Participants previously treated or untreated for this condition who are asymptomatic in the absence of corticosteroid and anti-epileptic therapy are allowed. Brain metastases must be stable for ≥4 weeks prior to randomization.
26. Known contraindication to receiving cetuximab including hypersensitivity or toxicity that would suggest an inability to tolerate maximum cetuximab dose of 500 $mg/m^2$.
27. Known sensitivity or contraindication to any component of study intervention or their excipients at the planned doses.
28. Known contraindication to receive any component of the Control Arm at the planned doses; refer to the most recent local label, as applicable.
29. Pregnant, confirmed by a positive β-hCG laboratory test result, or is breastfeeding (lactating).

## 5.3. Lifestyle Considerations

### 5.3.1. Contraception

The investigator or his or her designee, in consultation with the participant, will confirm that the participant has selected an appropriate method of contraception for the individual participant and his or her partner(s) from the permitted list of contraception methods (see Appendix 4 Section 10.4.4) and will confirm that the participant has been instructed in its consistent and correct use. At time points indicated in the SoA, the investigator or designee will inform the participant of the need to use highly effective contraception consistently and correctly and document the conversation and the participant's affirmation in the participant's chart (participants need to affirm their consistent and correct use of at least 1 of the selected methods of contraception). In addition, the investigator or designee will instruct the participant to call immediately if the selected contraception method is discontinued or if pregnancy is known or suspected in the participant or partner.

### 5.3.2. Meals and Dietary Restrictions

Participants treated in the SLI, Arm A and Arm B must avoid consumption of grapefruit, pomegranates, star fruits, Seville oranges or products containing the juice of each during the entire study and preferably 7 days before the first dose of study intervention, due to potential CYP3A4 interaction with encorafenib (see Section 6.5). Orange juice is allowed.

## 5.4. Screen Failures

Screen failures are defined as participants who consent/assent to participate in the clinical study but are not subsequently enrolled in the study. A minimal set of screen failure



090177e194745c57\Approved\Approved On: 22-Jul-2020 00:48 (GMT)

information is required to ensure transparent reporting of screen failure participants to meet the CONSORT publishing requirements and to respond to queries from regulatory authorities. Minimal information includes demography, screen failure details, eligibility criteria, and any SAEs. For Molecular Prescreening failures, results of local *BRAF* assay available at Molecular Prescreening or Screening, AEs or SAEs possibly related to a study procedure will be reported.

Tests with results that fail eligibility requirements may be repeated during Screening if the Investigator believes the result to be in error. Additionally, a participant who fails Screening (screen failure) may repeat the Screening process 1 time if the Investigator believes that there has been a change in eligibility status.

# 6. STUDY INTERVENTION

Study intervention is defined as any investigational intervention(s), marketed product(s), placebo, medical device(s), or study procedure(s) intended to be administered to a study participant according to the study protocol.

For the purposes of this protocol, study intervention refers to capecitabine, cetuximab, encorafenib, 5-FU, irinotecan, leucovorin, oxaliplatin, and bevacizumab.

090177e194745c57\Approved\Approved On: 22-Jul-2020 00:48 (GMT)

090177e194745c57\Approved\Approved On: 22-Jul-2020 00:48 (GMT)

## 6.1. Study Interventions Administered

| **Intervention Name** | **Encorafenib** | **Cetuximab** | **Fluorouracil** | **Leucovorin[a]** | **Irinotecan** | **Oxaliplatin** | **Capecitabine** | **Bevacizumab** |
|---|---|---|---|---|---|---|---|---|
| **Treatment Arm** | SLI; Phase 3, Arm A and Arm B | SLI; Phase 3, Arm A and Arm B | SLI; Phase 3, Arm B; Phase 3, Control Arm | SLI; Phase 3, Arm B; Phase 3, Control Arm | SLI; Phase 3, Arm B[b]; Phase 3, Control Arm | SLI; Phase 3, Arm B[b]; Phase 3, Control Arm | Phase 3, Control Arm | Phase 3, Control Arm |
| **Type** | Drug | Drug | Drug | Drug | Drug | Drug | Drug | Drug |
| **Dose Formulation** | Capsule | Injection for intravenous use | Injection for intravenous use | Injection | Solution for intravenous infusion | Powder for solution for intravenous use | Tablet | Injection for intravenous use |
| **Unit Dose Strength(s)** | 75 mg | 100 mg/vial, 200 mg/vial, or 500 mg/vial | 250 mg/vial, 500 mg/vial, or 1000 mg/vial | 50 mg/vial, 100 mg/vial, 200 mg/vial, or 350 mg/vial | 40 mg/vial, 100 mg/vial, or 300 mg/vial | 50 mg/vial, 100 mg/vial, or 200 mg/vial | 150 mg or 500 mg | 100 mg/vial or 400 mg/vial |
| **Route of Administration** | Oral | Intravenous | Intravenous | Intravenous | Intravenous | Intravenous | Oral | Intravenous |
| **Use** | Experimental | Experimental | Experimental (Arms A and B); Comparator (Control Arm) | Experimental (Arms A and B); Comparator (Control Arm) | Experimental (Arms A and B); Comparator (Control Arm) | Experimental (Arms A and B); Comparator (Control Arm) | Comparator | Comparator |
| **IMP or NIMP** | IMP | IMP | IMP | IMP | IMP | IMP | IMP | IMP |
| **Sourcing** | Provided centrally by the sponsor. | Provided centrally by the sponsor or locally by the trial site. | Provided centrally by the sponsor or locally by the trial site. | Provided centrally by the sponsor or locally by the trial site. | Provided centrally by the sponsor or locally by the trial site. | Provided centrally by the sponsor or locally by the trial site. | Provided centrally by the sponsor or locally by the trial site. | Provided centrally by the sponsor or locally by the trial site. |
| **Packaging and Labeling** | Study intervention will be provided in bottles. Each bottle will be open labeled as required per country requirement. | Study intervention will be provided in vials. Each vial will be open labeled as required per country requirement. | Study intervention will be provided in vials. Each vial will be open labeled as required per country requirement. | Study intervention will be provided in vials. Each vial will be open labeled as required per country requirement. | Study intervention will be provided in vials. Each vial will be open labeled as required per country requirement. | Study intervention will be provided in vials. Each vial will be open labeled as required per country requirement. | Study intervention will be provided in bottles. Each bottle will be open labeled as required per country requirement. | Study intervention will be provided in vials. Each vial will be open labeled as required per country requirement. |

a Alternatively, 200 mg/m$^2$ levoleucovorin may be used.
b Use of either irinotecan or oxaliplatin in Arm B will depend on the results of the SLI (see Section 4.1).

### 6.1.1. Safety Lead-in

Since the EC regimen has not previously been combined with cytotoxic chemotherapy, the study includes an SLI, to be conducted at a limited number of sites, to evaluate the safety/tolerability and PK of EC + mFOLFOX6 and EC + FOLFIRI in up to 30 participants per cohort. Participants will be enrolled on a rolling basis in an alternating manner to each cohort when possible based on eligibility criteria.

The tolerability assessment will be based on occurrence of DLTs as defined in Section 6.1.1.1. Tolerability will be reviewed by the Sponsor and investigators in approximately weekly telephone conferences during the SLI. If 3 of the first 9 participants are determined to have experienced DLTs in a given cohort, enrollment to that cohort will be put on hold until a discussion with the SC and E-DMC can occur. If the DLTs are confirmed by the SC and the E-DMC and it is determined that the regimen is not tolerable, dosing in that cohort will be suspended.

The SC will review safety data after the 9$^{th}$ evaluable participant in a particular cohort has been followed for at least one 28-day cycle. In either cohort, if the doses are determined to be tolerable by the SC in the first 9 evaluable participants based on an observed rate of DLTs in <33% of participants and evaluation of the overall toxicity profile, the SLI will be expanded for that particular cohort by up to a maximum of approximately 30 participants. Enrollment may continue while the SC reviews safety data from the first 9 participants. The E-DMC will also review data after the 9$^{th}$ participant in a particular cohort has been followed for at least one 28-day cycle to confirm tolerability of the doses.

At the completion of the SLI when all evaluable participants have been followed for a minimum of one 28-day cycle, the SC and the E-DMC will review all accumulated safety data to confirm that the doses are acceptable for use in Arm B (EC + mFOLFOX6 or EC + FOLFIRI) of the randomized Phase 3 portion of the study. The primary criteria in deciding which combination to use in Phase 3 will be based on identifying the combination with the fewest DLTs. If the DLT rate is ≥33% in either cohort, that regimen will not be used in Phase 3. If the DLT rate is <33% in both cohorts, additional parameters that may be used to determine the most suitable combination will include dose modifications, AEs of at least Grade 3, AEs leading to treatment discontinuations, SAEs, and PK results including the potential for DDIs. Since only 1 of the combination regimens will be included in the Phase 3 portion of the study, a rigorous selection process will be implemented based on the totality of the data. Finally, although efficacy is not a primary objective, the number of objective responses and DORs may also be taken into consideration in making a final decision.

Although the SLI is planned to enroll up to 30 evaluable participants per cohort, the decision to proceed to Phase 3 may occur earlier if one of the combination regimens is clearly superior based on the collective safety/tolerability and PK. If there are no clearly distinguishing differences between the 2 combinations, the most appropriate regimen to be used in Phase 3 will be determined following a review of the totality of the SLI data. If neither regimen is considered tolerable and/or suitable for evaluation in the randomization portion of the trial, the study may proceed as a 2-arm study of EC versus SOC chemotherapy. The decision to initiate Phase 3 will require approval from the study SC and the E-DMC.



090177e194745c57\Approved\Approved On: 22-Jul-2020 00:48 (GMT)

090177e194745c57\Approved\Approved On: 22-Jul-2020 00:48 (GMT)

#### 6.1.1.1. Dose Limiting Toxicity Definition

For purposes of tolerability decisions, a DLT is defined as any AE or abnormal laboratory value assessed as unrelated to disease, disease progression, intercurrent illness or concomitant medications/therapies occurring during the first 28 days of treatment that meets at least 1 of the criteria listed in Table 9. A participant is classified as DLT-evaluable if he/she experiences a DLT or in the absence of a DLT, receives at least 75 % of the planned dose intensity of each study intervention during the DLT window (see Section 9.3). If a participant does not meet these criteria, he/she may be replaced.

Adverse events that are clinically significant and considered to be related to the study intervention that occur after the DLT observation period will be reviewed in context of all safety data available. That review may result in re-evaluation of the dosing level or regimen.

Severity of AEs will be graded according to CTCAE v4.03. In Cohort 1 (EC + FOLFIRI), any DLT occurring prior to initiation of encorafenib on Day 3 will not be considered for tolerability decisions.

**Table 9. DLT Criteria**

| |
|---|
| • Any AE or laboratory value considered unrelated to underlying disease, disease progression, intercurrent illness or concomitant medications/therapies resulting in the inability to tolerate at least 75% of the planned dose intensity ([administered dose in mg/planned dose in mg] x 100) of each study intervention during the 28-day DLT period. |
| **General disorders and administration site conditions**<br>• Fatigue Grade 3 for >14 consecutive days |
| **Respiratory disorders**<br>• Interstitial lung disease/pneumonitis Grade ≥2 |
| **Skin and subcutaneous tissue disorders**[a]<br>• Rash, HFSR, or photosensitivity Grade 3 for >14 consecutive days despite maximal skin toxicity treatment (as per local practice)<br>• Rash, HFSR, or photosensitivity Grade 4 |
| **Gastrointestinal disorders**[a]<br>• Diarrhea Grade 3 for ≥48 hours despite optimal use of antidiarrheal therapy<br>• Diarrhea Grade 4<br>• Nausea/vomiting Grade 3 for ≥48 hours despite optimal use of antiemetic therapy<br>• Nausea/vomiting Grade 4<br>• Mucositis Grade ≥3 |

090177e194745c57\Approved\Approved On: 22-Jul-2020 00:48 (GMT)

## Table 9. DLT Criteria

**Investigations**

- Total bilirubin Grade ≥3
- AST or ALT Grade ≥3 in conjunction with total bilirubin Grade ≥2 of any duration
- AST or ALT Grade 3 for >7 consecutive days
- AST or ALT Grade 4
- Serum creatinine Grade ≥3
- ANC Grade 4 for >7 consecutive days
- Febrile neutropenia (ANC <1000 and T ≥38.5°C)
- Platelet count Grade 3 with signs of clinically significant bleeding
- Platelet count Grade 4
- ECG QTcF prolonged ≥Grade 3[b]

**Uveitis**

- Grade ≥3 for >21 consecutive days
- Grade 4 confirmed by ophthalmic examination

**Neurotoxicity**

- Paresthesia/dysesthesias Grade ≥3

**Other hematologic and nonhematologic toxicities[c]**

- Any other Grade ≥3 AE except:
    - Lymphocyte count decreased (lymphopenia) Grade ≥3 unless clinically significant

a. Prophylactic treatment for nausea/vomiting or skin AEs should be used at the discretion of the Investigator if the participant experiences nausea/vomiting and/or skin AEs Grade ≥1.
b. QTcF must be prolonged on two separate ECGs at least 5 minutes apart.
c. Isolated laboratory changes (eg alkaline phosphatase, cholesterol, lipase, serum amylase) or those due to sampling or laboratory errors without associated clinical signs or symptoms may be determined to not be DLTs upon review and agreement by the Investigator and medical monitor.

### 6.1.2. Treatment Regimens

Participants enrolled in the SLI will receive either a regimen of EC + mFOLFOX6 or a regimen of EC + FOLFIRI in 28 day cycles.

**Table 10. Treatment Regimens (Safety Lead-in)**

| Study Treatments | Dose | Frequency |
|---|---|---|
| Encorafenib | 300 mg (4 × 75 mg) oral capsule | QD |
| Cetuximab | 500 $mg/m^2$ (120-minute IV infusion) | Q2W |
| mFOLFOX6 | Oxaliplatin 85 $mg/m^2$ (120-minute IV infusion)<br>Leucovorin 400 $mg/m^2$ (120-minute IV infusion)[a,b]<br>5-FU 400 $mg/m^2$ IV bolus, then 5-FU 2400 $mg/m^2$ continuous IV infusion over 46-48 hours | Q2W |
| Encorafenib | 300 mg (4 × 75 mg) oral capsule[c] | QD |
| Cetuximab | 500 $mg/m^2$ (120-minute IV infusion) | Q2W |
| FOLFIRI | Irinotecan 180 $mg/m^2$ (90-minute IV infusion)<br>Leucovorin 400 $mg/m^2$ (120-minute IV infusion) [a,b]<br>5-FU 400 $mg/m^2$ IV bolus, then 5-FU 2400 $mg/m^2$ continuous IV infusion over 46-48 hours | Q2W |

a Alternatively, 200 $mg/m^2$ levoleucovorin (120-minute IV infusion) may be administered

b Leucovorin may be co-administered with the oxaliplatin or irinotecan infusion

c Encorafenib dosing will not begin until Cycle 1 Day 3 for participants receiving FOLFIRI

Participants randomized to Arm A will receive encorafenib + cetuximab in 28 day cycles.

**Table 11. Treatment Regimen (Phase 3, Arm A)**

| Study Treatments | Dose | Frequency |
|---|---|---|
| Encorafenib | 300 mg (4 × 75 mg) oral capsule | QD |
| Cetuximab | 500 $mg/m^2$ (120-minute IV infusion) | Q2W |

***Note***: In the Phase 3 portion of the study, participants must receive their first dose of study intervention within 5 days of randomization.

090177e194745c57\Approved\Approved On: 22-Jul-2020 00:48 (GMT)

Participants randomized to Arm B will receive either a regimen of EC + mFOLFOX6 or a regimen of EC + FOLFIRI in 28-day cycles. The use of mFOLFOX6 or FOLFIRI in Arm B will depend on the results of the SLI portion of the study (see Section 6.1.1).

**Table 12. Treatment Regimens (Phase 3, Arm B)**

| Study Treatments | Dose | Frequency |
|---|---|---|
| Encorafenib | 300 mg (4 × 75 mg) oral capsule | QD |
| Cetuximab | 500 mg/m$^2$ (120-minute IV infusion) | Q2W |
| mFOLFOX6 | Oxaliplatin 85 mg/m$^2$ (120-minute IV infusion)<br>Leucovorin 400 mg/m$^2$ (120-minute IV infusion)[a,b]<br>5-FU 400 mg/m$^2$ IV bolus, then 5-FU 2400 mg/m$^2$ continuous IV infusion over 46-48 hours | Q2W |
| Encorafenib | 300 mg (4 × 75 mg) oral capsule[c] | QD |
| Cetuximab | 500 mg/m$^2$ (120-minute IV infusion) | Q2W |
| FOLFIRI | Irinotecan 180 mg/m$^2$ (90-minute IV infusion)<br>Leucovorin 400 mg/m$^2$ (120-minute IV infusion)[a,b]<br>5-FU 400 mg/m$^2$ IV bolus, then 5-FU 2400 mg/m$^2$ continuous IV infusion over 46-48 hours | Q2W |

a Alternatively, 200 mg/m$^2$ levoleucovorin (120-minute IV infusion) may be administered

b Leucovorin may be co-administered with the oxaliplatin or irinotecan infusion

c Encorafenib dosing will not begin until Cycle 1 Day 3 for participants receiving FOLFIRI

***Note***: In the Phase 3 portion of the study, participants must receive their first dose of study intervention within 5 days of randomization.

090177e194745c57\Approved\Approved On: 22-Jul-2020 00:48 (GMT)

Participants randomized to the Control Arm will receive one of the following per Investigator choice:

**Table 13. Treatment Regimens (Phase 3, Control Arm [Arm C])**

| Study Treatments | Dose | Frequency |
|---|---|---|
| mFOLFOX6 ± Bevacizumab | Oxaliplatin 85 mg/m$^2$ (120-minute IV infusion)<br>Leucovorin 400 mg/m$^2$ (120-minute IV infusion)[a,b]<br>5-FU 400 mg/m$^2$ IV bolus, then 5-FU 2400 mg/m$^2$ continuous IV infusion over 46-48 hours<br>Bevacizumab (optional; given per prescribing instructions) | Q2W 28-day Cycle |
| FOLFOXIRI ± Bevacizumab | Irinotecan 165 mg/m$^2$ (90-minute IV infusion)<br>Oxaliplatin 85 mg/m$^2$ (120-minute IV infusion)<br>Leucovorin 400 mg/m$^2$ (120-minute IV infusion)[a,b]<br>5-FU 2400 or 3200 mg/m$^2$ continuous IV infusion over 46-48 hours<br>Bevacizumab (optional; given per prescribing instructions) | Q2W 28-day Cycle |
| FOLFIRI ± Bevacizumab | Irinotecan 180 mg/m$^2$ (90-minute IV infusion)<br>Leucovorin 400 mg/m$^2$ (120-minute IV infusion)[a,b]<br>5-FU 400 mg/m$^2$ IV bolus, then 5-FU 2400 mg/m$^2$ continuous IV infusion over 46-48 hours<br>Bevacizumab (optional; given per prescribing instructions) | Q2W 28-day Cycle |
| CAPOX ± Bevacizumab | Oxaliplatin 130 mg/m$^2$ (120-minute IV infusion)<br>Capecitabine 1000 mg/m$^2$ oral tablet<br>Bevacizumab (optional; given per prescribing instructions) | Q3W 21-day Cycle<br>BID Days 1-14 |

a Alternatively, 200 mg/m$^2$ levoleucovorin (120-minute IV infusion) may be administered

b Leucovorin may be co-administered with the oxaliplatin or irinotecan infusion

***Note***: If mFOLFOX6 is used in Arm B, the Control Arm will consist of Investigator's choice of mFOLFOX6 ± bevacizumab, FOLFOXIRI ± bevacizumab or CAPOX ± bevacizumab. If FOLFIRI is used in Arm B, the Control Arm will consist of Investigator's choice of FOLFIRI ± bevacizumab or FOLFOXIRI ± bevacizumab. If Phase 3 proceeds without Arm B, the Control Arm will consist of Investigator's choice of mFOLFOX6 ± bevacizumab, FOLFIRI ± bevacizumab, FOLFOXIRI ± bevacizumab or CAPOX ± bevacizumab.

***Note***: The decision of which regimen to use in the Control Arm is at the participant/treating Investigator's discretion but must be declared prior to randomization and the choice of regimen must not be changed during the course of the participant's treatment.

***Note***: Participants with colonic or rectal stent in place should not receive bevacizumab in any of the Control Arm regimens.

***Note***: In the Phase 3 portion of the study, participants must receive their first dose of study intervention within 5 days of randomization.

090177e194745c57\Approved\Approved On: 22-Jul-2020 00:48 (GMT)

### 6.1.3. Administration

#### 6.1.3.1. Administration of Encorafenib

Encorafenib will be administered on a QD schedule, PO as a flat-fixed dose, and not by body weight or BSA. Encorafenib should be taken without regard to food. Participants should be instructed to swallow the capsules whole and not to chew or crush them.

Participants should be instructed to take encorafenib capsules daily with a large glass of water (~250 mL) in the morning at approximately the same time every day. If a participant vomits at any time after dosing, the dose of study intervention should not be re-administered. Doses of encorafenib that are omitted for AEs or any other reason should not be made up during the day (ie, within the 12 hours of the next encorafenib dose), or at the end of the dosing period. Additional information regarding encorafenib administration can be found in the Pharmacy Manual. On days when a blood collection is scheduled at the investigational site, participants will take the morning dose of encorafenib at the site under the supervision of the Investigator or designee. On all other days, participants will take encorafenib at home.

Participants must avoid consumption of grapefruit, pomegranates, star fruits, Seville oranges or products containing the juice of each during the entire study and preferably 7 days before the first dose of study intervention, due to potential CYP3A4 interaction with the study interventions. Orange juice is allowed.

When EC is combined with FOLFIRI in the SLI and Phase 3 portions of the study, the first dose of encorafenib will be administered on Cycle 1 Day 3. In the SLI, encorafenib will be administered within 5 minutes before the start of the irinotecan infusion on the full PK day, Cycle 1 Day 15 of Cohort 1 (EC + FOLFIRI), or within 5 minutes before the start of oxaliplatin infusion on the full PK days, Cycle 1 Day 1 and Day 15 of Cohort 2 (EC + mFOLFOX6). On all other days, encorafenib will be administered with the start of irinotecan or oxaliplatin infusion.

For participants receiving EC + FOLFIRI, if encorafenib is interrupted for ≥4 consecutive days, in order to minimize the potential for drug-drug interactions, encorafenib should be restarted ≥2 days prior to the next planned irinotecan infusion or ≥2 days after the next irinotecan infusion. Irinotecan administration should not be delayed.

The pharmacist or study nurse will ensure that the appropriate dose is dispensed and will provide the participant with at least the appropriate number of encorafenib capsules for the number of doses to be taken prior to the next scheduled visit. The site personnel will train the participant and/or the participant's caregiver on dosing procedures for the study intervention.

Participants will receive a diary to document self-administered dosing of encorafenib and capecitabine in each cycle to include the dose of study intervention taken, the date of dosing (and times if applicable), and if any doses were missed and the reason for the missed dose. One diary will be provided per cycle. Participants will be instructed to return unused encorafenib, capecitabine and the participant diary to the site at the end of each cycle. Drug accountability must be performed on a regular basis.



090177e194745c57\Approved\Approved On: 22-Jul-2020 00:48 (GMT)

The Investigator or responsible site personnel should instruct the participant to take encorafenib as per protocol (promote compliance). The dosage prescribed and dispensed to the participant and all dose changes and all missed doses during the study must be recorded in the eCRF.

#### 6.1.3.2. Administration of Capecitabine, Cetuximab, 5-Fluorouracil, Irinotecan, Leucovorin, Oxaliplatin and Bevacizumab

Capecitabine, cetuximab, 5-FU, irinotecan, leucovorin, oxaliplatin and bevacizumab are to be administered in accordance with their respective SRSDs. For detailed dosing information, see Section 6.1.2.

### 6.2. Preparation/Handling/Storage/Accountability

1. The investigator or designee must confirm appropriate temperature conditions have been maintained during transit for all study interventions received and any discrepancies are reported and resolved before use of the study intervention.

2. Only participants enrolled in the study may receive study intervention and only authorized site staff may supply or administer study intervention. All study interventions must be stored in a secure, environmentally controlled, and monitored (manual or automated recording) area in accordance with the labeled storage conditions with access limited to the investigator and authorized site staff. At a minimum, daily minimum and maximum temperatures for all site storage locations must be documented and available upon request. Data for nonworking days must indicate the minimum and maximum temperatures since previously documented for all site storage locations upon return to business.

3. Any excursions from the study intervention label storage conditions should be reported to Pfizer upon discovery along with any actions taken. The site should actively pursue options for returning the study intervention to the storage conditions described in the labeling, as soon as possible. Once an excursion is identified, the study intervention must be quarantined and not used until Pfizer provides permission to use the study intervention. Specific details regarding the definition of an excursion and information the site should report for each excursion will be provided to the site in the IP manual.

4. Any storage conditions stated in the SRSD will be superseded by the storage conditions stated on the label.

5. Study interventions should be stored in their original containers.

6. Site staff will instruct participants on the proper storage requirements for take-home study intervention.

7. See the IP manual for storage conditions of the study intervention once reconstituted and/or diluted.



090177e194745c57\Approved\Approved On: 22-Jul-2020 00:48 (GMT)

8. The investigator, institution, or the head of the medical institution (where applicable) is responsible for study intervention accountability, reconciliation, and record maintenance (ie, receipt, reconciliation, and final disposition records), such as the IPAL or sponsor-approved equivalent. All study interventions will be accounted for using a study intervention accountability form/record. All study intervention that is taken home by the participant, both used and unused, must be returned to the investigator by the participant. Returned study intervention must not be redispensed to the participants.

9. Further guidance and information for the final disposition of unused study interventions are provided in the IP manual. All destruction must be adequately documented. If destruction is authorized to take place at the investigator site, the investigator must ensure that the materials are destroyed in compliance with applicable environmental regulations, institutional policy, and any special instructions provided by Pfizer.

Upon identification of a product complaint, notify the sponsor within 1 business day of discovery as described in the IP Manual.

### 6.2.1. Preparation and Dispensing

A qualified staff member will dispense the study intervention using the IRT system via unique container numbers in the bottles or vials provided, in quantities appropriate according to the SoA. A second staff member will verify the dispensing. The participant/caregiver should be instructed to maintain the product in the bottles or vials, provided throughout the course of dosing and return the bottles or vials to the site at the next study visit.

See the IP manual for instructions on how to prepare the study intervention for administration. Study intervention should be prepared and dispensed by an appropriately qualified and experienced member of the study staff (eg, physician, nurse, physician's assistant, nurse practitioner, pharmacy assistant/technician, or pharmacist) as allowed by local, state, and institutional guidance. A second staff member will verify the dispensing.

All vials are single-use.

Only qualified personnel who are familiar with procedures that minimize undue exposure to themselves and to the environment should undertake the preparation, handling, and safe disposal of chemotherapeutic agents.

## 6.3. Measures to Minimize Bias: Randomization and Blinding

### 6.3.1. Allocation to Study Intervention

This is an open-label study; however, the specific study intervention dispensed to the participant will be assigned using an IRT. The site will contact the IRT prior to the start of study intervention administration for each participant. The site will record the study intervention assignment on the applicable CRF, if required. Potential bias will be reduced by central randomization and BICR evaluation of imaging data (Section 8.1.1.1).



090177e194745c57\Approved\Approved On: 22-Jul-2020 00:48 (GMT)

The investigator's knowledge of the treatment should not influence the decision to enroll a particular participant or affect the order in which participants are enrolled.

Study intervention will be dispensed at the study visits summarized in the SoA.

Returned study intervention must not be redispensed to the participants.

The study-specific IRT reference manual and IP manual will provide the contact information and further details on the use of the IRT system.

## 6.4. Study Intervention Compliance

When participants are dosed at the site, they will receive study intervention directly from the investigator or designee, under medical supervision. The dose of study intervention and study participant identification will be confirmed at the time of dosing by a member of the study site staff other than the person administering the study intervention.

The site will complete the required dosage Preparation Record located in the IP manual. The use of the Preparation Record is preferred, but it does not preclude the use of an existing appropriate clinical site documentation system. The existing clinical site's documentation system should capture all pertinent/required information on the preparation and administration of the dose. This may be used in place of the Preparation Record after approval from the sponsor and/or designee.

When participants self-administer study intervention(s) at home, compliance with study intervention will be assessed at each visit. Participants will receive a diary to document self-administered dosing of study intervention (encorafenib or capecitabine, as applicable) in each cycle to include the dose of study intervention taken, the date of dosing, if any doses were missed and the reason for the missed dose. Compliance will be assessed by direct questioning, counting returned tablets/capsules, and a review of diary entries during the site visits and documented in the source documents and eCRF. Deviation(s) from the prescribed dosage regimen should be recorded in the eCRF.

A record of the number of encorafenib capsules and capecitabine tablets dispensed to and taken by each participant must be maintained and reconciled with study intervention and compliance records. Intervention start and stop dates, including dates for intervention delays and/or dose reductions, will also be recorded in the eCRF.

## 6.5. Concomitant Therapy

The following subsections detail permitted and prohibited concomitant therapies for encorafenib. Guidance on concomitant therapy for all other study interventions can be found in the respective SRSDs.

### 6.5.1. Permitted Concomitant Medications/Therapies

In general, the use of any concomitant medication/therapies deemed necessary for the care of the participant is permitted, unless otherwise specified. Additional information regarding



090177e194745c57\Approved\Approved On: 22-Jul-2020 00:48 (GMT)

concomitant medications/therapies is provided in the Investigator's Brochure for encorafenib and the SRSD for all other study interventions.

Participants receiving medications outlined below must be carefully monitored for potentiating of toxicity due to any individual concomitant medication and may require dose titration of the drug substance. Investigators should use caution when prescribing concomitant medications, as clinical experience with these compounds in participants with cancer is often limited. Investigators should contact the Sponsor when they are unsure whether a drug should be prescribed to a participant in the clinical study. All concomitant medications/therapies, transfusions, procedures and dietary supplements must be documented on the eCRF.

#### 6.5.1.1. Surgical Resection

Participants who undergo complete resection of all metastatic disease may continue study intervention at the discretion of the Investigator and following discussion with the medical monitor.

#### 6.5.1.2. CYP and UGT Substrates and Inhibitors

Encorafenib is a reversible inhibitor of CYP1A2, CYP2B6, CYP2C8/9, CYP2D6, CYP3A4 and UGT1A1. It is also a time-dependent inhibitor of CYP3A4, and induced CYP1A2, CYP2B6, CYP2C9 and CYP3A4 in human hepatocytes. Permitted medications to be used with caution in this study include those that are sensitive substrates of CYP2B6, CYP2C8/9, CYP2D6, CYP3A4 and UGT1A1 or those substrates that have an NTI.

**There is a potential for encorafenib to induce CYP3A4, which may reduce the effectiveness of hormonal contraception methods. Therefore, the use of at least 1 form of non-hormonal contraception is required for females of childbearing potential during participation in this study.**

Caution should be used in participants receiving concomitant treatment with other drugs that are substrates of CYP3A4 as the efficacy of these drugs could be reduced when administered with encorafenib.

Encorafenib has been identified to be primarily metabolized by CYP3A4 and to a lesser extent by CYP2C19 in vitro. **Concomitant use of moderate CYP3A4 inhibitors should be avoided.** If use of a moderate CYP3A4 inhibitor is unavoidable, short-term use (≤30 days) following discussion with the Sponsor may be permitted with an accompanying dose reduction to one-half of the encorafenib dose prior to use of the moderate CYP3A4 inhibitor. After the inhibitor has been discontinued for 3 to 5 elimination half-lives, resume the encorafenib dose that was taken prior to initiating the CYP3A4 inhibitor.

For tabulated CYP substrates, inhibitors and inducers to be used with caution or avoided, please consult the following open-access websites:

- https://www.fda.gov/drugs/developmentapprovalprocess/developmentresources/druginteractionslabeling/ucm093664.htm



090177e194745c57\Approved\Approved On: 22-Jul-2020 00:48 (GMT)

- https://drug-interactions.medicine.iu.edu/MainTable.aspx

Additional information on CYP substrates, inhibitors and inducers can be found on the following subscription-based website:

- https://didb.druginteractioninfo.org/

#### 6.5.1.3. Transporter Substrates and Inhibitors

Encorafenib is a BCRP and P-gp inhibitor as well as a potent inhibitor of the renal transporters, OAT1, OAT3 and OCT2, and the hepatic transporters OATP1B1 and OATP1B3.

The co-administration of drugs that are known to be sensitive or NTI substrates of BCRP, P-gp, OAT1, OAT3, OCT2, OATP1B1 and OATP1B3 should be used with caution.

For tabulated transporter substrates, inhibitors and inducers to be used with caution or avoided, please consult with the FDA website:

- https://www.fda.gov/drugs/developmentapprovalprocess/developmentresources/druginteractionslabeling/ucm093664.htm

#### 6.5.1.4. Drugs with a Conditional or Possible Risk to Prolong the QT Interval and/or Induce Torsade de Pointes

Investigators should use caution when administering encorafenib with concomitant medications with a known, conditional or possible risk to prolong the QT interval and/or induce TdP. Participants receiving such medications must be carefully monitored for potentiating of toxicity due to any individual concomitant medication and may require dose titration of the concomitant medication. See the CredibleMeds® website:

- https://crediblemeds.org/pdftemp/pdf/CombinedList.pdf

### 6.5.2. Prohibited Concomitant Therapy

The following therapies are prohibited while participants are receiving study intervention (unless otherwise noted):

- Additional systemic anticancer agents such as cytotoxic chemotherapy, small-molecule targeted agents, biological agents, immune response modifiers or hormonal therapy.
- Investigational drugs and devices.
- Radiation therapy (not including palliative radiotherapy at focal sites that covers ≤10% of the bone marrow reserve)
  ***Note:*** The participant must have clear measurable disease outside the radiated field.
  Note: Radiation therapy to a symptomatic solitary lesion or to the brain may be considered on a case-by-case basis after consultation with the medical monitor.
  ***Note:*** Administration of palliative radiation therapy may be considered clinical



090177e194745c57\Approved\Approved On: 22-Jul-2020 00:48 (GMT)

progression for the purposes of determining PFS if accompanied by radiologic progression.

- Concomitant strong systemic CYP3A4 inhibitors, which could significantly increase the exposure of encorafenib.

  For tabulated prohibited CYP substrates, inhibitors and inducers, please consult the following open-access websites:

  - https://www.fda.gov/drugs/developmentapprovalprocess/developmentresources/druginteractionslabeling/ucm093664.htm
  - https://drug-interactions.medicine.iu.edu/MainTable.aspx

  Additional information on CYP substrates, inhibitors and inducers can be found on the following subscription-based website:

  - https://didb.druginteractioninfo.org/

- Concomitant moderate or strong systemic CYP3A4 inducers, which could significantly decrease the exposure of encorafenib.

### 6.5.3. Premedication and Concurrent Medication

#### 6.5.3.1. Premedication for Cetuximab Administration

All participants in the SLI and those in Arm A and Arm B of the Phase 3 portion must be premedicated in accordance with the cetuximab SRSD prior to the first dose of cetuximab in an effort to prevent an infusion or hypersensitivity reaction. Premedication is recommended prior to subsequent doses, but at the investigator's discretion.

#### 6.5.3.2. Loperamide

For symptoms of diarrhea and/or abdominal cramping, participants should be instructed to begin taking loperamide per the investigator's discretion. It is generally recommended that loperamide be started at the earliest sign of (1) a poorly formed or loose stool or (2) the occurrence of 1 to 2 more bowel movements than usual in 1 day or (3) an increase in stool volume or liquidity.

The loperamide is to be sourced locally and participants should have sufficient supply on hand prior to their initial dose in case antidiarrheal support is required. Additional antidiarrheal measures may be used at the discretion of the treating physician. Participants should be instructed to increase fluid intake to help maintain fluid and electrolyte balance during episodes of diarrhea.

#### 6.5.3.3. Antibiotics

Oral fluroquinolone treatment should be considered for any of the following:

- Diarrhea persisting for more than 24 hours despite loperamide
- ANC <500 (even in the absence of diarrhea or fever)
- Fever with diarrhea (even in the absence of neutropenia)



090177e194745c57\Approved\Approved On: 22-Jul-2020 00:48 (GMT)

- Antibiotic therapy should also be considered in participants who are hospitalized with prolonged diarrhea (even in the absence of neutropenia)

#### 6.5.3.4. Atropine

Lacrimation, rhinorrhea, miosis, diaphoresis, hot flashes, flushing, abdominal cramping, diarrhea, or other symptoms of early cholinergic syndrome may occur during or shortly after receiving irinotecan. Atropine, 0.25-1.0 mg IV or SC may be used to treat these symptoms. In participants with troublesome or recurrent symptoms, prophylactic administration of atropine shortly before irinotecan therapy may be considered. Additional antidiarrheal measures may be used at the discretion of the treating physician. Combination anticholinergic medications containing barbiturates or other agents (eg, Donnatal®) should not be used because these may affect irinotecan metabolism. Anticholinergics should be used with caution in participants with potential contraindications (eg, obstructive uropathy, glaucoma, tachycardia, etc).

## 6.6. Dose Modification

Cycles will consist of 21 days in participants receiving CAPOX (with or without bevacizumab) in the Control Arm and will be 28 days in duration for all other participants. In the SLI, and Arm B, Day 1 of a cycle is defined by administration of cetuximab and any component of mFOLFOX6 or FOLFIRI, and in the Control Arm by administration of any component of the chemotherapy regimen.

For participants in the SLI and those in Arm B and the Control Arm of the Phase 3 portion, Day 1 of a cycle may not begin until the ANC is $\geq$1500/mm$^3$, the platelet count is $\geq$100,000/mm$^3$, and any treatment-related GI toxicity has resolved to $\leq$Grade 1. Participants in the SLI and Arm B of the Phase 3 portion may continue daily encorafenib, however dosing with cetuximab should not be administered until Week 1 of the next cycle along with chemotherapy (mFOLFOX6 or FOLFIRI).

Participants will be monitored for AEs on an ongoing basis. If the participant develops a toxicity, the dose may be modified as outlined in the following sections. All dose modifications should be based on the worst preceding toxicity. All dosing interruptions and modifications must be recorded in the eCRF.

If assessment of the toxicity can be clearly attributed to a single study intervention, administration of the offending drug should be modified and the toxicity should be followed until resolution or return to baseline according to the respective dose modification guidelines described in the following sections. Treatment with the other agents in the combination may continue without alterations.

If attribution of the toxicity cannot be clearly ascribed to a single study intervention, then all agents should be evaluated as to attribution and dose modifications for each individual agent should be assessed according to the tables below. The toxicity should be followed until resolution or return to baseline.

090177e194745c57\Approved\Approved On: 22-Jul-2020 00:48 (GMT)

If the toxicity recurs after a dose reduction, the process outlined above should be followed once again to determine attribution to a single study intervention or to all agents.

### 6.6.1. Dose Modifications for Encorafenib

The recommended dose reductions for encorafenib are presented in Table 14. Dose reductions beyond the second dose reduction are not allowed for encorafenib.

Administration of encorafenib as a single agent is not recommended; if cetuximab is permanently discontinued in Arm A in the Phase 3 portion of the study, permanently discontinue encorafenib.

In the SLI and Arm B of the Phase 3 portion of the study, if encorafenib is permanently discontinued, cetuximab must also be permanently discontinued; however, therapy with the selected mFOLFOX6 or FOLFIRI may continue.

**Table 14. Recommended Encorafenib Dose Reductions**

| Dose Level | Encorafenib Dose |
|---|---|
| First Dose Reduction | 225 mg orally QD |
| Second Dose Reduction | 150 mg orally QD |

The recommended dose modifications for encorafenib are presented in Table 15 and are based on the existing dose modification guidelines provided in the current USPI for encorafenib.[30]

**Table 15. Recommended Encorafenib Dose Modifications**

| Severity of Adverse Reaction[a] | Dose Modification for Encorafenib |
|---|---|
| ***New Primary Malignancies*** | |
| Cutaneous (SCC, KA and any Other Suspicious Skin Lesion) | Maintain dose of encorafenib (dose interruptions or modifications are not required). Treatment of SCC, KA, and any other suspicious skin lesion (eg, new primary melanoma) should occur based upon institutional practice. |
| Non-cutaneous *RAS* mutation-positive malignancies | Permanently discontinue encorafenib. |
| ***Uveitis*** | |
| Grade 1-3 | If Grade 1 or 2 uveitis does not respond to specific ocular therapy, or for Grade 3 uveitis, hold encorafenib for up to 6 weeks.<br>• If improved, resume at same or reduced dose.<br>• If not improved, permanently discontinue encorafenib. |
| Grade 4 | Permanently discontinue encorafenib |



090177e194745c57\Approved\Approved On: 22-Jul-2020 00:48 (GMT)

### Table 15. Recommended Encorafenib Dose Modifications

| Severity of Adverse Reaction[a] | Dose Modification for Encorafenib |
|---|---|
| ***QTcF Prolongation*** | |
| QTcF >500 ms and ≤60 ms increase from baseline | Hold encorafenib until QTcF ≤500 ms. Resume at reduced dose.<br>• If more than one recurrence, permanently discontinue encorafenib. |
| QTcF >500 ms and >60 ms increase from baseline | Permanently discontinue encorafenib. |
| ***Hepatotoxicity*** | |
| Grade 2 AST or ALT increased | Maintain encorafenib dose.<br>• If no improvement within 4 weeks, hold encorafenib until improves to Grade 0-1 or to pre-treatment/baseline levels and then resume at same dose. |
| Grade 3 or 4 AST or ALT increased | see *Other Adverse Reactions.* |
| ***Rash*** | |
| Grade 1 | Maintain dose level of encorafenib.<br>Initiate rash treatment per institutional standards; rash should be closely monitored. |
| Grade 2 | 1st occurrence:<br>• Maintain dose level of encorafenib<br>• Initiate rash treatment per institutional standards; rash should be closely monitored.<br>• Reassess within ≤14 days. If rash worsens or does not improve, interrupt dosing of encorafenib until resolved to Grade ≤1. Then resume treatment at current dose level of encorafenib. For dermatitis acneiform, treatment with encorafenib may be maintained if, in the judgment of the investigator, the rash is considered to be unrelated to encorafenib. If treatment with encorafenib was maintained and no improvement within 8 days, interrupt dosing of encorafenib<br>2nd occurrence:<br>• Reassess within ≤14 days. If rash worsens or does not improve, interrupt dosing of encorafenib until resolved to Grade ≤1. Then resume treatment at current dose level of encorafenib. For dermatitis acneiform rash, treatment with encorafenib may be maintained if, in the judgment of the investigator, the rash is considered to be unrelated to encorafenib. If treatment with encorafenib was maintained and no improvement within 8 days, interrupt dosing of encorafenib |



090177e194745c57\Approved\Approved On: 22-Jul-2020 00:48 (GMT)

## Table 15. Recommended Encorafenib Dose Modifications

| **Severity of Adverse Reaction[a]** | **Dose Modification for Encorafenib** |
|---|---|
| Grade 3 | 1st occurrence:<br>• Interrupt dosing of encorafenib until resolved to Grade ≤1. Reassess weekly. Then resume treatment at current dose level of encorafenib.<br>• Consider referral to dermatologist and manage rash per dermatologist's recommendation.<br>2nd occurrence:<br>• Interrupt dosing of encorafenib until resolved to Grade ≤1. Then resume treatment at 1 reduced dose level of encorafenib. Resume treatment with encorafenib at the same dose level if, in the judgment of the Investigator, the rash is considered to be unrelated to encorafenib<br>• Consider referral to dermatologist and manage rash per dermatologist's recommendation |
| Grade 4 | Permanently discontinue encorafenib. |
| ***Nausea/Vomiting*** | |
| Grade 1-2 | Maintain dose level of encorafenib. Promptly institute antiemetic measure. |
| Grade 3 | Interrupt dosing of encorafenib until resolved to Grade ≤1. Then resume treatment at 1 reduced dose level of encorafenib.<br>Note: Interrupt dosing of encorafenib for ≥Grade 3 vomiting or Grade 3 nausea only if the vomiting or nausea cannot be controlled with optimal antiemetics (as per local practice) |
| Grade 4 | Permanently discontinue encorafenib. |
| ***Other Adverse Reactions (including Hemorrhage)*** | |
| Recurrent Grade 2<br>*OR*<br>First occurrence of any Grade 3 | Hold encorafenib for up to 4 weeks.<br>• If improves to Grade 0-1 or to pretreatment/baseline level, resume at reduced dose.<br>• If no improvement, permanently discontinue encorafenib. |
| First occurrence of any Grade 4 | Permanently discontinue encorafenib<br>*OR*<br>Hold encorafenib for up to 4 weeks.<br>• If improved to Grade 0-1 or to pretreatment/baseline level, then resume at reduced dose.<br>• If no improvement, permanently discontinue encorafenib. |
| Recurrent Grade 3 | Consider permanently discontinuing encorafenib. |
| Recurrent Grade 4 | Permanently discontinue encorafenib. |

a. NCI CTCAE version 4.03

090177e194745c57\Approved\Approved On: 22-Jul-2020 00:48 (GMT)

090177e194745c57\Approved\Approved On: 22-Jul-2020 00:48 (GMT)

### 6.6.2. Dose Modifications for Cetuximab

The recommended dose reductions for cetuximab are presented in Table 16.

Cetuximab may be reduced 2 dose levels to a minimum of 300 mg/m$^2$ for AEs or laboratory abnormalities (Appendix 3). Dose reductions beyond the second dose reduction are not allowed for cetuximab. When a dose reduction is required because of an AE, no subsequent dose re-escalation of cetuximab will be permitted for that participant for the duration of study treatment. If after resolution of an AE, treatment is resumed at the same dose, and the same toxicity reoccurs with the same severity, any re-initiation of treatment must be at the next lower dose level irrespective of duration, with some exceptions for skin toxicity. In addition, a participant must permanently discontinue study treatment if, after treatment is resumed at a lower dose of cetuximab, the same toxicity reoccurs with the same or worse severity.

Administration of cetuximab as a single agent is not recommended; if encorafenib is permanently discontinued in Arm A in the Phase 3 portion of the study, permanently discontinue cetuximab. In the SLI and Arm B in the Phase 3 portion of the study, if cetuximab is permanently discontinued, encorafenib may be continued in combination with the selected mFOLFOX6 or FOLFIRI.

**Table 16. Recommended Cetuximab Dose Reductions**

| **Dose Level** | **Cetuximab Dose** |
|---|---|
| First Dose Reduction | 400 mg/m$^2$ Q2W |
| Second Dose Reduction | 300 mg/m$^2$ Q2W |

Recommended dose modifications for cetuximab based on the occurrence of cetuximab treatment-related AEs are summarized in Table 17.

**Table 17. Recommended Dose Modifications for Cetuximab related Adverse Events**

| **Severity of Adverse Event** [a] | **Dose Modification for Cetuximab During a Cycle of Therapy** |
|---|---|
| ***Hypomagnesemia*** | Hypomagnesemia has been seen with cetuximab. Should hypomagnesemia occur, magnesium supplementation should be provided. No dose adjustment is required; however, continue careful monitoring. |
| ***Infusion Reaction:*** *If an infusion reaction occurs while cetuximab is being infused, the infusion should be stopped immediately and the participant should be evaluated.* | |
| Grade 1 or 2 | Restart and complete the disrupted infusion at the discretion of the Investigator. The infusion must be restarted at a reduced rate. Additional pre-medications such as antihistamines or low-dose systemic corticosteroids may be administered when the infusion is restarted per institutional standards.<br>All subsequent infusions must also be administered at the reduced rate. |
| Grade 3 or 4 | Permanently discontinue cetuximab |

**Table 17. Recommended Dose Modifications for Cetuximab related Adverse Events**

| Severity of Adverse Event [a] | Dose Modification for Cetuximab During a Cycle of Therapy |
|---|---|
| ***Pulmonary toxicities*** | |
| Grade 2 or worsening pulmonary symptoms | Cetuximab therapy should be stopped and symptoms investigated. Cetuximab therapy may resume when symptoms resolve to ≤Grade 1 |
| Grade 3 cough, dyspnea, hypoxia, pneumonitis, or pulmonary infiltrates | Hold cetuximab until interstitial lung disease is ruled out. Permanently discontinue cetuximab if interstitial lung disease is confirmed |
| ***Rash*** | |
| Grade 1 or 2 | Maintain dose level; consider initiating appropriate therapy (such as antihistamines, topical corticosteroids, and low-dose systemic corticosteroids) |
| Grade 3, despite therapy | Hold cetuximab until resolved to ≤Grade 2, then:<br>• If resolved in ≤7 days (or ≤14 days for acneiform rash), then maintain dose level<br>• If not resolved in ≤7 days despite appropriate skin toxicity therapy (or ≤14 days for acneiform rash), then permanently discontinue cetuximab |
| Grade 3 recurrent | Hold cetuximab until resolved to ≤Grade 2, then:<br>• If resolved in ≤7 days (or ≤14 days for acneiform rash), then decrease 1 dose level<br>• If not resolved in ≤7 days despite appropriate skin toxicity therapy (or ≤14 days for acneiform rash), then permanently discontinue cetuximab<br>• Permanently discontinue cetuximab after 3rd recurrence (upon 4th occurrence) |
| Grade 4, despite skin toxicity therapy | Permanently discontinue cetuximab |

a NCI CTCAE version 4.03

### 6.6.3. Dose Modifications for Oxaliplatin, Irinotecan and 5-FU as Part of mFOLFOX6, FOLFIRI, and FOLFOXIRI Regimens

The recommended dose reductions for oxaliplatin, irinotecan and 5-FU are presented in Table 18 and Table 19. Dose adjustments of each agent may be made independently based on the specific types of toxicities observed. Only those agents specified in the table below should be dose reduced.

090177e194745c57\Approved\Approved On: 22-Jul-2020 00:48 (GMT)

**Table 18. Recommended Dose Reductions for Oxaliplatin, Irinotecan and 5-FU as Part of mFOLFOX6 and FOLFIRI Q2W**

| Dose Level | Oxaliplatin | Irinotecan | 5-FU Bolus | 5-FU Infusion |
|---|---|---|---|---|
| First Dose Reduction | 65 mg/m$^2$ | 150 mg/m$^2$ | 320 mg/m$^2$ | 1920 mg/m$^2$ per 46-48 h |
| Second Dose Reduction | 50 mg/m$^2$ | 120 mg/m$^2$ | 270 mg/m$^2$ | 1600 mg/m$^2$ per 46-48 h |
| Third Dose Reduction | 40 mg/m$^2$ | 100 mg/m$^2$ | 230 mg/m$^2$ | 1360 mg/m$^2$ per 46-48 h |

**Table 19. Recommended Dose Reductions for Oxaliplatin, Irinotecan and 5-FU as Part of FOLFOXIRI Q2W**

| Dose Level | Oxaliplatin | Irinotecan | 5-FU Infusion (Starting dose 2400 mg/m$^2$) | 5-FU Infusion (Starting dose 3200 mg/m$^2$) |
|---|---|---|---|---|
| First Dose Reduction | 65 mg/m$^2$ | 130 mg/m$^2$ | 1920 mg/m$^2$ | 2560 mg/m$^2$ |
| Second Dose Reduction | 50 mg/m$^2$ | 100 mg/m$^2$ | 1600 mg/m$^2$ | 2100 mg/m$^2$ |
| Third Dose Reduction | 40 mg/m$^2$ | 80 mg/m$^2$ | 1360 mg/m$^2$ | 1720 mg/m$^2$ |

Recommended dose modifications for oxaliplatin, irinotecan, and 5-FU based on the occurrence of mFOLFOX6, FOLFIRI and FOLFOXIRI treatment-related AEs are summarized in Table 20.

Participants who require multiple dose reductions during a cycle for Grade 2 toxicity may, at the investigator's discretion, and based on the duration and reversibility of the toxicity, begin the following cycle at one dose level higher than the final dose level during that cycle. Dose reductions beyond the third dose reduction are not allowed for Oxaliplatin, Irinotecan or 5-FU. In the SLI and Arm B of the Phase 3, if oxaliplatin or irinotecan are permanently discontinued, therapy with 5-FU, encorafenib and cetuximab may continue. In addition, if the entire regimen of mFOLFOX6 or FOLFIRI are permanently discontinued, therapy with encorafenib and cetuximab may continue. In the Phase 3 Control Arm, if oxaliplatin is discontinued from the CAPOX regimen, treatment with capecitabine may continue.

No dose modifications are implemented for leucovorin. If 5-FU is held or omitted, then leucovorin should also be held or omitted, accordingly.

**Table 20. Recommended Oxaliplatin, Irinotecan and 5-FU Dose Modifications**

| Severity of Adverse Reaction[a] | Dose Modifications During a Cycle of Therapy | Doses for the Next and all Subsequent Cycles of Therapy |
|---|---|---|
| ***Hematologic toxicities*** | | |
| Grade 2 thrombocytopenia | Reduce 5-FU (bolus and infusion) and oxaliplatin or irinotecan one dose level for the remainder of the cycle. | Resume 5-FU (bolus and infusion) and oxaliplatin or irinotecan at the dose levels at the start of the prior cycle, provided platelets ≥100,000. |



090177e194745c57\Approved\Approved On: 22-Jul-2020 00:48 (GMT)

**Table 20. Recommended Oxaliplatin, Irinotecan and 5-FU Dose Modifications**

| Severity of Adverse Reaction[a] | Dose Modifications During a Cycle of Therapy | Doses for the Next and all Subsequent Cycles of Therapy |
|---|---|---|
| Grade 3 neutropenia or thrombocytopenia | Hold 5-FU (bolus and infusion) and oxaliplatin or irinotecan. If, during the current cycle, counts recover to ANC ≥1500 and platelets ≥75,000, hold the 5-FU bolus, resume 5-FU infusion and oxaliplatin or irinotecan may be resumed at one lower dose level for the remainder of the cycle. Held doses are not to be made up. | Permanently discontinue bolus 5-FU, continue infusional 5-FU and oxaliplatin or irinotecan at the reduced dose levels from the previous cycle. |
| Febrile neutropenia (defined as ANC <1000 and T ≥38.5°C) | Hold 5-FU (bolus and infusion) and either oxaliplatin or irinotecan. If fever and neutropenia resolve during the current cycle, hold the 5-FU bolus, resume 5-FU infusion and either oxaliplatin or irinotecan may be resumed at one lower dose level for the remainder of the cycle, provided ANC ≥1500 and platelets ≥75,000. Held doses are not to be made up. | Permanently discontinue bolus 5-FU, continue infusional 5-FU and oxaliplatin or irinotecan at the reduced dose levels from the previous cycle. |
| ***Gastrointestinal toxicities*** | | |
| For symptoms of diarrhea and/or abdominal cramping, participants should be instructed to begin taking loperamide as described in Section 6.5.3.2. In some instances, treatment with oral fluroquinolone and/or atropine may be appropriate as described in Section 6.5.3.3 and Section 6.5.3.4, respectively. | | |
| Grade 2 diarrhea following maximal medical intervention | Reduce 5-FU (bolus and infusion) and irinotecan one dose level for the remainder of the cycle. | Resume 5-FU (bolus and infusion) and irinotecan at the dose levels at the start of the prior cycle, provided diarrhea has fully resolved. |
| Grade 3 or 4 diarrhea | Hold 5-FU (bolus and infusion) and oxaliplatin or irinotecan. If diarrhea resolves to ≤Grade 2 during the current cycle, 5-FU (bolus and infusion) and oxaliplatin or irinotecan may be resumed at one lower dose level for the remainder of the cycle. Held doses are not made up. | Continue 5-FU (bolus and infusion) and oxaliplatin or irinotecan at the reduced dose levels from the previous cycle. |
| Grade 2 mucositis | Reduce 5-FU (bolus and infusion) and oxaliplatin or irinotecan one dose level for the remainder of the cycle. | Continue 5-FU (bolus and infusion) and oxaliplatin or irinotecan at the dose levels at the start of the prior cycle, provided mucositis has fully resolved. |

090177e194745c57\Approved\Approved On: 22-Jul-2020 00:48 (GMT)

### Table 20. Recommended Oxaliplatin, Irinotecan and 5-FU Dose Modifications

| Severity of Adverse Reaction[a] | Dose Modifications During a Cycle of Therapy | Doses for the Next and all Subsequent Cycles of Therapy |
|---|---|---|
| Grade 3 mucositis | Hold 5-FU (bolus and infusion) and oxaliplatin or irinotecan. If mucositis resolves to ≤Grade 2 during the current cycle, hold 5-FU bolus, resume 5-FU infusion and oxaliplatin or irinotecan will be resumed at one lower dose level for the remainder of the cycle. Held doses will not be made up. | Permanently discontinue 5-FU bolus, resume 5-FU infusion, and oxaliplatin or irinotecan at the dose levels at the start of the prior cycle. |
| Grade 4 mucositis | Hold all protocol therapy. If mucositis resolves to ≤Grade 2 during the current cycle, hold 5-FU bolus, resume 5-FU infusion and oxaliplatin or irinotecan may be resumed at one lower dose level for the remainder of the cycle. Bevacizumab and/or cetuximab will be resumed at the prior dose. Held doses will not be made up. | Permanently discontinue 5-FU bolus, resume 5-FU infusion and oxaliplatin or irinotecan at the reduced dose levels from the previous cycle |
| Grade 3 nausea or vomiting | If they persist/occur despite two treatments with adequate (combination) antiemetic therapy:<br>Reduce oxaliplatin or irinotecan one dose level for the remainder of the cycle.<br>***Note***: The use of aprepitant is prohibited for those participants receiving FOLFIRI. | Continue oxaliplatin or irinotecan at the reduced dose level from the previous cycle |
| Grade 4 nausea or vomiting | If they persist/occur despite two treatments with adequate (combination) antiemetic therapy:<br>Reduce 5-FU (bolus and infusion) and oxaliplatin or irinotecan one dose level for the remainder of the cycle.<br>***Note***: The use of aprepitant is prohibited for those participants receiving FOLFIRI. | Continue 5-FU (bolus and infusion) and oxaliplatin or irinotecan at the reduced dose levels from the previous cycle |
| ***Pulmonary toxicities*** | | |
| Grade 3 cough, dyspnea, hypoxia, pneumonitis, or pulmonary infiltrates | Hold oxaliplatin and bevacizumab (and cetuximab) until interstitial lung disease is ruled out. Continue 5-FU (bolus and infusion). Permanently discontinue all protocol therapy if interstitial lung disease is confirmed | |

090177e194745c57\Approved\Approved On: 22-Jul-2020 00:48 (GMT)

## Table 20. Recommended Oxaliplatin, Irinotecan and 5-FU Dose Modifications

| Severity of Adverse Reaction[a] | Dose Modifications During a Cycle of Therapy | Doses for the Next and all Subsequent Cycles of Therapy |
|---|---|---|
| ***Neurotoxicities*** | | |
| Grade 3 Paresthesias/dysesthesias | For Grade 3 neurotoxicity resolving to ≤Grade 2 between treatments, reduce oxaliplatin by one dose level for the remainder of the cycle | Continue oxaliplatin at the reduced dose levels from the previous cycle |
| | For ***recurrent*** Grade 3 neurotoxicity resolving to ≤Grade 2 between treatments, reduce oxaliplatin by one additional dose level for the remainder of the cycle and for subsequent cycles. (oxaliplatin will not be reduced beyond the third dose reduction. If further dose reduction is required for neurotoxicity, oxaliplatin will be permanently discontinued. Participants should continue to receive other protocol therapy) | Continue oxaliplatin at the reduced dose levels from the previous cycle |
| | For Grade 3 neurotoxicity ***persisting*** between treatments:<br>Permanently discontinue oxaliplatin. Participants should continue to receive other protocol therapy | |
| Grade 4 Paresthesias/dysesthesias | Permanently discontinue oxaliplatin. Participants should continue to receive other protocol therapy | |
| Pharyngo-laryngeal dysesthesia | Consider increasing the duration of oxaliplatin infusion up to 6 hours for all subsequent treatments.<br>Should a participant develop oxaliplatin-induced pharyngo-laryngeal dysesthesia, her/his oxygen saturation should be evaluated via a pulse oximeter; if normal, an anxiolytic agent may be given and the participant observed in the clinic until the episode has resolved. Following this toxicity, participants may continue protocol therapy if it is felt appropriate. | |
| ***Extravasation*** | Extravasation of oxaliplatin has been associated with necrosis; if extravasation is suspected, the infusion should be stopped, and the drug administered at another site. Extravasation may be treated according to institutional guidelines. | |
| ***Cardiovascular toxicities*** | | |
| Grade 4 or recurrent/worsening venous thromboembolic events after resumption of bevacizumab (See Table 23) | Permanently discontinue all protocol therapy (oxaliplatin, Irinotecan and 5-FU [bolus and infusion]). | |



090177e194745c57\Approved\Approved On: 22-Jul-2020 00:48 (GMT)

090177e194745c57\Approved\Approved On: 22-Jul-2020 00:48 (GMT)

**Table 20. Recommended Oxaliplatin, Irinotecan and 5-FU Dose Modifications**

| Severity of Adverse Reaction[a] | Dose Modifications During a Cycle of Therapy | Doses for the Next and all Subsequent Cycles of Therapy |
|---|---|---|
| Grade 3 pulmonary embolism | Permanently discontinue bevacizumab | |
| Grade 4 pulmonary embolism | Permanently discontinue all protocol therapy (oxaliplatin, Irinotecan and 5-FU [bolus and infusion]). | |
| Grade 3 cardiac ischemia/infarction | Permanently discontinue all protocol therapy (oxaliplatin, Irinotecan and 5-FU [bolus and infusion]). | |
| Grade 4 arterial thrombotic event | Permanently discontinue all protocol therapy (oxaliplatin, Irinotecan and 5-FU [bolus and infusion]). | |
| Grade 4 LV dysfunction | Permanently discontinue all protocol therapy (oxaliplatin, Irinotecan and 5-FU [bolus and infusion]). | |
| ***Hypersensitivity reactions*** | | |
| Grade 1 | Decrease the infusion rate of the suspect agent by 50% until symptoms resolve, then resume at the initial planned rate. | |
| Grade 2 | Stop infusion of the suspect agent. Administer H1 and/or H2 blockers, and/or steroids according to institutional policy. Restart the infusion when symptoms resolve and pretreat before all subsequent doses. Treat according to institutional policy. | |
| Grade 3 or 4 | Stop infusion of the suspect agent. Permanently discontinue the suspect agent (oxaliplatin, Irinotecan or 5-FU [bolus and infusion]) and notify the Sponsor. | |
| ***Other AEs*** | | |
| ≥Grade 3 | Hold all protocol treatment (oxaliplatin, Irinotecan and 5-FU [bolus and infusion]) and monitor toxicity at least weekly. If toxicity resolves to ≤Grade 1 within 4 weeks, treatment may be resumed, with 5-FU (bolus and infusion) and irinotecan or oxaliplatin at one lower dose level | Continue 5-FU (bolus and infusion) and oxaliplatin or irinotecan at the reduced dose levels from the previous cycle |

a. NCI CTCAE version 4.03

### 6.6.4. Dose Modifications for Capecitabine

The recommended dose reductions for capecitabine are presented in Table 21.

**Table 21. Recommended Capecitabine Dose Reductions**

| Dose Level | Capecitabine Dose |
|---|---|
| First Dose Reduction | 800 mg/$m^2$ orally BID |
| Second Dose Reduction | 600 mg/$m^2$ orally BID |

Recommended dose modifications for capecitabine treatment-related AEs are summarized in Table 22. In the Phase 3 Control Arm, if capecitabine is discontinued from the CAPOX regimen, treatment with oxaliplatin may continue.

**Table 22. Recommended Capecitabine Dose Modifications**

| Toxicity NCIC Grades | During a Course of Therapy | Dose Adjustment for Next Treatment (% of starting dose) |
|---|---|---|
| **Grade 1** | Maintain dose level | Maintain dose level |
| **Grade 2** | | |
| 1st appearance | Interrupt until resolved to Grade 0-1 | 100% |
| 2nd appearance | Interrupt until resolved to Grade 0-1 | 75% |
| 3rd appearance | Interrupt until resolved to Grade 0-1 | 50% |
| 4th appearance | Permanently discontinue treatment permanently | |
| **Grade 3** | | |
| 1st appearance | Interrupt until resolved to Grade 0-1 | 75% |
| 2nd appearance | Interrupt until resolved to Grade 0-1 | 50% |
| 3rd appearance | Interrupt until resolved to Grade 0-1 | |
| **Grade 4** | | |
| 1st appearance | Permanently discontinue permanently<br>*OR*<br>If physician deems it to be in the participant's best interest to continue, interrupt until resolved to Grade 0-1 | 50% |

### 6.6.5. Dose Modifications for Bevacizumab

No dose reductions for bevacizumab are recommended. Participants with colon or rectal stents should not receive bevacizumab due to increased risk of perforation.

For participants for whom elective surgery is contemplated, bevacizumab is to be discontinued for at least 8 weeks prior to surgery. Bevacizumab may be resumed after at least 4 weeks following surgery. These participants should also discontinue aspirin at least 1 week prior to surgery.



090177e194745c57\Approved\Approved On: 22-Jul-2020 00:48 (GMT)

For patients for whom non-elective surgery is required, hold bevacizumab as long as possible prior to surgery and for at least 6 weeks following surgery. Other protocol treatment may be given while bevacizumab is held at the discretion of the treating physician.

Recommended dose modifications for bevacizumab based on the occurrence of bevacizumab treatment-related AEs are summarized in Table 23.

**Table 23. Recommended Bevacizumab Dose Modifications**

| Severity of Adverse Reaction[a] | Dose Modification for Bevacizumab |
|---|---|
| ***Infusion Reactions*** | |
| If infusion-related adverse reactions occur, subsequent infusions should be administered over the shortest period that is well-tolerated. Participants may receive premedication with diphenhydramine 25 to 50 mg intravenously or orally 30 minutes prior to bevacizumab if they have previously experienced mild infusion reactions. Acetaminophen premedication may also be used. | |
| ***Reversible Posterior Leukoencephalopathy Syndrome (RPLS)*** | |
| For signs and symptoms suggestive of RPLS (eg, confusion, headache, seizures, cortical blindness) interrupt bevacizumab. Suspected RPLS should be investigated with MRI. If diagnosis of RPLS is confirmed, bevacizumab should be permanently discontinued. | |
| Grade 3 | If RPLS is ruled out via MRI, the decision on resuming bevacizumab should be based on the nature of the signs/symptoms. For grade 3 events, bevacizumab may be resumed if toxicities completely resolve within 4 weeks. |
| Grade 4 | If RPLS is ruled out via MRI, the decision on resuming bevacizumab should be based on the nature of the signs/symptoms. For grade 4 events with likely relationship to bevacizumab, discontinue bevacizumab. |
| ***Hypertension*** | |
| Grade 3 | Hold bevacizumab if not controlled with medical management; resume once controlled. Consider administering the following antihypertensive therapies in order of preference: calcium antagonists, ACE inhibitors, diuretics, and beta-blockers |
| Grade 4 | Permanently discontinue bevacizumab |
| ***Wound Healing Complications*** | |
| **Wound healing complications requiring medical intervention** | Permanently discontinue bevacizumab |
| Necrotizing fasciitis | Permanently discontinue bevacizumab |



090177e194745c57\Approved\Approved On: 22-Jul-2020 00:48 (GMT)

090177e194745c57\Approved\Approved On: 22-Jul-2020 00:48 (GMT)

### Table 23. Recommended Bevacizumab Dose Modifications

| Severity of Adverse Reaction[a] | Dose Modification for Bevacizumab |
|---|---|
| ***Posterior Reversible Encephalopathy Syndrome (PRES)*** | |
| Any Grade | Permanently discontinue bevacizumab |
| ***Hemorrhage*** | |
| Grade ≥2 pulmonary or CNS | Permanently discontinue bevacizumab |
| Grade 3 non-pulmonary and non-CNS | Hold bevacizumab until all of the following criteria are met:<br>• The bleeding has resolved and hemoglobin is stable<br>• There is no bleeding diathesis that would increase the risk of therapy<br>• There is no anatomic or pathologic condition that significantly increases the risk of hemorrhage recurrence |
| Grade 4 | Permanently discontinue bevacizumab |
| ***Venous thrombosis*** | |
| Grade 3 or 4 | Permanently discontinue bevacizumab |
| ***Arterial thromboembolic event*** | |
| Any Grade | Permanently discontinue bevacizumab |
| ***Congestive heart failure*** | |
| Symptomatic or NYHA Class ≥2 heart failure | Permanently discontinue bevacizumab |
| ***Renal Injury and Proteinuria*** | |
| Nephrotic syndrome | Permanently discontinue bevacizumab |
| ***GI perforation*** | |
| Any Grade | Permanently discontinue bevacizumab |
| ***Bowel obstruction*** | |
| Grade 1 | Participants who experience partial obstruction not requiring medical intervention may continue on bevacizumab |
| Grade 2 | Hold bevacizumab in participants who experience partial obstruction requiring medical intervention. Resume upon complete resolution |
| Grade 3/4 | Permanently discontinue bevacizumab |

**Table 23. Recommended Bevacizumab Dose Modifications**

| Severity of Adverse Reaction[a] | Dose Modification for Bevacizumab |
|---|---|
| Wound dehiscence | Permanently discontinue bevacizumab |
| ***Pulmonary toxicities*** | |
| Grade 3 cough, dyspnea, hypoxia, pneumonitis, or pulmonary infiltrates | Hold bevacizumab until interstitial lung disease is ruled out. Permanently discontinue bevacizumab if interstitial lung disease is confirmed |
| ***Other unspecified bevacizumab-related adverse events*** | |
| Grade 3 | Hold bevacizumab until recovery to ≤Grade 1 |
| Grade 4 | Permanently discontinue bevacizumab |

a. NCI CTCAE version 4.03

### 6.6.6. Guidance For Administration of Study Intervention in Participants with SARS-CoV-2 Infection

Each individual participants case is unique and decisions surrounding study intervention should be based on the investigator's clinical judgement and in accordance with local and regional recommendations. The following modifications are general suggestions for participants with active SARS-CoV-2 infection including those that are confirmed positive based on a locally approved test or presumed positive based on clinical suspicion:

- In participants with active infection and symptoms, consider delaying study intervention for at least 14 days from start of symptoms.
- Consider restarting treatment when SARS-CoV-2-associated symptoms have recovered to ≤Grade 1 for ≥72 hours and participant has been afebrile for ≥72 hours

## 6.7. Intervention After the End of the Study

Any participants still receiving study intervention at the End of Study will be allowed to continue study intervention at the discretion of the Investigator and Sponsor and as long as none of the treatment discontinuation criteria are met (see Section 7.1).

090177e194745c57\Approved\Approved On: 22-Jul-2020 00:48 (GMT)

# 7. DISCONTINUATION OF STUDY INTERVENTION AND PARTICIPANT DISCONTINUATION/WITHDRAWAL

## 7.1. Discontinuation of Study Intervention

It may be necessary for a participant to permanently discontinue study intervention (definitive discontinuation). Reasons for definitive discontinuation of study intervention include the following:

- Withdrawal of consent/assent. Participants may choose to withdraw from the study at any time without penalty of jeopardizing their healthcare or loss of benefits to which the participant is otherwise entitled. Participants will have the option of withdrawing consent/assent for study treatment but continue in the follow-up period of the study for safety/efficacy assessments. See Section 7.2.1.
- Unacceptable AEs or failure to tolerate study intervention defined as:
  - Grade 4 or life-threatening AE as outlined in Appendix 3, (except with approval from the medical monitor);
  - Toxicity requiring more than the allowed number of dose reductions for any study intervention as described in Section 6.5.3;
  - Occurrence of an AE that is related to study treatment and in the judgment of the Investigator compromises the participant's ability to continue study-specific procedures, or is considered to not be in the participant's best interest.
- Dose interruption due to an AE or clinically significant laboratory abnormality, unless judged by the Investigator and medical monitor or designee to be in the best interest of the participant to continue treatment, as follows:
  - Encorafenib: Dose interruption >28 consecutive days;
  - Cetuximab: >2 missed consecutive doses of cetuximab;
  - mFOLFOX6/FOLFIRI/FOLFOXIRI: If the initiation of a new cycle or therapy during a cycle is delayed for ≥4 weeks;
  - CAPOX: If the initiation of a new cycle or therapy during a cycle is delayed for ≥3 weeks.
- Progressive disease (defined by RECIST, v1.1; see Section 8.1.1).
  ***Note***: For the SLI, disease progression will be determined by Investigator. For the Phase 3 portion, disease progression will be identified by the investigator and confirmed by BICR.
  ***Note***: In Phase 3, if disease progression is identified by Investigator (based on clinical or radiographic evidence), every effort should be made to continue study intervention until confirmed by BICR (see Section 8.1.1.1.2).



090177e194745c57\Approved\Approved On: 22-Jul-2020 00:48 (GMT)

- Participant becomes pregnant or begins breastfeeding.
- Significant protocol deviation that, in the opinion of the Investigator and/or Sponsor, renders the participant unsuitable for further study intervention administration.
- Participant is noncompliant with study procedures or study treatment that in the judgment of the Investigator or Sponsor renders the participant unsuitable for further study participation.
- Lost to follow-up.
- Death.
- Termination of the study by the Sponsor.

Note that discontinuation of study intervention does not represent withdrawal from the study. If study intervention is definitively discontinued, the participant will remain in the study to be evaluated for safety, disease assessments, subsequent anticancer therapies, and survival. See the SoA for data to be collected at the time of discontinuation of study intervention and follow-up for any further evaluations that need to be completed.

In the event of discontinuation of study intervention, it must be documented on the appropriate CRF/in the medical records whether the participant is discontinuing further receipt of study intervention or also from study procedures, posttreatment study follow-up, and/or future collection of additional information.

In the event of study termination, the sponsor may work with sites to explore options for alternative source of post-study treatment for remaining participants.

After participants have discontinued treatment for any reason, they will be contacted approximately every 3 months until withdrawal of consent/assent, the participant is lost to follow-up, death or defined End of Study (Section 4.4). If participants withdraw consent/assent for study intervention they will continue to be contacted for survival status unless they specifically request that they not be contacted. If the participant refuses to be contacted, attempts to determine survival status should be made via access to public records where permitted by local laws.

When a participant discontinues treatment for "disease progression" without BICR confirmation, it will not be counted as a PFS event and disease assessments will continue to be performed until PD is determined by BICR (see Section 8.1.1).

## 7.2. Participant Discontinuation/Withdrawal From the Study

A participant may withdraw from the study at any time at his/her own request. Reasons for discontinuation from the study include the following:

- Refused further follow-up (ie, withdrawal of consent/assent);
- Lost to follow-up;

090177e194745c57\Approved\Approved On: 22-Jul-2020 00:48 (GMT)

- Death;
- Study terminated by sponsor;
- Termination of the study by the local health authority, IRB or IEC.

At the time of discontinuing from the study, if possible, an end of treatment visit should be conducted. See the SoA for assessments to be collected at the time of study discontinuation and follow-up and for any further evaluations that need to be completed.

If a participant withdraws from the study, he/she may request destruction of any remaining samples taken and not tested, and the investigator must document any such requests in the site study records and notify the sponsor accordingly.

If the participant withdraws from the study and also withdraws consent/assent (see Section 7.2.1) for disclosure of future information, no further evaluations should be performed and no additional data should be collected. The sponsor may retain and continue to use any data collected before such withdrawal of consent/assent.

Lack of completion of all or any of the withdrawal/early termination procedures will not be viewed as protocol deviations so long as the participant's safety was preserved.

### 7.2.1. Withdrawal of Consent/Assent

Participants who request to discontinue receipt of study intervention will remain in the study and must continue to be followed for protocol-specified follow-up procedures. The only exception to this is when a participant specifically withdraws consent/assent for any further contact with him or her or persons previously authorized by the participant to provide this information. Participants should notify the investigator in writing of the decision to withdraw consent/assent from future follow-up, whenever possible. The withdrawal of consent/assent should be explained in detail in the medical records by the investigator, as to whether the withdrawal is only from further receipt of study intervention or also from study procedures and/or posttreatment study follow-up, and entered on the appropriate CRF page. In the event that vital status (whether the participant is alive or dead) is being measured, publicly available information should be used to determine vital status only as appropriately directed in accordance with local law.

## 7.3. Lost to Follow-up

A participant will be considered lost to follow-up if he or she repeatedly fails to return for scheduled visits and is unable to be contacted by the study site.

The following actions must be taken if a participant fails to return to the clinic for a required study visit:

- The site must attempt to contact the participant and reschedule the missed visit as soon as possible and counsel the participant on the importance of maintaining the



090177e194745c57\Approved\Approved On: 22-Jul-2020 00:48 (GMT)

assigned visit schedule and ascertain whether or not the participant wishes to and/or should continue in the study;

- Before a participant is deemed lost to follow-up, the investigator or designee must make every effort to regain contact with the participant (where possible, 3 telephone calls and, if necessary, a certified letter to the participant's last known mailing address or local equivalent methods). These contact attempts should be documented in the participant's medical record;
- Should the participant continue to be unreachable, he/she will be considered to have withdrawn from the study.

# 8. STUDY ASSESSMENTS AND PROCEDURES

The investigator (or an appropriate delegate at the investigator site) must obtain a signed and dated ICD before performing any study-specific procedures.

Study procedures and their timing are summarized in the SoA. Protocol waivers or exemptions are not allowed.

Safety issues should be discussed with the sponsor immediately upon occurrence or awareness to determine whether the participant should continue or discontinue study intervention (see Section 10.1.1.1).

Adherence to the study design requirements, including those specified in the SoA, is essential and required for study conduct.

All screening evaluations must be completed and reviewed to confirm that potential participants meet all eligibility criteria. The investigator will maintain a screening log to record details of all participants screened and to confirm eligibility or record reasons for screening failure, as applicable.

Procedures conducted as part of the participant's routine clinical management (eg, blood count) and obtained before signing of the ICD may be utilized for screening or baseline purposes provided the procedures met the protocol-specified criteria and were performed within the time frame defined in the SoA.

Every effort should be made to ensure that protocol-required tests and procedures are completed as described. However, it is anticipated that from time to time there may be circumstances outside the control of the investigator that may make it unfeasible to perform the test. In these cases, the investigator must take all steps necessary to ensure the safety and well-being of the participant. When a protocol-required test cannot be performed, the investigator will document the reason for the missed test and any corrective and preventive actions that he or she has taken to ensure that required processes are adhered to as soon as possible. The study team must be informed of these incidents in a timely manner.

090177e194745c57\Approved\Approved On: 22-Jul-2020 00:48 (GMT)

For samples being collected and shipped, detailed collection, processing, storage, and shipment instructions and contact information will be provided to the investigator site prior to initiation of the study.

## 8.1. Efficacy Assessments

### 8.1.1. Tumor Response Assessments

Tumor responses will be evaluated according to RECIST, v1.1 (Appendix 9). Measurable disease will be based on local Investigator assessment of baseline scans. Baseline scans should be sent to the BICR within 30 days of randomization. For the SLI portion of the study, tumor responses will be evaluated locally by the Investigator and may be transmitted to an imaging vendor for BICR review. For the Phase 3 portion of the study, tumor responses will be evaluated locally by the Investigator and centrally by BICR.

All potential sites of tumor lesions will be assessed at the time points specified in Table 1 (Screening), Table 2 (SLI), Table 3 (Arm A and Arm B), Table 4 and Table 5 (Control Arm):

At screening (prior to randomization for participants in the Phase 3 portion), the following should be performed:

- A CT scan with IV contrast of chest, abdomen and pelvis is the preferred technique. If there is concern about radiation exposure, an MRI may be used instead of a CT.
- In participants with a history of asymptomatic brain metastases, a brain MRI or CT scan
- If clinically indicated, a whole body bone scan (ie, if bone metastases are suspected or known at baseline). Sites may use a whole body bone imaging method per their local standard of care (eg, Tc99m bone scan, FDG-PET, NaF PET scan or whole-body bone MRI). Skeletal lesions identified on a whole body bone scan at baseline, which are not visible on the chest, abdomen, or pelvis CT (or MRI) scan should be imaged at baseline using localized CT, MRI, or X-ray.

Every effort must be made to assess each lesion that is measured at screening by the same method throughout the study so that the comparison is consistent.

The first post-baseline scan must be performed 42 to 48 days from the date of first dose. All post-screening assessments should be performed every 6 weeks from the date of randomization (or from the first dose for SLI) for the first 18 months of treatment, then every 8 weeks thereafter until BICR-confirmed disease progression, withdrawal of consent/assent, initiation of subsequent anticancer therapy, participant is lost to follow-up, death or defined End of Study (Section 4.4).

Regardless of whether study treatment is discontinued, the following should be performed:

- Chest, abdomen, and pelvis CT (or MRI) scans



090177e194745c57\Approved\Approved On: 22-Jul-2020 00:48 (GMT)

- Brain MRI or CT scan, if metastases were documented at baseline

- Skeletal lesions identified at baseline should continue to be imaged at subsequent scheduled visits using localized CT, MRI, or X-ray (using the same method used at baseline for all visits for any given lesion). After baseline, whole body bone scans need not be repeated, unless clinically indicated.

- Additional imaging evaluations may be performed if there is symptomatic evidence suggesting the possibility of disease progression based on clinical symptoms or physical examination at any time.

If off-schedule imaging evaluations are performed or if progression is suspected, every effort should be made to perform subsequent imaging evaluations in accordance with the original imaging schedule.

All CT scans should be performed with IV contrast. If a participant is known to have a medical contraindication to the contrast agent or develops a contraindication during the study, a CT scan without IV contrast of the chest and MRI with IV contrast, if possible, of the abdomen and pelvis may be performed. A CT scan of the brain, with IV contrast, if possible, may be performed if MRI is contra-indicated.

Chest X-ray or ultrasound should not be used for tumor response assessments in this study.

Any lesions that have been subjected to loco-regional therapies (eg, radiotherapy, ablation, etc) should be considered non-measurable, unless they have clearly progressed since the therapy. Previously treated lesions that have not progressed should be considered non-measurable and therefore, assessed as non-target lesions.

While FDG-PET scans are not required for this study, sites may perform combined PET/CT scans per their local standard of care, provided the CT is of similar diagnostic quality as CT performed without PET, including the use of oral and IV contrast media. If acquired according to local standard of care, FDG-PET may be relied upon to document PD in accordance with RECIST.

Each center should have a designated radiologist responsible for the interpretation of scans and response evaluations for study participants. At a minimum, a single radiologist should perform all evaluations for an individual participant.

#### 8.1.1.1. BICR Evaluation of Imaging Data

All imaging data acquired in the Phase 3 portion of the study for efficacy purposes (eg, CT/MRI scans) will be transmitted to an imaging vendor for BICR review. Imaging data acquired in the SLI portion of the study may also be transmitted to an imaging vendor for BICR review. These images will be read by readers who are blinded to treatment assignment and to other clinical data as specified in the BICR Charter. Image transmission to the imaging vendor should be performed according to the imaging vendor manual.



090177e194745c57\Approved\Approved On: 22-Jul-2020 00:48 (GMT)

090177e194745c57\Approved\Approved On: 22-Jul-2020 00:48 (GMT)

##### 8.1.1.1.1. Time Points without Locally Determined Progression

Tumor measurements and/or assessments for time points with no locally determined progression received by the BICR will be read on an ongoing basis (rolling reads) in Phase 3 as detailed in the Imaging Manual. BICR interpretation of these data will not be communicated to the site, even if progression is assessed by the BICR unless it is an expedited review (please see below).

##### 8.1.1.1.2. Time Points with Locally Determined Progression

All participants in Phase 3 who have PD determined by the local Investigator require an expedited tumor response review by the BICR, and sites must send the radiological data to the BICR immediately upon acquisition. The Investigator seeking an expedited review must indicate this request on the radiology transmittal form. The imaging vendor will ensure that the central reviewers involved in the BICR are blinded to the expedited status of the reading.

The BICR will perform an expedited review of radiological data and the results of the reading will be communicated to the site.

While the Investigator is waiting for the results from the BICR, every effort should be made to continue administration of study intervention. However, during this time, the Investigator should do whatever is medically necessary for the participant.

If the BICR determines PD, the study site will be informed and the participant will discontinue study intervention, cease tumor assessments and begin follow-up assessments as outlined in the SoA.

If the BICR does not conclude PD, the study site will be informed and the participant should continue receiving study intervention unless clinically indicated (eg, rapid clinical deterioration) for an immediate change in therapy. Participants will continue to have tumor assessments performed every 6 weeks from randomization for the first 18 months, then every 8 weeks for the remainder of the study, irrespective of the days of study intervention administration, until the BICR declares PD, withdrawal of consent/assent, initiation of subsequent anticancer therapy, lost to follow-up or death.

In summary, for expedited time points (time points assessed as PD by the local Investigator):

- Sites must send the radiological and/or pathological data to the BICR immediately upon acquisition. While the Investigator is waiting for the results from the BICR, it is preferable that the participant continue treatment with study intervention.
- If the BICR determines PD, the study site will be informed. This participant will then discontinue study intervention, cease tumor assessments and begin follow-up assessments.

- If the BICR does not conclude PD, the study site will be informed. As long as it is clinically acceptable, every effort should be made to continue the participant on study intervention until the BICR declares PD.

### 8.1.2. Patient-reported Outcomes

Cancer-specific global health status and quality of life, functioning, and symptoms data will be collected using the EORTC QLQ-C30 questionnaire and general health status will be assessed using the EQ-5D-5L health questionnaire. PGIS and PGIC will also be collected (Appendix 10) according to FDA guidance[55] to enable the implementation of anchor-based methods to establish meaningful change in PROs.

These questionnaires will be administered at the time points specified in Table 3 (Arm A and Arm B), and Table 4 and Table 5 (Control Arm). These questionnaires will be used to explore PRO measures of health-related QoL, functioning, cancer symptoms, and treatment-related side effects.

The EORTC QLQ-C30 is a reliable and valid self-administered questionnaire used in oncology trials.[48-50] The questionnaire is easily understood by most participants and is quick to complete (mean time 11 minutes).

It contains 30 items and is composed of both multi-item scales and single-item measures. These include five functional scales (physical, role, emotional, cognitive and social functioning), three symptom scales (fatigue, nausea/vomiting, and pain), six single items (dyspnea, insomnia, appetite loss, constipation, diarrhea and financial impact) and a global health status/QoL scale . All the scales and single-item measures range in score from 0 to 100.

Higher scores on the functional scales represent higher levels of functioning. Higher scores on the global health status/quality of life scale represent higher health status/quality of life. Higher scores on symptom scales/items represent a greater presence of symptoms.

The EQ-5D-5L is a standardized measure of health utility that provides a single index value for the participant's health status. It is frequently used for economic evaluations of health care and has been shown to be a valid and reliable instrument, and comprises a short descriptive system questionnaire and a visual analogue scale (EQ VAS) that are cognitively undemanding, taking about 2 minutes to complete.

The EQ-5D-5L contains one item for each of five dimensions of HRQoL (ie, mobility, self-care, usual activities, pain or discomfort, and anxiety or depression). Response options for each item vary from having no problems (eg, "...no problems walking about"), moderate problems (e.g., "...some problems walking about"), or extreme problems (eg, "...unable to walk about"). Participant responses to the five dimensions of HRQoL reflect a specific health state that corresponds to a population preference weight for that state on a continuous scale of 0 (death) to 1 (perfect health).

090177e194745c57\Approved\Approved On: 22-Jul-2020 00:48 (GMT)

The visual analog scale (ranging from 0 to 100) is included to capture participant's rating of their overall health status. Higher scores of the EQ-5D-5L represent better health states. All scoring and handling of data will follow the User's Guide defined by the EuroQoL Group.

The PGIS is a single-item questionnaire designed to assess participant's overall impression of disease severity at a given point in time. The PGIC is a single-item questionnaire designed to assess the participant's overall sense of whether there has been a change in symptoms or QoL since starting treatment. The PGIS and PGIC can be employed as anchors in responder analyses of other PRO instruments, to help interpret clinically meaningful changes in scores.

The questionnaires should be administered to participants in their preferred language at the beginning of the study visit prior to receiving any study treatment, prior to any other study assessment or consultation with the Investigator, and prior to being informed of their current disease status.

Attempts should be made to collect all questionnaires for all participants, including those who discontinue prior to the EOT visit. However, if the participant refuses to complete the questionnaires, this should be documented in study source records. Participant refusal to complete study questionnaires is not a protocol deviation.

Participants should be given sufficient space and time to complete all study questionnaires, and all administered questionnaires should be reviewed for completeness. If missing responses are noted, participants should be encouraged to complete any missing responses.

Completed questionnaires, including both responses to the questions and any unsolicited comments written by the participant, should be reviewed by the Investigator or designee to ensure every question has been answered and that there is only 1 response for each question. If omissions or double responses occur, they should be brought to the attention of the participant. Investigators must not encourage the participant to change responses reported in questionnaires.

In addition, the completed questionnaires should be reviewed and assessed by the Investigator for responses which may indicate potential AEs or SAEs. This review should be documented in study source records.

## 8.2. Safety Assessments

Planned time points for all safety assessments are provided in the SoA. Unscheduled clinical laboratory measurements may be obtained at any time during the study to assess any perceived safety issues.

Medical history, physical examination, and neurological examination information, as applicable, collected during the course of the study will be considered source data only and will not be required to be reported on the case report form, unless otherwise noted. However, any untoward findings identified on physical and/or neurological examinations conducted during the active collection period will be captured as AEs, if those findings meet the definition of an AE. Data collected at screening that are used for inclusion/exclusion criteria,



090177e194745c57\Approved\Approved On: 22-Jul-2020 00:48 (GMT)

such as laboratory data, ECGs, and vital signs, as well as demographic data collected at screening will be reported.

### 8.2.1. Participant Demographics and Other Baseline Characteristics

Demographic data will be collected at Prescreening and general medical history will be collected at Screening by the Investigator or qualified designee and will include relevant medical and surgical history within the last 10 years and current illnesses.

### 8.2.2. Disease Characteristics and Treatment History

A disease-targeted medical and treatment history will be collected at Screening. Details regarding the participant's malignancy under study, including date and stage at initial diagnosis, date and extent of metastatic disease at study entry, tumor histology, relevant disease characteristics and prior treatments, including systemic, radiation and surgical procedures, will be recorded.

### 8.2.3. Physical Examinations

At Screening, the physical examination should be comprehensive and include the examination of general appearance, skin, neck (including thyroid), eyes, ears, nose, throat, lungs, heart, abdomen, back, lymph nodes, extremities, vascular and neurological. If indicated based on medical history and/or symptoms, rectal, external genitalia, breast and pelvic examinations will be performed. Body weight will be measured as part of the physical examination on Day 1 of each cycle. For participants ≥18 year old, height will be measured only at Screening. For participants <18 year old, height will be measured at Screening and Day 1 of each cycle.

For subsequent visits, the physical examinations should be targeted as clinically indicated. Investigators should pay special attention to clinical signs related to previous serious illnesses.

All physical examinations occurring on dosing days must be performed prior to study intervention administration. Any treatment-emergent abnormal findings will be recorded as AEs.

### 8.2.4. Body Surface Area

BSA will be calculated at the start of each cycle. The most recent participant weight available should be used to calculate BSA in order to determine the appropriate dose for that cycle. If the participant's weight at the beginning of each cycle varies by >10% from the previous cycle, the dose must be recalculated.

In calculating the BSA, actual height and weight should be used. In participants with BSA >2.2 $m^2$, either actual BSA or 2.2 $m^2$ may be used. As per ASCO guidelines, any of the established formulas may be used to calculate BSA.[56] A single formula should be used consistently throughout the study for a given participant.

090177e194745c57\Approved\Approved On: 22-Jul-2020 00:48 (GMT)

090177e194745c57\Approved\Approved On: 22-Jul-2020 00:48 (GMT)

### 8.2.5. Dermatological Examinations

Dermatologic evaluations will be performed at the site by the Investigator to monitor for the possible development of keratoacanthoma and/or squamous cell carcinoma, as these have been reported to occur with selective BRAF inhibitor treatment.[57-59] This assessment may be performed predose or postdose at the time points specified in the SoA. Following the 28-day follow-up, dermatologic examinations for skin malignancies are recommended every 2 months until 6 months after the last dose of encorafenib or until initiation of another antineoplastic therapy. Participants should be referred to a dermatologist for any abnormalities identified on the Investigator's dermatologic examination.

### 8.2.6. Vital Signs

Temperature, pulse rate, respiratory rate, and blood pressure will be assessed per institutional standards prior to blood collection for laboratory tests.

All vital sign measurements occurring on dosing days must be performed prior to study intervention administration. Abnormal vital sign results identified after the first dose of study treatment constitute an AE if they are considered clinically meaningful, induce clinical signs or symptoms, require concomitant therapy or require changes in study intervention/treatment.

### 8.2.7. Electrocardiograms

Standard 12-lead ECGs utilizing limb leads (with a 10 second rhythm strip) should be collected at times specified in the SoA using an ECG machine that automatically calculates the heart rate and measures PR, QT, and QTcF intervals and QRS complex. Alternative lead placement methodology using torso leads (eg, Mason-Likar) should not be done given the potential risk of discrepancies with ECGs acquired using standard limb lead placement.

A triplicate ECG (3 serial ECGs conducted within approximately 5 to 10 minutes total time) will be performed at screening to determine eligibility and predose on Cycle 1 Day 1 to serve as baseline. A single ECG will be performed at all remaining time points specified in the SoA. If single ECG reveals QTc abnormality, continuous ECG should be performed as noted below. All scheduled ECGs should be performed after the participant has rested quietly for at least 10 minutes in a supine position. When an ECG is to be performed at the same time point as a blood collection, the ECG is to be performed first.

A study participant should undergo continuous ECG monitoring if:

a. a postdose QTcF interval remains ≥60 msec from the baseline **and** is >450 msec; or

b. an absolute QTcF value is ≥500 msec for any scheduled ECG for greater than 4 hours (or sooner, at the discretion of the investigator); or

c. QTcF intervals get progressively longer by 30 ms increments.

Consider consulting a cardiologist if QTcF intervals do not return to less than the criteria listed above after 8 hours of monitoring (or sooner, at the discretion of the investigator). New or worsened clinically significant findings occurring after the informed consent/assent

must be recorded as an AE in the eCRF. ECG tracings should be made available if requested by the Sponsor.

In some cases, it may be appropriate to repeat abnormal ECGs to rule out improper lead placement as contributing to the ECG abnormality. It is important that leads be placed in the same positions each time in order to achieve precise ECG recordings. If a machine-read QTcF value is prolonged, as defined above, repeat measurements may not be necessary if a qualified medical provider's interpretation determines that the QTcF values are in the acceptable range.

ECG values of potential clinical concern are listed in Appendix 7.

### 8.2.8. Clinical Safety Laboratory Assessments

See Appendix 2 for the list of clinical safety laboratory tests to be performed and the SoA for the timing and frequency. All protocol-required laboratory assessments, as defined in Appendix 2, must be conducted in accordance with the laboratory manual and the SoA. Unscheduled clinical laboratory measurements may be obtained at any time during the study to assess any perceived safety issues.

The investigator must review the laboratory report, document this review, and record any clinically relevant changes occurring during the study in the AE section of the CRF. Clinically significant abnormal laboratory findings are those which are not associated with the underlying disease, unless judged by the investigator to be more severe than expected for the participant's condition.

All laboratory tests with values considered clinically significantly abnormal during participation in the study or within 28 days after the last dose of study intervention should be repeated until the values return to normal or baseline or are no longer considered clinically significant by the investigator or medical monitor.

If such values do not return to normal/baseline within a period of time judged reasonable by the investigator, the etiology should be identified and the sponsor notified.

See Appendix 6 for suggested actions and follow-up assessments in the event of potential drug-induced liver injury.

At Screening, laboratory results from the central laboratory should be used by the Investigator to determine the participant's eligibility for the study. If a particular central laboratory result is not available, a local laboratory test result for that particular analyte may be utilized to determine a participant's eligibility to participate in the study. Local laboratory test results may also be used at baseline and when results are time-sensitive (eg, for participant safety or adjustment of study intervention dose or schedule). Part of the sample obtained for local laboratory analysis should be sent to the central laboratory for analysis. Local laboratory results obtained during the study will not be captured in the eCRF unless they are needed to clarify why a treatment decision was made or an AE was recorded.

090177e194745c57\Approved\Approved On: 22-Jul-2020 00:48 (GMT)

Additional unscheduled clinical laboratory tests may be obtained at any time during the study at the Investigator's discretion. In addition to local testing, these samples should also be sent to the central laboratory for analysis when feasible.

### 8.2.9. Pregnancy Testing

Pregnancy tests may be urine or serum tests (serum required at Screening), but must have a sensitivity of at least 25 mIU/mL. Pregnancy tests will be performed in WOCBP at the times listed in the SoA. Following a negative pregnancy test result at screening, appropriate contraception must be commenced and a second negative pregnancy test result will be required at the baseline visit prior the participant's receiving the study intervention. Pregnancy tests will also be done whenever 1 menstrual cycle is missed during the active treatment period (or when potential pregnancy is otherwise suspected) and at the end of the study. Pregnancy tests may also be repeated if requested by IRBs/ethics committees or if required by local regulations. If a urine test cannot be confirmed as negative (eg, an ambiguous result), a serum pregnancy test is required. In such cases, the participant must be permanently discontinued from all study intervention if the serum pregnancy result is positive.

### 8.2.10. ECOG Performance Status

Assessment of ECOG PS (Table 24) will be assessed at the time points specified in Table 1 (Screening), Table 2 (SLI), Table 3 (Arm A and Arm B), Table 4 and Table 5 (Control Arm).

ECOG PS should be obtained on the scheduled day, even if study treatment is being held.

**Table 24. Eastern Cooperative Oncology Group (ECOG) Performance Status Scale**

| | |
|---|---|
| 0 | Fully active, able to carry on all predisease performance without restriction. |
| 1 | Restricted in physically strenuous activity but ambulatory and able to carry out work of a light or sedentary nature, eg, light housework, office work. |
| 2 | Ambulatory and capable of all self-care but unable to carry out any work activities. Up and about more than 50% of waking hours. |
| 3 | Capable of only limited self-care, confined to bed or chair more than 50% of waking hours. |
| 4 | Completely disabled. Cannot carry on any self-care. Totally confined to bed or chair. |
| 5 | Dead |

## 8.3. Adverse Events and Serious Adverse Events

The definitions of an AE and an SAE can be found in Appendix 3.

AEs will be reported by the participant (or, when appropriate, by a caregiver, surrogate, or the participant's legally authorized representative).

The investigator and any qualified designees are responsible for detecting, documenting, and recording events that meet the definition of an AE or SAE and remain responsible to pursue



090177e194745c57\Approved\Approved On: 22-Jul-2020 00:48 (GMT)

and obtain adequate information both to determine the outcome and to assess whether the event meets the criteria for classification as an SAE or caused the participant to discontinue the study intervention (see Section 7.1).

Each participant will be questioned about the occurrence of AEs in a nonleading manner.

In addition, the investigator may be requested by Pfizer Safety to obtain specific follow-up information in an expedited fashion.

### 8.3.1. Time Period and Frequency for Collecting AE and SAE Information

The time period for actively eliciting and collecting AEs and SAEs ("active collection period") for each participant begins from the time the participant provides informed consent/assent, which is obtained before the participant's participation in the study (ie, before undergoing any study-related procedure and/or receiving study intervention), through and including a minimum of 28 calendar days, except as indicated below, after the last administration of the study intervention. Beyond 28 days after the last dose of study treatment, only SAEs considered related to study treatment should be collected.

Follow-up by the investigator continues throughout and after the active collection period and until the AE or SAE or its sequelae resolve or stabilize at a level acceptable to the investigator and Pfizer concurs with that assessment.

For participants who are screen failures, the active collection period ends when screen failure status is determined.

If the participant withdraws from the study and also withdraws consent/assent for the collection of future information, the active collection period ends when consent/assent is withdrawn.

If a participant definitively discontinues or temporarily discontinues study intervention because of an AE or SAE, the AE or SAE must be recorded on the CRF and the SAE reported using the CT SAE Report Form.

Investigators are not obligated to actively seek AEs or SAEs after the participant has concluded study participation. However, if the investigator learns of any SAE, including a death, at any time after a participant has completed the study, and he/she considers the event to be reasonably related to the study intervention, the investigator must promptly report the SAE to Pfizer using the CT SAE Report Form.

#### 8.3.1.1. Reporting SAEs to Pfizer Safety

All SAEs occurring in a participant during the active collection period as described in Section 8.3.1 are reported to Pfizer Safety on the CT SAE Report Form immediately upon awareness and under no circumstance should this exceed 24 hours, as indicated in Appendix 3. The investigator will submit any updated SAE data to the sponsor within 24 hours of it being available.



090177e194745c57\Approved\Approved On: 22-Jul-2020 00:48 (GMT)

If a participant begins a new anticancer therapy, SAEs occurring during the above-indicated active collection period must still be reported to Pfizer Safety irrespective of any intervening treatment. Note that a switch to a commercially available version of the study intervention is considered as a new anticancer therapy for the purposes of SAE reporting.

#### 8.3.1.2. Recording Nonserious AEs and SAEs on the CRF

All nonserious AEs and SAEs occurring in a participant during the active collection period, which begins after obtaining informed consent as described in Section 8.3.1 will be recorded in the AE section of the CRF.

The investigator is to record on the CRF all directly observed and all spontaneously reported AEs and SAEs reported by the participant.

If a participant begins a new anticancer therapy, the recording period for nonserious AEs ends at the time the new treatment is started; however, SAEs must continue to be recorded on the CRF during the above-indicated active collection period. Note that a switch to a commercially available version of the study intervention is considered as a new anticancer therapy for the purposes of SAE reporting.

### 8.3.2. Method of Detecting AEs and SAEs

The method of recording, evaluating, and assessing causality of AEs and SAEs and the procedures for completing and transmitting SAE reports are provided in Appendix 3.

Care will be taken not to introduce bias when detecting AEs and/or SAEs. Open-ended and nonleading verbal questioning of the participant is the preferred method to inquire about AE occurrences.

### 8.3.3. Follow-up of AEs and SAEs

After the initial AE/SAE report, the investigator is required to proactively follow each participant at subsequent visits/contacts. For each event, the investigator must pursue and obtain adequate information until resolution, stabilization, the event is otherwise explained, or the participant is lost to follow-up (as defined in Section 7.3).

In general, follow-up information will include a description of the event in sufficient detail to allow for a complete medical assessment of the case and independent determination of possible causality. Any information relevant to the event, such as concomitant medications and illnesses, must be provided. In the case of a participant death, a summary of available autopsy findings must be submitted as soon as possible to Pfizer Safety.

Further information on follow-up procedures is given in Appendix 3.

### 8.3.4. Regulatory Reporting Requirements for SAEs

Prompt notification by the investigator to the sponsor of an SAE is essential so that legal obligations and ethical responsibilities towards the safety of participants and the safety of a study intervention under clinical investigation are met.



090177e194745c57\Approved\Approved On: 22-Jul-2020 00:48 (GMT)

The sponsor has a legal responsibility to notify both the local regulatory authority and other regulatory agencies about the safety of a study intervention under clinical investigation. The sponsor will comply with country-specific regulatory requirements relating to safety reporting to the regulatory authority, IRBs/ethics committees, and investigators.

Investigator safety reports must be prepared for SUSARs according to local regulatory requirements and sponsor policy and forwarded to investigators as necessary.

An investigator who receives SUSARs or other specific safety information (eg, summary or listing of SAEs) from the sponsor will review and then file it along with the SRSD(s) for the study and will notify the IRB/ethics committee, if appropriate according to local requirements.

### 8.3.5. Exposure During Pregnancy or Breastfeeding, and Occupational Exposure

Exposure to the study intervention under study during pregnancy or breastfeeding and occupational exposure are reportable to Pfizer Safety within 24 hours of investigator awareness.

#### 8.3.5.1. Exposure During Pregnancy

An EDP occurs if:

- A female participant is found to be pregnant while receiving or after discontinuing study intervention.
- A male participant who is receiving or has discontinued study intervention exposes a female partner prior to or around the time of conception.
- A female is found to be pregnant while being exposed or having been exposed to study intervention due to environmental exposure. Below are examples of environmental exposure during pregnancy:
  - A female family member or healthcare provider reports that she is pregnant after having been exposed to the study intervention by inhalation or skin contact.
  - A male family member or healthcare provider who has been exposed to the study intervention by inhalation or skin contact then exposes his female partner prior to or around the time of conception.

The investigator must report EDP to Pfizer Safety within 24 hours of the investigator's awareness, irrespective of whether an SAE has occurred. The initial information submitted should include the anticipated date of delivery (see below for information related to termination of pregnancy).

- If EDP occurs in a participant or a participant's partner, the investigator must report this information to Pfizer Safety on the CT SAE Report Form and an EDP Supplemental Form, regardless of whether an SAE has occurred. Details of the



090177e194745c57\Approved\Approved On: 22-Jul-2020 00:48 (GMT)

pregnancy will be collected after the start of study intervention and until 6 months after the last dose.

- If EDP occurs in the setting of environmental exposure, the investigator must report information to Pfizer Safety using the CT SAE Report Form and EDP Supplemental Form. Since the exposure information does not pertain to the participant enrolled in the study, the information is not recorded on a CRF; however, a copy of the completed CT SAE Report Form is maintained in the investigator site file.

Follow-up is conducted to obtain general information on the pregnancy and its outcome for all EDP reports with an unknown outcome. The investigator will follow the pregnancy until completion (or until pregnancy termination) and notify Pfizer Safety of the outcome as a follow-up to the initial EDP Supplemental Form. In the case of a live birth, the structural integrity of the neonate can be assessed at the time of birth. In the event of a termination, the reason(s) for termination should be specified and, if clinically possible, the structural integrity of the terminated fetus should be assessed by gross visual inspection (unless preprocedure test findings are conclusive for a congenital anomaly and the findings are reported).

Abnormal pregnancy outcomes are considered SAEs. If the outcome of the pregnancy meets the criteria for an SAE (ie, ectopic pregnancy, spontaneous abortion, intrauterine fetal demise, neonatal death, or congenital anomaly in a live-born baby, a terminated fetus, an intrauterine fetal demise, or a neonatal death), the investigator should follow the procedures for reporting SAEs. Additional information about pregnancy outcomes that are reported to Pfizer Safety as SAEs follows:

- Spontaneous abortion including miscarriage and missed abortion;
- Neonatal deaths that occur within 1 month of birth should be reported, without regard to causality, as SAEs. In addition, infant deaths after 1 month should be reported as SAEs when the investigator assesses the infant death as related or possibly related to exposure to the study intervention.

Additional information regarding the EDP may be requested by the sponsor. Further follow-up of birth outcomes will be handled on a case-by-case basis (eg, follow-up on preterm infants to identify developmental delays). In the case of paternal exposure, the investigator will provide the participant with the Pregnant Partner Release of Information Form to deliver to his partner. The investigator must document in the source documents that the participant was given the Pregnant Partner Release of Information Form to provide to his partner.

#### 8.3.5.2. Exposure During Breastfeeding

An exposure during breastfeeding occurs if:

- A female participant is found to be breastfeeding while receiving or after discontinuing study intervention.



090177e194745c57\Approved\Approved On: 22-Jul-2020 00:48 (GMT)

- A female is found to be breastfeeding while being exposed or having been exposed to study intervention (ie, environmental exposure). An example of environmental exposure during breastfeeding is a female family member or healthcare provider who reports that she is breastfeeding after having been exposed to the study intervention by inhalation or skin contact.

The investigator must report exposure during breastfeeding to Pfizer Safety within 24 hours of the investigator's awareness, irrespective of whether an SAE has occurred. The information must be reported using the CT SAE Report Form. When exposure during breastfeeding occurs in the setting of environmental exposure, the exposure information does not pertain to the participant enrolled in the study**,** so the information is not recorded on a CRF. However, a copy of the completed CT SAE Report Form is maintained in the investigator site file.

An exposure during breastfeeding report is not created when a Pfizer drug specifically approved for use in breastfeeding women (eg, vitamins) is administered in accord with authorized use. However, if the infant experiences an SAE associated with such a drug, the SAE is reported together with the exposure during breastfeeding.

#### 8.3.5.3. Occupational Exposure

An occupational exposure occurs when a person receives unplanned direct contact with the study intervention, which may or may not lead to the occurrence of an AE. Such persons may include healthcare providers, family members, and other roles that are involved in the trial participant's care.

The investigator must report occupational exposure to Pfizer Safety within 24 hours of the investigator's awareness, regardless of whether there is an associated SAE. The information must be reported using the CT SAE Report Form. Since the information does not pertain to a participant enrolled in the study, the information is not recorded on a CRF; however, a copy of the completed CT SAE Report Form is maintained in the investigator site file.

### 8.3.6. Cardiovascular and Death Events

Not applicable.

### 8.3.7. Disease-Related Events and/or Disease-Related Outcomes Not Qualifying as AEs or SAEs

The following DREs are common in participants with advanced CRC and can be serious/life threatening:

- Progression of Disease.

  Progression of malignancy (including fatal outcomes), if documented by use of appropriate method (for example, as per RECIST criteria for solid tumors) will be designated as progression of disease in the eCRF and should not be reported as a AEs or SAEs unless a causal relationship to study intervention is suspected.

- Elective hospitalization for treatment of disease under study.



090177e194745c57\Approved\Approved On: 22-Jul-2020 00:48 (GMT)

Because these events are typically associated with the disease under study, they will not be reported according to the standard process for expedited reporting of SAEs even though the event may meet the definition of an SAE. These events will be recorded on the corresponding CRF page in the participant's CRF within the appropriate time frame. These DREs will be monitored by a safety review team on a routine basis.

NOTE: However, if either of the following conditions applies, then the event must be recorded and reported as an SAE (instead of a DRE):

- The event is, in the investigator's opinion, of greater intensity, frequency, or duration than expected for the individual participant.

OR

- The investigator considers that there is a reasonable possibility that the event was related to study intervention.

### 8.3.8. Adverse Events of Special Interest

Not applicable.

#### 8.3.8.1. Lack of Efficacy

Lack of efficacy is reportable to Pfizer Safety only if associated with an SAE.

### 8.3.9. Medical Device Deficiencies

Not applicable.

### 8.3.10. Medication Errors

Medication errors may result from the administration or consumption of the study intervention by the wrong participant, or at the wrong time, or at the wrong dosage strength.

Exposures to the study intervention under study may occur in clinical trial settings, such as medication errors.

| **Safety Event** | **Recorded on the CRF** | **Reported on the CT SAE Report Form to Pfizer Safety Within 24 Hours of Awareness** |
|---|---|---|
| Medication errors | All (regardless of whether associated with an AE) | Only if associated with an SAE |

Medication errors include:

- Medication errors involving participant exposure to the study intervention;

- Potential medication errors or uses outside of what is foreseen in the protocol that do or do not involve the study participant.



090177e194745c57\Approved\Approved On: 22-Jul-2020 00:48 (GMT)

Such medication errors occurring to a study participant are to be captured on the medication error page of the CRF, which is a specific version of the AE page.

In the event of a medication dosing error, the sponsor should be notified within 24 hours.

Whether or not the medication error is accompanied by an AE, as determined by the investigator, the medication error is recorded on the medication error page of the CRF and, if applicable, any associated AE(s), serious and nonserious, are recorded on the AE page of the CRF.

Medication errors should be reported to Pfizer Safety within 24 hours on a CT SAE Report Form **only when associated with an SAE.**

## 8.4. Treatment of Overdose

For this study, any dose of study intervention greater than the protocol-specified dose and schedule (see Section 6.1.2) will be considered an overdose.

The Sponsor does not recommend specific treatment for an overdose.

In the event of an overdose, the investigator should:

1. Contact the medical monitor within 24 hours.
2. Closely monitor the participant for any AEs/SAEs and laboratory abnormalities until study intervention can no longer be detected systemically (at least 100 days for an overdose of bevacizumab; at least 28 days for all other study interventions).
3. Document the quantity of the excess dose as well as the duration of the overdose in the CRF.
4. Overdose is reportable to Safety **only when associated with an SAE.**

Decisions regarding dose interruptions or modifications will be made by the investigator in consultation with the medical monitor based on the clinical evaluation of the participant.

## 8.5. Pharmacokinetics

Blood samples of up to approximately ~7 mL will be collected from all participants in the SLI for measurement of plasma concentrations of encorafenib and LHY746 and irinotecan and SN-38 or oxaliplatin (total platinum and platinum in plasma ultrafiltrate) as specified in Table 25 and Table 26. Pre-dose blood samples (~2 mL) prior to encorafenib administration will be collected on Day 1 of Cycle 1 through 6 from all participants in Phase 3 Arm A and Arm B for plasma concentrations of encorafenib and LHY746. Instructions for the collection and handling of PK samples will be provided in the laboratory manual or by the sponsor. The actual date and time (24-hour clock time) of each sample will be recorded.

090177e194745c57\Approved\Approved On: 22-Jul-2020 00:48 (GMT)

### Table 25. PK Sampling Schedule for Cohort 1 (EC + FOLFIRI) in SLI

| Visit | Sampling Time | Irinotecan PK Sample (3 mL Blood) | Encorafenib PK Sample (2 mL Blood) |
|---|---|---|---|
| Cycle 1 Day 1 | 0 h (predose[b]) | X | |
| | 0.75 h | X | |
| | 1.5 h | X[c] | |
| | 2.5 h | X | |
| | 3.5 h | X | |
| | 5.5 h | X | |
| | 7.5 h | X | |
| Cycle 1 Day 3 | Predose[d] | X | |
| Cycle 1 Day 15[a] | 0 h (predose[d]) | X | X |
| | 0.75 h | X | X |
| | 1.5 h | X[c] | X |
| | 2.5 h | X | X |
| | 3.5 h | X | X |
| | 5.5 h | X | X |
| | 7.5 h | X | X |
| Cycle 1 Day 17 | Predose[d] | X | X |
| Day 1 of Cycle 2 – 6 | Predose[d] | X | X |

a Encorafenib will be administered within 5 minutes before the start of irinotecan infusion
b Predose is within 30 minutes prior to irinotecan infusion
c Immediately before irinotecan infusion ends
d Predose is within 30 minutes prior to encorafenib

090177e194745c57\Approved\Approved On: 22-Jul-2020 00:48 (GMT)

090177e194745c57\Approved\Approved On: 22-Jul-2020 00:48 (GMT)

### Table 26. PK Sampling Schedule for Cohort 2 (EC + mFOLFOX6) in SLI

| Visit | Sampling Time | Oxaliplatin PK Sample (5 mL Blood) | Encorafenib PK Sample (2 mL Blood) |
|---|---|---|---|
| Cycle 1 Day 1[a] | 0 h (predose[b]) | X | X |
| | 1 h | X | X |
| | 2 h | X[c] | X |
| | 3 h | X | X |
| | 4 h | X | X |
| | 6 h | X | X |
| | 8 h | X | X |
| Cycle 1 Day 3 | Predose[b] | X | X |
| Cycle 1 Day 15[a] | 0 h (predose[b]) | X | X |
| | 1 h | X | X |
| | 2 h | X[c] | X |
| | 3 h | X | X |
| | 4 h | X | X |
| | 6 h | X | X |
| | 8 h | X | X |
| Cycle 1 Day 17 | Predose[b] | X | X |
| Day 1 of Cycle 2 – 6 | Predose[b] | X | X |

a Encorafenib will be administered within 5 minutes before the start of oxaliplatin infusion
b Predose is within 30 minutes prior to encorafenib
c Immediately before oxaliplatin infusion ends

The actual times may vary but the number of samples will remain the same. All efforts should be made to obtain the samples at the exact nominal time relative to dosing. Collection of samples up to and including 8 hours after dose administration that are obtained within 10% of the nominal time relative to dosing (eg, within 6 minutes of a 60-minute sample) will not be captured as a protocol deviation, as long as the exact time of the collection is noted on the source document and the eCRF.

Samples collected for analyses of study intervention-plasma -concentration may also be used to evaluate safety or efficacy aspects related to concerns arising during or after the study, for metabolite identification and/or evaluation of the bioanalytical method, or for other internal exploratory purposes.

Genetic analyses will not be performed on these plasma samples.

Samples collected for measurement of plasma concentrations of encorafenib and a metabolite LHY746, irinotecan and a metabolite SN-38, or oxaliplatin will be analyzed using validated analytical methods in compliance with applicable SOPs. Exploratory metabolite

identification or quantitation may be performed using either validated or exploratory methods.

The PK samples must be processed and shipped as indicated in the instructions provided to the investigator site to maintain sample integrity. Any deviations from the PK sample handling procedure (eg, sample collection and processing steps, interim storage or shipping conditions), including any actions taken, must be documented and reported to the sponsor. On a case-by-case basis, the sponsor may make a determination as to whether sample integrity has been compromised.

Any changes in the timing or addition of time points for any planned study assessments must be documented and approved by the relevant study team member and then archived in the sponsor and site study files, but will not constitute a protocol amendment. The IRB/ethics committee will be informed of any safety issues that require alteration of the safety monitoring scheme or amendment of the ICD.

### 8.5.1. Pharmacokinetics for Participants in China

For participants randomized to Arm A or Arm B in China, pre-dose blood samples (~2 mL) prior to encorafenib administration will be collected on Day 1 of Cycle 1 through 6 for plasma concentrations of encorafenib and LHY746.

Up to the first 16 participants ≥18 years old randomized to Arm A in China (to obtain approximately 12 PK evaluable participants) will undergo a more intensive PK sampling, including serial whole blood samples (2 mL) collected on Day 1 and Day 15 of Cycle 1 at pre-dose and at 0.5, 1, 2, 3, 4, 5, 6, 8 and 24 hours post-dose to assess plasma concentration of encorafenib and its metabolite LHY746. If a dose interruption occurs prior to Cycle 1 Day 15, the intensive PK sampling on Cycle 1 Day 15 is suggested to be changed to Day 1 or Day 15 of Cycle 2. Participants may be skipped if they are determined by the investigator to be ineligible for intensive PK sampling. Additional informed consent should be documented before intensive PK sample collection.

## 8.6. Pharmacodynamics

Biomarker samples may be used for exploratory PD analyses (see Section 8.8).

Pre-dose blood samples will be collected to enable measurements which may include but are not limited to: cfDNA, proteomic profiles, or epigenetics at the timepoints specified in the SoA.

As part of understanding the PD of the investigational product, samples may be used for evaluation of the bioanalytical method, as well as for other internal exploratory purposes. These data may not be included in the CSR.

Some samples may be analyzed using a validated analytical method in compliance with applicable SOPs while others may be analyzed using a non-characterized assay (non-validated).



090177e194745c57\Approved\Approved On: 22-Jul-2020 00:48 (GMT)

The pharmacodynamic samples must be processed and shipped as indicated in the instructions provided to the investigator site to maintain sample integrity. Any deviations from the pharmacodynamic sample handling procedure (eg, sample collection and processing steps, interim storage or shipping conditions), including any actions taken, must be documented and reported to the sponsor. On a case-by-case basis, the sponsor may make a determination as to whether sample integrity has been compromised.

## 8.7. Genetics

### 8.7.1. Specified Genetics

Genetics (specified analyses) are not evaluated in this study.

### 8.7.2. *BRAF* Testing

Participants will be eligible for the study based on identification of a *BRAF* V600E mutation in the tumor as determined by the central laboratory as part of Molecular Prescreening for the trial or by a local assay result from either blood or tumor tissue biopsy obtained any time prior to Screening (see Figure 1). Only PCR and NGS-based local assays results will be acceptable. If the participant is enrolled based on local assay results (using either tumor tissue or blood), the *BRAF* mutation status must be confirmed by the central laboratory (using tumor tissue only) no later than 30 days from first dose of study treatment. Either archival tumor tissue or newly collected fixed biopsy sample is required for the assessment of *BRAF* V600E mutation by the Sponsor designated central laboratory(ies).

In cases where there is discordance between the local assay and central laboratory results, or if the central laboratory is not able to confirm presence of a *BRAF* V600E mutation due to inadequate or poor sample condition within 30 days of initiating study therapy, participants may only continue treatment if there is no clinical indication of deterioration or disease progression and the investigator determines that the participant is deriving benefit. In such instances, participants must be informed that the *BRAF* mutation status is unconfirmed and must acknowledge on the ICD that includes this information and describes alternative treatment options.

Central laboratory *BRAF* mutation tests with a definitive result (positive or negative) cannot be repeated to resolve a discordant result. Participants whose sample is determined to be inadequate or who have an indeterminate result on central testing may have samples resubmitted for testing. Lack of *BRAF* V600E confirmation by the central laboratory may be due to discordance between the local assay and central laboratory results (potential false positive local assay results), or due to inadequate or poor sample condition for central testing (indeterminate results). If at any time in the study there is lack of *BRAF* V600E confirmation in a total of 6% of the total planned enrollment of the Phase 3 portion of the trial (52 participants) or discordance between the local assay and the central laboratory in 3% of the total planned enrollment (26 participants), all subsequent participants will be required to have *BRAF* V600E determined by the central laboratory for enrollment (ie, local *BRAF* testing will no longer be accepted for trial eligibility). Information regarding sites and laboratories associated with discordant results will be maintained and results from laboratories with more than 1 prior discordant result will not be accepted for participant enrollment. Sites with more



090177e194745c57\Approved\Approved On: 22-Jul-2020 00:48 (GMT)

than 2 randomized participants having indeterminate results will be required to enroll all subsequent participants based only on central laboratory assay results.

As the central laboratory's diagnostic test may be more sensitive and specific than local testing, tumor samples previously determined to be *BRAF*-wt by local assessment may be submitted to the central laboratory. In particular, tumors with clinicopathological features of *BRAF* mutations such as right colon tumors; poorly differentiated, mucinous, or signet-ring carcinomas; or tumors metastasized to the peritoneum,[60] should be considered for testing by central laboratory regardless of the results of prior local *BRAF* mutation testing.

**Figure 1: *BRAF* Testing**

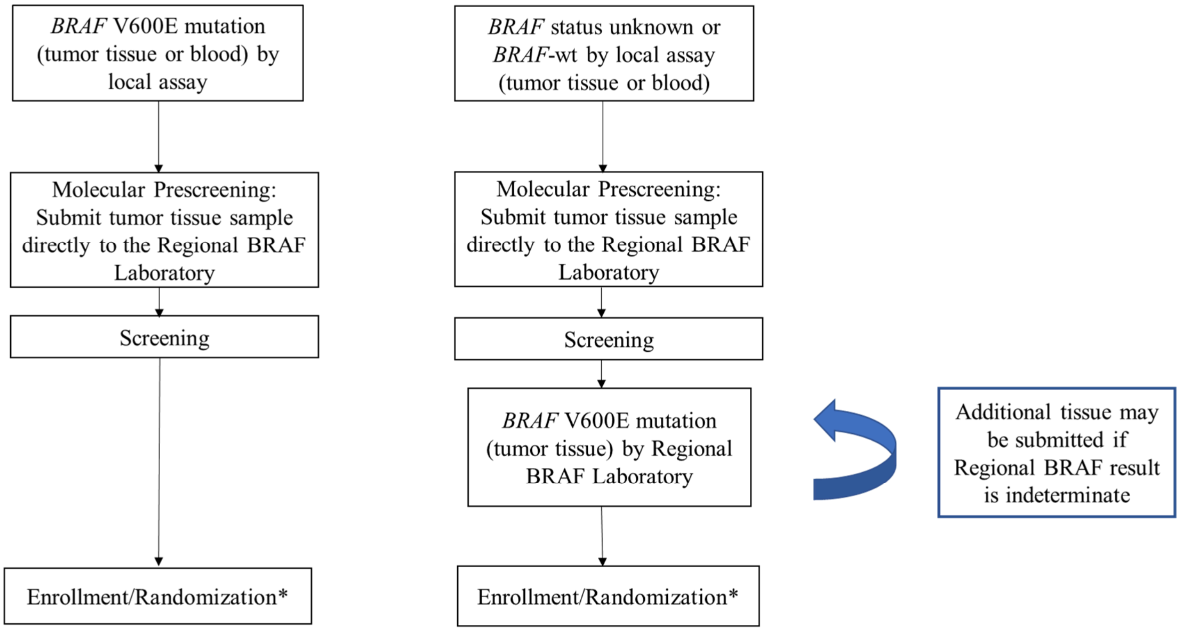


*Confirmation of *BRAF* V600E by Regional BRAF Laboratory required within 30 days of starting study intervention

### 8.7.3. Molecular Prescreening

Molecular Prescreening can be performed at any time prior to Screening. An informed consent/assent must be signed prior to any Molecular Prescreening procedure for confirmation of *BRAF* V600E status. A FFPE tumor tissue block from the most recent tumor resection or biopsy must be provided for all participants enrolled in the study and submitted to the designated central laboratory(ies). This tissue block should be obtained from a biopsy or surgery that was performed within 2 years prior to study enrollment. If an archival tumor sample is not available, a fresh tumor biopsy must be obtained.

Slide sections must be freshly cut (ie, cut no more than 30 days prior to shipment to the central laboratory), 4-5 µm thick and mounted on positively-charged microscope slides. A minimum of 15 slides should be provided for *BRAF* mutation test and tumor tissue assessment (Section 8.8.2). Participants with fewer than 15 slides of analyzable tissue may be considered eligible if the Sponsor determines that the slides are sufficient for central testing



090177e194745c57\Approved\Approved On: 22-Jul-2020 00:48 (GMT)

of *BRAF* V600E mutation status. In participants with documented *BRAF* V600E status determined by local assay prior to the study, when possible, the same tissue source should be submitted to the central laboratory in order to minimize the potential for discordance. Tumor tissue from cytologic sampling (eg, fine needle aspiration, including FFPE cell pellet material) is not adequate and should not be submitted.

Information regarding tissue specimen requirements, sample handling and shipment will be provided in the Laboratory Manual. Participants will be registered for the study using the IRT after the informed consent/assent is obtained. Refer and comply with detailed guidelines in the IRT manual.

### 8.7.4. Screening

Participants with documented *BRAF* V600E status determined by the central laboratory during Molecular Prescreening or by local assay prior to this study must sign an ICD before additional screening procedures to determine eligibility for participation in the study are performed. A copy of the ICD will be given to the participant or their legal representative. The date that informed consent/assent was obtained must be documented in source documents.

Participant eligibility will be verified against the inclusion and exclusion criteria once all Screening procedures are completed. The eligibility check will be embedded in the IRT system.

### 8.7.5. Companion Diagnostic for Eligibility

Participants are required to have their disease assessed for a *BRAF* V600E mutation based on testing de novo or archival tumor tissue using the QIAGEN *therascreen*® BRAF V600E RGQ PCR Kit (FDA approved [USA]; CE marked for use in Europe) performed by a study-sponsored CAP/CLIA-certified central clinical laboratory. Testing may be performed during Molecular Prescreening or Screening.

Details on processes for collection and shipment of these samples can be found in the laboratory manual.

Although it is anticipated that most samples will be consumed by planned analyses during the course of the study, any unused samples may be stored at a facility selected by the sponsor for a maximum of 10 years (or according to local regulations) following the last participant's last visit for the study unless prohibited by local regulations or ethics committee decision.

### 8.7.6. Companion Diagnostic Assay Development

An in vitro diagnostic assay may be developed in accordance with FDA's Quality System Regulations and Design Control requirements. Any tumor samples remaining after testing for *BRAF* V600E and/or a blood sample collected during Screening ("Blood sample for BRAF CDx" – 20 mL of blood optimized for plasma preparation) may be used for companion diagnostic development and Health Authority registration, and to assess the concordance between the results for *BRAF* V600E status obtained using the central laboratory assay to

090177e194745c57\Approved\Approved On: 22-Jul-2020 00:48 (GMT)

determine eligibility for the study and the companion diagnostic assay which will be submitted for premarket approval.

Details on processes for collection and shipment of these samples can be found in the laboratory manual.

Although it is anticipated that most samples will be consumed by planned analyses during the course of the study, any unused samples may be stored at a facility selected by the sponsor for a maximum of 10 years (or according to local regulations) following the last participant's last visit for the study unless prohibited by local regulations or ethics committee decision.

***Note***: For participants in China, sample collection and testing for *BRAF* V600E will not be performed until approval by HGRAC.

#### 8.7.7. Banked Biospecimens for Genetics

A 4 mL blood sample optimized for DNA isolation [Prep D1] will be collected at the time specified in the Schedule of Activities as local regulations and IRBs/ethics committees allow.

Banked Biospecimens may be used for research related to the study intervention(s) and cancer. Genes and other analytes (eg, proteins, RNA, nondrug metabolites) may be studied using the banked samples.

See Appendix 5 for information regarding genetic research. Details on processes for collection and shipment of these samples can be found in the lab manual.

***Note***: Banked biospecimens are not applicable for participants in China.

### 8.8. Biomarkers

Unless prohibited by local regulations or ethics committee decision, the following samples for biomarker research are required and will be collected from all participants in this study within the SLI and Phase 3 portions as specified in the SoA:

- Approximately 2 mL blood sample at designated time points to measure levels of CRP and CEA.
- Pre-treatment tumor tissue samples to enable genomic profiling (eg *RAS* mutations, MSI status) and expression analyses (eg gene, protein).
- Approximately 20 mL blood samples at designated time points to be processed to plasma for assessment of cfDNA.
- Approximately 10 mL blood samples at designated time points to be processed to serum for proteomic profiling.
- Approximately 4 mL whole blood samples at baseline for filtering of somatic mutation data.

090177e194745c57\Approved\Approved On: 22-Jul-2020 00:48 (GMT)

- ***Optional:*** Tumor tissue samples at progression/EOT for assessment of tumoral changes which may occur over the course of therapy, including potential acquired resistance mechanisms.

***Note***: For participants in China, sample collection and biomarker testing will not be performed until approval by HGRAC.

### 8.8.1. CRP and CEA assessments

Blood samples (2 mL) will be collected to measure levels of CRP and CEA using routine clinical laboratory testing. These analyses will be used to explore relationships between levels of CRP and CEA and clinical outcome.

### 8.8.2. Tumor Tissue Assessments

Tumor biospecimens representing FFPE tissue samples from tumor resection or biopsy will be used to analyze candidate DNA, RNA, or protein markers, or relevant signature of markers for their ability to identify those participants who are most likely to benefit from treatment with the study intervention.

Tumor tissue requirements are included in Section 8.7.2 and further details are provided in the Laboratory Manual for mandatory pre-screening/screening tumor samples.

An optional biopsy at End of Treatment is requested except in instances where the procedure poses unacceptable risks per Investigator documentation. The End of Treatment tumor biopsy should be performed before initiation of subsequent anti-cancer therapy and preferably no later than 7 days after the End of Treatment visit. Tumor tissue requirements/procedures are same as for mandatory pre-screening/screening tumor samples (Section 8.7.2) and further details are provided in the Laboratory Manual.

Targeted and/or whole exome/genome sequencing and/or transcriptome and/or epigenetic analyses will be performed using the remainder of the de novo or archival tumor tissue samples submitted at prescreening or screening for eligibility assessment to evaluate the genomic landscape (eg, *RAS* mutations, MSI status, etc) within the participant population. Additionally, these samples and tumor tissue collected at progression/EOT (when available) will be used to gain insight into the mechanisms that confirm sensitivity/resistance to study treatment. The results of these analyses may be combined with the results from targeted genomic analyses performed for enrollment eligibility assessment to further explore correlations of molecular profiling with response to study treatment. In addition, these samples may be used to help support development of a potential tumor tissue diagnostic test for encorafenib.

Unless prohibited by local regulations or ethics committee decision, whole blood samples (4 mL) will be collected from all participants on Day 1 before the first dose of study treatment (Cycle 1 Day 1) for exploratory targeted and/or whole exome/genome sequencing. These samples will be used to characterize the germline status of genetic variants (eg, DDR genes, MMR genes, etc). They will not otherwise be used to generate free-standing germline sequencing results, but rather as controls to assist in identifying and profiling somatic tumor



090177e194745c57\Approved\Approved On: 22-Jul-2020 00:48 (GMT)

DNA mutations. These samples are required for all enrolled participants where tumor tissue for genomic screening was collected from same participant.

### 8.8.3. Circulating Free DNA (cfDNA)

Blood samples (20 mL) for cfDNA analyses will be collected from all participants at timepoints specified in the SoA and may also include remnant sample from "Blood sample for *BRAF* CDx". These samples will be used to assess genetic changes (eg, *BRAF*, *KRAS*, *NRAS*, etc) in cfDNA to evaluate ctDNA burden (as assessed by variant allele fraction) with response to study treatment, potentially explore correlations with tumor molecular profiling and to understand potential mechanisms of acquired resistance to study treatment. A targeted sequencing panel may be used for these analyses, which may include DDR genes and MSI status. Exploratory whole exome/genome sequencing and/or epigenetic analyses may be performed. In addition, these samples may be used to help support development of a potential blood-based test for encorafenib.

Although it is anticipated that most samples will be consumed by planned analyses during the course of the study, any unused samples may be stored at a facility selected by the sponsor for a maximum of 10 years (or according to local regulations) following the last participant's last visit for the study.

### 8.8.4. Specified Gene Expression (RNA) Research

An FFPE tissue sample for RNA isolation will be collected as noted in Section 8.8.2. The samples will be analyzed by targeted and/or transcriptomic profiling to evaluate gene expression levels and may be used to identify signatures associated with response, resistance and adverse events in response to treatment

Details on processes for collection and shipment of these samples can be found in the laboratory manual.

Although it is anticipated that most samples will be consumed by planned analyses during the course of the study, any unused samples may be stored at a facility selected by the sponsor for a maximum of 10 years (or according to local regulations) following the last participant's last visit for the study.

### 8.8.5. Specified Protein Research

Approximately 10 mL blood sample (processed for approximately 5 mL of serum) for protein analyses will be collected from all participants at the timepoints specified in the SoA. The sample(s) may be analyzed for proteomic signatures associated with response or resistance to therapy in pretreatment and on-treatment samples. This sample may also be used for additional research based on emerging knowledge of CRC biology.

Details on processes for collection and shipment of these sample(s) can be found in the laboratory manual.

090177e194745c57\Approved\Approved On: 22-Jul-2020 00:48 (GMT)

Although it is anticipated that most samples will be consumed by planned analyses during the course of the study, any unused samples may be stored at a facility selected by the sponsor for a maximum of 10 years (or according to local regulations) following the last participant's last visit for the study.

### 8.8.6. Specified Metabolomic Research

Specified metabolomic research is not included in this study.

## 8.9. Immunogenicity Assessments

Immunogenicity assessments are not included in this study.

## 8.10. Health Economics

Health economic parameters will be captured as part of the trial to understand overall and differential healthcare resource utilization associated with each treatment and the Control Arm. Both direct medication and direct non-medical resource use elements will be captured. Resource use consumption will be reported for both scheduled and unscheduled visits, covering health care resource use for disease monitoring, treatment monitoring, and management of treatment-emergent adverse events. Resource use for scheduled visits will be captured via the eCRF. Resource use for unscheduled visits will be captured via eCRF or study questionnaire and will include the timing and reason for the visit, along with any tests, procedures or medications received. If hospital admission has occurred, additional information such as reason for admission, length of stay may be collected. These data may be supplemented with medical records. All information will be kept confidential and will be used exclusively for the study purpose. All records will be de-identified with individual's records labeled only with the assigned subject study identification number.

# 9. STATISTICAL CONSIDERATIONS

Detailed methodology for summary and statistical analyses of the data collected in this study is outlined here and further detailed in a SAP, which will be maintained by the sponsor. The SAP may modify what is outlined in the protocol where appropriate; however, any major modifications of the primary endpoint definitions or their analyses will also be reflected in a protocol amendment.

## 9.1. Estimands and Statistical Hypotheses

### 9.1.1. Estimands

Estimands are defined below for the primary endpoint of incidence of DLTs in the SLI as well as the primary and key secondary endpoints in the Phase 3 portion of the study.

090177e194745c57\Approved\Approved On: 22-Jul-2020 00:48 (GMT)

**Safety Lead-in**

Primary Estimand (DLT):

- Population: all participants with local or central laboratory confirmed *BRAF* V600E-mCRC, as defined by the screening inclusion/exclusion criteria in Section 5.1.2 and Section 5.2.2 to reflect the targeted population of the SLI portion of the study.
- Variable: DLT rate during the DLT-evaluation period, which is the first 28 days after the first dose of study intervention in the SLI. DLTs are defined in Section 6.1.1.1.
- Intercurrent events: hypothetical strategy will be applied for the intercurrent events. The intercurrent event is treatment discontinuation for reasons other than treatment-related toxicity that leads to <75% of the planned dose of each study intervention during the DLT evaluation period. Participants without DLTs and with the intercurrent event will not be included in the DLT rate calculation.
- Population-level summary measure: DLT rate defined as the number of DLT-evaluable participants with DLTs in the DLT-evaluation period divided by the number of DLT-evaluable participants.

**Phase 3**

Primary Estimand (PFS):

- Population: all participants with either a local or a central laboratory confirmed result of *BRAF* V600E-mutant mCRC, as defined by the screening inclusion/exclusion criteria in Section 5.1.2 and Section 5.2.2 to reflect the targeted population of the Phase 3 portion of the study.
- Variable: PFS by BICR, defined as the time from the date of randomization to the earliest documented disease progression per RECIST v1.1, or death due to any cause.
- Intercurrent events: hypothetical strategy will be applied for the intercurrent events. Details of the intercurrent events and censoring rules for the primary analysis are summarized in Table 27.
- Population-level summary measure: hazard ratio for PFS and corresponding 2-sided CI based on Cox proportional hazard model stratified by ECOG performance status at randomization and region. PFS will be compared between the two treatment arms using a 1-sided stratified log-rank test.

Supportive Estimand 1 (PFS):

- Population: all participants with a central laboratory confirmed result of *BRAF* V600E-mutant mCRC, as defined by the screening inclusion/exclusion criteria in Section 5.1.2 and Section 5.2.2 to reflect the targeted population of the Phase 3 portion of the study.
- Variable: same as primary estimand of PFS.
- Intercurrent events: same as primary estimand of PFS.



090177e194745c57\Approved\Approved On: 22-Jul-2020 00:48 (GMT)

- Population-level summary measure: same as primary estimand of PFS.

Supportive Estimand 2 (PFS):

- Population: same as primary estimand of PFS.
- Variable: same as primary estimand of PFS.
- Intercurrent events: treatment policy strategy will be applied for the intercurrent event of starting new anti-cancer therapy or undergoing metastasectomy procedures. All other intercurrent events will be addressed in the same approach as the primary estimand of PFS. Table 28 summarizes the details of the intercurrent events and censoring rules for this supportive analysis.
- Population-level summary measure: same as primary estimand of PFS.

Key Secondary Estimand (OS):

- Population: all participants with local or central laboratory confirmed *BRAF* V600E-mCRC, as defined by the screening inclusion/exclusion criteria in Section 5.1.2 and Section 5.2.2 to reflect the targeted population of the Phase 3 portion of the study.
- Variable: OS, defined as the time from the date of randomization to death due to any cause.
- Intercurrent events: hypothetical strategy will be applied for the intercurrent events of lost to follow-up or withdrawal of consent/assent. Participants not known to have died are censored at the date of last contact.
- Population-level summary measure: hazard ratio for OS and corresponding 2-sided CI based on Cox proportional hazard model stratified by ECOG performance status at randomization and region. OS will be compared between the two treatment arms using a 1-sided stratified log-rank test.

Supportive Secondary Estimand 1 (OS):

- Population: all participants with a central laboratory confirmed result of *BRAF* V600E-mutant mCRC, as defined by the screening inclusion/exclusion criteria in Section 5.1.2 and Section 5.2.2 to reflect the targeted population of the Phase 3 portion of the study.
- Variable: same as key secondary estimand of OS.
- Intercurrent events: same as key secondary estimand of OS.
- Population-level summary measure: same as key secondary estimand of OS.

090177e194745c57\Approved\Approved On: 22-Jul-2020 00:48 (GMT)

### Table 27. PFS Outcome and Event Dates – Primary Analysis

| Situation | Date of Progression/Censoring | Outcome |
|---|---|---|
| No adequate baseline assessment | Date of randomization [a] | Censored [a] |
| PD or death ≤12 (or 16) [b] weeks after last adequate tumor assessment or ≤12 weeks after date of randomization | Date of PD or death | Event |
| PD or death >12 (or 16) [b] weeks after the last adequate tumor assessment [c] | Date of last adequate tumor assessment [c] documenting no PD prior to new anti-cancer therapy, metastasectomy procedures or missed assessments | Censored |
| No PD | | |
| New anti-cancer therapy given or undergoing metastasectomy procedures | | |

a If the participant dies ≤12 weeks after date of randomization, the death is an event with date on death date.

b Durations are equal to 2 times the length of the tumor assessment interval, which is 12 weeks for the first 18 months after randomization, and 16 weeks thereafter.

c If there are no adequate post-baseline assessments prior to the PD or death, then the time without adequate assessment should be measured from the date of randomization; if the criteria is met, the censoring will be on the date of randomization.

### Table 28. PFS Outcome and Event Dates – Supportive Analysis

| Situation | Date of Progression/Censoring | Outcome |
|---|---|---|
| No adequate baseline assessment | Date of randomization [a] | Censored [a] |
| PD or death ≤12 (or 16) [b] weeks after last adequate tumor assessment or ≤12 weeks after date of randomization | Date of PD or death | Event |
| PD or death >12 (or 16) [b] weeks after the last adequate tumor assessment [b] | Date of last adequate tumor assessment [c] documenting no PD prior to missed assessments | Censored |
| No PD | | |

a If the participant dies ≤12 weeks after date of randomization, the death is an event with date on death date.

b Durations are equal to 2 times the length of the tumor assessment interval, which is 12 weeks for the first 18 months after randomization, and 16 weeks thereafter.

c If there are no adequate post- baseline assessments prior to the PD or death, then the time without adequate assessment should be measured from the date of randomization; if the criteria were met the censoring will be on the date of randomization.

090177e194745c57\Approved\Approved On: 22-Jul-2020 00:48 (GMT)

### 9.1.2. Statistical Hypotheses

There is no statistical hypothesis for the SLI portion of the study.

For Phase 3 portion of the study, the primary objectives are to compare the efficacy, as measured by the primary endpoint of PFS by BICR, of Arm A versus the Control Arm and of Arm B versus the Control Arm. The following statistical hypotheses will be tested to address the primary objectives:

$H_{01}$: $HR_{PFS\ (A\ versus\ Control)} \geq 1$ versus $H_{11}$: $HR_{PFS\ (A\ versus\ Control)} < 1$

$H_{02}$: $HR_{PFS\ (B\ versus\ Control)} \geq 1$ versus $H_{12}$: $HR_{PFS\ (B\ versus\ Control)} < 1$

where $HR_{PFS\ (A\ versus\ Control)}$ is the hazard ratio for PFS of Arm A versus the Control Arm and $HR_{PFS\ (B\ versus\ Control)}$ is the hazard ratio for PFS of Arm B versus the Control Arm.

The key secondary objectives are to compare the efficacy, as measured by the key secondary endpoint of OS, of Arm A versus the Control Arm and of Arm B versus Control. The following statistical hypotheses will be tested to address the key secondary objectives:

$H_{03}$: $HR_{OS\ (A\ versus\ Control)} \geq 1$ versus $H_{13}$: $HR_{OS\ (A\ versus\ Control)} < 1$

$H_{04}$: $HR_{OS\ (B\ versus\ Control)} \geq 1$ versus $H_{14}$: $HR_{OS\ (B\ versus\ Control)} < 1$

where $HR_{OS\ (A\ versus\ Control)}$ is the hazard ratio for OS of Arm A versus the Control Arm and $HR_{OS\ (B\ versus\ Control)}$ is the hazard ratio for OS of Arm B versus the Control Arm.

A graphical multiple testing strategy[61] will be used to strongly control the family-wise error rate of the study at 0.025 (1-sided) level. Details of this strategy are described in Section 9.4.1.1. The study will be considered positive (ie, demonstrated evidence of effectiveness) if at least one of the hypothesis tests of the primary endpoint is statistically significant.

## 9.2. Sample Size Determination

The planned sample size of the study is approximately 930 participants including approximately 60 participants in the SLI, and approximately 870 participants in the Phase 3 portion of the study.

### Safety Lead-in

The primary endpoint of the SLI of the study is incidence of DLTs. Up to 60 evaluable participants will be enrolled on a rolling basis in an alternating manner to receive either EC + mFOLFOX6 or EC + FOLFIRI (up to 30 participants per cohort). The safety data will be evaluated by the SC after the first 9 evaluable participants in each cohort have been followed for a minimum of 28 days with EC + mFOLFOX6 or EC + FOLFIRI. The target DLT rate in the first 28 days is <33% (ie, <3 out of 9 evaluable participants with DLTs).

Table 29 provides a comparison of the characteristics of this DLT evaluation rule and the traditional 3 + 3 rules based on 9 participants.



090177e194745c57\Approved\Approved On: 22-Jul-2020 00:48 (GMT)

**Table 29. Characteristics of DLT Probability During the DLT Evaluation Period**

| True DLT Rate During DLT Evaluation Period | Probability of Dose Declared Toxic Using 3 + 3 Rules | Probability of Observed DLT Rate ≥33% in 9 Participants During DLT Evaluation Period |
|---|---|---|
| 10% | 0.094 | 0.053 |
| 20% | 0.291 | 0.262 |
| 30% | 0.506 | 0.537 |
| 40% | 0.691 | 0.768 |
| 50% | 0.828 | 0.910 |

The DLT evaluation period is the first 28 days after the first dose of study intervention in the SLI, and the DLT-evaluable is defined in Section 6.1.1.1.

If the doses are determined to be tolerable in the first 9 evaluable participants in either or both regimens based on the observed DLT rate (<33%) and evaluation of the overall toxicity profile, the SLI will be expanded for a total of up to 30 evaluable participants in each cohort. If the DLT rate is ≥33% in either cohort, that combination regimen will not be evaluated further in the SLI and will not be used in Phase 3.

Table 30 summarizes the probability of observing at least one instance of a toxicity that has a true incidence rate of 10% and 15% for the given number of participants.

**Table 30. Probability of Observing ≥1 Instance of Toxicity at 10% and 15% Incidence Rate**

| True Toxicity Incidence Rate | Number of Participants | Probability of Observing ≥1 Instance of Toxicity |
|---|---|---|
| 10% | 15 | 0.79 |
| | 20 | 0.88 |
| | 30 | 0.96 |
| 15% | 15 | 0.91 |
| | 20 | 0.96 |
| | 30 | 0.99 |

If any participant is deemed non-evaluable for DLT, additional participants may be enrolled to ensure there are a sufficient number of evaluable participants in the SLI.

**Phase 3**

A total of approximately 870 eligible participants will be randomized in a 1:1:1 ratio, stratified by ECOG performance status (0 versus 1) and region (US/Canada versus Europe versus Rest of World), to one of the three treatment arms (ie, approximately 290 participants/arm). It is assumed that participant accrual will reach a maximum of 40 participants per month for an accrual duration of approximately 27 months.

090177e194745c57\Approved\Approved On: 22-Jul-2020 00:48 (GMT)

Primary Endpoint

The primary analysis of PFS will occur once both of the following criteria are met: at least 393 BICR-assessed PFS events are observed in Arm A + the Control Arm and at least 313 BICR-assessed PFS events are observed in Arm B + the Control Arm. It is anticipated that this will occur approximately 34 months after the first participant is randomized. It is anticipated that approximately 380 PFS events will be observed in Arm B + the Control Arm by this time, but only the first 313 PFS events will be included in the primary analysis.

For the comparison of Arm A versus Control PFS, this design will achieve 90% power to detect a HR of 0.70 with a 1-sided alpha of 0.0125 under the assumption that PFS will follow an exponential distribution and approximately 10% of participants will be lost to follow-up. This corresponds to an improvement in the median PFS of 3 months (10 months in Arm A versus 7 months in the Control Arm). With a total of 393 BICR-assessed PFS events in Arm A + the Control Arm, the null hypothesis that the PFS in Arm A is the same or worse than the Control Arm will be rejected if the HR is smaller (ie better) than 0.80 at the primary analysis.

For the comparison of Arm B versus Control PFS, this design will achieve 90% power to detect a HR of 0.67 with a 1-sided alpha of 0.0125 under the same assumption of PFS distribution and dropout rate as Arm A. This corresponds to an improvement in the median PFS of 3.5 months (10.5 months in Arm B versus 7 months in the Control Arm). With a total of 313 BICR-assessed PFS events in Arm B + the Control Arm, the null hypothesis that the PFS in Arm B is the same or worse than the Control Arm will be rejected if the HR is smaller than 0.78 at the primary analysis.

Key Secondary Endpoint

The final analysis of OS will occur once both of the following criteria are met: at least 403 OS events are observed in Arm A + the Control Arm and at least 344 OS events are observed in Arm B + the Control Arm. This is expected to occur approximately 49 months after the first participant is randomized (ie, approximately 22 months after randomization is complete). At the time of the final analysis of OS, it is anticipated that approximately 400 OS events will be observed in Arm B + the Control Arm by this time, but only the first 344 OS events will be included.

For the comparison of Arm A versus Control OS, 403 OS events in Arm A + the Control Arm will achieve 85% power to detect an OS HR of 0.72 with a 1-sided alpha of 0.0125 under the assumption that OS will follow an exponential distribution and approximately 5% of participants will be lost to follow-up. This corresponds to an improvement in the median OS of 5.8 months (20.8 months in Arm A versus 15 months in the Control Arm). With a total of 403 OS events in Arm A + the Control Arm, the null hypothesis that the OS in Arm A is the same or worse than the Control Arm will be rejected if the HR is smaller (ie better) than 0.80 at the final analysis of OS.

For the comparison of Arm B versus Control OS, 344 OS events in Arm B + the Control Arm will achieve 85% power to detect an OS HR of 0.70 with a 1-sided alpha of 0.0125



090177e194745c57\Approved\Approved On: 22-Jul-2020 00:48 (GMT)

under the same assumption of OS distribution and dropout rate as Arm A. This corresponds to an improvement in the median OS of 6.4 months (21.4 months in Arm B versus 15 months in the Control Arm). With a total of 344 OS events in Arm B + the Control Arm, the null hypothesis that the OS in Arm B is the same or worse than the Control Arm will be rejected if the HR is smaller than 0.78 at the final analysis of OS.

## 9.3. Analysis Sets

For purposes of analysis, the following analysis sets are defined:

| Participant Analysis Set | Description |
|---|---|
| **Enrolled** | All participants who sign the Screening ICD. |
| **DLT-Evaluable Analysis Set** | All participants who receive at least 1 dose of study intervention in the SLI and either experience DLT during the DLT evaluation period or complete the DLT evaluation period without DLT. Participants without DLTs who withdraw from study treatment before receiving at least 75% of the planned dose of each study intervention during the DLT evaluation period for reasons other than treatment-related toxicity are not evaluable for DLT. The DLT evaluation period is the first 28 days after the first dose of study intervention. |
| **Full Analysis Set (FAS)** | For participants in the SLI, the FAS includes all participants who receive at least 1 dose of study intervention.<br><br>For participants in the Phase 3 portion, the FAS includes all participants who are randomized in the Phase 3 portion of the study. Participants will be analyzed according to the study treatment assigned at randomization. |
| **Centrally Assessed *BRAF* V600E Positive Analysis Set** | All participants who are randomized in the Phase 3 portion of the study with a confirmed central laboratory result of *BRAF* V600E mutation. Participants will be analyzed according to the study treatment assigned at randomization. |
| **Safety Analysis Set** | All participants who receive at least 1 dose of study intervention.<br><br>For the Phase 3 portion of the study, participants will be analyzed according to the study treatment assigned at randomization unless the incorrect treatment(s) was/were received throughout the dosing period in which case participants will be analyzed according to the actual study treatment received. |
| **Patient-Reported Outcome (PRO) Analysis Set** | All participants who are in the FAS and who completed a baseline (last PRO assessment prior to the first dose of study treatment) and at least 1 post-baseline PRO assessment. |
| **PK Analysis Set** | The PK concentration set is defined as all enrolled participants who are treated and have at least 1 analyte concentration.<br><br>The PK parameter analysis set is defined as all enrolled participants treated who have sufficient information to estimate at least 1 of the PK parameters of interest and have no major protocol deviations affecting PK assessment. |

090177e194745c57\Approved\Approved On: 22-Jul-2020 00:48 (GMT)

| | |
|---|---|
| **Biomarker Analysis Set** | All participants who are in the safety analysis set and who had at least 1 of the pharmacodynamic parameters or biomarkers evaluated at pre and/or post dose. |

## 9.4. Statistical Analyses

The SAP will be developed and finalized before any analyses are performed and will describe the analyses and procedures for accounting for missing, unused, and spurious data. This section is a summary of the planned statistical analyses of the primary and secondary endpoints.

### 9.4.1. General Considerations

All analyses will be performed by treatment arm for the SLI and Phase 3 portion of the study separately. All efficacy analyses will be performed using the FAS, and all safety analyses will be performed using the safety analysis set.

In general, descriptive summaries will be presented for the efficacy and safety variables collected. Continuous variables will be summarized using mean, standard deviation, minimum, median, and maximum. Categorical variables will be summarized using frequency counts and percentages.

Unless otherwise specified, the calculation of proportions will be based on the sample size of the population of interest. Counts of missing observations will be included in the denominator and presented as a separate category if not otherwise specified in the SAP.

Statistical analyses will be performed using SAS® version 9.4 or higher.

#### 9.4.1.1. Statistical Testing Strategy

A graphical multiple testing strategy[61] will be used to strongly control the family-wise error rate of the study at 0.025 (1-sided) level. Initially the type I error rate will be equally split between the two null hypotheses defined by the primary objectives, ie, an alpha of 0.0125 (1-sided) will be assigned to test Arm A versus Control PFS, and an alpha of 0.0125 (1-sided) to test Arm B versus Control PFS.

An interim non-binding futility analysis will be conducted on both primary endpoint comparisons once there is at least 40% information for the Arm A versus Control PFS comparison and at least 50% information for the Arm B versus Control PFS comparison. Details of the PFS interim futility analysis are described in Section 9.5.1.

If neither Arm A nor Arm B are stopped for futility, the testing of PFS will be performed in parallel between Arm A versus the Control Arm, and between Arm B versus the Control Arm at the time of the PFS primary analysis.

If a hypothesis test of the primary endpoint is found to be statistically significant at 0.0125 (1 -sided) alpha, the corresponding OS comparison will be tested in an interim analysis

090177e194745c57\Approved\Approved On: 22-Jul-2020 00:48 (GMT)

conducted at the time of the PFS primary analysis. Details of the OS interim efficacy analysis are described in Section 9.5.2. The subsequent steps of the graphical multiple testing strategy are outlined in Figure 2.

If only one of the hypothesis tests of the key secondary endpoint exceeds the superiority boundary at the interim analysis, the final analysis of the remaining OS comparison will be conducted when the number of required events occur for that comparison.

If neither regimen in the SLI is considered tolerable and/or suitable for evaluation in the randomization portion of the trial, the study will proceed as a 2-arm study of Arm A versus the Control Arm. In this instance, all alpha (ie, 1-sided alpha=0.025) will be assigned to the PFS comparison of Arm A versus the Control Arm. If PFS is found to be statistically significant, OS of Arm A versus the Control Arm will then be formally tested at the same alpha level.

The overall significance level is 0.025 (1-sided) and all statistical testing will be performed as 1-sided. No formal statistical testing will be performed on the secondary and exploratory endpoints (with the exception of the OS described above), sensitivity analyses and subgroup analyses. These results will be reported with descriptive statistics only.

Confidence intervals will be 2-sided with a confidence level of 95%, if not otherwise specified.

090177e194745c57\Approved\Approved On: 22-Jul-2020 00:48 (GMT)

**Figure 2: Graphical Gatekeeping Multiple Testing Strategy**

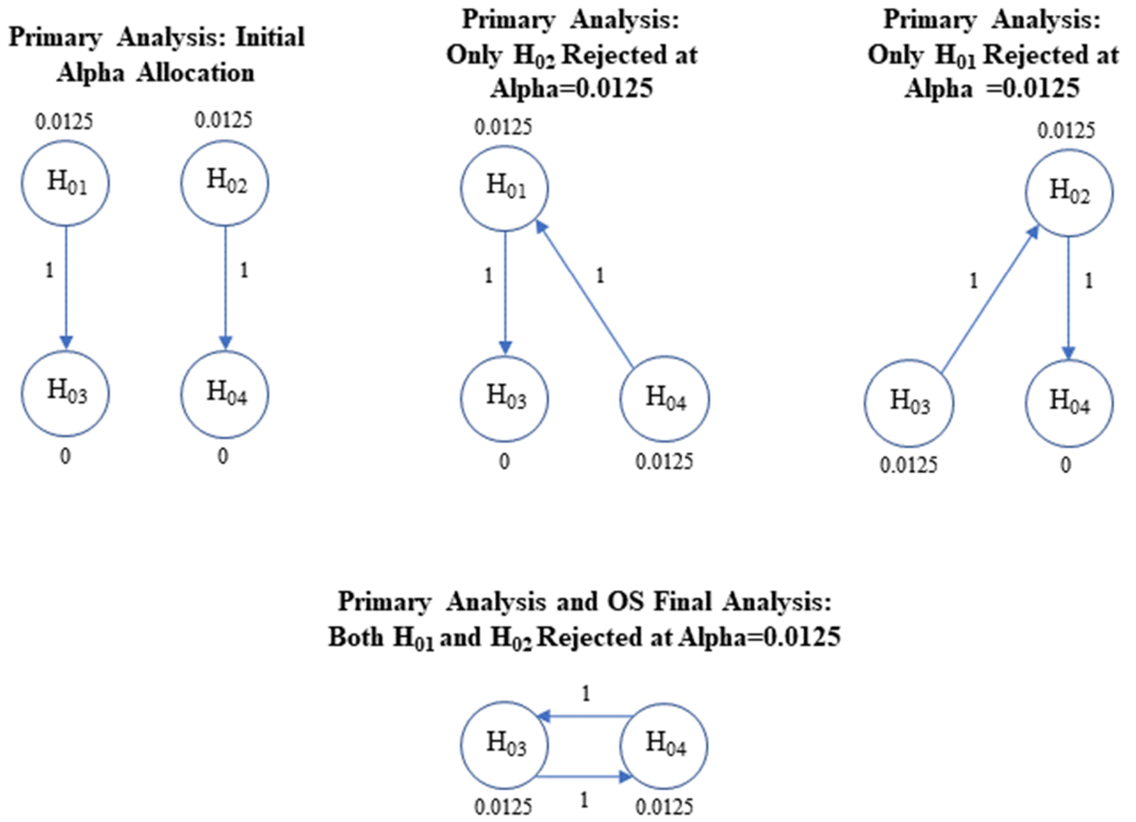


$H_{01}$: $HR_{PFS\ (A\ versus\ Control)} \geq 1$; $H_{02}$: $HR_{PFS\ (B\ versus\ Control)} \geq 1$; $H_{03}$: $HR_{OS\ (A\ versus\ Control)} \geq 1$; $H_{04}$: $HR_{OS\ (B\ versus\ Control)} \geq 1$

Values above and below the hypotheses (circles) represent the amount of alpha allocated to the given hypothesis at the start of the step. Numbers next to the arrows represent the proportion of the alpha that is propagated from the parent hypothesis to the descendant hypothesis when the parent null hypothesis is rejected.

If an OS null hypothesis (ie, $H_{03}$ or $H_{04}$) is rejected at the OS Final Analysis, no alpha can be propagated back to a PFS hypothesis as PFS can only be tested at the Primary Analysis.

#### 9.4.1.2. Pooling of Centers

In order to provide overall estimates of treatment effects, data will be pooled across trial centers. The "center" factor will not be considered in statistical models or for subgroup analyses due to the high number of participating centers in contrast to the anticipated small number of participants randomized at each center.

#### 9.4.1.3. Stratification Factors

The stratification factors used for stratified statistical analyses are those used at randomization via the IRT: ECOG performance status (0 versus 1) and region (US/Canada versus Europe versus Rest of World).

090177e194745c57\Approved\Approved On: 22-Jul-2020 00:48 (GMT)

#### 9.4.1.4. Definition of Baseline

For all endpoints assessments in the SLI and all safety assessments in the Phase 3 portion of the study, baseline is defined as the last completed assessment prior to date of first dose in the SLI and Phase 3, respectively. If an assessment that is planned to be performed prior to the first dose of study intervention in the protocol is performed on the same day as the first dose of study intervention and the time is unknown, it will be assumed that it was performed prior to study intervention administration and will be considered as baseline assessment.

For efficacy assessments in the Phase 3 portion of the study, baseline is defined as the last assessment prior to randomization. If the assessment that is planned to be performed before randomization per the protocol is performed on the same day as the date of randomization and assessment time point is missing, it will be assumed that it was performed prior to randomization and will be considered as baseline assessment. If such a value is missing, the last measurement prior to the first dose of study intervention will be used as the baseline measurement for the efficacy analysis except for analyses of tumor assessments data where the baseline assessment would be considered missing.

Unscheduled assessments will be used in the determination of baseline. Data reported at the End of Treatment visit are not eligible for baseline selection.

#### 9.4.1.5. Definition of On-treatment Period

The on-treatment period is defined as the time from the first dose of study treatment through minimum of (last dose of study treatment + 28 days, start day of subsequent anticancer therapy - 1 day) unless otherwise described in the SAP.

### 9.4.2. Primary Endpoints

**Safety Lead-in**

The primary endpoint in the SLI of the study is incidence of DLTs. The number and proportion of participants experiencing DLTs during the DLT-evaluation period will be summarized and listed by treatment cohort. Analyses of DLT will be performed on DLT-Evaluable Set.

**Phase 3**

The primary objectives are to compare the efficacy, as measured by the primary endpoint of PFS by BICR, of Arm A versus the Control Arm and of Arm B versus the Control Arm. PFS is defined as the time from the date of randomization to the earliest documented disease progression per RECIST v1.1, or death due to any cause. PFS will be calculated in months as follows:

$$\text{PFS (months)} = (\text{date of event or censoring} - \text{randomization date} + 1) / 30.4375$$

Participants without an event or with an event more than 12 weeks (for the first 18 months after randomization) or 16 weeks (after the first 18 months of randomization) after the last adequate tumor assessment will be censored on the date of the last adequate tumor



090177e194745c57\Approved\Approved On: 22-Jul-2020 00:48 (GMT)

assessment that documented no progression. In addition, if a new anti-cancer therapy is started or a metastasectomy procedure is performed prior to an event, the participant will be censored on the date of the last adequate tumor assessment that documented no progression prior to the start of the new anti-cancer therapy or undergoing metastasectomy procedures.

An adequate post-baseline assessment is defined as an assessment where a response of CR, PR, SD, non-CR/non-PD, or PD can be determined. Time points where the response is NE or no assessment was performed will not be used for determining the censoring date.

Participants with no baseline tumor assessment (including participants with an inadequate baseline assessment) or with no adequate post-baseline tumor assessments within 12 weeks after the date of randomization will be censored on the day of randomization, unless the participant dies within 12 weeks of randomization, in which case, death will be an event on date of death.

The censoring and event date options to be considered for the PFS primary analysis are presented in Table 27.

The primary analyses will be performed to compare PFS time between Arm A versus the Control Arm and between Arm B versus the Control Arm using a 1-sided stratified log-rank test at the significance level based on the graphical multiple testing strategy as outlined in Section 9.4.1.1.

The treatment effect will be estimated using a Cox's proportional hazards model stratified by randomization strata to calculate the hazard ratio for PFS of Arm A versus the Control Arm, and of Arm B versus the Control Arm, along with the corresponding 2-sided CI. The confidence level will match the alpha tested (eg, 97.5% CI when PFS is tested at 1-sided 0.0125). In addition, the unadjusted 95% CIs for the hazard ratios will also be reported.

PFS time will be estimated using the Kaplan-Meier method and displayed graphically by treatment arm. The median PFS and associated 95% CIs will be presented by treatment arm. PFS rates at different time points will be estimated with corresponding 2-sided 95% CIs.

An interim non-binding futility analysis will be performed on PFS between Arm A and the Control Arm, and between Arm B and the Control Arm. Details are described in Section 9.5.1.

Sensitivity Analyses

The following sensitivity analyses will be performed to further assess the comparisons described by the primary objectives. Additional sensitivity analyses may be specified in the SAP.

- The PFS analysis will be repeated based on the Centrally Assessed *BRAF* V600E Positive analysis set.
- Treatment policy estimand strategy will be used for intercurrent event of starting new anti-cancer therapy or undergoing metastasectomy procedures. PDs or deaths that



090177e194745c57\Approved\Approved On: 22-Jul-2020 00:48 (GMT)

occur after the start of new anticancer treatment or undergoing metastasectomy procedures will be considered as events. Detailed censoring rules are specified in Table 28.

PFS of Arm A versus Arm B will also be analyzed using the same analysis method described above. However, as this comparison is not part of the graphical multiple testing strategy as outlined in Section 9.4.1.1, no formal testing will be performed to compare PFS of these two treatment arms.

### 9.4.3. Secondary Endpoints

#### 9.4.3.1. OS

OS is defined as the time from the date of first dose (SLI) or the date of randomization (Phase 3) to death due to any cause. If a participant is not known to have died at the time of the cutoff for analysis, then OS will be censored at the date of last contact. OS will be calculated in months as follows:

OS (months) = [date of death or censoring – date of first dose (SLI) or randomization date (Phase 3) +1] / 30.4375

For SLI, OS time will be estimated using the Kaplan-Meier method for Cohort 1 (EC + FOLFIRI) and Cohort 2 (EC +mFOLFOX6), separately.

As the key secondary endpoint in Phase 3 portion of the study, OS will be compared between Arm A and the Control Arm, and between Arm B and the Control Arm using a 1-sided stratified log-rank test at the significance level based on the graphical multiple testing strategy as outlined in Section 9.4.1.1.

The treatment effect will be estimated using a Cox's proportional hazards model stratified by randomization strata to calculate the hazard ratio for OS of Arm A versus the Control Arm, and of Arm B versus the Control Arm. In order to account for the group sequential design on this endpoint, the repeated CI method[62] will be used to construct the 2-sided repeated CI for the hazard ratio at the interim and the final analyses of OS.

In addition, the unadjusted 95% CIs for the hazard ratios will also be reported at the interim and the final analyses for OS.

OS time will be estimated using the same Kaplan-Meier method as described for PFS.

OS of Arm A versus Arm B will also be analyzed using the same analysis method described above. However, as this comparison is not part of the graphical multiple testing strategy as outlined in Section 9.4.1.1, no formal testing will be performed to compare OS of these two treatment arms.

090177e194745c57\Approved\Approved On: 22-Jul-2020 00:48 (GMT)

Sensitivity Analyses

The following sensitivity analyses will be performed to further assess the comparisons described by the key secondary objectives. Additional sensitivity analyses may be specified in the SAP.

- The OS analysis will be repeated based on the Centrally Assessed *BRAF* V600E Positive analysis set.

#### 9.4.3.2. Objective Response Rate

ORR is defined as the proportion of participants who have achieved a BOR of CR or PR per RECIST v1.1. The BOR is the best response obtained among all tumor assessment visits after the date of first dose (SLI) or the date of randomization (Phase 3) until documented disease progression, death, or start of subsequent anticancer therapy. Clinical deterioration or clinical progression noted on the completion eCRF will not be considered as documented disease progression.

Two sets of BOR will be summarized, one for confirmed and one for confirmed + unconfirmed responses. For confirmed BOR, confirmation of the response will be performed per RECIST v1.1, preferably at the regularly scheduled assessment interval, but no sooner than 4 weeks after the initial documentation of CR or PR. Confirmation of PR or CR can be confirmed at an assessment later than the next assessment after the initial documentation of PR or CR, respectively.

A BOR of SD requires that a time point overall response of SD has been determined at a time point at least 6 weeks after the date of first dose (SLI) or the date of randomization (Phase 3).

A BOR of PD requires that the documented progression should be within 12 weeks after the date of first dose (SLI) or the date of randomization (Phase 3) and not qualifying as CR, PR or SD.

The ORR will be calculated along with corresponding 2-sided exact Clopper-Pearson 95% CI.

#### 9.4.3.3. Duration of Response

DOR is defined as the time from the date of first radiographic evidence of response (CR or PR) to the earliest documented disease progression per RECIST v1.1, or death due to any cause. DOR will be calculated for participants with a confirmed response (CR or PR).

DOR will be calculated in months as follows:

$$\text{DOR (months)} = (\text{date of event or censoring} - \text{date of first CR or PR} + 1) / 30.4375$$

The same censoring rules specified for PFS in Section 9.4.2 apply to DOR. Kaplan-Meier method will be used to estimate DOR time. The median DOR and its 95% CIs will be presented by treatment arm. DOR rates at different time points will be estimated with corresponding 2-sided 95% CIs.



090177e194745c57\Approved\Approved On: 22-Jul-2020 00:48 (GMT)

090177e194745c57\Approved\Approved On: 22-Jul-2020 00:48 (GMT)

#### 9.4.3.4. Time to Response

TTR is defined as the time from the date of first dose (SLI) or the date of randomization (Phase 3) to first radiographic evidence of response (CR or PR) per RECIST v1.1. TTR will be calculated for participants with a confirmed response (CR or PR).

TTR will be calculated in weeks as follows:

TTR (in weeks) = (data of first CR or PR – date of first dose (SLI) or date of randomization (Phase 3) + 1) / 7

TTR will be summarized using simple descriptive statistics (mean, standard deviation, median, minimum, maximum, first quartile, third quartile).

#### 9.4.3.5. PFS (Safety Lead-in)

PFS in the SLI is defined as the time from the date of first dose to the earliest documented disease progression per RECIST v1.1, or death due to any cause. PFS will be calculated in months as follows:

PFS (months) = (date of event or censoring – date of first dose in the SLI +1) / 30.4375

PFS time in the SLI will be estimated using the Kaplan-Meier method for Cohort 1 (EC + FOLFIRI) and Cohort 2 (EC + mFOLFOX6), separately. The same censoring rules specified for PFS in Section 9.4.2 will be used for PFS in the SLI with the date of first dose date as the starting date.

#### 9.4.3.6. PFS2

PFS2 is defined as the time from the date of randomization to second objective disease progression per RECIST v1.1, or death from any cause, whichever occurs first. Second objective disease progression is PD after the start of subsequent anticancer therapy. PFS2 will be calculated in months as follows:

PFS2 (months) = [minimum of (date of death, date of second objective PD) or censoring– randomization date +1] / 30.4375

Participants alive and for whom a second objective PD has not been observed will be censored at the last time known to be alive and without second objective PD. In addition, participants who die after missing 2 or more tumor assessments while on study treatment will remain censored for PFS2.

PFS2 time will be estimated using the same Kaplan-Meier method as described for PFS.

#### 9.4.3.7. Patient Reported Outcomes

Detailed methodology for summary and statistical analyses of the data on PROs will be further detailed in the SAP. All PRO analyses will be performed on PRO analysis set as defined in Section 9.3.

The EORTC QLQ-C30, EQ-5D-5L, PGIC and PGIS will be scored according to their respective user guides/scoring manuals.

The number and percentage of participants who complete each questionnaire will be summarized at each time point by treatment arm, as will the reasons for non-completion of these measures.

Patient-reported health-related QoL, disease and treatment-related symptoms of CRC, and health status will be assessed. Summary statistics (mean, standard deviation, median, range, and 95% CI) of absolute scores will be reported for the items and subscales of the EORTC QLQ-C30, and the EQ-5D-5L VAS scale. The mean change of absolute scores from baseline and corresponding 95% CI will also be calculated for the scales and for the PGIS. Line charts depicting the means and mean changes of items and subscales over time will be created by treatment arm.

The number and proportion of participants who improved, worsened, or remained stable for the symptom and functional domains, global QoL and single items of the EORTC QLQ-C30 and PGIC and PGIS will be summarized by treatment arm.

Improvement, worsening, and stable categories will be determined over each cycle and summarized as an average by participant in the symptom scales, functioning scales, and in the global QoL scale. The definitions of improvement/worsening are generally based on a variation of 10 points or 10% in the score. In Study ARRAY-818-302 (BEACON CRC), the definition of improvement/worsening was based on a 10% variation in scores. We will adopt the criterion of 10% variation in this study, and will perform additional analyses based on the criterion of 10-point variation.

Time to definitive 10% deterioration in the global QoL scale will be described in tabular and graphical format by treatment arm using Kaplan-Meier methods, including estimated median (in months) with 95% CI and $25^{th}$ / $75^{th}$ percentiles. Kaplan-Meier estimated probabilities with corresponding 95% CI will be calculated at pre-specified timepoints.

For the EQ-5D-5L health state profiles, the proportion of participants reporting “no”, “slight”, “moderate”, “severe”, or “extreme” problems will be reported at each time point.

Statistical comparisons of the treatment arms will be based on longitudinal repeated measures mixed effects models using baseline as a covariate.

Meaningful change will be evaluated using scores on the PGIC scale as an anchor with supportive analyses using the PGIS scale. Clinically meaningful change will be calculated using data from patients who rate their status as moderately improved on the PGIC questions. Changes in scores from baseline to EOT visit will be tabulated for the EQ-5D-5L, EQ-5D-5L VAS, and EORTC-QLQ-C30 GHS for each level of the PGIC. The tabulation will include baseline score, EOT visit score, difference, and SD max and min for the difference score.

090177e194745c57\Approved\Approved On: 22-Jul-2020 00:48 (GMT)

#### 9.4.3.8. Pharmacokinetic Analyses

Plasma concentrations of encorafenib and a metabolite LHY746, irinotecan and an active metabolite SN-38, and total and free plasma concentrations of platinum of oxaliplatin will be determined using validated assays. Descriptive statistics of concentrations of encorafenib and LHY746, irinotecan and SN-38, and oxaliplatin will be reported and summarized graphically. Pharmacokinetic parameters from the SLI arms will be generated by noncompartmental and/or compartmental approaches as appropriate, summarized and reported. Potential drug interactions between encorafenib and irinotecan or oxaliplatin will be assessed in the respective SLI arms by intra-participant comparisons or comparisons to historical results. Details of analyses will be included in statistical analysis plans and results will be reported.

#### 9.4.3.9. Exposure-Response of Encorafenib

Descriptive statistics of  trough plasma concentrations of encorafenib and a metabolite LHY746 will be reported for in the Phase 3 portion of this study. Phase 3 PK parameters will be estimated for encorafenib and LHY746 as appropriate using a population model-based approach. Relationships between PK of encorafenib and biomarkers, clinical response and/or safety will be conducted using post hoc exposure estimates (eg, $C_{max}$, AUC or $C_{min}$) from population PK modeling, if possible and appropriate. Measures of efficacy may include but are not limited to OS, PFS and ORR. Selection of safety measures will be based on frequency of observations or by selection of safety measures of interest. The impact of mFOLFOX6 or FOLFIRI on encorafenib exposure or responses will be evaluated by comparing Arm B to Arm A, or to historical data. Analyses will be described in a separate stand-alone modeling plan and a specific report will be produced.

#### 9.4.3.10. Biomarker Analyses

Biomarker data will be assessed separately for whole blood, serum, plasma and tumor tissue.

For continuous measurement biomarker results, summary statistics (eg, the mean, standard deviation, median, percent of coefficient of variation, and minimum/maximum levels) will be determined at baseline and on-treatment time points, as appropriate. Appropriate change from baseline measurements will be provided.

For discrete measurement biomarkers (eg, tumor marker status), frequencies and percentages of categorical biomarker measures will be determined at baseline and on-treatment time points. Shift tables may also be provided.

Data from biomarker assays may be analyzed using graphical methods and descriptive statistics such as Fisher's exact test, Wilcoxon rank-sum test, Kaplan-Meier estimates, and linear regression as appropriate. The statistical approaches will explore the correlations of biomarker results with pharmacokinetic parameters and/or measures of anti-tumor efficacy, such as tumor response and progression free survival.

### 9.4.4. Tertiary/Exploratory Endpoints

The details of tertiary/exploratory endpoints analyses will be described in the SAP or in a separate exploratory analysis plan.



090177e194745c57\Approved\Approved On: 22-Jul-2020 00:48 (GMT)

Results from exploratory analyses will be reported in the CSR where possible. However, given the exploratory nature of the objective and endpoints, the analyses may not be completed at the time of the CSR. Results from exploratory analyses that are not included in the CSR will be shared with the scientific community through publication at scientific conferences and/or in peer-reviewed scientific journals.

### 9.4.5. Safety Analyses

All safety analyses will be performed on the safety analysis set.

Safety data will be presented in tabular and/or graphical format and summarized descriptively, where appropriate.

#### 9.4.5.1. Adverse Events

Adverse events will be coded using current MedDRA. Severity of AEs will be graded using the NCI CTCAE v 4.03 toxicity grading scale. Incidence tables will be presented for all AEs by maximum severity, SAEs, AEs assessed as related to study intervention and AEs resulting in discontinuation of study intervention. Details will be described in the SAP.

#### 9.4.5.2. Clinical Laboratory Results

Clinical laboratory data will be classified by grade according to NCI CTCAE version 4.03 and will be analyzed using summary statistics. The worst on-treatment grades during the treatment period will be summarized. Shifts in toxicity grading from baseline to highest grade during the on-treatment period will be displayed. Results for laboratory tests that are not part of NCI CTCAE will be presented as below, within, or above normal limits. Only participants with post-baseline laboratory values will be included in these analyses. Further details of analyses for all the laboratory parameters will be provided in the SAP.

#### 9.4.5.3. Electrocardiogram Analyses

ECG parameters QT interval, heart rate, QTcF interval, PR interval, and QRS complex will be summarized by treatment and time point. Further details of analyses for ECG parameters will be provided in the SAP.

### 9.4.6. Other Analyses

Descriptive statistics will be used to summarize all participant characteristics, treatment administration/compliance, vital signs, body weight and ECOG performance status. Data will also be displayed graphically, where appropriate.

Other exploratory PRO analyses may be performed subsequently as needed.

Pharmacogenomic or biomarker data from Banked Biospecimens may be collected during or after the trial and retained for future analyses; the results of such analyses are not planned to be included in the CSR.

090177e194745c57\Approved\Approved On: 22-Jul-2020 00:48 (GMT)

#### 9.4.6.1. Subgroup Analyses

Subgroup analyses will be performed to further assess the comparisons described by the primary and key secondary objectives. The HR of PFS and OS and the corresponding 95% CI will be computed and displayed per subgroup level using a forest plot. Further details including the precise subgroups used will be detailed in the SAP.

## 9.5. Interim Analyses

### 9.5.1. Interim Futility Analysis for PFS

An interim non-binding futility analysis will be conducted on both primary endpoint comparisons once there are at least 157 PFS events in Arm A + the Control Arm (ie, approximately 40% information) and at least 157 PFS events in Arm B + the Control Arm (ie, approximately 50% information). This is anticipated to occur approximately 20 months after the first participant is randomized. The futility boundaries (non-binding) will be determined using a Rho family beta-spending function with parameter = 3. For PFS of Arm A versus the Control Arm, with 157 PFS events, the futility boundary will be crossed if the HR is >1.04. For PFS of Arm B versus the Control Arm, with 157 PFS events, the futility boundary will be crossed if the HR is >0.96. The exact futility boundaries will be updated prior to the time of analysis based on the actual number of events and the information fraction.

### 9.5.2. Interim Efficacy Analysis for OS

If one or both of the hypothesis tests of the PFS primary endpoint is statistically significant, an interim analysis for superiority will be conducted at that time on the corresponding OS comparison. It is anticipated that this will occur with approximately 70% information in the Arm A versus the Control Arm OS comparison and with approximately 80% information in the Arm B versus the Control Arm OS comparison. A Lan-DeMets alpha-spending function[63] that approximates O'Brien-Fleming stopping boundaries will be used to control the overall type I error rate at 0.0125 level (1-sided).

Table 31 summarizes the operating characteristics and efficacy boundaries for the OS analysis. The exact boundaries may be updated prior or the time of the analysis based on the actual number of events and the information fraction.

090177e194745c57\Approved\Approved On: 22-Jul-2020 00:48 (GMT)

**Table 31. OS – Summary of Planned Efficacy Boundaries**

| Formal Testing | Analysis | Information Fraction[a] | Cumulative OS Events | Cumulative Type I Error (1-sided)[b] | Efficacy Boundaries | | Cum. Prob of Reject $H_0$ for Efficacy if $H_1$ is True |
|---|---|---|---|---|---|---|---|
| | | | | | P-scale (1-sided) | HR Scale | |
| Arm A vs Control | Interim Efficacy | 70% | 282 | 0.0028 | 0.0028 | 0.72 | 0.496 |
| | Final | 100% | 403 | 0.0125 | 0.0116 | 0.80 | 0.850 |
| Arm B vs Control | Interim Efficacy | 80% | 282 | 0.0052 | 0.0052 | 0.73 | 0.654 |
| | Final | 100% | 344 | 0.0125 | 0.0109 | 0.78 | 0.850 |

The efficacy boundaries are determined using Lan-DeMets alpha spending function that approximates O'Brien-Fleming stopping boundaries.

a The exact efficacy boundaries will be calculated based on the actual number of events observed.

b The overall type I error rate was assumed to be 0.0125 (1-sided) for each endpoint. The actual alpha value used during testing will be based on the graphical multiple testing strategy outlined in Section 9.4.1.1.

Before any interim analysis is conducted, the details of the objectives, decision criteria, dissemination plan and method of maintaining the study blind as per Pfizer's SOPs will be documented and approved in an E-DMC charter. In addition, the analysis details will be documented and approved in the study SAP.

## 9.6. Data Monitoring Committee or Other Independent Oversight Committee

There will be an E-DMC to review cumulative safety data during the study conduct as well as review the interim futility analysis on the PFS data. The E-DMC is independent of the study (ie, does not include any investigators in the study) and includes only members external to study Sponsors. The E-DMC will evaluate the safety data and PK data as it is available at pre-specified intervals and at additional points during the conduct of the SLI. During the Phase 3 portion of the study, the E-DMC will assess safety/tolerability after the first 30 participants have been randomized and treated for at least 1 cycle and then approximately every 6 months.

The E-DMC will be responsible for ongoing monitoring on the efficacy, safety and PK data of participants in the study according to the charter. The recommendation made by the E-DMC to alter the conduct of the study will be forwarded to the appropriate Pfizer personnel for final decision. Pfizer will forward such decisions, which may include summaries of aggregate analyses of safety data, to regulatory authorities, as appropriate. The E-DMC charter describes the role of the E-DMC in more detail.

090177e194745c57\Approved\Approved On: 22-Jul-2020 00:48 (GMT)

# 10. SUPPORTING DOCUMENTATION AND OPERATIONAL CONSIDERATIONS

## 10.1. Appendix 1: Regulatory, Ethical, and Study Oversight Considerations

### 10.1.1. Regulatory and Ethical Considerations

This study will be conducted in accordance with the protocol and with the following:

- Consensus ethical principles derived from international guidelines including the Declaration of Helsinki and CIOMS International Ethical Guidelines;
- Applicable ICH GCP guidelines;
- Applicable laws and regulations, including applicable privacy laws.

The protocol, protocol amendments, ICD, SRSD(s), and other relevant documents (eg, advertisements) must be reviewed and approved by the sponsor and submitted to an IRB/ethics committee by the investigator and reviewed and approved by the IRB/ethics committee before the study is initiated.

Any amendments to the protocol will require IRB/ethics committee approval before implementation of changes made to the study design, except for changes necessary to eliminate an immediate hazard to study participants.

The investigator will be responsible for the following:

- Providing written summaries of the status of the study to the IRB/ethics committee annually or more frequently in accordance with the requirements, policies, and procedures established by the IRB/ethics committee;
- Notifying the IRB/ethics committee of SAEs or other significant safety findings as required by IRB/ethics committee procedures;
- Providing oversight of the conduct of the study at the site and adherence to requirements of 21 CFR, ICH guidelines, the IRB/ethics committee, European regulation 536/2014 for clinical studies (if applicable), and all other applicable local regulations.

#### 10.1.1.1. Reporting of Safety Issues and Serious Breaches of the Protocol or ICH GCP

In the event of any prohibition or restriction imposed (ie, clinical hold) by an applicable regulatory authority in any area of the world, or if the investigator is aware of any new information that might influence the evaluation of the benefits and risks of the study intervention, Pfizer should be informed immediately.

In addition, the investigator will inform Pfizer immediately of any urgent safety measures taken by the investigator to protect the study participants against any immediate hazard, and of any serious breaches of this protocol or of ICH GCP that the investigator becomes aware of.

090177e194745c57\Approved\Approved On: 22-Jul-2020 00:48 (GMT)

### 10.1.2. Financial Disclosure

Investigators and sub-investigators will provide the sponsor with sufficient, accurate financial information as requested to allow the sponsor to submit complete and accurate financial certification or disclosure statements to the appropriate regulatory authorities. Investigators are responsible for providing information on financial interests during the course of the study and for 1 year after completion of the study.

### 10.1.3. Informed Consent/Assent Process

The investigator or his/her representative will explain the nature of the study to the participant or his/her legally authorized representative and answer all questions regarding the study. The participant or his/her legally authorized representative should be given sufficient time and opportunity to ask questions and to decide whether or not to participate in the trial.

Participants must be informed that their participation is voluntary. Participants and/or his/her legally authorized representative, defined as parent(s) or legal guardian, will be required to sign a statement of informed consent/assent that meets the requirements of 21 CFR 50, local regulations, ICH guidelines, HIPAA requirements, where applicable, and the IRB/ethics committee or study center. When appropriate, adolescent participants will be included in all discussions regarding participation in the trial and their assent will be obtained and documented.

The investigator must ensure that each study participant or his/her legally authorized representative is fully informed about the nature and objectives of the study, the sharing of data related to the study, and possible risks associated with participation, including the risks associated with the processing of the participant's personal data.

The participant must be informed that his/her personal study-related data will be used by the sponsor in accordance with local data protection law. The level of disclosure must also be explained to the participant.

The participant must be informed that his/her medical records may be examined by Clinical Quality Assurance auditors or other authorized personnel appointed by the sponsor, by appropriate IRB/ethics committee members, and by inspectors from regulatory authorities.

The investigator further must ensure that each study participant or his/her legally authorized representative is fully informed about his or her right to access and correct his or her personal data and to withdraw consent/assent for the processing of his or her personal data.

The medical record must include a statement that written informed consent was obtained before the participant was enrolled in the study and the date the written consent was obtained. For adolescent participants, the medical record must include a statement that legally authorized representative (parent/guardian) consent and child/adolescent assent (if deemed appropriate by local ethics review) was obtained before the participant was enrolled in the study and the date the written consent/assent was obtained. The medical record should also describe how the clinical investigator determined that the person signing the ICD was the



090177e194745c57\Approved\Approved On: 22-Jul-2020 00:48 (GMT)

participant's legally authorized representative (parent/guardian).The authorized person obtaining the informed consent/assent must also sign the ICD.

Participants and/or his/her legally authorized representative must be reconsented/assented to the most current version of the ICD(s) during their participation in the study.

Minor participants must be reconsented if they reach the age of majority during the course of the study, in order to continue participating.

A copy of the ICD(s) must be provided to the participant or the participant's legally authorized representative.

A participant who is rescreened is not required to sign another ICD if the rescreening occurs within 28 days from the previous ICD signature date.

Unless prohibited by local requirements or IRB/ethics committee decision, the ICD will contain a separate section that addresses the use of samples for optional additional research. The optional additional research does not require the collection of any further samples. The investigator or authorized designee will explain to each participant the objectives of the additional research. Participants will be told that they are free to refuse to participate and may withdraw their consent/assent at any time and for any reason during the storage period. A separate signature will be required to document a participant's agreement to allow specimens to be used for additional research. Participants who decline to participate in this optional additional research will not provide this separate signature.

### 10.1.4. Data Protection

All parties will comply with all applicable laws, including laws regarding the implementation of organizational and technical measures to ensure protection of participant data.

Participants' personal data will be stored at the study site in encrypted electronic and/or paper form and will be password protected or secured in a locked room to ensure that only authorized study staff have access. The study site will implement appropriate technical and organizational measures to ensure that the personal data can be recovered in the event of disaster. In the event of a potential personal data breach, the study site will be responsible for determining whether a personal data breach has in fact occurred and, if so, providing breach notifications as required by law.

To protect the rights and freedoms of participants with regard to the processing of personal data, participants will be assigned a single, participant-specific numerical code. Any participant records or data sets that are transferred to the sponsor will contain the numerical code; participant names will not be transferred. All other identifiable data transferred to the sponsor will be identified by this single, participant-specific code. The study site will maintain a confidential list of participants who participated in the study, linking each participant's numerical code to his or her actual identity and medical record identification. In case of data transfer, the sponsor will protect the confidentiality of participants' personal data consistent with the clinical study agreement and applicable privacy laws.



090177e194745c57\Approved\Approved On: 22-Jul-2020 00:48 (GMT)

### 10.1.5. Dissemination of Clinical Study Data

Pfizer fulfills its commitment to publicly disclose clinical study results through posting the results of studies on www.clinicaltrials.gov (ClinicalTrials.gov), the EudraCT, and/or www.pfizer.com, and other public registries in accordance with applicable local laws/regulations. In addition, Pfizer reports study results outside of the requirements of local laws/regulations pursuant to its SOPs.

In all cases, study results are reported by Pfizer in an objective, accurate, balanced, and complete manner and are reported regardless of the outcome of the study or the country in which the study was conducted.

<u>www.clinicaltrials.gov</u>

Pfizer posts clinical trial results on www.clinicaltrials.gov for Pfizer-sponsored interventional studies (conducted in participants) that evaluate the safety and/or efficacy of a product, regardless of the geographical location in which the study is conducted. These results are submitted for posting in accordance with the format and timelines set forth by US law.

<u>EudraCT</u>

Pfizer posts clinical trial results on EudraCT for Pfizer-sponsored interventional studies in accordance with the format and timelines set forth by EU requirements.

<u>www.pfizer.com</u>

Pfizer posts public disclosure synopses (CSR synopses in which any data that could be used to identify individual participants have been removed) on www.pfizer.com for Pfizer-sponsored interventional studies at the same time the corresponding study results are posted to www.clinicaltrials.gov.

<u>Documents within marketing authorization packages/submissions</u>

Pfizer complies with the European Union Policy 0070, the proactive publication of clinical data to the EMA website. Clinical data, under Phase 1 of this policy, includes clinical overviews, clinical summaries, CSRs, and appendices containing the protocol and protocol amendments, sample CRFs, and statistical methods. Clinical data, under Phase 2 of this policy, includes the publishing of individual participant data. Policy 0070 applies to new marketing authorization applications submitted via the centralized procedure since 01 January 2015 and applications for line extensions and for new indications submitted via the centralized procedure since 01 July 2015.

<u>Data Sharing</u>

Pfizer provides researchers secure access to participant-level data or full CSRs for the purposes of "bona-fide scientific research" that contributes to the scientific understanding of the disease, target, or compound class. Pfizer will make available data from these trials 24 months after study completion. Participant-level data will be anonymized in accordance



090177e194745c57\Approved\Approved On: 22-Jul-2020 00:48 (GMT)

with applicable privacy laws and regulations. CSRs will have personally identifiable information redacted.

Data requests are considered from qualified researchers with the appropriate competencies to perform the proposed analyses. Research teams must include a biostatistician. Data will not be provided to applicants with significant conflicts of interest, including individuals requesting access for commercial/competitive or legal purposes.

### 10.1.6. Data Quality Assurance

All participant data relating to the study will be recorded on printed or electronic CRF unless transmitted to the sponsor or designee electronically (eg, laboratory data). The investigator is responsible for verifying that data entries are accurate and correct by physically or electronically signing the CRF.

The investigator must maintain accurate documentation (source data) that supports the information entered in the CRF.

The investigator must ensure that the CRFs are securely stored at the study site in encrypted electronic and/or paper form and are password protected or secured in a locked room to prevent access by unauthorized third parties.

The investigator must permit study-related monitoring, audits, IRB/ethics committee review, and regulatory agency inspections and provide direct access to source data documents. This verification may also occur after study completion. It is important that the investigator(s) and their relevant personnel are available during the monitoring visits and possible audits or inspections and that sufficient time is devoted to the process.

Monitoring details describing strategy (eg, risk-based initiatives in operations and quality such as risk management and mitigation strategies and analytical risk-based monitoring), methods, responsibilities, and requirements, including handling of noncompliance issues and monitoring techniques (central, remote, or on-site monitoring), are provided in the monitoring plan.

The sponsor or designee is responsible for the data management of this study, including quality checking of the data.

Study monitors will perform ongoing source data verification to confirm that data entered into the CRF by authorized site personnel are accurate, complete, and verifiable from source documents; that the safety and rights of participants are being protected; and that the study is being conducted in accordance with the currently approved protocol and any other study agreements, ICH GCP, and all applicable regulatory requirements.

Records and documents, including signed ICDs, pertaining to the conduct of this study must be retained by the investigator for 15 years after study completion unless local regulations or institutional policies require a longer retention period. No records may be destroyed during the retention period without the written approval of the sponsor. No records may be



090177e194745c57\Approved\Approved On: 22-Jul-2020 00:48 (GMT)

transferred to another location or party without written notification to the sponsor. The investigator must ensure that the records continue to be stored securely for as long as they are maintained.

When participant data are to be deleted, the investigator will ensure that all copies of such data are promptly and irrevocably deleted from all systems.

The investigator(s) will notify the sponsor or its agents immediately of any regulatory inspection notification in relation to the study. Furthermore, the investigator will cooperate with the sponsor or its agents to prepare the investigator site for the inspection and will allow the sponsor or its agent, whenever feasible, to be present during the inspection. The investigator site and investigator will promptly resolve any discrepancies that are identified between the study data and the participant's medical records. The investigator will promptly provide copies of the inspection findings to the sponsor or its agent. Before response submission to the regulatory authorities, the investigator will provide the sponsor or its agents with an opportunity to review and comment on responses to any such findings.

### 10.1.7. Source Documents

Source documents provide evidence for the existence of the participant and substantiate the integrity of the data collected. Source documents are filed at the investigator site.

Data reported on the CRF or entered in the eCRF that are from source documents must be consistent with the source documents or the discrepancies must be explained. The investigator may need to request previous medical records or transfer records, depending on the study. Also, current medical records must be available.

Definition of what constitutes source data can be found in the clinical monitoring plan.

Description of the use of computerized system is documented in the Data Management Plan.

### 10.1.8. Study and Site Start and Closure

The study start date is the date on which the clinical study will be open for recruitment of participants.

The first act of recruitment is the date of the first participant's first visit and will be the study start date.

The sponsor designee reserves the right to close the study site or terminate the study at any time for any reason at the sole discretion of the sponsor. Study sites will be closed upon study completion. A study site is considered closed when all required documents and study supplies have been collected and a study -site closure visit has been performed.

The investigator may initiate study-site closure at any time upon notification to the sponsor or designee/CRO if requested to do so by the responsible IRB/ethics committee or if such termination is required to protect the health of study participants.

090177e194745c57\Approved\Approved On: 22-Jul-2020 00:48 (GMT)

Reasons for the early closure of a study site by the sponsor may include but are not limited to:

- Failure of the investigator to comply with the protocol, the requirements of the IRB/ethics committee or local health authorities, the sponsor's procedures, or GCP guidelines;
- Inadequate recruitment of participants by the investigator;
- Discontinuation of further study intervention development.

If the study is prematurely terminated or suspended, the sponsor shall promptly inform the investigators, the ethics committees/IRBs, the regulatory authorities, and any CRO(s) used in the study of the reason for termination or suspension, as specified by the applicable regulatory requirements. The investigator shall promptly inform the participant and should assure appropriate participant therapy and/or follow-up.

Study termination is also provided for in the clinical study agreement. If there is any conflict between the contract and this protocol, the contract will control as to termination rights.

#### 10.1.9. Publication Policy

The results of this study may be published or presented at scientific meetings by the investigator after publication of the overall study results or 1 year after the end of the study (or study termination), whichever comes first.

The investigator agrees to refer to the primary publication in any subsequent publications such as secondary manuscripts, and submits all manuscripts or abstracts to the sponsor 30 days before submission. This allows the sponsor to protect proprietary information and to provide comments and the investigator will, on request, remove any previously undisclosed confidential information before disclosure, except for any study- or Pfizer intervention-related information necessary for the appropriate scientific presentation or understanding of the study results.

For all publications relating to the study, the investigator will comply with recognized ethical standards concerning publications and authorship, including those established by the International Committee of Medical Journal Editors.

The sponsor will comply with the requirements for publication of the overall study results covering all investigator sites. In accordance with standard editorial and ethical practice, the sponsor will support publication of multicenter studies only in their entirety and not as individual site data. In this case, a coordinating investigator will be designated by mutual agreement.

Authorship of publications for the overall study results will be determined by mutual agreement and in line with International Committee of Medical Journal Editors authorship requirements.

090177e194745c57\Approved\Approved On: 22-Jul-2020 00:48 (GMT)

If publication is addressed in the clinical study agreement, the publication policy set out in this section will not apply.

### 10.1.10. Sponsor's Qualified Medical Personnel

The contact information for the sponsor's appropriately qualified medical personnel for the study is documented in the study contact list located in the Study Manual.

To facilitate access to appropriately qualified medical personnel on study-related medical questions or problems, participants are provided with a contact card at the time of informed consent/assent. The contact card contains, at a minimum, protocol and study intervention identifiers, participant numbers, contact information for the investigator site, and contact details for a contact center in the event that the investigator site staff cannot be reached to provide advice on a medical question or problem originating from another healthcare professional not involved in the participant's participation in the study. The contact number can also be used by investigator staff if they are seeking advice on medical questions or problems; however, it should be used only in the event that the established communication pathways between the investigator site and the study team are not available. It is therefore intended to augment, but not replace, the established communication pathways between the investigator site and the study team for advice on medical questions or problems that may arise during the study. The contact number is not intended for use by the participant directly, and if a participant calls that number, he or she will be directed back to the investigator site.

090177e194745c57\Approved\Approved On: 22-Jul-2020 00:48 (GMT)

090177e194745c57\Approved\Approved On: 22-Jul-2020 00:48 (GMT)

## 10.2. Appendix 2: Clinical Laboratory Tests

The following safety laboratory tests will be performed at times defined in the SoA section of this protocol. Additional laboratory results may be reported on these samples as a result of the method of analysis or the type of analyzer used by the clinical laboratory, or as derived from calculated values. These additional tests would not require additional collection of blood. Unscheduled clinical laboratory measurements may be obtained at any time during the study to assess any perceived safety issues.

**Table 32. Protocol-Required Safety Laboratory Assessments**

| Hematology | Chemistry | Urinalysis | Coagulation | Other |
|---|---|---|---|---|
| • Hemoglobin<br>• Hematocrit<br>• RBC count<br>• Platelet count<br>• WBC count<br>• Total neutrophils (Abs) | • BUN<br>• Creatinine<br>• Glucose<br>• Calcium<br>• Sodium<br>• Magnesium<br>• Potassium<br>• Chloride<br>• Total $CO_2$ (bicarbonate)<br>• AST, ALT<br>• Albumin<br>• Total bilirubin<br>• Direct bilirubin (if total bilirubin values are above normal)<br>• Alkaline phosphatase<br>• Uric acid<br>• Total protein<br>• Lipase | • pH<br>• Glucose (qual)<br>• Protein (qual)<br>• Blood (qual)<br>• Ketones | • PT or INR<br>• PTT or aPTT | • Pregnancy test (β-hCG)[a]<br><br>At screening only:<br>• FSH[b]<br>• Hepatitis B surface antigen (at Screening)[c]<br>• Hepatitis C antibody (at Screening)[d]<br>• HIV, as applicable per local regulations<br><br>Tumor markers:<br>• LDH<br>• CRP<br>• CEA |

a Serum at screening and urine β-hCG during the study for female participants of childbearing potential. Local urine testing will be standard for the protocol unless serum testing is required by local regulation or IRB/ethics committee.

b For confirmation of postmenopausal status only.

c If HBsAg positive, HBV DNA must be measured

d If HCV antibody positive, HCV RNA must be measured

Investigators must document their review of each laboratory safety report.

## 10.3. Appendix 3: Adverse Events: Definitions and Procedures for Recording, Evaluating, Follow-up, and Reporting

### 10.3.1. Definition of AE

| AE Definition |
|---|
| • An AE is any untoward medical occurrence in a patient or clinical study participant, temporally associated with the use of study intervention, whether or not considered related to the study intervention.<br><br>• NOTE: An AE can therefore be any unfavorable and unintended sign (including an abnormal laboratory finding), symptom, or disease (new or exacerbated) temporally associated with the use of study intervention. |

| Events Meeting the AE Definition |
|---|
| • Any abnormal laboratory test results (hematology, clinical chemistry, or urinalysis) or other safety assessments (eg, ECG, radiological scans, vital sign measurements), including those that worsen from baseline, considered clinically significant in the medical and scientific judgment of the investigator  Any abnormal laboratory test results that meet any of the conditions below must be recorded as an AE:<br><br>    • Is associated with accompanying symptoms.<br><br>    • Requires additional diagnostic testing or medical/surgical intervention.<br><br>    • Leads to a change in study dosing (outside of any protocol-specified dose adjustments) or discontinuation from the study, significant additional concomitant drug treatment, or other therapy.<br><br>• Exacerbation of a chronic or intermittent preexisting condition including either an increase in frequency and/or intensity of the condition.<br><br>• New conditions detected or diagnosed after study intervention administration even though it may have been present before the start of the study.<br><br>• Signs, symptoms, or the clinical sequelae of a suspected drug-drug interaction.<br><br>• Signs, symptoms, or the clinical sequelae of a suspected overdose of either study intervention or a concomitant medication.  Overdose per se will not be reported as an AE/SAE unless it is an intentional overdose taken with possible suicidal/self-harming intent.  Such overdoses should be reported regardless of sequelae. |

090177e194745c57\Approved\Approved On: 22-Jul-2020 00:48 (GMT)

| Events NOT Meeting the AE Definition |
|---|
| • Any clinically significant abnormal laboratory findings or other abnormal safety assessments which are associated with the underlying disease, unless judged by the investigator to be more severe than expected for the participant's condition.<br>• The disease/disorder being studied or expected progression, signs, or symptoms of the disease/disorder being studied, unless more severe than expected for the participant's condition.<br>• Medical or surgical procedure (eg, endoscopy, appendectomy): the condition that leads to the procedure is the AE.<br>• Situations in which an untoward medical occurrence did not occur (social and/or convenience admission to a hospital).<br>• Anticipated day-to-day fluctuations of preexisting disease(s) or condition(s) present or detected at the start of the study that do not worsen.<br>• Worsening of signs and symptoms of the malignancy under study should be recorded as AEs in the appropriate section of the CRF. Disease progression assessed by measurement of malignant lesions on radiographs or other methods should not be reported as AEs. |

### 10.3.2. Definition of SAE

If an event is not an AE per definition above, then it cannot be an SAE even if serious conditions are met (eg, hospitalization for signs/symptoms of the disease under study, death due to progression of disease).

090177e194745c57\Approved\Approved On: 22-Jul-2020 00:48 (GMT)

| An SAE is defined as any untoward medical occurrence that, at any dose: |
|---|
| **a. Results in death** |
| **b. Is life-threatening**<br>The term "life-threatening" in the definition of "serious" refers to an event in which the participant was at risk of death at the time of the event. It does not refer to an event that hypothetically might have caused death if it were more severe. |
| **c. Requires inpatient hospitalization or prolongation of existing hospitalization**<br>In general, hospitalization signifies that the participant has been detained (usually involving at least an overnight stay) at the hospital or emergency ward for observation and/or treatment that would not have been appropriate in the physician's office or outpatient setting. Complications that occur during hospitalization are AEs. If a complication prolongs hospitalization or fulfills any other serious criteria, the event is serious. When in doubt as to whether "hospitalization" occurred or was necessary, the AE should be considered serious.<br><br>Hospitalization for elective treatment of a preexisting condition that did not worsen from baseline is not considered an AE. |
| **d. Results in persistent disability/incapacity**<br>• The term disability means a substantial disruption of a person's ability to conduct normal life functions.<br>• This definition is not intended to include experiences of relatively minor medical significance such as uncomplicated headache, nausea, vomiting, diarrhea, influenza, and accidental trauma (eg, sprained ankle) which may interfere with or prevent everyday life functions but do not constitute a substantial disruption. |
| **e. Is a congenital anomaly/birth defect** |
| **f. Other situations:**<br>• Medical or scientific judgment should be exercised in deciding whether SAE reporting is appropriate in other situations such as important medical events that may not be immediately life-threatening or result in death or hospitalization but may jeopardize the participant or may require medical or surgical intervention to prevent one of the other outcomes listed in the above definition. These events should usually be considered serious.<br>• Examples of such events include invasive or malignant cancers, intensive treatment in an emergency room or at home for allergic bronchospasm, blood dyscrasias or |

090177e194745c57\Approved\Approved On: 22-Jul-2020 00:48 (GMT)

convulsions that do not result in hospitalization, or development of drug dependency or drug abuse.

- Progression of the malignancy under study (including signs and symptoms of progression) should not be reported as an SAE unless the outcome is fatal within the active collection period. Hospitalization due to signs and symptoms of disease progression should not be reported as an SAE. If the malignancy has a fatal outcome during the study or within the active collection period, then the event leading to death must be recorded as an AE on the CRF, and as an SAE with CTCAE Grade 5 (see the Assessment of Intensity section).

- Suspected transmission via a Pfizer product of an infectious agent, pathogenic or non-pathogenic, is considered serious. The event may be suspected from clinical symptoms or laboratory findings indicating an infection in a patient exposed to a Pfizer product. The terms "suspected transmission" and "transmission" are considered synonymous. These cases are considered unexpected and handled as serious expedited cases by pharmacovigilance personnel. Such cases are also considered for reporting as product defects, if appropriate.

### 10.3.3. Recording/Reporting and Follow-up of AEs and/or SAEs

**AE and SAE Recording/Reporting**

The table below summarizes the requirements for recording adverse events on the CRF and for reporting serious adverse events on the CT SAE Report Form to Pfizer Safety. These requirements are delineated for 3 types of events: (1) SAEs; (2) nonserious adverse events (AEs); and (3) exposure to the study intervention under study during pregnancy or breastfeeding, and occupational exposure.

It should be noted that the CT SAE Report Form for reporting of SAE information is not the same as the AE page of the CRF. When the same data are collected, the forms must be completed in a consistent manner. AEs should be recorded using concise medical terminology and the same AE term should be used on both the CRF and the CT SAE Report Form for reporting of SAE information.

| Safety Event | Recorded on the CRF | Reported on the CT SAE Report Form to Pfizer Safety Within 24 Hours of Awareness |
|---|---|---|
| SAE | All | All |
| Nonserious AE | All | None |

090177e194745c57\Approved\Approved On: 22-Jul-2020 00:48 (GMT)

| | | |
|---|---|---|
| Exposure to the study intervention under study during pregnancy or breastfeeding, and occupational exposure | All AEs/SAEs associated with exposure during pregnancy or breastfeeding<br><br>Occupational exposure is not recorded. | All (and EDP supplemental form for EDP)<br>Note: Include all SAEs associated with exposure during pregnancy or breastfeeding. Include all AEs/SAEs associated with occupational exposure. |

- When an AE/SAE occurs, it is the responsibility of the investigator to review all documentation (eg, hospital progress notes, laboratory reports, and diagnostic reports) related to the event.
- The investigator will then record all relevant AE/SAE information in the CRF.
- It is **not** acceptable for the investigator to send photocopies of the participant's medical records to Pfizer Safety in lieu of completion of the CT SAE Report Form/AE/SAE CRF page.
- There may be instances when copies of medical records for certain cases are requested by Pfizer Safety. In this case, all participant identifiers, with the exception of the participant number, will be redacted on the copies of the medical records before submission to Pfizer Safety.
- The investigator will attempt to establish a diagnosis of the event based on signs, symptoms, and/or other clinical information. Whenever possible, the diagnosis (not the individual signs/symptoms) will be documented as the AE/SAE.

**Assessment of Intensity**

The investigator will make an assessment of intensity for each AE and SAE reported during the study and assign it to 1 of the following categories:

| GRADE | Clinical Description of Severity |
|---|---|
| 1 | MILD adverse event |
| 2 | MODERATE adverse event |
| 3 | SEVERE adverse event |
| 4 | LIFE-THREATENING consequences; urgent intervention indicated |
| 5 | DEATH RELATED TO adverse event |

090177e194745c57\Approved\Approved On: 22-Jul-2020 00:48 (GMT)

| **Assessment of Causality** |
|---|
| • The investigator is obligated to assess the relationship between study intervention and each occurrence of each AE/SAE.<br><br>• A "reasonable possibility" of a relationship conveys that there are facts, evidence, and/or arguments to suggest a causal relationship, rather than a relationship cannot be ruled out.<br><br>• The investigator will use clinical judgment to determine the relationship.<br><br>• Alternative causes, such as underlying disease(s), concomitant therapy, and other risk factors, as well as the temporal relationship of the event to study intervention administration, will be considered and investigated.<br><br>• The investigator will also consult the IB and/or product information, for marketed products, in his/her assessment.<br><br>• For each AE/SAE, the investigator **must** document in the medical notes that he/she has reviewed the AE/SAE and has provided an assessment of causality.<br><br>• There may be situations in which an SAE has occurred and the investigator has minimal information to include in the initial report to the sponsor. However, **it is very important that the investigator always make an assessment of causality for every event before the initial transmission of the SAE data to the sponsor.**<br><br>• The investigator may change his/her opinion of causality in light of follow-up information and send an SAE follow-up report with the updated causality assessment.<br><br>• The causality assessment is one of the criteria used when determining regulatory reporting requirements.<br><br>• If the investigator does not know whether or not the study intervention caused the event, then the event will be handled as "related to study intervention" for reporting purposes, as defined by the sponsor. In addition, if the investigator determines that an SAE is associated with study procedures, the investigator must record this causal relationship in the source documents and CRF, and report such an assessment in the dedicated section of the CT SAE Report Form and in accordance with the SAE reporting requirements. |

090177e194745c57\Approved\Approved On: 22-Jul-2020 00:48 (GMT)

| Follow-up of AEs and SAEs |
| --- |
| • The investigator is obligated to perform or arrange for the conduct of supplemental measurements and/or evaluations as medically indicated or as requested by the sponsor to elucidate the nature and/or causality of the AE or SAE as fully as possible. This may include additional laboratory tests or investigations, histopathological examinations, or consultation with other healthcare providers.<br>• If a participant dies during participation in the study or during a recognized follow-up period, the investigator will provide Pfizer Safety with a copy of any postmortem findings including histopathology.<br>• New or updated information will be recorded in the originally completed CRF.<br>• The investigator will submit any updated SAE data to the sponsor within 24 hours of receipt of the information. |

### 10.3.4. Reporting of SAEs

| SAE Reporting to Pfizer Safety via an Electronic Data Collection Tool |
| --- |
| • The primary mechanism for reporting an SAE to Pfizer Safety will be the electronic data collection tool.<br>• If the electronic system is unavailable, then the site will use the paper SAE data collection tool (see next section) in order to report the event within 24 hours.<br>• The site will enter the SAE data into the electronic system as soon as the data become available.<br>• After the study is completed at a given site, the electronic data collection tool will be taken off-line to prevent the entry of new data or changes to existing data.<br>• If a site receives a report of a new SAE from a study participant or receives updated data on a previously reported SAE after the electronic data collection tool has been taken off-line, then the site can report this information on a paper SAE form (see next section) or to Pfizer Safety by telephone. |

090177e194745c57\Approved\Approved On: 22-Jul-2020 00:48 (GMT)

| SAE Reporting to Pfizer Safety via CT SAE Report Form |
| --- |
| • Facsimile transmission of the CT SAE Report Form is the preferred method to transmit this information to Pfizer Safety.<br>• In circumstances when the facsimile is not working, notification by telephone is acceptable with a copy of the CT SAE Report Form sent by overnight mail or courier service.<br>• Initial notification via telephone does not replace the need for the investigator to complete and sign the CT SAE Report Form pages within the designated reporting time frames. |

090177e194745c57\Approved\Approved On: 22-Jul-2020 00:48 (GMT)

## 10.4. Appendix 4: Contraceptive Guidance

### 10.4.1. Male Participant Reproductive Inclusion Criteria

Male participants are eligible to participate if they agree to the following requirements during the intervention period and for at least 3 months after the last dose of study intervention, which corresponds to the time needed to eliminate reproductive safety risk of the study intervention(s):

- Refrain from donating sperm.

PLUS either**:**

- Be abstinent from heterosexual intercourse with a female of childbearing potential as their preferred and usual lifestyle (abstinent on a long-term and persistent basis) and agree to remain abstinent.

  OR

- Must agree to use a male condom when engaging in any activity that allows for passage of ejaculate to another person.

### 10.4.2. Female Participant Reproductive Inclusion Criteria

A female participant is eligible to participate if she is not pregnant or breastfeeding, and at least 1 of the following conditions applies:

- Is not a WOCBP (see definitions below in Section 10.4.3).

  OR

- Is a WOCBP and using a contraceptive method that is highly effective (with a failure rate of <1% per year), preferably with low user dependency, as described below during the intervention period and for at least 6 months after the last dose of study intervention, which corresponds to the time needed to eliminate any reproductive safety risk of the study intervention(s). The investigator should evaluate the effectiveness of the contraceptive method in relationship to the first dose of study intervention.

- Is a WOCBP and using a contraceptive method that is highly effective (with a failure rate of <1% per year), with high user dependency, as described below during the intervention period and for at least 6 months after the last dose of study intervention, which corresponds to the time needed to eliminate any reproductive safety risk of the study intervention(s). In addition, a second effective method of contraception, as described below, must be used. The investigator should evaluate the effectiveness of the contraceptive method in relationship to the first dose of study intervention.

090177e194745c57\Approved\Approved On: 22-Jul-2020 00:48 (GMT)

The investigator is responsible for review of medical history, menstrual history, and recent sexual activity to decrease the risk for inclusion of a woman with an early undetected pregnancy.

### 10.4.3. Woman of Childbearing Potential

A woman is considered fertile following menarche and until becoming postmenopausal unless permanently sterile (see below).

If fertility is unclear (eg, amenorrhea in adolescents or athletes) and a menstrual cycle cannot be confirmed before the first dose of study intervention, additional evaluation should be considered.

Women in the following categories are not considered WOCBP:

1. Premenarchal.

2. Premenopausal female with 1 of the following:

    - Documented hysterectomy;
    - Documented bilateral salpingectomy;
    - Documented bilateral oophorectomy.

    For individuals with permanent infertility due to an alternate medical cause other than the above, (eg, mullerian agenesis, androgen insensitivity), investigator discretion should be applied to determining study entry.

    Note: Documentation for any of the above categories can come from the site personnel's review of the participant's medical records, medical examination, or medical history interview. The method of documentation should be recorded in the participant's medical record for the study.

3. Postmenopausal female:

    - A postmenopausal state is defined as no menses for 12 months without an alternative medical cause. In addition, a
        - high FSH level in the postmenopausal range must be used to confirm a postmenopausal state in women under 60 years of age and not using hormonal contraception or HRT.
        - Female on HRT and whose menopausal status is in doubt will be required to use one of the nonestrogen hormonal highly effective contraception methods if they wish to continue their HRT during the study. Otherwise, they must discontinue HRT to allow confirmation of postmenopausal status before study enrollment.



090177e194745c57\Approved\Approved On: 22-Jul-2020 00:48 (GMT)

### 10.4.4. Contraception Methods

Contraceptive use by men or women should be consistent with local availability/regulations regarding the use of contraceptive methods for those participating in clinical trials.

**Highly Effective Methods That Have Low User Dependency**

1. Implantable progestogen-only hormone contraception associated with inhibition of ovulation.
2. Intrauterine device.
3. Intrauterine hormone-releasing system.
4. Bilateral tubal occlusion.
5. Vasectomized partner.
    - Vasectomized partner is a highly effective contraceptive method provided that the partner is the sole sexual partner of the WOCBP and the absence of sperm has been confirmed. If not, an additional highly effective method of contraception should be used. The spermatogenesis cycle is approximately 90 days.

**Highly Effective Methods That Are User Dependent**

6. Combined (estrogen- and progestogen-containing) hormonal contraception associated with inhibition of ovulation:
    - Oral;
    - Intravaginal;
    - Transdermal;
    - Injectable.
7. Progestogen-only hormone contraception associated with inhibition of ovulation:
    - Oral;
    - Injectable.
8. Sexual abstinence:
    - Sexual abstinence is considered a highly effective method only if defined as refraining from heterosexual intercourse during the entire period of risk associated with the study intervention. The reliability of sexual abstinence needs to be

090177e194745c57\Approved\Approved On: 22-Jul-2020 00:48 (GMT)

evaluated in relation to the duration of the study and the preferred and usual lifestyle of the participant.

One of the following effective barrier methods must be used in addition to the highly effective methods listed above:

- Male or female condom with or without spermicide;
- Cervical cap, diaphragm, or sponge with spermicide;
- A combination of male condom with either cervical cap, diaphragm, or sponge with spermicide (double-barrier methods).

090177e194745c57\Approved\Approved On: 22-Jul-2020 00:48 (GMT)

## 10.5. Appendix 5: Genetics

### Use/Analysis of DNA

- Genetic variation may impact a participant's response to study intervention, susceptibility to, and severity and progression of disease. Therefore, where local regulations and IRBs/ethics committees allow, a blood sample will be collected for DNA analysis.

- The scope of the genetic research may be narrow (eg, 1 or more candidate genes) or broad (eg, the entire genome), as appropriate to the scientific question under investigation.

- The samples may be analyzed as part of a multistudy assessment of genetic factors involved in the response to the study interventions or study interventions of this class to understand treatments for the disease(s) under study or the disease(s) themselves.

- The results of genetic analyses may be reported in CSR or in a separate study summary, or may be used for internal decision making without being included in a study report.

- The sponsor will store the DNA samples in a secure storage space with adequate measures to protect confidentiality.

- The samples will be retained as indicated:

    - Samples for banking will be stored indefinitely or for another period as per local requirements.

- Participants may withdraw their consent/assent for the storage and/or use of their Banked Biospecimens at any time by making a request to the investigator; in this case, any remaining material will be destroyed. Data already generated from the samples will be retained to protect the integrity of existing analyses.

- Banked Biospecimens will be labeled with a code. The key between the code and the participant's personally identifying information (eg, name, address) will be held at the study site and will not be provided to the sample bank.

090177e194745c57\Approved\Approved On: 22-Jul-2020 00:48 (GMT)

## 10.6. Appendix 6: Liver Safety: Suggested Actions and Follow-up Assessments

### Potential Cases of Drug-Induced Liver Injury

Humans exposed to a drug who show no sign of liver injury (as determined by elevations in transaminases) are termed "tolerators," while those who show transient liver injury, but adapt are termed "adaptors." In some participants, transaminase elevations are a harbinger of a more serious potential outcome. These participants fail to adapt and therefore are "susceptible" to progressive and serious liver injury, commonly referred to as DILI. Participants who experience a transaminase elevation above 3 × ULN should be monitored more frequently to determine if they are an "adaptor" or are "susceptible."

In the majority of DILI cases, elevations in AST and/or ALT precede TBili elevations (>2 × ULN) by several days or weeks. The increase in TBili typically occurs while AST/ALT is/are still elevated above 3 × ULN (ie, AST/ALT and TBili values will be elevated within the same laboratory sample). In rare instances, by the time TBili elevations are detected, AST/ALT values might have decreased. This occurrence is still regarded as a potential DILI. Therefore, abnormal elevations in either AST OR ALT in addition to TBili that meet the criteria outlined below are considered potential DILI (assessed per Hy's law criteria) cases and should always be considered important medical events, even before all other possible causes of liver injury have been excluded.

The threshold of laboratory abnormalities for a potential DILI case depends on the participant's individual baseline values and underlying conditions. Participants who present with the following laboratory abnormalities should be evaluated further as potential DILI (Hy's law) cases to definitively determine the etiology of the abnormal laboratory values:

- Participants with AST/ALT and TBili baseline values within the normal range who subsequently present with AST OR ALT values >3 × ULN AND a TBili value >2 × ULN with no evidence of hemolysis and an alkaline phosphatase value <2 × ULN or not available.
- For participants with baseline AST **OR** ALT **OR** TBili values above the ULN, the following threshold values are used in the definition mentioned above, as needed, depending on which values are above the ULN at baseline:
    - Preexisting AST or ALT baseline values above the normal range: AST or ALT values >2 times the baseline values AND >3 × ULN; or >8 × ULN (whichever is smaller).
    - Preexisting values of TBili above the normal range: TBili level increased from baseline value by an amount of at least 1 × ULN **or** if the value reaches >3 × ULN (whichever is smaller).

Rises in AST/ALT and TBili separated by more than a few weeks should be assessed individually based on clinical judgment; any case where uncertainty remains as to whether it represents a potential Hy's law case should be reviewed with the sponsor.



090177e194745c57\Approved\Approved On: 22-Jul-2020 00:48 (GMT)

The participant should return to the investigator site and be evaluated as soon as possible, preferably within 48 hours from awareness of the abnormal results. This evaluation should include laboratory tests, detailed history, and physical assessment. The possibility of hepatic neoplasia (primary or secondary) should be considered.

In addition to repeating measurements of AST and ALT and TBili for suspected cases of Hy's law, additional laboratory tests should include albumin, CK, direct and indirect bilirubin, GGT, PT/INR, total bile acids, and alkaline phosphatase. Consideration should also be given to drawing a separate tube of clotted blood and an anticoagulated tube of blood for further testing, as needed, for further contemporaneous analyses at the time of the recognized initial abnormalities to determine etiology. A detailed history, including relevant information, such as review of ethanol, acetaminophen/paracetamol (either by itself or as a coformulated product in prescription or over-the-counter medications), recreational drug, supplement (herbal) use and consumption, family history, sexual history, travel history, history of contact with a jaundiced person, surgery, blood transfusion, history of liver or allergic disease, and potential occupational exposure to chemicals, should be collected. Further testing for acute hepatitis A, B, C, D, and E infection and liver imaging (eg, biliary tract) and collection of serum samples for acetaminophen/paracetamol drug and/or protein adduct levels may be warranted.

All cases demonstrated on repeat testing as meeting the laboratory criteria of AST/ALT and TBili elevation defined above should be considered potential DILI (Hy's law) cases if no other reason for the LFT abnormalities has yet been found. **Such potential DILI (Hy's law) cases are to be reported as SAEs, irrespective of availability of all the results of the investigations performed to determine etiology of the LFT abnormalities.**

A potential DILI (Hy's law) case becomes a confirmed case only after all results of reasonable investigations have been received and have excluded an alternative etiology.

090177e194745c57\Approved\Approved On: 22-Jul-2020 00:48 (GMT)

## 10.7. Appendix 7: ECG Findings of Potential Clinical Concern

| **ECG Findings That <u>May</u> Qualify as Adverse Events** |
|---|
| • Marked sinus bradycardia (rate <40 bpm) lasting minutes.<br>• New PR interval prolongation >280 msec.<br>• New prolongation of QTcF to >480 msec (absolute) or by >60 msec from baseline.<br>• New-onset atrial flutter or fibrillation, with controlled ventricular response rate: ie, rate <120 bpm.<br>• New-onset type I second-degree (Wenckebach) AV block of >30 seconds' duration.<br>• Frequent PVCs, triplets, or short intervals (<30 seconds) of consecutive ventricular complexes. |
| **ECG Findings That <u>May</u> Qualify as Serious Adverse Events** |
| • QTcF prolongation >500 msec.<br>• New ST-T changes suggestive of myocardial ischemia.<br>• New-onset left bundle branch block (QRS >120 msec).<br>• New-onset right bundle branch block (QRS >120 msec).<br>• Symptomatic bradycardia.<br>• Asystole:<br>  ◦ In awake, symptom-free patients in sinus rhythm, with documented periods of asystole ≥3.0 seconds or any escape rate <40 bpm, or with an escape rhythm that is below the AV node;<br>  ◦ In awake, symptom-free patients with atrial fibrillation and bradycardia with 1 or more pauses of at least 5 seconds or longer;<br>  ◦ Atrial flutter or fibrillation, with rapid ventricular response rate: rapid = rate >120 bpm.<br>• Sustained supraventricular tachycardia (rate >120 bpm) ("sustained" = short duration with relevant symptoms or lasting >1 minute). |

090177e194745c57\Approved\Approved On: 22-Jul-2020 00:48 (GMT)

- Ventricular rhythms >30 seconds' duration, including idioventricular rhythm (heart rate <40 bpm), accelerated idioventricular rhythm (HR 40 bpm to <100 bpm), and monomorphic/polymorphic ventricular tachycardia (HR >100 bpm (such as torsades de pointes)).
- Type II second-degree (Mobitz II) AV block.
- Complete (third-degree) heart block.

**ECG Findings That Qualify as Serious Adverse Events**

- Change in pattern suggestive of new myocardial infarction.
- Sustained ventricular tachyarrhythmias (>30 seconds' duration).
- Second- or third-degree AV block requiring pacemaker placement.
- Asystolic pauses requiring pacemaker placement.
- Atrial flutter or fibrillation with rapid ventricular response requiring cardioversion.
- Ventricular fibrillation/flutter.
- At the discretion of the investigator, any arrhythmia classified as an adverse experience.

The enumerated list of major events of potential clinical concern are recommended as "alerts" or notifications from the core ECG laboratory to the investigator and Pfizer study team, and not to be considered as all inclusive of what to be reported as AEs/SAEs.

090177e194745c57\Approved\Approved On: 22-Jul-2020 00:48 (GMT)

## 10.8. Appendix 8: Country-Specific Requirements

### 10.8.1. France Contract Unique

1. **GCP Training**

   Before enrolling any participants, the investigator and any subinvestigators will complete the Pfizer-provided Good Clinical Practice training course ("Pfizer GCP Training") or training deemed equivalent by Pfizer. Any investigators who later join the study will do the same before performing study-related duties. For studies of applicable duration, the investigator and subinvestigators will complete Pfizer GCP Training or equivalent every 3 years during the term of the study, or more often if there are significant changes to the ICH GCP guidelines or course materials.

2. **Study Intervention**

   No participants or third-party payers will be charged for study intervention.

3. **Urgent Safety Measures**

   In addition, the investigator will inform Pfizer immediately of any urgent safety measures taken by the investigator to protect the study participants against any immediate hazard, and of any serious breaches of this protocol or of ICH GCP that the investigator becomes aware of.

4. **Termination Rights**

   Pfizer retains the right to discontinue development of encorafenib at any time.

090177e194745c57\Approved\Approved On: 22-Jul-2020 00:48 (GMT)

## 10.9. Appendix 9: RECIST 1.1

## CATEGORIZING LESIONS AT BASELINE

Measurable Lesions

Lesions that can be accurately measured in at least one dimension.

- Lesions with longest diameter twice the slice thickness and at least 10 mm or greater when assessed by computed tomography or MRI (slice thickness 5-8 mm).
- Lesions with longest diameter at least 20 mm when assessed by chest X-ray.
- Superficial lesions with longest diameter 10 mm or greater when assessed by caliper.
- Malignant lymph nodes with the short axis 15 mm or greater when assessed by computed tomography.

**NOTE: The shortest axis is used as the diameter for malignant lymph nodes, longest axis for all other measurable lesions.**

Non-measurable disease

Non-measurable disease includes lesions too small to be considered measurable (including nodes with short axis between 10 and <15 mm) and truly non-measurable disease such as pleural or pericardial effusions, ascites, inflammatory breast disease, leptomeningeal disease, lymphangitic involvement of skin or lung, clinical lesions that cannot be accurately measured with calipers, abdominal masses identified by physical exam that are not measurable by reproducible imaging techniques.

- Bone disease: Bone disease is non-measurable with the exception of soft tissue components that can be evaluated by computed tomography or MRI and meet the definition of measurability at baseline.
- Previous local treatment: A previously irradiated lesion (or lesion subjected to other local treatment) is non-measurable unless it has progressed since completion of treatment.

Normal sites

- Cystic lesions: Simple cysts should not be considered as malignant lesions and should not be recorded either as target or non-target disease. Cystic lesions thought to represent cystic metastases can be measurable lesions, if they meet the specific definition above. If non-cystic lesions are also present, these are preferred as target lesions.
- Normal nodes: Nodes with short axis <10 mm are considered normal and should not be recorded or followed either as measurable or non-measurable disease.

090177e194745c57\Approved\Approved On: 22-Jul-2020 00:48 (GMT)

090177e194745c57\Approved\Approved On: 22-Jul-2020 00:48 (GMT)

## RECORDING TUMOR ASSESSMENTS

All sites of disease must be assessed at baseline. Baseline assessments should be done as close as possible prior to study start. For an adequate baseline assessment, all required scans must be done within 28 days prior to treatment and all disease must be documented appropriately. If baseline assessment is inadequate, subsequent statuses generally should be indeterminate.

Target lesions

- All measurable lesions up to a maximum of 2 lesions per organ, 5 lesions in total, representative of all involved organs, should be identified as target lesions at baseline. Target lesions should be selected on the basis of size (longest lesions) and suitability for accurate repeated measurements. Record the longest diameter for each lesion, except in the case of pathological lymph nodes for which the short axis should be recorded. The sum of the diameters (longest for non-nodal lesions, short axis for nodal lesions) for all target lesions at baseline will be the basis for comparison to assessments performed post-baseline.
- If two target lesions coalesce the measurement of the coalesced mass is used. If a large target lesion splits, the sum of the parts is used.
- Measurements for target lesions that become small should continue to be recorded. If the lesion is considered to have disappeared, 0 mm should be recorded; otherwise if a lesion is determined to be present but too small to measure, the lesion status will indicate "too small to measure and judged to be less than 10 mm" and 5 mm will be used in the calculation of the sum of the diameters.

**NOTE: When nodal lesions decrease to <10 mm (normal), the actual measurement should still be recorded.**

Non-target disease

- All non-measurable disease is non-target. All measurable lesions not identified as target lesions are also included as non-target disease. Measurements are not required but rather assessments will be expressed as ABSENT, INDETERMINATE, PRESENT/NOT INCREASED, INCREASED. Multiple non-target lesions in one organ may be recorded as a single item on the case report form (eg, 'multiple enlarged pelvic lymph nodes' or 'multiple liver metastases').

## OBJECTIVE RESPONSE STATUS AT EACH EVALUATION.

Disease sites must be assessed using the same technique as baseline, including consistent administration of contrast and timing of scanning. If a change needs to be made the case must be discussed with the Investigator or radiologist and the sponsor to determine if substitution is possible. If not, subsequent objective statuses are not evaluable.

090177e194745c57\Approved\Approved On: 22-Jul-2020 00:48 (GMT)

Target disease

- Complete Response (CR): Complete disappearance of all target lesions with the exception of nodal disease. All target nodes must decrease to normal size (short axis <10 mm). All target lesions must be assessed.
- Partial Response (PR): Greater than or equal to 30% decrease under baseline of the sum of diameters of all target measurable lesions. All target lesions must be assessed.
- Stable Disease (SD): Does not qualify for CR, PR or Progression. All target lesions must be assessed. Stable can follow PR only in the rare case that the sum increases by less than 20% from the nadir (smallest sum of diameters; consider baseline and all assessments prior to the time point under evaluation), but enough that a previously documented 30% decrease no longer holds.
- Objective Progression (PD): 20% increase in the sum of diameters of target measurable lesions above the smallest sum observed (over baseline if no decrease in the sum is observed during therapy), with a minimum absolute increase of 5 mm.
- Not evaluable (NE): Progression has not been documented, and
  - one or more target lesions have not been assessed or
  - assessment methods used were inconsistent with those used at baseline or
  - one or more target lesions cannot be measured accurately (eg, poorly visible unless due to being too small to measure) or
  - one or more target lesions were excised or irradiated and have not reappeared or increased.

Non-target disease

- CR: Disappearance of all non-target lesions and normalization of tumor marker levels (if being followed). All lymph nodes must be 'normal' in size (<10 mm short axis).
- Non-CR/Non-PD: Persistence of any non-target lesions and/or tumor marker level (if being followed) above the normal limits.
- PD: Unequivocal progression of pre-existing lesions. Generally the overall tumor burden must increase sufficiently to merit discontinuation of therapy. In the presence of SD or PR in target disease, progression due to unequivocal increase in non-target disease should be rare.
- Not evaluable (NE): Progression has not been determined and
  - one or more non-target lesion sites have not been assessed or
  - assessment methods used were inconsistent with those used at baseline or
  - one or more non-target lesions cannot be assessed (eg, poorly visible or unclear images) or

- one or more non-target lesions were excised or irradiated and have not reappeared or increased.

New Lesions

The appearance of any new unequivocal malignant lesion indicates PD. If a new lesion is equivocal, for example due to its small size, continued assessment will clarify the etiology. If repeat assessments confirm the lesion, then progression should be recorded on the date of the initial assessment. A lesion identified in an area not previously scanned will be considered a new lesion.

Supplemental Investigations

- If CR determination depends on a residual lesion that decreased in size but did not disappear completely, it is recommended the residual lesion be investigated with biopsy or fine needle aspirate. If no disease is identified, objective status is CR.
- If progression determination depends on a lesion with an increase possibly due to necrosis, the lesion may be investigated with biopsy or fine needle aspirate to clarify status.

Subjective progression

Participants requiring discontinuation of treatment without objective evidence of disease progression should not be reported as PD on tumor assessment CRFs. This should be indicated on the end of treatment CRF as off treatment due to Global Deterioration of Health Status. Every effort should be made to document PD even after discontinuation of study treatment.

090177e194745c57\Approved\Approved On: 22-Jul-2020 00:48 (GMT)

## 10.10. Appendix 10: Patient Global Impression of Severity and Patient Global Impression of Change

### Patient Global Impression of Severity

Right now, my colorectal cancer symptoms are (choose one response):.

- None
- Mild
- Moderate
- Severe
- Very severe

### Patient Global Impression of Change

Since starting the study medication, my colorectal cancer symptoms are:

- Greatly improved
- Moderately improved
- Slightly improved
- Not changed
- Slightly worsened
- Moderately worsened
- Greatly worsened

090177e194745c57\Approved\Approved On: 22-Jul-2020 00:48 (GMT)

## 10.11. Appendix 11: Abbreviations

The following is a list of abbreviations that may be used in the protocol.

| Abbreviation | Term |
|---|---|
| 5-FU | fluorouracil |
| Abs | absolute |
| ACE | angiotensin-converting enzyme |
| ADR | adverse drug reaction |
| AE | adverse event |
| ALT | alanine aminotransferase |
| ANC | absolute neutrophil count |
| aPTT | activated partial thromboplastin time |
| Arm C | Control Arm |
| ASCO | American Society of Clinical Oncology |
| AST | aspartate aminotransferase |
| ATP | adenosine triphosphate |
| AUC | area under the concentration-time curve |
| AV | atrioventricular |
| BCRP | breast cancer-resistance protein |
| β-hCG | beta-human chorionic gonadotropin |
| BICR | blinded independent central review |
| BID | twice a day |
| BOR | best overall response |
| Bpm | beats per minute |
| *BRAF* | B-Raf proto-oncogene |
| BRAF | serine/threonine-protein kinase B-Raf |
| BSA | body surface area |
| BUN | blood urea nitrogen |
| CAP | College of American Pathologists |
| CAPOX | capecitabine/oxaliplatin |
| CD4 | cluster of differentiation 4 |
| CDx | companion diagnostic |
| CE | Conformité Européene |
| CEA | carcinoembryonic antigen |
| cfDNA | circulating free DNA |
| CFR | Code of Federal Regulations |
| CHMP | Committee for Medicinal Products for Human Use |
| CI | confidence interval |
| CIOMS | Council for International Organizations of Medical Sciences |
| CK | creatine kinase |
| CLIA | Clinical Laboratory Improvement Amendments |
| $C_{max}$ | maximum concentration |
| $C_{min}$ | minimum concentration |
| CNS | central nervous system |



090177e194745c57\Approved\Approved On: 22-Jul-2020 00:48 (GMT)

| Abbreviation | Term |
|---|---|
| CO2 | carbon dioxide (bicarbonate) |
| CONSORT | Consolidated Standards of Reporting Trials |
| CR | complete response |
| CRC | colorectal cancer |
| CRF | case report form |
| CRO | contract research organization |
| CRP | C-reactive protein |
| CSR | Clinical Study Report |
| CT | clinical trial; computed tomography |
| CTCAE | Common Terminology Criteria for Adverse Events |
| ctDNA | circulating tumor DNA |
| CYP | cytochrome P450 enzyme (1A2, 2B6, 2C8, 2C9, 2C19 and 3A4 refer to isoforms) |
| DDI | drug-drug interaction |
| DDR | DNA damage response and repair |
| DILI | drug-induced liver injury |
| DLT | dose-limiting toxicity |
| dMMR | deficient mismatch repair |
| DOR | duration of response |
| DPD | dihydropyrimidine dehydrogenase |
| DRE | disease-related event |
| EC | encorafenib plus cetuximab |
| ECG | electrocardiogram |
| ECOG PS | Eastern Cooperative Oncology Group Performance Status |
| eCRF | electronic case report form |
| E-DMC | External Data Monitoring Committee |
| EDP | exposure during pregnancy |
| EGFR | epidermal growth factor receptor |
| EMA | European Medicines Agency |
| EORTC QLQ-C30 | European Organisation for Research and Treatment of Cancer Quality of Life Questionnaire for Cancer Patients – 30 Item Core Questionnaire |
| EOT | end of treatment |
| EQ-5D-5L | EuroQol-5D-5L |
| EQ VAS | EuroQol-visual analogue scale |
| ERK | extracellular signal-regulated kinase |
| ESMO | European Society for Medical Oncology |
| EU | European Union |
| EudraCT | European Clinical Trials Database |
| FAS | full analyses set |
| FDA | Food and Drug Administration |
| FDG-PET | fluorodeoxyglucose-positron emission tomography |
| FFPE | formalin-fixed paraffin-embedded |
| FOLFIRI | fluorouracil/leucovorin/irinotecan |



090177e194745c57\Approved\Approved On: 22-Jul-2020 00:48 (GMT)

| Abbreviation | Term |
|---|---|
| FOLFOX | fluorouracil/leucovorin/oxaliplatin |
| FOLFOXIRI | fluorouracil/leucovorin/oxaliplatin/irinotecan |
| FSH | follicle-stimulating hormone |
| GCP | Good Clinical Practice |
| GGT | gamma-glutamyl transferase |
| GI | gastrointestinal |
| HBcAb | hepatitis B core antibody |
| HBsAg | hepatitis B surface antigen |
| HBV | hepatitis B virus |
| HCV | hepatitis C virus |
| HFSR | hand foot skin reaction |
| HGRAC | Human Genetics Resources Administration of China |
| HIPAA | Health Insurance Portability and Accountability Act |
| HIV | human immunodeficiency virus |
| HR | hazard ratio; heart rate |
| HRT | hormone replacement therapy |
| HRQoL | Health-related quality of life |
| IB | Investigator’s Brochure |
| ICD | informed consent/assent document |
| ICH | International Council for Harmonisation |
| IEC | Independent Ethics Committee |
| IMP | investigational medicinal product |
| IND | Investigational New Drug |
| INR | international normalized ratio |
| IP manual | investigational product manual |
| IPAL | Investigational Product Accountability Log |
| IRB | Institutional Review Board |
| IRT | Interactive Response Technology |
| IV | intravenous |
| JSMO | Japanese Society of Medical Oncology |
| KA | keratoacanthoma |
| *KRAS* | KRAS proto-oncogene |
| LDH | lactate dehydrogenase |
| LFT | liver function test |
| LHY746 | encorafenib metabolite |
| LQTS | long QT syndrome |
| LV | left ventricular |
| mCRC | metastatic colorectal cancer |
| MDRD | modification of diet in renal disease |
| MedDRA | Medical Dictionary for Regulatory Activities |
| MEK | mitogen-activated protein kinase kinase |
| mFOLFOX6 | modified fluorouracil/leucovorin/oxaliplatin |
| MMR | mismatch repair |



090177e194745c57\Approved\Approved On: 22-Jul-2020 00:48 (GMT)

| Abbreviation | Term |
|---|---|
| mOS | median overall survival |
| mPFS | median progression-free survival |
| MRI | magnetic resonance imaging |
| MSI | microsatellite instability |
| MSI-H | microsatellite instability-high |
| MSS | microsatellite stable |
| MTD | maximum tolerated dose |
| n, N | number |
| NaF-PET | sodium fluoride-positron emission tomography |
| NCCN | National Comprehensive Cancer Network |
| NCI | National Cancer Institute |
| NCIC | National Cancer Information Center |
| NE | not evaluable |
| NGS | next generation sequencing |
| NIMP | noninvestigational medicinal product |
| NR | not reached |
| NRAS | NRAS Proto-Oncogene, GTPase |
| NTI | narrow therapeutic index |
| NYHA | New York Heart Association |
| OAT | organic anion transporter (1 and 3 refer to isoforms) |
| OATP | organic anion transporting polypeptide (1B1 and 1B3 refer to isoforms) |
| OCT | organic cation transporter (2 refers to isoform) |
| ORR | objective response rate |
| OS | overall survival |
| PCR | polymerase chain reaction |
| PD | progressive disease |
| PFS | progression-free survival |
| PFS2 | progression after next line of therapy |
| PGIC | Patient Global Impression of Change |
| PGIS | Patient Global Impression of Severity |
| P-gp | P-glycoprotein |
| PICC | peripherally inserted central catheter |
| PK | pharmacokinetic(s) |
| pMMR | proficient mismatch repair |
| PO | oral |
| PR | partial response |
| PRES | posterior reversible encephalopathy syndrome |
| PRO | patient reported outcome |
| PT | preferred term; prothrombin time |
| PTT | partial thromboplastin time |
| PVC | premature ventricular contraction/complex |
| Q2W | every 2 weeks |
| Q6W | every 6 weeks |



090177e194745c57\Approved\Approved On: 22-Jul-2020 00:48 (GMT)

| Abbreviation | Term |
|---|---|
| Q8W | every 8 weeks |
| QALY | quality-adjusted life-year |
| QD | once daily |
| QoL | quality of life |
| QT | QT interval |
| QTc | corrected QT interval |
| QTcF | QTc corrected using Fridericia's formula |
| *RAS* | RAS family of proto-oncogenes |
| RAS | RAS GTPase |
| *RAF* | RAF family of proto-oncogenes |
| RAF | RAF GTPase |
| RBC | red blood cell |
| RECIST | Response Evaluation Criteria in Solid Tumors |
| RGQ | Rotor-Gene Q |
| RP2D | recommended Phase 2 dose |
| RPLS | reversible posterior leukoencephalopathy syndrome |
| SAE | serious adverse event |
| SAP | Statistical Analysis Plan |
| SARS-CoV-2 | severe acute respiratory syndrome coronavirus 2 |
| SC | steering committee |
| SCC | squamous cell carcinoma |
| SD | standard deviation; stable disease |
| SEER | surveillance, epidemiology, and end results |
| SLI | Safety Lead-in |
| SmPC | Summary of Product Characteristics |
| SN-38 | active metabolite of irinotecan |
| SN-38G | inactive metabolite of irinotecan |
| SoA | schedule of activities |
| SOC | standard of care |
| SOP | standard operating procedure |
| SRSD | single reference safety document |
| SUSAR | suspected unexpected serious adverse reaction |
| T | temperature |
| TBili | total bilirubin |
| TdP | Torsades de Pointes |
| TTR | time to response |
| UGT | uridine 5'-diphospho-glucuronosyltransferase (1A1 refers to isoform) |
| UK | United Kingdom |
| ULN | upper limit of normal |
| US | United States |
| USA | United States of America |
| USPI | United States Prescribing Information |
| VAF | variant allele fraction |



090177e194745c57\Approved\Approved On: 22-Jul-2020 00:48 (GMT)

| Abbreviation | Term |
|---|---|
| VEGF | vascular endothelial growth factor |
| WBC | white blood cell |
| WHO | World Health Organization |
| WOCBP | woman of childbearing potential |
| wt | wild type |

090177e194745c57\Approved\Approved On: 22-Jul-2020 00:48 (GMT)

## REFERENCES

1. Bray F, Ferlay J, Soerjomataram I, et al. Global cancer statistics 2018: GLOBOCAN estimates of incidence and mortality worldwide for 36 cancers in 185 countries. CA: a cancer journal for clinicians. 2018;68(6):394-424.

2. Howlader N NA, Krapcho M, Miller D, Brest A, Yu M, Ruhl J, Tatalovich Z, Mariotto A, Lewis DR, Chen HS, Feuer EJ, Cronin KA (eds). SEER Cancer Statistics Review, 1975-2016: National Cancer Institute. Bethesda, MD; 2019 [based on November 2018 SEER data submission, posted to the SEER web site]. Available from: https://seer.cancer.gov/csr/1975_2016/.

3. Cancer Statistics in Japan 2018: Foundation for Promotion of Cancer Research (FPCR); 2019 [Available from: https://ganjoho.jp/data/reg_stat/statistics/brochure/2018/cancer_statistics_2018.pdf.

4. Altobelli E, D'Aloisio F, Angeletti PM. Colorectal cancer screening in countries of European Council outside of the EU-28. World journal of gastroenterology. 2016;22(20):4946.

5. Van Cutsem E, Cervantes A, Nordlinger B, et al. Metastatic colorectal cancer: ESMO Clinical Practice Guidelines for diagnosis, treatment and follow-up. Annals of oncology. 2014;25(suppl_3):iii1-iii9.

6. Barras D, Missiaglia E, Wirapati P, et al. BRAF V600E mutant colorectal cancer subtypes based on gene expression. Clinical Cancer Research. 2017;23(1):104-15.

7. Bylsma LC, Gillezeau C, Garawin T, et al. Prevalence of RAS and BRAF mutations in metastatic colorectal cancer (mCRC) patients by tumor location. Journal of Clinical Oncology. 2018;36(4_suppl):681-.

8. Clarke CN, Kopetz ES. BRAF mutant colorectal cancer as a distinct subset of colorectal cancer: clinical characteristics, clinical behavior, and response to targeted therapies. Journal of gastrointestinal oncology. 2015;6(6):660.

9. Davies H, Bignell GR, Cox C, et al. Mutations of the BRAF gene in human cancer. Nature. 2002;417(6892):949-54.

10. De Roock W, Claes B, Bernasconi D, et al. Effects of KRAS, BRAF, NRAS, and PIK3CA mutations on the efficacy of cetuximab plus chemotherapy in chemotherapy-refractory metastatic colorectal cancer: a retrospective consortium analysis. The lancet oncology. 2010;11(8):753-62.

090177e194745c57\Approved\Approved On: 22-Jul-2020 00:48 (GMT)

11. Sorbye H, Dragomir A, Sundström M, et al. High BRAF mutation frequency and marked survival differences in subgroups according to KRAS/BRAF mutation status and tumor tissue availability in a prospective population-based metastatic colorectal cancer cohort. PLoS One. 2015;10(6).

12. Corcoran RB, Ebi H, Turke AB, et al. EGFR-mediated reactivation of MAPK signaling contributes to insensitivity of BRAF-mutant colorectal cancers to RAF inhibition with vemurafenib. Cancer discovery. 2012;2(3):227-35.

13. Yuan Z-X, Wang X-Y, Qin Q-Y, et al. The prognostic role of BRAF mutation in metastatic colorectal cancer receiving anti-EGFR monoclonal antibodies: a meta-analysis. PLoS one. 2013;8(6).

14. Lochhead P, Kuchiba A, Imamura Y, et al. Microsatellite instability and BRAF mutation testing in colorectal cancer prognostication. Journal of the National Cancer Institute. 2013;105(15):1151-6.

15. Van Cutsem E, Kohne C-H, Láng I, et al. Cetuximab plus irinotecan, fluorouracil, and leucovorin as first-line treatment for metastatic colorectal cancer: updated analysis of overall survival according to tumor KRAS and BRAF mutation status. Clin Oncol. 2011;29(15):2011-9.

16. Tran B, Kopetz S, Tie J, et al. Impact of BRAF mutation and microsatellite instability on the pattern of metastatic spread and prognosis in metastatic colorectal cancer. Cancer. 2011;117(20):4623-32.

17. Venderbosch S, Nagtegaal ID, Maughan TS, et al. Mismatch repair status and BRAF mutation status in metastatic colorectal cancer patients: a pooled analysis of the CAIRO, CAIRO2, COIN, and FOCUS studies. Clinical Cancer Research. 2014;20(20):5322-30.

18. Qin S, Li J, Wang L, et al. Efficacy and tolerability of first-line cetuximab plus leucovorin, fluorouracil, and oxaliplatin (FOLFOX-4) versus FOLFOX-4 in patients with RAS wild-type metastatic colorectal cancer: the open-label, randomized, phase III TAILOR trial. Journal of Clinical Oncology. 2018;36(30):3031.

19. Bokemeyer C, Van Cutsem E, Rougier P, et al. Addition of cetuximab to chemotherapy as first-line treatment for KRAS wild-type metastatic colorectal cancer: pooled analysis of the CRYSTAL and OPUS randomised clinical trials. European journal of cancer. 2012;48(10):1466-75.

20. Douillard J-Y, Oliner KS, Siena S, et al. Panitumumab–FOLFOX4 treatment and RAS mutations in colorectal cancer. New England Journal of Medicine. 2013;369(11):1023-34.



090177e194745c57\Approved\Approved On: 22-Jul-2020 00:48 (GMT)

21. Goey KK, Elias SG, van Tinteren H, et al. Maintenance treatment with capecitabine and bevacizumab versus observation in metastatic colorectal cancer: updated results and molecular subgroup analyses of the phase 3 CAIRO3 study. Annals of Oncology. 2017;28(9):2128-34.

22. Stintzing S, Miller-Phillips L, Modest D, et al. Impact of BRAF and RAS mutations on first-line efficacy of FOLFIRI plus cetuximab versus FOLFIRI plus bevacizumab: analysis of the FIRE-3 (AIO KRK-0306) study. European Journal of Cancer. 2017;79:50-60.

23. Cremolini C, Loupakis F, Antoniotti C, et al. FOLFOXIRI plus bevacizumab versus FOLFIRI plus bevacizumab as first-line treatment of patients with metastatic colorectal cancer: updated overall survival and molecular subgroup analyses of the open-label, phase 3 TRIBE study. The Lancet Oncology. 2015;16(13):1306-15.

24. Overman MJ, McDermott R, Leach JL, et al. Nivolumab in patients with metastatic DNA mismatch repair-deficient or microsatellite instability-high colorectal cancer (CheckMate 142): an open-label, multicentre, phase 2 study. The Lancet Oncology. 2017;18(9):1182-91.

25. Le DT, Uram JN, Wang H, et al. PD-1 blockade in tumors with mismatch-repair deficiency. New England Journal of Medicine. 2015;372(26):2509-20.

26. Cohen R, Pellat A, Boussion H, et al. Immunotherapy and metastatic colorectal cancers with microsatellite instability or mismatch repair deficiency. Bulletin du cancer. 2019;106(2):137-42.

27. KEYTRUDA (pembrolizumab) USPI. Whitehouse Station, NJ, USA: Merck & Co. Inc.; 2020.

28. National Comprehensive Cancer Network. NCCN Clinical Practice Guidelines in Oncology (NCCN Guidelines®): Colon cancer, Version 2.2020 2020 [Available from: https://www.nccn.org/professionals/physician_gls/pdf/colon.pdf.

29. Investigator's Brochure, Encorafenib (PF-07263896). 24 June 2020.

30. BRAFTOVI (encorafenib) USPI. Boulder, CO, USA: Array BioPharma Inc.; 2020.

31. Kopetz S, Grothey A, Yaeger R, et al. Encorafenib, binimetinib, and cetuximab in BRAF V600E–mutated colorectal cancer. New England Journal of Medicine. 2019;381(17):1632-43.

090177e194745c57\Approved\Approved On: 22-Jul-2020 00:48 (GMT)

32. Chabot G, Abigerges D, Catimel G, et al. Population pharmacokinetics and pharmacodynamics of irinotecan (CPT-11) and active metabolite SN-38 during phase I trials. Annals of oncology. 1995;6(2):141-51.

33. de Man F, Goey AK, van Schaik RH, et al. Individualization of irinotecan treatment: a review of pharmacokinetics, pharmacodynamics, and pharmacogenetics. Clinical pharmacokinetics. 2018;57(10):1229-54.

34. CAMPTOSAR (irinotecan) USPI. NY, NY, USA: Pfizer Inc; 2020.

35. Smith NF, Figg WD, Sparreboom A. Pharmacogenetics of irinotecan metabolism and transport: an update. Toxicology in vitro. 2006;20(2):163-75.

36. Nozawa T, Minami H, Sugiura S, et al. Role of organic anion transporter OATP1B1 (OATP-C) in hepatic uptake of irinotecan and its active metabolite, 7-ethyl-10-hydroxycamptothecin: in vitro evidence and effect of single nucleotide polymorphisms. Drug Metabolism and Disposition. 2005;33(3):434-9.

37. Miura K, Kinouchi M, Ishida K, et al. 5-fu metabolism in cancer and orally-administrable 5-fu drugs. Cancers. 2010;2(3):1717-30.

38. Graham MA, Lockwood GF, Greenslade D, et al. Clinical pharmacokinetics of oxaliplatin: a critical review. Clinical Cancer Research. 2000;6(4):1205-18.

39. Leucovorin USPI. North Wales, PA: Teva Pharmaceuticals USA, Inc; 2018.

40. Van Cutsem E, Cervantes A, Adam R, et al. ESMO consensus guidelines for the management of patients with metastatic colorectal cancer. Annals of Oncology. 2016;27(8):1386-422.

41. Yoshino T, Arnold D, Taniguchi H, et al. Pan-Asian adapted ESMO consensus guidelines for the management of patients with metastatic colorectal cancer: a JSMO–ESMO initiative endorsed by CSCO, KACO, MOS, SSO and TOS. Annals of Oncology. 2018;29(1):44-70.

42. Cremolini C, Antoniotti C, Stein A, et al. FOLFOXIRI/bevacizumab (bev) versus doublets/bev as initial therapy of unresectable metastatic colorectal cancer (mCRC): A meta-analysis of individual patient data (IPD) from five randomized trials. Journal of Clinical Oncology. 2020;38(15_suppl):4015-.

090177e194745c57\Approved\Approved On: 22-Jul-2020 00:48 (GMT)

43. Loupakis F, Cremolini C, Masi G, et al. Initial therapy with FOLFOXIRI and bevacizumab for metastatic colorectal cancer. New England Journal of Medicine. 2014;371(17):1609-18.

44. US Food and Drug Administration. Guidance for Industry: Clinical Trial Endpoints for the Approval of Cancer Drugs and Biologics. Available from: https://www.fda.gov/regulatory-information/search-fda-guidance-documents/clinical-trial-endpoints-approval-cancer-drugs-and-biologics. Published: December 2018. Accessed 22 April 2020.

45. European Medicines Agency. Guideline on the evaluation of anticancer medicinal products in man. Available from: https://www.ema.europa.eu/en/documents/scientific-guideline/guideline-evaluation-anticancer-medicinal-products-man-revision-5_en.pdf. Published: September 2017. Accessed: 22 April 2020.

46. Zheng G, Tseng L-H, Haley L, et al. Clinical validation of coexisting driver mutations in colorectal cancers. Human pathology. 2019;86:12-20.

47. Richman SD, Seymour MT, Chambers P, et al. KRAS and BRAF mutations in advanced colorectal cancer are associated with poor prognosis but do not preclude benefit from oxaliplatin or irinotecan: results from the MRC FOCUS trial. Journal of Clinical Oncology. 2009;27(35):5931-7.

48. Grady WM, Carethers JM. Genomic and epigenetic instability in colorectal cancer pathogenesis. Gastroenterology. 2008;135(4):1079-99.

49. Sveen A, Kopetz S, Lothe RA. Biomarker-guided therapy for colorectal cancer: strength in complexity. Nature Reviews Clinical Oncology. 2019:1-22.

50. Thierry AR, Mouliere F, El Messaoudi S, et al. Clinical validation of the detection of KRAS and BRAF mutations from circulating tumor DNA. Nature medicine. 2014;20(4):430.

51. Hong DS, Morris VK, El Osta B, et al. Phase IB study of vemurafenib in combination with irinotecan and cetuximab in patients with metastatic colorectal cancer with BRAFV600E mutation. Cancer discovery. 2016;6(12):1352-65.

52. Corcoran RB, André T, Atreya CE, et al. Combined BRAF, EGFR, and MEK inhibition in patients with BRAFV600E-mutant colorectal cancer. Cancer discovery. 2018;8(4):428-43.

53. Tabernero J, Ciardiello F, Rivera F, et al. Cetuximab administered once every second week to patients with metastatic colorectal cancer: a two-part



090177e194745c57\Approved\Approved On: 22-Jul-2020 00:48 (GMT)

pharmacokinetic/pharmacodynamic phase I dose-escalation study. Annals of oncology. 2010;21(7):1537-45.

54. Martin-Martorell P, Rosello S, Rodriguez-Braun E, et al. Biweekly cetuximab and irinotecan in advanced colorectal cancer patients progressing after at least one previous line of chemotherapy: results of a phase II single institution trial. British journal of cancer. 2008;99(3):455-8.

55. US Food and Drug Administration. Patient-Focused Drug Development Guidance: Methods to Identify What is Important to Patients and Select, Develop or Modify Fit-for-Purpose Clinical Outcome Assessments. Available from: https://www.fda.gov/drugs/news-events-human-drugs/patient-focused-drug-development-guidance-methods-identify-what-important-patients-and-select. Published: October 2018. Accessed 03 May 2020. 2014.

56. Griggs JJ, Mangu PB, Temin S, et al. Appropriate chemotherapy dosing for obese adult patients with cancer: American Society of Clinical Oncology clinical practice guideline. Journal of oncology practice. 2012;8(4):e59.

57. Flaherty KT, Puzanov I, Kim KB, et al. Inhibition of mutated, activated BRAF in metastatic melanoma. New England Journal of Medicine. 2010;363(9):809-19.

58. Kefford R, Arkenau H, Brown MP, et al. Phase I/II study of GSK2118436, a selective inhibitor of oncogenic mutant BRAF kinase, in patients with metastatic melanoma and other solid tumors. Journal of Clinical Oncology. 2010;28(15_suppl):8503-.

59. Robert C, Arnault J-P, Mateus C. RAF inhibition and induction of cutaneous squamous cell carcinoma. Current opinion in oncology. 2011;23(2):177-82.

60. Yokota T, Ura T, Shibata N, et al. BRAF mutation is a powerful prognostic factor in advanced and recurrent colorectal cancer. British journal of cancer. 2011;104(5):856-62.

61. Maurer W, Bretz F. Multiple testing in group sequential trials using graphical approaches. Statistics in Biopharmaceutical Research. 2013;5(4):311-20.

62. Jennison C, Turnbull BW. Group sequential methods with applications to clinical trials: CRC Press; 1999.

63. Lan K, DeMets DL. Discrete sequential boundaries for clinical trials. Biometrika. 1983;70(3):659-63.

090177e194745c57\Approved\Approved On: 22-Jul-2020 00:48 (GMT)

090177e1a02cb071\Approved\Approved On: 14-Mar-2024 15:31 (GMT)

# Title Page

# AN OPEN-LABEL, MULTICENTER, RANDOMIZED PHASE 3 STUDY OF FIRST-LINE ENCORAFENIB PLUS CETUXIMAB WITH OR WITHOUT CHEMOTHERAPY VERSUS STANDARD OF CARE THERAPY WITH A SAFETY LEAD-IN OF ENCORAFENIB AND CETUXIMAB PLUS CHEMOTHERAPY IN PARTICIPANTS WITH METASTATIC BRAF V600E-MUTANT COLORECTAL CANCER

| | |
|---|---|
| **Study Intervention Number:** | PF-07263896; LGX818; ONO-7702 |
| **Study Intervention Name:** | Encorafenib |
| **US IND Number:** | 115298 |
| **EudraCT / EU CT Number:** | 2020-001288-99 / 2023-509405-77-00 |
| **ClinicalTrials.gov ID:** | NCT04607421 |
| **Pediatric Investigational Plan Number:** | NA |
| **Protocol Number:** | C4221015 |
| **Phase:** | 3 |
| **Sponsor Legal Address:** | Pfizer Inc.<br>66 Hudson Boulevard East<br>New York, NY 10001 |

**SHORT TITLE:** The BREAKWATER Study (***BR**AF* V600E-mutant colorectal cancer study evaluating **E**ncor**A**fenib ta**K**en **W**ith cetuxim**A**b plus or minus chemo**T**h**ER**apy)

This document and accompanying materials contain confidential information belonging to Pfizer. Except as otherwise agreed to in writing, by accepting or reviewing these documents, you agree to hold this information in confidence and not copy or disclose it to others (except where required by applicable law) or use it for unauthorized purposes. In the event of any actual or suspected breach of this obligation, Pfizer must be promptly notified.

## Document History

| Document | Version Date |
|---|---|
| Amendment 6 | 13 March 2024 |
| Amendment 5 | 20 December 2022 |
| Amendment 4 | 28 February 2022 |
| Amendment 3 | 24 February 2021 |
| Amendment 2 | 12 November 2020 |
| Amendment 1 | 07 August 2020 |
| Original protocol | 15 July 2020 |

The main purpose of Protocol Amendment 6 is to modify the required number of PFS events by BICR needed to conduct the primary PFS analysis for the Phase 3 portion of the study to enable a timely readout of the PFS given a slower-than-expected accrual of events. In addition, this amendment incorporates all revisions to date, including amendments made at the request of country health authorities and IRBs/ethics committees and any protocol administrative change letter(s).

## Protocol Amendment Summary of Changes Table

| Description of Change | Brief Rationale | Section # and Name |
|---|---|---|
| **Substantial Modification(s)** | | |
| Modified the power and the number of PFS events by BICR required for the Phase 3 PFS primary endpoint and removed the sentence about duration of follow up. | To enable a timely readout of the PFS given a slower-than-expected accrual of events. | Section 9.2 Sample Size Determination |
| Modified the number of PFS events by BICR required for the Phase 3 PFS primary endpoint and added the condition about minimal follow-up. Added a sentence that PFS of Cohort 3 will be analyzed descriptively if ORR analysis is not significant. | To align with revised sample size and specify that PFS for Cohort 3 will be analyzed descriptively if ORR analysis is not significant. | Section 9.4.1.1 Statistical Testing Strategy |
| **Non-Substantial Modification(s)** | | |
| Updated the secondary objective and endpoint pertaining to the correlation between ctDNA and outcome for both Phase 3 and Cohort 3. | To incorporate the observation from the ctMoniTR project that the presence or absence of detectable ctDNA was prognostic for PFS and OS following tyrosine kinase inhibitor treatment. | Section 1.1 Synopsis, Objectives, Endpoints, and Estimands<br>Section 3 Objectives, Estimands, and Endpoints |



090177e1a02cb071\Approved\Approved On: 14-Mar-2024 15:31 (GMT)

| Description of Change | Brief Rationale | Section # and Name |
|---|---|---|
| Added a new tertiary/exploratory objective and endpoint pertaining to the surgical conversion rate for both Phase 3 and Cohort 3. | To assess if study intervention enables patients to have surgical resections with curative intent. | Section 3 Objectives, Estimands, and Endpoints |
| Updated the population for the OR for Arm B vs Control Arm to the first 110 participants who are randomized in the Phase 3 portion of the study into each of Arm B and Arm C (Full Analysis Set, ORR Subset). | To clarify the population for the primary OR analysis so as to align with sample size determination in Section 9.2 as this change was missed in Protocol Amendment 5. | Section 9.1.1 Estimands |
| Updated the situation pertaining to 'no adequate baseline' assessment to include no disease at baseline. | To define the management of participants with no evidence of disease at baseline by BICR. | Section 9.1.1, Table 32: PFS Outcome and Event Dates – Primary Analysis |
| Modified the definition of new anticancer therapy by removing reference to target/non-target lesions. | To clarify that new treatments can pertain to any malignant lesion (eg, new lesions). | Section 9.1.1, Table 32: PFS Outcome and Event Dates – Primary Analysis<br>Section 9.4.2 Primary Endpoints |
| Added a 'Full Analysis Set, ORR Subset'. | To define the population used for the ORR primary analysis. | Section 9.3 Analysis Sets |
| Added a rule in the event that no efficacy assessment was performed before randomization, but was done before the start of study treatment. | To allow the evaluation of participants with such assessments. | Section 9.4.1.4 Definition of Baseline |
| Added a more detailed description of planned ctDNA analyses. | To incorporate recent findings from ctMoniTR project. | Section 9.4.3.11 Biomarker Analyses |
| Removed that details of IA for OS will be documented and approved in an E-DMC charter. | To clarify that details of IA for OS will not be provided in the E-DMC charter, as it will not be performed by E-DMC. | Section 9.5.1 Interim Efficacy Analysis for OS (Phase 3) |
| • Added that cumulative safety data will be reviewed by the E-DMC for Phase 3 and Cohort 3, in addition to the SLI.<br>• Added that ORR and DOR for Phase 3 and Cohort 3 will be evaluated by E-DMC. | To align with E-DMC charter. | Section 9.6 Data Monitoring Committee or Other Independent Oversight Committee |
| Added regulatory agency identification numbers and Sponsor address. | To comply with EU CTR content requirements. | Title page |
| • Defined abbreviations at first use and removed cross references to sections and | To comply with EU CTR content requirements. | Section 1.1 Synopsis |

090177e1a02cb071\Approved\Approved On: 14-Mar-2024 15:31 (GMT)

| Description of Change | Brief Rationale | Section # and Name |
|---|---|---|
| literature citations outside the synopsis.<br>• Added protocol title, brief title, and regulatory agency identification numbers.<br>• Removed tertiary/exploratory objectives and endpoints from the table in the synopsis for all 3 phases of the study.<br>• Added section on study population.<br>• Added section on ethical considerations. | | |
| Removed DDIs as a potential risk as this was removed from Section 2.3.1 in Protocol Amendment 5. | To incorporate a change that was missed in Protocol Amendment 5. | Section 2.3 Benefit/Risk Assessment |
| Updated the description and rationale for the ctDNA analyses. | To clarify that analyses are aligned with findings from ctMoniTR project. | Section 4.2.5 Biomarkers |
| • Added AxMP as a potential intervention classification type.<br>• Added a new row to include SRSD for each study intervention. | To comply with EU CTR content requirements. | Section 6.1 Study Interventions Administered |
| Updated the website link for CredibleMeds. | To provide a currently active website link. | Section 6.5.1.4 Drugs with a Conditional or Possible Risk to Prolong the QT Interval and/or Induce Torsade de Pointes |
| Updated the website link for Drug Interactions Solutions. | To provide a currently active website link. | Section 6.5.2 Prohibited Concomitant Therapy |
| Updated the analyses of cfDNA and added methylation changes to the description of possible assessments. | To adapt the description of assessments to the emerging data in the ctMoniTR project. | Section 8.8.3 Circulating Free DNA (cfDNA) |
| Incorporated protocol template updates throughout this section. | To align with current Pfizer protocol template. | Section 10.1 Appendix 1: Regulatory, Ethical, and Study Oversight Considerations |
| • Removed all text regarding the removal of Cohort 4.<br>• Corrected text pertaining to number of participants in Phase 3. | To correct errors in the SoC for Amendment 5. | Section 10.13, Appendix 13: Protocol Amendment History, Amendment 5 |



090177e1a02cb071\Approved\Approved On: 14-Mar-2024 15:31 (GMT)

| Description of Change | Brief Rationale | Section # and Name |
|---|---|---|
| The list of references was updated accordingly. | N/A | References |
| Editorial revisions and typographical errors were corrected. | To provide clarity, improve reliability, and maintain consistency throughout the protocol. | Throughout Protocol |
| Modified the resting period prior to an ECG from 10 minutes to 5 minutes. | To incorporate a correction that was implemented in a protocol administrative change letter dated 01 Aug 2023. | Section 8.2.7 Electrocardiograms |
| Modified the process for contacting a medically qualified individual from a medical escalation process via a Pfizer Call Center to direct clinical team contact using a Study Team Contact List. | To incorporate the change to the process for contacting a medically qualified individual that was implemented in a protocol administrative change letter dated 08 Feb 2024. | Section 10.1.10 Sponsor's Qualified Medical Personnel |

090177e1a02cb071\Approved\Approved On: 14-Mar-2024 15:31 (GMT)

# TABLE OF CONTENTS

LIST OF TABLES ....13
LIST OF FIGURES ....15
1. PROTOCOL SUMMARY ....16
1.1. Synopsis ....16
1.2. Schema ....24
1.3. Schedule of Activities ....24
2. INTRODUCTION ....60
2.1. Study Rationale ....60
2.2. Background ....60
2.2.1. *BRAF* V600E-Mutant Metastatic Colorectal Cancer ....60
2.2.2. Clinical Overview ....61
2.2.2.1. Current Recommendations for Treatment of *BRAF* V600E-mutant mCRC ....61
2.2.2.2. Encorafenib + Cetuximab for the Treatment of *BRAF* V600E-mutant mCRC ....62
2.2.2.3. Management of Cancer Patients During COVID-19 Pandemic ....64
2.3. Benefit/Risk Assessment ....64
2.3.1. Risk Assessment ....66
2.3.2. Benefit Assessment ....70
2.3.3. Overall Benefit/Risk Conclusion ....70
3. OBJECTIVES, ESTIMANDS, AND ENDPOINTS ....70
4. STUDY DESIGN ....74
4.1. Overall Design ....74
4.2. Scientific Rationale for Study Design ....75
4.2.1. Safety Lead-in ....75
4.2.2. Assessment of Potential DDI with Encorafenib ....75
4.2.3. Investigator's Choice of Control Regimen ....76
4.2.4. Progression-Free Survival and Objective Response Rate as Primary Endpoints ....77
4.2.5. Biomarkers ....77
4.2.6. Use of Contraceptives ....78



090177e1a02cb071\Approved\Approved On: 14-Mar-2024 15:31 (GMT)

4.3. Justification for Dose ....................78
4.4. End of Study Definition ....................79
5. STUDY POPULATION ....................79
5.1. Inclusion Criteria ....................79
5.1.1. Molecular Prescreening Inclusion Criteria ....................79
5.1.2. Screening Inclusion Criteria ....................80
5.2. Exclusion Criteria ....................82
5.2.1. Molecular Prescreening Exclusion Criteria ....................82
5.2.2. Screening Exclusion Criteria ....................83
5.3. Lifestyle Considerations ....................86
5.3.1. Contraception ....................86
5.3.2. Meals and Dietary Restrictions ....................87
5.3.3. Photosensitivity ....................87
5.3.4. Screen Failures ....................87
6. STUDY INTERVENTION ....................87
6.1. Study Interventions Administered ....................89
6.1.1. Safety Lead-in ....................90
6.1.1.1. SLI Dose Limiting Toxicity Definition ....................91
6.1.2. Treatment Regimens ....................92
6.1.3. Administration ....................96
6.1.3.1. Administration of Encorafenib ....................96
6.1.3.2. Administration of Capecitabine, Cetuximab, 5-Fluorouracil, Irinotecan, Leucovorin, Oxaliplatin and Bevacizumab ....................97
6.2. Preparation/Handling/Storage/Accountability ....................98
6.2.1. Preparation and Dispensing ....................99
6.3. Measures to Minimize Bias: Randomization and Blinding ....................99
6.3.1. Allocation to Study Intervention ....................99
6.4. Study Intervention Compliance ....................100
6.5. Concomitant Therapy ....................100
6.5.1. Permitted Concomitant Medications/Therapies ....................100
6.5.1.1. Surgical Resection ....................101



090177e1a02cb071\Approved\Approved On: 14-Mar-2024 15:31 (GMT)

6.5.1.2. CYP and UGT Substrates and Inhibitors ....101
6.5.1.3. Transporter Substrates and Inhibitors ....102
6.5.1.4. Drugs with a Conditional or Possible Risk to Prolong the QT Interval and/or Induce Torsade de Pointes ....102
6.5.2. Prohibited Concomitant Therapy ....102
6.5.3. Premedication and Concurrent Medication ....104
6.5.3.1. Premedication for Cetuximab Administration ....104
6.5.3.2. Loperamide ....104
6.5.3.3. Antibiotics ....104
6.5.3.4. Atropine ....104
6.5.3.5. Hematopoietic Growth Factors ....105
6.5.3.6. Antiemetics ....105
6.6. Dose Modification ....105
6.6.1. Dose Modifications for Encorafenib ....106
6.6.2. Dose Modifications for Cetuximab ....110
6.6.3. Dose Modifications for Oxaliplatin, Irinotecan, 5-FU and Capecitabine as Part of mFOLFOX6, FOLFIRI, FOLFOXIRI and CAPOX Regimens ....111
6.6.4. Dose Modifications for Bevacizumab ....118
6.6.5. Guidance For Administration of Study Intervention in Participants with SARS-CoV-2 Infection ....121
6.7. Intervention After the End of the Study ....121
7. DISCONTINUATION OF STUDY INTERVENTION AND PARTICIPANT DISCONTINUATION/WITHDRAWAL ....121
7.1. Discontinuation of Study Intervention ....121
7.2. Participant Discontinuation/Withdrawal From the Study ....123
7.2.1. Withdrawal of Consent/Assent ....124
7.3. Lost to Follow-up ....124
8. STUDY ASSESSMENTS AND PROCEDURES ....125
8.1. Efficacy Assessments ....125
8.1.1. Tumor Response Assessments ....125
8.1.1.1. BICR Evaluation of Imaging Data ....127
8.1.2. Patient-reported Outcomes ....129



090177e1a02cb071\Approved\Approved On: 14-Mar-2024 15:31 (GMT)

8.1.3. Survival Follow-up ....130
8.2. Safety Assessments ....130
8.2.1. Participant Demographics and Other Baseline Characteristics ....131
8.2.2. Disease Characteristics and Treatment History ....131
8.2.3. Physical Examinations ....131
8.2.4. Body Surface Area ....131
8.2.5. Dermatological Examinations ....132
8.2.6. Vital Signs ....132
8.2.7. Electrocardiograms ....132
8.2.8. Clinical Safety Laboratory Assessments ....133
8.2.9. Pregnancy Testing ....134
8.2.10. ECOG Performance Status ....134
8.3. Adverse Events and Serious Adverse Events ....135
8.3.1. Time Period and Frequency for Collecting AE and SAE Information ....135
8.3.1.1. Reporting SAEs to Pfizer Safety ....136
8.3.1.2. Recording Nonserious AEs and SAEs on the CRF ....136
8.3.2. Method of Detecting AEs and SAEs ....136
8.3.3. Follow-up of AEs and SAEs ....137
8.3.4. Regulatory Reporting Requirements for SAEs ....137
8.3.5. Exposure During Pregnancy or Breastfeeding, and Occupational Exposure ....137
8.3.5.1. Exposure During Pregnancy ....137
8.3.5.2. Exposure During Breastfeeding ....139
8.3.5.3. Occupational Exposure ....140
8.3.6. Cardiovascular and Death Events ....140
8.3.7. Disease-Related Events and/or Disease-Related Outcomes Not Qualifying as AEs or SAEs ....140
8.3.8. Adverse Events of Special Interest ....141
8.3.8.1. Lack of Efficacy ....141
8.3.9. Medical Device Deficiencies ....141
8.3.10. Medication Errors ....141
8.4. Treatment of Overdose ....142



090177e1a02cb071\Approved\Approved On: 14-Mar-2024 15:31 (GMT)

8.5. Pharmacokinetics ....142
8.5.1. Pharmacokinetics for Participants in Mainland China ....145
8.6. Pharmacodynamics....147
8.7. Genetics....147
8.7.1. Specified Genetics ....147
8.7.2. *BRAF* Testing....147
8.7.3. Molecular Prescreening ....149
8.7.4. Screening ....150
8.7.5. Companion Diagnostic for Eligibility ....150
8.7.6. Companion Diagnostic Assay Development....150
8.7.7. Banked Biospecimens for Genetics....151
8.8. Biomarkers ....151
8.8.1. CRP and CEA assessments....152
8.8.2. Tumor Tissue Assessments....152
8.8.3. Circulating Free DNA (cfDNA) ....153
8.8.4. Specified Gene Expression (RNA) Research ....153
8.8.5. Specified Protein Research....153
8.8.6. Specified Metabolomic Research ....154
8.9. Immunogenicity Assessments....154
8.10. Health Economics ....154
9. STATISTICAL CONSIDERATIONS ....154
9.1. Estimands and Statistical Hypotheses....154
9.1.1. Estimands....154
9.1.2. Statistical Hypotheses....157
9.2. Sample Size Determination....158
9.3. Analysis Sets ....161
9.4. Statistical Analyses ....162
9.4.1. General Considerations....162
9.4.1.1. Statistical Testing Strategy....162
9.4.1.2. Pooling of Centers....163
9.4.1.3. Stratification Factors ....163



090177e1a02cb071\Approved\Approved On: 14-Mar-2024 15:31 (GMT)

9.4.1.4. Definition of Baseline ....164
9.4.1.5. Definition of On-treatment Period ....164
9.4.2. Primary Endpoints ....164
9.4.3. Secondary Endpoints ....166
9.4.3.1. PFS Arm A versus Arm C, Arm A versus Arm B, and Cohort 3 ....166
9.4.3.2. OS ....167
9.4.3.3. Objective Response Rate ....168
9.4.3.4. Duration of Response ....168
9.4.3.5. Time to Response ....168
9.4.3.6. PFS (SLI) ....169
9.4.3.7. PFS2 ....169
9.4.3.8. Patient Reported Outcomes ....169
9.4.3.9. Pharmacokinetic Analyses ....169
9.4.3.10. Exposure-Response of Encorafenib ....170
9.4.3.11. Biomarker Analyses ....170
9.4.4. Tertiary/Exploratory Endpoints ....170
9.4.5. Safety Analyses ....171
9.4.5.1. Adverse Events ....171
9.4.5.2. Clinical Laboratory Results ....171
9.4.5.3. Electrocardiogram Analyses ....171
9.4.6. Other Analyses ....171
9.4.6.1. Subgroup Analyses ....171
9.5. Interim Analyses ....172
9.5.1. Interim Efficacy Analysis for OS (Phase 3) ....172
9.6. Data Monitoring Committee or Other Independent Oversight Committee ....172
10. SUPPORTING DOCUMENTATION AND OPERATIONAL CONSIDERATIONS ....173
10.1. Appendix 1: Regulatory, Ethical, and Study Oversight Considerations ....173
10.1.1. Regulatory and Ethical Considerations ....173
10.1.1.1. Reporting of Safety Issues and Serious Breaches of the Protocol or ICH GCP ....174



090177e1a02cb071\Approved\Approved On: 14-Mar-2024 15:31 (GMT)

10.1.2. Financial Disclosure ....174
10.1.3. Informed Consent/Assent Process ....174
10.1.3.1. Adult Participants ....174
10.1.3.2. Pediatric Participants ....175
10.1.4. Data Protection ....177
10.1.5. Dissemination of Clinical Study Data ....178
10.1.6. Data Quality Assurance ....179
10.1.7. Source Documents ....180
10.1.8. Study and Site Start and Closure ....180
10.1.9. Publication Policy ....181
10.1.10. Sponsor's Qualified Medical Personnel ....182
10.2. Appendix 2: Clinical Laboratory Tests ....183
10.3. Appendix 3: Adverse Events: Definitions and Procedures for Recording, Evaluating, Follow-up, and Reporting ....185
10.3.1. Definition of AE ....185
10.3.2. Definition of SAE ....186
10.3.3. Recording/Reporting and Follow-up of AEs and/or SAEs....188
10.3.4. Reporting of SAEs ....191
10.4. Appendix 4: Contraceptive Guidance ....192
10.4.1. Male Participant Reproductive Inclusion Criteria ....192
10.4.2. Female Participant Reproductive Inclusion Criteria....192
10.4.3. Woman of Childbearing Potential ....193
10.4.4. Contraception Methods....194
10.5. Appendix 5: Genetics ....196
10.6. Appendix 6: Liver Safety: Suggested Actions and Follow-up Assessments ....197
10.7. Appendix 7: ECG Findings of Potential Clinical Concern ....199
10.8. Appendix 8: Country-Specific Requirements ....201
10.8.1. France Contract Unique ....201
10.8.2. Mainland China Specific Procedures....201
10.9. Appendix 9: RECIST 1.1 ....202
10.10. Appendix 10: Patient Global Impression of Severity and Patient Global Impression of Change....207



090177e1a02cb071\Approved\Approved On: 14-Mar-2024 15:31 (GMT)

10.11. Appendix 11: Abbreviations ....................................................................208
10.12. Appendix 12: Alternative Measures During Public Emergencies.....................214
10.12.1. Eligibility ............................................................................................214
10.12.2. Telehealth Visits ...................................................................................214
10.12.3. Alternative Facilities for Safety Assessments .....................................214
10.12.3.1. Laboratory Testing .........................................................214
10.12.3.2. Imaging...........................................................................215
10.12.3.3. Electrocardiograms.........................................................215
10.12.4. Study Intervention ...............................................................................215
10.12.5. Home Health Visits...............................................................................216
10.12.6. Adverse Events and Serious Adverse Events .....................................216
10.12.7. Efficacy Assessments ..........................................................................216
10.12.8. Independent Oversight Committees ...................................................216
10.13. Appendix 13: Protocol Amendment History...................................................217
REFERENCES .............................................................................................................229

## LIST OF TABLES

Table 1. Schedule of Activities (Molecular Prescreening and Screening) .............25
Table 2. Schedule of Activities (Safety Lead-in) .................................................28
Table 3. Schedule of Activities (Phase 3 Arm A and Arm B; Cohort 3 Arm D ) ..........37
Table 4. Schedule of Activities (Phase 3 Control Arm [Arm C]: mFOLFOX6 or FOLFOXIRI ± Bevacizumab; Cohort 3 Arm E: FOLFIRI ±Bevacizumab) .........47
Table 5. Schedule of Activities (Control Arm [Arm C]: CAPOX ± Bevacizumab) ........54
Table 6. Efficacy of Standard of Care First-Line Therapies in Patients with *BRAF*-mutant mCRC ........61
Table 7. Study ARRAY-818-302 (EC Arm and Control Arm): Efficacy Results..........63
Table 8. Study ARRAY-818-302 (EC Arm and Control Arm): Adverse Events Regardless of Causality Reported in ≥15% of EC Patients ..........63
Table 9. DLT Criteria .........................................................................91



090177e1a02cb071\Approved\Approved On: 14-Mar-2024 15:31 (GMT)

Table 10. Treatment Regimens (Safety Lead-in)....93
Table 11. Treatment Regimen (Phase 3, Arm A) ....93
Table 12. Treatment Regimens (Phase 3, Arm B)....94
Table 13. Treatment Regimens (Phase 3, Control Arm [Arm C])....95
Table 14. Treatment Regimens: Cohort 3 (Arm D)....96
Table 15. Treatment Regimens: Cohort 3 (Arm E) ....96
Table 16. Recommended Encorafenib Dose Reductions....107
Table 17. Recommended Encorafenib Dose Modifications ....107
Table 18. Recommended Cetuximab Dose Reductions....110
Table 19. Recommended Dose Modifications for Cetuximab related Adverse Events ....110
Table 20. Recommended Dose Reductions for Oxaliplatin, Irinotecan and 5-FU as Part of mFOLFOX6 (SLI participants, Phase 3 Arm B and Arm C [Control]), and FOLFIRI (in SLI and Cohort 3 Arm D and Arm E....112
Table 21. Recommended Dose Reductions for Oxaliplatin, Irinotecan and 5-FU as Part of FOLFOXIRI (Phase 3 Arm C [Control Arm])....113
Table 22. Recommended Dose Reductions for Oxaliplatin and Capecitabine as Part of CAPOX (Phase 3 Control Arm) ....113
Table 23. Recommended Oxaliplatin, Irinotecan and 5-FU Dose Modifications....113
Table 24. Recommended Capecitabine Dose Modifications....118
Table 25. Recommended Bevacizumab Dose Modifications....119
Table 26. Eastern Cooperative Oncology Group (ECOG) Performance Status Scale....135
Table 27. PK Sampling Schedule for Cohort 1 (EC + FOLFIRI) in SLI ....143
Table 28. PK Sampling Schedule for Cohort 2 (EC + mFOLFOX6) in SLI....144
Table 29. PK Sampling Schedule for Phase 3 Arm A and Arm B and Cohort 3 Arm D ....144
Table 30. PK Sampling Schedule for the First 16 Participants Enrolled in Phase 3 Arm A in Mainland China....146
Table 31. PK Sampling Schedule for Participants Enrolled in Phase 3 Arm A and Arm B, and Cohort 3 Arm D (Who Will Not Undergo Intensive PK Sampling) in Mainland China....147
Table 32. PFS Outcome and Event Dates – Primary Analysis....156



090177e1a02cb071\Approved\Approved On: 14-Mar-2024 15:31 (GMT)

Table 33. Characteristics of DLT Probability During the DLT Evaluation Period ....159
Table 34. Probability of Observing ≥1 Instance of Toxicity at 10% and 15% Incidence Rate ....159
Table 35. Protocol-Required Safety Laboratory Assessments ....183
Table 36. Objective Response Status at Each Assessment for Participants With Measurable Disease at Baseline....205

## LIST OF FIGURES

Figure 1. *BRAF* Testing....149

090177e1a02cb071\Approved\Approved On: 14-Mar-2024 15:31 (GMT)

# 1. PROTOCOL SUMMARY

## 1.1. Synopsis

**Protocol Title:** An Open-label, Multicenter, Randomized Phase 3 Study of First-line Encorafenib Plus Cetuximab with or without Chemotherapy versus Standard-of-Care Therapy with a Safety Lead-in of Encorafenib and Cetuximab Plus Chemotherapy in Participants with Metastatic *BRAF* V600E-Mutant Colorectal Cancer

**Brief Title:** The BREAKWATER Study (*BRAF* V600E-mutant colorectal cancer study evaluating EncorAfenib taKen With cetuximAb plus or minus chemoThERapy)

**Regulatory Agency Identification Number(s):**

| | |
|---|---|
| **US IND Number:** | 115298 |
| **EudraCT / EU CT Number:** | 2020-001288-99 / 2023-509405-77-00 |
| **ClinicalTrials.gov ID:** | NCT04607421 |
| **Pediatric Investigational Plan Number:** | NA |
| **Protocol Number:** | C4221015 |
| **Phase:** | 3 |

### Rationale

The purpose of the study is to evaluate whether encorafenib plus cetuximab (EC), alone or in combination with chemotherapy, can improve clinical outcomes relative to current standard-of-care (SOC) chemotherapy in participants with previously untreated *BRAF* V600E-mutant metastatic colorectal cancer (mCRC). Since encorafenib has not previously been combined with chemotherapy, the tolerability and pharmacokinetics (PK) of EC in combination with modified fluorouracil/leucovorin/oxaliplatin (mFOLFOX6) and in combination with fluorouracil/leucovorin/irinotecan (FOLFIRI) will be evaluated in separate cohorts in the Safety Lead-in (SLI) portion of the trial in order to identify which chemotherapy combination is to be used in the Phase 3 portion of the study.

After reviewing the totality of the SLI data, the external Data Monitoring Committee (E-DMC) and Steering Committee (SC) determined that there were no new safety signals and recommended that the study can proceed as planned. As a result of the SLI data review, Arm B will consist of EC in combination with mFOLFOX6. In addition, Phase 3 treatment Control Arm (Arm C) will consist of Investigator's choice of mFOLFOX6 ± bevacizumab, fluorouracil/leucovorin/oxaliplatin/irinotecan (FOLFOXIRI) ± bevacizumab or capecitabine/oxaliplatin (CAPOX) ± bevacizumab.

The SLI data continues to show a tolerable safety profile and encouraging antitumor activity of EC + mFOLFOX6 and EC + FOLFIRI in both first-line and second-line participants (Tabernero J et al, Ann Oncol. 2022;33:S808-69). Based on this, an additional primary



090177e1a02cb071\Approved\Approved On: 14-Mar-2024 15:31 (GMT)

endpoint of objective response rate (ORR) (Arm B versus the Control Arm) was included and a cohort with 2 randomized arms was added to the study after the approval of Protocol Amendment 5: EC in combination with FOLFIRI (Arm D) and FOLFIRI with or without bevacizumab (Arm E) in first-line participants (Cohort 3).

In addition, enrollment in Arm A (EC) was discontinued after the approval of Protocol Amendment 5 because 1) antitumor activity, safety and tolerability observed with EC + mFOLFOX6 and EC + FOLFIRI in the SLI portion was encouraging (Tabernero J et al, Ann Oncol. 2022;33:S808-69); 2) the probability to demonstrate superiority for EC versus SOC chemotherapy is relatively low given the data from the ANCHOR-CRC study (NCT03693170), which showed comparable efficacy results of EC + binimetinib in comparison to historical chemotherapy (Van Cutsem E et al. Ann Oncol. 2021;32:S222); and 3) in general, *BRAF* V600E-mutant mCRC patients require a first-line regimen to control the aggressive tumor growth, supporting investigating EC + mFOLFOX6 or EC + FOLFIRI as potentially better treatment options in this patient population.

## Objectives, Estimands, and Endpoints

| Safety Lead-in | |
|---|---|
| **Objectives** | **Endpoints** |
| **Primary** | |
| • To determine the safety and tolerability of EC + mFOLFOX6 and EC + FOLFIRI | • Incidence of dose-limiting toxicities (DLTs) |
| **Secondary** | |
| • To assess the overall safety and tolerability of EC + mFOLFOX6 and EC + FOLFIRI | • Incidence and severity of adverse events (AEs) graded according to the National Cancer Institute (NCI) Common Terminology Criteria for Adverse Events (CTCAE) v4.03 and changes in clinical laboratory parameters, vital signs and electrocardiograms (ECGs)<br>• Incidence of dose interruptions, dose modifications and discontinuations due to AEs |
| • To estimate the efficacy of EC + mFOLFOX6 and EC + FOLFIRI | • ORR by Investigator, defined as the proportion of participants who have achieved a confirmed best overall response (BOR) (complete response [CR] or partial response [PR]) per Response Evaluation Criteria in Solid Tumors (RECIST) v1.1<br>• Duration of response (DOR) by Investigator, defined as the time from the date of first radiographic evidence of response (CR or PR) to the earliest documented disease progression per RECIST v1.1, or death due to any cause<br>• Progression-free survival (PFS) by Investigator, defined as the time from first dose to the earliest documented disease progression per RECIST v1.1, or death due to any cause<br>• Time to response (TTR) by Investigator, defined as the time from first dose to first radiographic |



090177e1a02cb071\Approved\Approved On: 14-Mar-2024 15:31 (GMT)

| | |
|---|---|
| | evidence of response (CR or PR) per RECIST v1.1 |
| • To compare the efficacy of EC + mFOLFOX6 and EC + FOLFIRI | • Overall survival (OS) defined as the time from first dose to death due to any cause |
| • To characterize the PK of encorafenib, irinotecan, oxaliplatin and relevant metabolites | • PK parameters of encorafenib, irinotecan, oxaliplatin and relevant metabolites |
| • To assess drug-drug interaction of encorafenib with irinotecan or oxaliplatin | • Changes in exposures of irinotecan and its metabolite (SN-38) on Cycle 1 Day 15 compared to Cycle 1 Day 1 in Cohort 1 (EC + FOLFIRI)<br>• Changes in exposures of oxaliplatin on Cycle 1 Day 15 compared to Cycle 1 Day 1 in Cohort 2 (EC + mFOLFOX6) |
| **Phase 3** | |
| **Objectives** | **Endpoints** |
| **Primary** | |
| • To compare the efficacy of EC + mFOLFOX6 (Arm B) vs SOC (Control Arm [Arm C]) as measured by PFS and by ORR | • PFS by blinded independent central review (BICR), defined as the time from the date of randomization to the earliest documented disease progression per RECIST v1.1, or death due to any cause<br>• ORR by BICR |
| **Key Secondary** | |
| • To further compare the efficacy of Arm B vs the Control Arm as measured by OS | • OS, defined as the time from the date of randomization to death due to any cause |
| **Secondary (Descriptive Statistics Only)** | |
| • To further evaluate the efficacy of Arm B vs the Control Arm as measured by ORR, DOR, PFS, progression after next line of therapy (PFS2) and TTR<br>• To evaluate the efficacy of EC (Arm A) vs the Control Arm as measured by ORR, DOR, PFS, PFS2, TTR, and OS<br>• To evaluate the efficacy of Arm A vs Arm B as measured by OS, PFS, PFS2, ORR, DOR and TTR | • ORR by Investigator<br>• ORR by BICR (Arm A vs Control Arm, Arm A vs Arm B)<br>• DOR by BICR and by Investigator<br>• PFS by BICR (Arm A vs Control Arm, Arm A vs Arm B)<br>• OS (Arm A vs Control Arm, Arm A vs Arm B)<br>• PFS by Investigator<br>• TTR (by BICR and by Investigator), defined as the time from the date of randomization to first radiographic evidence of response (CR or PR) per RECIST v1.1<br>• PFS2, defined as the time from the date of randomization to the date of discontinuation of next-line treatment after first objective PD by investigator assessment, the second objective disease progression, or death from any cause, whichever occurs first |
| • To determine the safety and tolerability of EC<br>• To determine the safety and tolerability of EC + mFOLFOX6 | • Incidence and severity of AEs graded according to the NCI CTCAE v4.03 and changes in clinical laboratory parameters, vital signs, and ECGs |



090177e1a02cb071\Approved\Approved On: 14-Mar-2024 15:31 (GMT)

| | |
|---|---|
| • To evaluate quality of life and health states, captured by patient-reported outcome (PRO) measures | • PRO scores as measured by the European Organisation for Research and Treatment of Cancer Quality of Life Questionnaire for Cancer Patients – 30 Item Core Questionnaire (EORTC QLQ-C30), EuroQol-5D-5L (EQ-5D-5L), and anchoring instruments Patient Global Impression of Severity (PGIS) and Patient Global Impression of Change (PGIC). |
| • To evaluate trough concentrations of encorafenib and its metabolite LHY746 in Arm A and Arm B | • Trough plasma concentrations of encorafenib and the metabolite LHY746 in Arm A and Arm B |
| • To characterize the PK of encorafenib and its metabolite LHY746 in participants randomized in mainland China (Arm A and Arm B) | • PK parameters of encorafenib and its metabolite LHY746 |
| • To confirm the microsatellite instability (MSI) status in tumor tissue | • Summarize MSI-status as determined by retrospective central testing of baseline tumor tissue |
| • To determine the correlation between circulating tumor DNA (ctDNA) levels, *BRAF* V600 alterations, and clinical outcome | • ctDNA levels and *BRAF* V600 variant allele fraction (VAF) from ctDNA analysis of plasma samples collected at baseline and on treatment |
| **Cohort 3** | |
| **Objectives** | **Endpoints** |
| **Primary** | |
| • To compare the efficacy of EC + FOLFIRI (Arm D) vs FOLFIRI with or without bevacizumab (Control Arm [Arm E]) as measured by ORR | • ORR by BICR |
| **Key Secondary** | |
| • To further compare the efficacy of Arm D vs Arm E as measured by PFS | • PFS by BICR, defined as the time from the date of randomization to the earliest documented disease progression per RECIST v1.1, or death due to any cause |
| **Secondary (Descriptive Statistics Only)** | |
| • To further evaluate the efficacy of Arm D vs Arm E as measured by ORR, DOR, PFS, TTR and OS | • ORR by Investigator<br>• DOR by BICR and by Investigator, defined as the time from the date of first radiographic evidence of response (CR or PR) to the earliest documented disease progression per RECIST v1.1, or death due to any cause<br>• PFS by Investigator, defined as the time from the date of randomization to the earliest documented disease progression per RECIST v1.1, or death due to any cause<br>• OS, defined as the time from the date of randomization to death due to any cause<br>• TTR (by BICR and by Investigator), defined as the time from the date of randomization to first |



090177e1a02cb071\Approved\Approved On: 14-Mar-2024 15:31 (GMT)

| | |
|---|---|
| | radiographic evidence of response (CR or PR) per RECIST v1.1 |
| • To determine the safety and tolerability of EC + FOLFIRI | • Incidence and severity of AEs graded according to the NCI CTCAE v4.03 and changes in clinical laboratory parameters, vital signs, and ECGs |
| • To evaluate quality of life and health states, captured by PRO measures | • PRO scores as measured by the EORTC QLQ-C30, EQ-5D-5L, and anchoring instruments PGIS and PGIC |
| • To evaluate trough concentrations of encorafenib and its metabolite LHY746 in Cohort 3 Arm D | • Trough plasma concentrations of encorafenib and the metabolite LHY746 in Cohort 3 Arm D |
| • To confirm the MSI status in tumor tissue | • Summarize MSI-status as determined by retrospective central testing of baseline tumor tissue |
| • To determine the correlation between ctDNA levels, *BRAF* V600 alterations, and clinical outcome | • ctDNA levels and *BRAF* V600 VAF from ctDNA analysis of plasma samples collected at baseline and on treatment |

**Estimands**

The primary estimand in the SLI is the DLT rate estimated based on data from DLT-evaluable participants during the DLT-evaluation period, which is the first 28 days after the first dose of study intervention in the SLI. The number and proportion of participants experiencing DLTs during the DLT-evaluation period will be summarized for Cohort 1 (EC + FOLFIRI) and Cohort 2 (EC + mFOLFOX6), separately.

There are 2 primary estimands in the Phase 3 portion of the study:

- The treatment effect in PFS by BICR, as measured by the hazard ratio. PFS is defined as the time from the date of randomization to the earliest documented disease progression per RECIST v1.1, or death due to any cause. The hypothetical strategy will be applied for the intercurrent events. PFS will be compared between Arm B and the Control Arm (Arm C) using a 1-sided stratified log-rank test.

- The treatment effect of Arm B on objective response (OR) based on BICR assessment per RECIST v1.1 compared to the Control Arm from randomization to the earliest of progression of disease, start of subsequent anticancer therapy, or death due to any cause. Both CR and PR must be confirmed by repeat assessments performed no less than 4 weeks after the criteria for response are first met. ORR comparison between the 2 treatment arms will be assessed using stratified Cochran-Mantel-Haenszel (CMH) test.

The primary estimand in Cohort 3 is the treatment effect of Arm D on OR based on BICR assessment per RECIST v1.1 compared to Arm E from randomization to the earliest of progression of disease, start of subsequent anticancer therapy, or death due to any cause. Both CR and PR must be confirmed by repeat assessments performed no less than 4 weeks after the criteria for response are first met. ORR comparison between the 2 treatment arms will be assessed using stratified CMH test.



090177e1a02cb071\Approved\Approved On: 14-Mar-2024 15:31 (GMT)

**Overall Design**

This is an open-label, multicenter, randomized Phase 3 study of EC with or without chemotherapy versus SOC chemotherapy in participants with previously untreated *BRAF* V600E-mutant mCRC. Prior to the Phase 3 portion, a SLI will be conducted at a limited number of sites to evaluate the safety/tolerability and PK of EC in combination with either FOLFIRI or mFOLFOX6 (Cohort 1 and Cohort 2, respectively). In addition to the Phase 3 portion, a Cohort 3 with 2 randomized arms will be included: EC in combination with FOLFIRI (Arm D) and FOLFIRI with or without bevacizumab (Arm E) in first-line participants. Cohort 3 will start after the enrollment of the Phase 3 portion is complete and enrollment will only be conducted in countries where authorized.

The primary objective in the Phase 3 portion of the study is to compare the efficacy, as measured by the primary endpoints of PFS by BICR and ORR by BICR, of Arm B versus the Control Arm. The study will be considered positive (ie, demonstrated evidence of effectiveness) if the hypothesis test of either primary endpoint is statistically significant.

The primary objective in the Cohort 3 portion of the study is to compare the efficacy, as measured by the primary endpoint of ORR by BICR of Arm D versus Arm E. The results of Cohort 3 will be considered positive (ie, demonstrated evidence of effectiveness) if the hypothesis test of primary endpoint is statistically significant.

**Number of Participants**

Approximately 60 participants will be enrolled in the SLI portion (up to 30 per regimen). The results of the SLI will inform which chemotherapy regimen is used in Arm B of the Phase 3 portion of the study. In the Phase 3 portion, approximately 620 participants will be randomized overall, initially at a ratio of 1:1:1 to receive EC (Arm A), EC + chemotherapy (Arm B) or SOC chemotherapy (Control Arm), and then 1:1 to receive EC + chemotherapy (Arm B) or SOC chemotherapy (Control Arm) after the approval of Protocol Amendment 5, with a total of approximately 150 participants for Arm A and 235 participants each for Arm B and the Control Arm (Arm C). Randomization will be stratified based on Eastern Cooperative Oncology Group performance status (ECOG PS) (0 versus 1) and region (United States [US]/Canada versus Europe versus Rest of World). In Cohort 3, 135 participants will be randomized overall, at a ratio of 2:1 to receive EC + FOLFIRI (Arm D, n=90) or FOLFIRI with or without bevacizumab (Arm E, n=45). Randomization will be stratified based on ECOG PS (0 versus 1).

**Study Population**

The study population is participants with *BRAF* V600E-mutant mCRC who have received ≤1 (SLI) or no (Phase 3 and Cohort 3) prior systemic regimen(s) for metastatic disease.

Key inclusion and exclusion criteria are listed below:



090177e1a02cb071\Approved\Approved On: 14-Mar-2024 15:31 (GMT)

090177e1a02cb071\Approved\Approved On: 14-Mar-2024 15:31 (GMT)

Inclusion Criteria

Participants must meet the following key inclusion criteria to be eligible for enrollment into the study:

- Male and female participants age ≥18 years (SLI) or ≥16 years (Phase 3 and Cohort 3, where permitted locally).
- Histologically or cytologically confirmed Stage IV colorectal adenocarcinoma with presence of a *BRAF* V600E mutation in tumor tissue or blood.
- Have received ≤1 (SLI) or no (Phase 3 and Cohort 3) prior systemic regimen(s) for metastatic disease (note: prior adjuvant or neoadjuvant therapy is considered to be metastatic treatment if relapse/metastasis < 6 month from end of adjuvant/neoadjuvant treatment).
- Measurable disease (Phase 3 and Cohort 3) and measurable or non-measurable but evaluable disease per RECIST, v1.1 (SLI), as assessed by Investigator and evidenced by available baseline scans.
- ECOG PS of 0 or 1.
- Adequate bone marrow, hepatic, and renal function.

Exclusion Criteria

Participants with any of the following characteristics/conditions will be excluded:

- Locally confirmed deficient mismatch repair (dMMR) or microsatellite instability-high (MSI-H) colorectal carcinoma or unknown MSI/mismatch repair (MMR) status unless participant is unable to receive immune checkpoint inhibitors due to a pre-existing medical condition.
- Evidence of active and uncontrolled bacterial or viral infection within 2 weeks prior to start of study intervention, with certain noted exceptions for chronic infection with human immunodeficiency virus (HIV), hepatitis B or hepatitis C.
- Symptomatic brain metastases.

**Intervention Groups and Duration**

**SLI:** Participants in the SLI will receive either encorafenib (300 mg once daily [QD]) + cetuximab (500 mg/m$^2$ every 2 weeks [Q2W]) + mFOLFOX6 or encorafenib (300 mg QD) + cetuximab (500 mg/m$^2$ Q2W) + FOLFIRI.

**Phase 3:** Participants randomized to Arm A of Phase 3 will receive encorafenib (300 mg QD) + cetuximab (500 mg/m$^2$ Q2W). Based on the results of the SLI, participants randomized to Arm B of Phase 3 will receive encorafenib (300 mg QD) + cetuximab (500 mg/m$^2$ Q2W) + mFOLFOX6. Since mFOLFOX6 is used in Arm B, the Control Arm will consist of Investigator's choice of mFOLFOX6 ± bevacizumab, FOLFOXIRI ± bevacizumab or CAPOX ± bevacizumab.

**Cohort 3:** Participants in Cohort 3 will be randomized 2:1 to receive either encorafenib (300 mg QD) + cetuximab (500 mg/m$^2$ Q2W) + FOLFIRI (Arm D) or FOLFIRI with or without bevacizumab (Arm E).

Participants will receive study intervention until disease progression (confirmed by BICR in Phase 3 and Cohort 3), withdrawal of consent/assent, lost to follow-up, death or unacceptable toxicity. After discontinuation of study intervention, participants will be followed for survival, until withdrawal of consent/assent, the participant is lost to follow-up, death, or the final OS analyses.

**Data Monitoring Committee or Other Independent Oversight Committee**

This study will use an E-DMC to review cumulative safety data during the study conduct as well as to review the efficacy at the interim analyses according to the charter. The E-DMC is independent of the study team and includes only external members.

All imaging data acquired in the study for efficacy purposes (eg, computed tomography [CT]/magnetic resonance imaging [MRI] scans) will be transmitted to an imaging vendor for BICR review. These images will be read by readers who are blinded to treatment assignment and to other clinical data as specified in the BICR Charter.

**Ethical Considerations**

Potential risks of synergistic toxicities of study intervention include QT prolongation, peripheral neuropathy, and gastrointestinal toxicities. Investigators should refer to the approved local label of the products used in this trial (capecitabine, cetuximab, fluorouracil [5-FU], irinotecan, leucovorin, oxaliplatin, and bevacizumab) for the management of participants, especially concerning contraindications, duration of contraception, special warnings and precautions, posology adaptation in case of toxicity, monitoring, as well as medications that are contraindicated or that must be used with caution.

The EC regimen is approved in several countries for the treatment of patients with *BRAF* V600E-mutant mCRC that has progressed after 1 or 2 prior regimens. Based on the activity observed in previously treated patients, there is the potential for the EC regimen to be efficacious in the first-line metastatic setting in patients with *BRAF* V600E-mutant mCRC. Furthermore, based on its demonstrated activity in patients with *BRAF* V600E-mutant mCRC, there is the potential for additional benefits to patients when EC is added to chemotherapy regimens that are the current SOC.

Considering the measures taken to minimize risk to study participants, the potential risks identified in association with the EC regimen with or without mFOLFOX6 or FOLFIRI, as well as the potential risks associated with study procedures and laboratory tests (eg, CT or MRI scans, *BRAF* testing), are justified by the anticipated benefits that may be afforded to participants with *BRAF* V600E-mutant mCRC.

090177e1a02cb071\Approved\Approved On: 14-Mar-2024 15:31 (GMT)

090177e1a02cb071\Approved\Approved On: 14-Mar-2024 15:31 (GMT)

## 1.2. Schema

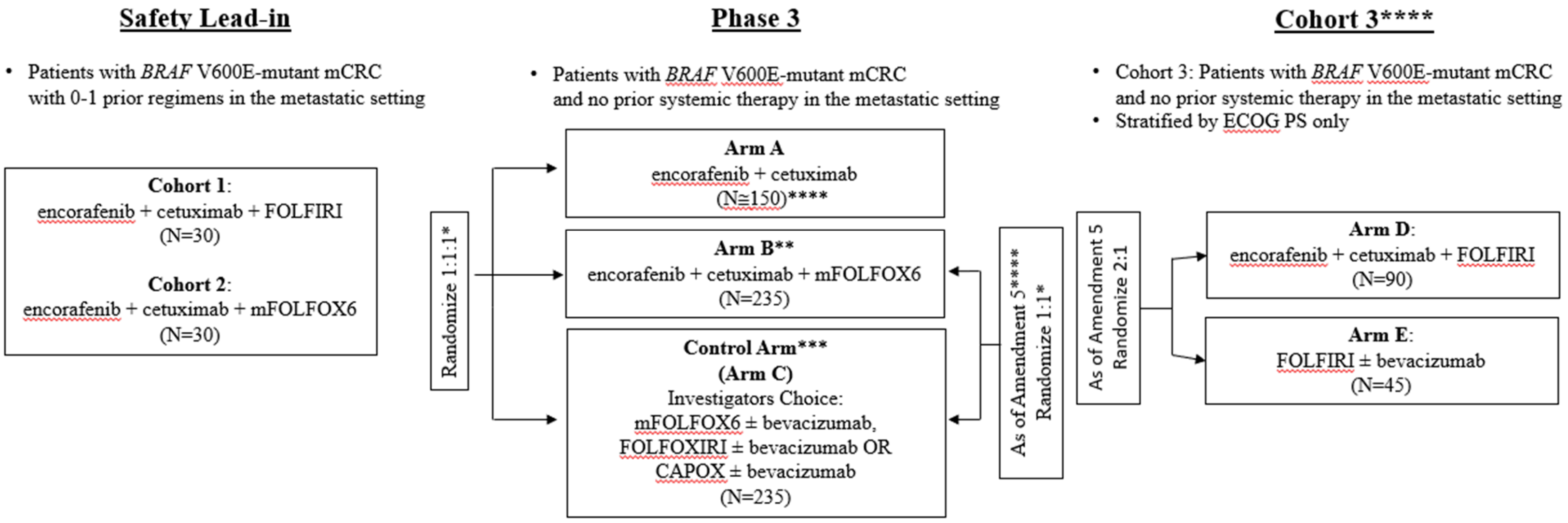


* Stratified by: ECOG PS (0 vs 1) and Region (US/Canada vs Europe vs Rest of World).
** mFOLFOX6 will be used in Arm B based on results of the SLI portion of the study.
*** Since mFOLFOX6 is used in Arm B, the Control Arm will consist of investigator's choice of mFOLFOX6 ± bevacizumab, FOLFOXIRI ± bevacizumab or CAPOX ± bevacizumab.
**** Upon Protocol Amendment 5 approval by site EC/IRB: randomization to Arm A is discontinued; Cohort 3 to begin after Phase 3 enrollment is complete.

## 1.3. Schedule of Activities

The SoA table provides an overview of the protocol visits and procedures. Refer to the STUDY ASSESSMENTS AND PROCEDURES section of the protocol for detailed information on each procedure and assessment required for compliance with the protocol.

The investigator may schedule visits (unplanned visits) in addition to those listed in the SoA table, in order to conduct evaluations or assessments required to protect the well-being of the participant.

090177e1a02cb071\Approved\Approved On: 14-Mar-2024 15:31 (GMT)

**Table 1. Schedule of Activities (Molecular Prescreening and Screening)**

| **Evaluation/Window**<br>Abbreviations used in this table may be found in Appendix 11. | **Prescreening**<br>**Any Time Before Screening** | **Screening**<br>**Day -28 to -1** | **Randomization** | **Notes/Protocol Section** |
|---|---|---|---|---|
| **Administrative Procedures** | | | | |
| Informed consent/assent | X | | | See Section 10.1.3 |
| Molecular Prescreening inclusion/exclusion criteria | X | | | See Section 5.1.1 and Section 5.2.1 |
| Contact IRT | | X | X | All participants will be registered for the study using the IRT after informed consent/assent is obtained. Phase 3 and Cohort 3 participants will be randomized via IRT upon completion of Screening.<br>Phase 3 and Cohort 3 participants must receive their first dose of study intervention within 5 days of randomization.<br>See Section 6.3.1 |
| Demography | X | | | See Section 8.2.1 |
| Screening inclusion/exclusion criteria | | X | | See Section 5.1.2 and Section 5.2.2 |
| Medical history | | X | | See Section 8.2.1 |
| Prior antineoplastic therapy/concomitant medication | | X | | See Section 6.5 |
| ECOG PS | | X | | See Section 8.2.10 |
| Verification of eligibility criteria | | X | | See Section 5 |

090177e1a02cb071\Approved\Approved On: 14-Mar-2024 15:31 (GMT)

**Table 1. Schedule of Activities (Molecular Prescreening and Screening)**

| **Evaluation/Window**<br>Abbreviations used in this table may be found in Appendix 11. | **Prescreening**<br>**Any Time Before Screening** | **Screening**<br>**Day -28 to -1** | **Randomization** | **Notes/Protocol Section** |
|---|---|---|---|---|
| **Clinical Procedures/Assessments** | | | | |
| Tumor tissue sample for *BRAF* V600E mutation testing | X | | | Central laboratory test for *BRAF* V600E mutation and other molecular markers.<br>See Section 8.7.2 and Section 8.8.2 |
| Tumor radiographic assessment (CT, MRI) | | X | | Must be performed prior to enrollment for participants in the SLI and prior to randomization for participants in the Phase 3 portion and Cohort 3. May be performed up to Day -35.<br>See Section 8.1.1 |
| Physical exam | | X | | See Section 8.2.3 |
| Height/Weight | | X | | See Section 8.2.3 |
| Vital signs | | X | | See Section 8.2.6 |
| Triplicate 12-lead ECG | | X | | See Section 8.2.7 |
| Adverse event assessment | | X | | See Section 8.3 and Appendix 3 |
| Dermatologic exam | | X | | See Section 8.2.5 |
| **Laboratory Assessments** | | | | |
| Hematology | | X | | See Section 8.2.8 and Table 35 |
| Chemistry | | X | | See Section 8.2.8 and Table 35 |
| Coagulation | | X | | See Section 8.2.8 and Table 35 |
| Full Urinalysis | | X | | See Section 8.2.8 and Table 35 |
| HBV and HCV serology;<br>HIV where mandated | | X | | See Section 5.2.2 and Table 35 |
| Serum pregnancy test | | X | | See Section 8.2.9 and Table 35 |

090177e1a02cb071\Approved\Approved On: 14-Mar-2024 15:31 (GMT)

**Table 1. Schedule of Activities (Molecular Prescreening and Screening)**

| **Evaluation/Window**<br>Abbreviations used in this table may be found in Appendix 11. | **Prescreening**<br>**Any Time Before Screening** | **Screening**<br>**Day -28 to -1** | **Randomization** | **Notes/Protocol Section** |
|---|---|---|---|---|
| Discussion of fertility preservation | | X | | See Section 5.3 |
| FSH | | X | | See Section 10.4.3 and Table 35 |
| Blood sample for CRP | | X | | See Section 8.8.1 |
| Blood sample for CEA | | X | | See Section 8.8.1 |
| Blood sample for *BRAF* CDx | | X | | Collect 20 mL of blood optimized for plasma preparation.<br>See Section 8.7.6 |

090177e1a02cb071\Approved\Approved On: 14-Mar-2024 15:31 (GMT)

**Table 2. Schedule of Activities (Safety Lead-in)**

| **Evaluation** | **Treatment Period (Each 28 Day Cycle)** | | | | **Post-Treatment Period** | | | **Notes/Protocol Section** |
|---|---|---|---|---|---|---|---|---|
| Abbreviations used in this table may be found in Appendix 11. | **Day 1** | **Day 3** | **Day 15** | **Day 17** | **End-of-Treatment (EOT) Visit** | **Follow-up Visit (28 days post last dose)** | **Long Term Follow-up (Every 3 months)** | End of Treatment is defined as when participant permanently discontinues study treatment.<br>With the exception of Cycle 1, all visits during the treatment period, EOT visit, and follow-up visit have a ± 3-day window.<br>Long Term Follow-up visits have a ± 7-day window for assessments. |
| **Administrative Procedures** | | | | | | | | |
| Verify Screening inclusion/exclusion criteria | Cycle 1 | | | | | | | Must occur prior to first dose.<br>See Section 5.1.2 and Section 5.2.2 |
| Verify demography; medical history from Screening to Day 1 | Cycle 1 | | | | | | | See Section 8.2.1 and Section 8.2.2 |
| Contraceptive check | X | | | | X | X | X | Performed monthly during long term follow-up; recommended to be followed for 9 months for females and 6 months for males following the last dose of study intervention.<br>See Section 5.3.1 |
| Concomitant medication/therapies | Assess continuously | | | | | | | See Section 6.5 |
| ECOG PS | Cycle 1 | | | | | | | Does not need to be repeated if performed within 72 hours prior to Cycle 1 Day 1 (ie, first day of dosing).<br>See Section 8.2.10 |

090177e1a02cb071\Approved\Approved On: 14-Mar-2024 15:31 (GMT)

### Table 2. Schedule of Activities (Safety Lead-in)

| **Evaluation**<br>Abbreviations used in this table may be found in Appendix 11. | **Treatment Period (Each 28 Day Cycle)** | | | | **Post-Treatment Period** | | | **Notes/Protocol Section** |
|---|---|---|---|---|---|---|---|---|
| | **Day 1** | **Day 3** | **Day 15** | **Day 17** | **End-of-Treatment (EOT) Visit** | **Follow-up Visit**<br>**(28 days post last dose)** | **Long Term Follow-up**<br>**(Every 3 months)** | End of Treatment is defined as when participant permanently discontinues study treatment.<br>With the exception of Cycle 1, all visits during the treatment period, EOT visit, and follow-up visit have a ± 3-day window.<br>Long Term Follow-up visits have a ± 7-day window for assessments. |
| Document survival status | | | | | | X | X | After discontinuation of study intervention, participants will be followed approximately every 3 months until withdrawal of consent, the participant is lost to follow-up, death, or the final OS analysis.<br>See Section 7.1 |
| Document subsequent therapies and dates of progression | | | | | Assess continuously | | | See Section 7.1 |
| Dispense encorafenib (with diary) | X | | | | | | | Encorafenib dosing will not begin until Cycle 1 Day 3 for participants receiving FOLFIRI<br>See Section 6.1.3.1 |
| Cetuximab IV infusion | X | | X | | | | | See Section 6.1.3.2 |
| mFOLFOX6 or FOLFIRI | X | | X | | | | | See Section 6.1.3.2 |

090177e1a02cb071\Approved\Approved On: 14-Mar-2024 15:31 (GMT)

**Table 2. Schedule of Activities (Safety Lead-in)**

| **Evaluation** Abbreviations used in this table may be found in Appendix 11. | **Treatment Period (Each 28 Day Cycle)** | | | | **Post-Treatment Period** | | | **Notes/Protocol Section** |
|---|---|---|---|---|---|---|---|---|
| | **Day 1** | **Day 3** | **Day 15** | **Day 17** | **End-of-Treatment (EOT) Visit** | **Follow-up Visit (28 days post last dose)** | **Long Term Follow-up (Every 3 months)** | End of Treatment is defined as when participant permanently discontinues study treatment. With the exception of Cycle 1, all visits during the treatment period, EOT visit, and follow-up visit have a ± 3-day window. Long Term Follow-up visits have a ± 7-day window for assessments. |
| Assess study intervention compliance | X | | | | X | | | See Section 6.4 |
| **Clinical Procedures/Assessments** | | | | | | | | |
| Physical exam | X | | | | X | X | | Does not need to be repeated if performed within 72 hours prior to Cycle 1 Day 1 (ie, first day of dosing). Exam to include visual assessment. See Section 8.2.3 |
| Weight | X | | X | | | | | Does not need to be repeated if performed within 72 hours prior to Cycle 1 Day 1 (ie, first day of dosing). See Section 8.2.3 |
| BSA | X | | | | | | | Does not need to be repeated if performed within 72 hours prior to Cycle 1 Day 1 (ie, first day of dosing). See Section 8.2.4 |
| Vital signs | X | | X | | X | X | | See Section 8.2.6 |

090177e1a02cb071\Approved\Approved On: 14-Mar-2024 15:31 (GMT)

**Table 2. Schedule of Activities (Safety Lead-in)**

| **Evaluation** Abbreviations used in this table may be found in Appendix 11. | **Treatment Period (Each 28 Day Cycle)** | | | | **Post-Treatment Period** | | | **Notes/Protocol Section** |
|---|---|---|---|---|---|---|---|---|
| | **Day 1** | **Day 3** | **Day 15** | **Day 17** | **End-of-Treatment (EOT) Visit** | **Follow-up Visit (28 days post last dose)** | **Long Term Follow-up (Every 3 months)** | End of Treatment is defined as when participant permanently discontinues study treatment. With the exception of Cycle 1, all visits during the treatment period, EOT visit, and follow-up visit have a ± 3-day window. Long Term Follow-up visits have a ± 7-day window for assessments. |
| Triplicate 12-lead ECG | Cycle 1 | | | | | | | Predose only. See Section 8.2.7 |
| Single 12-lead ECG | Cycles 2+ | | | | X | X | | Predose only. Perform continuous ECGs if QTc abnormality. See Section 8.2.7 |
| Tumor radiographic assessment (CT, MRI) | The first post-baseline scan must be performed 42 to 49 days from the date of first dose then Q6W (± 7D) for the first 18 months after the date of first dose then Q8W (± 7D) thereafter or until confirmed PD, withdrawal of consent/assent, initiation of subsequent anticancer therapy, participant is lost to follow-up, death, or the final OS analysis. | | | | | | | See Section 8.1.1 |

090177e1a02cb071\Approved\Approved On: 14-Mar-2024 15:31 (GMT)

**Table 2. Schedule of Activities (Safety Lead-in)**

| **Evaluation** Abbreviations used in this table may be found in Appendix 11. | **Treatment Period (Each 28 Day Cycle)** | | | | **Post-Treatment Period** | | | **Notes/Protocol Section** |
|---|---|---|---|---|---|---|---|---|
| | **Day 1** | **Day 3** | **Day 15** | **Day 17** | **End-of-Treatment (EOT) Visit** | **Follow-up Visit (28 days post last dose)** | **Long Term Follow-up (Every 3 months)** | End of Treatment is defined as when participant permanently discontinues study treatment. With the exception of Cycle 1, all visits during the treatment period, EOT visit, and follow-up visit have a ± 3-day window. Long Term Follow-up visits have a ± 7-day window for assessments. |
| Dermatologic exam | X (Cycles 1, 3, 5…) | | | | X | X | X[a] | Performed every 8 weeks from Cycle 1 Day 1 (ie, on Day 1 of Cycles 3, 5, 7…). a. Following the 28-day follow-up, it is recommended participants be monitored with dermatological examinations for cutaneous secondary malignancies approximately every 2 months for up to 6 months after the last encorafenib dose or until initiation of another antineoplastic therapy. Clinically significant findings that are considered related to study intervention should be reported as SAEs. See Section 8.2.5 |
| Adverse event assessment | Assess continuously | | | | | | | See Section 8.3 and Appendix 3 |

090177e1a02cb071\Approved\Approved On: 14-Mar-2024 15:31 (GMT)

**Table 2. Schedule of Activities (Safety Lead-in)**

| **Evaluation** Abbreviations used in this table may be found in Appendix 11. | **Treatment Period (Each 28 Day Cycle)** | | | | **Post-Treatment Period** | | | **Notes/Protocol Section** |
|---|---|---|---|---|---|---|---|---|
| | **Day 1** | **Day 3** | **Day 15** | **Day 17** | **End-of-Treatment (EOT) Visit** | **Follow-up Visit (28 days post last dose)** | **Long Term Follow-up (Every 3 months)** | End of Treatment is defined as when participant permanently discontinues study treatment. With the exception of Cycle 1, all visits during the treatment period, EOT visit, and follow-up visit have a ± 3-day window. Long Term Follow-up visits have a ± 7-day window for assessments. |
| **Laboratory Assessments** | | | | | | | | |
| Hematology | X | | X | | X | X | | Does not need to be repeated if performed within 72 hours prior to Cycle 1 Day 1 (ie, first day of dosing). See Section 8.2.8 and Table 35 |
| Chemistry | X | | X | | X | X | | Does not need to be repeated if performed within 72 hours prior to Cycle 1 Day 1 (ie, first day of dosing). See Section 8.2.8 and Table 35 |
| Coagulation | X | | X | | X | X | | Does not need to be repeated if performed within 72 hours prior to Cycle 1 Day 1 (ie, first day of dosing). See Section 8.2.8 and Table 35 |
| Urinalysis | X | | | | X | X | | Does not need to be repeated if performed within 72 hours prior to Cycle 1 Day 1 (ie, first day of dosing). Full urinalysis on Cycle 1 Day 1, only urine protein thereafter. See Section 8.2.8 and Table 35 |

090177e1a02cb071\Approved\Approved On: 14-Mar-2024 15:31 (GMT)

**Table 2. Schedule of Activities (Safety Lead-in)**

| **Evaluation** Abbreviations used in this table may be found in Appendix 11. | **Treatment Period (Each 28 Day Cycle)** | | | | **Post-Treatment Period** | | | **Notes/Protocol Section** |
|---|---|---|---|---|---|---|---|---|
| | **Day 1** | **Day 3** | **Day 15** | **Day 17** | **End-of-Treatment (EOT) Visit** | **Follow-up Visit (28 days post last dose)** | **Long Term Follow-up (Every 3 months)** | End of Treatment is defined as when participant permanently discontinues study treatment. With the exception of Cycle 1, all visits during the treatment period, EOT visit, and follow-up visit have a ± 3-day window. Long Term Follow-up visits have a ± 7-day window for assessments. |
| Urine pregnancy test | X | | | | X | X | | Does not need to be repeated if performed within 72 hours prior to Cycle 1 Day 1 (ie, first day of dosing). See Section 8.2.9 and Table 35 |
| Blood sample for PK | Cycles 1-6 | Cycle 1 | Cycle 1 | Cycle 1 | | | | PK sampling must occur on the Day of dosing in the clinic (ie, no 3 day window). Days 1 and 15 of Cycle 1 of Cohort 1 (EC + FOLFIRI): predose, 0.75, 1.5, 2.5, 3.5, 5.5 and 7.5 h post-dosing Days 1 and 15 of Cycle 1 of Cohort 2 (EC + mFOLFOX6): predose, 1, 2, 3, 4, 6 and 8 h post-dosing All other timepoints: Predose only See Section 8.5 |
| Blood sample for CRP | X | | | | X | | | See Section 8.8.1 |
| Blood sample for CEA | X | | | | X | | | See Section 8.8.1 |

090177e1a02cb071\Approved\Approved On: 14-Mar-2024 15:31 (GMT)

## Table 2. Schedule of Activities (Safety Lead-in)

| **Evaluation** Abbreviations used in this table may be found in Appendix 11. | **Treatment Period (Each 28 Day Cycle)** | | | | **Post-Treatment Period** | | | **Notes/Protocol Section** |
|---|---|---|---|---|---|---|---|---|
| | **Day 1** | **Day 3** | **Day 15** | **Day 17** | **End-of-Treatment (EOT) Visit** | **Follow-up Visit (28 days post last dose)** | **Long Term Follow-up (Every 3 months)** | End of Treatment is defined as when participant permanently discontinues study treatment. With the exception of Cycle 1, all visits during the treatment period, EOT visit, and follow-up visit have a ± 3-day window. Long Term Follow-up visits have a ± 7-day window for assessments. |
| Blood sample for cfDNA | Cycles 1,7 | | Cycles 1, 2 | | X | | | Collect 20 mL of blood optimized for plasma preparation at indicated visits (all pre-dose). See Section 8.8.3 |
| Blood sample for protein biomarkers | Cycles 1, 2 | | Cycles 1, 2 | | X | | | Collect 10 mL of whole blood for processing into serum at indicated visits (all pre-dose). See Section 8.8.5 |
| Blood sample for germline comparator | Cycle 1 | | | | | | | Collect a 4 mL blood biospecimen prior to first dose of study intervention. See Section 8.8.2 |

090177e1a02cb071\Approved\Approved On: 14-Mar-2024 15:31 (GMT)

**Table 2. Schedule of Activities (Safety Lead-in)**

| **Evaluation**<br>Abbreviations used in this table may be found in Appendix 11. | **Treatment Period (Each 28 Day Cycle)** | | | | **Post-Treatment Period** | | | **Notes/Protocol Section** |
|---|---|---|---|---|---|---|---|---|
| | **Day 1** | **Day 3** | **Day 15** | **Day 17** | **End-of-Treatment (EOT) Visit** | **Follow-up Visit**<br>**(28 days post last dose)** | **Long Term Follow-up**<br>**(Every 3 months)** | End of Treatment is defined as when participant permanently discontinues study treatment.<br>With the exception of Cycle 1, all visits during the treatment period, EOT visit, and follow-up visit have a ± 3-day window.<br>Long Term Follow-up visits have a ± 7-day window for assessments. |
| Blood sample for banked biospecimen | Cycle 1 | | | | | | | Unless prohibited by local regulations or ethics committee decision, collect a blood sample (4 mL) optimized for genomic analysis before the first dose of study intervention.<br>If not collected on the designated collection day, collect at the next available time point when biospecimens are being collected in conjunction with a participant visit.<br>See Section 8.7.7 |
| FFPE Tumor Tissue | | | | | X<br>(optional) | | | See Section 8.8.2 and Section 8.8.4 |

090177e1a02cb071\Approved\Approved On: 14-Mar-2024 15:31 (GMT)

**Table 3. Schedule of Activities (Phase 3 Arm A and Arm B; Cohort 3 Arm D )**

| **Evaluation** Abbreviations used in this table may be found in Appendix 11. | **Treatment Period (Each 28 Day Cycle)** | | **Post-Treatment Period** | | | **Notes/Protocol Section** |
|---|---|---|---|---|---|---|
| | **Day 1** | **Day 15** | **End-of-Treatment (EOT) Visit** | **Follow-up Visit (28 days post last dose)** | **Long Term Follow-up (Every 3 months)** | End of Treatment is defined as when participant permanently discontinues all study treatment.<br>Cycle 1 Day 1 assessments, unless otherwise specified, do not need to be repeated if performed within 72 hours prior to initial treatment (ie, first day of dosing).<br><br>All remaining visits during the treatment period, EOT visit, and follow-up visit have a ± 3-day window.<br>Long Term-Follow-up visits have ±7-day window for assessments. |
| **Administrative Procedures** | | | | | | |
| Verify Screening inclusion/exclusion criteria | Cycle 1 | | | | | Must occur prior to first dose.<br>See Section 5.1.2 and Section 5.2.2 |
| Verify demography; medical history from Screening to Day 1 | Cycle 1 | | | | | See Section 8.2.1 and Section 8.2.2 |
| Contraceptive check | X | | X | X | X | Performed monthly during long term follow-up; recommended to be followed for 9 months for females and 6 months for males following the last dose of study intervention.<br>See Section 5.3.1 |

090177e1a02cb071\Approved\Approved On: 14-Mar-2024 15:31 (GMT)

**Table 3. Schedule of Activities (Phase 3 Arm A and Arm B; Cohort 3 Arm D )**

| **Evaluation** Abbreviations used in this table may be found in Appendix 11. | **Treatment Period (Each 28 Day Cycle)** | | **Post-Treatment Period** | | | **Notes/Protocol Section** |
|---|---|---|---|---|---|---|
| | **Day 1** | **Day 15** | **End-of-Treatment (EOT) Visit** | **Follow-up Visit (28 days post last dose)** | **Long Term Follow-up (Every 3 months)** | End of Treatment is defined as when participant permanently discontinues all study treatment.<br>Cycle 1 Day 1 assessments, unless otherwise specified, do not need to be repeated if performed within 72 hours prior to initial treatment (ie, first day of dosing).<br><br>All remaining visits during the treatment period, EOT visit, and follow-up visit have a ± 3-day window.<br>Long Term-Follow-up visits have ±7-day window for assessments. |
| Concomitant medication/therapies | Assess continuously | | | | | See Section 6.5 |
| PGIS, PGIC, EORTC QLQ-C30, and EQ-5D-5L | Q6W for the first 18 months after the date of randomization (then Q8W thereafter or until End of Treatment | | X | X | | While on treatment, PRO assessments should be done with the same frequency as the tumor radiographic assessments.<br>PGIC will not be performed at baseline.<br>See Section 8.1.2 |
| ECOG PS | Cycle 1 | | | | | See Section 8.2.10 |
| Document survival status | | | | X | X | After discontinuation of study intervention, participants will be followed approximately every 3 months, until withdrawal of consent/assent, the participant is lost to follow-up, death, or final OS analysis.<br>See Section 7.1 |

090177e1a02cb071\Approved\Approved On: 14-Mar-2024 15:31 (GMT)

**Table 3. Schedule of Activities (Phase 3 Arm A and Arm B; Cohort 3 Arm D )**

| **Evaluation** Abbreviations used in this table may be found in Appendix 11. | **Treatment Period (Each 28 Day Cycle)** | | **Post-Treatment Period** | | | **Notes/Protocol Section** |
|---|---|---|---|---|---|---|
| | **Day 1** | **Day 15** | **End-of-Treatment (EOT) Visit** | **Follow-up Visit (28 days post last dose)** | **Long Term Follow-up (Every 3 months)** | End of Treatment is defined as when participant permanently discontinues all study treatment.<br>Cycle 1 Day 1 assessments, unless otherwise specified, do not need to be repeated if performed within 72 hours prior to initial treatment (ie, first day of dosing).<br><br>All remaining visits during the treatment period, EOT visit, and follow-up visit have a ± 3-day window.<br>Long Term-Follow-up visits have ±7-day window for assessments. |
| Document subsequent therapies and dates of progression | | | Assess continuously | | | See Section 7.1 |
| Dispense encorafenib (with diary) | X | | | | | See Section 6.1.3.1 |
| Cetuximab IV infusion | X | X | | | | See Section 6.1.3.2 |
| For Arm B Only: mFOLFOX6 | X | X | | | | See Section 6.1.3.2 |
| For Cohort 3 Arm D Only: FOLFIRI | X | X | | | | See Section 6.1.3.2 |
| Assess study intervention compliance | X | | X | | | See Section 6.4 |

090177e1a02cb071\Approved\Approved On: 14-Mar-2024 15:31 (GMT)

**Table 3. Schedule of Activities (Phase 3 Arm A and Arm B; Cohort 3 Arm D )**

| **Evaluation** Abbreviations used in this table may be found in Appendix 11. | **Treatment Period (Each 28 Day Cycle)** | | **Post-Treatment Period** | | | **Notes/Protocol Section** |
|---|---|---|---|---|---|---|
| | **Day 1** | **Day 15** | **End-of-Treatment (EOT) Visit** | **Follow-up Visit (28 days post last dose)** | **Long Term Follow-up (Every 3 months)** | End of Treatment is defined as when participant permanently discontinues all study treatment. Cycle 1 Day 1 assessments, unless otherwise specified, do not need to be repeated if performed within 72 hours prior to initial treatment (ie, first day of dosing). All remaining visits during the treatment period, EOT visit, and follow-up visit have a ± 3-day window. Long Term-Follow-up visits have ±7-day window for assessments. |
| **Clinical Procedures/Assessments** | | | | | | |
| Physical exam | X | | X | X | | Exam to include visual assessment. See Section 8.2.3 |
| Weight | X | X | | | | See Section 8.2.3 |
| Height | X | | | | | Height to be measured in participants <18 years old. See Section 8.2.3 |
| BSA | X | | | | | See Section 8.2.4 |
| Vital signs | X | X | X | X | | See Section 8.2.6 |
| Triplicate 12-lead ECG | Cycle 1 | | | | | Must be conducted on Cycle 1 Day 1 (ie, no window). Predose only. See Section 8.2.7 |

090177e1a02cb071\Approved\Approved On: 14-Mar-2024 15:31 (GMT)

## Table 3. Schedule of Activities (Phase 3 Arm A and Arm B; Cohort 3 Arm D )

| **Evaluation** Abbreviations used in this table may be found in Appendix 11. | **Treatment Period (Each 28 Day Cycle)** | | **Post-Treatment Period** | | | **Notes/Protocol Section** |
|---|---|---|---|---|---|---|
| | **Day 1** | **Day 15** | **End-of-Treatment (EOT) Visit** | **Follow-up Visit (28 days post last dose)** | **Long Term Follow-up (Every 3 months)** | End of Treatment is defined as when participant permanently discontinues all study treatment.<br>Cycle 1 Day 1 assessments, unless otherwise specified, do not need to be repeated if performed within 72 hours prior to initial treatment (ie, first day of dosing).<br><br>All remaining visits during the treatment period, EOT visit, and follow-up visit have a ± 3-day window.<br>Long Term-Follow-up visits have ±7-day window for assessments. |
| Single 12-lead ECG | Cycles 2+ | | X | X | | Predose only. Perform continuous ECGs if QTc abnormality.<br>See Section 8.2.7 |
| Tumor radiographic assessment (CT, MRI) | The first post-baseline scan must be performed 42 to 49 days from the date of randomization then Q6W (± 7D) for the first 18 months after the date of randomization then Q8W (± 7D) thereafter or until BICR-confirmed PD (regardless of new anticancer therapy), withdrawal of consent/assent, participant is lost to follow-up, death, or final OS analysis. | | | | | See Section 8.1.1 |

090177e1a02cb071\Approved\Approved On: 14-Mar-2024 15:31 (GMT)

**Table 3. Schedule of Activities (Phase 3 Arm A and Arm B; Cohort 3 Arm D )**

| **Evaluation** Abbreviations used in this table may be found in Appendix 11. | **Treatment Period (Each 28 Day Cycle)** | | **Post-Treatment Period** | | | **Notes/Protocol Section** |
|---|---|---|---|---|---|---|
| | **Day 1** | **Day 15** | **End-of-Treatment (EOT) Visit** | **Follow-up Visit (28 days post last dose)** | **Long Term Follow-up (Every 3 months)** | End of Treatment is defined as when participant permanently discontinues all study treatment.<br>Cycle 1 Day 1 assessments, unless otherwise specified, do not need to be repeated if performed within 72 hours prior to initial treatment (ie, first day of dosing).<br><br>All remaining visits during the treatment period, EOT visit, and follow-up visit have a ± 3-day window.<br>Long Term-Follow-up visits have ±7-day window for assessments. |
| Dermatologic exam | X (Cycles 1, 3, 5…) | | X | X | | Performed every 8 weeks from Cycle 1 Day 1 (ie, on Day 1 of Cycles 3, 5, 7…).<br>Following the 28-day follow up, it is recommended participants be monitored with dermatological examinations for cutaneous secondary malignancies approximately every 2 months for up to 6 months after the last encorafenib dose or until initiation of another antineoplastic therapy. Clinically significant findings that are considered related to study intervention should be reported as SAEs.<br>See Section 8.2.5 |
| Adverse event assessment | Assess continuously | | | | | See Section 8.3 and Appendix 3 |

090177e1a02cb071\Approved\Approved On: 14-Mar-2024 15:31 (GMT)

**Table 3. Schedule of Activities (Phase 3 Arm A and Arm B; Cohort 3 Arm D )**

| **Evaluation** Abbreviations used in this table may be found in Appendix 11. | **Treatment Period (Each 28 Day Cycle)** | | **Post-Treatment Period** | | | **Notes/Protocol Section** |
|---|---|---|---|---|---|---|
| | **Day 1** | **Day 15** | **End-of-Treatment (EOT) Visit** | **Follow-up Visit (28 days post last dose)** | **Long Term Follow-up (Every 3 months)** | End of Treatment is defined as when participant permanently discontinues all study treatment.<br>Cycle 1 Day 1 assessments, unless otherwise specified, do not need to be repeated if performed within 72 hours prior to initial treatment (ie, first day of dosing).<br><br>All remaining visits during the treatment period, EOT visit, and follow-up visit have a ± 3-day window.<br>Long Term-Follow-up visits have ±7-day window for assessments. |
| **Laboratory Assessments** | | | | | | |
| Hematology | X | X | X | X | | See Section 8.2.8 and Table 35 |
| Chemistry | X | X | X | X | | See Section 8.2.8 and Table 35 |
| Coagulation | X | X | X | X | | See Section 8.2.8 and Table 35 |
| Urinalysis | X | | X | X | | Full urinalysis on Cycle 1 Day 1, only urine protein thereafter.<br>See Section 8.2.8 and Table 35 |
| Urine pregnancy test | X | | X | X | | See Section 8.2.9 and Table 35 |

090177e1a02cb071\Approved\Approved On: 14-Mar-2024 15:31 (GMT)

**Table 3. Schedule of Activities (Phase 3 Arm A and Arm B; Cohort 3 Arm D )**

| **Evaluation**<br>Abbreviations used in this table may be found in Appendix 11. | **Treatment Period (Each 28 Day Cycle)** | | **Post-Treatment Period** | | | **Notes/Protocol Section** |
|---|---|---|---|---|---|---|
| | **Day 1** | **Day 15** | **End-of-Treatment (EOT) Visit** | **Follow-up Visit (28 days post last dose)** | **Long Term Follow-up (Every 3 months)** | End of Treatment is defined as when participant permanently discontinues all study treatment.<br>Cycle 1 Day 1 assessments, unless otherwise specified, do not need to be repeated if performed within 72 hours prior to initial treatment (ie, first day of dosing).<br>All remaining visits during the treatment period, EOT visit, and follow-up visit have a ± 3-day window.<br>Long Term-Follow-up visits have ±7-day window for assessments. |
| Blood sample for PK | Cycles 1-6 | | | | | With the exception of Cycle 1 Day 1, which may be performed within 72 hours prior to initial treatment, PK sampling must occur on the Day of dosing encorafenib in the clinic (ie, no 3 day window).<br>Day 1: Predose See Section 8.5<br>For participants in mainland China, see Section 8.5.1 |
| Blood sample for CRP | X | | X | | | See Section 8.8.1 |
| Blood sample for CEA | X | | X | | | See Section 8.8.1 |

090177e1a02cb071\Approved\Approved On: 14-Mar-2024 15:31 (GMT)

**Table 3. Schedule of Activities (Phase 3 Arm A and Arm B; Cohort 3 Arm D )**

| **Evaluation** Abbreviations used in this table may be found in Appendix 11. | **Treatment Period (Each 28 Day Cycle)** | | **Post-Treatment Period** | | | **Notes/Protocol Section** |
|---|---|---|---|---|---|---|
| | **Day 1** | **Day 15** | **End-of-Treatment (EOT) Visit** | **Follow-up Visit (28 days post last dose)** | **Long Term Follow-up (Every 3 months)** | End of Treatment is defined as when participant permanently discontinues all study treatment.<br>Cycle 1 Day 1 assessments, unless otherwise specified, do not need to be repeated if performed within 72 hours prior to initial treatment (ie, first day of dosing).<br>All remaining visits during the treatment period, EOT visit, and follow-up visit have a ± 3-day window.<br>Long Term-Follow-up visits have ±7-day window for assessments. |
| Blood sample for cfDNA | Cycles 1, 7 | Cycles 1, 2 | X | | | Collect 20 mL (or 10 mL for mainland China sites) of blood optimized for plasma preparation at indicated visits (all pre-dose).<br>See Section 8.8.3 |
| Blood sample for protein biomarkers | Cycles 1, 2 | Cycles 1, 2 | X | | | Collect 10 mL (or 1 mL for mainland China sites) of whole blood for processing into serum at indicated visits (all pre-dose).<br>See Section 8.8.5 |
| Blood sample for germline comparator | Cycle 1 | | | | | Collect a 4 mL blood biospecimen prior to first dose of study intervention.<br>See Section 8.8.2 |

090177e1a02cb071\Approved\Approved On: 14-Mar-2024 15:31 (GMT)

**Table 3. Schedule of Activities (Phase 3 Arm A and Arm B; Cohort 3 Arm D )**

| **Evaluation**<br>Abbreviations used in this table may be found in Appendix 11. | **Treatment Period (Each 28 Day Cycle)** | | **Post-Treatment Period** | | | **Notes/Protocol Section** |
|---|---|---|---|---|---|---|
| | **Day 1** | **Day 15** | **End-of-Treatment (EOT) Visit** | **Follow-up Visit (28 days post last dose)** | **Long Term Follow-up (Every 3 months)** | End of Treatment is defined as when participant permanently discontinues all study treatment.<br>Cycle 1 Day 1 assessments, unless otherwise specified, do not need to be repeated if performed within 72 hours prior to initial treatment (ie, first day of dosing).<br><br>All remaining visits during the treatment period, EOT visit, and follow-up visit have a ± 3-day window.<br>Long Term-Follow-up visits have ±7-day window for assessments. |
| Blood sample for banked biospecimen | Cycle 1 | | | | | Unless prohibited by local regulations or ethics committee decision, collect a blood sample (4 mL) optimized for genomic analysis before the first dose of study intervention.<br>If not collected on the designated collection day, collect at the next available time point when biospecimens are being collected in conjunction with a participant visit.<br>See Section 8.7.7 |
| FFPE Tumor Tissue | X (optional) | | | | | On-treatment biopsies, including surgical specimens, should be collected when available but are optional.<br>See Section 8.8.2 and Section 8.8.4 |

090177e1a02cb071\Approved\Approved On: 14-Mar-2024 15:31 (GMT)

**Table 4. Schedule of Activities (Phase 3 Control Arm [Arm C]: mFOLFOX6 or FOLFOXIRI ± Bevacizumab; Cohort 3 Arm E: FOLFIRI ±Bevacizumab)**

| **Evaluation** Abbreviations used in this table may be found in Appendix 11. | **Treatment Period (Each 28 Day Cycle)** | | **Post-Treatment Period** | | | **Notes/Protocol Section** |
|---|---|---|---|---|---|---|
| | **Day 1** | **Day 15** | **End-of-Treatment (EOT) Visit** | **Follow-up Visit (28 days post last dose)** | **Long Term Follow-up (Every 3 months)** | End of Treatment is defined as when participant permanently discontinues all study treatment.<br>Cycle 1 Day 1 assessments, unless otherwise specified, do not need to be repeated if performed within 72 hours prior to initial treatment (ie, first day of dosing).<br>All remaining visits during the treatment period, EOT visit, and follow-up visit have a ± 3-day window.<br>Long Term-Follow-up visits have a ±7-day window for assessments. |
| **Administrative Procedures** | | | | | | |
| Verify inclusion/exclusion criteria | Cycle 1 | | | | | Must occur prior to first dose.<br>See Section 5.1.2 and Section 5.2.2 |
| Verify demography; medical history from Screening to Day 1 | Cycle 1 | | | | | See Section 8.2.1 and Section 8.2.2 |
| Contraceptive check | X | | X | X | X | Performed monthly during long term follow-up; recommended to be followed for 9 months for females and 6 months for males following the last dose of study intervention.<br>See Section 5.3.1 |
| Concomitant medication/ therapies | Assess continuously | | | | | See Section 6.5 |

090177e1a02cb071\Approved\Approved On: 14-Mar-2024 15:31 (GMT)

**Table 4. Schedule of Activities (Phase 3 Control Arm [Arm C]: mFOLFOX6 or FOLFOXIRI ± Bevacizumab; Cohort 3 Arm E: FOLFIRI ±Bevacizumab)**

| **Evaluation** Abbreviations used in this table may be found in Appendix 11. | **Treatment Period (Each 28 Day Cycle)** | | **Post-Treatment Period** | | | **Notes/Protocol Section** |
|---|---|---|---|---|---|---|
| | **Day 1** | **Day 15** | **End-of-Treatment (EOT) Visit** | **Follow-up Visit (28 days post last dose)** | **Long Term Follow-up (Every 3 months)** | End of Treatment is defined as when participant permanently discontinues all study treatment.<br>Cycle 1 Day 1 assessments, unless otherwise specified, do not need to be repeated if performed within 72 hours prior to initial treatment (ie, first day of dosing).<br>All remaining visits during the treatment period, EOT visit, and follow-up visit have a ± 3-day window.<br>Long Term-Follow-up visits have a ±7-day window for assessments. |
| PGIS, PGIC, EORTC QLQ-C30, and EQ-5D-5L | Q6W for the first 18 months after the date of randomization then Q8W thereafter or until End of Treatment | | X | X | | While on treatment, PRO assessments should be done with the same frequency as the tumor radiographic assessments.<br>PGIC will not be performed at baseline.<br>See Section 8.1.2 |
| ECOG PS | Cycle 1 | | | | | See Section 8.2.10 |
| Document survival status | | | | X | X | After discontinuation of study intervention, participants will be followed approximately every 3 months until withdrawal of consent/assent, the participant is lost to follow-up, death, or final OS analysis.<br>See Section 7.1 |
| Document subsequent therapies and dates of progression | | | Assess continuously | | | See Section 7.1 |

090177e1a02cb071\Approved\Approved On: 14-Mar-2024 15:31 (GMT)

**Table 4. Schedule of Activities (Phase 3 Control Arm [Arm C]: mFOLFOX6 or FOLFOXIRI ± Bevacizumab; Cohort 3 Arm E: FOLFIRI ±Bevacizumab)**

| **Evaluation** Abbreviations used in this table may be found in Appendix 11. | **Treatment Period (Each 28 Day Cycle)** | | **Post-Treatment Period** | | | **Notes/Protocol Section** |
|---|---|---|---|---|---|---|
| | **Day 1** | **Day 15** | **End-of-Treatment (EOT) Visit** | **Follow-up Visit (28 days post last dose)** | **Long Term Follow-up (Every 3 months)** | End of Treatment is defined as when participant permanently discontinues all study treatment.<br>Cycle 1 Day 1 assessments, unless otherwise specified, do not need to be repeated if performed within 72 hours prior to initial treatment (ie, first day of dosing).<br>All remaining visits during the treatment period, EOT visit, and follow-up visit have a ± 3-day window.<br>Long Term-Follow-up visits have a ±7-day window for assessments. |
| mFOLFOX6 (Arm C only), FOLFOXIRI (Arm C only),<br>FOLFIRI (Arm E only)administration | X | X | | | | Choice of treatment regimen is at the participant/treating investigator's discretion but must be declared prior to randomization and must not be changed during the course of the participant's treatment.<br>See Section 6.1.3.2 |
| Bevacizumab administration (optional) | X | X | | | | If the investigator decides to administer bevacizumab (Zirabev™ or Avastin®), Zirabev™ must be administered where approved. Where Zirabev™ is not approved, Avastin® may be administered. The decision to use bevacizumab must be made prior to randomization.<br>See Section 6.1.2 and Section 6.1.3.2 |
| Assess study intervention compliance | X | | X | | | See Section 6.4 |
| **Clinical Procedures/Assessments** | | | | | | |
| Physical exam | X | | X | X | | Exam to include visual assessment.<br>See Section 8.2.3 |

090177e1a02cb071\Approved\Approved On: 14-Mar-2024 15:31 (GMT)

**Table 4. Schedule of Activities (Phase 3 Control Arm [Arm C]: mFOLFOX6 or FOLFOXIRI ± Bevacizumab; Cohort 3 Arm E: FOLFIRI ±Bevacizumab)**

| **Evaluation** Abbreviations used in this table may be found in Appendix 11. | **Treatment Period (Each 28 Day Cycle)** | | **Post-Treatment Period** | | | **Notes/Protocol Section** |
|---|---|---|---|---|---|---|
| | **Day 1** | **Day 15** | **End-of-Treatment (EOT) Visit** | **Follow-up Visit (28 days post last dose)** | **Long Term Follow-up (Every 3 months)** | End of Treatment is defined as when participant permanently discontinues all study treatment.<br>Cycle 1 Day 1 assessments, unless otherwise specified, do not need to be repeated if performed within 72 hours prior to initial treatment (ie, first day of dosing).<br>All remaining visits during the treatment period, EOT visit, and follow-up visit have a ± 3-day window.<br>Long Term-Follow-up visits have a ±7-day window for assessments. |
| Weight | X | X | | | | See Section 8.2.3 |
| Height | X | | | | | Height to be measured in participants <18 years old.<br>See Section 8.2.3 |
| BSA | X | | | | | See Section 8.2.4 |
| Vital signs | X | X | X | X | | See Section 8.2.6 |
| Triplicate 12-lead ECG | Cycle 1 | | | | | Must be conducted on Cycle 1 Day 1 (ie, no window). Predose only.<br>See Section 8.2.7 |
| Single 12-lead ECG | Cycles 2+ | | X | X | | Predose only. Perform continuous ECGs if QTc abnormality.<br>See Section 8.2.7 |
| Tumor radiographic assessment (CT, MRI) | The first post-baseline scan must be performed 42 to 49 days from the date of randomization then Q6W (± 7D) for the first 18 months after the date of randomization then Q8W (± 7D) thereafter or until BICR-confirmed PD (regardless of new anticancer therapy), withdrawal of consent/assent, participant is lost to follow-up, death, or final OS analysis. | | | | | See Section 8.1.1 |

090177e1a02cb071\Approved\Approved On: 14-Mar-2024 15:31 (GMT)

**Table 4. Schedule of Activities (Phase 3 Control Arm [Arm C]: mFOLFOX6 or FOLFOXIRI ± Bevacizumab; Cohort 3 Arm E: FOLFIRI ±Bevacizumab)**

| **Evaluation** Abbreviations used in this table may be found in Appendix 11. | **Treatment Period (Each 28 Day Cycle)** | | **Post-Treatment Period** | | | **Notes/Protocol Section** |
|---|---|---|---|---|---|---|
| | **Day 1** | **Day 15** | **End-of-Treatment (EOT) Visit** | **Follow-up Visit (28 days post last dose)** | **Long Term Follow-up (Every 3 months)** | End of Treatment is defined as when participant permanently discontinues all study treatment.<br>Cycle 1 Day 1 assessments, unless otherwise specified, do not need to be repeated if performed within 72 hours prior to initial treatment (ie, first day of dosing).<br>All remaining visits during the treatment period, EOT visit, and follow-up visit have a ± 3-day window.<br>Long Term-Follow-up visits have a ±7-day window for assessments. |
| Adverse event assessment | Assess continuously | | | | | See Section 8.3 and Appendix 3 |
| **Laboratory Assessments** | | | | | | |
| Hematology | X | X | X | X | | See Section 8.2.8 and Table 35 |
| Chemistry | X | X | X | X | | See Section 8.2.8 and Table 35 |
| Coagulation | X | X | X | X | | See Section 8.2.8 and Table 35 |
| Urinalysis | X | | X | X | | Full urinalysis on Cycle 1 Day 1, only urine protein thereafter.<br>See Section 8.2.8 and Table 35 |
| Urine pregnancy test | X | | X | X | | See Section 8.2.9 and Table 35 |
| Blood samples for CRP | X | | X | | | See Section 8.8.1 |
| Blood sample for CEA | X | | X | | | See Section 8.8.1 |

090177e1a02cb071\Approved\Approved On: 14-Mar-2024 15:31 (GMT)

**Table 4. Schedule of Activities (Phase 3 Control Arm [Arm C]: mFOLFOX6 or FOLFOXIRI ± Bevacizumab; Cohort 3 Arm E: FOLFIRI ±Bevacizumab)**

| **Evaluation** Abbreviations used in this table may be found in Appendix 11. | **Treatment Period (Each 28 Day Cycle)** | | **Post-Treatment Period** | | | **Notes/Protocol Section** |
|---|---|---|---|---|---|---|
| | **Day 1** | **Day 15** | **End-of-Treatment (EOT) Visit** | **Follow-up Visit (28 days post last dose)** | **Long Term Follow-up (Every 3 months)** | End of Treatment is defined as when participant permanently discontinues all study treatment.<br>Cycle 1 Day 1 assessments, unless otherwise specified, do not need to be repeated if performed within 72 hours prior to initial treatment (ie, first day of dosing).<br>All remaining visits during the treatment period, EOT visit, and follow-up visit have a ± 3-day window.<br>Long Term-Follow-up visits have a ±7-day window for assessments. |
| Blood sample for cfDNA | Cycles 1, 7 | Cycles 1, 2 | X | | | Collect 20 mL (or 10 mL for mainland China sites) of blood optimized for plasma preparation at indicated visits (all pre-dose).<br>See Section 8.8.3 |
| Blood sample for protein biomarkers | Cycles 1, 2 | Cycles 1, 2 | X | | | Collect 10 mL (or 1 mL for mainland China sites) of whole blood for processing into serum at indicated visits (all pre-dose).<br>See Section 8.8.5 |
| Blood sample for germline comparator | Cycle 1 | | | | | Collect a 4 mL blood biospecimen prior to first dose of study intervention.<br>See Section 8.8.2 |
| Blood sample for banked biospecimen | Cycle 1 | | | | | Unless prohibited by local regulations or ethics committee decision, collect a blood sample (4 mL) optimized for genomic analysis before the first dose of study intervention.<br>If not collected on the designated collection day, collect at the next available time point when biospecimens are being collected in conjunction with a participant visit.<br>See Section 8.7.7 |

090177e1a02cb071\Approved\Approved On: 14-Mar-2024 15:31 (GMT)

**Table 4. Schedule of Activities (Phase 3 Control Arm [Arm C]: mFOLFOX6 or FOLFOXIRI ± Bevacizumab; Cohort 3 Arm E: FOLFIRI ±Bevacizumab)**

| **Evaluation** Abbreviations used in this table may be found in Appendix 11. | **Treatment Period (Each 28 Day Cycle)** | | **Post-Treatment Period** | | | **Notes/Protocol Section** |
|---|---|---|---|---|---|---|
| | **Day 1** | **Day 15** | **End-of-Treatment (EOT) Visit** | **Follow-up Visit (28 days post last dose)** | **Long Term Follow-up (Every 3 months)** | End of Treatment is defined as when participant permanently discontinues all study treatment. Cycle 1 Day 1 assessments, unless otherwise specified, do not need to be repeated if performed within 72 hours prior to initial treatment (ie, first day of dosing). All remaining visits during the treatment period, EOT visit, and follow-up visit have a ± 3-day window. Long Term-Follow-up visits have a ±7-day window for assessments. |
| FFPE Tumor Tissue | X (optional) | | | | | On-treatment biopsies, including surgical specimens, should be collected when available but are optional. See Section 8.8.2 and Section 8.8.4 |

090177e1a02cb071\Approved\Approved On: 14-Mar-2024 15:31 (GMT)

**Table 5. Schedule of Activities (Control Arm [Arm C]: CAPOX ± Bevacizumab)**

| **Evaluation/Window** Abbreviations used in this table may be found in Appendix 11. | **Treatment Period (Each 21 Day Cycle)** | **Post-Treatment Period** | | | **Notes/Protocol Section** |
|---|---|---|---|---|---|
| | **Day 1** | **End-of-Treatment (EOT) Visit** | **Follow-up Visit (28 days post last dose)** | **Long Term Follow-up (Every 3 months)** | End of Treatment is defined as when participant permanently discontinues all study treatment.<br>Cycle 1 Day 1 assessments, unless otherwise specified, do not need to be repeated if performed within 72 hours prior to initial treatment (ie, first day of dosing).<br>All remaining visits during the treatment period, EOT visit, and follow-up visit have a ± 3-day window.<br>Long Term Follow-up visits have a ±7-day window for assessments. |
| **Administrative Procedures** | | | | | |
| Verify inclusion/ exclusion criteria | Cycle 1 | | | | Must occur prior to first dose.<br>See Section 5.1.2 and Section 5.2.2 |
| Verify demography; medical history from Screening to Day 1 | Cycle 1 | | | | See Section 8.2.1 and Section 8.2.2 |
| Contraceptive check | X | X | X | X | Performed monthly during long term follow-up; recommended to be followed for 9 months for females and 6 months for males following the last dose of study intervention.<br>See Section 5.3.1 |
| Concomitant medication/therapies | Assess continuously | | | | See Section 6.5 |
| PGIS, PGIC, EORTC QLQ-C30, and EQ-5D-5L | Q6W for the first 18 months after the date of randomization then Q8W thereafter or until End of Treatment | X | X | | While on treatment, PRO assessments should be done with the same frequency as the tumor radiographic assessments.<br>PGIC will not be performed at baseline.<br>See Section 8.1.2 |

090177e1a02cb071\Approved\Approved On: 14-Mar-2024 15:31 (GMT)

**Table 5. Schedule of Activities (Control Arm [Arm C]: CAPOX ± Bevacizumab)**

| **Evaluation/Window** Abbreviations used in this table may be found in Appendix 11. | **Treatment Period (Each 21 Day Cycle)** | **Post-Treatment Period** | | | **Notes/Protocol Section** |
|---|---|---|---|---|---|
| | **Day 1** | **End-of-Treatment (EOT) Visit** | **Follow-up Visit (28 days post last dose)** | **Long Term Follow-up (Every 3 months)** | End of Treatment is defined as when participant permanently discontinues all study treatment.<br>Cycle 1 Day 1 assessments, unless otherwise specified, do not need to be repeated if performed within 72 hours prior to initial treatment (ie, first day of dosing).<br>All remaining visits during the treatment period, EOT visit, and follow-up visit have a ± 3-day window.<br>Long Term Follow-up visits have a ±7-day window for assessments. |
| ECOG PS | Cycle 1 | | | | See Section 8.2.10 |
| Document survival status | | | X | X | After discontinuation of study intervention, participants will be followed approximately every 3 months until withdrawal of consent/assent, the participant is lost to follow-up, death, or final OS analysis.<br>See Section 7.1 |
| Document subsequent therapies and dates of progression | | Assess continuously | | | See Section 7.1 |
| Dispense capecitabine (with diary) | X | | | | Choice of treatment regimen is at the participant/treating Investigator's discretion but must be declared prior to randomization and must not be changed during the course of the participant's treatment.<br>See Section 6.1.3.2 |
| Oxaliplatin administration | X | | | | See Section 6.1.3.2 |

090177e1a02cb071\Approved\Approved On: 14-Mar-2024 15:31 (GMT)

**Table 5. Schedule of Activities (Control Arm [Arm C]: CAPOX ± Bevacizumab)**

| **Evaluation/Window**<br>Abbreviations used in this table may be found in Appendix 11. | **Treatment Period (Each 21 Day Cycle)** | **Post-Treatment Period** | | | **Notes/Protocol Section** |
|---|---|---|---|---|---|
| | **Day 1** | **End-of-Treatment (EOT) Visit** | **Follow-up Visit (28 days post last dose)** | **Long Term Follow-up (Every 3 months)** | End of Treatment is defined as when participant permanently discontinues all study treatment.<br>Cycle 1 Day 1 assessments, unless otherwise specified, do not need to be repeated if performed within 72 hours prior to initial treatment (ie, first day of dosing).<br>All remaining visits during the treatment period, EOT visit, and follow-up visit have a ± 3-day window.<br>Long Term Follow-up visits have a ±7-day window for assessments. |
| Bevacizumab administration (optional) | X | | | | If the investigator decides to administer bevacizumab (Zirabev™ or Avastin®), Zirabev™ must be administered where approved. Where Zirabev™ is not approved, Avastin® may be administered. The decision to use bevacizumab must be made prior to randomization.<br>See Section 6.1.2 and 6.1.3.2 |
| Assess study intervention compliance | X | X | | | See Section 6.4 |
| **Clinical Procedures/Assessments** | | | | | |
| Physical exam | X | X | X | | Exam to include visual assessment.<br>See Section 8.2.3 |
| Weight | X | | | | See Section 8.2.3 |
| Height | X | | | | Height to be measured in participants <18 years old.<br>See Section 8.2.3 |
| BSA | X | | | | See Section 8.2.4 |
| Vital signs | X | X | X | | See Section 8.2.6 |

090177e1a02cb071\Approved\Approved On: 14-Mar-2024 15:31 (GMT)

**Table 5. Schedule of Activities (Control Arm [Arm C]: CAPOX ± Bevacizumab)**

| **Evaluation/Window** Abbreviations used in this table may be found in Appendix 11. | **Treatment Period (Each 21 Day Cycle)** | **Post-Treatment Period** | | | **Notes/Protocol Section** |
|---|---|---|---|---|---|
| | **Day 1** | **End-of-Treatment (EOT) Visit** | **Follow-up Visit (28 days post last dose)** | **Long Term Follow-up (Every 3 months)** | End of Treatment is defined as when participant permanently discontinues all study treatment.<br>Cycle 1 Day 1 assessments, unless otherwise specified, do not need to be repeated if performed within 72 hours prior to initial treatment (ie, first day of dosing).<br>All remaining visits during the treatment period, EOT visit, and follow-up visit have a ± 3-day window.<br>Long Term Follow-up visits have a ±7-day window for assessments. |
| Triplicate 12-lead ECG | Cycle 1 | | | | Must be conducted on Cycle 1 Day 1 (ie, no window).<br>Predose only.<br>See Section 8.2.7 |
| Single 12-lead ECG | Cycles 2+ | X | X | | Predose only. Perform continuous ECGs if QTc abnormality.<br>See Section 8.2.7 |
| Tumor radiographic assessment | The first post-baseline scan must be performed 42 to 49 days from the date of randomization then Q6W (± 7D) for the first 18 months after the date of randomization then Q8W (± 7D) thereafter or until BICR-confirmed PD (regardless of new anticancer therapy), withdrawal of consent/assent, participant is lost to follow-up, death, or final OS analysis. | | | | See Section 8.1.1 |
| Adverse event assessment | Assess continuously | | | | See Section 8.3 and Appendix 3 |
| **Laboratory Assessments** | | | | | |
| Hematology | X | X | X | | See Section 8.2.8 and Table 35 |
| Chemistry | X | X | X | | See Section 8.2.8 and Table 35 |
| Coagulation | X | X | X | | See Section 8.2.8 and Table 35 |

090177e1a02cb071\Approved\Approved On: 14-Mar-2024 15:31 (GMT)

**Table 5. Schedule of Activities (Control Arm [Arm C]: CAPOX ± Bevacizumab)**

| **Evaluation/Window** Abbreviations used in this table may be found in Appendix 11. | **Treatment Period (Each 21 Day Cycle)** | **Post-Treatment Period** | | | **Notes/Protocol Section** |
|---|---|---|---|---|---|
| | **Day 1** | **End-of-Treatment (EOT) Visit** | **Follow-up Visit (28 days post last dose)** | **Long Term Follow-up (Every 3 months)** | End of Treatment is defined as when participant permanently discontinues all study treatment.<br>Cycle 1 Day 1 assessments, unless otherwise specified, do not need to be repeated if performed within 72 hours prior to initial treatment (ie, first day of dosing).<br>All remaining visits during the treatment period, EOT visit, and follow-up visit have a ± 3-day window.<br>Long Term Follow-up visits have a ±7-day window for assessments. |
| Urinalysis | X | X | X | | Full urinalysis on Cycle 1 Day 1, only urine protein thereafter.<br>See Section 8.2.8 and Table 35 |
| Urine pregnancy test | X | X | X | | See Section 8.2.9 and Table 35 |
| Blood sample for CRP | X | X | | | See Section 8.8.1 |
| Blood sample for CEA | X | X | | | See Section 8.8.1 |
| Blood sample for cfDNA | Cycles 1-3, 9 | X | | | Collect 20 mL (or 10 mL for mainland China sites) of blood optimized for plasma preparation at indicated visits (all pre-dose).<br>See Section 8.8.3 |
| Blood sample for protein biomarkers | Cycles 1-3 | X | | | Collect 10 mL (or 1 mL for mainland China sites) of whole blood for processing into serum at indicated visits (all pre-dose).<br>See Section 8.8.5 |
| Blood sample for germline comparator | Cycle 1 | | | | Collect a 4 mL blood biospecimen prior to first dose of study intervention.<br>See Section 8.8.2 |

**Table 5. Schedule of Activities (Control Arm [Arm C]: CAPOX ± Bevacizumab)**

| **Evaluation/Window** Abbreviations used in this table may be found in Appendix 11. | **Treatment Period (Each 21 Day Cycle)** | **Post-Treatment Period** | | | **Notes/Protocol Section** |
|---|---|---|---|---|---|
| | **Day 1** | **End-of-Treatment (EOT) Visit** | **Follow-up Visit (28 days post last dose)** | **Long Term Follow-up (Every 3 months)** | End of Treatment is defined as when participant permanently discontinues all study treatment.<br>Cycle 1 Day 1 assessments, unless otherwise specified, do not need to be repeated if performed within 72 hours prior to initial treatment (ie, first day of dosing).<br>All remaining visits during the treatment period, EOT visit, and follow-up visit have a ± 3-day window.<br>Long Term Follow-up visits have a ±7-day window for assessments. |
| Blood sample for banked biospecimen | Cycle 1 | | | | Unless prohibited by local regulations or ethics committee decision, collect a blood sample (4 mL) optimized for genomic analysis before the first dose of study intervention.<br>If not collected on the designated collection day, collect at the next available time point when biospecimens are being collected in conjunction with a participant visit.<br>See Section 8.7.7 |
| FFPE tumor tissue | X (optional) | | | | On-treatment biopsies, including surgical specimens, should be collected when available but are optional.<br>See Section 8.8.2 and Section 8.8.4 |



090177e1a02cb071\Approved\Approved On: 14-Mar-2024 15:31 (GMT)

# 2. INTRODUCTION

Encorafenib (BRAFTOVI®, also known as PF-07263896, LGX818 or ONO-7702) is being investigated in this study in combination with cetuximab with or without mFOLFOX6 or FOLFIRI in participants with previously untreated metastatic *BRAF* V600E-mutant CRC.

## 2.1. Study Rationale

The purpose of the study is to evaluate whether encorafenib plus cetuximab (EC), alone or in combination with chemotherapy, can improve clinical outcomes relative to current standard-of-care chemotherapy in participants with previously untreated *BRAF* V600E-mutant mCRC. Since encorafenib has not previously been combined with chemotherapy, the tolerability and PK of EC in combination with mFOLFOX6 and in combination with FOLFIRI will be evaluated in separate cohorts in the safety lead-in portion of the trial in order to identify which chemotherapy combination is to be used in the Phase 3 portion of the study.

## 2.2. Background

### 2.2.1. *BRAF* V600E-Mutant Metastatic Colorectal Cancer

Metastatic colorectal cancer continues to be a life-threatening condition. Globally, colorectal cancer is the third most commonly diagnosed cancer in males and the second in females, with 1.8 million new cases and almost 861,000 deaths in 2018 according to the WHO.[1] In the US, according to the SEER database, the estimated number of new CRC cases for 2019 was 145,600 resulting in an estimated 51,000 deaths.[2] In Japan, CRC is the second most common cancer in women with over 150,000 new cases in 2017; there were over 50,000 deaths due to CRC and it was the leading cause of cancer death in women and third most common cause of cancer death in men in 2018.[3] In the WHO European Region, CRC is the most common tumor type, with 471,000 new cases each year and a mean mortality rate of 28.2 per 100,000 population.[4] Approximately 25% of patients present with metastases and 50% of patients eventually develop metastatic disease.[5]

*BRAF* mutations occur in approximately 10% to 15% of CRC, with a broad range of estimates ranging from as low as 5% to as high as 21%. These mutations primarily (>95%) occur at the V600E codon (consisting of a valine to glutamic acid substitution), are essentially mutually exclusive with *RAS* mutations,[6-11] and lead to constitutive activation of BRAF kinase and sustained RAS/RAF/MEK/ERK pathway signaling, resulting in increased cell proliferation and survival.[12]

*BRAF* mutations are associated with a poor prognosis.[13-17] Retrospective analyses of Phase 3 studies using standard regimens revealed patients with *BRAF*-mutant mCRC achieved mPFS of approximately 7 months and mOS of approximately 12 months compared to approximately 10 months PFS and 25 months OS in patients with *BRAF*-wt tumors.[16,18-23]

Previous studies have demonstrated an association between the presence of MSI-H or dMMR and presence of *BRAF* mutations in CRC tumors. Approximately 15-30% of *BRAF*-mutant mCRC tumors were also identified as MSI-H/dMMR.[14,16,24,25] Immune checkpoint inhibitors



090177e1a02cb071\Approved\Approved On: 14-Mar-2024 15:31 (GMT)

have been shown to be active in patients with previously treated MSI-H/dMMR mCRC[25,26] and more recently in previously untreated patients with mCRC as well.[27]

## 2.2.2. Clinical Overview

### 2.2.2.1. Current Recommendations for Treatment of *BRAF* V600E-mutant mCRC

Currently, no treatments are indicated specifically for patients with *BRAF* V600E-mutant mCRC in the first-line setting and thus patients receive systemic therapy that is recommended for mCRC in general. The common regimens used in patients with mCRC include mFOLFOX6 (oxaliplatin, leucovorin and fluorouracil [5-FU]), FOLFIRI (irinotecan, leucovorin and 5-FU), FOLFOXIRI (irinotecan, oxaliplatin, leucovorin and 5-FU) and CAPOX (oxaliplatin and capecitabine) each with or without bevacizumab.[28] Retrospective subgroup analyses of Phase 2 and 3 studies in previously untreated patients with mCRC patients have shown that all of the standard regimens have similar efficacy in patients with *BRAF*-mutant tumors (Table 6).

**Table 6. Efficacy of Standard of Care First-Line Therapies in Patients with *BRAF*-mutant mCRC**

| Regimen | *BRAF*-mutant n (%) | Median PFS (Months) | Median OS (Months) |
|---|---|---|---|
| FOLFOX4[22] | 25 (12.5) | 3.8 | NR |
| FOLFOX4 + cetuximab[22] | 27 (14.0) | 2.0 | NR |
| FOLFIRI + bevacizumab[19] | 12 (4.7) | 5.5 | 10.7 |
| FOLFOXIRI + bevacizumab[19] | 16 (6.3) | 7.5 | 19.0 |
| FOLFIRI or FOLFOX-4 (pooled)[18] | 38 (8.5) | 3.7 | 9.9 |
| FOLFIRI or FOLFOX-4 (pooled) + cetuximab[18] | 32 (8.0) | 7.1 | 14.1 |
| FOLFIRI + cetuximab[23] | 23 (7.7) | 6.6 | 12.3 |
| FOLFIRI + bevacizumab[23] | 25 (8.5) | 6.6 | 13.7 |
| CAIRO: capecitabine + irinotecan + oxaliplatin (sequential vs combination)[16]<br>CAIRO2: capecitabine + oxaliplatin + bevacizumab ± cetuximab[16]<br>COIN: capecitabine (or 5-FU + leucovorin) + oxaliplatin ± cetuximab[16]<br>FOCUS: 5-FU + leucovorin + irinotecan vs 5-FU + leucovorin ± irinotecan + oxaliplatin[16] | 250 (8.2) | 6.2 | 11.4 |
| CAPOX + bevacizumab in induction + capecitabine + bevacizumab[21] | 30 (5.4) | 9.5 | 15.8 |

The use of anti-EGFR antibodies such as cetuximab in patients with *BRAF*-mutant mCRC is currently a topic of debate, two meta-analyses of patients with mCRC treated in the 1st-line,



090177e1a02cb071\Approved\Approved On: 14-Mar-2024 15:31 (GMT)

2nd-line, or refractory settings, have shown no benefit for the addition of anti-EGFR antibodies to standard therapy or best supportive care.[29,30] In previously treated patients data suggest that mutated BRAF is a marker of resistance to anti-EGFR therapy.[31-33] In the 1st line mCRC setting, unplanned retrospective subset analyses have demonstrated contradictory findings of the effects of adding anti-EGFR antibodies to chemotherapy with some studies showing slight numerical but not statistically significant improvements in OS as discussed above, while others showing either no benefit or even detrimental effects.[20,34]

ESMO guidelines suggest that there is accumulating data that patients with *BRAF* mutant tumors do not benefit from anti-EGFR antibodies alone or in combination with cytotoxic chemotherapy and the NCCN guidelines state that responses are highly unlikely in patients with *BRAF* V600E-mutant mCRC treated with cetuximab as a single agent or in combination with cytotoxic chemotherapy unless administered concomitantly with a BRAF inhibitor.[28,35] The addition of anti-EGFR antibodies as part of 1[st] line therapy is only recommended in patients with *KRAS/NRAS/BRAF* wt and left-sided tumors.[18,28]

#### 2.2.2.2. Encorafenib + Cetuximab for the Treatment of *BRAF* V600E-mutant mCRC

Encorafenib is a highly selective ATP-competitive small-molecule RAF kinase inhibitor, with the greatest sensitivity observed in melanoma and CRC tumor cell lineages expressing *BRAF* V600E.[36] Encorafenib in combination with binimetinib (a potent and selective allosteric, ATP-uncompetitive inhibitor of MEK1/2) has received marketing approval in several jurisdictions for the treatment of patients with unresectable or metastatic melanoma with a *BRAF* V600E or V600K mutation.

Study ARRAY-818-302 (BEACON CRC; NCT02928224) is a Phase 3 trial in patients with *BRAF* V600E-mutant mCRC whose disease had progressed following 1 or 2 prior systemic therapies. Patients in the study were randomized 1:1:1 to receive either EC, EC + binimetinib, or Control (irinotecan + cetuximab or FOLFIRI + cetuximab). The dosage of encorafenib in all patients was 300 mg QD PO. The dosage of cetuximab in all patients was 400 mg/m$^2$ IV for the first dose followed by 250 mg/m$^2$ IV weekly.

In April 2020, based on the results of the Study ARRAY-818-302, the EC regimen received marketing approval in the US for the treatment of adult patients with mCRC with a *BRAF* V600E mutation, as detected by an FDA-approved test, after prior therapy.[37] In June 2020, the EC regimen received approval from the European Commission for the same indication.

**Efficacy**

A total of 220 patients were randomized to the EC arm and 221 to the Control Arm. The EC regimen demonstrated statistically significant improvement in OS, ORR, and PFS compared to the Control Arm as shown in Table 7. The addition of binimetinib to EC did not improve efficacy (ORR, PFS, or OS) significantly compared with EC.[38]

090177e1a02cb071\Approved\Approved On: 14-Mar-2024 15:31 (GMT)

**Table 7. Study ARRAY-818-302 (EC Arm and Control Arm): Efficacy Results**

| | EC<br>N = 220 | Irinotecan + Cetuximab or FOLFIRI + Cetuximab<br>N = 221 |
|---|---|---|
| Overall Survival | | |
| Median OS, months (95% CI) | 8.4 (7.5, 11.0) | 5.4 (4.8, 6.6) |
| HR (95% CI) | 0.60 (0.45, 0.79) | |
| p-value | <0.001 | |
| Overall Response Rate (per BICR) | | |
| ORR (95% CI) | 20% (13%, 29%) | 2% (0%, 7%) |
| CR | 5% | 0% |
| PR | 15% | 2% |
| Progression Free Survival (per BICR) | | |
| Median PFS, months (95% CI) | 4.2 (3.7, 5.4) | 1.5 (1.5, 1.7) |
| HR (95% CI) | 0.40 (0.31, 0.52) | |
| *P*-value | <0.001 | |

**Safety**

In study ARRAY-818-302, the safety of the EC regimen was evaluated in 216 patients. The most common adverse events in the EC treated patients were gastrointestinal-related and skin-related events, including nausea, diarrhea, and acneiform dermatitis (Table 8). Most of these toxicities were generally reversible and manageable by supportive medical care, dose modifications or discontinuations. Other important adverse events occurring in <15% of patients in the EC arm were melanocytic nevi (14%), and myalgia (13%).[38]

**Table 8. Study ARRAY-818-302 (EC Arm and Control Arm): Adverse Events Regardless of Causality Reported in ≥15% of EC Patients**

| | EC<br>(N = 216) | | Irinotecan + Cetuximab or FOLFIRI + Cetuximab<br>(N = 193) | |
|---|---|---|---|---|
| | Any Grade<br>n (%) | Grade ≥3<br>n (%) | Any Grade<br>n (%) | Grade ≥3<br>n (%) |
| Any adverse event | 212 (98) | 108 (50) | 188 (97) | 117 (61) |
| Nausea | 74 (34) | 1 (<1) | 80 (41) | 2 (1) |
| Diarrhea | 72 (33) | 4 (2) | 93 (48) | 19 (10) |
| Fatigue | 65 (30) | 9 (4) | 53 (27) | 8 (4) |
| Acneiform dermatitis | 63 (29) | 1 (<1) | 76 (39) | 5 (3) |
| Decreased appetite | 58 (27) | 3 (1) | 52 (27) | 6 (3) |
| Abdominal pain | 49 (23) | 5 (2) | 48 (25) | 9 (5) |
| Vomiting | 46 (21) | 3 (1) | 56 (29) | 5 (3) |

090177e1a02cb071\Approved\Approved On: 14-Mar-2024 15:31 (GMT)

**Table 8. Study ARRAY-818-302 (EC Arm and Control Arm): Adverse Events Regardless of Causality Reported in ≥15% of EC Patients**

| | EC (N = 216) | | Irinotecan + Cetuximab or FOLFIRI + Cetuximab (N = 193) | |
|---|---|---|---|---|
| | Any Grade n (%) | Grade ≥3 n (%) | Any Grade n (%) | Grade ≥3 n (%) |
| Asthenia | 46 (21) | 7 (3) | 49 (25) | 9 (5) |
| Headache | 42 (19) | 0 (0) | 5 (3) | 0 (0) |
| Arthralgia | 41 (19) | 2 (1) | 1 (1) | 0 (0) |
| Pyrexia | 35 (16) | 2 (1) | 27 (14) | 1 (1) |
| Constipation | 33 (15) | 0 (0) | 35 (18) | 2 (1) |

The most common laboratory abnormalities (≥2%, Grade ≥3) in patients treated in the EC arm were elevated bilirubin (2%), elevated creatinine (2%), and decreased hemoglobin (4%).[38]

#### 2.2.2.3. Management of Cancer Patients During COVID-19 Pandemic

Patients with advanced cancers are known to be at higher risk of contracting infections including COVID-19 infection possibly due to underlying immunosuppression and/or increased co-existing medical comorbidities.[39] Since advanced cancer is also associated with high risk of morbidity and mortality, the decision to initiate cancer therapies should be a clinical decision based on the patient's condition and access to supportive care. Recently established ESMO guidelines recommend that anti-cancer treatments be initiated within 6 weeks of diagnosis in patients with previously untreated mCRC and especially in those with good clinical condition and for whom the treatment may reduce tumor bulk, or reduce cancer-related symptoms or complications.[40] Due to the poor prognosis of patients with *BRAF* V600E mCRC, continuation of treatment in the frame of clinical trials is also recommended.[40] In cancer patients with COVID-19, increased mortality was associated with a variety of factors such as increased age, male sex, smoking, and number of co-morbidities.[41] Recent retrospective analyses have reported no association between all-cause mortality and treatment with cytotoxic chemotherapy within 28-30 days prior to COVID-19 diagnosis.[42,43]

### 2.3. Benefit/Risk Assessment

More detailed information about the known and expected benefits and risks and reasonably expected AEs of encorafenib may be found in the Investigator's Brochure, which is the SRSD for this study. The SRSDs for capecitabine and cetuximab are the respective EU SmPCs. The SRSDs for 5-FU, irinotecan, leucovorin (or levo-leucovorin which may be supplied), and oxaliplatin are the respective SmPCs (see Section 6.1). Hereafter leucovorin, folinic acid and calcium folinate are collectively referred to as leucovorin.

090177e1a02cb071\Approved\Approved On: 14-Mar-2024 15:31 (GMT)

In the Control Arm of Phase 3, investigators may choose to administer an anti-VEGF agent. If this decision is made to use an anti-VEGF agent, Zirabev™ must be administered where approved. In regions where Zirabev™ is not approved, participants will receive Avastin®. Hereafter, Zirabev™ and Avastin® are collectively referred to as bevacizumab. The SRSD for these agents is the Avastin® EU SmPC.

Detailed information about the benefits and risks of each study intervention, when administered as a single agent, can be found in the respective SRSDs. Potential risks of synergistic toxicities are outlined in Section 2.3.1. Investigators should refer to the approved local label of the products used in this trial (capecitabine, cetuximab, 5-FU, irinotecan, leucovorin, oxaliplatin, and bevacizumab) for the management of patients, especially concerning contraindications, duration of contraception, special warnings and precautions, posology adaptation in case of toxicity, monitoring, as well as medications that are contraindicated or that must be used with caution.

090177e1a02cb071\Approved\Approved On: 14-Mar-2024 15:31 (GMT)

090177e1a02cb071\Approved\Approved On: 14-Mar-2024 15:31 (GMT)

### 2.3.1. Risk Assessment

| Potential Risk of Clinical Significance | Summary of Data/Rationale for Risk | Mitigation Strategy |
|---|---|---|
| **Study Intervention: Treatment Regimens** | | |
| Potential Risks of Synergistic Toxicities | | |
| QT prolongation | In the single-agent encorafenib study CLGX818X2101, an increase of 12.2 milliseconds in QTcF was predicted following 450 mg encorafenib.<br>In the pooled encorafenib single-agent population, the incidence of new QTc prolongation >500 ms or change in QTc >60 ms from baseline (Grade 3/4), was 1.0% (3/301). There was 1 serious Grade 3 event of QT prolongation, which resolved after permanent discontinuation of encorafenib. There were no AEs of Torsades de pointes or ventricular arrhythmias in patients in the encorafenib single-agent pool.<br>In study ARRAY-818-302, 7 (3.2%) patients in the Doublet arm (encorafenib + cetuximab) had a newly occurring QTcF > 500 ms and increases from baseline of >60 ms were observed in 19 (8.8%) patients. There were no AEs of torsades de pointes, ventricular arrhythmias, pre-syncope, syncope, sudden death or cardiac arrest reported contemporaneously with ECG QTcF prolongation > 500 ms.<br>The CredibleMeds® database (https://www.crediblemeds.org/) lists oxaliplatin as having a known risk of TdP cardiac arrhythmia and 5-FU as having a potential risk of TdP. Both are listed as drugs to avoid in patients with congenital LQTS. | Risk for QT prolongation is appropriately described in the encorafenib IB.<br>Administration with medicinal products with a known potential to prolong QT/QTc should be avoided, where possible.<br>Participants at high risk of QT prolongation are excluded from the study as outlined in Section 5.2.2.<br>Monitoring and dose adjustments for QT prolongation are outlined in Table 17. |
| Peripheral Neuropathy | AEs related to peripheral neuropathies may potentially be more severe and/or more frequent in patients receiving combination treatment regimens with encorafenib and oxaliplatin.<br>In study ARRAY-818-302, 26 (12%) patients in the Doublet arm (encorafenib + cetuximab) had a peripheral neuropathy AE of any Grade and 3 (1.4%) patients had a Grade 3+ event. Most of the 26 AEs were PTs for sensory neuropathies. Almost all patients (95.5%) had received prior oxaliplatin. In the Phase 3 study of binimetinib + encorafenib versus vemurafenib in patients with BRAF-mutant melanoma (study CMEK162B2301), the rates of peripheral neuropathy were 12% and 13%, respectively. | Dose modifications for oxaliplatin in the setting of neurotoxicity are described in Table 23.<br>Nervous system disorders including peripheral neuropathy are described in the IB and considered identified risks for encorafenib. Dose modification for encorafenib in the setting of peripheral neuropathy AEs are described in Table 17. |

090177e1a02cb071\Approved\Approved On: 14-Mar-2024 15:31 (GMT)

| Potential Risk of Clinical Significance | Summary of Data/Rationale for Risk | Mitigation Strategy |
|---|---|---|
| | According to the oxaliplatin SmPC, symptoms of dysaesthesia/paraesthesia occur in up to 95% of patients and are most often reversible, although about 3% of patients present with persisting paraesthesias of moderate severity up to 3 years after discontinuation of treatment. Approximately 0.5% of patients have decreased functional activities that are unresolved after discontinuation. | |
| Gastrointestinal Toxicities | AEs related to GI toxicities may potentially be more severe and/or more frequent in patients receiving combination treatment regimens with encorafenib + cetuximab concomitantly with mFOLFOX6 or FOLFIRI.<br><br>In ARRAY-818-302, diarrhea, nausea, and vomiting occurred in 21-34% of the patients in the Doublet arm (encorafenib + cetuximab). Seventy-two (33%) patients had diarrhea ADRs (4 patients -Grade 3+), 74 (34%) patients had nausea ADRs (1 patient - Grade 3+), and 46 (21%) had vomiting ADRs (3 Grade 3+).<br><br>Nausea and vomiting are common (≥40%) in patients receiving oxaliplatin. In patients treated with irinotecan in combination with 5-FU on a Q2W regimen, Grade 3-4 diarrhea occurs in approximately 14% of patients. 5-FU is also known to cause severe diarrhea requiring electrolyte replacement and antidiarrheal therapy. | GI toxicities including diarrhea, nausea, and vomiting are known ADRs for encorafenib in combination with cetuximab and described in the encorafenib US and EU product labels.<br><br>Dose modifications for AEs of GI toxicity are described for the various treatment regimens in Section 6.5.3. |
| **Study Procedures** | | |
| CT or MRI procedures for tumor assessments. | CT scans expose the participant to a small dose of radiation. The contrast dye used with the CT scan may cause pain or burning when injected. The dye may worsen kidney function for those with kidney disease or are dehydrated. The dye may cause an allergic reaction.<br><br>MRI risks include possible reactions to metals due to magnets, loud noises from the machine causing hearing issues, increase in body temperature during long MRIs, and claustrophobia.<br><br>Participants in this study will be subjected to more frequent (every 6 weeks) CT/MRI scans than is typical for mCRC patients (every 8 weeks) for the first 18 months of the study. Based on this scanning schedule, a patient would receive 3 additional scans in the 18 month period compared with the typical scanning regimen. | Only experienced professionals will conduct the CT or MRI procedures. The participant will be asked about allergies to the contrast dye. Medical history will be reviewed for kidney disease, and information will be provided to minimize dehydration. If possible, an open MRI can be used for those experiencing claustrophobia. The participant may be offered earplugs and/or headphones to minimize the noise. |

090177e1a02cb071\Approved\Approved On: 14-Mar-2024 15:31 (GMT)

| Potential Risk of Clinical Significance | Summary of Data/Rationale for Risk | Mitigation Strategy |
|---|---|---|
| **Other** | | |
| *BRAF* testing | Patients will be eligible for the study based on identification of a *BRAF* V600E mutation. Inaccurate *BRAF* testing (ie false positive or false negative) may lead to inappropriate enrollment to or exclusion from this study. A participant with a false negative result may be excluded from receiving potentially beneficial study intervention. Alternatively, a false positive result may lead a participant to receive study intervention, which may be inappropriate given the tumor mutation status. | In cases where there is discordance between the local assay and central laboratory results, or if the central laboratory is not able to confirm presence of a *BRAF* V600E mutation due to inadequate or poor sample condition within 30 days of initiating study therapy, participants may only continue treatment if there is no clinical indication of deterioration or disease progression and the investigator determines that the participant is deriving benefit. In such instances, participants must be informed that the *BRAF* mutation status is unconfirmed and must acknowledge on the ICDs that includes this information and describes alternative treatment options. |
| COVID-19 | As discussed in Section 2.2.2.3, cancer patients are at increased risk of infections including COVID-19. In cancer patients who contract COVID-19, increased mortality has been shown to be primarily associated with increased age, male sex, and number of co-morbid medical conditions. Recent retrospective analyses have suggested that cytotoxic chemotherapy is not associated with increased mortality in patients with COVID-19. | Participants with any active infection including COVID-19 are excluded from the study.<br>The following is recommended for the administration of study intervention for participants who have active confirmed (positive by regulatory authority-approved test) or presumed (test pending/clinical suspicion) SARS-CoV-2 infection:<br>• For symptomatic participants with active SARS-CoV-2 infection, study intervention should be delayed for at least 14 days from the start of symptoms. This delay is intended to allow the resolution of symptoms of SARS-CoV-2 infection. |

| Potential Risk of Clinical Significance | Summary of Data/Rationale for Risk | Mitigation Strategy |
|---|---|---|
| | | • Prior to restarting treatment, the participant should be afebrile for 72 hours, and SARS-CoV-2-related symptoms should have recovered to ≤ Grade 1 for a minimum of 72 hours. Notify the study team when treatment is restarted.<br><br>Additional mitigation strategies are provided in Appendix 12. |
| Adolescent participation | The combination of encorafenib and cetuximab has not previously been evaluated in patients <18. Although rare, when it does occur, young-onset CRC tends to present with distant metastases more often than in older patients, have larger tumor size, and be associated with perineural or lymphovascular invasion and signet cell or mucinous histology. The standard of care treatment for young adult and adolescent patients with Stage IV CRC is the same as in older patients and consists of an oxaliplatin- or irinotecan-containing multiagent regimen combined with a biological agent.[44] The influence of pubertal changes (changes in body composition, maturation of liver metabolism, and renal function) on drug metabolism have not been shown to result in additional safety risks and the activity and maturation of the enzymes responsible for the metabolism of encorafenib are expected to be comparable to adults.[45-49] Thus no relevant changes in exposure due to enzyme differences are expected in adolescents.<br><br>The inclusion of younger patients is aligned with the recent emphasis on consideration of including adolescent patients in adult clinical trials in indications with the same basic biology and clinical outcomes for whom effective therapeutic options are lacking.[45,46,50] | Monitor results of ongoing study, C4221011 (A Multicenter, Open-label Phase 1b Study of the Combination of Binimetinib and Encorafenib in Adolescent Patients with Unresectable or Metastatic *BRAF* V600-mutant Melanoma) [NCT03878719], which is evaluating encorafenib 450 mg QD in combination with binimetinib (approved adult doses for patients with *BRAF* V600-mutant melanoma) in adolescent patients (aged 12 to <18 years old) with *BRAF* V600-mutant advanced melanoma. |

***Note***: The pooled encorafenib single-agent population data set was derived from 4 clinical studies (CLGX818X2103 [Phase 1b/2], CMEK162B2301 Part 1 [Phase 3], CMEK162X2102 [Phase 2], CLGX818AUS03 [Phase 2]) with a total of 301 total patients.



090177e1a02cb071\Approved\Approved On: 14-Mar-2024 15:31 (GMT)

090177e1a02cb071\Approved\Approved On: 14-Mar-2024 15:31 (GMT)

### 2.3.2. Benefit Assessment

The EC regimen is approved in several countries for the treatment of patients with *BRAF* V600E-mutant mCRC that has progressed after 1 or 2 prior regimens (Section 2.2.2). Based on the activity observed in previously treated patients, there is the potential for the EC regimen to be efficacious in the first-line metastatic setting in patients with *BRAF* V600E-mutant mCRC. Furthermore, based on its demonstrated activity in patients with *BRAF* V600E-mutant mCRC, there is the potential for additional benefits to patients when EC is added to chemotherapy regimens that are the current SOC.

### 2.3.3. Overall Benefit/Risk Conclusion

Considering the measures taken to minimize risk to study participants, the potential risks identified in association with the EC regimen with or without mFOLFOX6 or FOLFIRI are justified by the anticipated benefits that may be afforded to participants with *BRAF* V600E-mutant mCRC.

The potential risks of study intervention in association with COVID-19 infection are justified by the anticipated benefits in participants with *BRAF* V600E mCRC.

## 3. OBJECTIVES, ESTIMANDS, AND ENDPOINTS

| **Safety Lead-in** | |
|---|---|
| **Objectives** | **Endpoints** |
| **Primary** | |
| • To determine the safety and tolerability of EC + mFOLFOX6 and EC + FOLFIRI | • Incidence of DLTs |
| **Secondary** | |
| • To assess the overall safety and tolerability of EC + mFOLFOX6 and EC + FOLFIRI | • Incidence and severity of AEs graded according to the NCI CTCAE v4.03 and changes in clinical laboratory parameters, vital signs and ECGs<br>• Incidence of dose interruptions, dose modifications and discontinuations due to AEs |
| • To estimate the efficacy of EC + mFOLFOX6 and EC + FOLFIRI | • ORR by Investigator, defined as the proportion of participants who have achieved a confirmed BOR (CR or PR) per RECIST v1.1<br>• DOR by Investigator, defined as the time from the date of first radiographic evidence of response (CR or PR) to the earliest documented disease progression per RECIST v1.1, or death due to any cause<br>• PFS by Investigator, defined as the time from the first dose to the earliest documented disease progression per RECIST v1.1, or death due to any cause<br>• TTR by Investigator, defined as the time from first dose to first radiographic evidence of response (CR or PR) per RECIST v1.1 |

| | |
|---|---|
| • To estimate the efficacy of EC + mFOLFOX6 and EC + FOLFIRI | • OS defined as the time from the first dose to death due to any cause |
| • To characterize the PK of encorafenib, irinotecan, oxaliplatin and relevant metabolites | • PK parameters of encorafenib, irinotecan, oxaliplatin and relevant metabolites |
| • To assess drug-drug interaction of encorafenib with irinotecan or oxaliplatin | • Changes in exposures of irinotecan and its metabolite (SN-38) on Cycle 1 Day 15 compared to Cycle 1 Day 1 in Cohort 1 (EC + FOLFIRI)<br>• Changes in exposures of oxaliplatin on Cycle 1 Day 15 compared to Cycle 1 Day 1 in Cohort 2 (EC + mFOLFOX6) |
| **Tertiary/Exploratory** | |
| • To understand the relationship between the therapeutic intervention(s) being studied and the biology of the participant's disease | • Measurements of biomarkers, consisting of DNA, RNA, protein or defined cell types, resulting from analyses of peripheral blood and/or tumor tissue biospecimen obtained at baseline, on treatment and/or at end-of-study |
| **Phase 3** | |
| **Objectives** | **Endpoints** |
| **Primary** | |
| • To compare the efficacy of EC + mFOLFOX6 (Arm B) vs SOC (Control Arm [Arm C]) as measured by PFS and by ORR | • PFS by BICR, defined as the time from the date of randomization to the earliest documented disease progression per RECIST v1.1, or death due to any cause<br>• ORR by BICR |
| **Key Secondary** | |
| • To further compare the efficacy of Arm B vs the Control Arm as measured by OS | • OS, defined as the time from the date of randomization to death due to any cause |
| **Secondary (Descriptive Statistics Only)** | |
| • To further evaluate the efficacy of Arm B vs the Control Arm as measured by ORR, DOR, PFS, PFS2 and TTR<br>• To evaluate the efficacy of EC (Arm A) vs the Control Arm as measured by ORR, DOR, PFS, PFS2, TTR, and OS<br>• To evaluate the efficacy of Arm A vs Arm B as measured by OS, PFS, PFS2, ORR, DOR and TTR | • ORR by Investigator<br>• ORR by BICR (Arm A vs Control Arm, Arm A vs Arm B)<br>• DOR by BICR and by Investigator<br>• PFS by BICR (Arm A vs Control Arm, Arm A vs Arm B)<br>• OS (Arm A vs Control Arm, Arm A vs Arm B)<br>• PFS by Investigator<br>• TTR (by BICR and by Investigator), defined as the time from the date of randomization to first radiographic evidence of response (CR or PR) per RECIST v1.1<br>• PFS2, defined as the time from the date of randomization to the date of discontinuation of next-line treatment after first objective PD by investigator assessment, the second objective |



090177e1a02cb071\Approved\Approved On: 14-Mar-2024 15:31 (GMT)

| | |
|---|---|
| | disease progression, or death from any cause, whichever occurs first |
| • To determine the safety and tolerability of EC<br>• To determine the safety and tolerability of EC + mFOLFOX6 | • Incidence and severity of AEs graded according to the NCI CTCAE v4.03 and changes in clinical laboratory parameters, vital signs, and ECGs |
| • To evaluate quality of life and health states, captured by PRO measures | • PRO scores as measured by the EORTC QLQ-C30, EQ-5D-5L, and anchoring instruments PGIS and PGIC. |
| • To evaluate trough concentrations of encorafenib and its metabolite LHY746 in Arm A and Arm B | • Trough plasma concentrations of encorafenib and the metabolite LHY746 in Arm A and Arm B |
| • To characterize the PK of encorafenib and its metabolite LHY746 in participants randomized in mainland China (Arm A and Arm B) | • PK parameters of encorafenib and its metabolite LHY746 |
| • To confirm the MSI-status in tumor tissue | • Summarize MSI-status as determined by retrospective central testing of baseline tumor tissue |
| • To determine the correlation between ctDNA levels, *BRAF* V600 alterations, and clinical outcome | • ctDNA levels and *BRAF* V600 VAF from ctDNA analysis of plasma samples collected at baseline and on treatment |
| **Tertiary/Exploratory** | |
| • To understand the relationship between the therapeutic intervention(s) being studied and the biology of the participant's disease | • Measurements of biomarkers, consisting of DNA, RNA, protein or defined cell types, resulting from analyses of peripheral blood and/or tumor tissue biospecimen obtained at baseline, on treatment and/or at end-of-study |
| • To understand the surgical conversion rate | • Surgical conversion rate, defined as the rate of participants who become eligible for surgery and undergo surgery with curative intent as a result of study intervention |
| **Cohort 3** | |
| **Objectives** | **Endpoints** |
| **Primary** | |
| • To compare the efficacy of EC + FOLFIRI (Arm D) vs FOLFIRI with or without bevacizumab (Control Arm [Arm E]) as measured by ORR | • ORR by BICR |
| **Key Secondary** | |
| • To further compare the efficacy of Arm D vs Arm E as measured by PFS | • PFS by BICR, defined as the time from the date of randomization to the earliest documented disease progression per RECIST v1.1, or death due to any cause |
| **Secondary (Descriptive Statistics Only)** | |
| • To further evaluate the efficacy of Arm D vs Arm E as measured by ORR, DOR, PFS, TTR and OS | • ORR by Investigator<br>• DOR by BICR and by Investigator, defined as the time from the date of first radiographic |



090177e1a02cb071\Approved\Approved On: 14-Mar-2024 15:31 (GMT)

| | |
|---|---|
| | evidence of response (CR or PR) to the earliest documented disease progression per RECIST v1.1, or death due to any cause<br>• PFS by Investigator, defined as the time from the date of randomization to the earliest documented disease progression per RECIST v1.1, or death due to any cause<br>• OS, defined as the time from the date of randomization to death due to any cause<br>• TTR (by BICR and by Investigator), defined as the time from the date of randomization to first radiographic evidence of response (CR or PR) per RECIST v1.1 |
| • To determine the safety and tolerability of EC + FOLFIRI | • Incidence and severity of AEs graded according to the NCI CTCAE v4.03 and changes in clinical laboratory parameters, vital signs, and ECGs |
| • To evaluate quality of life and health states, captured by PRO measures | • PRO scores as measured by the EORTC QLQ-C30, EQ-5D-5L, and anchoring instruments PGIS and PGIC |
| • To evaluate trough concentrations of encorafenib and its metabolite LHY746 in Arm D | • Trough plasma concentrations of encorafenib and the metabolite LHY746 in Arm D |
| • To confirm the MSI status in tumor tissue | • Summarize MSI-status as determined by retrospective central testing of baseline tumor tissue |
| • To determine the correlation between ctDNA levels, *BRAF* V600 alterations, and clinical outcome | • ctDNA levels and *BRAF* V600 VAF from ctDNA analysis of plasma samples collected at baseline and on treatment |
| **Tertiary/Exploratory** | |
| • To understand the relationship between the therapeutic intervention(s) being studied and the biology of the participant's disease | • Measurements of biomarkers, consisting of DNA, RNA, protein or defined cell types, resulting from analyses of peripheral blood and/or tumor tissue biospecimen obtained at baseline, on treatment and/or at end-of-study |
| • To understand the surgical conversion rate | • Surgical conversion rate, defined as the rate of participants who become eligible for surgery and undergo surgery with curative intent as a result of study intervention |

**Estimands**

The primary estimand in the SLI is the DLT rate estimated based on data from DLT-evaluable participants during the DLT-evaluation period, which is the first 28 days after the first dose of study intervention in the SLI. The number and proportion of participants experiencing DLTs during the DLT-evaluation period will be summarized for Cohort 1 (EC + FOLFIRI) and Cohort 2 (EC + mFOLFOX6), separately.

There are 2 primary estimands in the Phase 3 portion of the study:

090177e1a02cb071\Approved\Approved On: 14-Mar-2024 15:31 (GMT)

- The treatment effect in PFS by BICR, as measured by the hazard ratio. PFS is defined as the time from date of randomization to the earliest documented disease progression per RECIST v1.1, or death due to any cause. The hypothetical strategy will be applied for the intercurrent events. PFS will be compared between Arm B and the Control Arm using a 1-sided stratified log-rank test.

- The treatment effect of Arm B on OR based on BICR assessment per RECIST v1.1 compared to the Control Arm from randomization to the earliest of progression of disease, start of subsequent anticancer therapy, or death due to any cause. Both CR and PR must be confirmed by repeat assessments performed no less than 4 weeks after the criteria for response are first met. ORR comparison between the 2 treatment arms will be assessed using stratified Cochran-Mantel-Haenszel (CMH) test.

The primary estimand in Cohort 3 is the treatment effect of Arm D on OR based on BICR assessment per RECIST v1.1 compared to the Arm E from randomization to the earliest of progression of disease, start of subsequent anticancer therapy, or death due to any cause. Both CR and PR must be confirmed by repeat assessments performed no less than 4 weeks after the criteria for response are first met. ORR comparison between the 2 treatment arms will be assessed using stratified Cochran-Mantel-Haenszel (CMH) test.

A complete list of estimands is defined in Section 9.1.1.

# 4. STUDY DESIGN

## 4.1. Overall Design

This is an open-label, multicenter, randomized Phase 3 study of EC with or without chemotherapy versus standard-of-care chemotherapy in participants with previously untreated *BRAF* V600E-mutant mCRC. Prior to the Phase 3 portion, a SLI will be conducted at a limited number of sites to evaluate the safety/tolerability and PK of EC in combination with either FOLFIRI or mFOLFOX6 (Cohort 1 and Cohort 2, respectively). In addition to the Phase 3 portion, a Cohort 3 with 2 randomized arms will be included: EC in combination with FOLFIRI (Arm D) and FOLFIRI with or without bevacizumab (Arm E) in first-line participants. Cohort 3 will start after the enrollment of the Phase 3 portion is complete and enrollment will only be conducted in countries where authorized.

Approximately 60 participants will be enrolled in the SLI portion (up to 30 per regimen). The results of the SLI will inform which chemotherapy regimen is used in Arm B of the Phase 3 portion of the study. In the Phase 3 portion, approximately 620 participants will be randomized overall, initially at a ratio of 1:1:1 to receive EC (Arm A), EC + chemotherapy (Arm B) or SOC chemotherapy (Control Arm), and then 1:1 to receive EC + chemotherapy (Arm B) or SOC chemotherapy (Control Arm) after the approval of Protocol Amendment 5, with a total of approximately 150 participants for Arm A and 235 participants each for Arm B and the Control Arm (Arm C). Approximately 135 participants will be enrolled in Cohort 3 at a ratio of 2:1 to receive EC + FOLFIRI (Arm D, n=90) or FOLFIRI with or without bevacizumab (Arm E, n=45).



090177e1a02cb071\Approved\Approved On: 14-Mar-2024 15:31 (GMT)

The primary objective in the Phase 3 portion of the study is to compare the efficacy, as measured by the primary endpoints of PFS by BICR and ORR by BICR, of Arm B versus the Control Arm. The study will be considered positive (ie, demonstrated evidence of effectiveness) if the hypothesis test of either primary endpoint is statistically significant.

The primary objective in the Cohort 3 portion of the study is to compare the efficacy, as measured by the primary endpoint of ORR by BICR of Arm D versus Arm E. The results of Cohort 3 will be considered positive (ie, demonstrated evidence of effectiveness) if the hypothesis test of primary endpoint is statistically significant.

After reviewing the totality of the SLI data, the E-DMC and SC determined that there were no new safety signals and recommended that the study can proceed as planned. As a result of the SLI data review, Arm B will consist of EC in combination with mFOLFOX6. In addition, Phase 3 treatment Control Arm (Arm C) will consist of Investigator's choice of mFOLFOX6 ± bevacizumab, FOLFOXIRI ± bevacizumab or CAPOX ± bevacizumab.

## 4.2. Scientific Rationale for Study Design

### 4.2.1. Safety Lead-in

Since the EC regimen has not previously been combined with cytotoxic chemotherapy, a SLI will be conducted prior to the Phase 3 portion of the study to evaluate the safety/tolerability and PK of EC + mFOLFOX6 and EC + FOLFIRI. The results of the SLI will inform which chemotherapy regimen is used in Arm B of the Phase 3 portion of the study (see Section 6.1.1 for detailed description of SLI). The most frequently reported AEs (>25% of patients) among patients treated with EC in the BEACON CRC trial were nausea, diarrhea, fatigue, dermatitis acneiform, and decreased appetite. The potential overlapping toxicities between EC and the chemotherapy regimens are primarily GI toxicities that are managed routinely by treating physicians. Therefore, the safety and tolerability of the combinations will be assessed using the standard doses of all agents.

The SLI will enroll up to 30 participants per cohort to fully assess the safety/tolerability and PK, including the potential for DDI, of EC + mFOLFOX6 and EC + FOLFIRI. For each cohort, the probability of observing at least one instance of a toxicity in 30 participants is >95% when the true toxicity incidence rate is 10%. The decision to initiate Phase 3 will require approval from the study SC and the E-DMC. The E-DMC will also review safety data to confirm the tolerability of the EC + chemotherapy combination after the first 30 participants have been randomized and treated for at least 1 cycle in the Phase 3 portion of the study.

### 4.2.2. Assessment of Potential DDI with Encorafenib

Irinotecan is metabolized to SN-38, which is 100-1,000 times more potent than the parent. The exposures of both irinotecan and SN-38 have been shown to correlate with the efficacy and toxicity (eg, neutropenia and diarrhea) of irinotecan treatment.[51] SN-38 is conjugated by the enzyme UGT1A1 to form an inactive metabolite, SN-38G, which is readily eliminated.



090177e1a02cb071\Approved\Approved On: 14-Mar-2024 15:31 (GMT)

090177e1a02cb071\Approved\Approved On: 14-Mar-2024 15:31 (GMT)

An alternative pathway of irinotecan metabolism is CYP3A4-mediated oxidation, which produces inactive metabolites. Thus, the PK of irinotecan and SN-38 can be altered when co-administered with CYP3A4 inducers or inhibitors.[52] Therefore, strong CYP3A4 inducers or inhibitors should not be co-administered with irinotecan unless there are no therapeutic alternatives.[53]

In vitro studies indicated that encorafenib is a reversible inhibitor of UGT1A1. Based on recently available data from an ongoing DDI study in patients, single dose encorafenib does not inhibit CYP3A at clinically used doses. However, in patients receiving therapeutic doses, encorafenib plasma exposures at steady-state are lower than expected, indicating net auto-induction following multiple doses. Encorafenib also inhibits multiple transporters that are also involved in disposition and elimination of irinotecan and its metabolites (SN-38 and SN-38G).[54,55] Therefore, when co-administrated with FOLFIRI, encorafenib has the potential to alter the PK of irinotecan or SN-38 via changes in enzyme activities of CYP3A4, UGT1A1 and various transporters. The metabolism of oxaliplatin, 5-FU, and leucovorin is not mediated by CYP450 enzymes, and the risk of CYP450-mediated DDI of encorafenib with these agents is expected to be low.[56-58]

The potential for a drug-interaction between encorafenib and irinotecan has now been characterized and indicates that encorafenib decreases irinotecan and SN-38 concentrations by approximately 25% at steady state.[59] Additionally, no increase in the rate of AEs due to higher plasma exposure of irinotecan or SN-38 is anticipated at the initiation of combination treatment (Cycle 1 Day 1). Based on the data from the ongoing DDI study, encorafenib and irinotecan can be co-administered from the initiation of combination treatment.

### 4.2.3. Investigator's Choice of Control Regimen

Treatment of investigator's choice was chosen as the comparator in the Phase 3 portion of the study because a number of therapeutic options are available as initial therapy for mCRC, allowing selection of the regimen believed to potentially confer the greatest clinical benefit to a given participant. Currently, no treatments are indicated specifically for patients with *BRAF* V600E-mutant mCRC in the first-line setting and there is no single SOC regimen established in this indication (see Table 6).

Clinical guidelines established by ASCO, ESMO, and JSMO suggest that mFOLFOX6, FOLFIRI, or CAPOX are all acceptable therapeutic options and should be combined with bevacizumab where possible for initial treatment of patients with mCRC irrespective of tumor *BRAF* status.[28,35,60,61] The more intensive combination of FOLFOXIRI + bevacizumab was also shown to be active based on retrospective analyses of clinical trial data although more recent data from a meta-analysis suggests that the triple combination of 5-FU, oxaliplatin, and irinotecan (with bevacizumab) has similar efficacy as doublet chemotherapy and bevacizumab in patients with *BRAF* V600E-mutant mCRC.[62] Since the FOLFOXIRI plus bevacizumab regimen is associated with greater toxicity, it is only recommended for patients who are defined as being fit and highly motivated in the ESMO guidelines or those with excellent performance status.

### 4.2.4. Progression-Free Survival and Objective Response Rate as Primary Endpoints

The primary endpoints will be PFS by BICR and ORR by BICR, and OS will be the key secondary endpoint for the Phase 3 portion. ORR by BICR will be the primary endpoint and PFS by BICR will be the key secondary endpoint for Cohort 3. Given the study design and nature of the disease, it is likely that at the time of the primary endpoint (ie, ORR, PFS) analysis, OS may still be immature, and significance may not have been observed. PFS as a primary endpoint with OS as a key secondary endpoint is commonly used in oncology trials including trials in first-line mCRC.[19,63]

According to the Dec 2018 FDA guidance on 'Clinical Trial Endpoints for the Approval of Cancer Drugs and Biologics,' a large improvement in PFS can be used to support traditional approval in select indications.[64] The EMA CHMP Guideline on the 'Evaluation of Anticancer Medicinal Products in Man' states that OS and PFS are acceptable endpoints, and recommends that if PFS is selected as the primary endpoint, OS should be used as a secondary endpoint.[65]

This is an open-label study, therefore the primary endpoints of PFS and ORR in the Phase 3 portion will be determined by BICR to minimize evaluation bias. An E-DMC will review data throughout the trial. An E-DMC Charter that outlines E-DMC membership, the data that will be reviewed and the timing and frequency of the reviews will be established. Because the assessment of PFS for determination of the primary endpoint will be based on a BICR, disease progression will be confirmed by expedited central review to minimize informative censoring.

In addition, because spontaneous remission is rare, ORR provides clear evidence of antitumor activity. ORR could be evaluated earlier than PFS and could be used for an accelerated/conditional approval if clinically meaningful and durable ORR is shown. Thus, the primary endpoint of ORR has been added to the Phase 3 portion of the study and will be the only primary endpoint for Cohort 3, defined as the proportion of participants with confirmed CR or PR according to RECIST v1.1 as assessed by BICR.

### 4.2.5. Biomarkers

The biomarker analyses applied to pre-treatment, on-treatment (blood only) and end of treatment tumor and/or blood biospecimens are intended to evaluate established and candidate prognostic/predictive biomarkers that may identify participants who preferentially benefit from the study treatments and to evaluate mechanisms of action and/or resistance for each of the combinations being assessed.

*BRAF* and *KRAS* mutations are associated with poor prognosis in advanced CRC and are almost always mutually exclusive.[10,66,67] The predominant (>95%) *BRAF* mutation observed in CRC patients is *BRAF* V600E, which leads to constitutive activation of BRAF kinase and sustained RAS/RAF/MEK/ERK pathway signaling, resulting in increased cell proliferation and survival.[16] In mCRC, approximately 5% of patients are MSI-H, compared to 15% in primary CRC.[16,68] Approximately 15-30% of *BRAF*-mutant mCRC tumors were also identified as MSI-H/dMMR.[14,16,24,25] The presence of a *BRAF* V600 mutation appears to be a



090177e1a02cb071\Approved\Approved On: 14-Mar-2024 15:31 (GMT)

poor prognostic factor independent of MSI status in mCRC,[14,16] which differs from the MSS-dependent poor prognostic value of *BRAF* V600 in primary CRC.[69] In this study, MSI status will be confirmed by retrospective central testing in tumor tissue as a secondary endpoint.

Detection of *BRAF* V600E in ctDNA is concordant with tumor tissue in CRC patients.[70] A decrease in *BRAF* V600E ctDNA levels predicted improved radiographic responses to vemurafenib + cetuximab + irinotecan[71] and was significantly greater in responders compared to non-responders in *BRAF*-mutant CRC patients treated with dabrafenib + panitumumab ± trametinib.[72] Assessments of ctDNA levels as a surrogate marker for tumor burden and of *BRAF* V600 VAF in ctDNA from plasma samples collected at baseline and on treatment will be secondary endpoints in this study based on recent findings from the ctMoniTR Project and subsequent studies.[73,74]

Unless prohibited by local regulations or ethics committee decision, additional blood samples will be collected for various exploratory biomarker analyses as described in Section 8. Banked Biospecimens will be collected and stored for further analyses which may, for example, provide greater understanding of the study intervention.

### 4.2.6. Use of Contraceptives

The study interventions being administered are known to cause risk for severe manifestations of developmental toxicity in humans or suspected on the basis of the intended pharmacology. Therefore, the use of a highly effective method of contraception is required (see Section 5.3.1 and Appendix 4).

## 4.3. Justification for Dose

In order to optimize the potential for benefit, while limiting the potential for toxicity, the starting encorafenib dose in the current study is 300 mg QD, corresponding to its single-agent RP2D. This dose of encorafenib was administered in combination with cetuximab in Study ARRAY-818-302 (BEACON CRC) and was shown to be both effective and tolerable in participants with previously treated *BRAF* V600E-mutant mCRC. Based on these results, the approved dose of encorafenib for this indication is 300 mg QD when given in combination with cetuximab.[37] Further details are available in the encorafenib SRSD.

The dose regimens selected for capecitabine, 5-FU, irinotecan, leucovorin, oxaliplatin, and bevacizumab are consistent with the respective SRSDs.

The cetuximab regimen approved globally is 400 mg/m$^2$ first-dose followed by 250 mg/m$^2$ IV weekly. Because many of the cytotoxic doublets used in mCRC are administered biweekly, a biweekly cetuximab regimen has also been tested. In a Phase 1 dose-escalation study in patients with mCRC, doses of cetuximab 400 to 700 mg/m$^2$ every 2 weeks were evaluated. The MTD was not identified and 500 mg/m$^2$ was identified as the dose for further development based on its PK parameters being most similar to the weekly regimen.[75] A Phase 2 study in patients with previously treated mCRC treated with biweekly cetuximab 500 mg/m$^2$ in combination with irinotecan demonstrated similar efficacy and tolerability as historical data with similar regimens using weekly cetuximab.[76] The use of the biweekly



090177e1a02cb071\Approved\Approved On: 14-Mar-2024 15:31 (GMT)

regimen is fairly common in current practice and is included as an acceptable regimen in the NCCN guidelines.[28] Allowing use of this regimen will provide greater flexibility and perhaps enhance compliance and recruitment within the study in which participants are expected to remain on treatment for approximately 10 months.

## 4.4. End of Study Definition

The end of study is defined as LPLV. LPLV is defined as the date of the last visit or follow-up contact of the last participant in the study globally.

# 5. STUDY POPULATION

This study can fulfill its objectives only if appropriate participants are enrolled. The following eligibility criteria are designed to select participants for whom participation in the study is considered appropriate. All relevant medical and nonmedical conditions should be taken into consideration when deciding whether a particular participant is suitable for this protocol.

Prospective approval of protocol deviations to recruitment and enrollment criteria, also known as protocol waivers or exemptions, is not permitted.

Pfizer or designee will review select eligibility criteria verified by the investigator or qualified designee to confirm that participants meet study eligibility criteria before they are enrolled into the study. The enrollment approval process will be initiated for a participant after an informed consent/assent document has been signed and the investigator or qualified designee has assessed the participant as eligible. The enrollment approval will be based on review of CRF/system data.

## 5.1. Inclusion Criteria

Participants are eligible to be included in the study only if all of the following criteria apply:

### 5.1.1. Molecular Prescreening Inclusion Criteria

**Age and Sex:**

1. **SLI**: Male or female participants age ≥18 years at the time of informed consent. **Phase 3 and Cohort 3**: Male or female participants age ≥16 years at the time of informed consent/assent in all countries where permitted. In countries or sites where enrollment of adolescents is not permitted (eg, Germany), male or female participants age ≥18 years at the time of informed consent.
   - Refer to Appendix 4 for reproductive criteria for male (Section 10.4.1) and female (Section 10.4.2) participants.

**Weight:**

2. Body weight ≥40 kg.

090177e1a02cb071\Approved\Approved On: 14-Mar-2024 15:31 (GMT)

**Type of Participant and Disease Characteristics:**

3. Participants with histologically or cytologically confirmed colorectal adenocarcinoma.

4. Participants with evidence of Stage IV metastatic disease.
   ***Note***: Patients with oligometastatic disease previously treated with curative intent are eligible to participate in the study as long as they have baseline measurable disease per RECIST 1.1. Oligometastatic colorectal cancer is characterized by a limited metastatic spread of disease. Oligometastatic disease is defined as the involvement of up to 3 sites with 5 or sometimes more metastases that for their anatomic localization is amenable to local therapies, thus rendering the patient free of disease.[77]

5. Able to provide a sufficient amount of representative tumor specimen for central testing of *BRAF* V600E mutation status (see Section 8.7.2) and tumor tissue assessment (see Section 8.8.2).
   ***Note***: Tumor sample can be archival or de novo (newly collected fixed biopsy sample) and must be in an FFPE block, or provide a minimum of 15 (a minimum of 10 for mainland China) unstained slides of analyzable tissue. This tissue specimen should be obtained from a biopsy or surgery that was performed within 2 years prior to study enrollment. Participants with fewer than the required number of slides with analyzable tissue may be considered eligible if the Sponsor determines that the slides are sufficient for central testing.

**Informed Consent/Assent:**

6. Capable of giving signed informed consent/assent as described in Appendix 1, which includes compliance with the requirements and restrictions listed in the ICD and in this protocol.
   ***Note***: Participants ≥16 years old that are under guardianship may participate with the consent of their legally authorized guardian if permitted by local regulations. When appropriate, adolescent participants will be included in all discussions (see Section 10.1.3).

### 5.1.2. Screening Inclusion Criteria

**Type of Participant and Disease Characteristics:**

7. Participants who have met all Molecular Prescreening inclusion criteria.

8. Participants who are willing and able to comply with all scheduled visits, treatment plan, laboratory tests, lifestyle considerations, and other study procedures.

9. Presence of a *BRAF* V600E mutation in tumor tissue or blood (eg, ctDNA genetic testing). The following are acceptable:

   a. Local laboratory assay (PCR or NGS-based only) performed at any time prior to Screening using either tumor tissue or blood.

090177e1a02cb071\Approved\Approved On: 14-Mar-2024 15:31 (GMT)

b. Central laboratory assay performed during the Screening period using tumor tissue alone (not blood).

***Note***: For participants enrolled on the basis of a local *BRAF* mutation assay, tumor samples must be submitted to the central laboratory for *BRAF* testing as soon as possible following signing of the ICD. The *BRAF* status must be confirmed no later than 30 days following first dose of study intervention.

10. The Investigator must obtain prior to Cycle 1 Day 1 (SLI) or date of randomization (Phase 3 and Cohort 3) adequate tumor tissue (primary or metastatic, archival or newly obtained) for submission to a central laboratory for confirmation of *BRAF* V600E (see Section 8.7.2) and tumor tissue assessment (see Section 8.8.2). Sample must be an FFPE block or minimum of 15 (a minimum of 10 for mainland China) unstained slides containing recently cut, 4-5 µm sections on positively charged slides. This tissue specimen should be obtained from a biopsy or surgery that was performed within 2 years prior to study enrollment. Participants with fewer than the required number of slides with analyzable tissue may be considered eligible if the Sponsor determines that the slides are sufficient for central testing.
***Note***: Once *BRAF* V600E mutation status is determined by the central laboratory (tumor tissue), the results will be considered definitive for eligibility. No repeat testing will be performed.
***Note***: Lack of *BRAF* V600E confirmation by the central laboratory may be due to discordance between the local assay and central laboratory results (potential false positive local assay results), or due to inadequate or poor sample condition for central testing (indeterminate results). See Section 8.7.2 for additional information.
***Note***: Participants whose sample is determined to be inadequate or who have an indeterminate result on central testing may have additional tumor samples submitted for testing.

11. Participants who have received ≤1 (SLI) or no (Phase 3 and Cohort 3) prior systemic regimen(s) for metastatic disease.
***Note***: SLI participants who have received prior oxaliplatin-based regimens in first-line treatment will be enrolled to the FOLFIRI combination cohort (Cohort 1) and participants who have received prior irinotecan in first-line treatment will be enrolled in the mFOLFOX6 combination cohort (Cohort 2).
***Note***: Participants with early stage disease (eg, Stages I-III) treated with surgery followed by chemotherapy (eg, treatment in the adjuvant setting) or have received prior systemic neoadjuvant therapy with or without radiation who present with new lesions or evidence of disease recurrence during or within 6 months of the last dose of chemotherapy would be considered as having received 1 prior systemic therapy in the metastatic setting.

12. ECOG performance status of 0 or 1.

13. Measurable disease in the Phase 3 and Cohort 3 portions of the study and measurable or non-measurable but evaluable disease per RECIST, v1.1 in the SLI, as assessed by Investigator and evidenced by available baseline scans.



090177e1a02cb071\Approved\Approved On: 14-Mar-2024 15:31 (GMT)

***Note***: Baseline scan is defined as the last scan prior to the date of first dose (SLI ) or the date of randomization (Phase 3 and Cohort 3) (Section 9.4.1).
***Note***: Baseline scans will be required to be submitted to a central radiology vendor to be assessed by the BICR.

14. Adequate bone marrow function characterized by the following at screening:
    a. ANC ≥1.5 × $10^9$/L;
    b. Platelets ≥100 × $10^9$/L;
    c. Hemoglobin ≥9.0 g/dL (with or without blood transfusions).
15. Adequate hepatic and renal function characterized by the following at screening:
    a. Serum total bilirubin ≤1.5 x ULN and <2 mg/dL.
    ***Note***: Total bilirubin >1.5 x ULN is allowed if direct (conjugated) ≤1.5 x ULN and indirect (unconjugated) bilirubin is ≤4.25 x ULN.
    ***Note***: Participants with hyperbilirubinemia due to non-hepatic cause (eg, hemolysis, hematoma) may be enrolled following discussion and agreement with the medical monitor.
    b. ALT and AST ≤2.5 × ULN, or ≤5 × ULN in the presence of liver metastases.
    c. Adequate renal function defined by an estimated creatinine clearance ≥50 mL/min according to the Cockcroft Gault formula or by 24-hour urine collection for creatinine clearance, or according to local institutional standard method.
    d. Adequate electrolytes, defined as serum potassium and magnesium levels within institutional normal limits.
    ***Note***: Replacement treatment to achieve adequate electrolytes will be allowed.
16. Able to swallow, retain, and absorb oral medications.

## 5.2. Exclusion Criteria

Participants are excluded from the study if any of the following criteria apply:

### 5.2.1. Molecular Prescreening Exclusion Criteria

**Medical Conditions:**

1. Other medical or psychiatric condition including recent (within the past year) or active suicidal ideation/behavior or laboratory abnormality that may increase the risk of study participation or, in the investigator's judgment, make the participant inappropriate for the study.
2. Presence of acute or chronic pancreatitis.
3. Leptomeningeal disease.



090177e1a02cb071\Approved\Approved On: 14-Mar-2024 15:31 (GMT)

4. History of chronic inflammatory bowel disease requiring medical intervention (immunomodulatory or immunosuppressive medications or surgery) ≤12 months prior to randomization.
5. Known DPD deficiency; refer to local fluorouracil or capecitabine label or local clinical guidance, for DPD status recommendation prior to starting treatment.
6. Gilbert's syndrome or known homozygous UGT1A1*28/*28 or UGT1A1*6/*6 genotypes or double heterozygous UGT1A1*6/*28 genotype:
   a. **SLI**: Participants with documented Gilbert's syndrome or known homozygous UGT1A1*28/*28 or UGT1A1*6/*6 genotypes or double heterozygous UGT1A1*6/*28 genotype will be excluded from Cohort 1 (EC + FOLFIRI) of the SLI.
   b. **Phase 3**: Participants with documented Gilbert's syndrome or known homozygous UGT1A1*28/*28 or UGT1A1*6/*6 genotypes or double heterozygous UGT1A1*6/*28 genotype may be enrolled, but may not receive FOLFOXIRI if randomized to the Control Arm.
   c. **Cohort 3:** Participants with documented Gilbert's syndrome or known homozygous UGT1A1*28/*28 or UGT1A1*6/*6 genotypes or double heterozygous UGT1A1*6/*28 genotype will be excluded from Cohort 3 Arm D and Arm E (EC + FOLFIRI and FOLFIRI ± bevacizumab).

**Other Exclusions:**

7. Investigator site staff or Pfizer employees directly involved in the conduct of the study, site staff otherwise supervised by the investigator, and their respective family members.
8. Colorectal adenocarcinoma that is RAS mutant or for which RAS mutation status is unknown.
9. Locally confirmed dMMR or MSI-H colorectal carcinoma or unknown MSI/MMR status. If participant is locally confirmed dMMR or MSI-H and unable to receive immune checkpoint inhibitors due to a pre-existing medical condition, they may be enrolled.

### 5.2.2. Screening Exclusion Criteria

**Medical Conditions:**

10. Impaired gastrointestinal function (eg, uncontrolled nausea, vomiting or diarrhea, malabsorption syndrome, small bowel resection) or disease which may significantly alter the absorption of oral study intervention or recent changes in bowel function suggesting current or impending bowel obstruction.
11. Clinically significant cardiovascular diseases, including any of the following:

090177e1a02cb071\Approved\Approved On: 14-Mar-2024 15:31 (GMT)

a. History of acute myocardial infarction, acute coronary syndromes (including unstable angina, coronary artery bypass graft, coronary angioplasty or stenting) ≤6 months prior to randomization;

b. Congestive heart failure requiring treatment (New York Heart Association Class II and above);

c. Recent history (within 1 year prior to randomization) or presence of clinically significant cardiac arrhythmias (including uncontrolled atrial fibrillation or uncontrolled paroxysmal supraventricular tachycardia);

d. History of thromboembolic or cerebrovascular events ≤12 weeks prior to randomization. Examples include transient ischemic attacks, cerebrovascular accidents, hemodynamically significant (ie, massive or sub-massive) deep vein thrombosis or pulmonary emboli.
***Note***: Participants with either deep vein thrombosis or pulmonary emboli that do not result in hemodynamic instability are allowed to enroll as long as they are on a stable dose of anticoagulants for at least 4 weeks.
***Note***: Participants with thromboembolic events related to indwelling catheters (including PICC lines) or other procedures may be enrolled.

e. Triplicate average QTcF interval ≥480 ms or a history of prolonged QT syndrome.

   ***Note:*** Participants with bundle-branch block (BBB) or with an implanted cardiac pacemaker, may enroll into the study following consultation with the Sponsor.

f. Congenital LQTS.

12. Evidence of active noninfectious pneumonitis.

13. Evidence of active and uncontrolled bacterial or viral infection, with certain exceptions, as noted below, for chronic infection with HIV, hepatitis B or hepatitis C (please see below), within 2 weeks prior to start of study intervention.

14. Participants positive for HIV are ineligible unless they meet all of the following:

    a. A stable regimen of highly active anti-retroviral therapy that is not contraindicated (see Section 6.5);

    b. No requirement for concurrent antibiotics or antifungal agents for the prevention of opportunistic infections;

    c. A CD4 count >250 cells/mcL, and an undetectable HIV viral load on standard PCR-based tests.

15. Active hepatitis B or hepatitis C infection

    a. Active HBV is defined as any of the following:

    - HBsAg(+), HBV DNA >200 IU/mL;



090177e1a02cb071\Approved\Approved On: 14-Mar-2024 15:31 (GMT)

- HBsAg(+), HBV DNA ≤200 IU/mL and persistent or intermittent elevation of ALT/AST (ie, above normal range) and/or liver biopsy showing chronic hepatitis with moderate or severe necroinflammation.

***Note***: Participants who are HBsAg(-), HBcAb(+) are eligible and should be monitored/treated as per local standard of care.

b. Active HCV is defined as:

- HCV antibody positive; AND
- Presence of HCV RNA.

16. Concurrent or previous other malignancy within 2 years of study entry, except curatively treated basal or squamous cell skin cancer, prostate intraepithelial neoplasm, carcinoma in-situ of the cervix, Bowen's disease or prostate cancer with a Gleason score ≤6. Participants with other curatively treated malignancies with low risk of recurrence not listed may also be considered eligible after review and approval by the medical monitor.
17. Residual CTCAE ≥Grade 2 toxicity from any prior anticancer therapy, with the exception of Grade 2 alopecia or Grade 2 neuropathy.

***Note***: Participants with ≥ Grade 2 neurotoxicity are excluded from receiving mFOLFOX 6 in the SLI, and entirely from Phase 3.

**Prior/Concomitant Therapy:**

18. **SLI**: Treatment with any of the following:
    - Prior systemic regimen containing both oxaliplatin and irinotecan (eg, FOLFOXIRI).
    - Cyclical chemotherapy within a period of time that was shorter than the cycle length used for that treatment (e.g., 6 weeks for nitrosourea, mitomycin-C) prior to starting study treatment.
    - Biologic therapy (e.g., antibodies) except bevacizumab or aflibercept, continuous or intermittent small molecule therapeutics, or any other investigational agents within a period of time that is ≤ 5 half-lives ($t_{1/2}$) or ≤ 4 weeks (whichever is shorter) prior to starting study treatment.
    - Bevacizumab or aflibercept therapy ≤ 3 weeks prior to starting study treatment.
19. Previous treatment with any selective BRAF inhibitor (eg, encorafenib, dabrafenib, vemurafenib, XL281/BMS-908662) or any EGFR inhibitor (eg, cetuximab, panitumumab) prior to screening.
20. Use of any prohibited medication (including herbal medication), supplement or food that is a moderate or strong inhibitor or inducer of CYP3A4/5 ≤1 week prior to the start of study intervention (Section 6.5.1.2).



090177e1a02cb071\Approved\Approved On: 14-Mar-2024 15:31 (GMT)

21. Major surgery or completion of radiation therapy ≤4 weeks prior to enrollment/randomization or radiation therapy that included >30% of the bone marrow.

**Prior/Concurrent Clinical Study Experience:**

22. Previous administration with an investigational product (eg, drug or vaccine) within 30 days (or as determined by the local requirement) or 5 half-lives preceding the first dose of study intervention used in this study (whichever is longer).

**Other Exclusions:**

23. Has met any of the Molecular Prescreening exclusion criteria.
24. Has documented clinical disease progression (eg, worsening of performance status, clinical symptoms, or clinically significant laboratory parameters demonstrating worsening of disease) or radiographic disease progression during the Screening period.
25. Symptomatic brain metastasis.
    ***Note***: Participants previously treated or untreated for this condition who are asymptomatic in the absence of corticosteroid and anti-epileptic therapy are allowed. Brain metastases must be stable for ≥4 weeks prior to randomization.
26. Known contraindication to receiving cetuximab including hypersensitivity or toxicity that would suggest an inability to tolerate maximum cetuximab dose of 500 $mg/m^2$.
27. Known sensitivity or contraindication to any component of study intervention or their excipients at the planned doses.
28. Known contraindication to receive any component of the Control Arm at the planned doses; refer to the most recent local label, as applicable.
29. Pregnant, confirmed by a positive β-hCG laboratory test result, or is breastfeeding (lactating).

## 5.3. Lifestyle Considerations

The investigator or his or her designee, will discuss with the participant fertility preservation strategies prior to the start of study intervention.

### 5.3.1. Contraception

Due to the potential of encorafenib to induce CYP3A4 (see Section 6.5.1.2), hormonal agents (including but not limited to birth control patch, vaginal ring, oral, injectable, or implanted contraceptives) are permissible for participants treated in the SLI, Arm A, Arm B and Cohort 3 Arm D only when combined with other highly effective or acceptable methods. The investigator or his or her designee, in consultation with the participant, will confirm that the participant has selected an appropriate method of contraception for the individual participant and his or her partner(s) from the permitted list of contraception methods (see Appendix 4

090177e1a02cb071\Approved\Approved On: 14-Mar-2024 15:31 (GMT)

Section 10.4.4) and will confirm that the participant has been instructed in its consistent and correct use. At time points indicated in the SoA, the investigator or designee will inform the participant of the need to use highly effective contraception consistently and correctly and document the conversation and the participant's affirmation in the participant's chart (participants need to affirm their consistent and correct use of at least 1 of the selected methods of contraception). It is recommended that participants be followed for contraception use for 9 months for females and 6 months for males following the last dose of study intervention. Appropriate tests can also be carried out at home, so that monitoring of successful contraception can be guaranteed even if the participants no longer come to the study site regularly. In addition, the investigator or designee will instruct the participant to call immediately if the selected contraception method is discontinued or if pregnancy is known or suspected in the participant or partner.

### 5.3.2. Meals and Dietary Restrictions

Participants treated in the SLI, Arm A, Arm B and Arm D must avoid consumption of grapefruit, pomegranates, star fruits, Seville oranges or products containing the juice of each during the entire study and preferably 7 days before the first dose of study intervention, due to potential CYP3A4 interaction with encorafenib (see Section 6.5). Orange juice is allowed.

### 5.3.3. Photosensitivity

Participants should avoid extended exposure to ultraviolet light and when outdoors, should wear occlusive clothing, sunscreen and sunglasses when receiving 5-FU.

### 5.3.4. Screen Failures

Screen failures are defined as participants who consent/assent to participate in the clinical study but are not subsequently enrolled in the study. A minimal set of screen failure information is required to ensure transparent reporting of screen failure participants to meet the CONSORT publishing requirements and to respond to queries from regulatory authorities. Minimal information includes demography, screen failure details, eligibility criteria, and any SAEs. For Molecular Prescreening failures, results of local *BRAF* assay available at Molecular Prescreening or Screening, AEs or SAEs possibly related to a study procedure will be reported.

Tests with results that fail eligibility requirements may be repeated during Screening if the Investigator believes the result to be in error. Additionally, a participant who fails Screening (screen failure) may repeat the Screening process 1 time if the Investigator believes that there has been a change in eligibility status.

## 6. STUDY INTERVENTION

Study intervention is defined as any investigational intervention(s), marketed product(s), placebo, medical device(s), or study procedure(s) intended to be administered to a study participant according to the study protocol.

090177e1a02cb071\Approved\Approved On: 14-Mar-2024 15:31 (GMT)

For the purposes of this protocol, study intervention refers to capecitabine, cetuximab, encorafenib, 5-FU, irinotecan, leucovorin, oxaliplatin, and bevacizumab.

Investigators should refer to the approved local label of the products used in this trial (capecitabine, cetuximab, 5-FU, irinotecan, leucovorin, oxaliplatin, and bevacizumab) for the management of patients, especially concerning contraindications, duration of contraception, special warnings and precautions, posology adaptation in case of toxicity, monitoring, as well as medications that are contraindicated or that must be used with caution.

090177e1a02cb071\Approved\Approved On: 14-Mar-2024 15:31 (GMT)

090177e1a02cb071\Approved\Approved On: 14-Mar-2024 15:31 (GMT)

## 6.1. Study Interventions Administered

| Intervention Name | Encorafenib | Cetuximab | Fluorouracil | Leucovorin[a] | Irinotecan | Oxaliplatin | Capecitabine | Bevacizumab |
|---|---|---|---|---|---|---|---|---|
| **Treatment Arm** | SLI; Phase 3, Arm A and Arm B; Cohort 3 Arm D | SLI; Phase 3, Arm A and Arm B; Cohort 3 Arm D | SLI; Phase 3, Arm B; Phase 3, Control Arm; Cohort 3 | SLI; Phase 3, Arm B; Phase 3, Control Arm; Cohort 3 | SLI; Phase 3, Control Arm; Cohorts 3 | SLI; Phase 3, Arm B[b]; Phase 3, Control Arm C | Phase 3, Control Arm C | Phase 3, Control Arm C; Cohort 3 Arm E |
| **Type** | Drug | Drug | Drug | Drug | Drug | Drug | Drug | Drug |
| **Dose Formulation** | Capsule | Injection for intravenous use | Injection for intravenous use | Injection | Solution for intravenous infusion | Concentrate for solution for intravenous use | Tablet | Injection for intravenous use |
| **Unit Dose Strength(s)** | 75 mg | 100 mg/vial, 200 mg/vial, or 500 mg/vial | 250 mg/vial, 500 mg/vial, or 1000 mg/vial | 50 mg/vial, 100 mg/vial, 200 mg/vial, or 350 mg/vial | 40 mg/vial, 100 mg/vial, or 300 mg/vial | 50 mg/vial, 100 mg/vial, or 200 mg/vial | 150 mg or 500 mg | 100 mg/vial or 400 mg/vial |
| **Route of Administration** | Oral | Intravenous | Intravenous | Intravenous | Intravenous | Intravenous | Oral | Intravenous |
| **Use** | Experimental | Experimental | Experimental (Arms A and B); Comparator (Control Arm) | Experimental (Arms A and B); Comparator (Control Arm) | Experimental (Arms A and B); Comparator (Control Arm) | Experimental (Arms A and B); Comparator (Control Arm) | Comparator | Comparator |
| **IMP or NIMP/AxMP** | IMP | IMP or NIMP (USA only) | IMP or NIMP (USA only) | IMP or NIMP (USA only) | IMP or NIMP (USA only) | IMP or NIMP (USA only) | IMP or NIMP (USA only) | IMP or NIMP (USA only) |
| **Sourcing** | Provided centrally by the sponsor. | Provided centrally by the sponsor or locally by the trial site. | Provided centrally by the sponsor or locally by the trial site. | Provided centrally by the sponsor or locally by the trial site. | Provided centrally by the sponsor or locally by the trial site. | Provided centrally by the sponsor or locally by the trial site. | Provided centrally by the sponsor or locally by the trial site. | Provided centrally by the sponsor or locally by the trial site. |
| **Packaging and Labeling** | Study intervention will be provided in bottles. Each bottle will be open labeled as required per country requirement. | If IMP study intervention will be provided in vials. Each vial will be open labeled as required per country requirement. | If IMP study intervention will be provided in vials. Each vial will be open labeled as required per country requirement. | If IMP study intervention will be provided in vials. Each vial will be open labeled as required per country requirement. | If IMP study intervention will be provided in vials. Each vial will be open labeled as required per country requirement. | If IMP study intervention will be provided in vials. Each vial will be open labeled as required per country requirement. | If IMP study intervention will be provided in bottles. Each bottle will be open labeled as required per country requirement. | If IMP study intervention will be provided in vials. Each vial will be open labeled as required per country requirement. |
| **SRSD** | IB | EU SmPC | UK SmPC | EU/UK SmPC[c] | EU SmPC | EU SmPC | EU SmPC | EU SmPC |

a Alternatively, levo-leucovorin or calcium folinate (100 mg/vial, 200 mg/vial, or 300 mg/vial) may be supplied. All three presentations are 10 mg/mL strength.
b Based on the results of the SLI, oxaliplatin will be used in Arm B (see Section 4.1).
c SRSD for leucovorin (calcium folinate) is the EU SmPC and SRSD for levo-leucovorin is the UK SmPC.

### 6.1.1. Safety Lead-in

Since the EC regimen has not previously been combined with cytotoxic chemotherapy, the study includes an SLI, to be conducted at a limited number of sites, to evaluate the safety/tolerability and PK of EC + mFOLFOX6 and EC + FOLFIRI in up to 30 participants per cohort. Participants will be enrolled on a rolling basis in an alternating manner to each cohort when possible based on eligibility criteria.

The tolerability assessment will be based on occurrence of DLTs as defined in Section 6.1.1.1. Tolerability will be reviewed by the Sponsor and investigators in approximately weekly telephone conferences during the SLI. If 3 of the first 9 participants are determined to have experienced DLTs in a given cohort, enrollment to that cohort will be put on hold until a discussion with the SC and E-DMC can occur. If the DLTs are confirmed by the SC and the E-DMC and it is determined that the regimen is not tolerable, dosing in that cohort will be suspended.

The SC will review safety data after the 9$^{th}$ evaluable participant in a particular cohort has been followed for at least one 28-day cycle. In either cohort, if the doses are determined to be tolerable by the SC in the first 9 evaluable participants based on an observed rate of DLTs in <33% of participants and evaluation of the overall toxicity profile, the SLI will be expanded for that particular cohort by up to a maximum of approximately 30 participants. Enrollment may continue while the SC reviews safety data from the first 9 participants. The E-DMC will also review data after the 9$^{th}$ participant in a particular cohort has been followed for at least one 28-day cycle to confirm tolerability of the doses.

At the completion of the SLI when all evaluable participants have been followed for a minimum of one 28-day cycle, the SC and the E-DMC will review all accumulated safety data to confirm that the doses are acceptable for use in Arm B (EC + mFOLFOX6 or EC + FOLFIRI) of the randomized Phase 3 portion of the study. The primary criteria in deciding which combination to use in Phase 3 will be based on identifying the combination with the fewest DLTs. If the DLT rate is ≥33% in either cohort, that regimen will not be used in Phase 3. If the DLT rate is <33% in both cohorts, additional parameters that may be used to determine the most suitable combination will include dose modifications, AEs of at least Grade 3, AEs leading to treatment discontinuations, SAEs, and PK results including the potential for DDIs (Section 4.2.2). Since only 1 of the combination regimens will be included in the Phase 3 portion of the study, a rigorous selection process will be implemented based on the totality of the data. Finally, although efficacy is not a primary objective, the number of objective responses and DORs may also be taken into consideration in making a final decision.

Although the SLI is planned to enroll up to 30 evaluable participants per cohort, the decision to proceed to Phase 3 may occur earlier if one of the combination regimens is clearly superior based on the collective safety/tolerability and PK. If there are no clearly distinguishing differences between the 2 combinations, the most appropriate regimen to be used in Phase 3 will be determined following a review of the totality of the SLI data. If neither regimen is considered tolerable and/or suitable for evaluation in the randomization portion of the trial,



090177e1a02cb071\Approved\Approved On: 14-Mar-2024 15:31 (GMT)

the decision to proceed as a 2-arm study of EC versus SOC chemotherapy will be made prior to start of randomization in the Phase 3 portion of the study. The decision to initiate Phase 3 will require approval from the study SC and the E-DMC.

#### 6.1.1.1. Dose Limiting Toxicity Definition

For purposes of tolerability decisions, a DLT is defined as any AE or abnormal laboratory value assessed as unrelated to disease, disease progression, intercurrent illness or concomitant medications/therapies occurring during the first 28 days of treatment that meets at least 1 of the criteria listed in Table 9. A participant is classified as DLT-evaluable if he/she experiences a DLT or in the absence of a DLT, receives at least 75 % of the planned dose intensity of each study intervention during the DLT window (see Section 9.3). If a participant does not meet these criteria, he/she may be replaced.

Adverse events that are clinically significant and considered to be related to the study intervention that occur after the DLT observation period will be reviewed in context of all safety data available. That review may result in re-evaluation of the dosing level or regimen.

Severity of AEs will be graded according to CTCAE v4.03. In Cohort 1 (EC + FOLFIRI), any DLT occurring prior to initiation of encorafenib on Day 3 will not be considered for tolerability decisions.

**Table 9. DLT Criteria**

| |
|---|
| • Any AE or laboratory value considered unrelated to underlying disease, disease progression, intercurrent illness or concomitant medications/therapies resulting in the inability to tolerate at least 75% of the planned dose intensity ([administered dose in mg/planned dose in mg] x 100) of each study intervention during the 28-day DLT period. |
| **General disorders and administration site conditions**<br>• Fatigue Grade 3 for >14 consecutive days |
| **Respiratory disorders**<br>• Interstitial lung disease/pneumonitis Grade ≥2 |
| **Skin and subcutaneous tissue disorders**[a]<br>• Rash, HFSR, or photosensitivity Grade 3 for >14 consecutive days despite maximal skin toxicity treatment (as per local practice)<br>• Rash, HFSR, or photosensitivity Grade 4 |
| **Gastrointestinal disorders**[a]<br>• Diarrhea Grade 3 for ≥48 hours despite optimal use of antidiarrheal therapy<br>• Diarrhea Grade 4<br>• Nausea/vomiting Grade 3 for ≥48 hours despite optimal use of antiemetic therapy<br>• Vomiting Grade 4<br>• Mucositis Grade ≥3 |

090177e1a02cb071\Approved\Approved On: 14-Mar-2024 15:31 (GMT)

**Table 9. DLT Criteria**

| |
|---|
| **Investigations**<br>• Total bilirubin Grade ≥3<br>• AST or ALT Grade ≥3 in conjunction with total bilirubin Grade ≥2 of any duration<br>• AST or ALT Grade 3 for >7 consecutive days<br>• AST or ALT Grade 4<br>• Serum creatinine Grade ≥3<br>• ANC Grade 4 for >7 consecutive days<br>• ≥ Grade 3 febrile neutropenia<br>• Grade 3 platelet count decreased with signs of clinically significant bleeding<br>• Platelet count Grade 4<br>• ECG QTcF prolonged ≥Grade 3[b] |
| **Uveitis**<br>• Grade ≥3 for >21 consecutive days<br>• Grade 4 confirmed by ophthalmic examination |
| **Neurotoxicity**<br>• Paresthesia/dysesthesias Grade ≥3 |
| **Other hematologic and nonhematologic toxicities[c]**<br>• Any other Grade ≥3 AE except:<br>  ○ Lymphocyte count decreased (lymphopenia) Grade ≥3 unless clinically significant |

a. Prophylactic treatment for nausea/vomiting or skin AEs should be used at the discretion of the Investigator if the participant experiences nausea/vomiting and/or skin AEs Grade ≥1.
b. QTcF must be prolonged on two separate ECGs at least 5 minutes apart.
c. Isolated laboratory changes (eg alkaline phosphatase, cholesterol, lipase, serum amylase) or those due to sampling or laboratory errors without associated clinical signs or symptoms may be determined to not be DLTs upon review and agreement by the Investigator and medical monitor.

### 6.1.2. Treatment Regimens

Participants enrolled in the SLI will receive either a regimen of EC + mFOLFOX6 or a regimen of EC + FOLFIRI in 28 day cycles. Recommended regimen administrations for cetuximab, 5-FU, irinotecan, leucovorin and oxaliplatin are described in Table 10; however, all should be administered in accordance with their respective locally approved label or local institutional standards.

090177e1a02cb071\Approved\Approved On: 14-Mar-2024 15:31 (GMT)

**Table 10. Treatment Regimens (Safety Lead-in)**

| Study Treatments | Dose | Frequency |
|---|---|---|
| Encorafenib | 300 mg (4 × 75 mg) oral capsule | QD |
| Cetuximab | 500 mg/m$^2$ (120-minute IV infusion)[a] | Q2W |
| mFOLFOX6 | Oxaliplatin 85 mg/m$^2$ (120-minute IV infusion) [a]<br>Leucovorin 400 mg/m$^2$ (120-minute IV infusion)[a,b,c]<br>5-FU 400 mg/m$^2$ IV bolus, then 5-FU 2400 mg/m$^2$ continuous IV infusion over 46-48 hours[a] | Q2W |
| Encorafenib<br>Cetuximab<br>FOLFIRI | 300 mg (4 × 75 mg) oral capsule[d]<br>500 mg/m$^2$ (120-minute IV infusion) [a]<br>Irinotecan 180 mg/m$^2$ (90-minute IV infusion) [a]<br>Leucovorin 400 mg/m$^2$ (120-minute IV infusion) [a,b,c]<br>5-FU 400 mg/m$^2$ IV bolus, then 5-FU 2400 mg/m$^2$ continuous IV infusion over 46-48 hours[a] | QD<br>Q2W<br>Q2W |

a Approximate infusion times
b Alternatively, 200 mg/m$^2$ levo-leucovorin (120-minute IV infusion) may be administered
c Leucovorin may be co-administered with the oxaliplatin or irinotecan infusion
d Encorafenib dosing will not begin until Cycle 1 Day 3 for participants receiving FOLFIRI

Participants randomized to Arm A will receive encorafenib + cetuximab in 28 day cycles. Recommended regimen administrations for cetuximab are described in Table 11; however, cetuximab should be administered in accordance with the locally approved label or local institutional standards.

**Table 11. Treatment Regimen (Phase 3, Arm A)**

| Study Treatments | Dose | Frequency |
|---|---|---|
| Encorafenib | 300 mg (4 × 75 mg) oral capsule | QD |
| Cetuximab | 500 mg/m$^2$ (120-minute IV infusion)[a] | Q2W |

a Approximate infusion times
***Note***: In the Phase 3 portion of the study, participants must receive their first dose of study intervention within 5 days of randomization.

Participants randomized to Arm B will receive a regimen of EC + mFOLFOX6 in 28-day cycles. Recommended regimen administrations for bolus and infusion treatment are described in Table 12; however, all should be administered in accordance with their respective locally approved label or local institutional standards.



090177e1a02cb071\Approved\Approved On: 14-Mar-2024 15:31 (GMT)

**Table 12. Treatment Regimens (Phase 3, Arm B)**

| Study Treatments | Dose | Frequency |
|---|---|---|
| Encorafenib | 300 mg (4 × 75 mg) oral capsule | QD |
| Cetuximab | 500 mg/m² (120-minute IV infusion)[a] | Q2W |
| mFOLFOX6 | Oxaliplatin 85 mg/m² (120-minute IV infusion) [a]<br>Leucovorin 400 mg/m² (120-minute IV infusion)[a,b,c]<br>5-FU 400 mg/m² IV bolus, then 5-FU 2400 mg/m² continuous IV infusion over 46-48 hours[a] | Q2W |

a Approximate infusion times

b Alternatively, 200 mg/m² levo-leucovorin (120-minute IV infusion) may be administered

c Leucovorin may be co-administered with the oxaliplatin

***Note***: In the Phase 3 portion of the study, participants must receive their first dose of study intervention within 5 days of randomization.

Participants randomized to the Control Arm will receive one of the following per Investigator choice: mFOLFOX6 ± bevacizumab, FOLFOXIRI ± bevacizumab or CAPOX ± bevacizumab.Recommended regimen administrations for 5-FU, irinotecan, leucovorin, oxaliplatin capecitabine and bevacizumab are described in Table 13; however, all should be administered in accordance with their respective locally approved label or local institutional standards.

090177e1a02cb071\Approved\Approved On: 14-Mar-2024 15:31 (GMT)

**Table 13. Treatment Regimens (Phase 3, Control Arm [Arm C])**

| Study Treatments | Dose | Frequency |
|---|---|---|
| mFOLFOX6 ± Bevacizumab | Oxaliplatin 85 mg/m$^2$ (120-minute IV infusion)[a]<br>Leucovorin 400 mg/m$^2$ (120-minute IV infusion)[a,b,c]<br>5-FU 400 mg/m$^2$ IV bolus, then 5-FU 2400 mg/m$^2$ continuous IV infusion over 46-48 hours[a]<br>Bevacizumab (optional; given per prescribing instructions) | Q2W 28-day Cycle |
| FOLFOXIRI ± Bevacizumab | Irinotecan 165 mg/m$^2$ (90-minute IV infusion)[a]<br>Oxaliplatin 85 mg/m$^2$ (120-minute IV infusion)[a]<br>Leucovorin 400 mg/m$^2$ (120-minute IV infusion)[a,b,c]<br>5-FU 2400 or 3200 mg/m$^2$ continuous IV infusion over 46-48 hours (per local standard of care)[a]<br>Bevacizumab (optional; given per prescribing instructions) | Q2W 28-day Cycle |
| CAPOX ± Bevacizumab | Oxaliplatin 130 mg/m$^2$ (120-minute IV infusion)[a]<br>Capecitabine 1000 mg/m$^2$ oral tablet<br>Bevacizumab (optional; given per prescribing instructions) | Q3W 21-day Cycle<br>BID Days 1-14 |

a Approximate infusion times

b Alternatively, 200 mg/m$^2$ levo-leucovorin (120-minute IV infusion) may be administered

c Leucovorin may be co-administered with the oxaliplatin or irinotecan infusion

***Note***: The decision of which regimen to use in the Control Arm is at the participant/treating Investigator's discretion but must be declared prior to randomization and the choice of regimen must not be changed during the course of the participant's treatment.

***Note***: Participants with colonic or rectal stent in place should not receive bevacizumab in any of the Control Arm regimens.

***Note***: In the Phase 3 portion of the study, participants must receive their first dose of study intervention within 5 days of randomization.

Two randomized (2:1 randomization) treatment arms in Cohort 3 will be included: EC in combination with FOLFIRI in first-line participants (Arm D) (Table 14) and FOLFIRI with or without bevacizumab in first-line participants (Arm E) (Table 15). Cohort 3 will start after the enrollment of the Phase 3 portion is complete and enrollment will only be conducted in countries where authorized.

090177e1a02cb071\Approved\Approved On: 14-Mar-2024 15:31 (GMT)

**Table 14. Treatment Regimens: Cohort 3 (Arm D)**

| Study Treatments | Dose | Frequency |
|---|---|---|
| Encorafenib | 300 mg (4 × 75 mg) oral capsule[d] | QD |
| Cetuximab | 500 mg/m² (120-minute IV infusion)[a] | Q2W |
| FOLFIRI | Irinotecan 180 mg/m² (90-minute IV infusion) [a]<br>Leucovorin 400 mg/m² (120-minute IV infusion) [a,b,c]<br>5-FU 400 mg/m² IV bolus, then 5-FU 2400 mg/m² continuous IV infusion over 46-48 hours[a] | Q2W |

a Approximate infusion times
b Alternatively, 200 mg/m² levo-leucovorin (120-minute IV infusion) may be administered
c Leucovorin may be co-administered with the irinotecan infusion
d Encorafenib dosing will begin on Cycle 1 Day 1

**Table 15. Treatment Regimens: Cohort 3 (Arm E)**

| Study Treatments | Dose | Frequency |
|---|---|---|
| FOLFIRI | Irinotecan 180 mg/m² (90-minute IV infusion) [a]<br>Leucovorin 400 mg/m² (120-minute IV infusion) [a,b,c]<br>5-FU 400 mg/m² IV bolus, then 5-FU 2400 mg/m² continuous IV infusion over 46-48 hours[a] | Q2W |
| Bevacizumab | Bevacizumab (optional; given per prescribing instructions) | |

a Approximate infusion times
b Alternatively, 200 mg/m² levo-leucovorin (120-minute IV infusion) may be administered
c Leucovorin may be co-administered with the irinotecan infusion

### 6.1.3. Administration

#### 6.1.3.1. Administration of Encorafenib

Encorafenib will be administered on a QD schedule, PO as a flat-fixed dose, and not by body weight or BSA. Encorafenib should be taken without regard to food. Participants will swallow the encorafenib capsules whole, and will not manipulate (eg, open or crush) or chew the encorafenib capsules.



090177e1a02cb071\Approved\Approved On: 14-Mar-2024 15:31 (GMT)

Participants should be instructed to take encorafenib capsules daily with a large glass of water (~250 mL) in the morning at approximately the same time every day. If a participant vomits at any time after dosing, the dose of study intervention should not be re-administered. Doses of encorafenib that are omitted for AEs or any other reason should not be made up during the day (ie, within the 12 hours of the next encorafenib dose), or at the end of the dosing period. Additional information regarding encorafenib administration can be found in the IP Manual. On days when a blood collection is scheduled at the investigational site, participants will take the morning dose of encorafenib at the site under the supervision of the Investigator or designee. On all other days, participants will take encorafenib at home.

Participants must avoid consumption of grapefruit, pomegranates, star fruits, Seville oranges or products containing the juice of each during the entire study and preferably 7 days before the first dose of study intervention, due to potential CYP3A4 interaction with the study interventions. Orange juice is allowed.

When EC is combined with FOLFIRI in the SLI, the first dose of encorafenib will be administered on Cycle 1 Day 3. When encorafenib is combined with FOLFIRI in Cohort 3 Arm D, the first dose of encorafenib will be administered on Cycle 1 Day 1. When encorafenib is administered with irinotecan, encorafenib should be administered immediately prior to the start of the irinotecan infusion. On PK days in particular, it should be ensured that the encorafenib dosing occurs within 5 minutes prior to start of infusion. On all other days, encorafenib will be administered with the start of irinotecan or oxaliplatin infusion.

The pharmacist or study nurse will ensure that the appropriate dose is dispensed and will provide the participant with at least the appropriate number of encorafenib capsules for the number of doses to be taken prior to the next scheduled visit. The site personnel will train the participant and/or the participant's caregiver on dosing procedures for the study intervention.

Participants will receive a diary to document self-administered dosing of encorafenib and capecitabine in each cycle to include the dose of study intervention taken, the date of dosing (and times if applicable), and if any doses were missed and the reason for the missed dose. One diary will be provided per cycle. Participants will be instructed to return unused encorafenib, capecitabine and the participant diary to the site at the end of each cycle. Drug accountability must be performed on a regular basis.

The Investigator or responsible site personnel should instruct the participant to take encorafenib as per protocol (promote compliance). The dosage prescribed and dispensed to the participant and all dose changes and all missed doses during the study must be recorded in the eCRF.

#### 6.1.3.2. Administration of Capecitabine, Cetuximab, 5-Fluorouracil, Irinotecan, Leucovorin, Oxaliplatin and Bevacizumab

Capecitabine, cetuximab, 5-FU, irinotecan, leucovorin, oxaliplatin and bevacizumab are to be administered in accordance with their respective locally approved labels or local institutional standards. For detailed dosing information, see Section 6.1.2. Prior to the first cetuximab



090177e1a02cb071\Approved\Approved On: 14-Mar-2024 15:31 (GMT)

infusion, participants should receive premedication with an antihistamine and a corticosteroid at least 1 hour prior to administration.

## 6.2. Preparation/Handling/Storage/Accountability

1. The investigator or designee must confirm appropriate temperature conditions have been maintained during transit for all study interventions received and any discrepancies are reported and resolved before use of the study intervention.

2. Only participants enrolled in the study may receive study intervention and only authorized site staff may supply or administer study intervention. All study interventions must be stored in a secure, environmentally controlled, and monitored (manual or automated recording) area in accordance with the labeled storage conditions with access limited to the investigator and authorized site staff. At a minimum, daily minimum and maximum temperatures for all site storage locations must be documented and available upon request. Data for nonworking days must indicate the minimum and maximum temperatures since previously documented for all site storage locations upon return to business.

3. Any excursions from the study intervention label storage conditions should be reported to Pfizer upon discovery along with any actions taken. The site should actively pursue options for returning the study intervention to the storage conditions described in the labeling, as soon as possible. Once an excursion is identified, the study intervention must be quarantined and not used until Pfizer provides permission to use the study intervention. Specific details regarding the definition of an excursion and information the site should report for each excursion will be provided to the site in the IP manual.

4. Any storage conditions stated in the SRSD will be superseded by the storage conditions stated on the label.

5. Study interventions should be stored in their original containers.

6. Site staff will instruct participants on the proper storage requirements for take-home study intervention.

7. See the IP manual for storage conditions of the study intervention once reconstituted and/or diluted.

8. The investigator, institution, or the head of the medical institution (where applicable) is responsible for study intervention accountability, reconciliation, and record maintenance (ie, receipt, reconciliation, and final disposition records), such as the IPAL or sponsor-approved equivalent. All study interventions will be accounted for using a study intervention accountability form/record. All study intervention that is taken home by the participant, both used and unused, must be returned to the investigator by the participant. Returned study intervention must not be redispensed to the participants.



090177e1a02cb071\Approved\Approved On: 14-Mar-2024 15:31 (GMT)

9. Further guidance and information for the final disposition of unused study interventions are provided in the IP manual. All destruction must be adequately documented. If destruction is authorized to take place at the investigator site, the investigator must ensure that the materials are destroyed in compliance with applicable environmental regulations, institutional policy, and any special instructions provided by Pfizer.

Upon identification of a product complaint, notify the sponsor within 1 business day of discovery as described in the IP Manual.

### 6.2.1. Preparation and Dispensing

A qualified staff member will dispense the study intervention using the IRT system via unique container numbers in the bottles or vials provided, in quantities appropriate according to the SoA. A second staff member will verify the dispensing. The participant/caregiver should be instructed to maintain the product in the bottles or vials, provided throughout the course of dosing and return the bottles or vials to the site at the next study visit.

See the IP manual for instructions on how to prepare the study intervention for administration. Study intervention should be prepared and dispensed by an appropriately qualified and experienced member of the study staff (eg, physician, nurse, physician's assistant, nurse practitioner, pharmacy assistant/technician, or pharmacist) as allowed by local, state, and institutional guidance. A second staff member will verify the dispensing.

All vials are single-use.

Only qualified personnel who are familiar with procedures that minimize undue exposure to themselves and to the environment should undertake the preparation, handling, and safe disposal of chemotherapeutic agents.

## 6.3. Measures to Minimize Bias: Randomization and Blinding

### 6.3.1. Allocation to Study Intervention

This is an open-label study; however, the specific study intervention dispensed to the participant will be assigned using an IRT. For the Phase 3 portion of the study, a randomization list stratified by ECOG (0 versus 1) and region (US/Canada versus Europe versus Rest of World) will be used. For Cohort 3 of the study, a randomization list stratified by ECOG (0 versus 1) will be used.

The site will contact the IRT prior to the start of study intervention administration for each participant. The site will record the study intervention assignment on the applicable CRF, if required. Potential bias will be reduced by central randomization and BICR evaluation of imaging data (Section 8.1.1.1).

The investigator's knowledge of the treatment should not influence the decision to enroll a particular participant or affect the order in which participants are enrolled.



090177e1a02cb071\Approved\Approved On: 14-Mar-2024 15:31 (GMT)

Study intervention will be dispensed at the study visits summarized in the SoA.

Returned study intervention must not be redispensed to the participants.

The study-specific IRT reference manual and IP manual will provide the contact information and further details on the use of the IRT system.

## 6.4. Study Intervention Compliance

When participants are dosed at the site, they will receive study intervention directly from the investigator or designee, under medical supervision. The dose of study intervention and study participant identification will be confirmed at the time of dosing by a member of the study site staff other than the person administering the study intervention.

The site will complete the required dosage Preparation Record located in the IP manual.  The use of the Preparation Record is preferred, but it does not preclude the use of an existing appropriate clinical site documentation system.  The existing clinical site's documentation system should capture all pertinent/required information on the preparation and administration of the dose.  This may be used in place of the Preparation Record after approval from the sponsor and/or designee.

When participants self-administer study intervention(s) at home, compliance with study intervention will be assessed at each visit.  Participants will receive a diary to document self-administered dosing of study intervention (encorafenib or capecitabine, as applicable) in each cycle to include the dose of study intervention taken, the date of dosing, if any doses were missed and the reason for the missed dose.  Compliance will be assessed by direct questioning, counting returned tablets/capsules, and a review of diary entries during the site visits and documented in the source documents and eCRF.  Deviation(s) from the prescribed dosage regimen should be recorded in the eCRF.

A record of the number of encorafenib capsules and capecitabine tablets dispensed to and taken by each participant must be maintained and reconciled with study intervention and compliance records.  Intervention start and stop dates, including dates for intervention delays and/or dose reductions, will also be recorded in the eCRF.

## 6.5. Concomitant Therapy

The following subsections detail permitted and prohibited concomitant therapies for encorafenib. Guidance on concomitant therapy for all other study interventions can be found in the respective SRSDs.

### 6.5.1. Permitted Concomitant Medications/Therapies

In general, the use of any concomitant medication/therapies deemed necessary for the care of the participant is permitted, unless otherwise specified. Additional information regarding concomitant medications/therapies is provided in the Investigator's Brochure for encorafenib and the SRSD for all other study interventions.



090177e1a02cb071\Approved\Approved On: 14-Mar-2024 15:31 (GMT)

Participants receiving medications outlined below must be carefully monitored for potentiating of toxicity due to any individual concomitant medication and may require dose titration of the drug substance. Investigators should use caution when prescribing concomitant medications, as clinical experience with these compounds in participants with cancer is often limited. Investigators should contact the Sponsor when they are unsure whether a drug should be prescribed to a participant in the clinical study. All concomitant medications/therapies, transfusions, procedures and dietary supplements must be documented on the eCRF.

#### 6.5.1.1. Surgical Resection

Participants who undergo complete resection of all metastatic disease may continue study intervention at the discretion of the Investigator and following discussion with the medical monitor.

#### 6.5.1.2. CYP and UGT Substrates and Inhibitors

Encorafenib is a reversible inhibitor of CYP1A2, CYP2B6, CYP2C8/9, CYP2D6, CYP3A4 and UGT1A1 based on in vitro studies. It is also a time-dependent inhibitor of CYP3A4, and induced CYP1A2, CYP2B6, CYP2C9 and CYP3A4 in human hepatocytes. Permitted medications to be used with caution in this study include those that are sensitive substrates of CYP2B6, CYP2C8/9, CYP2D6, CYP3A4 and UGT1A1 or those substrates that have an NTI. Based on recently available data from an ongoing DDI study in patients, single dose encorafenib does not inhibit CYP3A at clinically used doses. Following multiple doses encorafenib plasma exposures at steady-state are lower than expected, indicating net CYP3A auto-induction.

**There is a potential for encorafenib to induce CYP3A4, which may reduce the effectiveness of hormonal contraception methods. Therefore, the use of at least 1 form of non-hormonal contraception is required for females of childbearing potential during participation in this study.**

Caution should be used in participants receiving concomitant treatment with other drugs that are substrates of CYP3A4 as the efficacy of these drugs could be reduced when administered with encorafenib.

Encorafenib has been identified to be primarily metabolized by CYP3A4 and to a lesser extent by CYP2C19 in vitro. **Concomitant use of moderate CYP3A4 inhibitors should be avoided.** If use of a moderate CYP3A4 inhibitor is unavoidable, short-term use (≤30 days) following discussion with the Sponsor may be permitted with an accompanying dose reduction to one-half of the encorafenib dose prior to use of the moderate CYP3A4 inhibitor. After the inhibitor has been discontinued for 3 to 5 elimination half-lives, resume the encorafenib dose that was taken prior to initiating the CYP3A4 inhibitor.

Irinotecan is subject to extensive metabolic conversion by various enzyme systems, including esterases to form the active metabolite SN-38, and UGT1A1 mediating glucuronidation of



090177e1a02cb071\Approved\Approved On: 14-Mar-2024 15:31 (GMT)

SN-38 to form the inactive glucuronide metabolite SN-38G. **Concomitant use of strong UGT1A1 inhibitors should be avoided for patients taking irinotecan.**

For tabulated CYP substrates, inhibitors and inducers to be used with caution or avoided, please consult the following open-access websites:

- https://www.fda.gov/drugs/developmentapprovalprocess/developmentresources/druginteractionslabeling/ucm093664.htm
- https://drug-interactions.medicine.iu.edu/MainTable.aspx

Additional information on CYP substrates, inhibitors and inducers can be found on the following subscription-based website:

- https://www.druginteractionsolutions.org/

#### 6.5.1.3. Transporter Substrates and Inhibitors

Encorafenib is a BCRP and P-gp inhibitor as well as a potent inhibitor of the renal transporters, OAT1, OAT3 and OCT2, and the hepatic transporters OATP1B1 and OATP1B3.

The co-administration of drugs that are known to be sensitive or NTI substrates of BCRP, P-gp, OAT1, OAT3, OCT2, OATP1B1 and OATP1B3 should be used with caution. Dose reductions of drugs that are substrates of OATP1B1, OATP1B3, or BCRP may be required when used concomitantly with encorafenib.

For tabulated transporter substrates, inhibitors and inducers to be used with caution or avoided, please consult with the FDA website:

- https://www.fda.gov/drugs/developmentapprovalprocess/developmentresources/druginteractionslabeling/ucm093664.htm

#### 6.5.1.4. Drugs with a Conditional or Possible Risk to Prolong the QT Interval and/or Induce Torsade de Pointes

Investigators should use caution when administering encorafenib or oxaliplatin with concomitant medications with a known, conditional or possible risk to prolong the QT interval and/or induce TdP. Participants receiving such medications must be carefully monitored for potentiating of toxicity due to any individual concomitant medication and may require dose titration of the concomitant medication. See the CredibleMeds® website:

- https://crediblemeds.org/

### 6.5.2. Prohibited Concomitant Therapy

The following therapies are prohibited while participants are receiving study intervention (unless otherwise noted):



090177e1a02cb071\Approved\Approved On: 14-Mar-2024 15:31 (GMT)

- Additional systemic anticancer agents such as cytotoxic chemotherapy, small-molecule targeted agents, biological agents, immune response modifiers or hormonal therapy.

- Investigational drugs, vaccines and devices.
  ***Note:*** COVID-19 vaccines that are fully approved, approved under an emergency use authorization or are conditionally approved are considered allowed concomitant medications and standard AE collection and reporting processes should be followed. The timing of vaccine dosing relative to the dosing of study medication is at the discretion of the investigator although, should be avoided on the first day of study medication dosing and/or during the DLT observation period, if applicable.

  ***Note:*** When considering administration of live vaccines within 30 days before the first dose of study intervention, during, and within 90 days after the last dose of study intervention, the investigator should evaluate the benefits and risks for each individual patient, taking into account the administered regimen and the potential risks in immunocompromised patients.

- Radiation therapy (not including palliative radiotherapy at focal sites that covers ≤10% of the bone marrow reserve)
  ***Note:*** The participant must have clear measurable disease outside the radiated field.
  Note: Radiation therapy to a symptomatic solitary lesion or to the brain may be considered on a case-by-case basis after consultation with the medical monitor.
  ***Note:*** Administration of palliative radiation therapy may be considered clinical progression for the purposes of determining PFS if accompanied by radiologic progression.

- Concomitant strong systemic CYP3A4 inhibitors, which could significantly increase the exposure of encorafenib and irinotecan.

  For tabulated prohibited CYP substrates, inhibitors and inducers, please consult the following open-access websites:

  - https://www.fda.gov/drugs/developmentapprovalprocess/developmentresources/druginteractionslabeling/ucm093664.htm

  - https://drug-interactions.medicine.iu.edu/MainTable.aspx

  Additional information on CYP substrates, inhibitors and inducers can be found on the following subscription-based website:

  - https://www.druginteractionsolutions.org/

- Concomitant moderate or strong systemic CYP3A4 inducers, which could significantly decrease the exposure of encorafenib and irinotecan.



090177e1a02cb071\Approved\Approved On: 14-Mar-2024 15:31 (GMT)

### 6.5.3. Premedication and Concurrent Medication

#### 6.5.3.1. Premedication for Cetuximab Administration

All participants in the SLI, those in Arm A and Arm B of the Phase 3 portion, and those in Arm D of Cohort 3 must be premedicated in accordance with the cetuximab SRSD prior to the first dose of cetuximab in an effort to prevent an infusion or hypersensitivity reaction. Premedication is recommended prior to subsequent doses, but at the investigator's discretion.

#### 6.5.3.2. Loperamide

For symptoms of diarrhea and/or abdominal cramping, participants should be instructed to begin taking loperamide per the investigator's discretion. It is generally recommended that loperamide be started at the earliest sign of (1) a poorly formed or loose stool or (2) the occurrence of 1 to 2 more bowel movements than usual in 1 day or (3) an increase in stool volume or liquidity.

The loperamide is to be sourced locally and participants should have sufficient supply on hand prior to their initial dose in case antidiarrheal support is required. Additional antidiarrheal measures may be used at the discretion of the treating physician. Participants should be instructed to increase fluid intake to help maintain fluid and electrolyte balance during episodes of diarrhea.

#### 6.5.3.3. Antibiotics

Oral fluroquinolone treatment should be considered for any of the following:

- Diarrhea persisting for more than 24 hours despite loperamide
- ANC <500 (even in the absence of diarrhea or fever)
- Fever with diarrhea (even in the absence of neutropenia)
- Antibiotic therapy should also be considered in participants who are hospitalized with prolonged diarrhea (even in the absence of neutropenia)

#### 6.5.3.4. Atropine

Lacrimation, rhinorrhea, miosis, diaphoresis, hot flashes, flushing, abdominal cramping, diarrhea, or other symptoms of early cholinergic syndrome may occur during or shortly after receiving irinotecan. Atropine, 0.25-1.0 mg IV or SC may be used to treat these symptoms. In participants with troublesome or recurrent symptoms, prophylactic administration of atropine shortly before irinotecan therapy may be considered. Additional antidiarrheal measures may be used at the discretion of the treating physician. Combination anticholinergic medications containing barbiturates or other agents (eg, Donnatal®) should not be used because these may affect irinotecan metabolism. Anticholinergics should be used with caution in participants with potential contraindications (eg, obstructive uropathy, glaucoma, tachycardia, etc).



090177e1a02cb071\Approved\Approved On: 14-Mar-2024 15:31 (GMT)

#### 6.5.3.5. Hematopoietic Growth Factors

Hematopoietic growth factors (e.g., erythropoietin, granulocyte colony-stimulating factor [G-CSF] and granulocyte-macrophage colony stimulating factor [GM-CSF], such as Nivestym and Nyvepria) are not to be administered prior to first dose of study treatment. Use of these drugs after the first dose of study intervention, is permitted at the Investigator's discretion and should be reserved for participants requiring this therapy as per the labeling of these agents or as dictated by local practice (see also the ASCO guidelines).[78]

#### 6.5.3.6. Antiemetics

The following antiemetics are allowed, but should be used with caution when administered with encorafenib as they may affect encorafenib exposure.

- NK1 receptor antagonists rolapitant and fosaprepitant (weak CYP3A4 inhibitors) with no modification to encorafenib dosing
- Aprepitant PO (moderate CYP3A4 inhibitor) is allowed in participants but encorafenib should be reduced for 3-5 half-lives
  - Reduce encorafenib to 150 mg QD (50% reduction) when co-administered with aprepitant PO.
  - Washout period is 2 days after last dose of aprepitant PO (3 half-lives).
  - Encorafenib may be restarted at 300 mg QD on third day after last dose of aprepitant.
- Dexamethasone (weak CYP3A4 inducer) as 2-20 mg with short-term or intermittent use

### 6.6. Dose Modification

Cycles will consist of 21 days in participants receiving CAPOX (with or without bevacizumab) in the Control Arm and will be 28 days in duration for all other participants. In the SLI, Arm A, Arm B and Cohort 3 Arm D, Day 1 of a cycle is defined by administration of cetuximab and any component of the chemotherapy regimen, and in the Phase 3 Control Arm C and Cohort 3 Arm E by administration of any component of the chemotherapy regimen.

For participants in the SLI, those in Arm B and the Control Arm of the Phase 3 portion, and in Cohort 3, it is recommended that Day 1 of a cycle may not begin until the ANC is ≥1500/mm$^3$, the platelet count is ≥100,000/mm$^3$, and any treatment-related GI toxicity has resolved to ≤Grade 1 or according to Investigator's clinical judgement. Participants in the SLI, Arm B of the Phase 3 portion and Cohort 3 Arm D may continue daily encorafenib, however dosing with cetuximab should not be administered until Week 1 of the next cycle along with chemotherapy.

Participants will be monitored for AEs on an ongoing basis. If the participant develops a toxicity, the dose may be modified as outlined in the following sections. All dose



090177e1a02cb071\Approved\Approved On: 14-Mar-2024 15:31 (GMT)

modifications should be based on the worst preceding toxicity. All dosing interruptions and modifications must be recorded in the eCRF.

If assessment of the toxicity can be clearly attributed to a single study intervention, administration of the offending drug should be modified and the toxicity should be followed until resolution or return to baseline according to the respective dose modification guidelines described in the following sections. Treatment with the other agents in the combination may continue without alterations. In the instance of cetuximab infusion reaction on Cycle 1 Day 1, administration with the remaining study drugs may begin once toxicity resolves and at the Investigator's discretion.

If attribution of the toxicity cannot be clearly ascribed to a single study intervention, then all agents should be evaluated as to attribution and dose modifications for each individual agent should be assessed according to the tables below. The toxicity should be followed until resolution or return to baseline.

If the toxicity recurs after a dose reduction, the process outlined above should be followed once again to determine attribution to a single study intervention or to all agents.

### 6.6.1. Dose Modifications for Encorafenib

The recommended dose reductions for encorafenib are presented in Table 16. Dose reductions beyond the second dose reduction are not allowed for encorafenib. Once a dose has been reduced for a given participant, all subsequent doses should be administered at that dose level unless further dose reduction is required or dose is re-escalated. Encorafenib may be re-escalated by a maximum of 1 dose level (and not higher than the starting dose) when the AE that resulted in an encorafenib dose reduction resolves or improves to and remains stable to the participant's baseline for a minimum of 14 days, and provided there are no safety concerns or other concomitant toxicities that would prevent encorafenib re-escalation. There is no limit to the number of times a participant can have their dose of encorafenib reduced or re-escalated; however:

- No dose re-escalation of encorafenib is allowed after a dose reduction due to prolonged QTcF ≥ 500 msec
- No dose re-escalation of encorafenib is allowed after a dose reduction due to retinal toxicity Grade ≥ 2.

Administration of encorafenib as a single agent is not recommended; if cetuximab is permanently discontinued in Arm A in the Phase 3 portion of the study, permanently discontinue encorafenib.

In the SLI, Phase 3 Arm B portion of the study and Cohort 3 Arm D, if encorafenib is permanently discontinued, cetuximab must also be permanently discontinued; however, therapy with the chemotherapy regimen may continue.



090177e1a02cb071\Approved\Approved On: 14-Mar-2024 15:31 (GMT)

090177e1a02cb071\Approved\Approved On: 14-Mar-2024 15:31 (GMT)

**Table 16. Recommended Encorafenib Dose Reductions**

| Dose Level | Encorafenib Dose |
|---|---|
| First Dose Reduction | 225 mg orally QD |
| Second Dose Reduction | 150 mg orally QD |

The recommended dose modifications for encorafenib are presented in Table 17 and are based on the existing dose modification guidelines provided in the current USPI for encorafenib.[37]

**Table 17. Recommended Encorafenib Dose Modifications**

| Severity of Adverse Reaction[a] | Dose Modification for Encorafenib |
|---|---|
| ***New Primary Malignancies*** | |
| Cutaneous (SCC, KA and any Other Suspicious Skin Lesion) | Maintain dose of encorafenib (dose interruptions or modifications are not required). Treatment of SCC, KA, and any other suspicious skin lesion (eg, new primary melanoma) should occur based upon institutional practice. |
| Non-cutaneous *RAS* mutation-positive malignancies | Permanently discontinue encorafenib. |
| ***Uveitis*** | |
| Grade 1-3 | If Grade 1 or 2 uveitis does not respond to specific (eg, topical) ocular therapy, or for Grade 3 uveitis, hold encorafenib for up to 6 weeks and repeat ophthalmic examination within 2 weeks.<br>• If Grade 1 uveitis improves to Grade 0, resume at same dose.<br>• If Grade 2 or 3 uveitis improves to Grade 0 or 1, resume at reduced dose.<br>• If not improved, permanently discontinue encorafenib. |
| Grade 4 | Permanently discontinue encorafenib |
| ***QTcF Prolongation*** | |
| QTcF >500 ms and ≤60 ms increase from baseline | Patients should have regular ECG monitoring (continuous where appropriate) until an adequately trained physician (such as a cardiologist or internist) has reviewed the data. Electrolyte abnormalities including magnesium should be corrected and cardiac risk factors for QT prolongation (eg, congestive heart failure, bradyarrhythmias) should be controlled.<br>• Hold encorafenib until QTcF ≤500 ms. Resume at reduced dose.<br>• If more than one recurrence, permanently discontinue encorafenib. |

## Table 17. Recommended Encorafenib Dose Modifications

| Severity of Adverse Reaction[a] | Dose Modification for Encorafenib |
|---|---|
| QTcF >500 ms and >60 ms increase from baseline | Patients should have regular ECG monitoring (continuous where appropriate) until an adequately trained physician (such as a cardiologist or internist) has reviewed the data. Electrolyte abnormalities including magnesium should be corrected and cardiac risk factors for QT prolongation (e.g., congestive heart failure, bradyarrhythmias) should be controlled.<br>• Permanently discontinue encorafenib. |
| ***Hepatotoxicity*** | |
| Grade 2 AST or ALT increased | Maintain encorafenib dose.<br>• If no improvement within 4 weeks, hold encorafenib until improves to Grade 0-1 or to pre-treatment/baseline levels and then resume at same dose. |
| Grade 3 or 4 AST or ALT increased | see *Other Adverse Reactions.* |
| ***Rash*** | |
| Grade 1 | Maintain dose level of encorafenib.<br>Initiate rash treatment per institutional standards; rash should be closely monitored. |
| Grade 2 | 1st occurrence:<br>• Maintain dose level of encorafenib<br>• Initiate rash treatment per institutional standards; rash should be closely monitored.<br>• Reassess within ≤14 days. If rash worsens or does not improve, interrupt dosing of encorafenib until resolved to Grade ≤1. Then resume treatment at current dose level of encorafenib. For dermatitis acneiform, treatment with encorafenib may be maintained if, in the judgment of the investigator, the rash is considered to be unrelated to encorafenib. If treatment with encorafenib was maintained and no improvement within 8 days, interrupt dosing of encorafenib<br>2nd occurrence:<br>• Reassess within ≤14 days. If rash worsens or does not improve, interrupt dosing of encorafenib until resolved to Grade ≤1. Then resume treatment at current dose level of encorafenib. For dermatitis acneiform rash, treatment with encorafenib may be maintained if, in the judgment of the investigator, the rash is considered to be unrelated to encorafenib. If treatment with encorafenib was maintained and no improvement within 8 days, interrupt dosing of encorafenib |



090177e1a02cb071\Approved\Approved On: 14-Mar-2024 15:31 (GMT)

090177e1a02cb071\Approved\Approved On: 14-Mar-2024 15:31 (GMT)

## Table 17. Recommended Encorafenib Dose Modifications

| Severity of Adverse Reaction[a] | Dose Modification for Encorafenib |
|---|---|
| Grade 3 | 1st occurrence:<br>• Interrupt dosing of encorafenib until resolved to Grade ≤1. Reassess weekly. Then resume treatment at current dose level of encorafenib.<br>• Consider referral to dermatologist and manage rash per dermatologist's recommendation.<br>2nd occurrence:<br>• Interrupt dosing of encorafenib until resolved to Grade ≤1. Then resume treatment at 1 reduced dose level of encorafenib. Resume treatment with encorafenib at the same dose level if, in the judgment of the Investigator, the rash is considered to be unrelated to encorafenib<br>• Consider referral to dermatologist and manage rash per dermatologist's recommendation |
| Grade 4 | Permanently discontinue encorafenib. |
| ***Nausea/Vomiting*** | |
| Grade 1-2 | Maintain dose level of encorafenib. Promptly institute antiemetic measure. |
| Grade 3 | Interrupt dosing of encorafenib until resolved to Grade ≤1. Then resume treatment at 1 reduced dose level of encorafenib.<br>***Note:*** Interrupt dosing of encorafenib for ≥Grade 3 vomiting or Grade 3 nausea only if the vomiting or nausea cannot be controlled with optimal antiemetics (as per local practice) |
| Grade 4 | Permanently discontinue encorafenib. |
| ***Other Adverse Reactions (including Hemorrhage)*** | |
| Recurrent Grade 2<br>*OR*<br>First occurrence of any Grade 3 | Hold encorafenib for up to 4 weeks.<br>• If improves to Grade 0-1 or to pretreatment/baseline level, resume at reduced dose.<br>• If no improvement, permanently discontinue encorafenib. |
| First occurrence of any Grade 4 | Permanently discontinue encorafenib<br>*OR*<br>Hold encorafenib for up to 4 weeks.<br>• If improved to Grade 0-1 or to pretreatment/baseline level, then resume at reduced dose.<br>• If no improvement, permanently discontinue encorafenib. |
| Recurrent Grade 3 | Consider permanently discontinuing encorafenib. |
| Recurrent Grade 4 | Permanently discontinue encorafenib. |

a. NCI CTCAE version 4.03

### 6.6.2. Dose Modifications for Cetuximab

The recommended dose reductions for cetuximab are presented in Table 18.

Cetuximab may be reduced 2 dose levels to a minimum of 300 mg/m$^2$ for AEs or laboratory abnormalities (Appendix 3). Dose reductions beyond the second dose reduction are not allowed for cetuximab. When a dose reduction is required because of an AE, no subsequent dose re-escalation of cetuximab will be permitted for that participant for the duration of study treatment. If after resolution of an AE, treatment is resumed at the same dose, and the same toxicity reoccurs with the same severity, any re-initiation of treatment must be at the next lower dose level irrespective of duration, with some exceptions for skin toxicity. In addition, a participant must permanently discontinue cetuximab if, after treatment is resumed at a lower dose of cetuximab, the same toxicity reoccurs with the same or worse severity.

Administration of cetuximab as a single agent is not recommended; if encorafenib is permanently discontinued in the SLI, Phase 3 Arm A, Phase 3 Arm B, and Cohort 3 Arm D portions of the study, cetuximab must also be permanently discontinued; however, therapy with the chemotherapy regimen may continue.

**Table 18. Recommended Cetuximab Dose Reductions**

| Dose Level | Cetuximab Dose |
|---|---|
| First Dose Reduction | 400 mg/m$^2$ Q2W |
| Second Dose Reduction | 300 mg/m$^2$ Q2W |

Recommended dose modifications for cetuximab based on the occurrence of cetuximab treatment-related AEs are summarized in Table 19. Please always refer to the locally approved product label for any adverse reaction not specifically mentioned in the table and for the most updated recommendations.

**Table 19. Recommended Dose Modifications for Cetuximab related Adverse Events**

| Severity of Adverse Event [a] | Dose Modification for Cetuximab During a Cycle of Therapy |
|---|---|
| ***Hypomagnesemia*** | Hypomagnesemia has been seen with cetuximab. Should hypomagnesemia occur, magnesium supplementation should be provided. No dose adjustment is required; however, continue careful monitoring. |
| ***Infusion Reaction:*** *If an infusion reaction occurs while cetuximab is being infused, the infusion should be stopped immediately and the participant should be evaluated.* | |
| Grade 1 or 2 | Restart and complete the disrupted infusion at the discretion of the Investigator. The infusion must be restarted at a reduced rate. Additional pre-medications such as antihistamines or low-dose systemic corticosteroids may be administered when the infusion is restarted per institutional standards.<br>All subsequent infusions must also be administered at the reduced rate. |
| Grade 3 or 4 | Permanently discontinue cetuximab |



090177e1a02cb071\Approved\Approved On: 14-Mar-2024 15:31 (GMT)

**Table 19. Recommended Dose Modifications for Cetuximab related Adverse Events**

| Severity of Adverse Event [a] | Dose Modification for Cetuximab During a Cycle of Therapy |
|---|---|
| ***Pulmonary toxicities*** | |
| Grade 2 or worsening pulmonary symptoms | Cetuximab therapy should be stopped and symptoms investigated. Cetuximab therapy may resume when symptoms resolve to ≤Grade 1 |
| Grade 3 cough, dyspnea, hypoxia, pneumonitis, or pulmonary infiltrates | Hold cetuximab until interstitial lung disease is ruled out. Permanently discontinue cetuximab if interstitial lung disease is confirmed |
| ***Rash*** | |
| Grade 1 or 2 | Maintain dose level; consider initiating appropriate therapy (such as antihistamines, topical corticosteroids, and low-dose systemic corticosteroids) |
| Grade 3, despite therapy | Hold cetuximab until resolved to ≤Grade 2, then:<br>• If resolved in ≤7 days (or ≤14 days for acneiform rash), then maintain dose level<br>• If not resolved in ≤7 days despite appropriate skin toxicity therapy (or ≤14 days for acneiform rash), then permanently discontinue cetuximab |
| Grade 3 recurrent | Hold cetuximab until resolved to ≤Grade 2, then:<br>• If resolved in ≤7 days (or ≤14 days for acneiform rash), then decrease 1 dose level<br>• If not resolved in ≤7 days despite appropriate skin toxicity therapy (or ≤14 days for acneiform rash), then permanently discontinue cetuximab<br>• Permanently discontinue cetuximab after 3rd recurrence (upon 4th occurrence) |
| Grade 4, despite skin toxicity therapy | Permanently discontinue cetuximab |

a NCI CTCAE version 4.03

### 6.6.3. Dose Modifications for Oxaliplatin, Irinotecan, 5-FU and Capecitabine as Part of mFOLFOX6, FOLFIRI, FOLFOXIRI and CAPOX Regimens

The recommended dose reductions for oxaliplatin, irinotecan, 5-FU and capecitabine are presented in, Table 20, Table 21 and Table 22. These recommended dose adjustments for each agent are based on the specific types of toxicities observed and on Investigator judgment of each participant's benefit/risk ratio. Participants who require multiple dose reductions consecutively for Grade 2 toxicity may, at the investigator's discretion, and based on the duration and reversibility of the toxicity, begin the following dose at one dose level higher than the current reduced dose level.

Additional guidance on the recommended dose reductions are as follows:



090177e1a02cb071\Approved\Approved On: 14-Mar-2024 15:31 (GMT)

- Only those agents specified in the table below should be dose reduced.
- Dose reductions beyond the third dose reduction are not allowed for oxaliplatin, irinotecan or 5-FU or beyond the second dose reduction of capecitabine.
- No dose modifications are implemented for leucovorin. If 5-FU is held or omitted, then leucovorin should also be held or omitted, accordingly.
- In the SLI, Arm B in the Phase 3 portion of the study and Arm D of Cohort 3, if oxaliplatin or irinotecan are permanently discontinued, therapy with 5-FU, encorafenib and cetuximab may continue.
- In the SLI, Arm B in the Phase 3 portion of the study and Arm D of Cohort 3, if cetuximab is permanently discontinued, encorafenib may be continued in combination with the chemotherapy regimen.
- In the SLI and Arm B of the Phase 3 portion of the study, if only part of the mFOLFOX6 regimen is permanently discontinued, therapy with the remaining chemotherapy, encorafenib and cetuximab, may continue.
- In the SLI and Arm B of the Phase 3 portion of the study, if the entire regimen of mFOLFOX6 is permanently discontinued, therapy with encorafenib and cetuximab may continue.
- In the SLI and Arm B of the Phase 3 portion of the study, if the entire regimen of mFOLFOX6 and cetuximab are permanently discontinued, encorafenib also needs to be discontinued.
- In the Phase 3 Control Arm C and Cohort 3 Arm E, if only part of the chemotherapy regimen is permanently discontinued, therapy with the remaining chemotherapy regimen may continue.

**Table 20. Recommended Dose Reductions for Oxaliplatin, Irinotecan and 5-FU as Part of mFOLFOX6 (SLI participants, Phase 3 Arm B and Arm C [Control]), and FOLFIRI (in SLI and Cohort 3 Arm D and Arm E**

| Dose Level | Oxaliplatin | Irinotecan | 5-FU Bolus | 5-FU Infusion |
|---|---|---|---|---|
| First Dose Reduction | 65 $mg/m^2$ | 150 $mg/m^2$ | 200 $mg/m^2$ | 1920 $mg/m^2$ per 46-48 h |
| Second Dose Reduction | 50 $mg/m^2$ | 120 $mg/m^2$ | Omit | 1600 $mg/m^2$ per 46-48 h |
| Third Dose Reduction | 40 $mg/m^2$ | 100 $mg/m^2$ | Omit | 1360 $mg/m^2$ per 46-48 h |

090177e1a02cb071\Approved\Approved On: 14-Mar-2024 15:31 (GMT)

**Table 21. Recommended Dose Reductions for Oxaliplatin, Irinotecan and 5-FU as Part of FOLFOXIRI (Phase 3 Arm C [Control Arm])**

| Dose Level | Oxaliplatin | Irinotecan | 5-FU Infusion (Starting dose 2400 mg/m$^2$) | 5-FU Infusion (Starting dose 3200 mg/m$^2$) |
|---|---|---|---|---|
| First Dose Reduction | 65 mg/m$^2$ | 130 mg/m$^2$ | 1920 mg/m$^2$ | 2560 mg/m$^2$ |
| Second Dose Reduction | 50 mg/m$^2$ | 100 mg/m$^2$ | 1600 mg/m$^2$ | 2100 mg/m$^2$ |
| Third Dose Reduction | 40 mg/m$^2$ | 80 mg/m$^2$ | 1360 mg/m$^2$ | 1720 mg/m$^2$ |

**Table 22. Recommended Dose Reductions for Oxaliplatin and Capecitabine as Part of CAPOX (Phase 3 Control Arm)**

| Dose Level | Oxaliplatin | Capecitabine |
|---|---|---|
| First Dose Reduction | 100 mg/m$^2$ | 750 mg/m$^2$ |
| Second Dose Reduction | 80 mg/m$^2$ | 500 mg/m$^2$ |
| Third Dose Reduction | 65 mg/m$^2$ | Omit |

Recommended dose modifications for oxaliplatin, irinotecan, and 5-FU based on the occurrence of mFOLFOX6, FOLFIRI and FOLFOXIRI treatment-related AEs are summarized in Table 23. Please also refer to the locally approved product label for any adverse reaction not specifically mentioned in the table and for the most updated recommendations regarding dose modifications for individual chemotherapeutic agents, which may vary according to individual product labels.

**Table 23. Recommended Oxaliplatin, Irinotecan and 5-FU Dose Modifications**

| Severity of Adverse Reaction[a] | Recommended Dose Modifications |
|---|---|
| ***Hematologic toxicities*** | |
| Grade 2 neutropenia or thrombocytopenia | • Reduce 5-FU bolus one dose level with or without reducing 5-FU infusion<br>• Consider reducing or discontinuing 5-FU bolus before reducing 5-FU infusion<br>• Consider reducing either oxaliplatin or irinotecan one dose level<br>For recurrence:<br>• Consider discontinuing 5-FU bolus |



090177e1a02cb071\Approved\Approved On: 14-Mar-2024 15:31 (GMT)

### Table 23. Recommended Oxaliplatin, Irinotecan and 5-FU Dose Modifications

| Severity of Adverse Reaction[a] | Recommended Dose Modifications |
|---|---|
| Grade 3 or 4 neutropenia or thrombocytopenia | Hold 5-FU (bolus and infusion) and either oxaliplatin or irinotecan<br>• If counts recover to ANC ≥1500 and platelets ≥75,000:<br>  ○ Permanently discontinue 5-FU bolus<br>  ○ Resume 5-FU infusion<br>  ○ Resume either oxaliplatin or irinotecan at one lower dose level<br>  ○ Administer G-CSF at the investigator's discretion<br>  ○ Held doses will not be made up |
| Febrile neutropenia (defined as ANC <1000 and T ≥38.5°C) | • Hold 5-FU (bolus and infusion) and either oxaliplatin or irinotecan<br>• If fever and neutropenia resolve (ANC ≥1500 and platelets ≥75,000):<br>  ○ Permanently discontinue 5-FU bolus<br>  ○ Resume 5-FU infusion and either oxaliplatin or irinotecan at one lower dose level<br>  ○ Administer G-CSF at the investigator's discretion<br>  ○ Held doses will not be made up |
| ***Gastrointestinal toxicities*** | |
| For symptoms of diarrhea and/or abdominal cramping, participants should be instructed to begin taking loperamide as described in Section 6.5.3.2. In some instances, treatment with oral fluroquinolone and/or atropine may be appropriate as described in Section 6.5.3.3 and Section 6.5.3.4, respectively. For severe reactions during the first cycle of treatment, a DPD deficiency may be involved; testing for DPD deficiency may be considered. | |
| Grade 2 diarrhea following maximal medical intervention | Reduce 5-FU (bolus and/or infusion) and irinotecan one dose level |
| Grade 3 or 4 diarrhea | • Hold 5-FU (bolus and infusion) and either oxaliplatin or irinotecan<br>• If diarrhea resolves to ≤Grade 2, resume 5-FU (bolus and infusion) and either oxaliplatin or irinotecan at one lower dose level<br>• Held doses will not be made up |
| Grade 2 mucositis | • Reduce 5-FU bolus one dose level with or without reducing 5-FU infusion |

090177e1a02cb071\Approved\Approved On: 14-Mar-2024 15:31 (GMT)

### Table 23. Recommended Oxaliplatin, Irinotecan and 5-FU Dose Modifications

| Severity of Adverse Reaction[a] | Recommended Dose Modifications |
|---|---|
| Grade 3 mucositis | • Hold 5-FU (bolus and infusion) and either oxaliplatin or irinotecan<br>• If mucositis resolves to ≤Grade 2:<br>  ○ Permanently discontinue 5-FU bolus<br>  ○ Resume 5-FU infusion and either oxaliplatin or irinotecan at one lower dose level<br>  ○ Held doses will not be made up |
| Grade 4 mucositis | • Hold all study treatment<br>• If mucositis resolves to ≤Grade 2:<br>  ○ Permanently discontinue 5-FU bolus<br>  ○ Resume 5-FU infusion at two lower dose levels<br>  ○ Resume either oxaliplatin or irinotecan at one lower dose level<br>  ○ Resume encorafenib, bevacizumab and/or cetuximab at the prior dose<br>  ○ Held doses will not be made up |
| Grade 3 nausea or vomiting | If they persist/occur despite two treatments with adequate (combination) antiemetic therapy, reduce oxaliplatin or irinotecan one dose level for the remainder of study treatment |
| Grade 4 vomiting | If they persist/occur despite two treatments with adequate (combination) antiemetic therapy, reduce 5-FU (bolus and/or infusion) and either oxaliplatin or irinotecan one dose level for the remainder of study treatment |
| ***Pulmonary toxicities*** | |
| Grade 3 cough, dyspnea, hypoxia, pneumonitis, or pulmonary infiltrates | • Hold oxaliplatin and bevacizumab (and cetuximab) until interstitial lung disease is ruled out<br>• Continue 5-FU (bolus and infusion)<br>• Permanently discontinue all protocol therapy if symptomatic, clinically relevant interstitial lung disease is confirmed |
| ***Neurotoxicities*** | |
| Grade 2 Paresthesias/dysesthesias | For Grade 2 neurotoxicity ***persisting*** between treatments:<br>• Consider reducing oxaliplatin by one dose level<br>• **Participants should continue to receive other protocol therapy** |



090177e1a02cb071\Approved\Approved On: 14-Mar-2024 15:31 (GMT)

## Table 23. Recommended Oxaliplatin, Irinotecan and 5-FU Dose Modifications

| Severity of Adverse Reaction[a] | Recommended Dose Modifications |
|---|---|
| Grade 3 Paresthesias/dysesthesias | For Grade 3 neurotoxicity resolving to ≤Grade 2 between treatments:<br>• Reduce oxaliplatin by one dose level<br>• If further dose reduction is required for neurotoxicity beyond the third dose reduction of oxaliplatin, permanently discontinue oxaliplatin<br>• **Participants should continue to receive other protocol therapy**<br><br>For Grade 3 neurotoxicity ***persisting*** between treatments:<br>• Permanently discontinue oxaliplatin<br>• **Participants should continue to receive other protocol therapy** |
| Grade 4 Paresthesias/dysesthesias | • Permanently discontinue oxaliplatin<br>• **Participants should continue to receive other protocol therapy** |
| Pharyngo-laryngeal dysesthesia | • Increase the duration of oxaliplatin infusion up to 6 hours for all subsequent treatments<br>• Monitor oxygen saturation via a pulse oximeter; if normal, an anxiolytic agent may be given and observe the participant in the clinic until the episode has resolved<br>• Once resolved, continue protocol therapy if appropriate |
| ***Extravasation*** | • Extravasation of oxaliplatin has been associated with necrosis. Stop oxaliplatin infusion and administer at another site<br>• Treat extravasation according to institutional guidelines |
| ***Cardiovascular toxicities*** | |
| Grade 4 or recurrent/worsening venous thromboembolic events (including pulmonary embolism) -OR-<br>Grade 3 cardiac ischemia/infarction -OR-<br>Grade 4 arterial thrombotic event -OR-<br>Grade 4 LV dysfunction | Discontinue all protocol therapy including encorafenib and cetuximab. Re-initiation of any study treatment should be based on Investigator judgement and following discussion with the Sponsor. |



090177e1a02cb071\Approved\Approved On: 14-Mar-2024 15:31 (GMT)

**Table 23. Recommended Oxaliplatin, Irinotecan and 5-FU Dose Modifications**

| Severity of Adverse Reaction[a] | Recommended Dose Modifications |
|---|---|
| QTcF >500 ms | • Perform regular ECG monitoring (continuous where appropriate) until an adequately trained physician (such as a cardiologist or internist) has reviewed the data<br>• Correct electrolyte abnormalities (including magnesium) and control cardiac risk factors for QT prolongation (e.g, congestive heart failure, bradyarrhythmias)<br>• Permanently discontinue oxaliplatin |
| ***Hypersensitivity reactions*** | |
| Grade 1 | • Decrease the infusion rate of the suspect agent by 50% until symptoms resolve, then resume at the initial planned rate |
| Grade 2 | • Stop infusion of the suspect agent<br>• Administer $H_1$ and/or $H_2$ blockers, and/or steroids according to institutional policy<br>• In case of anaphylactic manifestations, permanently discontinue oxaliplatin and notify the Sponsor<br>• Restart other infusions when symptoms resolve and pretreat before all subsequent doses |
| Grade 3 or 4 | • Stop infusion of the suspect agent<br>• Permanently discontinue the suspect agent and notify the Sponsor |
| ***Other AEs*** | |
| ≥Grade 3 | • Hold 5-FU (bolus and infusion) and either oxaliplatin or irinotecan<br>• Monitor toxicity at least weekly; if toxicity resolves to ≤Grade 1 within 4 weeks, resume treatment at one lower dose level<br>• Consider reducing or discontinuing 5-FU bolus before reducing 5-FU infusion dose |

a. NCI CTCAE version 4.03

Recommended dose modifications for capecitabine treatment-related AEs are summarized in Table 24. Please always refer to the locally approved product label for any adverse reaction not specifically mentioned in the table and for the most updated recommendations. In the Phase 3 Control Arm, if capecitabine is discontinued from the CAPOX regimen, treatment with oxaliplatin may continue.



090177e1a02cb071\Approved\Approved On: 14-Mar-2024 15:31 (GMT)

**Table 24. Recommended Capecitabine Dose Modifications**

| Toxicity NCIC Grades | During a Course of Therapy |
|---|---|
| **Grade 1** | Maintain dose level |
| **Grade 2** | |
| 1st occurrence | Interrupt until resolved to Grade 0-1; resume treatment at original dose level |
| 2nd occurrence | Interrupt until resolved to Grade 0-1, resume treatment at one lower dose level |
| 3rd occurrence | Interrupt until resolved to Grade 0-1, resume treatment at two lower dose levels |
| 4th occurrence | Discontinue treatment permanently |
| **Grade 3** | |
| 1st occurrence | Interrupt until resolved to Grade 0-1, resume treatment at one lower dose level |
| 2nd occurrence | Interrupt until resolved to Grade 0-1, resume treatment at two lower dose levels |
| 3rd occurrence | Discontinue treatment permanently |
| **Grade 4** | |
| 1st occurrence | Discontinue permanently<br>*OR*<br>If investigator deems it to be in the participant's best interest to continue, interrupt until resolved to Grade 0-1, resume treatment at two lower dose levels |
| 2nd occurrence | Discontinue permanently |

### 6.6.4. Dose Modifications for Bevacizumab

No dose reductions for bevacizumab are recommended. Participants with colon or rectal stents should not receive bevacizumab due to increased risk of perforation.

For participants for whom elective surgery is contemplated, bevacizumab is to be discontinued for at least 8 weeks prior to surgery. Bevacizumab may be resumed after at least 4 weeks following surgery. These participants should also discontinue aspirin at least 1 week prior to surgery.

For patients for whom non-elective surgery is required, hold bevacizumab as long as possible prior to surgery and for at least 6 weeks following surgery. Other protocol treatment may be given while bevacizumab is held at the discretion of the treating physician.

Recommended dose modifications for bevacizumab based on the occurrence of bevacizumab treatment-related AEs are summarized in Table 25. Please always refer to the locally approved product label for any adverse reaction not specifically mentioned in the table and for the most updated recommendations.



090177e1a02cb071\Approved\Approved On: 14-Mar-2024 15:31 (GMT)

090177e1a02cb071\Approved\Approved On: 14-Mar-2024 15:31 (GMT)

### Table 25. Recommended Bevacizumab Dose Modifications

| Severity of Adverse Reaction[a] | Dose Modification for Bevacizumab |
|---|---|
| ***Infusion Reactions*** | |
| If a reaction occurs, the infusion should be discontinued and appropriate medical therapies should be administered. Systemic premedication is not warranted. | |
| ***Reversible Posterior Leukoencephalopathy Syndrome (RPLS)*** | |
| For signs and symptoms suggestive of RPLS (eg, confusion, headache, seizures, cortical blindness) interrupt bevacizumab. Suspected RPLS should be investigated with MRI. If diagnosis of RPLS is confirmed, bevacizumab should be permanently discontinued. | |
| Grade 3 | If RPLS is ruled out via MRI, the decision on resuming bevacizumab should be based on the nature of the signs/symptoms. For grade 3 events, bevacizumab may be resumed if toxicities completely resolve within 4 weeks. |
| Grade 4 | If RPLS is ruled out via MRI, the decision on resuming bevacizumab should be based on the nature of the signs/symptoms. For grade 4 events with likely relationship to bevacizumab, discontinue bevacizumab. |
| ***Hypertension*** | |
| Grade 3 | Hold bevacizumab if not controlled with medical management; resume once controlled. Consider administering the following antihypertensive therapies in order of preference: calcium antagonists, ACE inhibitors, diuretics, and beta-blockers |
| Grade 4 | Permanently discontinue bevacizumab |
| ***Wound Healing Complications*** | |
| **Wound healing complications requiring medical intervention** | Permanently discontinue bevacizumab |
| Necrotizing fasciitis | Permanently discontinue bevacizumab |
| ***Posterior Reversible Encephalopathy Syndrome (PRES)*** | |
| Any Grade | Permanently discontinue bevacizumab |
| ***Hemorrhage*** | |
| Grade ≥2 pulmonary or CNS | Permanently discontinue bevacizumab |

### Table 25. Recommended Bevacizumab Dose Modifications

| Severity of Adverse Reaction[a] | Dose Modification for Bevacizumab |
|---|---|
| Grade 3 or 4 non-pulmonary and non-CNS | Permanently discontinue bevacizumab |
| ***Venous thrombosis*** | |
| Grade 3 or 4 | Permanently discontinue bevacizumab |
| ***Arterial thromboembolic event*** | |
| Any Grade | Permanently discontinue bevacizumab |
| ***Congestive heart failure*** | |
| Symptomatic or NYHA Class ≥2 heart failure | Permanently discontinue bevacizumab |
| ***Renal Injury and Proteinuria*** | |
| Nephrotic syndrome | Permanently discontinue bevacizumab |
| ***GI perforation*** | |
| Any Grade | Permanently discontinue bevacizumab |
| ***Bowel obstruction*** | |
| Grade 1 | Participants who experience partial obstruction not requiring medical intervention may continue on bevacizumab |
| Grade 2 | Hold bevacizumab in participants who experience partial obstruction requiring medical intervention. Resume upon complete resolution |
| Grade 3/4 | Permanently discontinue bevacizumab |
| Wound dehiscence | Permanently discontinue bevacizumab |
| ***Pulmonary toxicities*** | |
| Grade 3 cough, dyspnea, hypoxia, pneumonitis, or pulmonary infiltrates | Hold bevacizumab until interstitial lung disease is ruled out. Permanently discontinue bevacizumab if interstitial lung disease is confirmed |
| ***Other unspecified bevacizumab-related adverse events*** | |
| Grade 3 | Hold bevacizumab until recovery to ≤Grade 1 |
| Grade 4 | Permanently discontinue bevacizumab |



090177e1a02cb071\Approved\Approved On: 14-Mar-2024 15:31 (GMT)

**Table 25. Recommended Bevacizumab Dose Modifications**

| Severity of Adverse Reaction[a] | Dose Modification for Bevacizumab |
|---|---|

a. NCI CTCAE version 4.03

### 6.6.5. Guidance For Administration of Study Intervention in Participants with SARS-CoV-2 Infection

Each individual participants case is unique and decisions surrounding study intervention should be based on the investigator's clinical judgement and in accordance with local and regional recommendations. The following modifications are general suggestions for participants with active SARS-CoV-2 infection including those that are confirmed positive based on a locally approved test or presumed positive based on clinical suspicion:

- In participants with active infection and symptoms, consider delaying study intervention for at least 14 days from start of symptoms.
- Consider restarting treatment when SARS-CoV-2-associated symptoms have recovered to ≤Grade 1 for ≥72 hours and participant has been afebrile for ≥72 hours

Additional guidance is provided in Appendix 12.

## 6.7. Intervention After the End of the Study

At the end of the study, any participants who are still deriving clinical benefit from study intervention, Sponsor will work with investigator to explore options for continued study intervention (eg, continuation study), in accordance with local regulations and requirements.

# 7. DISCONTINUATION OF STUDY INTERVENTION AND PARTICIPANT DISCONTINUATION/WITHDRAWAL

## 7.1. Discontinuation of Study Intervention

It may be necessary for a participant to permanently discontinue study intervention (definitive discontinuation). Reasons for definitive discontinuation of study intervention include the following:

- Withdrawal of consent/assent. Participants may choose to withdraw from the study at any time without penalty of jeopardizing their healthcare or loss of benefits to which the participant is otherwise entitled. Participants will have the option of withdrawing consent/assent for study treatment but continue in the follow-up period of the study for safety/efficacy assessments. See Section 7.2.1.
- Unacceptable AEs or failure to tolerate study intervention defined as:

090177e1a02cb071\Approved\Approved On: 14-Mar-2024 15:31 (GMT)

  - Grade 4 or life-threatening AE as outlined in Appendix 3, (except with approval from the medical monitor);
  - Toxicity requiring more than the allowed number of dose reductions for any study intervention as described in Section 6.6;
  - Occurrence of an AE that is related to study treatment and in the judgment of the Investigator compromises the participant's ability to continue study-specific procedures, or is considered to not be in the participant's best interest.

- Dose interruption due to an AE or clinically significant laboratory abnormality, unless judged by the Investigator and medical monitor or designee to be in the best interest of the participant to continue treatment, as follows:
  - Encorafenib: Dose interruption >28 consecutive days;
  - Cetuximab: >2 missed consecutive doses of cetuximab;
  - mFOLFOX6/FOLFIRI/FOLFOXIRI: If the initiation of a new cycle or therapy during a cycle is delayed for ≥4 weeks;
  - CAPOX: If the initiation of a new cycle or therapy during a cycle is delayed for ≥3 weeks.

- Progressive disease (defined by RECIST, v1.1; see Section 8.1.1).
  ***Note***: For the SLI, disease progression will be determined by Investigator. For the Phase 3 portion and Cohort 3, disease progression will be identified by the investigator and confirmed by BICR.
  ***Note***: In Phase 3 and Cohort 3, if disease progression is identified by Investigator (based on clinical or radiographic evidence), every effort should be made to continue study intervention until confirmed by BICR (see Section 8.1.1.1.2).
  ***Note***: Participants with disease progression who are continuing to derive clinical benefit from the study intervention per the Investigator will be eligible to continue, provided the risk/benefit for doing so is favorable and after discussion with the Sponsor.
- Participant becomes pregnant or begins breastfeeding.
- Significant protocol deviation that, in the opinion of the Investigator and/or Sponsor, renders the participant unsuitable for further study intervention administration.
- Participant is noncompliant with study procedures or study treatment that in the judgment of the Investigator or Sponsor renders the participant unsuitable for further study participation.
- Lost to follow-up.
- Death.

090177e1a02cb071\Approved\Approved On: 14-Mar-2024 15:31 (GMT)

- Termination of the study by the Sponsor.

At the time of study treatment discontinuation, an end of treatment visit should be conducted. Note that discontinuation of study intervention does not represent withdrawal from the study. If study intervention is definitively discontinued, the participant will remain in the study to be evaluated for safety, disease assessments, subsequent anticancer therapies, and survival. See the SoA for data to be collected and for any further evaluations that need to be completed at the EOT visit, 28-day follow-up visit and long-term follow-up.

In the event of discontinuation of study intervention, it must be documented on the appropriate CRF/in the medical records whether the participant is discontinuing further receipt of study intervention or also from study procedures, posttreatment study follow-up, and/or future collection of additional information.

In the event of study termination, the sponsor may work with sites to explore options for alternative source of post-study treatment for remaining participants.

After participants have discontinued treatment for any reason, they will be contacted approximately every 3 months in long-term follow-up until withdrawal of consent/assent, the participant is lost to follow-up, death, or the final OS analysis. If participants withdraw consent/assent for study intervention they will continue to be contacted for survival status unless they specifically request that they not be contacted. If the participant refuses to be contacted, attempts to determine survival status should be made via access to public records where permitted by local laws.

When a participant discontinues treatment for "disease progression" without BICR confirmation, it will not be counted as a PFS event and disease assessments will continue to be performed until PD is determined by BICR (see Section 8.1.1).

## 7.2. Participant Discontinuation/Withdrawal From the Study

A participant may withdraw from the study at any time at his/her own request. Reasons for discontinuation from the study include the following:

- Refused further follow-up (ie, withdrawal of consent/assent);
- Lost to follow-up;
- Death;
- Study terminated by sponsor;
- Termination of the study by the local health authority, IRB or IEC.

If a participant withdraws from the study, he/she may request destruction of any remaining samples taken and not tested, and the investigator must document any such requests in the site study records and notify the sponsor accordingly.



090177e1a02cb071\Approved\Approved On: 14-Mar-2024 15:31 (GMT)

If the participant withdraws from the study and also withdraws consent/assent (see Section 7.2.1) for disclosure of future information, no further evaluations should be performed and no additional data should be collected. The sponsor may retain and continue to use any data collected before such withdrawal of consent/assent.

Lack of completion of all or any of the withdrawal/early termination procedures will not be viewed as protocol deviations so long as the participant's safety was preserved.

### 7.2.1. Withdrawal of Consent/Assent

Participants who request to discontinue receipt of study intervention will remain in the study and must continue to be followed for protocol-specified follow-up procedures. The only exception to this is when a participant specifically withdraws consent/assent for any further contact with him or her or persons previously authorized by the participant to provide this information. Participants should notify the investigator in writing of the decision to withdraw consent/assent from future follow-up, whenever possible. The withdrawal of consent/assent should be explained in detail in the medical records by the investigator, as to whether the withdrawal is only from further receipt of study intervention or also from study procedures and/or posttreatment study follow-up, and entered on the appropriate CRF page. In the event that vital status (whether the participant is alive or dead) is being measured, publicly available information should be used to determine vital status only as appropriately directed in accordance with local law.

## 7.3. Lost to Follow-up

A participant will be considered lost to follow-up if he or she repeatedly fails to return for scheduled visits and is unable to be contacted by the study site.

The following actions must be taken if a participant fails to return to the clinic for a required study visit:

- The site must attempt to contact the participant and reschedule the missed visit as soon as possible and counsel the participant on the importance of maintaining the assigned visit schedule and ascertain whether or not the participant wishes to and/or should continue in the study;
- Before a participant is deemed lost to follow-up, the investigator or designee must make every effort to regain contact with the participant (where possible, 3 telephone calls and, if necessary, a certified letter to the participant's last known mailing address or local equivalent methods). These contact attempts should be documented in the participant's medical record;
- Should the participant continue to be unreachable, he/she will be considered to have withdrawn from the study.

090177e1a02cb071\Approved\Approved On: 14-Mar-2024 15:31 (GMT)

# 8. STUDY ASSESSMENTS AND PROCEDURES

The investigator (or an appropriate delegate at the investigator site) must obtain a signed and dated ICD before performing any study-specific procedures.

Study procedures and their timing are summarized in the SoA. Protocol waivers or exemptions are not allowed.

Safety issues should be discussed with the sponsor immediately upon occurrence or awareness to determine whether the participant should continue or discontinue study intervention (see Section 10.1.1.1).

Adherence to the study design requirements, including those specified in the SoA, is essential and required for study conduct.

All screening evaluations must be completed and reviewed to confirm that potential participants meet all eligibility criteria. The investigator will maintain a screening log to record details of all participants screened and to confirm eligibility or record reasons for screening failure, as applicable.

Procedures conducted as part of the participant's routine clinical management (eg, blood count) and obtained before signing of the ICD may be utilized for screening or baseline purposes provided the procedures met the protocol-specified criteria and were performed within the time frame defined in the SoA.

Every effort should be made to ensure that protocol-required tests and procedures are completed as described. However, it is anticipated that from time to time there may be circumstances outside the control of the investigator that may make it unfeasible to perform the test. In these cases, the investigator must take all steps necessary to ensure the safety and well-being of the participant. When a protocol-required test cannot be performed, the investigator will document the reason for the missed test and any corrective and preventive actions that he or she has taken to ensure that required processes are adhered to as soon as possible. The study team must be informed of these incidents in a timely manner.

For samples being collected and shipped, detailed collection, processing, storage, and shipment instructions and contact information will be provided to the investigator site prior to initiation of the study.

## 8.1. Efficacy Assessments

### 8.1.1. Tumor Response Assessments

Tumor responses will be evaluated according to RECIST, v1.1 (Appendix 9). Measurable disease will be based on local Investigator assessment of baseline scans. Baseline scans should be sent to the BICR within 30 days of randomization. For the SLI portion of the study, tumor responses will be evaluated locally by the Investigator and may be transmitted to an imaging vendor for BICR review. For the Phase 3 and Cohort 3 portions of the study, tumor responses will be evaluated locally by the Investigator and centrally by BICR.



090177e1a02cb071\Approved\Approved On: 14-Mar-2024 15:31 (GMT)

All potential sites of tumor lesions will be assessed at the time points specified in Table 1 (Screening), Table 2 (SLI), Table 3 (Phase 3 Arm A, Arm B and Cohort 3 Arm D ), Table 4 (Phase 3 Arm C and Cohort 3 Arm E), and Table 5 (Phase 3 Arm C, CAPOX with or without bevacizumab):

At screening (prior to randomization for participants in the Phase 3 portion and Cohort 3), the following should be performed:

- A CT scan with IV contrast of chest, abdomen and pelvis is the preferred technique. If there is concern about radiation exposure, an MRI may be used instead of a CT.
- In participants with a history of asymptomatic brain metastases, a brain MRI or CT scan
- If clinically indicated, a whole body bone scan (ie, if bone metastases are suspected or known at baseline). Sites may use a whole body bone imaging method per their local standard of care (eg, Tc99m bone scan, FDG-PET, NaF PET scan or whole-body bone MRI). Skeletal lesions identified on a whole body bone scan at baseline, which are not visible on the chest, abdomen, or pelvis CT (or MRI) scan should be imaged at baseline using localized CT, MRI, or X-ray. Skeletal target lesions identified at baseline should continue to be imaged at subsequent scheduled visits using localized CT, MRI, or X-ray.

Every effort must be made to assess each lesion that is identified at screening by the same method throughout the study so that the comparison is consistent.

The first post-baseline scan must be performed 42 to 49 days from the date of first dose or from randomization for Phase 3. All post-screening assessments should be performed every 6 weeks from the date of randomization (or from the first dose for SLI ) for the first 18 months of treatment, then every 8 weeks thereafter until BICR-confirmed disease progression (regardless of new anticancer therapy for Phase 3 and Cohort 3; for SLI confirmation by BICR is not required), withdrawal of consent/assent, participant is lost to follow-up, death, or the final OS analysis.

Regardless of whether study treatment is discontinued, the following should be performed:

- Chest, abdomen, and pelvis CT (or MRI) scans
- Brain MRI or CT scan, if metastases were documented at baseline
- Skeletal target lesions identified at baseline should continue to be imaged at subsequent scheduled visits using localized CT, MRI, or X-ray (using the same method used at baseline for all visits for any given lesion). After baseline, whole body bone scans need not be repeated, unless clinically indicated.



090177e1a02cb071\Approved\Approved On: 14-Mar-2024 15:31 (GMT)

- Additional imaging evaluations may be performed if there is symptomatic evidence suggesting the possibility of disease progression based on clinical symptoms or physical examination at any time.

If off-schedule imaging evaluations are performed or if progression is suspected, every effort should be made to perform subsequent imaging evaluations in accordance with the original imaging schedule.

All CT scans should be performed with IV contrast. If a participant is known to have a medical contraindication to the contrast agent or develops a contraindication during the study, a CT scan without IV contrast of the chest and MRI with IV contrast, if possible, of the abdomen and pelvis may be performed. A CT scan of the brain, with IV contrast, if possible, may be performed if MRI is contra-indicated.

Chest X-ray or ultrasound should not be used for tumor response assessments in this study.

Any lesions that have been subjected to loco-regional therapies (eg, radiotherapy, ablation, etc) should be considered non-measurable, unless they have clearly progressed since the therapy. Previously treated lesions that have not progressed should be considered non-measurable and therefore, assessed as non-target lesions.

While FDG-PET scans are not required for this study, sites may perform combined PET/CT scans per their local standard of care, provided the CT is of similar diagnostic quality as CT performed without PET, including the use of oral and IV contrast media. If acquired according to local standard of care, FDG-PET may be relied upon to document PD in accordance with RECIST.

Preferably, each center should preferably have a designated radiologist responsible for the interpretation of scans and response evaluations for study participants. At a minimum, a single radiologist will perform all evaluations for an individual participant.

#### 8.1.1.1. BICR Evaluation of Imaging Data

All imaging data acquired in the study for efficacy purposes (eg, CT/MRI scans) will be transmitted to an imaging vendor for BICR review. These images will be read by readers who are blinded to treatment assignment and to other clinical data as specified in the BICR Charter. Image transmission to the imaging vendor should be performed according to the Imaging Vendor Manual.

##### 8.1.1.1.1. Time Points without Locally Determined Progression

Tumor measurements and/or assessments for time points with no locally determined progression received by the BICR will be read on an ongoing basis (rolling reads) in Phase 3 and Cohort 3 as detailed in the Imaging Manual. BICR interpretation of these data will not be communicated to the site, even if progression is assessed by the BICR (please see below).



090177e1a02cb071\Approved\Approved On: 14-Mar-2024 15:31 (GMT)

#### 8.1.1.1.2. Time Points with Locally Determined Progression

All participants in Phase 3 and Cohort 3 who have PD determined by the local Investigator require an expedited tumor response review by the BICR, and sites must send the radiological data to the BICR immediately (within 2 business days) upon acquisition. The Investigator seeking an expedited review must indicate this request on the radiology transmittal form. The imaging vendor will ensure that the central reviewers involved in the BICR are blinded to the expedited status of the reading.

The BICR will perform the review of radiological data and the results of the reading will be communicated to the site within 5 business days from BICR confirmation on acceptable image receipt.

While the Investigator is waiting for the results from the BICR, every effort should be made to continue administration of study intervention. However, during this time, the Investigator should do whatever is medically necessary for the participant.

If the BICR determines PD, the study site will be informed and the participant will discontinue study intervention, cease tumor assessments and begin follow-up assessments as outlined in the SoA. If according to the Investigator's clinical judgment, a participant with evidence of PD is still experiencing clinical benefit, the participant may be eligible for continued treatment after discussion between the Investigator and the Sponsor.

If the BICR does not conclude PD, the study site will be informed and the participant should continue receiving study intervention unless clinically indicated (eg, rapid clinical deterioration) for an immediate change in therapy. Participants will continue to have tumor assessments performed every 6 weeks from randomization for the first 18 months, then every 8 weeks for the remainder of the study, irrespective of the days of study intervention administration, until the BICR declares PD, withdrawal of consent/assent, lost to follow-up or death.

In summary, for expedited time points (time points assessed as PD locally by the Investigator):

- Sites must send the radiological and/or pathological data to the BICR immediately (within 2 business days) upon acquisition. While the Investigator is waiting for the results from the BICR, it is preferable that the participant continue treatment with study intervention.

- If the BICR determines PD, the study site will be informed. This participant will then discontinue study intervention, cease tumor assessments and begin follow-up assessments. If according to the Investigator's clinical judgment, a participant with evidence of PD is still experiencing clinical benefit, the participant may be eligible for continued treatment after discussion between the Investigator and the Sponsor.



090177e1a02cb071\Approved\Approved On: 14-Mar-2024 15:31 (GMT)

- If the BICR does not conclude PD, the study site will be informed. As long as it is clinically acceptable, every effort should be made to continue the participant on study intervention until the BICR declares PD.

### 8.1.2. Patient-reported Outcomes

Cancer-specific global health status and quality of life, functioning, and symptoms data will be collected using the EORTC QLQ-C30 questionnaire and general health status will be assessed using the EQ-5D-5L health questionnaire. PGIS and PGIC will also be collected (See Appendix 10) according to FDA guidance[79] to enable the implementation of anchor-based methods to establish meaningful change in PROs.

These questionnaires will be administered at the time points specified in Table 3 (Arm A, Arm B and Cohort 3 Arm D ), and Table 4 (Phase 3 Arm C and Cohort 3 Arm E) and Table 5 (Phase 3 Arm C, CAPOX with or without bevacizumab). These questionnaires will be used to explore PRO measures of health-related QoL, functioning, cancer symptoms, and treatment-related side effects.

The EORTC QLQ-C30 is a reliable and valid self-administered questionnaire used in oncology trials.[80] The questionnaire is easily understood by most participants and is quick to complete (mean time 11 minutes).

It contains 30 items and is composed of both multi-item scales and single-item measures. These include five functional scales (physical, role, emotional, cognitive and social functioning), three symptom scales (fatigue, nausea/vomiting, and pain), six single items (dyspnea, insomnia, appetite loss, constipation, diarrhea and financial impact) and a global health status/QoL scale. All the scales and single-item measures range in score from 0 to 100.

Higher scores on the functional scales represent higher levels of functioning. Higher scores on the global health status/quality of life scale represent higher health status/quality of life. Higher scores on symptom scales/items represent a greater presence of symptoms.

The EQ-5D-5L is a standardized measure of health utility that provides a single index value for the participant's health status. It is frequently used for economic evaluations of health care and has been shown to be a valid and reliable instrument, and comprises a short descriptive system questionnaire and a visual analogue scale (EQ VAS) that are cognitively undemanding, taking about 2 minutes to complete.

The EQ-5D-5L contains one item for each of five dimensions of health (ie, mobility, self-care, usual activities, pain or discomfort, and anxiety or depression). Response options for each item vary from having no problems (eg, "...no problems walking about"), moderate problems (e.g., "...some problems walking about"), or extreme problems (eg, "...unable to walk about"). Participant responses to the five dimensions reflect a specific health state that corresponds to a population preference weight for that state on a continuous scale of 0 (death) to 1 (perfect health).



090177e1a02cb071\Approved\Approved On: 14-Mar-2024 15:31 (GMT)

The visual analog scale (ranging from 0 to 100) is included to capture participant's rating of their overall health status. Higher scores of the EQ-5D-5L represent better health states. All scoring and handling of data will follow the User's Guide defined by the EuroQoL Group.

The PGIS is a single-item questionnaire designed to assess participant's overall impression of disease severity at a given point in time. The PGIC is a single-item questionnaire designed to assess the participant's overall sense of whether there has been a change in symptoms or QoL since starting treatment. The PGIS and PGIC can be employed as anchors in responder analyses of other PRO instruments, to help interpret clinically meaningful changes in scores.

The questionnaires should be administered to participants in their preferred language at the beginning of the study visit prior to receiving any study treatment, prior to any other study assessment or consultation with the Investigator, and prior to being informed of their current disease status. Participants should be given sufficient space and time to complete the study questionnaires.

Attempts should be made to collect all questionnaires for all participants, including those who discontinue prior to the EOT visit. However, if the participant refuses to complete the questionnaires, this should be documented in study source records. Participant refusal to complete study questionnaires is not a protocol deviation.

### 8.1.3. Survival Follow-up

Participants who have discontinued all study intervention (for any reason) will be contacted (eg, telephone, email, site visits, family member contact) approximately every 3 months for survival status and initiation of subsequent anticancer therapies/dates of progression, until death, withdrawal of consent, lost to follow-up, or the final OS analysis, whichever occurs first. If participants withdraw consent for study intervention, they will continue to be contacted for survival status unless they specifically request that they are not to be contacted. If the participant refuses to be contacted, attempts to determine survival status should be made via access to public records where permitted by local laws.

Upon the final OS analysis in each portion of the study (SLI, Phase 3 and Cohort 3), as specified in Section 9.4.3.2, no further contact for survival status will occur for participants enrolled in the specific study portion in the long-term survival follow-up stage. In addition, for participants remaining on study intervention, the sponsor will work with sites to explore options for alternative sources of post-study study intervention (see Section 6.7) until all patients are discontinued from study intervention.

## 8.2. Safety Assessments

Planned time points for all safety assessments are provided in the SoA. Unscheduled clinical laboratory measurements may be obtained at any time during the study to assess any perceived safety issues.

Medical history, physical examination, and neurological examination information, as applicable, collected during the course of the study will be considered source data only and



090177e1a02cb071\Approved\Approved On: 14-Mar-2024 15:31 (GMT)

will not be required to be reported on the case report form, unless otherwise noted. However, any untoward findings identified on physical and/or neurological examinations conducted during the active collection period will be captured as AEs, if those findings meet the definition of an AE. Data collected at screening that are used for inclusion/exclusion criteria, such as laboratory data, ECGs, and vital signs, as well as demographic data collected at screening will be reported.

### 8.2.1. Participant Demographics and Other Baseline Characteristics

Demographic data will be collected at Prescreening and general medical history will be collected at Screening by the Investigator or qualified designee and will include relevant medical and surgical history within the last 10 years and current illnesses.

### 8.2.2. Disease Characteristics and Treatment History

A disease-targeted medical and treatment history will be collected at Screening. Details regarding the participant's malignancy under study, including date and stage at initial diagnosis, date and extent of metastatic disease at study entry, tumor histology, relevant disease characteristics and prior treatments, including systemic, radiation and surgical procedures, will be recorded.

### 8.2.3. Physical Examinations

At Screening, the physical examination should be comprehensive and include the examination of general appearance, skin, neck (including thyroid), eyes, ears, nose, throat, lungs, heart, abdomen, back, lymph nodes, extremities, vascular and neurological. Participants should be assessed at each visit for symptoms of new or worsening visual disturbance. If symptoms of new or worsening visual disturbances including diminished central vision, blurred vision or loss of vision are identified, these should be recorded as adverse events and a prompt ophthalmologic examination is recommended. If indicated based on medical history and/or symptoms, rectal, external genitalia, breast and pelvic examinations will be performed. Body weight will be measured as part of the physical examination on Day 1 of each cycle and on Day 15 during 28-day cycle regimens. For participants ≥18 years old, height will be measured only at Screening. For participants <18 years old, height will be measured at Screening and Day 1 of each cycle.

For subsequent visits, the physical examinations should be targeted as clinically indicated. Investigators should pay special attention to clinical signs related to previous serious illnesses.

All physical examinations occurring on dosing days must be performed prior to study intervention administration. Any treatment-emergent abnormal findings will be recorded as AEs.

### 8.2.4. Body Surface Area

BSA will be calculated at the start of each cycle. The most recent participant weight available should be used to calculate BSA in order to determine the appropriate dose for that cycle. If



090177e1a02cb071\Approved\Approved On: 14-Mar-2024 15:31 (GMT)

the participant's weight at the beginning of each cycle varies by >10% from the previous cycle, the dose must be recalculated.

In calculating the BSA, actual height and weight should be used. In participants with BSA >2.2 $m^2$, either actual BSA or 2.2 $m^2$ may be used. As per ASCO guidelines, any of the established formulas may be used to calculate BSA.[81] A single formula should be used consistently throughout the study for a given participant.

### 8.2.5. Dermatological Examinations

Dermatologic evaluations will be performed at the site by the Investigator to monitor for the possible development of keratoacanthoma and/or squamous cell carcinoma, as these have been reported to occur with selective BRAF inhibitor treatment.[82-84] This assessment may be performed predose or postdose at the time points specified in the SoA. Following the 28-day follow-up, dermatologic examinations for skin malignancies are recommended every 2 months until 6 months after the last dose of encorafenib or until initiation of another antineoplastic therapy. Participants should be referred to a dermatologist for any abnormalities identified on the Investigator's dermatologic examination.

### 8.2.6. Vital Signs

Temperature, pulse rate, respiratory rate, and blood pressure will be assessed per institutional standards prior to blood collection for laboratory tests (when timing is feasible).

All vital sign measurements occurring on dosing days must be performed prior to study intervention administration. Abnormal vital sign results identified after the first dose of study treatment constitute an AE if they are considered clinically meaningful, induce clinical signs or symptoms, require concomitant therapy or require changes in study intervention/treatment.

### 8.2.7. Electrocardiograms

Standard 12 lead ECGs utilizing limb leads (with a 10 second rhythm strip) should be collected at times specified in the SoA using an ECG machine that automatically calculates the heart rate and measures PR, QT, and QTcF intervals and QRS complex. For ECG machines that do not report QTcF, calculation of QTcF from QT and heart rate, for example using online tools, is required. Alternative lead placement methodology using torso leads (eg, Mason-Likar) should not be used given the potential risk of discrepancies with ECGs acquired using standard limb lead placement. All scheduled ECGs should be performed after the participant has rested quietly for at least 5 minutes in a supine position.

A triplicate ECG (3 serial ECGs conducted within approximately 5 to 10 minutes total time) will be performed at screening to determine eligibility and predose on Cycle 1 Day 1 before administration of any study intervention to serve as baseline. A single ECG will be performed at all remaining time points specified in the SoA. The oxaliplatin SmPc notes that the QT interval should be monitored on a regular basis before and after administration of oxaliplatin. If single ECG reveals QTc abnormality, continuous ECG should be performed as noted below. All scheduled ECGs should be performed after the participant has rested quietly



090177e1a02cb071\Approved\Approved On: 14-Mar-2024 15:31 (GMT)

for at least 5 minutes in a supine position. When an ECG is to be performed at the same time point as a blood collection, the ECG is to be performed first.

A study participant should undergo continuous ECG monitoring until an adequately trained physician (ie, cardiologist or internist) has reviewed the data if:

a. a postdose QTcF interval remains ≥60 msec from the baseline **and** is >450 msec; or

b. an absolute QTcF value is ≥500 msec for any scheduled ECG for greater than 4 hours (or sooner, at the discretion of the investigator); or

c. QTcF intervals get progressively longer by 30 ms increments.

Caution should be exercised in patients with a history or a predisposition for prolongation of QT, those who are taking medicinal products known to prolong QT interval, and those with electrolyte disturbances such as hypokalaemia, hypocalcaemia, or hypomagnesaemia. In case of QT prolongation, oxaliplatin treatment should be discontinued.

Consider consulting a cardiologist if QTcF intervals do not return to less than the criteria listed above after 8 hours of monitoring (or sooner, at the discretion of the investigator). New or worsened clinically significant findings occurring after the informed consent/assent must be recorded as an AE in the eCRF. ECG tracings should be made available if requested by the Sponsor.

In some cases, it may be appropriate to repeat abnormal ECGs to rule out improper lead placement as contributing to the ECG abnormality. It is important that leads be placed in the same positions each time in order to achieve precise ECG recordings. If a machine-read QTcF value is prolonged, as defined above, repeat measurements may not be necessary if a qualified medical provider's interpretation determines that the QTcF values are in the acceptable range.

ECG values of potential clinical concern are listed in Appendix 7.

### 8.2.8. Clinical Safety Laboratory Assessments

See Appendix 2 for the list of clinical safety laboratory tests to be performed and the SoA for the timing and frequency. All protocol-required laboratory assessments, as defined in Appendix 2, must be conducted in accordance with the laboratory manual and the SoA. Unscheduled clinical laboratory measurements may be obtained at any time during the study to assess any perceived safety issues.

The investigator must review the laboratory report, document this review, and record any clinically relevant changes occurring during the study in the AE section of the CRF. Clinically significant abnormal laboratory findings are those which are not associated with the underlying disease, unless judged by the investigator to be more severe than expected for the participant's condition.



090177e1a02cb071\Approved\Approved On: 14-Mar-2024 15:31 (GMT)

All laboratory tests with values considered clinically significantly abnormal during participation in the study or within 28 days after the last dose of study intervention should be repeated until the values return to normal or baseline or are no longer considered clinically significant by the investigator or medical monitor.

If such values do not return to normal/baseline within a period of time judged reasonable by the investigator, the etiology should be identified and the sponsor notified.

See Appendix 6 for suggested actions and follow-up assessments in the event of potential drug-induced liver injury.

At Screening, laboratory results from the central laboratory should be used by the Investigator to determine the participant's eligibility for the study. If a particular central laboratory result is not available, a local laboratory test result for that particular analyte may be utilized to determine a participant's eligibility to participate in the study. Local laboratory test results may also be used at baseline and when results are time-sensitive (eg, for participant safety or adjustment of study intervention dose or schedule). Part of the sample obtained for local laboratory analysis should be sent to the central laboratory for analysis. Local laboratory results obtained during the study will not be captured in the eCRF unless they are needed to clarify why a treatment decision was made or an AE was recorded.

Additional unscheduled clinical laboratory tests may be obtained at any time during the study at the Investigator's discretion. In addition to local testing, these samples should also be sent to the central laboratory for analysis when feasible.

### 8.2.9. Pregnancy Testing

Pregnancy tests may be urine or serum tests (serum required at Screening), but must have a sensitivity of at least 25 mIU/mL. Pregnancy tests will be performed in WOCBP at the times listed in the SoA. Following a negative pregnancy test result at screening, appropriate contraception must be commenced and a second negative pregnancy test result will be required at the baseline visit prior the participant's receiving the study intervention. Pregnancy tests will also be done whenever 1 menstrual cycle is missed during the active treatment period (or when potential pregnancy is otherwise suspected). Pregnancy tests may also be repeated if requested by IRBs/ethics committees or if required by local regulations. If a urine test cannot be confirmed as negative (eg, an ambiguous result), a serum pregnancy test is required. In such cases, the participant must be permanently discontinued from all study intervention if the serum pregnancy result is positive.

### 8.2.10. ECOG Performance Status

Assessment of ECOG PS (Table 26) will be assessed at the time points specified in Table 1 (Screening), Table 2 (SLI), Table 3 (Arm A, Arm B and Cohort 3 Arm D), Table 4 (Phase 3 Arm C and Cohort 3 Arm E) and Table 5 (Phase 3 Arm C, CAPOX with or without bevacizumab).

ECOG PS should be obtained on the scheduled day, even if study treatment is being held.



090177e1a02cb071\Approved\Approved On: 14-Mar-2024 15:31 (GMT)

**Table 26. Eastern Cooperative Oncology Group (ECOG) Performance Status Scale**

| | |
|---|---|
| 0 | Fully active, able to carry on all predisease performance without restriction. |
| 1 | Restricted in physically strenuous activity but ambulatory and able to carry out work of a light or sedentary nature, eg, light housework, office work. |
| 2 | Ambulatory and capable of all self-care but unable to carry out any work activities. Up and about more than 50% of waking hours. |
| 3 | Capable of only limited self-care, confined to bed or chair more than 50% of waking hours. |
| 4 | Completely disabled. Cannot carry on any self-care. Totally confined to bed or chair. |
| 5 | Dead |

## 8.3. Adverse Events and Serious Adverse Events

The definitions of an AE and an SAE can be found in Appendix 3.

AEs will be reported by the participant (or, when appropriate, by a caregiver, surrogate, or the participant's legally authorized representative).

The investigator and any qualified designees are responsible for detecting, documenting, and recording events that meet the definition of an AE or SAE and remain responsible to pursue and obtain adequate information both to determine the outcome and to assess whether the event meets the criteria for classification as an SAE or caused the participant to discontinue the study intervention (see Section 7.1).

Each participant will be questioned about the occurrence of AEs in a nonleading manner.

In addition, the investigator may be requested by Pfizer Safety to obtain specific follow-up information in an expedited fashion.

### 8.3.1. Time Period and Frequency for Collecting AE and SAE Information

The time period for actively eliciting and collecting AEs and SAEs ("active collection period") for each participant begins from the time the participant provides informed consent/assent, which is obtained before the participant's participation in the study (ie, before undergoing any study-related procedure and/or receiving study intervention), through and including a minimum of 28 calendar days, except as indicated below, after the last administration of the study intervention. Beyond 28 days after the last dose of study treatment, only SAEs considered related to study treatment should be collected.

Follow-up by the investigator continues throughout and after the active collection period and until the AE or SAE or its sequelae resolve or stabilize at a level acceptable to the investigator and Pfizer concurs with that assessment.

For participants who are screen failures, the active collection period ends when screen failure status is determined.



090177e1a02cb071\Approved\Approved On: 14-Mar-2024 15:31 (GMT)

If the participant withdraws from the study and also withdraws consent/assent for the collection of future information, the active collection period ends when consent/assent is withdrawn.

If a participant definitively discontinues or temporarily discontinues study intervention because of an AE or SAE, the AE or SAE must be recorded on the CRF and the SAE reported using the CT SAE Report Form.

Investigators are not obligated to actively seek AEs or SAEs after the participant has concluded study participation. However, if the investigator learns of any SAE, including a death, at any time after a participant has completed the study, and he/she considers the event to be reasonably related to the study intervention, the investigator must promptly report the SAE to Pfizer using the CT SAE Report Form.

#### 8.3.1.1. Reporting SAEs to Pfizer Safety

All SAEs occurring in a participant during the active collection period as described in Section 8.3.1 are reported to Pfizer Safety on the CT SAE Report Form immediately upon awareness and under no circumstance should this exceed 24 hours, as indicated in Appendix 3. The investigator will submit any updated SAE data to the sponsor within 24 hours of it being available.

If a participant begins a new anticancer therapy, SAEs occurring during the above-indicated active collection period must still be reported to Pfizer Safety irrespective of any intervening treatment. Note that a switch to a commercially available version of the study intervention is considered as a new anticancer therapy for the purposes of SAE reporting.

#### 8.3.1.2. Recording Nonserious AEs and SAEs on the CRF

All nonserious AEs and SAEs occurring in a participant during the active collection period, which begins after obtaining informed consent as described in Section 8.3.1 will be recorded in the AE section of the CRF.

The investigator is to record on the CRF all directly observed and all spontaneously reported AEs and SAEs reported by the participant.

If a participant begins a new anticancer therapy, the recording period for nonserious AEs ends at the time the new treatment is started; however, SAEs must continue to be recorded on the CRF during the above-indicated active collection period. Note that a switch to a commercially available version of the study intervention is considered as a new anticancer therapy for the purposes of SAE reporting.

### 8.3.2. Method of Detecting AEs and SAEs

The method of recording, evaluating, and assessing causality of AEs and SAEs and the procedures for completing and transmitting SAE reports are provided in Appendix 3.



090177e1a02cb071\Approved\Approved On: 14-Mar-2024 15:31 (GMT)

Care will be taken not to introduce bias when detecting AEs and/or SAEs. Open-ended and nonleading verbal questioning of the participant is the preferred method to inquire about AE occurrences.

### 8.3.3. Follow-up of AEs and SAEs

After the initial AE/SAE report, the investigator is required to proactively follow each participant at subsequent visits/contacts. For each event, the investigator must pursue and obtain adequate information until resolution, stabilization, the event is otherwise explained, or the participant is lost to follow-up (as defined in Section 7.3).

In general, follow-up information will include a description of the event in sufficient detail to allow for a complete medical assessment of the case and independent determination of possible causality. Any information relevant to the event, such as concomitant medications and illnesses, must be provided. In the case of a participant death, a summary of available autopsy findings must be submitted as soon as possible to Pfizer Safety.

Further information on follow-up procedures is given in Appendix 3.

### 8.3.4. Regulatory Reporting Requirements for SAEs

Prompt notification by the investigator to the sponsor of an SAE is essential so that legal obligations and ethical responsibilities towards the safety of participants and the safety of a study intervention under clinical investigation are met.

The sponsor has a legal responsibility to notify both the local regulatory authority and other regulatory agencies about the safety of a study intervention under clinical investigation. The sponsor will comply with country-specific regulatory requirements relating to safety reporting to the regulatory authority, IRBs/ethics committees, and investigators.

Investigator safety reports must be prepared for SUSARs according to local regulatory requirements and sponsor policy and forwarded to investigators as necessary.

An investigator who receives SUSARs or other specific safety information (eg, summary or listing of SAEs) from the sponsor will review and then file it along with the SRSD(s) for the study and will notify the IRB/ethics committee, if appropriate according to local requirements.

### 8.3.5. Exposure During Pregnancy or Breastfeeding, and Occupational Exposure

Exposure to the study intervention under study during pregnancy or breastfeeding and occupational exposure are reportable to Pfizer Safety within 24 hours of investigator awareness.

#### 8.3.5.1. Exposure During Pregnancy

An EDP occurs if:

090177e1a02cb071\Approved\Approved On: 14-Mar-2024 15:31 (GMT)

- A female participant is found to be pregnant while receiving or after discontinuing study intervention.
- A male participant who is receiving or has discontinued study intervention exposes a female partner prior to or around the time of conception.
- A female is found to be pregnant while being exposed or having been exposed to study intervention due to environmental exposure. Below are examples of environmental exposure during pregnancy:
  - A female family member or healthcare provider reports that she is pregnant after having been exposed to the study intervention by inhalation or skin contact.
  - A male family member or healthcare provider who has been exposed to the study intervention by inhalation or skin contact then exposes his female partner prior to or around the time of conception.

The investigator must report EDP to Pfizer Safety within 24 hours of the investigator's awareness, irrespective of whether an SAE has occurred. The initial information submitted should include the anticipated date of delivery (see below for information related to termination of pregnancy).

- If EDP occurs in a participant or a participant's partner, the investigator must report this information to Pfizer Safety on the CT SAE Report Form and an EDP Supplemental Form, regardless of whether an SAE has occurred. Details of the pregnancy will be collected after the start of study intervention and until 6 months after the last dose.
- If EDP occurs in the setting of environmental exposure, the investigator must report information to Pfizer Safety using the CT SAE Report Form and EDP Supplemental Form. Since the exposure information does not pertain to the participant enrolled in the study, the information is not recorded on a CRF; however, a copy of the completed CT SAE Report Form is maintained in the investigator site file.

Follow-up is conducted to obtain general information on the pregnancy and its outcome for all EDP reports with an unknown outcome. The investigator will follow the pregnancy until completion (or until pregnancy termination) and notify Pfizer Safety of the outcome as a follow-up to the initial EDP Supplemental Form. In the case of a live birth, the structural integrity of the neonate can be assessed at the time of birth. In the event of a termination, the reason(s) for termination should be specified and, if clinically possible, the structural integrity of the terminated fetus should be assessed by gross visual inspection (unless preprocedure test findings are conclusive for a congenital anomaly and the findings are reported).

Abnormal pregnancy outcomes are considered SAEs. If the outcome of the pregnancy meets the criteria for an SAE (ie, ectopic pregnancy, spontaneous abortion, intrauterine fetal



090177e1a02cb071\Approved\Approved On: 14-Mar-2024 15:31 (GMT)

demise, neonatal death, or congenital anomaly in a live-born baby, a terminated fetus, an intrauterine fetal demise, or a neonatal death), the investigator should follow the procedures for reporting SAEs. Additional information about pregnancy outcomes that are reported to Pfizer Safety as SAEs follows:

- Spontaneous abortion including miscarriage and missed abortion;
- Neonatal deaths that occur within 1 month of birth should be reported, without regard to causality, as SAEs. In addition, infant deaths after 1 month should be reported as SAEs when the investigator assesses the infant death as related or possibly related to exposure to the study intervention.

Additional information regarding the EDP may be requested by the sponsor. Further follow-up of birth outcomes will be handled on a case-by-case basis (eg, follow-up on preterm infants to identify developmental delays). In the case of paternal exposure, the investigator will provide the participant with the Pregnant Partner Release of Information Form to deliver to his partner. The investigator must document in the source documents that the participant was given the Pregnant Partner Release of Information Form to provide to his partner.

#### 8.3.5.2. Exposure During Breastfeeding

An exposure during breastfeeding occurs if:

- A female participant is found to be breastfeeding while receiving or after discontinuing study intervention.
- A female is found to be breastfeeding while being exposed or having been exposed to study intervention (ie, environmental exposure). An example of environmental exposure during breastfeeding is a female family member or healthcare provider who reports that she is breastfeeding after having been exposed to the study intervention by inhalation or skin contact.

The investigator must report exposure during breastfeeding to Pfizer Safety within 24 hours of the investigator’s awareness, irrespective of whether an SAE has occurred. The information must be reported using the CT SAE Report Form. When exposure during breastfeeding occurs in the setting of environmental exposure, the exposure information does not pertain to the participant enrolled in the study, so the information is not recorded on a CRF. However, a copy of the completed CT SAE Report Form is maintained in the investigator site file.

An exposure during breastfeeding report is not created when a Pfizer drug specifically approved for use in breastfeeding women (eg, vitamins) is administered in accord with authorized use. However, if the infant experiences an SAE associated with such a drug, the SAE is reported together with the exposure during breastfeeding.



090177e1a02cb071\Approved\Approved On: 14-Mar-2024 15:31 (GMT)

#### 8.3.5.3. Occupational Exposure

An occupational exposure occurs when a person receives unplanned direct contact with the study intervention, which may or may not lead to the occurrence of an AE. Such persons may include healthcare providers, family members, and other roles that are involved in the trial participant's care.

The investigator must report occupational exposure to Pfizer Safety within 24 hours of the investigator's awareness, regardless of whether there is an associated SAE. The information must be reported using the CT SAE Report Form. Since the information does not pertain to a participant enrolled in the study, the information is not recorded on a CRF; however, a copy of the completed CT SAE Report Form is maintained in the investigator site file.

### 8.3.6. Cardiovascular and Death Events

Not applicable.

### 8.3.7. Disease-Related Events and/or Disease-Related Outcomes Not Qualifying as AEs or SAEs

The following DREs are common in participants with advanced CRC and can be serious/life threatening:

- Progression of Disease.

  Progression of malignancy, if documented by use of appropriate method (for example, as per RECIST criteria for solid tumors) will be designated as progression of disease in the eCRF. If the malignancy has a fatal outcome during the study or within the active collection period, then the event leading to death must be recorded as an AE on the CRF, and as an SAE with CTCAE Grade 5.
- Elective hospitalization for treatment of disease under study.

Because these events are typically associated with the disease under study, they will not be reported according to the standard process for expedited reporting of SAEs even though the event may meet the definition of an SAE. These events will be recorded on the corresponding CRF page in the participant's CRF within the appropriate time frame. These DREs will be monitored by a safety review team on a routine basis.

NOTE: However, if either of the following conditions applies, then the event must be recorded and reported as an SAE (instead of a DRE):

- The event is, in the investigator's opinion, of greater intensity, frequency, or duration than expected for the individual participant.

OR

- The investigator considers that there is a reasonable possibility that the event was related to study intervention.



090177e1a02cb071\Approved\Approved On: 14-Mar-2024 15:31 (GMT)

090177e1a02cb071\Approved\Approved On: 14-Mar-2024 15:31 (GMT)

#### 8.3.8. Adverse Events of Special Interest

Not applicable.

##### 8.3.8.1. Lack of Efficacy

Lack of efficacy is reportable to Pfizer Safety only if associated with an SAE.

#### 8.3.9. Medical Device Deficiencies

Not applicable.

#### 8.3.10. Medication Errors

Medication errors may result from the administration or consumption of the study intervention by the wrong participant, or at the wrong time, or at the wrong dosage strength.

Exposures to the study intervention under study may occur in clinical trial settings, such as medication errors.

| Safety Event | Recorded on the CRF | Reported on the CT SAE Report Form to Pfizer Safety Within 24 Hours of Awareness |
|---|---|---|
| Medication errors | All (regardless of whether associated with an AE) | Only if associated with an SAE |

Medication errors include:

- Medication errors involving participant exposure to the study intervention;
- Potential medication errors or uses outside of what is foreseen in the protocol that do or do not involve the study participant.

Such medication errors occurring to a study participant are to be captured on the medication error page of the CRF, which is a specific version of the AE page.

In the event of a medication dosing error, the sponsor should be notified within 24 hours.

Whether or not the medication error is accompanied by an AE, as determined by the investigator, the medication error is recorded on the medication error page of the CRF and, if applicable, any associated AE(s), serious and nonserious, are recorded on the AE page of the CRF.

Medication errors should be reported to Pfizer Safety within 24 hours on a CT SAE Report Form **only when associated with an SAE.**

## 8.4. Treatment of Overdose

For this study, any dose of study intervention greater than the protocol-specified dose and schedule (see Section 6.1.2) will be considered an overdose.

The Sponsor does not recommend specific treatment for an overdose.

In the event of an overdose, the investigator should:

1. Contact the medical monitor within 24 hours.

2. Closely monitor the participant for any AEs/SAEs and laboratory abnormalities until study intervention can no longer be detected systemically (at least 100 days for an overdose of bevacizumab; at least 28 days for all other study interventions).

3. Document the quantity of the excess dose as well as the duration of the overdose in the CRF.

4. Overdose is reportable to Safety **only when associated with an SAE.**

Decisions regarding dose interruptions or modifications will be made by the investigator in consultation with the medical monitor based on the clinical evaluation of the participant.

## 8.5. Pharmacokinetics

Blood samples of up to approximately ~7 mL will be collected from all participants in the SLI for measurement of plasma concentrations of encorafenib and LHY746 and irinotecan and SN-38 or oxaliplatin (total platinum and platinum in plasma ultrafiltrate) as specified in Table 27 and Table 28. Pre-dose blood samples (~2 mL) prior to encorafenib administration will be collected on Day 1 of Cycle 1 through 6 from all participants in Phase 3 Arm A and Arm B, and Cohort 3 Arm D for plasma concentrations of encorafenib and LHY746 (Table 29). On days of PK blood sample collection, participants will take the morning dose of encorafenib at the investigational site under the supervision of the Investigator or designee. PK sampling must occur on the day of dosing in the clinic (ie, no 3-day window). Samples must not be drawn from the same arm in which IV study intervention is being administered. Instructions for the collection and handling of PK samples will be provided in the laboratory manual or by the sponsor. The actual date and time (24-hour clock time) of each sample will be recorded.

090177e1a02cb071\Approved\Approved On: 14-Mar-2024 15:31 (GMT)

### Table 27. PK Sampling Schedule for Cohort 1 (EC + FOLFIRI) in SLI

| Visit | Sampling Time | Irinotecan PK Sample (3 mL Blood) | Encorafenib PK Sample (2 mL Blood) |
|---|---|---|---|
| Cycle 1 Day 1 | 0 h (predose[b]) | X | |
| | 0.75 h | X | |
| | 1.5 h | X[c] | |
| | 2.5 h | X | |
| | 3.5 h | X | |
| | 5.5 h | X | |
| | 7.5 h | X | |
| Cycle 1 Day 3 | 0 h (predose[d]) | X | |
| Cycle 1 Day 15[a] | 0 h (predose[d]) | X | X |
| | 0.75 h | X | X |
| | 1.5 h | X[c] | X |
| | 2.5 h | X | X |
| | 3.5 h | X | X |
| | 5.5 h | X | X |
| | 7.5 h | X | X |
| Cycle 1 Day 17[a] | 0 h (predose[d]) | X | X |
| Day 1 of Cycle 2 – 6[a] | 0 h (Predose[d]) | X | X |

a Encorafenib will be administered within 5 minutes before the start of irinotecan infusion

b Predose is within 30 minutes prior to irinotecan infusion

c Immediately before irinotecan infusion ends

d Predose is within 30 minutes prior to encorafenib

Note: the time points are related to the start time of administration of respective drugs. Collection of samples up to and including 8 hours after dose administration should be obtained within ~ 10% of the nominal time relative to dosing (eg, within 6 minutes of a 1-hour sample)

090177e1a02cb071\Approved\Approved On: 14-Mar-2024 15:31 (GMT)

090177e1a02cb071\Approved\Approved On: 14-Mar-2024 15:31 (GMT)

### Table 28. PK Sampling Schedule for Cohort 2 (EC + mFOLFOX6) in SLI

| Visit | Sampling Time | Oxaliplatin PK Sample (5 mL Blood) | Encorafenib PK Sample (2 mL Blood) |
|---|---|---|---|
| Cycle 1 Day 1[a] | 0 h (predose[b]) | X | X |
| | 1 h | X | X |
| | 2 h | X[c] | X |
| | 3 h | X | X |
| | 4 h | X | X |
| | 6 h | X | X |
| | 8 h | X | X |
| Cycle 1 Day 3 | 0 h (Predose[b]) | X | X |
| Cycle 1 Day 15[a] | 0 h (predose[b]) | X | X |
| | 1 h | X | X |
| | 2 h | X[c] | X |
| | 3 h | X | X |
| | 4 h | X | X |
| | 6 h | X | X |
| | 8 h | X | X |
| Cycle 1 Day 17[a] | 0 h (predose[b]) | X | X |
| Day 1 of Cycle 2 – 6[a] | 0 h (predose[b]) | X | X |

a Encorafenib will be administered within 5 minutes before the start of oxaliplatin infusion

b Predose is within 30 minutes prior to encorafenib dosing

c Immediately before oxaliplatin infusion ends

Note: the time points are related to the start time of administration of respective drugs. Collection of samples up to and including 8 hours after dose administration should be obtained within ~ 10% of the nominal time relative to dosing (eg, within 6 minutes of a 1 hour sample)

### Table 29. PK Sampling Schedule for Phase 3 Arm A and Arm B and Cohort 3 Arm D

| Visit | Sampling Time | Encorafenib PK Sample (2 mL Blood) |
|---|---|---|
| Day 1 of Cycle 1 – 6 | 0 h (predose) | X |

Note: With the exception of Cycle 1 Day 1, which may be performed within 72 hours prior to initial treatment, predose is within 30 minutes prior to encorafenib dosing

The actual times may vary but the number of samples will remain the same. All efforts should be made to obtain the samples at the exact nominal time relative to dosing. Collection of samples up to and including 8 hours after dose administration that are obtained within 10% of the nominal time relative to dosing (eg, within 6 minutes of a 60-minute sample) will

not be captured as a protocol deviation, as long as the exact time of the collection is noted on the source document and the eCRF.

Samples collected for analyses of study intervention plasma concentration may also be used to evaluate safety or efficacy aspects related to concerns arising during or after the study, for metabolite identification and/or evaluation of the bioanalytical method, or for other internal exploratory purposes.

Genetic analyses will not be performed on these plasma samples.

Samples collected for measurement of plasma concentrations of encorafenib and a metabolite LHY746, irinotecan and a metabolite SN-38, or oxaliplatin will be analyzed using validated analytical methods in compliance with applicable SOPs. Exploratory metabolite identification or quantitation may be performed using either validated or exploratory methods.

The PK samples must be processed and shipped as indicated in the instructions provided to the investigator site to maintain sample integrity. Any deviations from the PK sample handling procedure (eg, sample collection and processing steps, interim storage or shipping conditions), including any actions taken, must be documented and reported to the sponsor. On a case-by-case basis, the sponsor may make a determination as to whether sample integrity has been compromised.

Any changes in the timing or addition of time points for any planned study assessments must be documented and approved by the relevant study team member and then archived in the sponsor and site study files, but will not constitute a protocol amendment. The IRB/ethics committee will be informed of any safety issues that require alteration of the safety monitoring scheme or amendment of the ICD.

### 8.5.1. Pharmacokinetics for Participants in Mainland China

Up to the first 16 participants ≥18 years old randomized to Arm A in mainland China (to obtain approximately 12 PK evaluable participants) will undergo a more intensive PK sampling (Table 30), including serial whole blood samples (2 mL) collected on Day 1 and Day 15 of Cycle 1 at pre-dose and at 0.5, 1, 2, 3, 4, 5, 6, 8 and 24 hours post-dose to assess plasma concentration of encorafenib and its metabolite LHY746. If a dose interruption occurs prior to Cycle 1 Day 15, the intensive PK sampling on Cycle 1 Day 15 is suggested to be changed to Day 1 or Day 15 of Cycle 2. Participants may be skipped if they are determined by the investigator to be ineligible for intensive PK sampling. Pre-dose blood samples prior to encorafenib administration will be collected on Day 1 of Cycle 2 through 6. Intensive PK sampling may be taken in Arm B participants in mainland China if 16 participants are not fully enrolled in Arm A.

For the other participants randomized to Arm A and Arm B or randomized in Cohort 3 Arm D who will not undergo intensive PK sampling in mainland China, pre-dose blood samples

090177e1a02cb071\Approved\Approved On: 14-Mar-2024 15:31 (GMT)

(~2 mL) prior to encorafenib administration will be collected on Day 1 of Cycle 1 through 6 for plasma concentrations of encorafenib and LHY746 (Table 31).

**Table 30. PK Sampling Schedule for the First 16 Participants Enrolled in Phase 3 Arm A in Mainland China**

| *Note:* **PK Sampling may be taken in Arm B if 16 participants are not fully enrolled in Arm A.** | | |
|---|---|---|
| **Visit** | **Sampling Time** | **Encorafenib PK Sample (2 mL Blood)** |
| Cycle 1 Day 1[a] | 0 h (predose[b]) | X |
| | 0.5 h | X |
| | 1 h | X |
| | 2 h | X |
| | 3 h | X |
| | 4 h | X |
| | 5 h | X |
| | 6 h | X |
| | 8 h | X |
| Cycle 1 Day 2 | 24 h (predose[b]) | X |
| Cycle 1 Day 15[a] | 0 h (predose[b]) | X |
| | 0.5 h | X |
| | 1 h | X |
| | 2 h | X |
| | 3 h | X |
| | 4 h | X |
| | 5 h | X |
| | 6 h | X |
| | 8 h | X |
| Cycle 1 Day 16 | 24 h (predose[b]) | X |
| Day 1 of Cycle 2 – 6[a] | 0 h (predose[b]) | X |

a Encorafenib will be administered within 5 minutes before the start of oxaliplatin infusion
b Predose is within 30 minutes prior to encorafenib dosing
Note: the time points are related to the start time of administration of respective drugs. Collection of samples up to and including 8 hours after dose administration should be obtained within ~ 10% of the nominal time relative to dosing (eg, within 6 minutes of a 1-hour sample)

090177e1a02cb071\Approved\Approved On: 14-Mar-2024 15:31 (GMT)

**Table 31. PK Sampling Schedule for Participants Enrolled in Phase 3 Arm A and Arm B, and Cohort 3 Arm D (Who Will Not Undergo Intensive PK Sampling) in Mainland China**

| Visit | Sampling Time | Encorafenib PK Sample (2 mL Blood) |
|---|---|---|
| Day 1 of Cycle 1 – 6 | 0 h (predose) | X |

Note: With the exception of Cycle 1 Day 1, which may be performed within 72 hours prior to initial treatment, predose is within 30 minutes prior to encorafenib dosing.

## 8.6. Pharmacodynamics

Biomarker samples may be used for exploratory PD analyses (see Section 8.8).

Pre-dose blood samples will be collected to enable measurements which may include but are not limited to: cfDNA, proteomic profiles, or epigenetics at the timepoints specified in the SoA.

As part of understanding the PD of the investigational product, samples may be used for evaluation of the bioanalytical method, as well as for other internal exploratory purposes. These data may not be included in the CSR.

Some samples may be analyzed using a validated analytical method in compliance with applicable SOPs while others may be analyzed using a non-characterized assay (non-validated).

The pharmacodynamic samples must be processed and shipped as indicated in the instructions provided to the investigator site to maintain sample integrity. Any deviations from the pharmacodynamic sample handling procedure (eg, sample collection and processing steps, interim storage or shipping conditions), including any actions taken, must be documented and reported to the sponsor. On a case-by-case basis, the sponsor may make a determination as to whether sample integrity has been compromised.

## 8.7. Genetics

### 8.7.1. Specified Genetics

Molecular profiling will be performed as described in Section 8.8. There is no intent to perform specific free-standing germline genomic analyses, but will be used as controls to assist in identifying and profiling somatic tumor mutations (see Section 8.8.2 for further details). No additional specified genetics will be evaluated in this study.

### 8.7.2. *BRAF* Testing

Participants will be eligible for the study based on identification of a *BRAF* V600E mutation in the tumor as determined by the central laboratory as part of Molecular Prescreening for the trial or by a local assay result from either blood or tumor tissue biopsy obtained any time prior to Screening (see Figure 1). Only PCR and NGS-based local assays results will be acceptable. If the participant is enrolled based on local assay results (using either tumor



090177e1a02cb071\Approved\Approved On: 14-Mar-2024 15:31 (GMT)

tissue or blood), the *BRAF* mutation status must be confirmed by the central laboratory (using tumor tissue only) no later than 30 days from first dose of study treatment. Either archival tumor tissue or newly collected fixed biopsy sample is required for the assessment of *BRAF* V600E mutation by the Sponsor designated central laboratory(ies). This tissue specimen should be obtained from a biopsy or surgery that was performed within 2 years prior to study enrollment. The *therascreen*® BRAF V600E RGQ PCR Kit is partially CE-marked for analytical performance according to 98/79/EC Annex VIII and is an approved medical device by the US FDA.

In cases where there is discordance between the local assay and central laboratory results, or if the central laboratory is not able to confirm presence of a *BRAF* V600E mutation due to inadequate or poor sample condition within 30 days of initiating study therapy, participants may only continue treatment if there is no clinical indication of deterioration or disease progression and the investigator determines that the participant is deriving benefit. In such instances, participants must be informed that the *BRAF* mutation status is unconfirmed and must acknowledge on the ICD that includes this information and describes alternative treatment options.

Central laboratory *BRAF* mutation tests with a definitive result (positive or negative) cannot be repeated to resolve a discordant result. Participants whose sample is determined to be inadequate or who have an indeterminate result on central testing may have samples resubmitted for testing. Lack of *BRAF* V600E confirmation by the central laboratory may be due to discordance between the local assay and central laboratory results (potential false positive local assay results), or due to inadequate or poor sample condition for central testing (indeterminate results). If at any time in the study there is lack of *BRAF* V600E confirmation in a total of 6% of the total planned enrollment of the Phase 3 portion or Cohort 3 of the trial or discordance between the local assay and the central laboratory in 3% of the total planned enrollment of Phase 3 or Cohort 3 , all subsequent participants of Phase 3 or Cohort 3 will be required to have *BRAF* V600E determined by the central laboratory for enrollment (ie, local *BRAF* testing will no longer be accepted for trial eligibility).

As the central laboratory's diagnostic test may be more sensitive and specific than local testing, tumor samples previously determined to be *BRAF*-wt by local assessment may be submitted to the central laboratory. In particular, tumors with clinicopathological features of *BRAF* mutations such as right colon tumors; poorly differentiated, mucinous, or signet-ring carcinomas; or tumors metastasized to the peritoneum,[85] should be considered for testing by central laboratory regardless of the results of prior local *BRAF* mutation testing.

090177e1a02cb071\Approved\Approved On: 14-Mar-2024 15:31 (GMT)

**Figure 1. *BRAF* Testing**

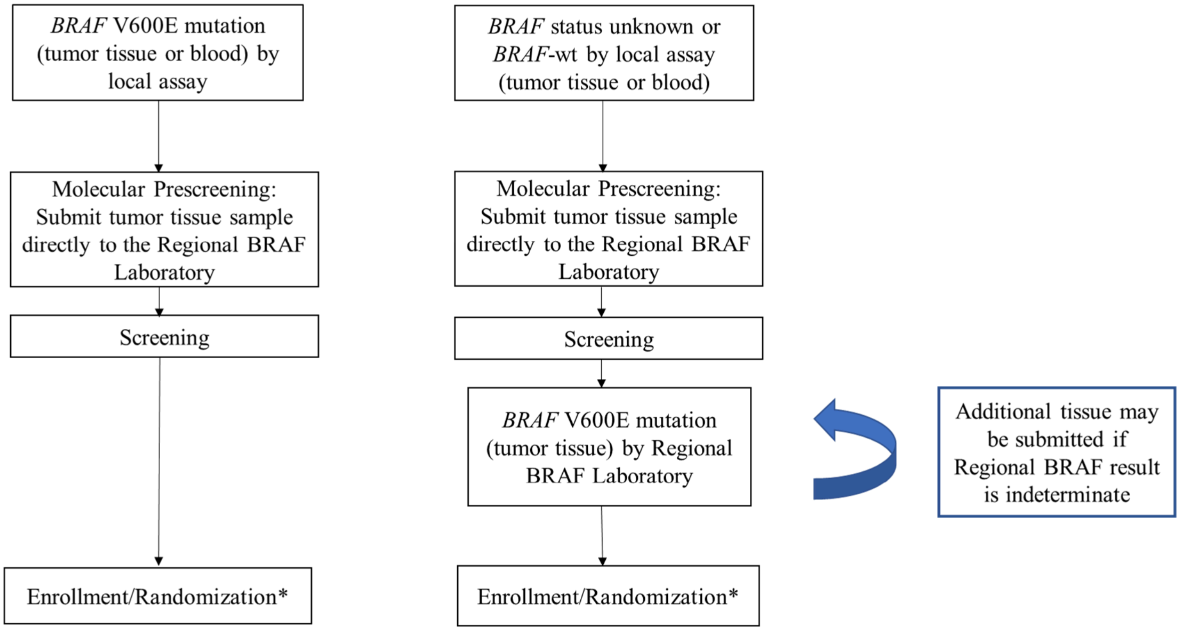


*Confirmation of *BRAF* V600E by Regional BRAF Laboratory required within 30 days of starting study intervention

### 8.7.3. Molecular Prescreening

Molecular Prescreening can be performed at any time prior to Screening. An informed consent/assent must be signed prior to any Molecular Prescreening procedure for confirmation of *BRAF* V600E status. A FFPE tumor tissue block or minimum of 15 slides (a minimum of 10 slides for mainland China) from the most recent tumor resection or biopsy must be provided for all participants enrolled in the study and submitted to the designated central laboratory(ies). This tissue specimen should be obtained from a biopsy or surgery that was performed within 2 years prior to study enrollment. If an archival tumor sample is not available, a fresh tumor biopsy must be obtained. In participants with documented *BRAF* V600E status determined by local assay prior to the study, when possible, the same tissue source should be submitted to the central laboratory in order to minimize the potential for discordance.

Slide sections must be freshly cut (ie, cut no more than 7 weeks prior to shipment to the central laboratory), 4-5 µm thick and mounted on positively-charged microscope slides. A minimum of 15 slides should be provided for *BRAF* mutation test and tumor tissue assessment (Section 8.8.2). Participants with fewer than 15 slides of analyzable tissue may be considered eligible if the Sponsor determines that the slides are sufficient for central testing of *BRAF* V600E mutation status and tumor tissue assessment. Tumor tissue from cytologic sampling (eg, fine needle aspiration, including FFPE cell pellet material) is not adequate and should not be submitted.



090177e1a02cb071\Approved\Approved On: 14-Mar-2024 15:31 (GMT)

Information regarding tissue specimen requirements, sample handling and shipment will be provided in the Laboratory Manual.

### 8.7.4. Screening

Participants with documented *BRAF* V600E status determined by the central laboratory during Molecular Prescreening or by local assay prior to this study must sign an ICD before additional screening procedures to determine eligibility for participation in the study are performed. A copy of the ICD will be given to the participant or their legal representative. The date that informed consent/assent was obtained must be documented in source documents. Participants will be registered for the study using the IRT after the informed consent/assent is obtained. Refer and comply with detailed guidelines in the IRT manual.

Participant eligibility will be verified against the inclusion and exclusion criteria once all Screening procedures are completed. The eligibility check will be embedded in the IRT system.

### 8.7.5. Companion Diagnostic for Eligibility

Participants are required to have their disease assessed for a *BRAF* V600E mutation based on testing de novo or archival tumor tissue using the QIAGEN *therascreen*® BRAF V600E RGQ PCR Kit (FDA approved [USA]; partially CE marked for use in Europe) performed by a study-sponsored CAP/CLIA-certified central clinical laboratory. Testing may be performed during Molecular Prescreening or Screening.

Details on processes for collection and shipment of these samples can be found in the laboratory manual.

Although it is anticipated that most samples will be consumed by planned analyses during the course of the study, any unused samples may be stored at a facility selected by the sponsor for a maximum of 10 years (or according to local regulations) following the last participant's last visit for the study unless prohibited by local regulations or ethics committee decision.

### 8.7.6. Companion Diagnostic Assay Development

An in vitro diagnostic assay may be developed in accordance with FDA's Quality System Regulations and Design Control requirements. Any tumor samples remaining after testing for *BRAF* V600E and/or a blood sample collected during Screening ("Blood sample for BRAF CDx" – 20 mL of blood optimized for plasma preparation) may be used for companion diagnostic development and Health Authority registration, and to assess the concordance between the results for *BRAF* V600E status obtained using the central laboratory assay to determine eligibility for the study and the companion diagnostic assay which will be submitted for premarket approval.

Details on processes for collection and shipment of these samples can be found in the laboratory manual.



090177e1a02cb071\Approved\Approved On: 14-Mar-2024 15:31 (GMT)

Although it is anticipated that most samples will be consumed by planned analyses during the course of the study, any unused samples may be stored at a facility selected by the sponsor for a maximum of 10 years (or according to local regulations) following the last participant's last visit for the study unless prohibited by local regulations or ethics committee decision.

***Note***: For participants in mainland China, sample collection and testing for *BRAF* V600E will not be performed until approval by HGRAC.

### 8.7.7. Banked Biospecimens for Genetics

A 4 mL blood sample optimized for DNA isolation [Prep D1] will be collected at the time specified in the SoA as local regulations and IRBs/ethics committees allow.

Banked Biospecimens may be used for research related to the study intervention(s) and cancer. Genes and other analytes (eg, proteins, RNA, nondrug metabolites) may be studied using the banked samples.

See Appendix 5 for information regarding genetic research. Details on processes for collection and shipment of these samples can be found in the lab manual.

***Note***: Banked biospecimens are not applicable for participants in mainland China.

## 8.8. Biomarkers

Unless prohibited by local regulations or ethics committee decision, the following samples for biomarker research are required and will be collected from all participants in this study within the SLI and Phase 3 portions as specified in the SoA:

- Approximately 2 mL blood sample at designated time points to measure levels of CRP and CEA.
- Pre-treatment tumor tissue samples to enable retrospective genomic profiling (eg *RAS* mutations, MSI status) and expression analyses (eg gene, protein).
- Approximately 20 mL blood samples (or 10 mL for mainland China sites) at designated time points to be processed to plasma for assessment of cfDNA.
- Approximately 10 mL blood samples (or 1 mL for mainland China sites) at designated time points to be processed to serum for proteomic profiling.
- Approximately 4 mL whole blood samples at baseline for filtering of somatic mutation data.
- ***Optional:*** Tumor tissue samples at progression/EOT for assessment of tumoral changes which may occur over the course of therapy, including potential acquired resistance mechanisms.

***Note***: For participants in mainland China, sample collection and biomarker testing will not be performed until approval by HGRAC.



090177e1a02cb071\Approved\Approved On: 14-Mar-2024 15:31 (GMT)

### 8.8.1. CRP and CEA assessments

Blood samples (2 mL) will be collected to measure levels of CRP and CEA using routine clinical laboratory testing. These analyses will be used to explore relationships between levels of CRP and CEA and clinical outcome.

### 8.8.2. Tumor Tissue Assessments

Tumor biospecimens representing FFPE tissue samples from tumor resection or biopsy will be used to analyze candidate DNA, RNA, or protein markers, or relevant signature of markers for their ability to identify those participants who are most likely to benefit from treatment with the study intervention.

Tumor tissue requirements are included in Section 8.7.2 and further details are provided in the Laboratory Manual for mandatory pre-screening/screening tumor samples. Optional on-treatment de novo tumor biopsies obtained during surgical resection or upon disease progression/EOT are requested except in instances where the procedure poses unacceptable risks per Investigator documentation. The EOT tumor biopsy should be performed before initiation of subsequent anti-cancer therapy and preferably no later than 7 days after the EOT visit. These tumor samples may be used to investigate acquired mechanisms of resistance and changes in the tumor and TME (eg, PD-L1 expression, presence of regulatory T-cells, myeloid-derived suppressors). Additional information on tissue collection procedures can be found in the Laboratory/Study Manual.

Targeted and/or whole exome/genome sequencing and/or transcriptome and/or epigenetic analyses will be performed using the remainder of the de novo or archival tumor tissue samples submitted at prescreening or screening for eligibility assessment to evaluate the genomic landscape (eg, *RAS* mutations, MSI status, etc) within the participant population. Additionally, these samples and tumor tissue collected at progression/EOT (when available) will be used to gain insight into the mechanisms that confirm sensitivity/resistance to study treatment. The results of these analyses may be combined with the results from targeted genomic analyses performed for enrollment eligibility assessment to further explore correlations of molecular profiling with response to study treatment. In addition, these samples may be used to help support development of a potential tumor tissue diagnostic test for encorafenib.

Unless prohibited by local regulations or ethics committee decision, whole blood samples (4 mL) will be collected from all participants on Day 1 before the first dose of study treatment (Cycle 1 Day 1) for exploratory targeted and/or whole exome/genome sequencing. These samples will be used to characterize the germline status of genetic variants (eg, DDR genes, MMR genes, etc). They will not otherwise be used to generate free-standing germline sequencing results, but rather as controls to assist in identifying and profiling somatic tumor DNA mutations. These samples are required for all enrolled participants where tumor tissue for genomic screening was collected from same participant.

090177e1a02cb071\Approved\Approved On: 14-Mar-2024 15:31 (GMT)

### 8.8.3. Circulating Free DNA (cfDNA)

Blood samples (20 mL or 10 mL for mainland China sites) for cfDNA analyses will be collected from all participants at timepoints specified in the SoA and may also include remnant sample from "Blood sample for *BRAF* CDx". cfDNA extracted from these samples will be used to assess epigenetic (eg, methylation) and genetic changes (eg, *BRAF*, *KRAS*, *NRAS*, etc) in ctDNA to (a) estimate the relationship between tumor burden (as assessed by VAF and/or methylation) and response to study treatment, (b) explore correlations with tumor molecular profiling, and (c) understand potential mechanisms of acquired resistance to study treatment. A targeted sequencing panel may be used for these analyses, which may include DDR genes and MSI status. Exploratory whole exome/genome sequencing and/or epigenetic analyses may be performed. In addition, these samples may be used to help support development of a potential blood-based test for encorafenib.

Although it is anticipated that most samples will be consumed by planned analyses during the course of the study, any unused samples may be stored at a facility selected by the sponsor for a maximum of 10 years (or according to local regulations) following the last participant's last visit for the study.

### 8.8.4. Specified Gene Expression (RNA) Research

An FFPE tissue sample for RNA isolation will be collected as noted in Section 8.8.2.  The samples will be analyzed by targeted and/or transcriptomic profiling to evaluate gene expression levels and may be used to identify signatures associated with response, resistance and adverse events in response to treatment.

Details on processes for collection and shipment of these samples can be found in the laboratory manual.

Although it is anticipated that most samples will be consumed by planned analyses during the course of the study, any unused samples may be stored at a facility selected by the sponsor for a maximum of 10 years (or according to local regulations) following the last participant's last visit for the study.

### 8.8.5. Specified Protein Research

Approximately 10 mL blood sample or 1 mL for mainland China sites (processed for approximately 5 mL of serum or 0.5 mL for mainland China sites) for protein analyses will be collected from all participants at the timepoints specified in the SoA.  The sample(s) may be analyzed for proteomic signatures associated with response or resistance to therapy in pretreatment and on-treatment samples. This sample may also be used for additional research based on emerging knowledge of CRC biology.

Details on processes for collection and shipment of these sample(s) can be found in the laboratory manual.

Although it is anticipated that most samples will be consumed by planned analyses during the course of the study, any unused samples may be stored at a facility selected by the sponsor



090177e1a02cb071\Approved\Approved On: 14-Mar-2024 15:31 (GMT)

for a maximum of 10 years (or according to local regulations) following the last participant's last visit for the study.

#### 8.8.6. Specified Metabolomic Research

Specified metabolomic research is not included in this study.

### 8.9. Immunogenicity Assessments

Immunogenicity assessments are not included in this study.

### 8.10. Health Economics

Health economic parameters may be captured as part of the trial to understand overall and differential healthcare resource utilization associated with each treatment and the Control Arm, by using the data entered in the clinical database. Such parameters may include, but will not be limited to, AEs and SAE data, concomitant medications, and surgical and non-surgical procedures. All information will be kept confidential.

## 9. STATISTICAL CONSIDERATIONS

Detailed methodology for summary and statistical analyses of the data collected in this study is outlined here and further detailed in a SAP, which will be maintained by the sponsor. The SAP may modify what is outlined in the protocol where appropriate; however, any major modifications of the primary endpoint definitions or their analyses will also be reflected in a protocol amendment.

### 9.1. Estimands and Statistical Hypotheses

#### 9.1.1. Estimands

Estimands are defined below for the primary endpoint of incidence of DLTs in the SLI, for the primary and key secondary endpoints in the Phase 3 portion of the study, and for the primary and key secondary endpoints in Cohort 3.

**Safety Lead-in**

Primary Estimand (DLT):

- Population: all participants with local or central laboratory confirmed *BRAF* V600E-mCRC, as defined by the screening inclusion/exclusion criteria in Section 5.1.2 and Section 5.2.2 to reflect the targeted population of the SLI portion of the study.
- Variable: DLT rate during the DLT-evaluation period, which is the first 28 days after the first dose of study intervention in the SLI. DLTs are defined in Section 6.1.1.1.
- Intercurrent events: hypothetical strategy will be applied for the intercurrent events. The intercurrent event is treatment discontinuation for reasons other than treatment-related toxicity that leads to <75% of the planned dose of each study intervention during the DLT evaluation period. Participants without DLTs and with the intercurrent event will not be included in the DLT rate calculation.



090177e1a02cb071\Approved\Approved On: 14-Mar-2024 15:31 (GMT)

- Population-level summary measure: DLT rate defined as the number of DLT-evaluable participants with DLTs in the DLT-evaluation period divided by the number of DLT-evaluable participants.

**Phase 3, Two Primary Estimands**

PFS for Arm B vs Control Arm:

- Population: all participants who are randomized in the Phase 3 portion of the study. This analysis population will be based on FAS defined in Section 9.3.
- Variable: PFS by BICR, defined as the time from the date of randomization to the earliest documented disease progression per RECIST v1.1, or death due to any cause.
- Intercurrent events: hypothetical strategy will be applied for the intercurrent events. Details of the intercurrent events and censoring rules for the primary analysis are summarized in Table 32.
- Population-level summary measure: hazard ratio for PFS and corresponding 2-sided CI based on Cox proportional hazard model stratified by ECOG performance status at randomization and region. PFS will be compared between the two treatment arms using a 1-sided stratified log-rank test.

OR for Arm B vs Control Arm:

- Population: the first 110 participants who are randomized in the Phase 3 portion of the study into each of Arm B and Arm C (Full Analysis Set, ORR Subset defined in Section 9.3).
- Variable: Objective response defined as complete response (CR), or partial response (PR) according to RECIST v1.1 based on BICR assessment, from the date of randomization until the date of the first documentation of progression of disease (PD). Both CR and PR must be confirmed by repeat assessments performed no less than 4 weeks after the criteria for response are first met. Only tumor assessments performed on or before the start date of subsequent anticancer therapies will be considered in the assessment.
- Intercurrent events: All data collected after an intercurrent event of subsequent anticancer therapy or first PD will be excluded.
- Population-level summary measure: stratified (by the randomization strata) odds ratio in terms of OR defined as the odds of OR with Arm B divided by the odds of OR

090177e1a02cb071\Approved\Approved On: 14-Mar-2024 15:31 (GMT)

with Arm C, with its 2-sided 95% CI. ORR treatment effect will be compared using Cochran-Mantel-Haenszel test stratified by the randomization strata.

Key Secondary Estimand (OS for Arm B vs Control Arm):

- Population: all participants who are randomized in the Phase 3 portion of the study. This analysis population will be based on FAS defined in Section 9.3.
- Variable: OS, defined as the time from the date of randomization to death due to any cause.
- Intercurrent events: hypothetical strategy will be applied for the intercurrent events of lost to follow-up or withdrawal of consent/assent. Participants not known to have died are censored at the date of last contact.
- Population-level summary measure: hazard ratio for OS and corresponding 2-sided CI based on Cox proportional hazard model stratified by ECOG performance status at randomization and region. OS will be compared between the two treatment arms using a 1-sided stratified log-rank test.

**Table 32. PFS Outcome and Event Dates – Primary Analysis**

| Situation | Date of Progression/Censoring | Outcome |
|---|---|---|
| No adequate baseline assessment, including no disease at baseline | Date of randomization [a] | Censored [a] |
| PD or death ≤12 (or 16) [b] weeks after last adequate tumor assessment or ≤12 weeks after date of randomization | Date of PD or death | Event |
| PD or death >12 (or 16) [b] weeks after the last adequate tumor assessment [c] | Date of last adequate tumor assessment [c] documenting no PD prior to new anti-cancer therapy, or missed assessments | Censored |
| No PD | | |
| New anti-cancer therapy given[d] | | |

a If the participant dies ≤12 weeks after date of randomization, the death is an event with date on death date.

b Durations are equal to 2 times the length of the tumor assessment interval, which is 12 weeks for the first 18 months after randomization, and 16 weeks thereafter.

c If there are no adequate post-baseline assessments prior to the PD or death, then the time without adequate assessment should be measured from the date of randomization; if the criteria is met, the censoring will be on the date of randomization.

d New anticancer therapy includes systemic therapy, radiation or surgery.

## Cohort 3

Primary Estimand (OR):

- Population: all participants who are randomized in Cohort 3. This analysis population will be based on FAS defined in Section 9.3.



090177e1a02cb071\Approved\Approved On: 14-Mar-2024 15:31 (GMT)

- Variable: Objective response defined as CR, or PR according to RECIST v1.1 based on BICR assessment, from the date of randomization until the date of the first documentation of PD, death or start of new anticancer therapy. Both CR and PR must be confirmed by repeat assessments performed no less than 4 weeks after the criteria for response are first met. Only tumor assessments performed on or before the start date of subsequent anticancer therapies will be considered in the assessment.
- Intercurrent events: All data collected after an intercurrent event of subsequent anticancer therapy or first PD will be excluded.
- Population-level summary measure: stratified (by the randomization strata) odds ratio in terms of OR defined as the odds of OR with Arm D divided by the odds of OR with Arm E, with its 2-sided 95% CI. ORR treatment effect will be compared using Cochran-Mantel-Haenszel test stratified by the randomization strata. Participants who do not have a post-baseline tumor assessment due to early progression of disease, who receive anti-cancer therapies other than the study treatments prior to reaching a CR or PR, or who die, have PD, or stop tumor assessments for any reason prior to reaching a CR or PR will be counted as non-responders in the assessment of OR. Each participant will have an objective response status (0: no OR; 1: OR).

Key Secondary Estimand (PFS):

- Population: all participants who are randomized in the Cohort 3. This analysis population will be based on FAS defined in Section 9.3.
- Variable: PFS by BICR, defined as the time from the date of randomization to the earliest documented disease progression per RECIST v1.1, or death due to any cause.
- Intercurrent events: hypothetical strategy will be applied for the intercurrent events. Details of the intercurrent events and censoring rules are summarized in Table 32.
- Population-level summary measure: hazard ratio for PFS and corresponding 2-sided CI based on Cox proportional hazard model stratified by the randomization strata. PFS will be compared between the two treatment arms using a 1-sided stratified log-rank test.

### 9.1.2. Statistical Hypotheses

There is no statistical hypothesis for the SLI portion of the study.

### Phase 3

For Phase 3 portion of the study, the primary objective is to compare the efficacy, as measured by the primary endpoints of PFS by BICR and ORR by BICR, of Arm B versus the Control Arm. The following statistical hypotheses will be tested to address the primary objective:

$H_{01}$: $HR_{PFS\ (B\ versus\ Control)} \geq 1$ versus $H_{11}$: $HR_{PFS\ (B\ versus\ Control)} < 1$

$H_{02}$: $OR_{OR(B\ versus\ Control)} \leq 1$ versus $H_{12}$: $OR_{OR(B\ versus\ Control)} > 1$



090177e1a02cb071\Approved\Approved On: 14-Mar-2024 15:31 (GMT)

where $HR_{PFS\ (B\ versus\ Control)}$ is the hazard ratio for PFS of Arm B versus the Control Arm and $OR_{OR(B\ versus\ Control)}$ is the odds ratio for objective response of Arm B versus the Control Arm.

The key secondary objective is to compare the efficacy, as measured by the key secondary endpoint of OS, of Arm B versus Control. The following statistical hypothesis will be tested to address the key secondary objective:

$$H_{03}: HR_{OS\ (B\ versus\ Control)} \geq 1 \text{ versus } H_{13}: HR_{OS\ (B\ versus\ Control)} < 1$$

where $HR_{OS\ (B\ versus\ Control)}$ is the hazard ratio for OS of Arm B versus the Control Arm.

Overall 1-sided alpha of 0.025 for the study will be preserved using 1-sided alpha of 0.001 for the comparison of ORR by BICR of Arm B vs Control Arm, 1-sided alpha of 0.023 for the comparison of PFS by BICR of Arm B vs Control Arm. A hierarchical testing procedure will be used to control the family-wise type I error rate. In addition, 1-sided alpha of 0.001 will be reserved for the comparison of PFS by BICR of Arm A vs Arm C, even if there is no plan for formal testing. Details of this strategy are described in Section 9.4.1.1. The study will be considered positive (ie, demonstrated evidence of effectiveness) if the hypothesis test of either primary endpoint is statistically significant.

**Cohort 3**

The primary objective is to compare the efficacy, as measured by ORR by BICR, of Arm D versus Arm E. The following statistical hypothesis will be tested:

$$H_{01}: OR_{OR(D\ versus\ E)} \leq 1 \text{ versus } H_{11}: OR_{OR(D\ versus\ E)} > 1$$

where $OR_{OR(D\ versus\ E)}$ is the odds ratio for objective response of Arm D versus Arm E.

The key secondary objective is to compare the efficacy, as measured by the key secondary endpoint of PFS by BICR, of Arm D versus Arm E. The following statistical hypothesis will be tested to address the key secondary objective:

$$H_{02}: HR_{PFS\ (D\ versus\ E)} \geq 1 \text{ versus } H_{12}: HR_{PFS\ (D\ versus\ E)} < 1$$

where $HR_{PFS\ (D\ versus\ E)}$ is the hazard ratio for PFS of Arm D versus Arm E

## 9.2. Sample Size Determination

The planned sample size of the study is approximately 815 participants including approximately 60 participants in the SLI, approximately 470 participants in the Phase 3 portion of the study (Arm B and Arm C), and approximately 135 participants in Cohort 3. In addition, up to the time of Protocol Amendment 5 approval, approximately 150 participants are estimated to be randomized in Arm A of the Phase 3 portion of the study.

**Safety Lead-in**

The primary endpoint of the SLI of the study is incidence of DLTs. Up to 60 evaluable participants will be enrolled on a rolling basis in an alternating manner to receive either EC + mFOLFOX6 or EC + FOLFIRI (up to 30 participants per cohort). The safety data will be



090177e1a02cb071\Approved\Approved On: 14-Mar-2024 15:31 (GMT)

evaluated by the SC after the first 9 evaluable participants in each cohort have been followed for a minimum of 28 days with EC + mFOLFOX6 or EC + FOLFIRI. The target DLT rate in the first 28 days is <33% (ie, <3 out of 9 evaluable participants with DLTs).

Table 33 provides a comparison of the characteristics of this DLT evaluation rule and the traditional 3 + 3 rules based on 9 participants.

**Table 33. Characteristics of DLT Probability During the DLT Evaluation Period**

| True DLT Rate During DLT Evaluation Period | Probability of Dose Declared Toxic Using 3 + 3 Rules | Probability of Observed DLT Rate ≥33% in 9 Participants During DLT Evaluation Period |
|---|---|---|
| 10% | 0.094 | 0.053 |
| 20% | 0.291 | 0.262 |
| 30% | 0.506 | 0.537 |
| 40% | 0.691 | 0.768 |
| 50% | 0.828 | 0.910 |

The DLT evaluation period is the first 28 days after the first dose of study intervention in the SLI, and the DLT-evaluable is defined in Section 6.1.1.1.

If the doses are determined to be tolerable in the first 9 evaluable participants in either or both regimens based on the observed DLT rate (<33%) and evaluation of the overall toxicity profile, the SLI will be expanded for a total of up to 30 evaluable participants in each cohort. If the DLT rate is ≥33% in either cohort, that combination regimen will not be evaluated further in the SLI and will not be used in Phase 3.

Table 34 summarizes the probability of observing at least one instance of a toxicity that has a true incidence rate of 10% and 15% for the given number of participants.

**Table 34. Probability of Observing ≥1 Instance of Toxicity at 10% and 15% Incidence Rate**

| True Toxicity Incidence Rate | Number of Participants | Probability of Observing ≥1 Instance of Toxicity |
|---|---|---|
| 10% | 15 | 0.79 |
| | 20 | 0.88 |
| | 30 | 0.96 |
| 15% | 15 | 0.91 |
| | 20 | 0.96 |
| | 30 | 0.99 |

If any participant is deemed non-evaluable for DLT, additional participants may be enrolled to ensure there are a sufficient number of evaluable participants in the SLI.

**Phase 3**

Approximately 620 participants will be randomized overall, initially at a ratio of 1:1:1 to receive EC (Arm A), EC + chemotherapy (Arm B) or SOC chemotherapy (Arm C), and then



090177e1a02cb071\Approved\Approved On: 14-Mar-2024 15:31 (GMT)

1:1 to receive EC + chemotherapy (Arm B) or SOC chemotherapy ( Arm C) after the approval of Protocol Amendment 5, with a total of approximately 150 participants for Arm A and 235 participants each for Arm B and Arm C. Randomization is stratified by ECOG performance status (0 versus 1) and region (US/Canada versus Europe versus Rest of World).

Primary Endpoints

Approximately 230 PFS by BICR events will be required to have at least 85% power to detect a hazard ratio of 0.67 between Arm B and Arm C using a 1-sided stratified log-rank test at a significance level of 0.023.

The sample size of 235 participants per arm was determined based on the assumptions of a hazard ratio of 0.67 under the exponential model assumptions, and median PFS of 7 months on the Arm C. The sample size further assumes a dropout hazard rate of 0.07 within each treatment arm, non-uniform participants accrual over 26 months (for 2 arms: 6 participants per month for the first 5 months, 11 participants a month for months 6 through 7, 16 participants a month for months 8 through 11, 23 participants a month for months 12 through 16, and 24 participants per month thereafter).

The sample size of 220 participants (110 per arm) will provide 90% power for test of odds ratio of 2 proportions between the Arm B and Arm C using a 1-sided chi-square test at a significance level of 0.001 assuming an ORR by BICR of 35% and 65% for Arm C and Arm B, respectively.

Key Secondary Endpoint

For the comparison of Arm B versus Arm C OS, 297 OS events in Arm B + Arm C will achieve 85% power to detect an OS HR of 0.70 using a 1-sided stratified log-rank test at a significance level of 0.023, and a 2-look group-sequential design with a Lan-DeMets (O'Brien-Fleming) spending function to determine the efficacy boundaries. An exponential distribution for OS was assumed, that corresponds to an improvement in the median OS of 6.4 months (21.4 months in Arm B versus 15 months in Arm C). The sample size further assumes a dropout hazard rate of 0.011 within each treatment arm, interim analysis after 80% of OS events, and follow-up after the last participant is randomized of about 24 months.

**Cohort 3**

Primary Endpoint

The sample size calculation is based on the primary endpoint of ORR as determined by BICR per RECIST v1.1. The hypothesis to be tested is described in Section 9.1.2. The sample size of 135 participants (90 in Arm D and 45 in Arm E) will provide 90% power for test of odds ratio of 2 proportions between the Arm D and Arm E using a 1-sided chi-square test at a significance level of 0.025 assuming an ORR by BICR of 35% and 65% for Arm E and Arm D, respectively.

Key Secondary Endpoint



090177e1a02cb071\Approved\Approved On: 14-Mar-2024 15:31 (GMT)

For the comparison of Arm D versus Arm E PFS by BICR, 135 participants and 82 PFS events in Arm D + Arm E will achieve 80% power to detect a PFS HR of 0.519 using a 1-sided stratified log-rank test at a significance level of 0.025. An exponential distribution for PFS was assumed, that corresponds to an improvement in the median PFS of 6.5 months (13.5 months in Arm D versus 7 months in Arm E). The sample size further assumes a dropout hazard rate of 0.015 within each treatment arm, uniform participants accrual of 20 participants a month, and follow-up after the last participant is randomized of about 14 months.

## 9.3. Analysis Sets

For purposes of analysis, the following analysis sets are defined:

| Participant Analysis Set | Description |
|---|---|
| **Enrolled** | All participants who sign the Screening ICD. |
| **DLT-Evaluable Analysis Set** | All participants who receive at least 1 dose of study intervention in the SLI and either experience DLT during the DLT evaluation period or complete the DLT evaluation period without DLT. Participants without DLTs who do not receive at least 75% of the planned dose of each study intervention during the DLT evaluation period for reasons other than treatment-related toxicity (eg, protocol violation) are not evaluable for DLT. The DLT evaluation period is the first 28 days after the first dose of study intervention. |
| **Full Analysis Set (FAS)** | For participants in the SLI the FAS includes all participants who receive at least 1 dose of study intervention.<br><br>For participants in the Phase 3 portion and Cohort 3, the FAS includes all participants who are randomized in the Phase 3 portion and Cohort 3 of the study, respectively. Participants will be analyzed according to the study treatment assigned at randomization. |
| **Full Analysis Set, ORR Subset** | For the primary analysis of ORR/DOR/TTR in the Phase 3 portion of the study, "Full Analysis Set, ORR Subset" comprises the first 110 participants randomized in each Arm B and Arm C. |
| **Centrally Assessed *BRAF* V600E Positive Analysis Set** | All participants who are randomized in the Phase 3 portion of the study with a confirmed central laboratory result of *BRAF* V600E mutation. Participants will be analyzed according to the study treatment assigned at randomization. |
| **Safety Analysis Set** | All participants who receive at least 1 dose of study intervention.<br><br>For the Phase 3 portion and Cohort 3 of the study, participants will be analyzed according to the study treatment assigned at randomization unless the incorrect treatment(s) was/were received throughout the dosing period in which case participants will be analyzed according to the actual study treatment received in the first cycle. |
| **PK Analysis Set** | The PK concentration set is defined as all enrolled participants who are treated and have at least 1 analyte concentration. |



090177e1a02cb071\Approved\Approved On: 14-Mar-2024 15:31 (GMT)

| Participant Analysis Set | Description |
|---|---|
| | The PK parameter analysis set is defined as all enrolled participants treated who have sufficient information to estimate at least 1 of the PK parameters of interest and have no major protocol deviations affecting PK assessment. |
| **Biomarker Analysis Set** | All participants who are in the safety analysis set and who had at least 1 of the pharmacodynamic parameters or biomarkers evaluated at pre and/or post dose. |

## 9.4. Statistical Analyses

The SAP will be developed and finalized before any analyses are performed and will describe the analyses and procedures for accounting for missing, unused, and spurious data. This section is a summary of the planned statistical analyses of the primary and secondary endpoints.

### 9.4.1. General Considerations

All analyses will be performed by treatment arm for the SLI, Phase 3 portion, and Cohort 3 of the study separately. All efficacy analyses will be performed using the FAS, and all safety analyses will be performed using the safety analysis set.

In general, descriptive summaries will be presented for the efficacy and safety variables collected. Continuous variables will be summarized using mean, standard deviation, minimum, median, and maximum. Categorical variables will be summarized using frequency counts and percentages.

Unless otherwise specified, the calculation of proportions will be based on the sample size of the population of interest. Counts of missing observations will be included in the denominator and presented as a separate category if not otherwise specified in the SAP.

Statistical analyses will be performed using SAS® version 9.4 or higher.

#### 9.4.1.1. Statistical Testing Strategy

For the Phase 3 portion of the study a hierarchical testing procedure will be used to control the family-wise type I error rate.

Eight months after randomization of the first 110 participants each in Arm B and in Arm C, or after the completion of enrollment of the Phase 3 portion of the study, whichever occurs later, the ORR by BICR will be compared for Arm B versus Arm C, on these first 220 participants, using a 1-sided alpha of 0.001.

Once at least 230 PFS events by BICR will be observed for Arm B + Arm C and at least 12 months after the completion of enrollment of the Phase 3 portion of the study, the PFS analysis for the primary comparison of PFS by BICR for Arm B versus the Arm C will be tested using a 1-sided alpha of 0.023.



090177e1a02cb071\Approved\Approved On: 14-Mar-2024 15:31 (GMT)

At the time of ORR and PFS analysis if at least one of them will be significant an interim analysis for OS will be conducted and hierarchical procedure for control of alpha for ORR/PFS and OS will be applied.

If the results of the interim analysis of OS are not statistically significant, a final OS analysis will be performed once 297 events are observed for Arm B + Arm C. Details of the OS interim efficacy analysis are described in Section 9.5.1 and will be included in the SAP.

The overall significance level for Phase 3 portion of the study is 0.025 (1-sided) and will be used as follows:

- 1-sided alpha of 0.001 for the comparison of ORR by BICR of Arm B vs Arm C;
- 1-sided alpha of 0.023 for the comparison of PFS by BICR of Arm B vs Arm C;
- alpha for the comparison of OS of Arm B vs Arm C will depend on the outcome of ORR and PFS;
- 1-sided alpha of 0.001 for the comparison of PFS by BICR of Arm A vs Arm C, even if there is no plan for formal testing;

In Cohort 3, the analysis of ORR by BICR comparing Arm D versus Arm E will be performed at least 8 months after the randomization of the last participant using 1-sided alpha of 0.025. A hierarchical testing procedure will be used to control the family-wise type I error rate at 0.025 (1-sided) level for ORR and PFS. PFS by BICR will be tested when at least 82 events will be observed, only if the analysis for ORR is statistically significant. If ORR results are not statistically significant, PFS will be analyzed descriptively.

No formal statistical testing will be performed on the secondary and exploratory endpoints (with the exception for key endpoint described above), sensitivity analyses and subgroup analyses. These results will be reported with descriptive statistics only.

Confidence intervals will be 2-sided with a confidence level of 95%, if not otherwise specified.

#### 9.4.1.2. Pooling of Centers

In order to provide overall estimates of treatment effects, data will be pooled across trial centers. The "center" factor will not be considered in statistical models or for subgroup analyses due to the high number of participating centers in contrast to the anticipated small number of participants randomized at each center.

#### 9.4.1.3. Stratification Factors

For Phase 3 portion the stratification factors used for stratified statistical analyses are those used at randomization via the IRT: ECOG performance status (0 versus 1) and region (US/Canada versus Europe versus Rest of World).



090177e1a02cb071\Approved\Approved On: 14-Mar-2024 15:31 (GMT)

For Cohort 3 the only stratification factor used for stratified statistical analyses and at randomization via the IRT is ECOG performance status (0 versus 1).

#### 9.4.1.4. Definition of Baseline

For all endpoints assessments in the SLI, and all safety assessments in the Phase 3 portion of the study and in Cohort 3, baseline is defined as the last completed assessment prior to date of first dose in the SLI, Cohort 3, and Phase 3, respectively. If an assessment that is planned to be performed prior to the first dose of study intervention in the protocol is performed on the same day as the first dose of study intervention and the time is unknown, it will be assumed that it was performed prior to study intervention administration and will be considered as baseline assessment.

For efficacy assessments in the Phase 3 portion of the study and in Cohort 3, baseline is defined as the last assessment prior to randomization. If the assessment that is planned to be performed before randomization per the protocol is performed on the same day as the date of randomization and assessment time point is missing, it will be assumed that it was performed prior to randomization and will be considered as baseline assessment. If no tumor assessment was performed prior to randomization, any tumor assessment conducted between randomization and prior to or on the first study intervention dose date will be considered as baseline tumor assessment.

Unscheduled assessments will be used in the determination of baseline. Data reported at the End of Treatment visit are not eligible for baseline selection.

#### 9.4.1.5. Definition of On-treatment Period

The on-treatment period is defined as the time from the first dose of study treatment through minimum of (last dose of study treatment + 28 days, start day of subsequent anticancer therapy - 1 day) unless otherwise described in the SAP.

### 9.4.2. Primary Endpoints

**Safety Lead-in**

The primary endpoint in the SLI of the study is incidence of DLTs. The number and proportion of participants experiencing DLTs during the DLT-evaluation period will be summarized and listed by treatment cohort. Analyses of DLT will be performed on DLT-Evaluable Set.

**Phase 3**

The primary objective is to compare the efficacy, as measured by the primary endpoints of PFS by BICR and ORR by BICR, of Arm B versus Arm C.

PFS is defined as the time from the date of randomization to the earliest documented disease progression per RECIST v1.1, or death due to any cause. PFS will be calculated in months as follows:



090177e1a02cb071\Approved\Approved On: 14-Mar-2024 15:31 (GMT)

PFS (months) = (date of event or censoring– randomization date +1) / 30.4375

Participants without an event or with an event more than 12 weeks (for the first 18 months after randomization) or 16 weeks (after the first 18 months of randomization) after the last adequate tumor assessment will be censored on the date of the last adequate tumor assessment that documented no progression. In addition, if a new anti-cancer therapy is started prior to an event, the participant will be censored on the date of the last adequate tumor assessment that documented no progression prior to the start of the new anti-cancer therapy. Anticancer therapy includes systemic anticancer therapy, radiation, and surgery.

An adequate post-baseline assessment is defined as an assessment where a response of CR, PR, SD, non-CR/non-PD, or PD can be determined. Time points where the response is NE or no assessment was performed will not be used for determining the censoring date.

Participants with no baseline tumor assessment (including participants with an inadequate baseline assessment) or with no adequate post-baseline tumor assessments within 12 weeks after the date of randomization will be censored on the day of randomization, unless the participant dies within 12 weeks of randomization, in which case, death will be an event on date of death.

The censoring and event date options to be considered for the PFS primary analysis are presented in Table 32.

The primary analysis will be performed to compare PFS between Arm B versus Arm C using a 1-sided stratified log-rank test at the 0.023 significance level.

The treatment effect will be estimated using a Cox's proportional hazards model stratified by randomization strata to calculate the hazard ratio for PFS of Arm B versus Arm C.

The 95% CIs for the hazard ratios will be reported. PFS time will be estimated using the Kaplan-Meier method and displayed graphically by treatment arm. The median PFS and associated 95% CIs will be presented by treatment arm. PFS rates at different time points will be estimated with corresponding 2-sided 95% CIs.

Sensitivity/Supplementary Analyses

The following analyses will be performed to further assess the comparison described by the primary objective. Additional sensitivity analyses may be specified in the SAP.

- The PFS analysis will be repeated based on the Centrally Assessed *BRAF* V600E Positive analysis set.
- The same methodology and summary as the main analysis will be used, but PD or death that occurs after the intercurrent events of starting new anticancer therapy will be considered PFS events. All other intercurrent events will be addressed in the same approach as the primary estimand of PFS.



090177e1a02cb071\Approved\Approved On: 14-Mar-2024 15:31 (GMT)

ORR is defined as the proportion of participants who have achieved a BOR of confirmed CR or PR per RECIST v1.1 as assessed by BICR. The BOR is the best response obtained among all tumor assessment visits after date of randomization until documented PD, or start of subsequent anticancer therapy, or death. Confirmation of the response will be no sooner than 4 weeks after the initial documentation of CR or PR.

A BOR of SD requires that an overall response of SD has been determined at a time point at least 6 weeks after the date of randomization.

A BOR of PD requires that the documented progression should be within 12 weeks after the date of randomization and not qualifying as CR, PR or SD. The ORR will be calculated along with corresponding 2-sided Wilson score 95% CI.

The treatment effect of the Arm B compared to Arm C, as measured by stratified (by the randomization strata) odds ratio and its 95% CI in terms of OR defined as the odds of OR with Arm B divided by the odds of OR with Arm C will be tested using Cochran-Mantel-Haenszel statistics stratified by the randomization strata at 1-sided 0.001 level of significance considering the first 110 randomized participants each in Arm B and in Arm C.

**Cohort 3**

The primary endpoint is ORR, defined as the proportion of participants who have achieved a BOR of confirmed CR or PR per RECIST v1.1 as assessed by BICR. The BOR is the best response obtained among all tumor assessment visits after date of randomization until documented PD, or start of subsequent anticancer therapy, or death. Confirmation of the response will be no sooner than 4 weeks after the initial documentation of CR or PR.

A BOR of SD requires that an overall response of SD has been determined at a time point at least 6 weeks after the date of randomization.

A BOR of PD requires that the documented progression should be within 12 weeks after the date of randomization and not qualifying as CR, PR or SD. The ORR will be calculated along with corresponding 2-sided Wilson score 95% CI.

The treatment effect of Arm D compared to Arm E, as measured by stratified (by the randomization strata) odds ratio and its 95% CI in terms of OR defined as the odds of OR with Arm D divided by the odds of OR with Arm E will be tested using Cochran-Mantel-Haenszel statistics stratified by the randomization strata at 1-sided 0.025 level of significance.

### 9.4.3. Secondary Endpoints

#### 9.4.3.1. PFS Arm A versus Arm C, Arm A versus Arm B, and Cohort 3

PFS by BICR and by investigator of Arm A versus Arm C and of Arm A versus Arm B, and PFS by investigator for Cohort 3 will be analyzed using the same analysis method described in Section 9.4.2, however, as these comparisons are not part of the multiple testing strategy as outlined in Section 9.4.1.1, no formal testing will be performed to compare PFS of these treatment arms.



090177e1a02cb071\Approved\Approved On: 14-Mar-2024 15:31 (GMT)

As the key secondary endpoint in Cohort 3, PFS by BICR will be compared between Arm D and Arm E, only if ORR analysis is statistically significant, using the same analysis method described in Section 9.4.2, and 1-sided alpha=0.025.

#### 9.4.3.2. OS

OS is defined as the time from the date of first dose (SLI) or the date of randomization (Phase 3 and Cohort 3) to death due to any cause. If a participant is not known to have died at the time of the cutoff for analysis, then OS will be censored at the date of last contact when the participant was known to be alive. OS will be calculated in months as follows:

> OS (months) = [date of death or censoring – date of first dose (SLI) or randomization date (Phase 3, Cohort 3) +1] / 30.4375

For SLI, OS time will be estimated using the Kaplan-Meier method for Cohort 1 (EC + FOLFIRI) and Cohort 2 (EC +mFOLFOX6) and by prior treatment line (0 or 1), separately.

Final OS analysis for SLI and Cohort 3 will be performed for each portion of the study approximately 24 months after the enrollment of last participant of that portion.

As the key secondary endpoint in Phase 3 portion of the study, OS will be compared between Arm B and Arm C using a 1-sided stratified log-rank test.

The treatment effect will be estimated using a Cox's proportional hazards model stratified by randomization strata to calculate the hazard ratio for OS of Arm B versus Arm C. In order to account for the group sequential design on this endpoint, the repeated CI method[86] will be used to construct the 2-sided repeated CI for the hazard ratio at the interim and the final analyses of OS.

In addition, the unadjusted 95% CIs for the hazard ratios will also be reported at the interim and the final analyses for OS.

OS time will be estimated using the same Kaplan-Meier method as described for PFS. Timing of OS analyses for Phase 3 are outlined in Section 9.5.1.

OS of Arm A versus Arm C, Arm A versus Arm B, Arm D versus Arm E will also be analyzed using the same analysis method described above. However, as these comparisons are not part of the multiple testing strategy as outlined in Section 9.4.1.1, no formal testing will be performed to compare OS of these treatment arms.

Sensitivity/Supplementary Analyses

The following analyses will be performed to further assess the comparisons described by the key secondary objectives. Additional sensitivity analyses may be specified in the SAP.

- The OS analysis will be repeated based on the Centrally Assessed *BRAF* V600E Positive analysis set.



090177e1a02cb071\Approved\Approved On: 14-Mar-2024 15:31 (GMT)

#### 9.4.3.3. Objective Response Rate

ORR is defined as the proportion of participants who have achieved a confirmed BOR of CR or PR per RECIST v1.1. The BOR is the best response obtained among all tumor assessment visits after the date of first dose (SLI) or the date of randomization (Phase 3, Cohort 3) until documented disease progression, death, or start of subsequent anticancer therapy. Clinical deterioration or clinical progression noted on the completion eCRF will not be considered as documented disease progression. Confirmation of the response will be performed per RECIST v1.1, preferably at the regularly scheduled assessment interval, but no sooner than 4 weeks after the initial documentation of CR or PR. Confirmation of PR or CR can be confirmed at an assessment later than the next assessment after the initial documentation of PR or CR, respectively.

A BOR of SD requires that a time point overall response of SD has been determined at a time point at least 6 weeks after the date of first dose (SLI) or the date of randomization (Phase 3, Cohort 3).

A BOR of PD requires that the documented progression should be within 12 weeks after the date of first dose (SLI,) or the date of randomization (Phase 3, Cohort 3) and not qualifying as CR, PR or SD.

The ORR will be calculated along with corresponding 2-sided Wilson score 95% CI.

#### 9.4.3.4. Duration of Response

DOR is defined as the time from the date of first radiographic evidence of response (CR or PR) to the earliest documented disease progression per RECIST v1.1, or death due to any cause. DOR will be calculated for participants with a confirmed response (CR or PR).

DOR will be calculated in months as follows:

DOR (months) = (date of event or censoring – date of first CR or PR + 1) / 30.4375

The same censoring rules specified for PFS in Section 9.4.2 apply to DOR. Kaplan-Meier method will be used to estimate DOR time. The median DOR and its 95% CIs will be presented by treatment arm and cohorts. DOR rates at different time points will be estimated with corresponding 2-sided 95% CIs.

#### 9.4.3.5. Time to Response

TTR is defined as the time from the date of first dose (SLI) or the date of randomization (Phase 3, Cohort 3) to first radiographic evidence of response (CR or PR) per RECIST v1.1. TTR will be calculated for participants with a confirmed response (CR or PR).

TTR will be calculated in weeks as follows:

TTR (in weeks) = (data of first CR or PR – date of first dose (SLI) or date of randomization (Phase 3, Cohort 3) + 1) / 7



090177e1a02cb071\Approved\Approved On: 14-Mar-2024 15:31 (GMT)

TTR will be summarized using simple descriptive statistics (mean, standard deviation, median, minimum, maximum, first quartile, third quartile).

#### 9.4.3.6. PFS (SLI)

PFS in the SLI is defined as the time from the date of first dose to the earliest documented disease progression per RECIST v1.1, or death due to any cause. PFS will be calculated in months as follows:

PFS (months) = (date of event or censoring – date of first dose +1) / 30.4375

PFS time in the SLI will be estimated using the Kaplan-Meier method for Cohort 1 (EC + FOLFIRI) and Cohort 2 (EC + mFOLFOX6) and by prior treatment line (0 or 1), separately. The same censoring rules specified for PFS in Section 9.4.2 will be used with the date of first dose date as the starting date.

#### 9.4.3.7. PFS2

PFS2 is defined as the time from the date of randomization to the date of discontinuation of next-line treatment after first objective PD by investigator assessment, to second objective disease progression, or death from any cause, whichever occurs first. Second objective disease progression is PD after the start of subsequent systemic anticancer therapy. PFS2 will be calculated in months as follows:

PFS2 (months) = [minimum of (date of death, date of second objective PD, date of discontinuation of next treatment) or censoring– randomization date +1] / 30.4375

Participants alive, on next treatment and for whom a second objective PD has not been observed will be censored at the last time known to be alive and without second objective PD. In addition, participants who die after missing 2 or more tumor assessments while on study treatment will remain censored for PFS2.

PFS2 time will be estimated using the same Kaplan-Meier method as described for PFS.

#### 9.4.3.8. Patient Reported Outcomes

FAS will be the primary set for PRO analyses in Phase 3 and Cohort 3. Detailed methodology for summary and statistical analyses of the PRO data collected in this study is outlined here and further detailed in the SAP.

The EORTC QLQ-C30, EQ-5D-5L, PGIC and PGIS will be scored according to their respective user guides/scoring manuals. For the EORTC QLQ-C30 and EQ-5D-5L, missing items will be handled per the respective scoring manuals of each questionnaire. For the PGIS and PGIC, no adjustments for missing data will be performed.

#### 9.4.3.9. Pharmacokinetic Analyses

Plasma concentrations of encorafenib and a metabolite LHY746, irinotecan and an active metabolite SN-38, and total and free plasma concentrations of platinum of oxaliplatin will be



090177e1a02cb071\Approved\Approved On: 14-Mar-2024 15:31 (GMT)

determined using validated assays. Descriptive statistics of concentrations of encorafenib and LHY746, irinotecan and SN-38, and oxaliplatin will be reported and summarized graphically. Pharmacokinetic parameters from the SLI arms and in the up to 16 Chinese participants in Phase 3 Arm A and Arm B will be generated by noncompartmental and/or compartmental approaches as appropriate, summarized and reported. Potential drug interactions between encorafenib and irinotecan or oxaliplatin will be assessed in the respective SLI arms by intra-participant comparisons or comparisons to historical results. Details of analyses will be included in statistical analysis plans and results will be reported.

#### 9.4.3.10. Exposure-Response of Encorafenib

Descriptive statistics of trough plasma concentrations of encorafenib and a metabolite LHY746 will be reported for the Phase 3 portion of this study and in Cohort 3 Arm D. Phase 3 and Cohort 3 Arm D PK parameters will be estimated for encorafenib and LHY746 as appropriate using a population model-based approach and will be included in separate population PK reports, if appropriate. Relationships between PK of encorafenib and biomarkers, clinical response and/or safety will be conducted using post hoc exposure estimates (eg, $C_{max}$, AUC or $C_{min}$) from population PK modeling, if possible and appropriate. Measures of efficacy may include but are not limited to OS, PFS and ORR. Selection of safety measures will be based on frequency of observations or by selection of safety measures of interest. If appropriate, the impact of mFOLFOX6 on encorafenib exposure or responses will be evaluated by comparing Arm B to Arm A, or to historical data. Analyses will be described in separate stand-alone modeling plans and specific reports will be produced. In addition, if data permits, ER analysis with select safety and efficacy endpoints from Cohort 3 Arm D will be conducted and included in a separate report.

#### 9.4.3.11. Biomarker Analyses

Patient plasma samples collected at C1D1 and C2D15 will be assayed to determine if ctDNA is detectable or not detectable. If ctDNA is detected it will be further assayed for the presence of tumor biomarkers such as *BRAF* V600 VAF and/or tumor-specific methylation. Patients will be assigned to subgroups based on whether ctDNA was detected or not at C1D1 and C2D15.

Summary statistics will be reported by subgroups for each arm. Analyses of ORR, PFS, and/or OS by treatment arm will be performed in subgroups considered to be adequately powered to provide informative results. Details of these analyses will be described in the SAP.

### 9.4.4. Tertiary/Exploratory Endpoints

The details of tertiary/exploratory endpoints analyses will be described in the SAP or in a separate exploratory analysis plan.

Results from exploratory analyses will be reported in the CSR where possible. However, given the exploratory nature of the objective and endpoints, the analyses may not be completed at the time of the CSR. Results from exploratory analyses that are not included in



090177e1a02cb071\Approved\Approved On: 14-Mar-2024 15:31 (GMT)

the CSR will be shared with the scientific community through publication at scientific conferences and/or in peer-reviewed scientific journals.

### 9.4.5. Safety Analyses

All safety analyses will be performed on the safety analysis set.

Safety data will be presented in tabular and/or graphical format and summarized descriptively, where appropriate.

#### 9.4.5.1. Adverse Events

Adverse events will be coded using current MedDRA. Severity of AEs will be graded using the NCI CTCAE v 4.03 toxicity grading scale. Incidence tables will be presented for all AEs by maximum severity, SAEs, AEs assessed as related to study intervention and AEs resulting in discontinuation of study intervention. Details will be described in the SAP.

#### 9.4.5.2. Clinical Laboratory Results

Clinical laboratory data will be classified by grade according to NCI CTCAE version 4.03 and will be analyzed using summary statistics. The worst on-treatment grades during the treatment period will be summarized. Shifts in toxicity grading from baseline to highest grade during the on-treatment period will be displayed. Results for laboratory tests that are not part of NCI CTCAE will be presented as below, within, or above normal limits. Only participants with post-baseline laboratory values will be included in these analyses. Further details of analyses for all the laboratory parameters will be provided in the SAP.

#### 9.4.5.3. Electrocardiogram Analyses

ECG parameters QT interval, heart rate, QTcF interval, PR interval, and QRS complex will be summarized by treatment and time point. Further details of analyses for ECG parameters will be provided in the SAP.

### 9.4.6. Other Analyses

Descriptive statistics will be used to summarize all participant characteristics, treatment administration/compliance, vital signs, body weight and ECOG performance status. Data will also be displayed graphically, where appropriate.

Other exploratory PRO analyses may be performed subsequently as needed.

Pharmacogenomic or biomarker data from Banked Biospecimens may be collected during or after the trial and retained for future analyses; the results of such analyses are not planned to be included in the CSR.

#### 9.4.6.1. Subgroup Analyses

Subgroup analyses will be performed to further assess the comparisons described by the primary and key secondary objectives. The HR of PFS and OS and the corresponding 95%



090177e1a02cb071\Approved\Approved On: 14-Mar-2024 15:31 (GMT)

CI will be computed and displayed per subgroup level using a forest plot. Further details including the precise subgroups used will be detailed in the SAP.

## 9.5. Interim Analyses

### 9.5.1. Interim Efficacy Analysis for OS (Phase 3)

The trial allows for stopping the study for a superior OS result, provided the primary ORR and/or PFS endpoint has already been shown to be statistically significant. A Lan-DeMets (O'Brien-Fleming) alpha-spending function[87] stopping boundaries will be used to control the overall type I error rate.

A maximum of three analyses are planned for OS as described below:

1) a first interim analysis at the time of ORR analysis if that analysis is positive;
2) if ORR analysis and/or the PFS result is positive, a second/first interim analysis could be performed at the time of PFS analysis;
3) a final analysis when 297 deaths are observed.

The exact boundaries will be calculated based on the actual number of events and the information fraction. The exact nominal p-values that will need to be observed to declare statistical significance at the time of these analyses for OS will depend on the number of OS events that have been observed at the time of these analyses and, the alpha for OS that can be used for this comparison and that already spent at the time of earlier analyses.

Interim analysis results may be used for decisions regarding stopping for early success, or adapting the study after the interim analysis. Participants may be discontinued from the study as a result of the interim analysis.

Before any interim analysis is conducted, the details of the objectives, decision criteria, dissemination plan and method of maintaining the study blind as per Pfizer's SOPs will be documented. In addition, the analysis details will be documented and approved in the study SAP.

## 9.6. Data Monitoring Committee or Other Independent Oversight Committee

There will be an E-DMC to review cumulative safety data during the study conduct for the SLI, Phase 3, and Cohort 3, as well as review the efficacy at the interim analyses according to the charter. The E-DMC is independent of the study (ie, does not include any investigators in the study) and includes only members external to study Sponsors. The E-DMC will evaluate the safety data and PK data as it is available at pre-specified intervals and at additional points during the conduct of the SLI. During the Phase 3 portion of the study, the E-DMC will assess safety/tolerability after the first 30 participants have been randomized and treated for at least 1 cycle and then approximately every 6 or 12 months, as specified in the charter.

ORR and DOR for Phase 3 and Cohort 3 portions will be evaluated by E-DMC.



090177e1a02cb071\Approved\Approved On: 14-Mar-2024 15:31 (GMT)

The recommendation made by the E-DMC to alter the conduct of the study will be forwarded to the appropriate Pfizer personnel for final decision. Pfizer will forward such decisions, which may include summaries of aggregate analyses of safety data, to regulatory authorities, as appropriate. The E-DMC charter describes the role of the E-DMC in more detail.

# 10. SUPPORTING DOCUMENTATION AND OPERATIONAL CONSIDERATIONS

## 10.1. Appendix 1: Regulatory, Ethical, and Study Oversight Considerations

### 10.1.1. Regulatory and Ethical Considerations

This study will be conducted in accordance with the protocol and with the following:

- Consensus ethical principles derived from international guidelines, including the Declaration of Helsinki and CIOMS International Ethical Guidelines;
- Applicable ICH GCP guidelines;
- Applicable laws and regulations, including applicable privacy laws.

The protocol, protocol amendments, ICD, SRSD(s), and other relevant documents (eg, advertisements) must be reviewed and approved by the sponsor, submitted to an IRB/ethics committee by the investigator, and reviewed and approved by the IRB/ethics committee before the study is initiated.

Any amendments to the protocol will require IRB/ethics committee approval before implementation of changes made to the study design, except for changes necessary to eliminate an immediate hazard to study participants.

Protocols and any substantial amendments to the protocol will require health authority approval prior to initiation except for changes necessary to eliminate an immediate hazard to study participants.

The investigator will be responsible for the following:

- Providing written summaries of the status of the study to the IRB/ethics committee annually or more frequently in accordance with the requirements, policies, and procedures established by the IRB/ethics committee;
- Notifying the IRB/ethics committee of SAEs or other significant safety findings as required by IRB/ethics committee procedures;
- Providing oversight of the conduct of the study at the site and adherence to requirements of 21 CFR, ICH GCP guidelines, the IRB/ethics committee, European regulation 536/2014 for clinical studies, European Medical Device Regulation 2017/745 for clinical device research, and all other applicable local regulations.

090177e1a02cb071\Approved\Approved On: 14-Mar-2024 15:31 (GMT)

#### 10.1.1.1. Reporting of Safety Issues and Serious Breaches of the Protocol or ICH GCP

In the event of any prohibition or restriction imposed (ie, clinical hold) by an applicable regulatory authority in any area of the world, or if the investigator is aware of any new information that might influence the evaluation of the benefits and risks of the study intervention, Pfizer should be informed immediately.

In addition, the investigator will inform Pfizer immediately of any urgent safety measures taken by the investigator to protect the study participants against any immediate hazard, and of any serious breaches of this protocol or of the ICH GCP that the investigator becomes aware of.

### 10.1.2. Financial Disclosure

Investigators and sub-investigators will provide the sponsor with sufficient, accurate financial information as requested to allow the sponsor to submit complete and accurate financial certification or disclosure statements to the appropriate regulatory authorities. Investigators are responsible for providing information on financial interests during the course of the study and for 1 year after completion of the study.

### 10.1.3. Informed Consent/Assent Process

#### 10.1.3.1. Adult Participants

The investigator or the investigator's representative will explain the nature of the study including the risks and benefits, to the participant or their legally authorized representative and answer all questions regarding the study. The participant or their legally authorized representative should be given sufficient time and opportunity to ask questions and to decide whether or not to participate in the trial.

Participants must be informed that their participation is voluntary. Participants or their legally authorized representative (if allowed by local regulations) will be required to sign a statement of informed consent that meets the requirements of 21 CFR 50, local regulations, ICH GCP guidelines, privacy and data protection requirements, where applicable, and the IRB/ethics committee or study center.

The investigator must ensure that each study participant or their legally authorized representative is fully informed about the nature and objectives of the study, the sharing of data related to the study, and possible risks associated with participation, including the risks associated with the processing of the participant's personal data.

The participant or their legally authorized representative must be informed that their personal study-related data will be used by the sponsor in accordance with local data protection law. The level of disclosure must also be explained to the participant or their legally authorized representative.

The participant or their legally authorized representative must be informed that their medical records may be examined by Clinical Quality Assurance auditors or other authorized



090177e1a02cb071\Approved\Approved On: 14-Mar-2024 15:31 (GMT)

personnel appointed by the sponsor, by appropriate IRB/ethics committee members, and by inspectors from regulatory authorities.

The investigator further must ensure that each study participant or their legally authorized representative is fully informed about his or her right to access and correct their personal data and to withdraw consent for the processing of his or her personal data.

The medical record must include a statement that written informed consent was obtained before the participant was enrolled in the study and the date on which the written consent was obtained. The authorized person obtaining the informed consent must also sign the ICD.

Participants or their legally authorized representative must be reconsented to the most current IRB/ethics committee version of the IRB/ethics committee-approved ICD(s) during their participation in the study as required per local regulations.

A copy of the ICD(s) must be provided to the participant or their legally authorized representative (if allowed by local regulations).

A participant who is rescreened is not required to sign another ICD if the rescreening occurs within 28 days from the previous ICD signature date.

Unless prohibited by local requirements or IRB/ethics committee decision, the ICD will contain a separate section that addresses the use of samples for optional additional research. The optional additional research does not require the collection of any further samples. The investigator or authorized designee will explain to each participant the objectives of the additional research. Participants will be told that they are free to refuse to participate and may withdraw their consent at any time and for any reason during the storage period. A separate signature will be required to document a participant's agreement to allow specimens to be used for additional research. Participants who decline to participate in this optional additional research will not provide this separate signature.

#### 10.1.3.2. Pediatric Participants

The investigator or their representative will explain the nature of the study to the participant and their parent(s)/legal guardian and answer all questions regarding the study. The participant and their parent(s)/legal guardian should be given sufficient time and opportunity to ask questions and to decide whether or not to participate in the trial.

When consent is obtained from a participant's parent(s)/legal guardian, the participant's assent (affirmative agreement) must be subsequently obtained when the participant has the capacity to provide assent, as determined by the IRB/ethics committee. If the investigator determines that a participant's decisional capacity is so limited they cannot reasonably be consulted, then, as permitted by the IRB/ethics committee and consistent with local regulatory and legal requirements, the participant's assent may be waived with source documentation of the reason assent was not obtained. If the study participant does not provide their own assent, the source documents must record why the participant did not provide assent (for example, the child is not of assenting age per local regulations or



090177e1a02cb071\Approved\Approved On: 14-Mar-2024 15:31 (GMT)

policies), how the investigator determined that the person signing the consent was the participant's parent(s)/legal guardian, the consent signer's relationship to the study participant, and that the participant's assent was obtained or waived. If assent is obtained verbally, it must be documented in the source documents.

If study participants are minors who reach the age of majority or if a child reaches the age of assent (per local IRB/ethics committee requirements) during the study, as recognized under local law, the child or adolescent must then provide the appropriate assent or consent to document their willingness to continue in the study. For an adolescent, who reaches the age of consent, parental consent would no longer be valid. If the enrollment of emancipated minors is permitted by the IRB/ethics committee and local law, the participant must provide documentation of legal status to give consent without the permission of a legally authorized representative.

Participants and their parent(s)/legal guardian must be informed that their participation is voluntary. The participant's parent(s)/legal guardian will be required to sign a statement of informed consent that meets the requirements of 21 CFR 50, local regulations, ICH guidelines, HIPAA requirements, where applicable, and the IRB/ethics committee or study center.

The investigator must ensure that each study participant's parent(s)/legal guardian and the study participant as applicable are fully informed about the nature and objectives of the study, the sharing of data related to the study, and possible risks associated with participation, including the risks associated with the processing of the participant's personal data.

The participant's parent(s)/legal guardian must be informed that the participant's personal study-related data will be used by the sponsor in accordance with local data protection law. The level of disclosure must also be explained to the participant's parent(s)/legal guardian.

The participant's parent(s)/legal guardian must be informed that the participant's medical records may be examined by Clinical Quality Assurance auditors or other authorized personnel appointed by the sponsor, by appropriate IRB/ethics committee members, and by inspectors from regulatory authorities.

The investigator further must ensure that each study participant's parent(s)/legal guardian is fully informed about their right to access and correct their child's personal data and to withdraw consent for the processing of their child's personal data, keeping in mind the privacy rights that may restrict access of older adolescents' medical records by their parent(s)/legal guardian in certain regions.

The source documentation must include a statement that written informed consent, and as applicable assent, was obtained before the participant was enrolled in the study and the date the written consent/assent was obtained. The authorized person obtaining the informed consent must also sign the ICD.



090177e1a02cb071\Approved\Approved On: 14-Mar-2024 15:31 (GMT)

Parent(s)/legal guardian and the participant must be reconsented to the most current version of the ICD(s)/assent during their participation in the study as required per local regulations.

A copy of the ICD(s) and assent, if written, must be provided to the parent(s)/legal guardian and the participant.

A participant who is rescreened is not required to sign another ICD if the rescreening occurs within 28 days from the previous ICD signature date.

Unless prohibited by local requirements or IRB/ethics committee decision, the ICD will contain a separate section that addresses the use of samples for optional additional research. The optional additional research does not require the collection of any further samples. The investigator or authorized designee will explain to each participant the objectives of the additional research. Participants will be told that they are free to refuse to participate and may withdraw their consent/assent at any time and for any reason during the storage period. A separate signature will be required to document a participant's agreement to allow specimens to be used for additional research. Participants who decline to participate in this optional additional research will not provide this separate signature.

### 10.1.4. Data Protection

All parties will comply with all applicable laws, including laws regarding the implementation of organizational and technical measures to ensure protection of participant data.

Participants' personal data will be stored at the study site in encrypted electronic and/or paper form and will be password protected or secured in a locked room to ensure that only authorized study staff have access. The study site will implement appropriate technical and organizational measures to ensure that the personal data can be recovered in the event of disaster. In the event of a potential personal data breach, the study site will be responsible for determining whether a personal data breach has in fact occurred and, if so, providing breach notifications as required by law.

To protect the rights and freedoms of participants with regard to the processing of personal data, participants will be assigned a single, participant-specific numerical code. Any participant records or data sets that are transferred to the sponsor will contain the numerical code; participant names will not be transferred. All other identifiable data transferred to the sponsor will be identified by this single, participant-specific code. The study site will maintain a confidential list of participants who participated in the study, linking each participant's numerical code to their actual identity and medical record ID. In case of data transfer, the sponsor will protect the confidentiality of participants' personal data consistent with the clinical study agreement and applicable privacy laws.

Information technology systems used to collect, process, and store study-related data are secured by technical and organizational security measures designed to protect such data against accidental or unlawful loss, alteration, or unauthorized disclosure or access.



090177e1a02cb071\Approved\Approved On: 14-Mar-2024 15:31 (GMT)

The sponsor maintains SOPs on how to respond in the event of unauthorized access, use, or disclosure of sponsor information or systems.

### 10.1.5. Dissemination of Clinical Study Data

Pfizer fulfills its commitment to publicly disclose clinical study results through posting the results of studies on www.clinicaltrials.gov (ClinicalTrials.gov), the EudraCT/CTIS, and/or www.pfizer.com, and other public registries and websites in accordance with applicable local laws/regulations. In addition, Pfizer reports study results outside of the requirements of local laws/regulations pursuant to its SOPs.

In all cases, study results are reported by Pfizer in an objective, accurate, balanced, and complete manner and are reported regardless of the outcome of the study or the country in which the study was conducted.

www.clinicaltrials.gov

Pfizer posts clinical trial results on www.clinicaltrials.gov for Pfizer-sponsored interventional studies (conducted in patients) that evaluate the safety and/or efficacy of a product, regardless of the geographical location in which the study is conducted. These results are submitted for posting in accordance with the format and timelines set forth by US law.

EudraCT/CTIS

Pfizer posts clinical trial results on EudraCT/CTIS for Pfizer-sponsored interventional studies in accordance with the format and timelines set forth by EU requirements.

www.pfizer.com

Pfizer posts CSR synopses and plain-language study results summaries on www.pfizer.com for Pfizer-sponsored interventional studies at the same time the corresponding study results are posted to www.clinicaltrials.gov. CSR synopses will have personally identifiable information anonymized.

Documents within marketing applications

Pfizer complies with applicable local laws/regulations to publish clinical documents included in marketing applications. Clinical documents include summary documents and CSRs including the protocol and protocol amendments, sample CRFs, and SAPs. Clinical documents will have personally identifiable information anonymized.

Data Sharing

Pfizer provides researchers secure access to participant-level data or full CSRs for the purposes of "bona-fide scientific research" that contributes to the scientific understanding of the disease, target, or compound class. Pfizer will make data from these trials available 18 months after study completion. Participant-level data will be anonymized in accordance



090177e1a02cb071\Approved\Approved On: 14-Mar-2024 15:31 (GMT)

with applicable privacy laws and regulations. CSRs will have personally identifiable information anonymized.

Data requests are considered from qualified researchers with the appropriate competencies to perform the proposed analyses. Research teams must include a biostatistician. Data will not be provided to applicants with significant conflicts of interest, including individuals requesting access for commercial/competitive or legal purposes.

### 10.1.6. Data Quality Assurance

All participant data relating to the study will be recorded on printed or electronic CRF unless transmitted to the sponsor or designee electronically (eg, laboratory data). The investigator is responsible for verifying that data entries are accurate and correct by physically or electronically signing the CRF.

Guidance on completion of CRFs will be provided in the CRF Completion Requirements document.

The investigator must ensure that the CRFs are securely stored at the study site in encrypted electronic and/or paper form and are password protected or secured in a locked room to prevent access by unauthorized third parties.

QTLs are predefined parameters that are monitored during the study. Important deviations from the QTLs and any remedial actions taken will be summarized in the CSR.

The investigator must permit study-related monitoring, audits, IRB/ethics committee review, and regulatory agency inspections and provide direct access to source records and documents. This verification may also occur after study completion. It is important that the investigator(s) and their relevant personnel are available during the monitoring visits and possible audits or inspections and that sufficient time is devoted to the process.

Monitoring details describing strategy including definition of study-critical data items and processes (eg, risk-based initiatives in operations and quality such as risk management and mitigation strategies and analytical risk-based monitoring), methods, responsibilities, and requirements, including handling of noncompliance issues and monitoring techniques (central, remote, or on-site monitoring), are provided in the data management plan and monitoring plan maintained and utilized by the sponsor or designee.

The sponsor or designee is responsible for the data management of this study, including quality checking of the data.

Records and documents, including signed ICDs, pertaining to the conduct of this study must be retained by the investigator for 15 years after study completion unless local regulations or institutional policies require a longer retention period. No records may be destroyed during the retention period without the written approval of the sponsor. No records may be transferred to another location or party without written notification to the sponsor. The



090177e1a02cb071\Approved\Approved On: 14-Mar-2024 15:31 (GMT)

investigator must ensure that the records continue to be stored securely for as long as they are maintained.

When participant data are to be deleted, the investigator will ensure that all copies of such data are promptly and irrevocably deleted from all systems.

The investigator(s) will notify the sponsor or its agents immediately of any regulatory inspection notification in relation to the study. Furthermore, the investigator will cooperate with the sponsor or its agents to prepare the investigator site for the inspection and will allow the sponsor or its agent, whenever feasible, to be present during the inspection. The investigator site and investigator will promptly resolve any discrepancies that are identified between the study data and the participant's medical records. The investigator will promptly provide copies of the inspection findings to the sponsor or its agent. Before response submission to the regulatory authorities, the investigator will provide the sponsor or its agents with an opportunity to review and comment on responses to any such findings.

### 10.1.7. Source Documents

Source documents provide evidence for the existence of the participant and substantiate the integrity of the data collected. Source documents are filed at the investigator site.

Data reported on the CRF or entered in the eCRF that are from source documents must be consistent with the source documents or the discrepancies must be explained. The investigator may need to request previous medical records or transfer records, depending on the study. Also, current medical records must be available.

Definition of what constitutes source data can be found in the clinical monitoring plan.

Description of the use of computerized system is documented in the Data Management Plan.

### 10.1.8. Study and Site Start and Closure

The study start date is the date of the first participant's first visit.

The sponsor designee reserves the right to close the study site or terminate the study at any time for any reason at the sole discretion of the sponsor, including (but not limited to) regulatory authority decision, change in opinion of the IRB/ethics committee, or change in benefit-risk assessment. Study sites will be closed upon study completion. A study site is considered closed when all required documents and study supplies have been collected and a study-site closure visit has been performed.

The investigator may initiate study-site closure at any time upon notification to the sponsor or designee/CRO if requested to do so by the responsible IRB/ethics committee or if such termination is required to protect the health of study participants.

Reasons for the early closure of a study site by the sponsor may include but are not limited to:



090177e1a02cb071\Approved\Approved On: 14-Mar-2024 15:31 (GMT)

- Failure of the investigator to comply with the protocol, the requirements of the IRB/ethics committee or local health authorities, the sponsor's procedures, or the ICH GCP guidelines;
- Inadequate recruitment of participants by the investigator;
- Discontinuation of further study intervention development.

If the study is prematurely terminated or suspended, the sponsor shall promptly inform the investigators, the ethics committees/IRBs, the regulatory authorities, and any CRO(s) used in the study of the reason for termination or suspension, as specified by the applicable regulatory requirements. The investigator shall promptly inform the participant and should assure appropriate participant therapy and/or follow-up.

Study termination is also provided for in the clinical study agreement. If there is any conflict between the contract and this protocol, the contract will control as to termination rights.

### 10.1.9. Publication Policy

For multicenter trials, the primary publication will be a joint publication developed by the investigator and Pfizer reporting the primary endpoint(s) of the study covering all study sites. The investigator agrees to refer to the primary publication in any subsequent publications. Pfizer will not provide any financial compensation for the investigator's participation in the preparation of the primary congress abstract, poster, presentation, or primary manuscript for the study.

Investigators are free to publish individual center results that they deem to be clinically meaningful after publication of the overall results of the study or 12 months after primary completion date or study completion at all sites, whichever occurs first, subject to the other requirements described in this section.

The investigator will provide Pfizer an opportunity to review any proposed publication or any other type of disclosure of the study results (collectively, "publication") before it is submitted or otherwise disclosed and will submit all publications to Pfizer 30 days before submission. If any patent action is required to protect intellectual property rights, the investigator agrees to delay the disclosure for a period not to exceed an additional 60 days upon request from Pfizer. This allows Pfizer to protect proprietary information and to provide comments, and the investigator will, on request, remove any previously undisclosed confidential information before disclosure, except for any study-intervention or Pfizer-related information necessary for the appropriate scientific presentation or understanding of the study results. For joint publications, should there be disagreement regarding interpretation and/or presentation of specific analysis results, resolution of, and responsibility for, such disagreements will be the collective responsibility of all authors of the publication.

For all publications relating to the study, the investigator and Pfizer will comply with recognized ethical standards concerning publications and authorship, including those



090177e1a02cb071\Approved\Approved On: 14-Mar-2024 15:31 (GMT)

established by the International Committee of Medical Journal Editors. The investigator will disclose any relationship with Pfizer and any relevant potential conflicts of interest, including any financial or personal relationship with Pfizer, in any publications. All authors will have access to the relevant statistical tables, figures, and reports (in their original format) required to develop the publication. The results of this study may be published or presented at scientific meetings by the investigator after publication of the overall study results or 1 year after the end of the study (or study termination), whichever comes first.

### 10.1.10. Sponsor's Qualified Medical Personnel

The sponsor will designate a medically qualified individual (MQI, also known as the medical monitor) to advise the investigator on study-related medical questions. The contact information for the study medical monitor is documented in the Study Team Contact List located in the Investigator Site File.

Participants are provided with a Pfizer study information card at the time of informed consent which includes contact information for their investigator in case of study-related medical questions. The study information card contains, at a minimum, (a) study number, (b) participant's study identification number, and (c) principal investigator contact information.

090177e1a02cb071\Approved\Approved On: 14-Mar-2024 15:31 (GMT)

## 10.2. Appendix 2: Clinical Laboratory Tests

The following safety laboratory tests will be performed at times defined in the SoA section of this protocol. Additional laboratory results may be reported on these samples as a result of the method of analysis or the type of analyzer used by the clinical laboratory, or as derived from calculated values. These additional tests would not require additional collection of blood. Unscheduled clinical laboratory measurements may be obtained at any time during the study to assess any perceived safety issues.

**Table 35. Protocol-Required Safety Laboratory Assessments**

| Hematology | Chemistry | Urinalysis | Coagulation | Other |
|---|---|---|---|---|
| • Hemoglobin<br>• Hematocrit<br>• RBC count<br>• Platelet count<br>• WBC count<br>• Total neutrophils (Abs) | • BUN[a]<br>• Creatinine<br>• Glucose<br>• Calcium<br>• Sodium<br>• Magnesium<br>• Potassium<br>• Chloride<br>• Total $CO_2$ (bicarbonate)[b]<br>• AST, ALT<br>• Albumin<br>• Total bilirubin<br>• Direct bilirubin (if total bilirubin values are above normal)<br>• Alkaline phosphatase<br>• Uric acid<br>• Total protein<br>• Lipase<br>• LDH | • pH<br>• Glucose (qual)<br>• Protein (qual)<br>• Blood (qual)<br>• Ketones | • PT or INR<br>• PTT or aPTT | • Pregnancy test (β-hCG)[c]<br><br><u>At screening only:</u><br>• FSH[d]<br>• Hepatitis B surface antigen (at Screening)[e]<br>• Hepatitis C antibody (at Screening)[f]<br>• HIV, as applicable per local regulations<br><br><u>Tumor markers:</u><br><br>• CRP<br>• CEA |

a Urea may be collected for local laboratory testing

b Not required if not included in the chemistry panel at the site laboratory. Investigators should follow up per institutional standard of care (eg, blood gas analysis) if warranted by required laboratory test results.

c Serum at screening and urine β-hCG during the study for female participants of childbearing potential. Local urine testing will be standard for the protocol unless serum testing is required by local regulation or IRB/ethics committee.

d For confirmation of postmenopausal status only.

e If HBsAg positive, HBV DNA must be measured

f If HCV antibody positive, HCV RNA must be measured



090177e1a02cb071\Approved\Approved On: 14-Mar-2024 15:31 (GMT)

The investigator must review the central laboratory report, document this review and record any clinically significant changes occurring during the study in the AE section of the CRF.

090177e1a02cb071\Approved\Approved On: 14-Mar-2024 15:31 (GMT)

090177e1a02cb071\Approved\Approved On: 14-Mar-2024 15:31 (GMT)

## 10.3. Appendix 3: Adverse Events: Definitions and Procedures for Recording, Evaluating, Follow-up, and Reporting

### 10.3.1. Definition of AE

| AE Definition |
|---|
| • An AE is any untoward medical occurrence in a patient or clinical study participant, temporally associated with the use of study intervention, whether or not considered related to the study intervention.<br><br>• NOTE: An AE can therefore be any unfavorable and unintended sign (including an abnormal laboratory finding), symptom, or disease (new or exacerbated) temporally associated with the use of study intervention. |

| Events <u>Meeting</u> the AE Definition |
|---|
| • Any abnormal laboratory test results (hematology, clinical chemistry, or urinalysis) or other safety assessments (eg, ECG, radiological scans, vital sign measurements), including those that worsen from baseline, considered clinically significant in the medical and scientific judgment of the investigator Any abnormal laboratory test results that meet any of the conditions below must be recorded as an AE:<br>  • Is associated with accompanying symptoms.<br>  • Requires additional diagnostic testing or medical/surgical intervention.<br>  • Leads to a change in study dosing (outside of any protocol-specified dose adjustments) or discontinuation from the study, significant additional concomitant drug treatment, or other therapy.<br><br>• Exacerbation of a chronic or intermittent preexisting condition including either an increase in frequency and/or intensity of the condition.<br><br>• New conditions detected or diagnosed after study intervention administration even though it may have been present before the start of the study.<br><br>• Signs, symptoms, or the clinical sequelae of a suspected drug-drug interaction.<br><br>• Signs, symptoms, or the clinical sequelae of a suspected overdose of either study intervention or a concomitant medication. Overdose per se will not be reported as an AE/SAE unless it is an intentional overdose taken with possible suicidal/self-harming intent. Such overdoses should be reported regardless of sequelae. |

| **Events NOT Meeting the AE Definition** |
|---|
| • Any clinically significant abnormal laboratory findings or other abnormal safety assessments which are associated with the underlying disease, unless judged by the investigator to be more severe than expected for the participant's condition.<br>• The disease/disorder being studied or expected progression, signs, or symptoms of the disease/disorder being studied, unless more severe than expected for the participant's condition.<br>• Medical or surgical procedure (eg, endoscopy, appendectomy): the condition that leads to the procedure is the AE.<br>• Situations in which an untoward medical occurrence did not occur (social and/or convenience admission to a hospital).<br>• Anticipated day-to-day fluctuations of preexisting disease(s) or condition(s) present or detected at the start of the study that do not worsen.<br>• Worsening of signs and symptoms of the malignancy under study should be recorded as AEs in the appropriate section of the CRF. Disease progression assessed by measurement of malignant lesions on radiographs or other methods should not be reported as AEs. |

### 10.3.2. Definition of SAE

If an event is not an AE per definition above, then it cannot be an SAE even if serious conditions are met (eg, hospitalization for signs/symptoms of the disease under study, death due to progression of disease).

| **An SAE is defined as any untoward medical occurrence that, at any dose:** |
|---|
| **a. Results in death** |
| **b. Is life-threatening**<br>The term "life-threatening" in the definition of "serious" refers to an event in which the participant was at risk of death at the time of the event. It does not refer to an event that hypothetically might have caused death if it were more severe. |
| **c. Requires inpatient hospitalization or prolongation of existing hospitalization**<br>In general, hospitalization signifies that the participant has been detained (usually involving at least an overnight stay) at the hospital or emergency ward for observation and/or treatment that would not have been appropriate in the physician's office or outpatient setting. Complications that occur during hospitalization are AEs. If a |



090177e1a02cb071\Approved\Approved On: 14-Mar-2024 15:31 (GMT)

complication prolongs hospitalization or fulfills any other serious criteria, the event is serious. When in doubt as to whether "hospitalization" occurred or was necessary, the AE should be considered serious.

Hospitalization for elective treatment of a preexisting condition that did not worsen from baseline is not considered an AE.

**d. Results in persistent disability/incapacity**

- The term disability means a substantial disruption of a person's ability to conduct normal life functions.
- This definition is not intended to include experiences of relatively minor medical significance such as uncomplicated headache, nausea, vomiting, diarrhea, influenza, and accidental trauma (eg, sprained ankle) which may interfere with or prevent everyday life functions but do not constitute a substantial disruption.

**e. Is a congenital anomaly/birth defect**

**f. Other situations:**

- Medical or scientific judgment should be exercised in deciding whether SAE reporting is appropriate in other situations such as important medical events that may not be immediately life-threatening or result in death or hospitalization but may jeopardize the participant or may require medical or surgical intervention to prevent one of the other outcomes listed in the above definition. These events should usually be considered serious.
- Examples of such events include invasive or malignant cancers, intensive treatment in an emergency room or at home for allergic bronchospasm, blood dyscrasias or convulsions that do not result in hospitalization, or development of drug dependency or drug abuse.
- Progression of the malignancy under study (including signs and symptoms of progression) should not be reported as an SAE unless the outcome is fatal within the active collection period. Hospitalization due to signs and symptoms of disease progression should not be reported as an SAE. If the malignancy has a fatal outcome during the study or within the active collection period, then the event leading to death must be recorded as an AE on the CRF, and as an SAE with CTCAE Grade 5 (see the Assessment of Intensity section).
- Suspected transmission via a Pfizer product of an infectious agent, pathogenic or non-pathogenic, is considered serious. The event may be suspected from clinical symptoms or laboratory findings indicating an infection in a patient exposed to a Pfizer product. The terms "suspected transmission" and "transmission" are considered synonymous. These cases are considered unexpected and handled as



090177e1a02cb071\Approved\Approved On: 14-Mar-2024 15:31 (GMT)

serious expedited cases by pharmacovigilance personnel. Such cases are also considered for reporting as product defects, if appropriate.

### 10.3.3. Recording/Reporting and Follow-up of AEs and/or SAEs

**AE and SAE Recording/Reporting**

The table below summarizes the requirements for recording adverse events on the CRF and for reporting serious adverse events on the CT SAE Report Form to Pfizer Safety. These requirements are delineated for 3 types of events: (1) SAEs; (2) nonserious adverse events (AEs); and (3) exposure to the study intervention under study during pregnancy or breastfeeding, and occupational exposure.

It should be noted that the CT SAE Report Form for reporting of SAE information is not the same as the AE page of the CRF. When the same data are collected, the forms must be completed in a consistent manner. AEs should be recorded using concise medical terminology and the same AE term should be used on both the CRF and the CT SAE Report Form for reporting of SAE information.

| Safety Event | Recorded on the CRF | Reported on the CT SAE Report Form to Pfizer Safety Within 24 Hours of Awareness |
|---|---|---|
| SAE | All | All |
| Nonserious AE | All | None |
| Exposure to the study intervention under study during pregnancy or breastfeeding, and occupational exposure | All AEs/SAEs associated with exposure during pregnancy or breastfeeding<br><br>Occupational exposure is not recorded. | All (and EDP supplemental form for EDP)<br>Note: Include all SAEs associated with exposure during pregnancy or breastfeeding. Include all AEs/SAEs associated with occupational exposure. |

- When an AE/SAE occurs, it is the responsibility of the investigator to review all documentation (eg, hospital progress notes, laboratory reports, and diagnostic reports) related to the event.
- The investigator will then record all relevant AE/SAE information in the CRF.



090177e1a02cb071\Approved\Approved On: 14-Mar-2024 15:31 (GMT)

- It is **not** acceptable for the investigator to send photocopies of the participant's medical records to Pfizer Safety in lieu of completion of the CT SAE Report Form/AE/SAE CRF page.
- There may be instances when copies of medical records for certain cases are requested by Pfizer Safety. In this case, all participant identifiers, with the exception of the participant number, will be redacted on the copies of the medical records before submission to Pfizer Safety.
- The investigator will attempt to establish a diagnosis of the event based on signs, symptoms, and/or other clinical information. Whenever possible, the diagnosis (not the individual signs/symptoms) will be documented as the AE/SAE.

**Assessment of Intensity**

The investigator will make an assessment of intensity for each AE and SAE reported during the study and assign it to 1 of the following categories:

| GRADE | Clinical Description of Severity |
|---|---|
| 1 | MILD adverse event |
| 2 | MODERATE adverse event |
| 3 | SEVERE adverse event |
| 4 | LIFE-THREATENING consequences; urgent intervention indicated |
| 5 | DEATH RELATED TO adverse event |

**Assessment of Causality**

- The investigator is obligated to assess the relationship between study intervention and each occurrence of each AE/SAE.
- A "reasonable possibility" of a relationship conveys that there are facts, evidence, and/or arguments to suggest a causal relationship, rather than a relationship cannot be ruled out.
- The investigator will use clinical judgment to determine the relationship.

090177e1a02cb071\Approved\Approved On: 14-Mar-2024 15:31 (GMT)

090177e1a02cb071\Approved\Approved On: 14-Mar-2024 15:31 (GMT)

- Alternative causes, such as underlying disease(s), concomitant therapy, and other risk factors, as well as the temporal relationship of the event to study intervention administration, will be considered and investigated.
- The investigator will also consult the IB and/or product information, for marketed products, in his/her assessment.
- For each AE/SAE, the investigator **must** document in the medical notes that he/she has reviewed the AE/SAE and has provided an assessment of causality.
- There may be situations in which an SAE has occurred and the investigator has minimal information to include in the initial report to the sponsor. However, **it is very important that the investigator always make an assessment of causality for every event before the initial transmission of the SAE data to the sponsor.**
- The investigator may change his/her opinion of causality in light of follow-up information and send an SAE follow-up report with the updated causality assessment.
- The causality assessment is one of the criteria used when determining regulatory reporting requirements.
- If the investigator does not know whether or not the study intervention caused the event, then the event will be handled as "related to study intervention" for reporting purposes, as defined by the sponsor. In addition, if the investigator determines that an SAE is associated with study procedures, the investigator must record this causal relationship in the source documents and CRF, and report such an assessment in the dedicated section of the CT SAE Report Form and in accordance with the SAE reporting requirements.

**Follow-up of AEs and SAEs**

- The investigator is obligated to perform or arrange for the conduct of supplemental measurements and/or evaluations as medically indicated or as requested by the sponsor to elucidate the nature and/or causality of the AE or SAE as fully as possible. This may include additional laboratory tests or investigations, histopathological examinations, or consultation with other healthcare providers.

| |
|---|
| • If a participant dies during participation in the study or during a recognized follow-up period, the investigator will provide Pfizer Safety with a copy of any postmortem findings including histopathology.<br>• New or updated information will be recorded in the originally completed CRF.<br>• The investigator will submit any updated SAE data to the sponsor within 24 hours of receipt of the information. |

### 10.3.4. Reporting of SAEs

| **SAE Reporting to Pfizer Safety via an Electronic Data Collection Tool** |
|---|
| • The primary mechanism for reporting an SAE to Pfizer Safety will be the electronic data collection tool.<br>• If the electronic system is unavailable, then the site will use the paper SAE data collection tool (see next section) in order to report the event within 24 hours.<br>• The site will enter the SAE data into the electronic system as soon as the data become available.<br>• After the study is completed at a given site, the electronic data collection tool will be taken offline to prevent the entry of new data or changes to existing data.<br>• If a site receives a report of a new SAE from a study participant or receives updated data on a previously reported SAE after the electronic data collection tool has been taken offline, then the site can report this information on a paper SAE form (see next section) or to Pfizer Safety by telephone. |

| **SAE Reporting to Pfizer Safety via CT SAE Report Form** |
|---|
| • Facsimile transmission of the CT SAE Report Form is the preferred method to transmit this information to Pfizer Safety.<br>• In circumstances when the facsimile is not working, notification by telephone is acceptable with a copy of the CT SAE Report Form sent by overnight mail or courier service.<br>• Initial notification via telephone does not replace the need for the investigator to complete and sign the CT SAE Report Form pages within the designated reporting time frames. |



090177e1a02cb071\Approved\Approved On: 14-Mar-2024 15:31 (GMT)

## 10.4. Appendix 4: Contraceptive Guidance

### 10.4.1. Male Participant Reproductive Inclusion Criteria

Male participants are eligible to participate if they agree to the following requirements during the intervention period and for at least 6 months after the last dose of study intervention, which corresponds to the time needed to eliminate reproductive safety risk of the study intervention(s):

- Refrain from donating sperm.

PLUS either**:**

- Be abstinent from heterosexual intercourse with a female of childbearing potential as their preferred and usual lifestyle (abstinent on a long-term and persistent basis) and agree to remain abstinent.

  OR

- Must agree to use a male condom when engaging in any activity that allows for passage of ejaculate to another person.

### 10.4.2. Female Participant Reproductive Inclusion Criteria

A female participant is eligible to participate if she is not pregnant or breastfeeding, and at least 1 of the following conditions applies:

- Is not a WOCBP (see definitions below in Section 10.4.3).

  OR

- Is a WOCBP and using a contraceptive method that is highly effective (with a failure rate of <1% per year), preferably with low user dependency, as described below during the intervention period and for at least 9 months after the last dose of study intervention, which corresponds to the time needed to eliminate any reproductive safety risk of the study intervention(s). The investigator should evaluate the effectiveness of the contraceptive method in relationship to the first dose of study intervention.

- Is a WOCBP and using a contraceptive method that is highly effective (with a failure rate of <1% per year), with high user dependency, as described below during the intervention period and for at least 9 months after the last dose of study intervention, which corresponds to the time needed to eliminate any reproductive safety risk of the study intervention(s). In addition, a second effective method of contraception, as described below, must be used. The investigator should evaluate the effectiveness of the contraceptive method in relationship to the first dose of study intervention.

090177e1a02cb071\Approved\Approved On: 14-Mar-2024 15:31 (GMT)

The investigator is responsible for review of medical history, menstrual history, and recent sexual activity to decrease the risk for inclusion of a woman with an early undetected pregnancy.

### 10.4.3. Woman of Childbearing Potential

A woman is considered fertile following menarche and until becoming postmenopausal unless permanently sterile (see below).

If fertility is unclear (eg, amenorrhea in adolescents or athletes) and a menstrual cycle cannot be confirmed before the first dose of study intervention, additional evaluation should be considered.

Women in the following categories are not considered WOCBP:

1. Premenarchal.

2. Premenopausal female with 1 of the following:

   - Documented hysterectomy;
   - Documented bilateral salpingectomy;
   - Documented bilateral oophorectomy.

   For individuals with permanent infertility due to an alternate medical cause other than the above, (eg, mullerian agenesis, androgen insensitivity), investigator discretion should be applied to determining study entry.

   Note: Documentation for any of the above categories can come from the site personnel's review of the participant's medical records, medical examination, or medical history interview. The method of documentation should be recorded in the participant's medical record for the study.

3. Postmenopausal female:

- A postmenopausal state is defined as no menses for 12 months without an alternative medical cause. In addition, a
  - high FSH level in the postmenopausal range must be used to confirm a postmenopausal state in women under 60 years of age and not using hormonal contraception or HRT.
  - Female on HRT and whose menopausal status is in doubt will be required to use one of the nonestrogen hormonal highly effective contraception methods if they wish to continue their HRT during the study. Otherwise, they must

090177e1a02cb071\Approved\Approved On: 14-Mar-2024 15:31 (GMT)

discontinue HRT to allow confirmation of postmenopausal status before study enrollment.

### 10.4.4. Contraception Methods

Contraceptive use by men or women should be consistent with local availability/regulations regarding the use of contraceptive methods for those participating in clinical trials.

**Highly Effective Methods That Have Low User Dependency**

1. Implantable progestogen-only hormone contraception associated with inhibition of ovulation.

2. Intrauterine device.

3. Intrauterine hormone-releasing system.

4. Bilateral tubal occlusion.

5. Vasectomized partner.

   - Vasectomized partner is a highly effective contraceptive method provided that the partner is the sole sexual partner of the WOCBP and the absence of sperm has been confirmed. If not, an additional highly effective method of contraception should be used. The spermatogenesis cycle is approximately 90 days.

**Highly Effective Methods That Are User Dependent**

6. Combined (estrogen and progestogen-containing) hormonal contraception associated with inhibition of ovulation:

   - Oral;
   - Intravaginal;
   - Transdermal;
   - Injectable.

7. Progestogenonly hormone contraception associated with inhibition of ovulation:

   - Oral;
   - Injectable.

8. Sexual abstinence:

090177e1a02cb071\Approved\Approved On: 14-Mar-2024 15:31 (GMT)

- Sexual abstinence is considered a highly effective method only if defined as refraining from heterosexual intercourse during the entire period of risk associated with the study intervention. The reliability of sexual abstinence needs to be evaluated in relation to the duration of the study and the preferred and usual lifestyle of the participant.

One of the following effective barrier methods must be used in addition to the highly effective methods listed above:

- Male or female condom with or without spermicide;
- Cervical cap, diaphragm, or sponge with spermicide;
- A combination of male condom with either cervical cap, diaphragm, or sponge with spermicide (double-barrier methods).

090177e1a02cb071\Approved\Approved On: 14-Mar-2024 15:31 (GMT)

## 10.5. Appendix 5: Genetics

### Use/Analysis of DNA

- Genetic variation may impact a participant's response to study intervention, susceptibility to, and severity and progression of disease. Therefore, where local regulations and IRBs/ethics committees allow, blood and tumor tissue samples will be collected for DNA and/or RNA analysis.

- The scope of the genetic research may be narrow (eg, 1 or more candidate genes) or broad (eg, the entire genome), as appropriate to the scientific question under investigation.

- The samples may be analyzed as part of a multistudy assessment of genetic factors involved in the response to the study interventions or study interventions of this class to understand treatments for the disease(s) under study or the disease(s) themselves.

- The results of genetic analyses may be reported in CSR or in a separate study summary, or may be used for internal decision making without being included in a study report.

- The sponsor will store the DNA samples in a secure storage space with adequate measures to protect confidentiality.

- The samples will be retained as indicated:

    - Samples for biomarker analyses (see Section 8.8) will be stored for up to 10 years following the last participant's last visit for the study or for another period as per local requirements.

    - Samples for banking will be stored indefinitely or for another period as per local requirements.

- Participants may withdraw their consent/assent for the storage and/or use of their Banked Biospecimens at any time by making a request to the investigator; in this case, any remaining material will be destroyed. Data already generated from the samples will be retained to protect the integrity of existing analyses.

- Banked Biospecimens will be labeled with a code. The key between the code and the participant's personally identifying information (eg, name, address) will be held at the study site and will not be provided to the sample bank.

090177e1a02cb071\Approved\Approved On: 14-Mar-2024 15:31 (GMT)

090177e1a02cb071\Approved\Approved On: 14-Mar-2024 15:31 (GMT)

## 10.6. Appendix 6: Liver Safety: Suggested Actions and Follow-up Assessments

### Potential Cases of Drug-Induced Liver Injury

Humans exposed to a drug who show no sign of liver injury (as determined by elevations in transaminases) are termed “tolerators,” while those who show transient liver injury, but adapt are termed “adaptors.” In some participants, transaminase elevations are a harbinger of a more serious potential outcome. These participants fail to adapt and therefore are "susceptible" to progressive and serious liver injury, commonly referred to as DILI. Participants who experience a transaminase elevation above 3 × ULN should be monitored more frequently to determine if they are an “adaptor” or are “susceptible.”

In the majority of DILI cases, elevations in AST and/or ALT precede TBili elevations (>2 × ULN) by several days or weeks. The increase in TBili typically occurs while AST/ALT is/are still elevated above 3 × ULN (ie, AST/ALT and TBili values will be elevated within the same laboratory sample). In rare instances, by the time TBili elevations are detected, AST/ALT values might have decreased. This occurrence is still regarded as a potential DILI. Therefore, abnormal elevations in either AST OR ALT in addition to TBili that meet the criteria outlined below are considered potential DILI (assessed per Hy’s law criteria) cases and should always be considered important medical events, even before all other possible causes of liver injury have been excluded.

The threshold of laboratory abnormalities for a potential DILI case depends on the participant’s individual baseline values and underlying conditions. Participants who present with the following laboratory abnormalities should be evaluated further as potential DILI (Hy’s law) cases to definitively determine the etiology of the abnormal laboratory values:

- Participants with AST/ALT and TBili baseline values within the normal range who subsequently present with AST OR ALT values ≥3 × ULN AND a TBili value ≥2 × ULN with no evidence of hemolysis and an alkaline phosphatase value <2 × ULN or not available.
- For participants with baseline AST **OR** ALT **OR** TBili values above the ULN, the following threshold values are used in the definition mentioned above, as needed, depending on which values are above the ULN at baseline:
    - Preexisting AST or ALT baseline values above the normal range: AST or ALT values ≥2 times the baseline values AND ≥3 × ULN; or ≥8 × ULN (whichever is smaller).
    - Preexisting values of TBili above the normal range: TBili level increased from baseline value by an amount of ≥ 1 × ULN **or** if the value reaches ≥3 × ULN (whichever is smaller).

Rises in AST/ALT and TBili separated by more than a few weeks should be assessed individually based on clinical judgment; any case where uncertainty remains as to whether it represents a potential Hy's law case should be reviewed with the sponsor.

The participant should return to the investigator site and be evaluated as soon as possible, preferably within 48 hours from awareness of the abnormal results. This evaluation should include laboratory tests, detailed history, and physical assessment. The possibility of hepatic neoplasia (primary or secondary) should be considered.

In addition to repeating measurements of AST and ALT and TBili for suspected cases of Hy's law, additional laboratory tests should include albumin, CK, direct and indirect bilirubin, GGT, PT/INR, total bile acids, and alkaline phosphatase. Consideration should also be given to drawing a separate tube of clotted blood and an anticoagulated tube of blood for further testing, as needed, for further contemporaneous analyses at the time of the recognized initial abnormalities to determine etiology. A detailed history, including relevant information, such as review of ethanol, acetaminophen/paracetamol (either by itself or as a coformulated product in prescription or over-the-counter medications), recreational drug, supplement (herbal) use and consumption, family history, sexual history, travel history, history of contact with a jaundiced person, surgery, blood transfusion, history of liver or allergic disease, and potential occupational exposure to chemicals, should be collected. Further testing for acute hepatitis A, B, C, D, and E infection and liver imaging (eg, biliary tract) and collection of serum samples for acetaminophen/paracetamol drug and/or protein adduct levels may be warranted.

All cases demonstrated on repeat testing as meeting the laboratory criteria of AST/ALT and TBili elevation defined above should be considered potential DILI (Hy's law) cases if no other reason for the LFT abnormalities has yet been found. **Such potential DILI (Hy's law) cases are to be reported as SAEs, irrespective of availability of all the results of the investigations performed to determine etiology of the LFT abnormalities.**

A potential DILI (Hy's law) case becomes a confirmed case only after all results of reasonable investigations have been received and have excluded an alternative etiology.

090177e1a02cb071\Approved\Approved On: 14-Mar-2024 15:31 (GMT)

## 10.7. Appendix 7: ECG Findings of Potential Clinical Concern

**ECG Findings That May Qualify as Adverse Events**

- Marked sinus bradycardia (rate <40 bpm) lasting minutes.
- New PR interval prolongation >280 msec.
- New prolongation of QTcF to >480 msec (absolute) or by >60 msec from baseline.
- New-onset- atrial flutter or fibrillation, with controlled ventricular response rate: ie, rate <120 bpm.
- New-onset type I second-degree (Wenckebach) AV block of >30 seconds' duration.
- Frequent PVCs, triplets, or short intervals (<30 seconds) of consecutive ventricular complexes.

**ECG Findings That May Qualify as Serious Adverse Events**

- QTcF prolongation >500 msec.
- New ST-T changes suggestive of myocardial ischemia.
- New-onset left bundle branch block (QRS >120 msec).
- New-onset right bundle branch block (QRS >120 msec).
- Symptomatic bradycardia.
- Asystole:
  - In awake, symptom-free patients in sinus rhythm, with documented periods of asystole ≥3.0 seconds or any escape rate <40 bpm, or with an escape rhythm that is below the AV node;
  - In awake, symptom-free patients with atrial fibrillation and bradycardia with 1 or more pauses of at least 5 seconds or longer;
  - Atrial flutter or fibrillation, with rapid ventricular response rate: rapid = rate >120 bpm.
- Sustained supraventricular tachycardia (rate >120 bpm) ("sustained" = short duration with relevant symptoms or lasting >1 minute).



090177e1a02cb071\Approved\Approved On: 14-Mar-2024 15:31 (GMT)

| |
|---|
| • Ventricular rhythms >30 seconds' duration, including idioventricular rhythm (heart rate <40 bpm), accelerated idioventricular rhythm (HR 40 bpm to <100 bpm), and monomorphic/polymorphic ventricular tachycardia (HR >100 bpm (such as torsades de pointes)).<br>• Type II second-degree (Mobitz II) AV block.<br>• Complete (third-degree) heart block. |
| **ECG Findings That Qualify as Serious Adverse Events** |
| • Change in pattern suggestive of new myocardial infarction.<br>• Sustained ventricular tachyarrhythmias (>30 seconds' duration).<br>• Second- or third-degree AV block requiring pacemaker placement.<br>• Asystolic pauses requiring pacemaker placement.<br>• Atrial flutter or fibrillation with rapid ventricular response requiring cardioversion.<br>• Ventricular fibrillation/flutter.<br>• At the discretion of the investigator, any arrhythmia classified as an adverse experience. |

The enumerated list of major events of potential clinical concern are recommended as "alerts" or notifications from the core ECG laboratory to the investigator and Pfizer study team, and not to be considered as all inclusive of what to be reported as AEs/SAEs.

090177e1a02cb071\Approved\Approved On: 14-Mar-2024 15:31 (GMT)

## 10.8. Appendix 8: Country-Specific Requirements

### 10.8.1. France Contract Unique

#### 1. GCP Training

Before enrolling any participants, the investigator and any subinvestigators will complete the Pfizer-provided Good Clinical Practice training course ("Pfizer GCP Training") or training deemed equivalent by Pfizer. Any investigators who later join the study will do the same before performing study-related duties. For studies of applicable duration, the investigator and subinvestigators will complete Pfizer GCP Training or equivalent every 3 years during the term of the study, or more often if there are significant changes to the ICH GCP guidelines or course materials.

#### 2. Study Intervention

No participants or third-party payers will be charged for study intervention.

#### 3. Urgent Safety Measures

In addition, the investigator will inform Pfizer immediately of any urgent safety measures taken by the investigator to protect the study participants against any immediate hazard, and of any serious breaches of this protocol or of ICH GCP that the investigator becomes aware of.

#### 4. Termination Rights

Pfizer retains the right to discontinue development of encorafenib at any time.

### 10.8.2. Mainland China Specific Procedures

In mainland China, central laboratory results should be used to confirm participant eligibility, however, local laboratory results will be acceptable throughout the remainder of the study for clinical safety laboratory assessments and captured in the eCRF.

090177e1a02cb071\Approved\Approved On: 14-Mar-2024 15:31 (GMT)

## 10.9. Appendix 9: RECIST 1.1

### CATEGORIZING LESIONS AT BASELINE

Measurable Lesions

Lesions that can be accurately measured in at least one dimension.

- Lesions with longest diameter twice the slice thickness and at least 10 mm or greater when assessed by computed tomography or MRI (slice thickness 5-8 mm).
- Lesions with longest diameter at least 20 mm when assessed by chest X-ray.
- Superficial lesions with longest diameter 10 mm or greater when assessed by caliper.
- Malignant lymph nodes with the short axis 15 mm or greater when assessed by computed tomography.

**NOTE: The shortest axis is used as the diameter for malignant lymph nodes, longest axis for all other measurable lesions.**

Non-measurable disease

Non-measurable disease includes lesions too small to be considered measurable (including nodes with short axis between 10 and <15 mm) and truly non-measurable disease such as pleural or pericardial effusions, ascites, inflammatory breast disease, leptomeningeal disease, lymphangitic involvement of skin or lung, clinical lesions that cannot be accurately measured with calipers, abdominal masses identified by physical exam that are not measurable by reproducible imaging techniques.

- Bone disease: Bone disease is non-measurable with the exception of soft tissue components that can be evaluated by computed tomography or MRI and meet the definition of measurability at baseline.
- Previous local treatment: A previously irradiated lesion (or lesion subjected to other local treatment) is non-measurable unless it has progressed since completion of treatment.

Normal sites

- Cystic lesions: Simple cysts should not be considered as malignant lesions and should not be recorded either as target or non-target disease. Cystic lesions thought to represent cystic metastases can be measurable lesions, if they meet the specific definition above. If non-cystic lesions are also present, these are preferred as target lesions.
- Normal nodes: Nodes with short axis <10 mm are considered normal and should not be recorded or followed either as measurable or non-measurable disease.

090177e1a02cb071\Approved\Approved On: 14-Mar-2024 15:31 (GMT)

## RECORDING TUMOR ASSESSMENTS

All sites of disease must be assessed at baseline. Baseline assessments should be done as close as possible prior to study start. For an adequate baseline assessment, all required scans should be done within 28 days prior to treatment and all disease must be documented appropriately. If baseline assessment is inadequate, subsequent statuses generally should be indeterminate.

Target lesions

- All measurable lesions up to a maximum of 2 lesions per organ, 5 lesions in total, representative of all involved organs, should be identified as target lesions at baseline. Target lesions should be selected on the basis of size (longest lesions) and suitability for accurate repeated measurements. Record the longest diameter for each lesion, except in the case of pathological lymph nodes for which the short axis should be recorded. The sum of the diameters (longest for non-nodal lesions, short axis for nodal lesions) for all target lesions at baseline will be the basis for comparison to assessments performed post-baseline.
- If two target lesions coalesce the measurement of the coalesced mass is used. If a large target lesion splits, the sum of the parts is used.
- Measurements for target lesions that become small should continue to be recorded. If the lesion is considered to have disappeared, 0 mm should be recorded; otherwise if a lesion is determined to be present but too small to measure, the lesion status will indicate "too small to measure and judged to be less than 10 mm" and 5 mm will be used in the calculation of the sum of the diameters.

**NOTE: When nodal lesions decrease to <10 mm (normal), the actual measurement should still be recorded.**

Non-target disease

- All non-measurable disease is non-target. All measurable lesions not identified as target lesions are also included as non-target disease. Measurements are not required but rather assessments will be expressed as ABSENT, INDETERMINATE, PRESENT/NOT INCREASED, INCREASED. Multiple non-target lesions in one organ may be recorded as a single item on the case report form (eg, 'multiple enlarged pelvic lymph nodes' or 'multiple liver metastases').

## OBJECTIVE RESPONSE STATUS AT EACH EVALUATION.

Disease sites must be assessed using the same technique as baseline, including consistent administration of contrast and timing of scanning. If a change needs to be made the case must be discussed with the Investigator or radiologist and the sponsor to determine if substitution is possible. If not, subsequent objective statuses are not evaluable.



090177e1a02cb071\Approved\Approved On: 14-Mar-2024 15:31 (GMT)

Target disease

- Complete Response (CR): Complete disappearance of all target lesions with the exception of nodal disease. All target nodes must decrease to normal size (short axis <10 mm). All target lesions must be assessed.
- Partial Response (PR): Greater than or equal to 30% decrease under baseline of the sum of diameters of all target measurable lesions. All target lesions must be assessed.
- Stable Disease (SD): Does not qualify for CR, PR or Progression. All target lesions must be assessed. Stable can follow PR only in the rare case that the sum increases by less than 20% from the nadir (smallest sum of diameters; consider baseline and all assessments prior to the time point under evaluation), but enough that a previously documented 30% decrease no longer holds.
- Objective Progression (PD): 20% increase in the sum of diameters of target measurable lesions above the smallest sum observed (over baseline if no decrease in the sum is observed during therapy), with a minimum absolute increase of 5 mm.
- Not evaluable (NE): Progression has not been documented, and
    - one or more target lesions have not been assessed or
    - assessment methods used were inconsistent with those used at baseline or
    - one or more target lesions cannot be measured accurately (eg, poorly visible unless due to being too small to measure) or
    - one or more target lesions were excised or irradiated and have not reappeared or increased.

Non-target disease

- CR: Disappearance of all non-target lesions and normalization of tumor marker levels (if being followed). All lymph nodes must be 'normal' in size (<10 mm short axis).
- Non-CR/Non-PD: Persistence of any non-target lesions and/or tumor marker level (if being followed) above the normal limits.
- PD: Unequivocal progression of pre-existing lesions. Generally the overall tumor burden must increase sufficiently to merit discontinuation of therapy. In the presence of SD or PR in target disease, progression due to unequivocal increase in non-target disease should be rare.
- Not evaluable (NE): Progression has not been determined and
    - one or more non-target lesion sites have not been assessed or
    - assessment methods used were inconsistent with those used at baseline or
    - one or more non-target lesions cannot be assessed (eg, poorly visible or unclear images) or

090177e1a02cb071\Approved\Approved On: 14-Mar-2024 15:31 (GMT)

- o one or more non-target lesions were excised or irradiated and have not reappeared or increased.

New Lesions

The appearance of any new unequivocal malignant lesion indicates PD. If a new lesion is equivocal, for example due to its small size, continued assessment will clarify the etiology. If repeat assessments confirm the lesion, then progression should be recorded on the date of the initial assessment. A lesion identified in an area not previously scanned will be considered a new lesion.

Supplemental Investigations

- If CR determination depends on a residual lesion that decreased in size but did not disappear completely, it is recommended the residual lesion be investigated with biopsy or fine needle aspirate. If no disease is identified, objective status is CR.
- If progression determination depends on a lesion with an increase possibly due to necrosis, the lesion may be investigated with biopsy or fine needle aspirate to clarify status.

Subjective progression

Participants requiring discontinuation of treatment without objective evidence of disease progression should not be reported as PD on tumor assessment CRFs. This should be indicated on the end of treatment CRF as off treatment due to Global Deterioration of Health Status. Every effort should be made to document PD even after discontinuation of study treatment.

Determination of Tumor Response by RECIST

When both target and non-target lesions are present, individual assessments will be recorded separately. New lesions will also be recorded separately. Determination of tumor response at each assessment based on target, non-target and new lesions is summarized below:

**Table 36. Objective Response Status at Each Assessment for Participants With Measurable Disease at Baseline**

| Target Lesions | Non-target Lesions | New Lesions | Objective status |
|---|---|---|---|
| CR | CR | No | CR |
| CR | Non-CR/Non-PD or not evaluated | No | PR |
| PR | Non-PD* or not all evaluated | No | PR |
| SD | Non-PD* or not all evaluated | No | SD |
| Not all evaluated | Non-PD | No | NE |
| PD | Any | Yes or No | PD |
| Any | PD | Yes or No | PD |
| Any | Any | Yes** | PD |

* Non-PD includes CR and Non-CR/Non-PD
** New lesions must be unequivocal



090177e1a02cb071\Approved\Approved On: 14-Mar-2024 15:31 (GMT)

090177e1a02cb071\Approved\Approved On: 14-Mar-2024 15:31 (GMT)

## 10.10. Appendix 10: Patient Global Impression of Severity and Patient Global Impression of Change

### Patient Global Impression of Severity

Please choose the response below that best describes the severity of your colorectal cancer over the past 7 days.

Select only ONE response.

- None
- Mild
- Moderate

Severe

### Patient Global Impression of Change

Please choose the response below that best describes the overall change in your colorectal cancer since you started taking the study medication.

Select only ONE response.

- Much better
- A little better
- No change
- A little worse
- Much worse

090177e1a02cb071\Approved\Approved On: 14-Mar-2024 15:31 (GMT)

## 10.11. Appendix 11: Abbreviations

The following is a list of abbreviations that may be used in the protocol.

| Abbreviation | Term |
|---|---|
| 5-FU | fluorouracil |
| Abs | absolute |
| ACE | angiotensin-converting enzyme |
| ADR | adverse drug reaction |
| AE | adverse event |
| ALT | alanine aminotransferase |
| ANC | absolute neutrophil count |
| aPTT | activated partial thromboplastin time |
| Arm C | Control Arm |
| ASCO | American Society of Clinical Oncology |
| AST | aspartate aminotransferase |
| ATP | adenosine triphosphate |
| AUC | area under the concentration-time curve |
| AV | atrioventricular |
| AxMP | auxiliary medicinal product |
| BBB | bundle-branch block |
| BCRP | breast cancer-resistance protein |
| β-hCG | beta-human chorionic gonadotropin |
| BICR | blinded independent central review |
| BID | twice a day |
| BOR | best overall response |
| Bpm | beats per minute |
| *BRAF* | B-Raf proto-oncogene |
| BRAF | serine/threonine-protein kinase B-Raf |
| BSA | body surface area |
| BUN | blood urea nitrogen |
| CAP | College of American Pathologists |
| CAPOX | capecitabine/oxaliplatin |
| CD4 | cluster of differentiation 4 |
| CDx | companion diagnostic |
| CE | Conformité Européene |
| CEA | carcinoembryonic antigen |
| cfDNA | circulating free DNA |
| CFR | Code of Federal Regulations |
| CHMP | Committee for Medicinal Products for Human Use |
| CI | confidence interval |
| CIOMS | Council for International Organizations of Medical Sciences |
| CK | creatine kinase |
| CLIA | Clinical Laboratory Improvement Amendments |



090177e1a02cb071\Approved\Approved On: 14-Mar-2024 15:31 (GMT)

| Abbreviation | Term |
|---|---|
| $C_{max}$ | maximum concentration |
| $C_{min}$ | minimum concentration |
| CNS | central nervous system |
| CO2 | carbon dioxide (bicarbonate) |
| CONSORT | Consolidated Standards of Reporting Trials |
| COVID-19 | coronavirus disease 2019 |
| CR | complete response |
| CRC | colorectal cancer |
| CRF | case report form |
| CRO | contract research organization |
| CRP | C-reactive protein |
| CSR | Clinical Study Report |
| CT | clinical trial; computed tomography |
| CTCAE | Common Terminology Criteria for Adverse Events |
| ctDNA | circulating tumor DNA |
| CYP | cytochrome P450 enzyme (1A2, 2B6, 2C8, 2C9, 2C19 and 3A4 refer to isoforms) |
| DDI | drug-drug interaction |
| DDR | DNA damage response and repair |
| DILI | drug-induced liver injury |
| DLT | dose-limiting toxicity |
| dMMR | deficient mismatch repair |
| DOR | duration of response |
| DPD | dihydropyrimidine dehydrogenase |
| DRE | disease-related event |
| EC | encorafenib plus cetuximab |
| ECG | electrocardiogram |
| ECOG PS | Eastern Cooperative Oncology Group performance status |
| eCRF | electronic case report form |
| E-DMC | External Data Monitoring Committee |
| EDP | exposure during pregnancy |
| EGFR | epidermal growth factor receptor |
| EMA | European Medicines Agency |
| EORTC QLQ-C30 | European Organisation for Research and Treatment of Cancer Quality of Life Questionnaire for Cancer Patients – 30 Item Core Questionnaire |
| EOT | end of treatment |
| EQ-5D-5L | EuroQol-5D-5L |
| EQ VAS | EuroQol-visual analogue scale |
| ERK | extracellular signal-regulated kinase |
| ESMO | European Society for Medical Oncology |
| EU | European Union |
| EudraCT | European Clinical Trials Database |

090177e1a02cb071\Approved\Approved On: 14-Mar-2024 15:31 (GMT)

| Abbreviation | Term |
|---|---|
| FAS | full analyses set |
| FDA | Food and Drug Administration |
| FDG-PET | fluorodeoxyglucose-positron emission tomography |
| FFPE | formalin-fixed paraffin-embedded |
| FOLFIRI | fluorouracil/leucovorin/irinotecan |
| FOLFOX | fluorouracil/leucovorin/oxaliplatin |
| FOLFOXIRI | fluorouracil/leucovorin/oxaliplatin/irinotecan |
| FSH | follicle-stimulating hormone |
| G-CSF | granulocyte colony-stimulating factor |
| GCP | Good Clinical Practice |
| GGT | gamma-glutamyl transferase |
| GI | gastrointestinal |
| GM-CSF | granulocyte-macrophage colony stimulating factor |
| HA | health authority |
| HBcAb | hepatitis B core antibody |
| HBsAg | hepatitis B surface antigen |
| HBV | hepatitis B virus |
| HCV | hepatitis C virus |
| HFSR | hand foot skin reaction |
| HGRAC | Human Genetics Resources Administration of China |
| HIPAA | Health Insurance Portability and Accountability Act |
| HIV | human immunodeficiency virus |
| HR | hazard ratio; heart rate |
| HRT | hormone replacement therapy |
| IA | Interim Analysis |
| IB | Investigator’s Brochure |
| ICD | informed consent/assent document |
| ICH | International Council for Harmonisation |
| ID | identification |
| IEC | Independent Ethics Committee |
| IMP | investigational medicinal product |
| IND | Investigational New Drug |
| INR | international normalized ratio |
| IP manual | investigational product manual |
| IPAL | Investigational Product Accountability Log |
| IRB | Institutional Review Board |
| IRT | Interactive Response Technology |
| IV | intravenous |
| JSMO | Japanese Society of Medical Oncology |
| KA | keratoacanthoma |
| *KRAS* | KRAS proto-oncogene |
| LDH | lactate dehydrogenase |



090177e1a02cb071\Approved\Approved On: 14-Mar-2024 15:31 (GMT)

| Abbreviation | Term |
|---|---|
| LFT | liver function test |
| LHY746 | encorafenib metabolite |
| LPLV | last participant last visit |
| LQTS | long QT syndrome |
| LV | left ventricular |
| mCRC | metastatic colorectal cancer |
| MDRD | modification of diet in renal disease |
| MedDRA | Medical Dictionary for Regulatory Activities |
| MEK | mitogen-activated protein kinase kinase |
| mFOLFOX6 | modified fluorouracil/leucovorin/oxaliplatin |
| MHRA | Medicines & Healthcare products Regulatory Agency |
| MMR | mismatch repair |
| mOS | median overall survival |
| mPFS | median progression-free survival |
| MQI | medically qualified individual |
| MRI | magnetic resonance imaging |
| MSI | microsatellite instability |
| MSI-H | microsatellite instability-high |
| MSS | microsatellite stable |
| MTD | maximum tolerated dose |
| n, N | number |
| NaF-PET | sodium fluoride-positron emission tomography |
| NCCN | National Comprehensive Cancer Network |
| NCI | National Cancer Institute |
| NCIC | National Cancer Information Center |
| NE | not evaluable |
| NGS | next generation sequencing |
| NIMP | noninvestigational medicinal product |
| NK1 | neurokinin-1 |
| NR | not reached |
| NRAS | NRAS Proto-Oncogene, GTPase |
| NTI | narrow therapeutic index |
| NYHA | New York Heart Association |
| OAT | organic anion transporter (1 and 3 refer to isoforms) |
| OATP | organic anion transporting polypeptide (1B1 and 1B3 refer to isoforms) |
| OCT | organic cation transporter (2 refers to isoform) |
| OR | objective response |
| ORR | objective response rate |
| OS | overall survival |
| PCR | polymerase chain reaction |
| PD | progressive disease |
| PD-L1 | programmed death-ligand 1 |



090177e1a02cb071\Approved\Approved On: 14-Mar-2024 15:31 (GMT)

| Abbreviation | Term |
|---|---|
| PFS | progression-free survival |
| PFS2 | progression after next line of therapy |
| PGIC | Patient Global Impression of Change |
| PGIS | Patient Global Impression of Severity |
| P-gp | P-glycoprotein |
| PICC | peripherally inserted central catheter |
| PK | pharmacokinetic(s) |
| pMMR | proficient mismatch repair |
| PO | oral |
| PR | partial response |
| PRES | posterior reversible encephalopathy syndrome |
| PRO | patient reported outcome |
| PT | preferred term; prothrombin time |
| PTT | partial thromboplastin time |
| PVC | premature ventricular contraction/complex |
| Q2W | every 2 weeks |
| Q6W | every 6 weeks |
| Q8W | every 8 weeks |
| QALY | quality-adjusted life-year |
| QD | once daily |
| QoL | quality of life |
| QT | QT interval |
| QTc | corrected QT interval |
| QTcF | QTc corrected using Fridericia's formula |
| *RAS* | RAS family of proto-oncogenes |
| RAS | RAS GTPase |
| *RAF* | RAF family of proto-oncogenes |
| RAF | RAF GTPase |
| RBC | red blood cell |
| RECIST | Response Evaluation Criteria in Solid Tumors |
| RGQ | Rotor-Gene Q |
| RP2D | recommended Phase 2 dose |
| RPLS | reversible posterior leukoencephalopathy syndrome |
| SAE | serious adverse event |
| SAP | Statistical Analysis Plan |
| SARS-CoV-2 | severe acute respiratory syndrome coronavirus 2 |
| SC | steering committee |
| SCC | squamous cell carcinoma |
| SD | standard deviation; stable disease |
| SEER | surveillance, epidemiology, and end results |
| SLI | Safety Lead-in |
| SmPC | Summary of Product Characteristics |



090177e1a02cb071\Approved\Approved On: 14-Mar-2024 15:31 (GMT)

| Abbreviation | Term |
|---|---|
| SN-38 | active metabolite of irinotecan |
| SN-38G | inactive metabolite of irinotecan |
| SoA | schedule of activities |
| SOC | standard of care |
| SOP | standard operating procedure |
| SRSD | single reference safety document |
| SUSAR | suspected unexpected serious adverse reaction |
| T | temperature |
| TBili | total bilirubin |
| TdP | Torsades de Pointes |
| TKI | tyrosine kinase inhibitor |
| TME | tumor microenvironment |
| TOC | table of contents |
| TTR | time to response |
| UGT | uridine 5’-diphospho-glucuronosyltransferase (1A1 refers to isoform) |
| UK | United Kingdom |
| ULN | upper limit of normal |
| US | United States |
| USA | United States of America |
| USPI | United States Prescribing Information |
| VAF | variant allele fraction |
| VEGF | vascular endothelial growth factor |
| WBC | white blood cell |
| WHO | World Health Organization |
| WOCBP | woman of childbearing potential |
| wt | wild type |

090177e1a02cb071\Approved\Approved On: 14-Mar-2024 15:31 (GMT)

## 10.12. Appendix 12: Alternative Measures During Public Emergencies

The alternative study measures described in this section are to be followed during public emergencies, including the COVID-19 pandemic globally, if allowed by local regulation. This appendix applies for the duration of the COVID-19 pandemic and will become effective for other public emergencies only upon written notification from Pfizer.

Use of these alternative study measures are expected to cease upon the return of business as usual circumstances (including the lifting of any quarantines and travel bans/advisories).

### 10.12.1. Eligibility

While SARS-CoV-2 testing is not mandated for this study, local clinical practice standards for testing should be followed. A participant should be excluded if he/she has a positive test result for SARS-CoV-2 infection, is known to have asymptomatic infection, or is suspected of having SARS-CoV-2. Participants with active infections are excluded from study participation as per exclusion criterion 13. When the infection resolves, the participant may be considered for re-screening.

### 10.12.2. Telehealth Visits

In the event that in-clinic study visits cannot be conducted, every effort should be made to follow up on the safety of study participants at scheduled visits per the Schedule of Activities (Section 1.3) or unscheduled visits. Telehealth visits may be used to continue to assess participant safety and collect data points. Telehealth includes the exchange of healthcare information and services via telecommunication technologies (e.g., audio, video, video-conferencing software) remotely, allowing the participant and the investigator to communicate on aspects of clinical care, including medical advice, reminders, education, and safety monitoring. The following assessments must be performed during a telehealth visit:

- Review and record study intervention(s), including compliance and missed doses.
- Review and record any AEs and SAEs since the last contact. Refer to Section 8.3.
- Review and record any new concomitant medications or changes in concomitant medications since the last contact.
- Review and record contraceptive method and results of pregnancy testing. Confirm that the participant is adhering to the contraception method(s) required in the protocol. Refer to Section 8.2.9, Appendix 4 (Section 10.4) and Section 10.12.3 of this appendix regarding pregnancy tests.

Study participants must be reminded to promptly notify site staff about any change in their health status.

### 10.12.3. Alternative Facilities for Safety Assessments

#### 10.12.3.1. Laboratory Testing

If a study participant is unable to visit the site for protocol-specified safety laboratory evaluations, testing may be conducted at a local laboratory if permitted by local regulations. The local laboratory may be a standalone institution or within a hospital. Refer to



090177e1a02cb071\Approved\Approved On: 14-Mar-2024 15:31 (GMT)

Section 8.2.8 Clinical Safety Laboratory Assessments and Section 10.12.3, for the list of safety laboratory evaluations, including pregnancy testing required per protocol.

If a local laboratory is used, qualified study site personnel must order, receive, and review results. Site staff must collect the local laboratory reference ranges and certifications/ accreditations for filing at the site. Laboratory test results are to be provided to the site staff as soon as possible. The local laboratory reports should be filed in the participant's source documents/medical records. Relevant data from the local laboratory report should be recorded on the CRF.

If a participant cannot visit a local laboratory for pregnancy testing, a home urine pregnancy testing kit with a sensitivity of at least 25 IU/mL may be used by the participant to perform the test at home, if compliant with local regulatory requirements. The pregnancy test outcome should be documented in the participant's source documents/medical records and relevant data recorded on the CRF. Confirm that the participant is adhering to the contraception method(s) required in this protocol.

#### 10.12.3.2. Imaging

If the participant is unable to visit the study site for safety imaging assessments, the participant may visit an alternative facility to have the safety imaging assessment(s) performed. Qualified study site personnel must order, receive, and review results.

#### 10.12.3.3. Electrocardiograms

If the participant is unable to visit the study site for ECGs, the participant may visit an alternative facility to have the ECGs performed. Qualified study site personnel must order, receive, and review results.

### 10.12.4. Study Intervention

If the safety of a trial participant is at risk because they cannot complete required evaluations or adhere to critical mitigation steps, then discontinuing that participant from study intervention must be considered.

Study intervention may be shipped by courier to study participants if permitted by local regulations and in accordance with storage and transportation requirements for the study intervention. Pfizer does not permit the shipment of study intervention by mail. The tracking record of shipments and the chain of custody of study intervention must be kept in the participant's source documents/medical records.

The following is recommended for the administration of study intervention for participants who have active confirmed (positive by regulatory authority-approved test) or presumed (test pending/clinical suspicion) SARS-CoV-2 infection:

- For symptomatic participants with active SARS-CoV-2 infection, study intervention should be delayed for at least 14 days from the start of symptoms. This delay is intended to allow the resolution of symptoms of SARS-CoV-2 infection.



090177e1a02cb071\Approved\Approved On: 14-Mar-2024 15:31 (GMT)

- Prior to restarting treatment, the participant should be afebrile for 72 hours, and SARS-CoV-2-related symptoms should have recovered to ≤ Grade 1 for a minimum of 72 hours. Notify the study team when treatment is restarted.
- Continue to consider potential drug-drug interactions as described in Section 6.5 for any concomitant medication administered for treatment of SARS-CoV-2 infection.

### 10.12.5. Home Health Visits

A home health care service may be utilized to facilitate scheduled visits per the Schedule of Activities. Home health visits include a healthcare provider conducting an in-person study visit at the participant's location, rather than an in-person study visit at the site. The following may be performed during a home health visit:

- Physical exam including dermatological lesions and vital signs
- Review and record study treatment(s), including compliance and missed doses.
- Review and record any AEs and SAEs since the last contact. Refer to Section 8.3.
- Review and record any new concomitant medications or changes in concomitant medications since the last contact.
- Review and record contraceptive method and results of pregnancy testing. Confirm that the participant is adhering to the contraception method(s) required in the protocol. Refer to Section 8.2.9 and Section 10.12.3 of this appendix regarding pregnancy tests.

### 10.12.6. Adverse Events and Serious Adverse Events

If a participant has COVID-19 during the study, this should be reported as an adverse event (AE) or serious adverse event (SAE) and appropriate medical intervention provided. Study treatment should continue unless the investigator/treating physician is concerned about the safety of the participant, in which case temporary or permanent discontinuation may be required.

It is recommended that the Investigator discuss temporary or permanent discontinuation of study intervention with the Pfizer medical monitor.

### 10.12.7. Efficacy Assessments

If the participant is unable to visit the study site for imaging assessments (e.g., CT, MRI, X-ray, FDG-PET), the participant may visit an alternative facility to have the imaging assessments performed. Qualified study site personnel must order, receive, and review results.

### 10.12.8. Independent Oversight Committees

There will be no impact on the Steering Committee Charter or E-DMC Charter. The Steering Committee and E-DMC will continue to be consulted during public health emergencies.



090177e1a02cb071\Approved\Approved On: 14-Mar-2024 15:31 (GMT)

## 10.13. Appendix 13: Protocol Amendment History

The Protocol Amendment Summary of Changes Table for the current amendment is located directly before the table of contents (TOC). The protocol amendment summary of changes tables for past amendment(s) can be found below:

| | | |
|---|---|---|
| Amendment 5 | 20 December 2022 | 1. Overall Protocol: Study design revision: Enrollment in Arm A was discontinued.<br>2. Section 1.1 Synopsis:<br>• Revised rationale text to include Cohort 3 Arms D and E.<br>• Revised Estimand text to include the Primary Estimand for Cohort 3.<br>• Revised Overall Design text to include Cohort 3 design and primary objective.<br>• Decreased the number of Phase 3 participants from 870 to 620: 150 participants in Arm A (enrollment discontinued), 235 each in Arm B and in Arm C. Added text for Cohort 3.<br>• Revised Intervention Groups and Duration text to include Arms D and E in Cohort 3.<br>3. Section 1.1 Objectives, Estimands, and Endpoints:<br>• Added ORR as primary objective and endpoint.<br>• Removed reference to Arm A as key secondary objective and PFS as endpoint.<br>• Added OS as secondary objective and ORR as secondary endpoint for Arm A vs Control Arm and Arm B.<br>• Added objectives and endpoints (ORR, DOR, PFS, TTR, & OS) for Cohort 3.<br>• Added Tertiary/Exploratory objectives/endpoints.<br>4. Section 1.1 Estimands: Added discussion of OR and ORR for Arm B and Control Arm.<br>5. Section 1.1 Overall Design: Added text about FOLFIRI and mFOLFOX6 for use in Cohort 3.<br>6. Section 1.1 Number of Participants: Revised number of participants for Phase 3 and added number of participants for Cohort 3.<br>7. Section 1.1 Intervention Groups and Duration: Added information for Cohort 3.<br>8. Section 1.1 Data Monitoring Committee or Other Independent Oversight Committee: Removed study phase specification in the paragraph that describes imaging.<br>9. Section 1.2 Schema: Revised study design figure to show revision of Phase 3 study design and to include Cohort 3.<br>10. Section 1.3 Schedule of Activities: Clarified +3 day window applies to a study visit.<br>Revised Table 3 to reflect the addition of Cohort 3 Arm D:<br>• Specified Cetuximab infusion is for Arm A, Arm B, and Cohort 3 Arm D Only and FOLFIRI is only applicable to Cohort 3 Arm D.<br>• Removed optional Bevacizumab dosing for Cohort Arm E.<br>• Removed Arm and Cohort specific details from tumor radiographic assessment (CT, MRI) timing.<br>Revised Table 4. To reflect the addition of Cohort 3 Arm E: |

090177e1a02cb071\Approved\Approved On: 14-Mar-2024 15:31 (GMT)

| | | |
|---|---|---|
| | | • Specified mFOLFOX6 and FOLFOXIRI are Arm C only and FOLFIRI is Arm E only.<br>11. Section 2.3.1 Risk Assessment: Removed Potential Risk of Drug-Drug Interaction section for over-exposure of irinotecan/SN-38 due to CYP3A4 inhibition by encorafenib. Rationale expanded in 4.2.2.<br>12. Section 3 Objectives, Estimands, and Endpoints: Changes as detailed above in Section 1.1.<br>13. Section 4.1 Overall Design:<br>• Revised text to include Cohort 3 (Arms D and E).<br>• Decreased the number of Phase 3 participants from 870 to 620: 150 participants in Arm A (enrollment discontinued), 235 each in Arm B and in Arm C. Added text for Cohort 3.<br>14. Section 4.2.2 Assessment of Potential DDI with Encorafenib:<br>• Revised text to include in vitro studies indicating encorafenib is reversible inhibitor of some CYP450 enzymes and UGT1A1.<br>• Added text on observed reduction in irinotecan exposure when co-administered with encorafenib at steady state.<br>• Added text that encorafenib does not inhibit CYP3A on Day 1 at clinically relevant doses.<br>15. Section 4.2.4 Progression-Free Survival and Objective Response Rate as Primary Endpoints: Section modified to account for addition of ORR as primary endpoint for Phase 3 and addition of Cohort 3 endpoints.<br>16. Section 4.4 End of Study Definition: Revised definition to be based on LSLV, not on number of events.<br>17. Section 5.1.1 Molecular Prescreening Inclusion Criteria:<br>• Included Cohort 3 in criterion #1<br>• Replaced text regarding the central laboratory requirement for BRAF V600E confirmation from criterion # 10 with a reference to 8.7.2 for further clarification.<br>• Added text specifying a minimum of 10 tumor sample slides for mainland China is inserted for criterion #5.<br>18. Section 5.1.2 Screening Inclusion Criteria:<br>• Added additional text to patients with oligometastatic colorectal cancer.<br>• Added text concerning prior systemic regimens and measurable disease for Cohort 3.<br>• Added text specifying a minimum of 10 tumor sample slides for mainland China is inserted for criterion #10.<br>19. Section 5.2.1 Molecular Prescreening Exclusion Criteria: Added text on Gilbert's syndrome for Cohort 3.<br>20. Section 5.2.1 Medical Conditions Exclusion Criteria: Updated HBV DNA test result parameter for one of the active HBV definitions.<br>21. Section 5.2.1 Prior/Concomitant Therapy Exclusion Criteria: Updated major surgery criteria.<br>22. Section 5.3.1 Contraception: Added text requiring the use of hormonal agents when combined with other highly effective methods for patients taking encorafenib.<br>23. Section 5.3.2 Meals and Dietary Restrictions: Added mention for Cohort 3. |



090177e1a02cb071\Approved\Approved On: 14-Mar-2024 15:31 (GMT)

| | | |
|---|---|---|
| | | 24. Section 5.3.3 Photosensitivity: Updated wording in alignment with encorafenib and 5-FU SRSDs.<br>25. Section 6.1 Study Interventions Administered:<br>• Added Cohort 3 where applicable in column headers.<br>• Updated footnote a regarding different presentations of leucovorin.<br>26. Section 6.1.2 Treatment Regimens: Added Tables 14 and 15 for Cohort 3 treatment regimens.<br>27. Section 6.1.3.1 Administration of Encorafenib:<br>• Revised text to include mention of Cohort 3.<br>• Removed text relating to DDI of encorafenib and irinotecan in SLI.<br>28. Section 6.3.1 Allocation to Study Intervention: Added information for Cohort 3.<br>29. Section 6.5.1.2 CYP and UGT Substrates and Inhibitors: Added information regarding CYP3A based on recent data from ongoing DDI study.<br>30. Section 6.5.1.3 Transporter Substrates and Inhibitors: Added text on dose reductions of drugs used concomitantly with encorafenib.<br>31. Section 6.5.2 Prohibited Concomitant Therapy: Irinotecan was added to the last 2 bullet points in the section.<br>32. Section 6.5.3.1 Premedication for Cetuximab Administration: Added Cohort 3 Arm D.<br>33. Section 6.6: Dose Modification: Added mention of Cohort 3.<br>34. Section 6.6.2 Dose Modification for Cetuximab: Added information for Cohort 3 as well as re-worded this section for clarity.<br>35. Section 6.6.3 Dose Modification for Oxaliplatin, Irinotecan, 5-FU and Capecitabine as Part of mFOLFOX6, FOLFIRI, FOLFOXIRI and CAPOX Regimens:<br>• Added information for Cohort 3.<br>• Combined Table 18 and 19 (amendment 4) into 1 table (Table 20 in amendment 5).<br>36. Section 6.7 Intervention After the End of the Study: Clarified the plan for intervention after the end of study.<br>37. Section 7.1 Discontinuation of Study Intervention: Added mention of Cohort 3.<br>38. Section 8 Study Assessments and Procedures: Included Cohort 3.<br>39. Section 8.1.3 Survival Follow-up: Section added to clarify procedures and duration of participant follow-up for OS.<br>40. Section 8.2.7 Electrocardiogram: Removed text regarding automatic QTcF and updated language about QTcF calculation.<br>41. Section 8.5 Pharmacokinetics:<br>• Added mention of PK sampling in Cohort 3.<br>• Added PK sampling in Arm B for mainland China.<br>42. Section 8.7.2 BRAF Testing: Revised language on discordance test results.<br>43. Section 8.7.3 (Molecular Prescreening): Removed text specifying only 10 tumor sample slides for mainland China.<br>44. Section 9.1.1 Estimands: Added information on primary endpoint of OR in Phase 3 (Arm B and Control Arm) and Cohort 3.<br>45. Section 9.1.2 Statistical Hypotheses: Revised text in Cohort 3 to include Arms D and E. |



090177e1a02cb071\Approved\Approved On: 14-Mar-2024 15:31 (GMT)

| | | |
|---|---|---|
| | | 46. Section 9.2 Sample Size Determination: Revised sample size for Arm B, Control Arm, and Cohort 3.<br>47. Section 9.3 Analysis Sets: Revised the FAS description and included Cohort 3 in the SAS description.<br>48. Section 9.4.1 General Considerations: Included Cohort 3.<br>49. Section 9.4.1.1 Statistical Testing Strategy:<br>• Revised information for Phase 3 Arms and added information on analyses of ORR for Cohort 3.<br>• Revised information for statistical testing strategy.<br>50. Section 9.4.1.3 Stratification Factors: Added stratification information for Cohort 3.<br>51. Section 9.4.2 Primary Endpoints: Added information on ORR and its use in Phase 3 and Cohort 3.<br>52. Section 9.4.3 Secondary Endpoints: Added information on OS, ORR, TTR, PFS, and PROs in Cohort 3.<br>53. Section 9.5.1 Interim Efficacy Analysis for OS (Phase 3): Removed interim analysis for PFS.<br>54. Section 9.5.2 Interim Efficacy Analysis for OS: Revised text for analyses planned for OS and removed table for planned efficacy boundaries for OS.<br>55. Throughout Protocol: Editorial revisions and typographical errors corrected.<br>56. REFERENCES: The list of references has been updated accordingly. |
| Amendment 4 | 28 February 2022 | Based on the safety lead-in (SLI) data showing the tolerability of encorafenib and cetuximab (EC)+chemotherapy treatment, the primary purpose of this amendment is to modify the primary and key secondary endpoints, the statistical procedure to preserve overall study alpha and update the sample size to align with the new statistical plan.<br>Additionally, based on review of the totality of the SLI data, Arm B will consist of EC + mFOLFOX6; therefore, references to the use of FOLFIRI in Arm B and Arm C have been removed throughout. Clarifications and other administrative modifications have also been included throughout.<br>1. Section 1 Protocol Summary, Section 3 Objectives, Estimands and Endpoints, Section 4 Study Design, Section 5.2.1 Molecular Prescreening Exclusion Criteria 6b, 6.1 Study Intervention, Section 6.6 Dose Modifications, Section 9.4.3.10 Exposure-Response Relationship of Encorafenib: Removed references to FOLFIRI in Arm B and Arm C of Phase 3<br>2. Section 1.1 Synopsis, Section 4.1 Overall Design: Added paragraph regarding SLI data review and decision that Arm B will consist of EC in combination with mFOLFOX6<br>3. Section 1.1 Synopsis, Section 3 Objectives, Estimands and Endpoints and Section 4.1 Overall Design: Modified to align with the primary and key secondary endpoints, testing strategy and updated sample size<br>4. Section 1.1 Synopsis and Section 3 Objectives, Estimands and Endpoints and Section 8.5 Pharmacokinetics: Clarified, where appropriate, that the Phase 3 PK objective is for mainland China sites<br>5. Section 1.1 Synopsis: Clarified E-DMC review to be done according to the charter |



090177e1a02cb071\Approved\Approved On: 14-Mar-2024 15:31 (GMT)

| | | |
|---|---|---|
| | | 6. Section 1.2 Schema: Updated figure to remove reference to FOLFIRI and updated Phase 3 sample size<br>7. Table 1 Schedule of Activities: Removed the DPD documentation row due to the heterogenicity in DPD testing practice worldwide<br>8. Table 2 Schedule of Activities: Removed BICR from radiological assessment row for consistency with Section 8.1.1<br>9. Table 2, 3 and 4 Schedule of Activities and Section 8.2.3 Physical Examination: Added weight assessment on Day 15 to align with standard practice<br>10. Table 4 Schedule of Activities: Added an X for bevacizumab administration to Day 15 to align with standard practice<br>11. Table 3, 4, 5 Schedule of Activities:<br>• Added clarification on Cycle 1 Day 1 window<br>• Added clarification on new anticancer therapy<br>• Added clarification on when on-treatment biopsies should be collected<br>12. Table 3, 4, 5 Schedule of Activities and Section 8.8 Biomarkers, Section 8.8.3 Circulating Free DNA (cfDNA) and Section 8.8.5 Specified Protein Research: Clarified the collection volume of the cfDNA and protein biomarkers samples for mainland China<br>13. Table 5 Schedule of Activities: Added an X for contraceptive check at EOT Visit for consistency with other SoA tables<br>14. Section 4.4 End of Study Definition: Modified end of study definition to align with revised statistical analysis<br>15. Section 5.1.1 Molecular Prescreening Exclusion Criteria and 5.1.2 Screening Inclusion Criteria: Added clarification in criteria 5 and 10 on sample type, age of specimen and number of slides<br>16. Section 5.1.2 Screening Inclusion Criteria and Section 8.7.2 BRAF Testing: Modified the number of allowed discordant results to align with new sample size<br>17. Section 5.2.1 Molecular Prescreening Exclusion Criteria:<br>• Changed criteria 2 from known history of pancreatitis to presence of pancreatitis for clarification<br>• Modified criteria 5 to clarify that investigators should align with local labels/clinical guidelines and recommendations due to the heterogenicity in DPD testing practice worldwide<br>• Clarified in criteria 9 that unknown MSI/MMR status is also excluded<br>18. Section 5.2.2 Screening Exclusion Criteria:<br>• Changed criteria 11c to Recent history (1 year) for clarification<br>• Added clarification on normal range in criteria 15a<br>• Added clarification regarding general anesthesia to criteria 21<br>19. Section 6.1 Study Interventions Administered:<br>• Added an additional vial size for calcium folinate to footnote a<br>• Modified footnote b to align with Arm B<br>20. Section 6.1.2 Treatment Regimens: Changed “cetuximab” to “bolus and infusion treatment” in the third paragraph for consistency with the regimens in Arm B |



090177e1a02cb071\Approved\Approved On: 14-Mar-2024 15:31 (GMT)

| | | |
|---|---|---|
| | | 21. Section 6.1.3.2 Administration of Capecitabine, Cetuximab, 5-Fluorouracil, Irinotecan, Leucovorin, Oxaliplatin and Bevacizumab: Added cetuximab pretreatment instructions<br>22. Section 6.3.1 Allocation to Study Intervention: Added clarification regarding Phase 3 randomization and stratification<br>23. Section 6.5.2 Prohibited Concomitant Therapy: Added clarification on administration of live vaccines<br>24. Section 6.5.3.5 Hematopoietic Growth Factor: Added GM-CSF examples and clarified when they are allowed<br>25. Section 6.5.3.6 Antiemetics: Added new section to provide clarification on allowed antiemetics<br>26. Section 6.6 Dose Modification:<br>• Clarified recommendations regarding dosing on Day 1 of each cycle<br>• Added dosing guidance in instance of cetuximab infusion reaction<br>27. Section 6.6.1 Dose Modifications for Encorafenib: Added guidance for encorafenib dose re-escalation<br>28. Section 6.6.2 Dose Modifications for Cetuximab: Moved the sentence regarding cetuximab discontinuation instructions to Section 6.6.3<br>29. Section 6.6.3 Dose Modifications for Oxaliplatin, Irinotecan and 5-FU and Capecitabine as Part of mFOLFOX6, FOLFIRI, and FOLFOXIRI and CAPOX Regimens: Modified entire section for clarification and alignment with standard-of-care practice<br>30. Section 7.1 Discontinuation of Study Intervention: Added language regarding treatment beyond progression<br>31. Section 8.1.1 Tumor Response Assessments:<br>• Added clarification on new anticancer therapy<br>• Added radiologist guidance<br>• Clarified BICR timing<br>• Added language regarding treatment beyond progression<br>32. Section 8.1.2 Patient Reported Outcomes: removed paragraphs that were not applicable to the study<br>33. Section 8.2.6 Vital Signs: Added flexibility on sequence of assessment with regards to laboratory tests<br>34. Section 8.3.7. Disease Related Events and/or Disease Related Outcomes Not Qualifying as AEs or SAEs: Added clarification regarding when to collect fatal outcomes<br>35. Section 8.5 Pharmacokinetics: Added clarification to the timing of predose samples to Table 28, Table 29 and Table 30 footnotes<br>36. Section 8.7.3 Molecular Prescreening: Modified language for consistency with Section 5.1.2 and laboratory manual<br>37. Section 8.8.2 Tumor Tissue Assessments: Added language regarding optional on-treatment de novo tumor biopsies<br>38. Section 8.10 Health Economics: Removed language for events not captured in the eCRF<br>39. Section 9 Statistical Considerations: Modified section throughout to align with the primary and key secondary endpoints, testing strategy and updated sample size<br>40. Section 10.1.1 Regulatory and Ethical Considerations: Modified section in response to health authority feedback<br>41. Appendix 2, Table 35 Clinical Laboratory Tests: Added footnote to allow for local urea testing to be performed |



090177e1a02cb071\Approved\Approved On: 14-Mar-2024 15:31 (GMT)

| | | |
|---|---|---|
| | | 42. Appendix 8 Country Specific Requirements: Added Section 10.8.2 for Mainland China Specific Procedures Appendix 9 RECIST 1.1: Added Table 36 clarifying determination of tumor response |
| Amendment 3 | 24 February 2021 | The primary purpose of this amendment is to incorporate HA recommendations. The majority of HA comments were with regards to aligning recommendations (dosing, patient management) with locally approved labels/prescribing information (hereafter referred to as labels). In addition, clarifications and administrative modifications have also been included.<br>1. Section 1.1 Synopsis and Section 3 Objectives, Estimands and Endpoints:<br>• Added clarification that the PFS by BICR and OS Phase 3 Secondary objectives are for Arm A vs Arm B<br>• Removed that PFS2 is per RECIST v1.1<br>2. Section 1.1 Synopsis, Number of Participants and Section 4.1 Overall Design: added clarification that if neither SLI regimen is tolerated, Phase 3 will proceed without Arm B<br>3. Table 1 Schedule of Activities:<br>• The X in the Contact IRT row has been moved from prescreening to the correct screening column<br>• A row for discussion of fertility preservation at screening has been added to align with locally approved product labels as requested by multiple health authorities<br>4. Table 2, 3, 4 and 5 Schedule of Activities:<br>• Added a definition of End of Treatment for clarity<br>• Clarified visit windows<br>• Added windows and combined notes in the tumor radiographic assessment row<br>• Added clarification that PK sampling must occur on the day of dosing<br>• Clarified that blood samples for germline comparator and banked biospecimen only need to be collected at Cycle 1<br>The following changes were made in response to health authority feedback:<br>• Added a contraceptive check row to align with locally approved product labels<br>• Added that physical exam should include a visual assessment to align with encorafenib approved label<br>• Added a urine pregnancy test at 28-day follow-up visit<br>5. Table 3, 4 and 5 Schedule of Activities: Added clarification that the PROs should be done with the same frequency as the tumor assessments not visits<br>6. Table 5 Schedule of Activities: clarified that verification of inclusion/exclusion and demography only need to be collected at Cycle 1<br>7. Section 2.2.2.1 Current Recommendations for Treatment of BRAF V600E-mutant mCRC: added rationale for not including cetuximab in the control arm per health authority feedback<br>8. Section 2.3 Benefit/Risk Assessment:<br>• Clarified that levo-leucovorin and calcium folinate may be supplied in other countries and not limited to Japan and added definition of leucovorin. |



090177e1a02cb071\Approved\Approved On: 14-Mar-2024 15:31 (GMT)

| | | |
|---|---|---|
| | | • Added clarity that investigators should refer to locally approved product labels for the management of patients<br>9. Section 2.3.1 Risk Assessment: added adolescent participation discussion per health authority feedback<br>10. Section 5.1 Inclusion Criteria:<br>• Criteria 11 was updated to simplify definition of prior therapy<br>The following changes were made in response to health authority feedback:<br>• Criteria 1 was updated to only allow participants ≥16 years in countries where permitted<br>• Criteria 4 was updated to clarify participants must have stage IV disease and a note added for patients with oligometastatic disease<br>11. Section 5.2 Exclusion Criteria:<br>• Criteria 9 was clarified to exclude patients with unknown dMMR or MSI-H status<br>• Criteria 11e was updated to add clarification around eligibility of participants with bundle-branch block (BBB) or with an implanted cardiac pacemaker<br>• Criteria 18 was edited to further define prior treatments excluded in the SLI<br>• Criteria 21 was edited to exclude radiation therapy that included > 30% of the bone marrow<br>The following changes were made in response to health authority feedback:<br>• Criteria 8 was updated to add unknown RAS mutation status<br>• Criteria 17 was updated to clarify participants with residual Grade 2 peripheral neuropathy may only be treated in the FOLFIRI cohort in SLI<br>• Criteria 22 was modified to clarify definition of investigational products<br>12. Section 5.3 Lifestyle Considerations:<br>The following changes were made in response to health authority feedback:<br>• Added a statement regarding participant fertility preservation prior to study intervention<br>• Section 5.3.1 Contraception: added recommendations for duration of contraception follow-up to align with locally approved product labels<br>• Added Section 5.3.3 regarding avoiding extended exposure to ultraviolet light to align with locally approved product labels<br>13. Section 6 Study Intervention:<br>• Added clarity that investigators should refer to locally approved product labels for the management of patients<br>• Table 6.1: oxaliplatin dose formulation was corrected<br>• Table 6.1: added clarification that all study interventions except encorafenib may be provided as non-IMP in the USA only |

090177e1a02cb071\Approved\Approved On: 14-Mar-2024 15:31 (GMT)

| | | |
|---|---|---|
| | | • Table 6.1 added that calcium folinate may also be supplied and clarified that levo-leucovorin and calcium folinate may be supplied in other countries and not limited to Japan<br>14. Section 6.1.1 Safety Lead-In: in response to health authority feedback, clarified that a decision to proceed as a 2-arm study will be made prior to the start of Phase 3<br>15. Table 9 DLT Criteria: updates have been made to align with CTCAE dictionary terms<br>16. Section 6.1.2 Treatment Regimens:<br>• Have added text to indicate that study interventions should be administered in accordance with respective locally approved label or local institutional standards<br>• Table 10, 12, and 13: added that calcium folinate may also be supplied and removed Japan to allow flexibility for future use in other countries<br>• Table 10, 11, 12, and 13: have added clarification that all infusion times are approximate infusion times<br>17. Section 6.1.3.1 Administration of Encorafenib: Modified to align with recommended encorafenib dosing instructions<br>18. Section 6.1.3.2 Administration of Capecitabine, Cetuximab, 5-Fluorouracil, Irinotecan, Leucovorin, Oxaliplatin and Bevacizumab: have added that these study interventions may also be administered per local institutional standards<br>19. Section 6.5.1.4 Drugs with a Conditional or Possible Risk to Prolong the QT Interval and/or Induce Torsade de Pointes: added investigators should also use caution when administering oxaliplatin in response to health authority feedback<br>20. Section 6.5.2 Prohibited Concomitant Therapy: guidance has been added regarding the administration of COVID-19 vaccines<br>21. Section 6.5.3.5 Hematopoietic Growth Factors: added section to outline the use of hematopoietic growth factors on the study<br>22. Table 15 Recommended Encorafenib Dose Modifications: edits have been made to align with approved encorafenib label<br>23. Section 6.6 Dose Modifications: A statement previously inserted in Amendment 2 has been removed as it now conflicts with instruction that study interventions should be administered in accordance with local institutional standards<br>24. Section 6.6.2, 6.6.3, 6.6.3, 6.6.4, and 6.6.5 Dose Modifications for the other study interventions: added instruction to refer to the locally approved product labels for adverse reactions not specifically mentioned in response to health authority feedback<br>25. Table 20, 21 and 22 Recommended Dose Modifications: edits have been made to align with locally approved product labels<br>26. Table 21 Recommended Capecitabine Dose Reductions: table has been removed based it conflicted with the previous Table 22 Recommended Capecitabine Dose Modifications<br>27. Section 7.1 Discontinuation of Study Intervention: clarifications have been made throughout regarding the end of treatment and follow-up visits.<br>28. Section 8.1.1 Tumor Response Assessments: added clarification that skeletal target lesions should be imaged by CT, MRI or X-ray<br>29. Section 8.2.3 Physical Examinations: In response to health authority feedback, added clarification that new or worsening visual disturbances should be assessed |



090177e1a02cb071\Approved\Approved On: 14-Mar-2024 15:31 (GMT)

| | | |
|---|---|---|
| | | 30. Section 8.2.7 Electrocardiograms: added guidance per locally approved product labels in response to health authority feedback<br>31. Section 8.2.9 Pregnancy Testing: redundant language has been removed<br>32. Section 8.5 Pharmacokinetics<br>• Added clarification that PK sampling must occur on the day of dosing and that samples should not be drawn from the same arm as the IV is being administered<br>• Added clarification around timepoints relative to dosing<br>• Added a table for Phase 3 sampling schedule<br>• Added tables for PK sampling schedules for participants in China<br>33. 8.7.3 Molecular Prescreening: Moved the statement regarding registration in the IRT to the appropriate section 8.7.2 Screening<br>34. Section 8.8 Biomarkers: clarified that genomic profiling is to be done retrospectively<br>35. Section 8.10 Health Economics: added examples medical use resources<br>36. Section 9.1.1 Estimands:<br>• Removed estimand text and table for supportive sensitivity analyses as not required per protocol and are covered in Section 9.4.2<br>• Added definition of analysis population for primary and key secondary estimands<br>37. Section 9.2 Sample Size Determination:<br>• Added 1-sided p-values and removed the critical hazard ratios for Primary and Key Secondary Endpoints<br>The following changes were made in response to health authority feedback:<br>• Added clarification that a decision to proceed as a 2-arm study will be made prior to the start of Phase 3<br>• Added statistical details of the 2-arm study scenario<br>38. Section 9.3 Analysis Sets: Clarified the definition of DLT-evaluable analysis set<br>39. Section 9.4.1.1 Statistical Testing Strategy: Added clarification that a decision to proceed as a 2-arm study will be made prior to the start of Phase 3 per health authority feedback<br>40. Section 9.4.3.7 Patient Reported Outcomes: language was simplified as full description of the analysis will be included in the SAP<br>41. Section 9.5 Interim Analyses:<br>• Statement added regarding that the study may be terminated based on the interim analysis<br>• Added the 1-sided p-values for the futility boundary and removed the critical hazard ratios<br>The following changes were made in response to health authority feedback:<br>Added statistical details of the 2-arm study scenario<br>42. Appendix 2 Clinical Laboratory Tests:<br>• Table 34 Protocol-Required Safety Laboratory Assessments: LDH has been moved from tumor markers column to the appropriate Chemistry column and footnote added regarding total $CO_2$ requirements |



090177e1a02cb071\Approved\Approved On: 14-Mar-2024 15:31 (GMT)

| | | |
|---|---|---|
| | | • Text has been updated to align with the text in Section 8.2.8<br>43. Appendix 4, Section 10.4.2 Female Participant Reproductive Inclusion Criteria: duration of contraception in female participants was extended to until 9 months after the last dose of study intervention in compliance with locally approved product labels<br>44. Appendix 10:<br>• Updated instructions for completion of the PROs<br>• Updated the PRO scores to reflect actual forms being used<br>45. Appendix 12: added that alternative study measures are to be followed if allowed by local regulations. |
| Amendment 2 | 12 November 2020 | The primary purpose of this amendment is to incorporate MHRA recommendations.<br>1. Added Section 2.2.2.3 Management of Cancer Patients During COVID-19 Pandemic.<br>Rationale: to provide information to consider in the treatment of participants during the COVID-19 pandemic.<br>2. Section 2.3.1 Risk Assessment: revised to add risks associated with COVID-19 and potential mitigation strategies.<br>3. Section 2.3.3 Overall Benefit/Risk Conclusion: added statement regarding benefit/risk for participants during the current COVID-19 pandemic.<br>4. Section 6.6.6 Guidance for Administration of Study Intervention in Participants with SARS Cov-2 Infection: added reference to Appendix 12 for further mitigation strategies.<br>5. Section 8.7.2 *BRAF* Testing: added clarification that the kit used is partially CE-marked for its analytical performance according to 98/79/EC Annex VIII and is an US FDA approved medical device.<br>6. Section 8.7.5 Companion Diagnostic for Eligibility: added clarification that BRAF V600E test kit is partially CE marked.<br>7. Appendix 4, Section 10.4.1 Male Participant Reproductive Inclusion Criteria: duration of contraception in male participants was extended to until six months after the last dose of study intervention in compliance with the SmPCs of oxaliplatin and 5-fluorouracil.<br>8. Added Appendix 12, Alternative Measures During Public Emergencies: to describe possible alternative study measures to be followed during public emergencies, including the COVID-19 pandemic. |
| Amendment 1 | 07 August 2020 | The primary purpose of this amendment is to incorporate CHMP Scientific Advice recommendations. In addition, clarifications and administrative modifications have also been included.<br>1. Table 1 Schedule of Activities and Exclusion Criteria (Section 5.2, Criteria 5): Added that, where recommended by local label or guidelines, patients must be tested for dihydropyrimidine dehydrogenase (DPD) deficiency before initiating treatment with fluorouracil or capecitabine.<br>Rationale: Alignment with recent EMA guidance (EMA recommendations on DPD testing prior to treatment with fluorouracil, capecitabine, tegafur and flucytosine- 30 April, 2020). |



090177e1a02cb071\Approved\Approved On: 14-Mar-2024 15:31 (GMT)

| | | |
|---|---|---|
| | | 2. Risk/Benefit Assessment (Section 2.3) and Study Interventions Administered (Section 6.1): Added clarification that levo-leucovorin may be supplied only in Japan.<br>Rationale: To provide clarification levo-leucovorin will not be supplied as an IMP in other participating countries.<br>3. Table 13 Treatment Regimens (Control Arm): Added a clarifying statement that the 5-FU dose used in FOLFOXIRI regimen may be administered per local standard of care.<br>Rationale: As per CHMP Scientific Advice that pointed out different doses are considered standard-of-care in Europe and US.<br>4. Dose Modifications (Section 6.6): Added a statement to the dose modification that Day 1 and Day 15 chemotherapy infusions may not begin until serum potassium and magnesium are within institutional normal limits (replacement permitted).<br>Rationale: To maintain alignment with inclusion/exclusion criteria.<br>5. Genetics (Section 8.7): Added the following: Molecular profiling will be performed as described in Section 8.8. There is no intent to perform specific free-standing germline genomic analyses, but will be used as controls to assist in identifying and profiling somatic tumor mutations (See Section 8.8.2 for further details). No additional specified genetics will be evaluated in this study.<br>Rationale: To update text with recently revised standard language from Pfizer Translational Oncology.<br>6. Table 31 OS – Summary of Planned Efficacy Boundaries: A data entry error regarding the number of cumulative OS events was corrected. |
| Original protocol | 15 July 2020 | N/A |

090177e1a02cb071\Approved\Approved On: 14-Mar-2024 15:31 (GMT)

## REFERENCES

1. Bray F, Ferlay J, Soerjomataram I, et al. Global cancer statistics 2018: GLOBOCAN estimates of incidence and mortality worldwide for 36 cancers in 185 countries. CA Cancer J Clin. 2018;68(6):394-424.

2. Howlader N, Noone A, Krapcho M, et al. (2019). SEER Cancer Statistics Review, 1975-2016. National Cancer Institute. Bethesda, MD. Available from: https://seer.cancer.gov/csr/1975_2016/. Accessed on: 20 Apr 2020.

3. Editorial Board of the Cancer Statistics in Japan. (2018). Cancer statistics in Japan 2018. Foundation for Promotion of Cancer Research (FPCR). Available from: https://ganjoho.jp/data/reg_stat/statistics/brochure/2018/cancer_statistics_2018.pdf. Accessed on: 20 Apr 2020.

4. Altobelli E, D'Aloisio F, Angeletti PM. Colorectal cancer screening in countries of European Council outside of the EU-28. World J Gastroenterol 2016;22(20):4946-57.

5. Van Cutsem E, Cervantes A, Nordlinger B, et al. Metastatic colorectal cancer: ESMO Clinical Practice Guidelines for diagnosis, treatment and follow-up. Ann Oncol. 2014;25(suppl_3):iii1-9.

6. Barras D, Missiaglia E, Wirapati P, et al. BRAF V600E mutant colorectal cancer subtypes based on gene expression. Clin Cancer Res. 2017;23(1):104-15.

7. Bylsma LC, Gillezeau C, Garawin T, et al. Prevalence of RAS and BRAF mutations in metastatic colorectal cancer (mCRC) patients by tumor location. J Clin Oncol. 2018;36(4_suppl):681.

8. Clarke CN, Kopetz ES. BRAF mutant colorectal cancer as a distinct subset of colorectal cancer: clinical characteristics, clinical behavior, and response to targeted therapies. J Gastrointest Oncol. 2015;6(6):660-7.

9. Davies H, Bignell GR, Cox C, et al. Mutations of the BRAF gene in human cancer. Nature. 2002;417(6892):949-54.

10. De Roock W, Claes B, Bernasconi D, et al. Effects of KRAS, BRAF, NRAS, and PIK3CA mutations on the efficacy of cetuximab plus chemotherapy in chemotherapy-refractory metastatic colorectal cancer: a retrospective consortium analysis. Lancet Oncol. 2010;11(8):753-62.

090177e1a02cb071\Approved\Approved On: 14-Mar-2024 15:31 (GMT)

11. Sorbye H, Dragomir A, Sundström M, et al. High BRAF mutation frequency and marked survival differences in subgroups according to KRAS/BRAF mutation status and tumor tissue availability in a prospective population-based metastatic colorectal cancer cohort. PLoS One. 2015;10(6):e0131046.

12. Corcoran RB, Ebi H, Turke AB, et al. EGFR-mediated re-activation of MAPK signaling contributes to insensitivity of BRAF mutant colorectal cancers to RAF inhibition with vemurafenib. Cancer Discov. 2012;2(3):227-35.

13. Lochhead P, Kuchiba A, Imamura Y, et al. Microsatellite instability and BRAF mutation testing in colorectal cancer prognostication. J Natl Cancer Inst. 2013;105(15):1151-6.

14. Tran B, Kopetz S, Tie J, et al. Impact of BRAF mutation and microsatellite instability on the pattern of metastatic spread and prognosis in metastatic colorectal cancer. Cancer. 2011;117(20):4623-32.

15. Van Cutsem E, Köhne CH, Láng I, et al. Cetuximab plus irinotecan, fluorouracil, and leucovorin as first-line treatment for metastatic colorectal cancer: updated analysis of overall survival according to tumor KRAS and BRAF mutation status. J Clin Oncol. 2011;29(15):2011-9.

16. Venderbosch S, Nagtegaal ID, Maughan TS, et al. Mismatch repair status and BRAF mutation status in metastatic colorectal cancer patients: a pooled analysis of the CAIRO, CAIRO2, COIN, and FOCUS studies. Clin Cancer Res. 2014;20(20):5322-30.

17. Yuan Z-X, Wang X-Y, Qin Q-Y, et al. The prognostic role of BRAF mutation in metastatic colorectal cancer receiving anti-EGFR monoclonal antibodies: a meta-analysis. PLoS One. 2013;8(6):e65995.

18. Bokemeyer C, Van Cutsem E, Rougier P, et al. Addition of cetuximab to chemotherapy as first-line treatment for KRAS wild-type metastatic colorectal cancer: pooled analysis of the CRYSTAL and OPUS randomised clinical trials. Eur J Cancer. 2012;48(10):1466-75.

19. Cremolini C, Loupakis F, Antoniotti C, et al. FOLFOXIRI plus bevacizumab versus FOLFIRI plus bevacizumab as first-line treatment of patients with metastatic colorectal cancer: updated overall survival and molecular subgroup analyses of the open-label, phase 3 TRIBE study. Lancet Oncol. 2015;16(13):1306-15.

20. Douillard JY, Oliner KS, Siena S, et al. Panitumumab-FOLFOX4 treatment and RAS mutations in colorectal cancer. N Engl J Med. 2013;369(11):1023-34.



090177e1a02cb071\Approved\Approved On: 14-Mar-2024 15:31 (GMT)

21. Goey KKH, Elias SG, van Tinteren H, et al. Maintenance treatment with capecitabine and bevacizumab versus observation in metastatic colorectal cancer: updated results and molecular subgroup analyses of the phase 3 CAIRO3 study. Ann Oncol. 2017;28(9):2128-34.

22. Qin S, Li J, Wang L, et al. Efficacy and tolerability of first-line cetuximab plus leucovorin, fluorouracil, and oxaliplatin (FOLFOX-4) versus FOLFOX-4 in patients with RAS wild-type metastatic clorectal cancer: the open-label, randomized, phase III TAILOR trial. J Clin Oncol. 2018;36(30):3031-9.

23. Stintzing S, Miller-Phillips L, Modest DP, et al. Impact of BRAF and RAS mutations on first-line efficacy of FOLFIRI plus cetuximab versus FOLFIRI plus bevacizumab: analysis of the FIRE-3 (AIO KRK-0306) study. Eur J Cancer. 2017;79:50-60.

24. Le DT, Uram JN, Wang H, et al. PD-1 blockade in tumors with mismatch-repair deficiency. New England Journal of Medicine. 2015;372(26):2509-20.

25. Overman MJ, McDermott R, Leach JL, et al. Nivolumab in patients with metastatic DNA mismatch repair-deficient or microsatellite instability-high colorectal cancer (CheckMate 142): an open-label, multicentre, phase 2 study. The Lancet Oncology. 2017;18(9):1182-91.

26. Cohen R, Pellat A, Boussion H, et al. Immunotherapy and metastatic colorectal cancers with microsatellite instability or mismatch repair deficiency. Bulletin du cancer. 2019;106(2):137-42.

27. KEYTRUDA [pembrolizumab] USPI. US Prescribing Information; Jun 2020, Whitehouse Station, NJ, USA: Merck Sharp & Dohme Corp., a subsidiary of Merck & Co., Inc. Available from: https://www.accessdata.fda.gov/drugsatfda_docs/label/2020/125514s071s090lbl.pdf. Accessed on: 26 Jun 2020.

28. NCCN. NCCN Clinical Practice Guidelines in Oncology (NCCN Guidelines®): Colon Cancer, Version 2.2021. 2021. Available from: https://www.nccn.org/professionals/physician_gls/pdf/colon.pdf. Accessed on: 14 Jan 2021.

29. Pietrantonio F, Petrelli F, Coinu A, et al. Predictive role of BRAF mutations in patients with advanced colorectal cancer receiving cetuximab and panitumumab: a meta-analysis. Eur J Cancer. 2015;51(5):587-94.



090177e1a02cb071\Approved\Approved On: 14-Mar-2024 15:31 (GMT)

30. Rowland A, Dias MM, Wiese MD, et al. Meta-analysis of BRAF mutation as a predictive biomarker of benefit from anti-EGFR monoclonal antibody therapy for RAS wild-type metastatic colorectal cancer. Br J Cancer. 2015;112(12):1888-94.

31. Di Nicolantonio F, Martini M, Molinari F, et al. Wild-type BRAF is required for response to panitumumab or cetuximab in metastatic colorectal cancer. J Clin Oncol. 2008;26(35):5705-12.

32. Laurent-Puig P, Cayre A, Manceau G, et al. Analysis of PTEN, BRAF, and EGFR status in determining benefit from cetuximab therapy in wild-type KRAS metastatic colon cancer. J Clin Oncol. 2009;27(35):5924-30.

33. Loupakis F, Ruzzo A, Cremolini C, et al. KRAS codon 61, 146 and BRAF mutations predict resistance to cetuximab plus irinotecan in KRAS codon 12 and 13 wild-type metastatic colorectal cancer. Br J Cancer. 2009;101(4):715-21.

34. Maughan TS, Adams RA, Smith CG, et al. Addition of cetuximab to oxaliplatin-based first-line combination chemotherapy for treatment of advanced colorectal cancer: results of the randomised phase 3 MRC COIN trial. Lancet. 2011;377(9783):2103-14.

35. Van Cutsem E, Cervantes A, Adam R, et al. ESMO consensus guidelines for the management of patients with metastatic colorectal cancer. Ann Oncol. 2016;27(8):1386-422.

36. Investigator's Brochure, Encorafenib (PF-07263896). 24 June 2020.

37. BRAFTOVI [encorafenib] USPI. US Prescribing Information; Apr 2020, Boulder, CO, USA: Array BioPharma Inc., a wholly owned subsidiary of Pfizer Inc. Available from: https://www.accessdata.fda.gov/drugsatfda_docs/label/2020/210496s006lbl.pdf. Accessed on: 31 Mar 2020.

38. Kopetz S, Grothey A, Yaeger R, et al. Encorafenib, binimetinib, and cetuximab in BRAF V600E-mutated colorectal cancer. N Engl J Med. 2019;381(17):1632-43.

39. Addeo A, Friedlaender A. Cancer and COVID-19: unmasking their ties. Cancer Treat Rev. 2020;88:102041.

40. Vecchione L, Stintzing S, Pentheroudakis G, et al. ESMO management and treatment adapted recommendations in the COVID-19 era: colorectal cancer. ESMO Open. 2020;5(Suppl 3):e000826.

090177e1a02cb071\Approved\Approved On: 14-Mar-2024 15:31 (GMT)

41. Kuderer NM, Choueiri TK, Shah DP, et al. Clinical impact of COVID-19 on patients with cancer (CCC19): a cohort study. Lancet. 2020;395(10241):1907-18.

42. Lee LY, Cazier JB, Angelis V, et al. COVID-19 mortality in patients with cancer on chemotherapy or other anticancer treatments: a prospective cohort study. Lancet. 2020;395(10241):1919-26.

43. Robilotti EV, Babady NE, Mead PA, et al. Determinants of COVID-19 disease severity in patients with cancer. Nat Med. 2020;26(8):1218-23.

44. You YN, Lee LD, Deschner BW, et al. Colorectal cancer in the adolescent and young adult population. JCO Oncol Pract. 2020;16(1):19-27.

45. Gaspar N, Marshall LV, Binner D, et al. Joint adolescent-adult early phase clinical trials to improve access to new drugs for adolescents with cancer: proposals from the multi-stakeholder platform-ACCELERATE. Ann Oncol. 2018;29(3):766-71.

46. Hill DA, Furman WL, Billups CA, et al. Colorectal carcinoma in childhood and adolescence: a clinicopathologic review. J Clin Oncol. 2007;25(36):5808-14.

47. Ince I, Knibbe CA, Danhof M, et al. Developmental changes in the expression and function of cytochrome P450 3A isoforms: evidence from in vitro and in vivo investigations. Clin Pharmacokinet. 2013;52(5):333-45.

48. Lacroix D, Sonnier M, Moncion A, et al. Expression of CYP3A in the human liver--evidence that the shift between CYP3A7 and CYP3A4 occurs immediately after birth. Eur J Biochem. 1997;247(2):625-34.

49. Stevens JC, Hines RN, Gu C, et al. Developmental expression of the major human hepatic CYP3A enzymes. J Pharmacol Exp Ther. 2003;307(2):573-82.

50. FDA. Food and Drug Administration Guidance for Industry: considerations for the inclusion of adolescent patients in adult oncology clinical trials. March 2019. Available from: https://www.fda.gov/media/113499/download. Accessed on: 14 Jan 2021.

51. Chabot G, Abigerges D, Catimel G, et al. Population pharmacokinetics and pharmacodynamics of irinotecan (CPT-11) and active metabolite SN-38 during phase I trials. Ann Oncol. 1995;6(2):141-51.

52. de Man F, Goey AK, van Schaik RH, et al. Individualization of irinotecan treatment: a review of pharmacokinetics, pharmacodynamics, and pharmacogenetics. Clin Pharmacokinet. 2018;57(10):1229-54.



090177e1a02cb071\Approved\Approved On: 14-Mar-2024 15:31 (GMT)

53. CAMPTOSAR (irinotecan) USPI. 2020, NY, NY, USA: Pfizer Inc.

54. Nozawa T, Minami H, Sugiura S, et al. Role of organic anion transporter OATP1B1 (OATP-C) in hepatic uptake of irinotecan and its active metabolite, 7-ethyl-10-hydroxycamptothecin: in vitro evidence and effect of single nucleotide polymorphisms. Drug Metab Dispos. 2005;33(3):434-9.

55. Smith NF, Figg WD, Sparreboom A. Pharmacogenetics of irinotecan metabolism and transport: an update. Toxicol In Vitro. 2006;20(2):163-75.

56. Graham MA, Lockwood GF, Greenslade D, et al. Clinical pharmacokinetics of oxaliplatin: a critical review. Clinical Cancer Research. 2000;6(4):1205-18.

57. Leucovorin USPI. 2018, North Wales, PA: Teva Pharmaceuticals USA, Inc.

58. Miura K, Kinouchi M, Ishida K, et al. 5-fu metabolism in cancer and orally-administrable 5-fu drugs. Cancers. 2010;2(3):1717-30.

59. Tabernero J, Yoshino T, Kim T, et al. LBA26 - BREAKWATER safety lead-in (SLI): Encorafenib (E) + cetuximab (C) + chemotherapy (chemo) for BRAFV600E metastatic colorectal cancer (mCRC). Ann Oncol. 2022;33:S808-69.

60. Herbst RS, Prager D, Hermann R, et al. TRIBUTE: a phase III trial of erlotinib hydrochloride (OSI-774) combined with carboplatin and paclitaxel chemotherapy in advanced non-small-cell lung cancer. J Clin Oncol. 2005;23(25):5892-9.

61. Yoshino T, Arnold D, Taniguchi H, et al. Pan-Asian adapted ESMO consensus guidelines for the management of patients with metastatic colorectal cancer: a JSMO–ESMO initiative endorsed by CSCO, KACO, MOS, SSO and TOS. Annals of Oncology. 2018;29(1):44-70.

62. Cremolini C, Antoniotti C, Stein A, et al. FOLFOXIRI/bevacizumab (bev) versus doublets/bev as initial therapy of unresectable metastatic colorectal cancer (mCRC): A meta-analysis of individual patient data (IPD) from five randomized trials. J Clin Oncol. 2020;38(15_suppl):4015.

63. Loupakis F, Cremolini C, Masi G, et al. Initial therapy with FOLFOXIRI and bevacizumab for metastatic colorectal cancer. New England Journal of Medicine. 2014;371(17):1609-18.

64. US Food and Drug Administration. Guidance for Industry: Clinical Trial Endpoints for the Approval of Cancer Drugs and Biologics. Available from:



090177e1a02cb071\Approved\Approved On: 14-Mar-2024 15:31 (GMT)

https://www.fda.gov/regulatory-information/search-fda-guidance-documents/clinical-trial-endpoints-approval-cancer-drugs-and-biologics. Published: December 2018. Accessed 22 April 2020.

65. European Medicines Agency. Guideline on the evaluation of anticancer medicinal products in man. Available from: https://www.ema.europa.eu/en/documents/scientific-guideline/guideline-evaluation-anticancer-medicinal-products-man-revision-5_en.pdf. Published: September 2017. Accessed: 22 April 2020.

66. Richman SD, Seymour MT, Chambers P, et al. KRAS and BRAF mutations in advanced colorectal cancer are associated with poor prognosis but do not preclude benefit from oxaliplatin or irinotecan: results from the MRC FOCUS trial. J Clin Oncol. 2009;27(35):5931-7.

67. Zheng G, Tseng L-H, Haley L, et al. Clinical validation of coexisting driver mutations in colorectal cancers. Hum Pathol. 2019;86:12-20.

68. Grady WM, Carethers JM. Genomic and epigenetic instability in colorectal cancer pathogenesis. Gastroenterology. 2008;135(4):1079-99.

69. Sveen A, Kopetz S, Lothe RA. Biomarker-guided therapy for colorectal cancer: strength in complexity. Nat Rev Clin Oncol. 2020;17(1):11-32.

70. Thierry AR, Mouliere F, El Messaoudi S, et al. Clinical validation of the detection of KRAS and BRAF mutations from circulating tumor DNA. Nat Med. 2014;20(4):430-5.

71. Hong DS, Morris VK, El Osta B, et al. Phase IB study of vemurafenib in combination with irinotecan and cetuximab in patients with metastatic colorectal cancer with BRAFV600E mutation. Cancer Discov. 2016;6(12):1352-65.

72. Corcoran RB, André T, Atreya CE, et al. Combined BRAF, EGFR, and MEK inhibition in patients with BRAF(V600E)-mutant colorectal cancer. Cancer Discov. 2018;8(4):428-43.

73. Paweletz CP, Heavey GA, Kuang Y, et al. Early changes in circulating cell-free KRAS G12C predict response to adagrasib in KRAS mutant non-small cell lung cancer patients. Clin Cancer Res. 2023;29(16):3074-80.

74. Stires H, Zariffa N, Eisele M, et al. Changes in ctDNA levels as an early indicator of outcomes in advanced NSCLC treated with TKI: initial findings from a retrospective aggregate analysis of 8 clinical trials. J Clin Oncol. 2023;41(16_suppl):3030.



090177e1a02cb071\Approved\Approved On: 14-Mar-2024 15:31 (GMT)

75. Tabernero J, Ciardiello F, Rivera F, et al. Cetuximab administered once every second week to patients with metastatic colorectal cancer: a two-part pharmacokinetic/pharmacodynamic phase I dose-escalation study. Ann Oncol. 2010;21(7):1537-45.

76. Martín-Martorell P, Roselló S, Rodríguez-Braun E, et al. Biweekly cetuximab and irinotecan in advanced colorectal cancer patients progressing after at least one previous line of chemotherapy: results of a phase II single institution trial. Br J Cancer. 2008;99(3):455-8.

77. Mauri G, Durinikova E, Amatu A, et al. Empowering clinical decision making in oligometastatic colorectal cancer: the potential role of drug screening of patient-derived organoids. JCO Precis Oncol. 2021;5:PO.21.00143.

78. Smith TJ, Bohlke K, Lyman GH, et al. Recommendations for the Use of WBC Growth Factors: American Society of Clinical Oncology Clinical Practice Guideline Update. J Clin Oncol. 2015;33(28):3199-212.

79. US Food and Drug Administration. Patient-Focused Drug Development Guidance: Methods to Identify What is Important to Patients and Select, Develop or Modify Fit-for-Purpose Clinical Outcome Assessments. Available from: https://www.fda.gov/drugs/news-events-human-drugs/patient-focused-drug-development-guidance-methods-identify-what-important-patients-and-select. Published: October 2018. Accessed 03 May 2020.

80. Aaronson NK, Ahmedzai S, Bergman B, et al. The European Organization for Research and Treatment of Cancer QLQ-C30: a quality-of-life instrument for use in international clinical trials in oncology. J Natl Cancer Inst. 1993;85(5):365-76.

81. Griggs JJ, Mangu PB, Temin S, et al. Appropriate chemotherapy dosing for obese adult patients with cancer: American Society of Clinical Oncology clinical practice guideline. J Oncol Pract. 2012;8(4):e59-61.

82. Flaherty KT, Puzanov I, Kim KB, et al. Inhibition of mutated, activated BRAF in metastatic melanoma. N Engl J Med. 2010;363(9):809-19.

83. Kefford R, Arkenau H, Brown MP, et al. Phase I/II study of GSK2118436, a selective inhibitor of oncogenic mutant BRAF kinase, in patients with metastatic melanoma and other solid tumors. J Clin Oncol. 2010;28(15_suppl):8503.

84. Robert C, Arnault J-P, Mateus C. RAF inhibition and induction of cutaneous squamous cell carcinoma. Curr Opin Oncol. 2011;23(2):177-82.



090177e1a02cb071\Approved\Approved On: 14-Mar-2024 15:31 (GMT)

85. Yokota T, Ura T, Shibata N, et al. BRAF mutation is a powerful prognostic factor in advanced and recurrent colorectal cancer. Br J Cancer. 2011;104(5):856-62.

86. Jennison C, Turnbull BW. Group sequential methods with applications to clinical trials. Boca Raton, FL: Chapman & Hall/CRC; 1999.

87. Lan K, DeMets DL. Discrete sequential boundaries for clinical trials. Biometrika. 1983;70(3):659-63.

090177e1a02cb071\Approved\Approved On: 14-Mar-2024 15:31 (GMT)

# Protocol C4221015

## AN OPEN-LABEL, MULTICENTER, RANDOMIZED PHASE 3 STUDY OF FIRST-LINE ENCORAFENIB PLUS CETUXIMAB WITH OR WITHOUT CHEMOTHERAPY VERSUS STANDARD OF CARE THERAPY WITH A SAFETY LEAD-IN OF ENCORAFENIB AND CETUXIMAB PLUS CHEMOTHERAPY IN PARTICIPANTS WITH METASTATIC *BRAF* V600E-MUTANT COLORECTAL CANCER

**Statistical Analysis Plan**
**(SAP)**

**Version:** **1.0**

**Date:** **09 September 2020**

090177e194e374fe\Approved\Approved On: 10-Sep-2020 19:24 (GMT)

## TABLE OF CONTENTS


LIST OF TABLES ........................................................................................................6

LIST OF FIGURES .....................................................................................................6

APPENDICES ............................................................................................................6

1. VERSION HISTORY ................................................................................................7

2. INTRODUCTION .....................................................................................................8

2.1. Study Objectives, Endpoints, and Estimands ..........................................................8

2.1.1. Primary Estimands ...........................................................................................10

2.1.2. Secondary Estimand ........................................................................................11

2.1.3. Additional Estimands .......................................................................................12

2.2. Study Design ........................................................................................................13

3. ENDPOINTS AND BASELINE VARIABLES: DEFINITIONS AND CONVENTIONS ........................................................................................................15

3.1. Safety Lead-in .......................................................................................................15

3.1.1. Primary Endpoint .............................................................................................15

3.1.2. Secondary Endpoints ........................................................................................15

3.1.2.1. Safety Endpoints ...........................................................................................15

3.1.2.2. Efficacy Endpoints ........................................................................................15

3.1.2.3. PK Endpoints ................................................................................................16

3.1.3. Exploratory Endpoints ......................................................................................16

3.2. Phase 3 ...............................................................................................................16

3.2.1. Primary Endpoint .............................................................................................16

3.2.2. Efficacy Secondary Endpoints ..........................................................................17

3.2.2.1. Overall Survival ............................................................................................17

3.2.2.2. Objective Response Rate ...............................................................................17

3.2.2.3. Duration of Response ....................................................................................17

3.2.2.4. Progression-free Survival by Investigator ........................................................17

3.2.2.5. Time to Response ..........................................................................................17

3.2.2.6. PFS after Next Line of Treatment (PFS2) ........................................................17

3.2.3. Other Secondary Endpoints ...............................................................................18

3.2.3.1. Patient-Reported Outcomes Endpoints ............................................................18

3.2.3.2. Pharmacokinetic Endpoints ............................................................................18




090177e194e374fe\Approved\Approved On: 10-Sep-2020 19:24 (GMT)

3.2.3.3. Biomarker Endpoints....18
3.2.4. Exploratory Endpoints....19
3.3. Baseline Variables....19
3.3.1. Stratification....19
3.3.2. Covariates....20
3.4. Safety Endpoints....20
3.4.1. Adverse Events....20
3.4.2. Laboratory Data....21
3.4.3. Vital Signs Data....21
3.4.4. Electrocardiograms Data....21
4. ANALYSIS SETS (POPULATIONS FOR ANALYSIS)....21
5. GENERAL METHODOLOGY AND CONVENTIONS....23
5.1. Hypotheses and Decision Rules....23
5.1.1. Hypotheses and Sample Size Calculation....23
5.1.1.1. Safety Lead-in....23
5.1.1.2. Phase 3....23
5.1.2. Decision Rules....25
5.1.2.1. Safety Lead-in....25
5.1.2.2. Phase 3....26
5.2. General Methods....28
5.2.1. Data Handling After the Cut-off Date....29
5.2.2. Pooling of Data by Center....29
5.2.3. Analyses to Assess the Impact of COVID-19 Pandemic....29
5.2.4. COVID-19 Anchor Date....29
5.2.5. Definition of Start Date....30
5.2.6. Definition of Study Day....30
5.2.7. On-treatment Period....30
5.2.8. Date of Last Contact....30
5.2.9. Unscheduled Assessments....31
5.2.10. Tumor Assessment Date....31
5.2.11. Adequate Baseline Tumor Assessment....31
5.2.12. Adequate Post-baseline Tumor Assessment....32



090177e194e374fe\Approved\Approved On: 10-Sep-2020 19:24 (GMT)

5.2.13. Standard Derivations and Reporting Conventions ....................................32

5.2.14. Analyses for Binary and Categorical Endpoints......................................32

5.2.15. Analyses for Continuous Endpoints ........................................................32

5.2.16. Analyses for Time-to-Event Endpoints ...................................................32

5.3. Methods to Manage Missing Data ...............................................................34

5.3.1. Missing Dates ..........................................................................................34

5.3.2. Missing Patient-Reported Outcomes Data................................................38

5.3.3. Missing Pharmacokinetic Data ................................................................38

5.3.4. Missing Biomarker Data...........................................................................39

6. ANALYSES AND SUMMARIES ...................................................................39

6.1. Primary Endpoints.........................................................................................39

6.1.1. Main Analysis...........................................................................................39

6.1.2. Sensitivity/Supplementary Analyses .........................................................42

6.2. Efficacy Secondary Endpoints .....................................................................45

6.2.1. Overall Survival........................................................................................45

6.2.1.1. Main Analysis .......................................................................................45

6.2.1.2. Sensitivity/Supplementary Analysis .......................................................46

6.2.2. Objective Response Rate ..........................................................................47

6.2.3. Duration of Response ...............................................................................48

6.2.4. Time to Response .....................................................................................49

6.2.5. Progression-free Survival (Safety Lead-in)...............................................49

6.2.6. Progression-free Survival 2 ......................................................................49

6.2.7. Efficacy Endpoints of Arm A vs. Arm B Comparison..............................51

6.3. Other Secondary Endpoints...........................................................................52

6.3.1. Patient-Reported Outcomes.......................................................................52

6.3.1.1. Scoring ..................................................................................................52

6.3.1.2. Instrument Completion Rates.................................................................52

6.3.1.3. Descriptive Summary.............................................................................52

6.3.1.4. Health Status .........................................................................................52

6.3.2. Pharmacokinetic Analysis ........................................................................52

6.3.2.1. Safety Lead-in .......................................................................................52

6.3.2.2. Phase 3...................................................................................................57




090177e194e374fe\Approved\Approved On: 10-Sep-2020 19:24 (GMT)

6.3.3. Biomarker Analyses....58
6.3.3.1. Tumor Tissue Analyses....59
6.3.3.2. cfDNA Analyses....59
6.4. Subset Analyses....60
6.5. Baseline and Other Summaries and Analyses....61
6.5.1. Baseline Summaries....61
6.5.1.1. Demographic Summary....61
6.5.1.2. Baseline and Disease Characteristics....62
6.5.1.3. Stratification Factors....62
6.5.1.4. Medical History....62
6.5.1.5. Prior Anticancer Treatments....63
6.5.2. Study Conduct and Participant Disposition....63
6.5.2.1. Patient Disposition....63
6.5.2.2. Protocol Deviations....64
6.5.3. Study Treatment Exposure....64
6.5.3.1. Dose Modification....66
6.5.4. Concomitant Medications....67
6.5.5. Subsequent Anticancer Therapies/Procedures....67
6.6. Safety Summaries and Analyses....67
6.6.1. Adverse Events....68
6.6.2. Deaths....70
6.6.3. Adverse Events of Special Interest....71
6.6.4. Laboratory Data....72
6.6.5. Vital Signs....75
6.6.6. Electrocardiograms....76
7. INTERIM ANALYSES....77
7.1. Introduction....77
7.2. Interim Analyses and Summaries....77
7.2.1. Interim Futility Analysis for PFS....78
7.2.2. Interim Efficacy Analysis for OS....78
8. REFERENCES....80
9. APPENDICES....81



090177e194e374fe\Approved\Approved On: 10-Sep-2020 19:24 (GMT)

## LIST OF TABLES

Table 1: Summary of Change ....7
Table 2: Characteristics of DLT Probability During the DLT Evaluation Period ....25
Table 3: Probability of Observing ≥ 1 Instance of Toxicity at 10% and 15% Incidence Rate ....26
Table 4: PFS Outcome and Event Dates – Primary Analysis ....40
Table 5: PFS Censoring Reasons and Hierarchy - Primary Analysis ....41
Table 6: Reverse Censoring Rules for PFS Follow-up Duration ....42
Table 7: PFS Outcome and Event Dates – Supplementary Analysis ....43
Table 8: Discordance Summary for Investigator vs BICR Assessment on PFS ....44
Table 9: Reverse Censoring Rules for OS Follow-up Duration ....46
Table 10: Outcome, Event Dates, Censoring Reasons and Hierarchy for PFS2 Analyses ....50
Table 11: PK Sampling Schedule for EC + FOLFIRI Cohort in the Safety Lead-in ....53
Table 12: PK Sampling Schedule for EC + mFOLFOX6 Cohort in the Safety Lead-in ....54
Table 13: Pharmacokinetic Parameters ....56
Table 14: Clinical Notable ECG Criteria ....77
Table 15: OS – Summary of Planned Efficacy Boundaries ....79

## LIST OF FIGURES

Figure 1: Study Design ....14
Figure 2: Graphical Gatekeeping Multiple Testing Strategy ....28

## APPENDICES

Appendix 1. List of Abbreviations ....81

090177e194e374fe\Approved\Approved On: 10-Sep-2020 19:24 (GMT)

# 1. VERSION HISTORY

## Table 1: Summary of Change

| Version/ Date | Associated Protocol Amendment | Rationale | Specific Changes |
|---|---|---|---|
| 1.0/ 09 September 2020 | 1.0/ 15 July 2020 | N/A | N/A |
| | | | |

090177e194e374fe\Approved\Approved On: 10-Sep-2020 19:24 (GMT)

## 2. INTRODUCTION

This statistical analysis plan (SAP) provides the detailed methodology for summary and statistical analyses of the data collected in Study C4221015. This document may modify the plans outlined in the protocol; however, any major modifications of the primary endpoint and key secondary endpoint definition or corresponding analyses will also be reflected in a protocol amendment.

As this is an open-label study, the treatment is unblinded on participant level due to different treatment regimens in each treatment arm. However, the aggregate/cumulative data summary by actual treatment arm should remain blinded to the Sponsor and the external investigators until the database lock for the primary analysis.

### 2.1. Study Objectives, Endpoints, and Estimands

| **Safety Lead-in** | |
|---|---|
| **Objectives** | **Endpoints** |
| **Primary** | |
| • To determine the safety and tolerability of EC + mFOLFOX6 and EC + FOLFIRI | • Incidence of DLTs |
| **Secondary** | |
| • To assess the overall safety and tolerability of EC + mFOLFOX6 and EC + FOLFIRI | • Incidence and severity of AEs graded according to the NCI CTCAE v4.03 and changes in clinical laboratory parameters, vital signs and ECGs<br>• Incidence of dose interruptions, dose modifications and discontinuations due to AEs |
| • To estimate the efficacy of EC + mFOLFOX6 and EC + FOLFIRI | • ORR by Investigator, defined as the proportion of participants who have achieved a confirmed BOR (CR or PR) per RECIST v1.1<br>• DOR by Investigator, defined as the time from the date of first radiographic evidence of response (CR or PR) to the earliest documented disease progression per RECIST v1.1, or death due to any cause<br>• PFS by Investigator, defined as the time from the first dose to the earliest documented disease progression per RECIST v1.1, or death due to any cause<br>• TTR by Investigator, defined as the time from first dose to first radiographic evidence of response (CR or PR) per RECIST v1.1 |
| • To estimate the efficacy of EC + mFOLFOX6 and EC + FOLFIRI | • OS defined as the time from the first dose to death due to any cause |
| • To characterize the PK of encorafenib, irinotecan, oxaliplatin and relevant metabolites | • PK parameters of encorafenib, irinotecan, oxaliplatin and relevant metabolites |



090177e194e374fe\Approved\Approved On: 10-Sep-2020 19:24 (GMT)

| | |
|---|---|
| • To assess drug-drug interaction of encorafenib with irinotecan or oxaliplatin | • Changes in exposures of irinotecan and its metabolite (SN-38) on Cycle 1 Day 15 compared to Cycle 1 Day 1 in Cohort 1 (EC + FOLFIRI)<br>• Changes in exposures of oxaliplatin on Cycle 1 Day 15 compared to Cycle 1 Day 1 in Cohort 2 (EC + mFOLFOX6) |
| **Tertiary/Exploratory** | |
| • To understand the relationship between the therapeutic intervention(s) being studied and the biology of the participant's disease | • Measurements of biomarkers, consisting of DNA, RNA, protein or defined cell types, resulting from analyses of peripheral blood and/or tumor tissue biospecimen obtained at baseline, on treatment and/or at end-of-study |
| **Randomized Phase 3** | |
| **Objectives** | **Endpoints** |
| **Primary** | |
| • To compare the efficacy of EC (Arm A) vs SoC (Control Arm [Arm C]) as measured by PFS<br>• To compare the efficacy EC + mFOLFOX6 or EC + FOLFIRI (Arm B) vs the Control Arm as measured by PFS | • PFS by BICR, defined as the time from the date of randomization to the earliest documented disease progression per RECIST v1.1, or death due to any cause |
| **Key Secondary** | |
| • To compare the efficacy of Arm A vs the Control Arm as measured by OS<br>• To compare the efficacy of Arm B vs the Control Arm as measured by OS | • OS, defined as the time from the date of randomization to death due to any cause |
| **Secondary** | |
| • To compare the efficacy of Arm A vs the Control Arm as measured by ORR, DOR, PFS, PFS2 and TTR<br>• To compare the efficacy of Arm B vs the Control Arm as measured by ORR, DOR, PFS, PFS2 and TTR<br>• To compare the efficacy of Arm A vs Arm B as measured by OS, PFS, PFS2, ORR, DOR and TTR | • ORR by BICR and by Investigator<br>• DOR by BICR and by Investigator<br>• PFS by BICR<br>• OS<br>• PFS by Investigator<br>• TTR (by BICR and by Investigator), defined as the time from the date of randomization to first radiographic evidence of response (CR or PR) per RECIST v1.1<br>• PFS2, defined as the time from the date of randomization to the second objective disease progression per RECIST v1.1, or death from any cause, whichever occurs first |
| • To determine the safety and tolerability of EC<br>• To determine the safety and tolerability of EC + mFOLFOX6 or EC + FOLFIRI | • Incidence and severity of AEs graded according to the NCI CTCAE v4.03 and changes in clinical laboratory parameters, vital signs, and ECGs |



090177e194e374fe\Approved\Approved On: 10-Sep-2020 19:24 (GMT)

| | |
|---|---|
| • To compare the PRO measures | • PROs as measured by the EORTC QLQ-C30, EQ-5D-5L, PGIS and PGIC. |
| • To evaluate trough concentrations of encorafenib and its metabolite LHY746 in Arm A and Arm B | • Trough plasma concentrations of encorafenib and the metabolite LHY746 in Arm A and Arm B |
| • To characterize the PK of encorafenib and its metabolite LHY746 in participants randomized in China (Arm A) | • PK parameters of encorafenib and its metabolite LHY746 |
| • To confirm the MSI-status in tumor tissue | • Summarize MSI-status as determined by retrospective central testing of baseline tumor tissue |
| • To determine the correlation between cfDNA genetic alterations and clinical outcome | • *BRAF* V600E variant allele fraction (VAF) and/or overall mean VAF from cfDNA analysis of plasma samples collected at baseline and on treatment |
| **Tertiary/Exploratory** | |
| • To understand the relationship between the therapeutic intervention(s) being studied and the biology of the participant's disease | • Measurements of biomarkers, consisting of DNA, RNA, protein or defined cell types, resulting from analyses of peripheral blood and/or tumor tissue biospecimen obtained at baseline, on treatment and/or at end-of-study |

### 2.1.1. Primary Estimands

Estimands are defined below for the primary endpoint of incidence of dose-limiting toxicities (DLTs) in the Safety Lead-in (SLI) as well as the primary and key secondary endpoints in the Phase 3 portion of the study.

**Safety Lead-in**

The primary estimand for the SLI portion of the study is the DLT rate estimated based on data from DLT-evaluable participants during the DLT-evaluation period, which is the first 28 days after the first dose of study intervention in the SLI. It includes the following attributes:

- Population: all participants with local or central laboratory confirmed *BRAF* V600E- metastatic colorectal cancer (mCRC), as defined by the screening inclusion/exclusion criteria in Section 5.1.2 and Section 5.2.2 in the protocol to reflect the targeted population of the SLI portion of the study.
- Variable: DLT rate during the DLT-evaluation period, which is the first 28 days after the first dose of study intervention in the SLI. DLTs are defined in Section 6.1.1.1 in the study protocol.



090177e194e374fe\Approved\Approved On: 10-Sep-2020 19:24 (GMT)

- Intercurrent events: hypothetical strategy will be applied for the intercurrent events. The intercurrent event is treatment discontinuation for reasons other than treatment-related toxicity that leads to <75% of the planned dose of each study intervention during the DLT evaluation period. Participants without DLTs and with the intercurrent event will not be included in the DLT rate calculation.

- Population-level summary measure: DLT rate defined as the number of DLT-evaluable participants with DLTs in the DLT-evaluation period divided by the number of DLT-evaluable participants.

**Phase 3**

The primary estimand in the Phase 3 portion of the study is the treatment effect in progression-free survival (PFS) by blinded independent central review (BICR) per Response Evaluation Criteria in Solid Tumors version 1.1 (RECIST v1.1), as measured by the hazard ratio (HR). It is the hypothetical estimand and includes the following 4 attributes:

- Population: all participants with either a local or a central laboratory confirmed result of *BRAF* V600E-mutant mCRC, as defined by the screening inclusion/exclusion criteria in Section 5.1.2 and Section 5.2.2 in the protocol to reflect the targeted population of the Phase 3 portion of the study.

- Variable: PFS by BICR, defined as the time from the date of randomization to the earliest documented disease progression per RECIST v1.1, or death due to any cause.

- Intercurrent events: hypothetical strategy will be applied for the intercurrent events. Details of the intercurrent events and censoring rules for the primary analysis are summarized in Table 4.

- Population-level summary measure: HR for PFS and corresponding 2-sided CI based on Cox proportional hazard model stratified by Eastern Cooperative Oncology Group (ECOG) performance status at baseline and region. PFS will be compared between the two treatment arms using a 1-sided stratified log-rank test.

### 2.1.2. Secondary Estimand

The key secondary estimand in the Phase 3 portion of the study is the treatment effect in overall survival (OS), as measured by the HR. It is the hypothetical estimand and includes the following 4 attributes:

- Population: all participants with local or central laboratory confirmed *BRAF* V600E-mCRC, as defined by the screening inclusion/exclusion criteria in Section 5.1.2 and Section 5.2.2 in the study protocol to reflect the targeted population of the Phase 3 portion of the study.

- Variable: OS, defined as the time from date of randomization to death due to any cause.

090177e194e374fe\Approved\Approved On: 10-Sep-2020 19:24 (GMT)

- Intercurrent events: hypothetical strategy will be applied for the intercurrent events of lost to follow-up or withdrawal of consent/assent. Participants not known to have died are censored at the date of last contact.
- Population-level summary measure: HR for OS and corresponding 2-sided CI based on Cox proportional hazard model stratified by ECOG performance status at baseline and region. OS will be compared between the two treatment arms using a 1-sided stratified log-rank test.

### 2.1.3. Additional Estimands

The following supportive estimands are defined for the Phase 3 portion of the study:

**Supportive Estimand 1 (PFS):**

- Population: all participants with a central laboratory confirmed result of *BRAF* V600E-mutant mCRC, as defined by the screening inclusion/exclusion criteria in Section 5.1.2 and Section 5.2.2 in the study protocol to reflect the targeted population of the Phase 3 portion of the study.
- Variable: same as primary estimand of PFS.
- Intercurrent events: same as primary estimand of PFS.
- Population-level summary measure: same as primary estimand of PFS.

**Supportive Estimand 2 (PFS):**

- Population: same as primary estimand of PFS.
- Variable: same as primary estimand of PFS.
- Intercurrent events: treatment policy strategy will be applied for the intercurrent event of starting new anticancer therapy or undergoing metastasectomy procedures. All other intercurrent events will be addressed in the same approach as the primary estimand of PFS. Table 5 summarizes the details of the intercurrent events and censoring rules for this supportive analysis.
- Population-level summary measure: same as primary estimand of PFS.

**Supportive Estimand 3 (OS):**

- Population: all participants with a central laboratory confirmed result of *BRAF* V600E-mutant mCRC, as defined by the screening inclusion/exclusion criteria in Section 5.1.2 and Section 5.2.2 in the study protocol to reflect the targeted population of the Phase 3 portion of the study.
- Variable: same as key secondary estimand of OS.
- Intercurrent events: same as key secondary estimand of OS.
- Population-level summary measure: same as key secondary estimand of OS.

090177e194e374fe\Approved\Approved On: 10-Sep-2020 19:24 (GMT)

## 2.2. Study Design

C4221015 study is an open-label, multicenter, randomized Phase 3 study of encorafenib plus cetuximab (EC) with or without chemotherapy vs. standard of care chemotherapy in participants with previously untreated *BRAF* V600E-mutant mCRC. It includes a SLI portion and a randomized Phase 3 portion.

Prior to the Phase 3 portion, a SLI will be conducted at a limited number of sites to evaluate the safety/tolerability and PK of EC in combination with either mFOLFOX6 or FOLFIRI. Approximately 60 participants will be enrolled in the SLI portion (up to 30 per regimen). The results of the SLI will inform which chemotherapy regimen is used in Arm B of the Phase 3 portion of the study.

In the Phase 3 portion, approximately 870 participants will be randomized 1:1:1 to receive EC (Arm A), EC + chemotherapy (Arm B) or standard of care (SoC) chemotherapy (Control Arm), with approximately 290 participants per arm. The primary objectives in the Phase 3 portion of the study are to compare the efficacy, as measured by the primary endpoint of PFS by BICR, of Arm A vs. the Control Arm and of Arm B vs. the Control Arm. The study will be considered positive (ie, demonstrated evidence of effectiveness) if at least one of the hypothesis tests of the primary endpoint is statistically significant.

A schematic of the study design is presented in Figure 1.



090177e194e374fe\Approved\Approved On: 10-Sep-2020 19:24 (GMT)

**Figure 1: Study Design**

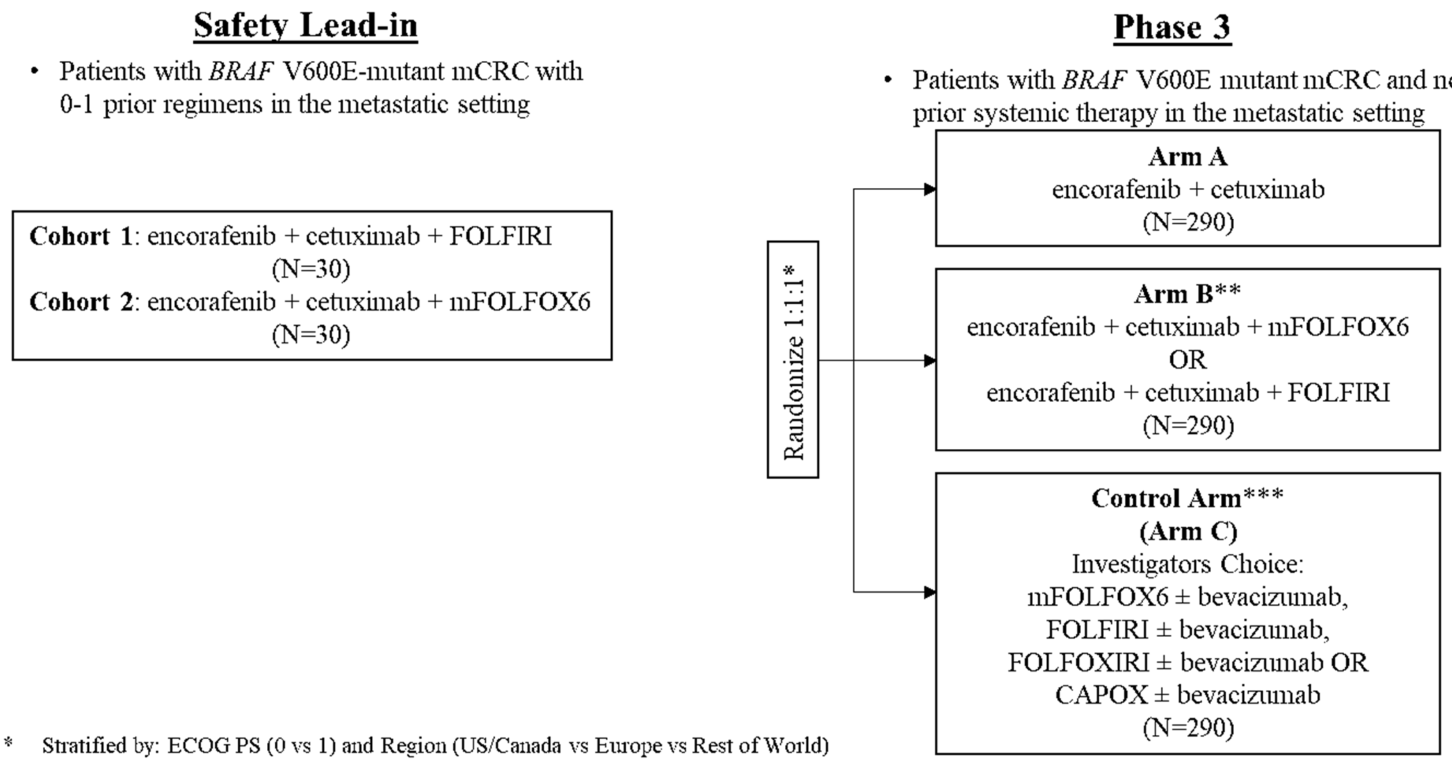


* Stratified by: ECOG PS (0 vs 1) and Region (US/Canada vs Europe vs Rest of World)

** Use of mFOLFOX6 or FOLFIRI in Arm B will depend on results of the SLI portion of the study. If neither SLI regimen is tolerated, Phase 3 will proceed without Arm B.

*** If mFOLFOX6 is used in Arm B, the Control Arm will consist of investigator's choice of mFOLFOX6 ± bevacizumab, FOLFOXIRI ± bevacizumab or CAPOX ± bevacizumab. If FOLFIRI is used in Arm B, the Control Arm will consist of investigator's choice of FOLFIRI ± bevacizumab or FOLFOXIRI ± bevacizumab. If Phase 3 proceeds without Arm B, the Control Arm will consist of investigator's choice of mFOLFOX6 ± bevacizumab, FOLFIRI ± bevacizumab, FOLFOXIRI ± bevacizumab or CAPOX ± bevacizumab.



090177e194e374fe\Approved\Approved On: 10-Sep-2020 19:24 (GMT)

# 3. ENDPOINTS AND BASELINE VARIABLES: DEFINITIONS AND CONVENTIONS

## 3.1. Safety Lead-in

### 3.1.1. Primary Endpoint

The primary endpoint for the SLI is incidence of DLTs estimated based on data from DLT-evaluable participants during the DLT-evaluation period, which is the first 28 days after the first dose of study intervention in the SLI. The criteria of DLTs are specified in Section 6.1.1.1 in the protocol.

### 3.1.2. Secondary Endpoints

#### 3.1.2.1. Safety Endpoints

- Incidence and severity of adverse events (AEs) graded according to the National Cancer Institute Common Terminology Criteria for Adverse Events version 4.03 (NCI CTCAE v4.03) and changes in clinical laboratory parameters, vital signs and ECGs.
- Incidence of dose interruptions, dose modifications and discontinuations due to AEs.

#### 3.1.2.2. Efficacy Endpoints

**Objective Response Rate**

Objective response rate (ORR) is defined as the proportion of participants who have achieved a best overall response (BOR) of complete response (CR) or partial response (PR) per RECIST v1.1. The BOR is the best response obtained among all tumor assessment visits after the date of first dose until documented disease progression, death, start of subsequent anticancer therapy or undergoing metastasectomy procedures. Clinical deterioration or clinical progression noted on the completion eCRF will not be considered as documented disease progression.

ORR based on Investigator assessment will be calculated for the SLI participants.

**Duration of Response**

Duration of response (DOR) is defined as the time from the date of first radiographic evidence of response (CR or PR) to the earliest documented disease progression per RECIST v1.1, or death due to any cause.

DOR based on Investigator assessment will be calculated for the SLI participants with a confirmed response (CR or PR).



090177e194e374fe\Approved\Approved On: 10-Sep-2020 19:24 (GMT)

**Progression-free Survival**

PFS is defined as the time from date of the first dose to the earliest documented disease progression per RECIST v1.1, or death due to any cause.

The censoring and event date options to be considered for the PFS analysis are described in details in Section 6.1.1.

PFS based on Investigator assessment will be calculated for the SLI participants.

**Time to Response**

Time to response (TTR) is defined as the time from the date of first dose to first radiographic evidence of response (CR or PR) per RECIST v1.1. TTR will be calculated for participants with a confirmed response (CR or PR).

TTR based on Investigator assessment will be calculated for the SLI participants.

**Overall Survival**

OS is defined as the time from the date of first dose to death due to any cause. If a participant is not known to have died at the time of the cutoff for analysis, then OS will be censored at the date of last contact.

#### 3.1.2.3. PK Endpoints

- PK parameters of encorafenib, irinotecan, oxaliplatin and relevant metabolites.
- Changes in exposures of irinotecan and its metabolite (SN-38) on Cycle 1 Day 15 compared to Cycle 1 Day 1 in Cohort 1 (EC + FOLFIRI).
- Changes in exposures of oxaliplatin on Cycle 1 Day 15 compared to Cycle 1 Day 1 in Cohort 2 (EC + mFOLFOX6).

### 3.1.3. Exploratory Endpoints

Measurements of biomarkers, consisting of DNA, RNA, protein or defined cell types, resulting from analyses of peripheral blood and/or tumor tissue biospecimen obtained at baseline, on treatment and/or at end-of-study.

## 3.2. Phase 3

### 3.2.1. Primary Endpoint

PFS by BICR is defined as the time from date of randomization to the earliest documented disease progression per RECIST v1.1 based on BICR assessment, or death due to any cause.

The censoring and event date options to be considered for the PFS primary analysis are described in details in Section 6.1.1.



090177e194e374fe\Approved\Approved On: 10-Sep-2020 19:24 (GMT)

### 3.2.2. Efficacy Secondary Endpoints

#### 3.2.2.1. Overall Survival

The key secondary endpoint of OS is defined as the time from the date of randomization to death due to any cause. If a participant is not known to have died at the time of the cutoff for analysis, then OS will be censored at the date of last contact.

#### 3.2.2.2. Objective Response Rate

ORR is defined as the proportion of participants who have achieved a BOR of CR or PR per RECIST v1.1. The BOR is the best response obtained among all tumor assessment visits after the date of randomization until documented disease progression, death, or start of subsequent anticancer therapy. Clinical deterioration or clinical progression noted on the completion eCRF will not be considered as documented disease progression.

Both ORR by BICR and ORR by Investigator will be calculated for randomized participants in the Phase 3 portion of the study.

#### 3.2.2.3. Duration of Response

DOR in the Phase 3 poriton of the study has the same definition of DOR as described in Section 3.1.2.2.

Both DOR by BICR and DOR by Investigator will be calculated for randomized participants in the Phase 3 portion of the study.

#### 3.2.2.4. Progression-free Survival by Investigator

PFS by Investigator is defined as the time from date of randomization to the earliest documented disease progression per RECIST v1.1 based on Investigator assessment, or death due to any cause.

#### 3.2.2.5. Time to Response

TTR is defined as the time from the date of randomization to first radiographic evidence of response (CR or PR) per RECIST v1.1. TTR will be calculated for participants with a confirmed response (CR or PR).

Both TTR by BICR and TTR by Investigator will be calculated for randomized participants in the Phase 3 portion of the study.

#### 3.2.2.6. PFS after Next Line of Treatment (PFS2)

PFS after next line of treatment (PFS2) is defined as the time from the date of randomization to second objective disease progression per RECIST v1.1, or death from any cause, whichever occurs first. Second objective disease progression is progressive disease (PD) after the start of subsequent anticancer therapy based on Investigator assessment.

090177e194e374fe\Approved\Approved On: 10-Sep-2020 19:24 (GMT)

### 3.2.3. Other Secondary Endpoints

#### 3.2.3.1. Patient-Reported Outcomes Endpoints

Patient-reported outcomes (PROs) as measured by the following instruments: European Organization for Treatment of Cancer Quality of Life Questionnaire (EORTC QLQ-C30), the EuroQol EQ-5D-5L (EQ-5D-5L) and visual analogue scale (VAS), patient global impression of disease severity (PGIS), and patient global impression of change (PGIC).

The EORTC QLQ-C30 is a 30-question survey, which can be grouped into 5 functional domain subscales, including a physical functioning subscale, a role functioning subscale, an emotional functioning subscale, a cognitive functioning subscale and a social functioning subscale. Higher scores on the functional domains are indicative of higher levels of functioning. Oncology related symptoms assessed by the EORTC QLQ-C30 include fatigue (3 items), pain (2 items), nausea and vomiting (2 items), and dyspnea, insomnia, appetite loss, constipation, diarrhea, and financial impact (1 item each). Higher scores are reflective of a greater presence of symptoms.

The EQ-5D-5L is a 6-item patient-completed questionnaire designed to assess health status in terms of a single index value or utility score1. There are 2 components, a Health State Profile which has individuals rate their level of problems (none, slight, moderate, severe, extreme/unable) in 5 areas (mobility, self-care, usual activities, pain/discomfort, and anxiety/depression), and a VAS in which participants rate their overall health status from 0 (worst imaginable) to 100 (best imaginable). Published weights are available that allow for the creation of a single summary score. Overall scores range from 0 to 1, with lower scores representing a higher level of dysfunction.

The PGIS is a single-item PRO designed to facilitate an anchor-based methodology for establishing meaningful within person change (MWPC) of other PRO-based endpoints such as the EORTC QLQ-C30. There are 5 response options, ranging from “none” to “very severe”.

The PGIC is a single-item PRO designed to assess the participant’s overall impression of the degree of change they have experienced since the start of study treatment. There are 7 response options, ranging from “very much improved” to “very much worse”.

#### 3.2.3.2. Pharmacokinetic Endpoints

- Trough plasma concentrations of encorafenib and the metabolite LHY746 in Arm A and Arm B.
- PK parameters of encorafenib and its metabolite LHY746 in participants randomized in China (Arm A).

#### 3.2.3.3. Biomarker Endpoints

- Microsatellite instability (MSI)-status as determined by retrospective central testing of baseline tumor tissue.



090177e194e374fe\Approved\Approved On: 10-Sep-2020 19:24 (GMT)

- *BRAF* V600E variant allele fraction (VAF) and/or overall mean VAF from cfDNA analysis of plasma samples collected at baseline and on treatment.

### 3.2.4. Exploratory Endpoints

Measurements of biomarkers, consisting of DNA, RNA, protein or defined cell types, resulting from analyses of peripheral blood and/or tumor tissue biospecimen obtained at baseline, on treatment and/or at end-of-study.

## 3.3. Baseline Variables

The date of first dose of study treatment is the earliest date of non-zero dosing of any study drug. The date of last dose of study treatment is the latest date of non-zero dosing of any study drug.

For all endpoints assessments in the SLI and all safety assessments in the Phase 3 portion of the study, baseline is defined as the last completed assessment prior to date of first dose of study treatment in the SLI and the Phase 3, respectively. If an assessment that is planned to be performed prior to the first dose of study treatment in the protocol is performed on the same day as the first dose of study treatment and the time is unknown, it will be assumed that it was performed prior to study treatment administration and will be considered as baseline assessment.

For efficacy assessments in the Phase 3 portion of the study, baseline is defined as the last assessment prior to randomization. If the assessment that is planned to be performed before randomization per the protocol is performed on the same day as the date of randomization and assessment time point is missing, it will be assumed that it was performed prior to randomization and will be considered as baseline assessment. If such a value is missing, the last measurement prior to the first dose of study treatment will be used as the baseline measurement for the efficacy analysis except for analyses of tumor assessments data where the baseline assessment would be considered missing.

For PRO the last measurement prior to or on the first dose of study treatment will be used as the baseline measurement. If there are no measurements meeting these criteria, then baseline is considered missing.

Unscheduled assessments will be used in the determination of baseline. Data reported at the End of Treatment visit are not eligible for baseline selection.

### 3.3.1. Stratification

Randomization is stratified by the following factors as recorded in the Interactive Response Technology (IRT) system:

- ECOG performance status: 0 vs. 1.
- Region: US/Canada vs. Europe vs. Rest of World.

090177e194e374fe\Approved\Approved On: 10-Sep-2020 19:24 (GMT)

All stratified analysis will utilize the strata defined in the IRT system.

### 3.3.2. Covariates

In addition to the randomization strata, the following baseline characteristics variables might be included as covariates in the Cox regression model to explore the potential prognostic impact on PFS per BICR and OS.

- Age (<65 years vs. ≥65 years)
- Gender (Male vs. Female).
- Race (Caucasian vs. non-Caucasian).
- Race (Asian vs. non-Asian).
- Number of organs involved at baseline (≤2 vs. ≥3).
- *BRAF* V600E mutation per central assessment (Positive vs. Negative/Indeterminate).
- Prior anticancer adjuvant/ neoadjuvant therapy (yes vs. no).
- *BRAF* V600E mutation per central assessment (Positive vs. Negative/Indeterminate).
- Removal status of primary tumor (no resection/ partial resection vs. complete resection).
- Side of tumor (left/right vs. left vs. right).
- Presence of liver metastases at baseline, based on target and non-target lesion assessment (yes vs. no).

## 3.4. Safety Endpoints

Safety endpoints will be summarized based on the on-treatment period unless otherwise specified. Safety data collected outside the on-treatment period will be listed but not summarized.

### 3.4.1. Adverse Events

An AE is considered a treatment-emergent adverse event (TEAE) if the event starts on or after the first dosing day and time/start time, if collected, but before the last dose +28 days, or start of subsequent anticancer therapy minus 1 day, whichever occurs first. Any events that started prior to the first dose date are not considered TEAEs. If an AE starts on the same day as the first dose date, it will be considered treatment emergent unless the CRF data indicates otherwise via explicitly recording time for AE onset and treatment dosing.

090177e194e374fe\Approved\Approved On: 10-Sep-2020 19:24 (GMT)

Adverse events will be coded using current Medical Dictionary for Regulatory Activities (MedDRA). Severity of AEs will be graded using the NCI-CTCAE v 4.03 toxicity grading scale.

### 3.4.2. Laboratory Data

Hematology, chemistry and coagulation tests results will be programmatically graded according to the NCI CTCAE version 4.03 for relevant parameters. Additional details are provided in Section 6.6.4.

### 3.4.3. Vital Signs Data

Vital signs data includes weight, pulse, systolic blood pressure (BP), and diastolic BP will be summarized. Additional details are provided in Section 6.6.5.

### 3.4.4. Electrocardiograms Data

Clinically notable ECG values during the on-treatment period will be summarized. Additional details are provided in Section 6.6.6.

## 4. ANALYSIS SETS (POPULATIONS FOR ANALYSIS)

Data for all participants will be assessed to determine if participants meet the criteria for inclusion in each analysis population prior to unblinding and releasing the database and classifications will be documented per standard operating procedures.

| Participant Analysis Set | Description |
|---|---|
| **Enrolled** | All participants who sign the Screening informed consent/assent document. |
| **DLT-Evaluable Analysis Set** | All participants who receive at least 1 dose of study drug in the SLI and either experience DLT during the DLT evaluation period or complete the DLT evaluation period without DLT. Participants without DLTs who withdraw from study treatment before receiving at least 75% of the planned dose of each study drug during the DLT evaluation period for reasons other than treatment-related toxicity are not evaluable for DLT. The DLT evaluation period is the first 28 days after the first dose of study intervention. |
| **Full Analysis Set (FAS)** | For participants in the SLI, the FAS includes all participants who receive at least 1 dose of study drug.<br><br>For participants in the Phase 3 portion, the FAS includes all participants who are randomized in the Phase 3 portion of the study. Participants will be analyzed according to the study treatment assigned at randomization. |

090177e194e374fe\Approved\Approved On: 10-Sep-2020 19:24 (GMT)

| Participant Analysis Set | Description |
|---|---|
| **Central *BRAF* V600E Positive Analysis Set** | All participants who are randomized in the Phase 3 portion of the study with a confirmed central laboratory result of *BRAF* V600E mutation. Participants will be analyzed according to the study treatment assigned at randomization. |
| **Safety Analysis Set** | All participants who receive at least 1 dose of study drug.<br><br>For the Phase 3 portion of the study, participants will be analyzed according to the study treatment assigned at randomization unless the incorrect treatment(s) was/were received throughout the dosing period in which case participants will be analyzed according to the actual study treatment received. |
| **Patient-Reported Outcome (PRO) Analysis Set** | All participants from the FAS who completed a baseline (last PRO assessment prior to or on the first dose of study treatment) and at least 1 post-baseline PRO assessment. |
| **PK Analysis Set** | The PK concentration set is defined as all enrolled participants who are treated and have at least 1 analyte concentration.<br><br>The PK parameter analysis set is defined as all enrolled participants treated who have sufficient information to estimate at least 1 of the PK parameters of interest and have no major protocol deviations affecting PK assessment. |
| **Biomarker Analysis Set** | All participants who are in the safety set and who had at least 1 of the pharmacodynamics or biomarkers evaluated at pre and/or post dose. Analysis sets will be defined separately for tumor tissue-based and blood-based biomarkers and will also be defined separately for non-paired and paired biomarkers.<br><br>The tumor tissue analysis set is defined as participants from the safety analysis set who had a tumor biomarker assessment at baseline.<br><br>The cfDNA analysis set is defined as participants from the safety analysis set who have a blood-based biomarker assessment at baseline.<br><br>The paired cfDNA analysis set is defined as all patients in the safety analysis set who have valid paired results and a blood-based biomarker assessment at baseline and post-treatment (eg, C1D15, C2D1, C2D15, EOT). |

090177e194e374fe\Approved\Approved On: 10-Sep-2020 19:24 (GMT)

| Participant Analysis Set | Description |
|---|---|
| | The tumor diagnostics concordance analysis set is defined as participants who have a result from both central tumor and local (captured in the CRF) tumor/plasma testing.<br><br>The cfDNA diagnostics concordance analysis set is defined as participants who have both a central tumor tissue result and a central plasma result. |

# 5. GENERAL METHODOLOGY AND CONVENTIONS

## 5.1. Hypotheses and Decision Rules

### 5.1.1. Hypotheses and Sample Size Calculation

The planned sample size of the study is approximately 930 participants including approximately 60 participants in the SLI, and approximately 870 participants in the Phase 3 portion of the study.

#### 5.1.1.1. Safety Lead-in

There is no statistical hypothesis for the SLI portion of the study.

The primary endpoint of the SLI is incidence of DLTs. Up to 60 participants will be enrolled on a rolling basis in an alternating manner to receive either EC + mFOLFOX6 or EC + FOLFIRI (up to 30 participants per cohort). The safety data will be evaluated by the Steering Committee and External Data Monitor Committee (E-DMC) after the first 9 evaluable participants in each cohort have been followed for a minimum of 28 days with EC + mFOLFOX6 or EC + FOLFIRI. The committees will also review the cumulative safety and PK data at the end of the SLI when all evaluable participants in each of the two cohort have been followed for a minimum of 28 days.

#### 5.1.1.2. Phase 3

A total of approximately 870 eligible participants will be randomized in a 1:1:1 ratio, stratified by ECOG performance status (0 vs 1) and region (US/Canada vs. Europe vs. Rest of World), to one of the three treatment arms (ie, approximately 290 participants/arm). It is assumed that participant accrual will reach a maximum of 40 participants per month for an accrual duration of approximately 27 months.

090177e194e374fe\Approved\Approved On: 10-Sep-2020 19:24 (GMT)

090177e194e374fe\Approved\Approved On: 10-Sep-2020 19:24 (GMT)

**Primary Endpoint**

The primary objectives in the Phase 3 portion of the study are to compare the efficacy, as measured by the primary endpoint of PFS by BICR, of Arm A vs. the Control Arm and of Arm B vs. the Control Arm. The following statistical hypotheses will be tested to address the primary objectives:

$H_{01}$: $HR_{PFS\ (A\ vs\ Control)} \geq 1$ vs $H_{11}$: $HR_{PFS\ (A\ vs\ Control)} < 1$

$H_{02}$: $HR_{PFS\ (B\ vs\ Control)} \geq 1$ vs $H_{12}$: $HR_{PFS\ (B\ vs\ Control)} < 1$

where $HR_{PFS\ (A\ vs\ Control)}$ is the HR for PFS of Arm A vs. the Control Arm and $HR_{PFS\ (B\ vs\ Control)}$ is the HR for PFS of Arm B vs. the Control Arm.

The primary analysis of PFS will occur once both of the following criteria are met: at least 393 BICR-assessed PFS events are observed in Arm A + the Control Arm and at least 313 BICR-assessed PFS events are observed in Arm B + the Control Arm. It is anticipated that this will occur approximately 34 months after the first participant is randomized. It is anticipated that approximately 380 PFS events will be observed in Arm B + the Control Arm by this time, but only the first 313 PFS events will be included in the primary analysis.

For the comparison of Arm A vs Control PFS, this design will achieve 90% power to detect a HR of 0.70 with a 1-sided alpha of 0.0125 under the assumption that PFS will follow an exponential distribution and approximately 10% of participants will be lost to follow-up. This corresponds to an improvement in the median PFS of 3 months (10 months in Arm A vs 7 months in the Control Arm). With a total of 393 BICR-assessed PFS events in Arm A + the Control Arm, the null hypothesis that the PFS in Arm A is the same or worse than the Control Arm will be rejected if the HR is smaller (ie, better) than 0.80 at the primary analysis.

For the comparison of Arm B vs Control PFS, this design will achieve 90% power to detect a HR of 0.67 with a 1-sided alpha of 0.0125 under the same assumption of PFS distribution and dropout rate as Arm A. This corresponds to an improvement in the median PFS of 3.5 months (10.5 months in Arm B vs. 7 months in the Control Arm). With a total of 313 BICR-assessed PFS events in Arm B + the Control Arm, the null hypothesis that the PFS in Arm B is the same or worse than the Control Arm will be rejected if the HR is smaller than 0.78 at the primary analysis.

**Key Secondary Endpoint**

The key secondary objectives are to compare the efficacy, as measured by the key secondary endpoint of OS, of Arm A vs. the Control Arm and of Arm B vs. the Control Arm. The following statistical hypotheses will be tested to address the key secondary objectives:

$H_{03}$: $HR_{OS\ (A\ vs\ Control)} \geq 1$ vs $H_{13}$: $HR_{OS\ (A\ vs\ Control)} < 1$

$H_{04}$: $HR_{OS\ (B\ vs\ Control)} \geq 1$ vs $H_{14}$: $HR_{OS\ (B\ vs\ Control)} < 1$

where $HR_{OS\ (A\ vs\ Control)}$ is the HR for OS of Arm A vs. the Control Arm and $HR_{OS\ (B\ vs\ Control)}$ is the HR for OS of Arm B vs. the Control Arm.

The final analysis of OS will occur once both of the following criteria are met: at least 403 OS events are observed in Arm A + the Control Arm and at least 344 OS events are observed in Arm B + the Control Arm. This is expected to occur approximately 49 months after the first participant is randomized (ie, approximately 22 months after randomization is complete). At the time of the final analysis of OS, it is anticipated that approximately 400 OS events will be observed in Arm B + the Control Arm by this time, but only the first 344 OS events will be included.

For the comparison of Arm A vs. Control OS, 403 OS events in Arm A + the Control Arm will achieve 85% power to detect an OS HR of 0.72 with a 1-sided alpha of 0.0125 under the assumption that OS will follow an exponential distribution and approximately 5% of participants will be lost to follow-up. This corresponds to an improvement in the median OS of 5.8 months (20.8 months in Arm A vs. 15 months in the Control Arm). With a total of 403 OS events in Arm A + the Control Arm, the null hypothesis that the OS in Arm A is the same or worse than the Control Arm will be rejected if the HR is smaller (ie, better) than 0.80 at the final analysis of OS.

For the comparison of Arm B vs. Control OS, 344 OS events in Arm B + the Control Arm will achieve 85% power to detect an OS HR of 0.70 with a 1-sided alpha of 0.0125 under the same assumption of OS distribution and dropout rate as Arm A. This corresponds to an improvement in the median OS of 6.4 months (21.4 months in Arm B vs. 15 months in the Control Arm). With a total of 344 OS events in Arm B + the Control Arm, the null hypothesis that the OS in Arm B is the same or worse than the Control Arm will be rejected if the HR is smaller than 0.78 at the final analysis of OS.

### 5.1.2. Decision Rules

#### 5.1.2.1. Safety Lead-in

The target DLT rate in the first 28 days in the SLI is <33% (ie, <3 out of 9 evaluable participants with DLTs).

Table 2 provides a comparison of the characteristics of this DLT evaluation rule and the traditional 3 + 3 rules based on 9 participants.

**Table 2: Characteristics of DLT Probability During the DLT Evaluation Period**

| True DLT Rate During DLT Evaluation Period | Probability of Dose Declared Toxic Using 3 + 3 Rules | Probability of Observed DLT Rate ≥33% in 9 Participants During DLT Evaluation Period |
|---|---|---|
| 10% | 0.094 | 0.053 |
| 20% | 0.291 | 0.262 |
| 30% | 0.506 | 0.537 |
| 40% | 0.691 | 0.768 |
| 50% | 0.828 | 0.910 |



090177e194e374fe\Approved\Approved On: 10-Sep-2020 19:24 (GMT)

The DLT evaluation period is the first 28 days after the first dose of study intervention in the SLI, and the DLT-evaluable is defined in Section 4.

If the doses are determined to be tolerable in the first 9 evaluable participants in either or both regimens based on the observed DLT rate (<33%) and evaluation of the overall toxicity profile, the SLI will be expanded for a total of up to 30 evaluable participants in each cohort. If the DLT rate is ≥33% in either cohort, that combination regimen will not be evaluated further in the SLI and will not be used in the Phase 3.

Table 3 summarizes the probability of observing at least one instance of a toxicity that has a true incidence rate of 10% and 15% for the given number of participants.

**Table 3: Probability of Observing ≥ 1 Instance of Toxicity at 10% and 15% Incidence Rate**

| True Toxicity Incidence Rate | Number of Participants | Probability of Observing ≥1 Instance of Toxicity |
|---|---|---|
| 10% | 15 | 0.79 |
| | 20 | 0.88 |
| | 30 | 0.96 |
| 15% | 15 | 0.91 |
| | 20 | 0.96 |
| | 30 | 0.99 |

If any participant is deemed non-evaluable for DLT, additional participants may be enrolled to ensure there are a sufficient number of evaluable participants in the SLI.

### 5.1.2.2. Phase 3

A graphical multiple testing strategy[1] will be used to strongly control the family-wise error rate of the study at 0.025 (1-sided) level. Initially the type I error rate will be equally split between the two null hypotheses defined by the primary objectives, ie, an alpha of 0.0125 (1-sided) will be assigned to test Arm A vs. Control PFS, and an alpha of 0.0125 (1-sided) to test Arm B vs. Control PFS. The study will be considered positive (ie, demonstrated evidence of effectiveness) if at least one of the hypothesis tests of the primary endpoint is statistically significant at the pre-specified alpha level.

An interim non-binding futility analysis will be performed on both primary endpoint comparisons once there is at least 157 PFS events in Arm A + the Control Arm (ie, approximately 40% information) and at least 157 PFS events in Arm B + the Control Arm (ie, approximately 50% information). Details of the PFS interim futility analysis are described in Section 7.2.1.

If either Arm A or Arm B is stopped for futility at the interim futility analysis, the study will continue with the remaining experimental arm for the primary analysis of PFS.

090177e194e374fe\Approved\Approved On: 10-Sep-2020 19:24 (GMT)

If neither Arm A nor Arm B are stopped for futility, the testing of PFS will be performed in parallel between Arm A vs. the Control Arm, and between Arm B vs. the Control Arm at the time of the primary analysis.

If a hypothesis test of the primary endpoint is found to be statistically significant at 0.0125 (1 -sided) alpha, the corresponding OS comparison will be tested in an interim analysis conducted at the time of the PFS primary analysis. Details of the OS interim efficacy analysis are described in Section 7.2.2. The subsequent steps of the graphical multiple testing strategy are outlined in Figure 2.

If only one of the hypothesis tests of the key secondary endpoint exceeds the superiority boundary at the interim analysis, the final analysis of the remaining OS comparison will be conducted when the number of required events occur for that comparison.

If neither regimen in the SLI is considered tolerable and/or suitable for evaluation in the randomization portion of the trial, the study will proceed as a 2-arm study of Arm A vs. the Control Arm. In this instance, all alpha (ie, 1-sided alpha=0.025) will be assigned to the PFS comparison of Arm A vs. the Control Arm. If PFS is found to be statistically significant, OS of Arm A vs. the Control Arm will then be formally tested at the same alpha level.

The overall significance level is 0.025 (1-sided) and all statistical testing will be performed as 1-sided. No formal statistical testing will be performed on the secondary and exploratory endpoints (with the exception of the OS described above), sensitivity analyses and subgroup analyses. These results will be reported with descriptive statistics only.

Confidence intervals will be 2-sided with a confidence level of 95%, if not otherwise specified.

090177e194e374fe\Approved\Approved On: 10-Sep-2020 19:24 (GMT)

**Figure 2: Graphical Gatekeeping Multiple Testing Strategy**

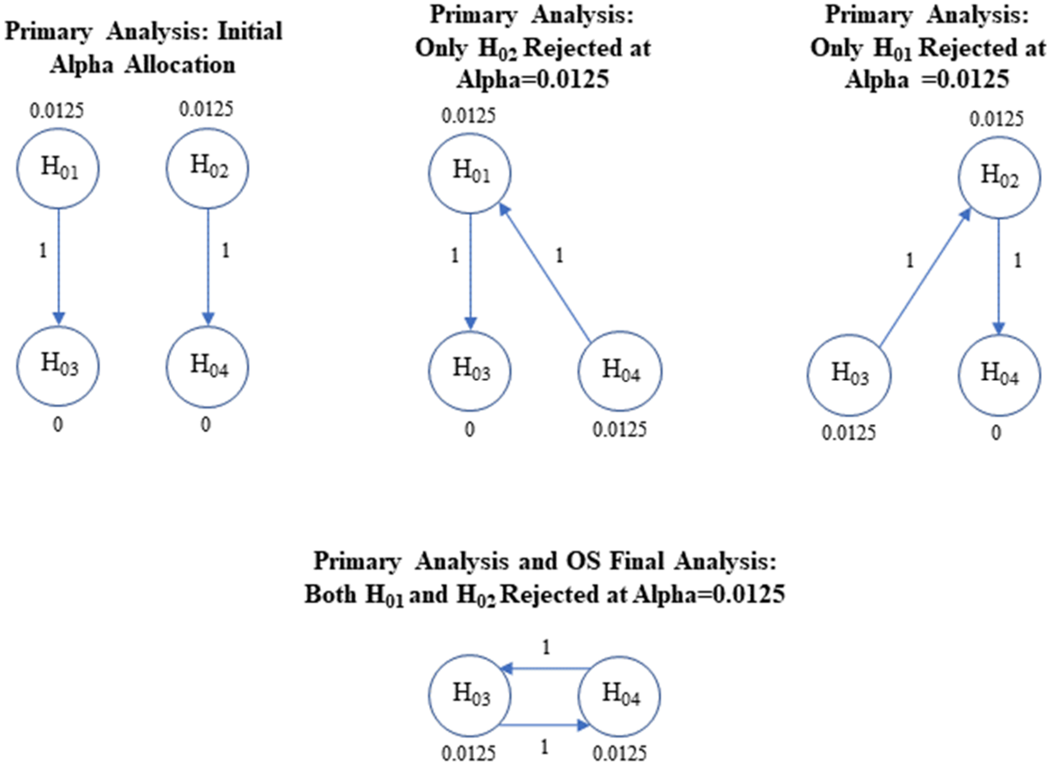


$H_{01}$: $HR_{PFS\ (A\ vs.\ Control)} \geq 1$; $H_{02}$: $HR_{PFS\ (B\ vs.\ Control)} \geq 1$; $H_{03}$: $HR_{OS\ (A\ vs.\ Control)} \geq 1$; $H_{04}$: $HR_{OS\ (B\ vs.\ Control)} \geq 1$

Values above and below the hypotheses (circles) represent the amount of alpha allocated to the given hypothesis at the start of the step. Numbers next to the arrows represent the proportion of the alpha that is propagated from the parent hypothesis to the descendant hypothesis when the parent null hypothesis is rejected.

If an OS null hypothesis (ie, $H_{03}$ or $H_{04}$) is rejected at the OS Final Analysis, no alpha can be propagated back to a PFS hypothesis as PFS can only be tested at the Primary Analysis.

## 5.2. General Methods

Unless otherwise specified, all analyses will be performed separately for the SLI and for the Phase 3 portion of the study.

In general, descriptive summaries will be presented for the efficacy and safety variables collected. Continuous variables will be summarized using mean, standard deviation, minimum, median, and maximum. Categorical variables will be summarized using frequency counts and percentages.

Unless otherwise specified, the calculation of proportions will be based on the sample size of the population of interest. Counts of missing observations will be included in the denominator and presented as a separate category.

090177e194e374fe\Approved\Approved On: 10-Sep-2020 19:24 (GMT)

All statistical analyses will be conducted using SAS®, version 9.4 or higher (SAS Institute, Inc.). Noncompartmental PK analyses will be performed using an internally validated software system, oNCA (version 2.1). Sample size calculation was performed with East version 6.5 (Cytel).[4]

### 5.2.1. Data Handling After the Cut-off Date

Data after the cut-off date may not undergo the cleaning process and will not be displayed in any listings or used for summary statistics, statistical analyses or imputations.

### 5.2.2. Pooling of Data by Center

In order to provide overall estimates of treatment effects, data will be pooled across trial centers. The "center" factor will not be considered in statistical models or for subgroup analyses due to the high number of participating centers in contrast to the anticipated small number of participants randomized at each center.

### 5.2.3. Analyses to Assess the Impact of COVID-19 Pandemic

The study enrollment may start during the COVID-19 pandemic period. If so, the following data summaries and analyses may be performed to assess the impact of COVID-19 on the trial population and study data. Additional analyses may be added in a SAP amendment if they are considered necessary to evaluate the outcome of the trial. Details of these summaries and analyses are included in the respective sections.

- A listing of all participants affected by COVID-19 related study disruption.
- A listing of protocol deviations related to COVID-19.
- COVID-19 related AEs and deaths.
- Summary of missing tumor assessments due to COVID-19.

### 5.2.4. COVID-19 Anchor Date

If additional analyses are needed to assess the impact of COVID-19 on the trial population and the study data, an anchor date will be used as a start date for COVID-19 pandemic related periods based on Pfizer guidance and standard operating procesure (SOP):

- For global pandemic reference date: Use the date the World Health Organization designated COVID-19 as a global pandemic - March 11, 2020.
- For China reference date: Use the date COVID-19 was identified as the causative agent of outbreak in Wuhan by the China Center for Disease Control and Prevention - January 9, 2020.

When producing data summaries intended to show the potential impacts of COVID-19 on the study, data will be presented as "before" and "during," where the anchor date is included in the "during" group.



090177e194e374fe\Approved\Approved On: 10-Sep-2020 19:24 (GMT)

A different anchor date may be used for purposes of regulatory submission should the regulatory authority requests.

### 5.2.5. Definition of Start Date

The start date is defined as date of the first dose of any study drug for the SLI and date of randomization for the Phase 3 portion of the study.

### 5.2.6. Definition of Study Day

The study day for assessments occurring on or after the first dose of study treatment (eg, adverse event onset, laboratory date) will be calculated as:

Study day = Date of the assessment/event - start date of study treatment +1.

The study day for assessments occurring prior to the first dose of study treatment (eg, baseline characteristics, medical history) will be negative and calculated as:

Study day = Date of the assessment/event -start date of study treatment.

For efficacy endpoints and for tumor assessments in the Phase 3 portion the study day will be calculated respect to the randomization date and not to the date of first dose of study treatment.

The study day will be displayed in all relevant data listings.

### 5.2.7. On-treatment Period

The on-treatment period is defined as the time from the first dose of study treatment through minimum of (last dose of study treatment +28 days, start day of subsequent anticancer therapy - 1 day).

### 5.2.8. Date of Last Contact

The date of last contact will be derived for participants not known to have died at the data cutoff date using the latest complete date (ie, imputed dates will not be used in the derivation) among the following:

- All participant assessment dates (eg, blood draws (laboratory, Pharmacokinetics (PK)), vital signs, performance status, ECG, , tumor assessments, hemodynamic assessment);
- Start and stop dates of concomitant therapies including non-drug treatments or procedures;
- Completion dates for PRO Questionnaires;
- Start and end dates of anticancer therapies administered after study treatment discontinuation including systemic therapy, radiation, and surgeries;
- AE start and end dates;



090177e194e374fe\Approved\Approved On: 10-Sep-2020 19:24 (GMT)

- Last date of contact collected on the 'Survival Follow-up' eCRF (do not use date of survival follow-up assessment unless status is 'alive');
- Study treatment start and end dates;
- Randomization date; and
- Date of discontinuation on disposition eCRF pages (do not use if reason for discontinuation is lost to follow-up or death).

Only dates associated with actual examinations of the participant will be used in the derivation. Dates associated with a technical operation unrelated to participant status such as the date a blood sample was processed or dates data were entered into the eCRF will not be used. Assessment dates after the data cutoff date will not be applied to derive the last contact date.

### 5.2.9. Unscheduled Assessments

Unless otherwise specified, unscheduled assessments will not be displayed in summary tables by nominal visit/timepoint. Unscheduled assessments will be used when deriving baseline and worst case on-treatment for safety and PRO analyses (except where noted for baseline ECGs). Additionally, unscheduled tumor assessments will be used for efficacy analyses (eg, defining date of progression/censoring, best overall response, date of last contact).

### 5.2.10. Tumor Assessment Date

If there are multiple scan dates associated with a tumor evaluation, ie, radiological assessments occur over a series of days rather than the same day, the earliest scan date associated with the evaluation will be used as the date of the assessment. The same convention should be used for both investigator and BICR assessments, noting however that in the event that the BICR provides the date of PD, that date should be used instead of deriving the date of PD.

### 5.2.11. Adequate Baseline Tumor Assessment

An adequate baseline tumor assessment is defined as follows:

- Baseline assessments of all lesions (target, and non-target) must be within 28 days prior to first dose date (SLI) or date of randomization (Phase 3).

  **Note**: If participants cannot visit the site for baseline assessment per Schedule of Activities in Section 1.3 of the protocol due to COVID-19 pandemic, a visit windowing of 6 days will be added for baseline assessments (28 days +2 times the on-study tumor assessment window of 3 days), ie, the baseline assessments for all lesions must be within 34 days prior to the first dose date (SLI) or date of randomization (Phase 3).

090177e194e374fe\Approved\Approved On: 10-Sep-2020 19:24 (GMT)

- All lesions (target and non-target) must have non-missing assessments.

  **Note**: for target lesions, an actual measurement should be recorded and it should meet the criteria for being measurable and for non-target lesions the actual status at baseline should indicate that the lesion was present).

### 5.2.12. Adequate Post-baseline Tumor Assessment

An adequate post-baseline tumor assessment is defined as an assessment where a response of CR, PR, SD, non-CR/non-PD, or PD can be determined. Time points where the response is NE or no assessment was performed will not be used for determining the censoring date.

### 5.2.13. Standard Derivations and Reporting Conventions

The following conversion factors will be used to convert days into weeks, months or years:
1 week = 7 days, 1 month = 30.4375 days, 1 year = 365.25 days.

Percentages will be reported to one decimal place. The rounding will be performed to closest integer/first decimal using the common mid-point between the two consecutive values. Eg, 5.1 to 5.4 will be rounded to an integer of 5, and 5.5 to 5.9 will be rounded to an integer of 6.

### 5.2.14. Analyses for Binary and Categorical Endpoints

Binary and categorical endpoints will be summarized by frequency counts and percentages along with corresponding exact 2-sided Clopper-Pearson 95% CI. Unless otherwise specified, the calculation of proportions will include the missing category. Therefore counts of missing observations will be included in the denominator and presented as a separate category.

In case the analysis refers only to certain visits, percentages will be based on the number of participants with an assessment at that visit, unless otherwise specified.

### 5.2.15. Analyses for Continuous Endpoints

Continuous endpoints will be summarized using descriptive statistics ie, number of non-missing values and number of missing values [ie, n (missing)], mean, median, standard deviation, minimum, maximum and first and third quartile (Q1 and Q3). PK summaries will also include coefficient of variation percent (%CV).

In case the analysis refers only to certain visits, percentages will be based on the number of participants with an assessment at that visit, unless otherwise specified.

### 5.2.16. Analyses for Time-to-Event Endpoints

The stratified log-rank test will be used for comparing treatments. Hazard ratios and the associated 2-sided confidence intervals (CIs) are estimated by stratified Cox proportional hazards model, including treatment as a covariate. To account for the required alpha spending of the group sequential design, 2-sided repeated CIs[2] will also be provided.



090177e194e374fe\Approved\Approved On: 10-Sep-2020 19:24 (GMT)

Time to event endpoints will be summarized using the Kaplan-Meier method and estimated survival curves will be displayed graphically when appropriate. Graphs will describe the number of participants at risk over time. The median, quartiles, and probabilities of an event at particular points in time will be estimated by the Kaplan-Meier method. Confidence intervals for medians and quartiles are based on the Brookmeyer-Crowley[3] method. Confidence intervals for the estimated probability of event at a particular timepoint will be generated using the log(-log) method with back transformation to a confidence interval on the untransformed scale. Summaries of the number and percentages of participants with an event and reason for censoring will also be provided on summary tables and/or figures.

**Assessment of proportional hazards**

Schoenfeld residuals for the stratified Cox proportional regression model may be plotted to investigate graphically violations from the proportional hazards (PH) assumption; a non-zero slope is evidence of departure from PH. The PH assumption may be formally tested using Schoenfeld's residual test (Schoenfeld, 1980;[5] Therneau & Grambsch, 2000).[6] Large departures from PH will be evidenced by a p-value <0.05. Note however that the test will not be sensitive to detect non-linear deviations from PH.

In addition, the PH assumption will be checked visually within each stratum by plotting

$$\log(-\log(S(t))) \text{ vs. } \log(t) ,$$

where S(t) is the estimated survival function at time t.

**Restricted Mean Survival Time**

The restricted mean survival time (RMST) methodology (Zhang, 2013)[7] is applicable independently of the PH assumption and can be used, at a minimum, as a sensitivity analysis to explore the robustness of the primary analysis result on time-to event endpoint.

In particular, as it pertains to the **cut-off point (τ)** to evaluate the RMST it is noted that the cut-off point should not exceed the minimum of the largest observed time for both treatment arms so that the RMST of all treatment arms being evaluated can be adequately estimated and comparison between treatments is feasible; τ should be clinically meaningful and closer to the end of the study follow-up so that the majority of survival outcomes will be covered by the time interval. The RMST up to time τ can be interpreted as the expected survival time restricted to the common follow-up time τ among all participants. Analyses will be repeated using the following criteria to define τ:

- $\tau_1$ = minimum of (largest observed survival time for experimental arm, largest observed survival time for control arm);
- $\tau_2$ = minimum of (largest survival event time for experimental arm, largest survival event time for control arm);



090177e194e374fe\Approved\Approved On: 10-Sep-2020 19:24 (GMT)

- $\tau 3$ = midpoint between $\tau 1$ and $\tau 2$.

RMST can be estimated consistently by the area under the Kaplan-Meier curve over $[0, \tau]$.

The treatment effect between the experimental arm and the control arm will be assessed based on the difference in RMST. The associated 95% CI for the difference in means and 1-sided p-value will be generated. RMST as a function of $\tau$ and the associated treatment effect between the experimental arm and the control arm will be plotted against time $\tau$.

## 5.3. Methods to Manage Missing Data

Unless otherwise specified, all data will be evaluated as observed, and no imputation method for missing values will be used.

Any imputations will occur at the analysis dataset level. Additionally, in all participant data listings imputed values will be presented and flagged as imputed.

Missing statistics, eg, when they cannot be calculated, should be presented as 'ND' for not done, 'NR' for not reached or 'NA' for not applicable. For example, if N=1, the measure of variability cannot be computed and should be presented as 'ND' or 'NA'.

### 5.3.1. Missing Dates

For purposes of data listings, dates will reflect only the information provided by the investigator on the eCRF.

If start dates for adverse events or concomitant medications are completely missing a worst case approach will be taken whereby the events will be considered treatment emergent and the medications will be considered concomitant. If only partial information are available (eg, only a month and year or only a year) and the partial information provide sufficient information to indicate the dates are prior to the start of study treatment (eg, month/year less than month/year of first dose) then these will be considered to have started prior to treatment; otherwise a similar worst case approach will apply and these will be considered to have started after treatment.

**<u>Missing or Partial Death Dates</u>**

Missing or partial death dates will be imputed based on the last contact date:

- If the entire date is missing it will be imputed as the day after the date of last contact (see derivation of date of last contact in Section 5.2.8).
- If the day or month is missing, death will be imputed to the maximum of the full (non-imputed) day after the date of last contact and the following:
  - Missing day: 1st day of the month and year of death, or
  - Missing day and month: January 1st of the year of death.



090177e194e374fe\Approved\Approved On: 10-Sep-2020 19:24 (GMT)

<u>**Date of First and Last Dose of Study Treatment**</u>

No imputation will be done for first dose date.

Date of last dose of study drug, if unknown or partially unknown, will be imputed as follows:

- If the last date of study drug is completely missing and there is no End of Treatment eCRF page and no death date, the participant should be considered to be ongoing and use the cutoff date for the analysis as the last dosing date. **Note:** the study team should confirm that the participant is actively receiving dose at the time of the data cutoff.
- If the last date of study drug is completely or partially missing and there is EITHER an End of Treatment eCRF page OR a death date available (within the data cutoff date), then impute as follows:
    - If only Year is available and Year < Year of min (EOT date, death date), impute the date as 31DECYYYY.
    - If both Year and Month are available and Year = Year of min (EOT date, death date) and Month < Month of min (EOT date, death date), impute as last day of the month.
    - For all other cases, impute as min (EOT date, death date).

<u>**Date of Start of New Anticancer Therapy**</u>

Incomplete dates for start date of new anticancer therapy will be imputed as follows and will be used for determining censoring dates for efficacy analyses. PD date below refers to PD date by investigator assessment. If the imputation results in an end date prior to the imputed start date then the imputed start date should be set to the end date.

- The end date of new anticancer therapy will be included in the imputations for start date of new anticancer therapy. If the end date of new anticancer therapy is:
    - completely missing then it will be ignored in the imputations below;
    - partially missing with only year (YYYY) available then the imputations below will consider 31DECYYYY as the end date of the new anticancer therapy;
    - partially missing with only month and year available then the imputations below will consider the last day of the month for MMMYYYY as the end date of the new anticancer therapy.
- For participants who have not discontinued study treatment at the analysis cut-off date, last dose of study treatment is set to the analysis cut-off date in the imputations below.

090177e194e374fe\Approved\Approved On: 10-Sep-2020 19:24 (GMT)

- If the start date of new anticancer therapy is completely or partially missing then the imputed start date of new anticancer therapy is derived as follows:
  - Start date of new anticancer therapy is completely missing:

    Imputed start date = min [max(PD date +1, last dose of study treatment +1), end date of new anticancer therapy]
  - Only year (YYYY) for start of anticancer therapy is available:

    IF YYYY < Year of min [max(PD date +1, last dose of study treatment +1), end date of new anticancer therapy] THEN imputed start date = 31DECYYYY;

    ELSE IF YYYY = Year of min [max(PD date +1, last dose of study treatment +1), end date of new anticancer therapy]

    THEN imputed start date = min [max(PD date +1, last dose of study treatment +1), end date of new anticancer therapy]

    ELSE IF YYYY > Year of min [max(PD date +1, last dose of study treatment +1), end date of new anticancer therapy]

    THEN imputed start date = 01JANYYYY
  - Both Year (YYYY) and Month (MMM) for start of anticancer therapy are available

    IF

    YYYY = Year of min [max(PD date +1, last dose of study treatment +1), end date of new anticancer therapy], AND

    MMM < Month of min [max(PD date +1 day, last dose of study treatment +1 day), end date of new anticancer therapy]

    THEN

    imputed start date = DAY (Last day of MMM) MMM YYYY ;

    ELSE IF

    YYYY = Year of min [max(PD date +1, last dose of study treatment +1), end date of new anticancer therapy], AND

    MMM = Month of min [max(PD date +1 day, last dose of study treatment +1 day), end date of new anticancer therapy]

    THEN

    imputed start date = min [max(PD date +1 day, last dose of study treatment +1 day), end date of new anticancer therapy]);

    ELSE IF

090177e194e374fe\Approved\Approved On: 10-Sep-2020 19:24 (GMT)

YYYY = Year of min [max(PD date +1, last dose of study treatment +1), end date of new anticancer therapy], AND

MMM > Month of min [max(PD date +1 day, last dose of study treatment +1 day), end date of new anticancer therapy]

THEN

imputed start date = 01 MMM YYYY;

ELSE IF

YYYY < Year of min [max(PD date +1, last dose of study treatment +1), end date of new anticancer therapy]

THEN

imputed start date = DAY (Last day of MMM) MMM YYYY;

ELSE IF

YYYY > Year of min [max(PD date +1, last dose of study treatment +1), end date of new anticancer therapy]

THEN

imputed start date = 01 MMM YYYY.

**AE Dates**

AE Onset Date:

If completely missing, the onset date will be set to first dose date if the first dose date is less than AE stop date. Otherwise if the first dose date is after AE stop date, then set the onset date to the earliest of non-missing AE stop date or informed consent date.

AE Stop Date:

If completely missing, the stop date will be imputed as the latest of the end of study date, death date, last dose date of the study treatment, or onset date.

Partial AE Date:

Partial AE date will be imputed bases on the imputation rule for "Other Missing or Partial Dates". If the AE onset date is imputed from a partial AE date and the first dose date falls in the same month as the AE onset date, the following will be done:

- The AE onset date is reset to the first dose date.

If AE stop date is imputed, and less than the first dose date, set the AE stop date to the first dose date.

090177e194e374fe\Approved\Approved On: 10-Sep-2020 19:24 (GMT)

090177e194e374fe\Approved\Approved On: 10-Sep-2020 19:24 (GMT)

**Partial Date of Progression after Subsequent Therapy**

Partial date of progression after subsequent therapy will be imputed as follows:

- If the entire date is missing or if only year is provided, no imputation will be performed.
- If month and year are provided and only the day is missing, the date will be imputed as the 15th of the month.

**Other Missing or Partial Dates**

Imputation methods for other partial dates are as follows:

- If the day of the month is missing for a start date used in a calculation, the 1st of the month will be used to replace the missing date.
- If both the day and month are missing, the first day of the year is used.
- For stop dates, the last day of the month, or last day of the year is used if the day or day and month are missing, respectively.

These rules are used unless the calculations result in negative time durations (eg, date of resolution cannot be prior to date of onset). In these case, the resolution and onset dates will be the same and the duration will be set to 1 day.

### 5.3.2. Missing Patient-Reported Outcomes Data

For the EORTC QLQ-C30 and EQ-5D-5L, missing items will be handled per the respective scoring manuals of each questionnaire. For the PGIS and PGIC, there will be no adjustments for missing data.

### 5.3.3. Missing Pharmacokinetic Data

**Deviations, Missing Concentrations and Anomalous Values**

In summary tables and plots of geometric mean profiles, concentrations will be set to missing if one of the following cases is true:

- A concentration has been reported as ND (ie, not done) or NS (ie, no sample);
- A deviation in sampling time is of sufficient concern or a concentration has been flagged as anomalous by the clinical pharmacologist.

Summary statistics will not be presented at a particular timepoint if more than 50% of the data are missing. For analysis of pharmacokinetic concentrations, no values will be imputed for missing data.

### 5.3.4. Missing Biomarker Data

No missing data will be imputed.

# 6. ANALYSES AND SUMMARIES

All efficacy analyses will be performed by cohort on the safety analysis set for the SLI, and by treatment arm on the FAS for the Phase 3.

## 6.1. Primary Endpoints

### 6.1.1. Main Analysis

**Safety Lead-in:**

The number and proportion of participants experiencing DLTs during the DLT-evaluation period will be summarized by System Organ Class (SOC) and preferred term (PT) based on maximum toxicity grade by treatment cohort. Analyses of DLT will be performed on DLT-Evaluable Set.

All DLTs and their attributes will be presented in data listings sorted by participant identifier, AE and date of onset of the AE.

**Phase 3:**

The primary efficacy analysis will compare PFS time by BICR between Arm A and the Control Arm, and between Arm B and the Control Arm using a 1-sided stratified log-rank test at the significance level based on the graphical multiple testing strategy as outlined in Section 5.1.2.2. The analysis will be performed based on the FAS. Strata will be based on those specified in the IRT system as described in Section 3.3.1.

PFS will be calculated in months as follows:

PFS (months) = (date of event or censoring – date of randomization +1)/30.4375

Participants without an event or with an event more than 12 weeks (for the first 18 months after randomization) or 16 weeks (after the first 18 months of randomization) after the last adequate tumor assessment that documented no progression will be censored on the date of the last adequate tumor assessment that documented no progression. In addition, if a new anticancer therapy is started or a metastasectomy procedure is performed prior to an event, the participant will be censored on the date of the last adequate tumor assessment that documented no progression prior to the start of the new anticancer therapy or undergoing metastasectomy procedures.

Adequate baseline assessment and adequate post-baseline assessment are defined in Section 5.2.11 and Section 5.2.12, respectively.

090177e194e374fe\Approved\Approved On: 10-Sep-2020 19:24 (GMT)

090177e194e374fe\Approved\Approved On: 10-Sep-2020 19:24 (GMT)

Participants with no baseline tumor assessment (including participants with an inadequate baseline assessment) or with no adequate post-baseline tumor assessments within 12 weeks after the date of randomization will be censored on the day of randomization, unless the participant dies within 12 weeks of randomization,  in which case, death will be an event on date of death.

The censoring and event date options to be considered for the PFS primary analysis are presented in Table 4.

**Table 4: PFS Outcome and Event Dates – Primary Analysis**

| Situation | Date of Progression/Censoring | Outcome |
|---|---|---|
| No adequate baseline assessment | Date of randomization[a] | Censored[a] |
| PD or death ≤12 (or 16)[b] weeks after last adequate tumor assessment or ≤12 weeks after date of randomization | Date of PD or death | Event |
| PD or death >12 (or 16)[b] weeks after the last adequate tumor assessment[c] | Date of last adequate tumor assessment [c] documenting no PD prior to new anticancer therapy, metastasectomy procedures or missed assessments | Censored |
| No PD | | |
| New anticancer therapy given or undergoing metastasectomy procedures | | |

[a] If the participant dies ≤12 weeks after date of randomization, the death is an event with date on death date.

[b] Durations are equal to 2 times the length of the tumor assessment interval, which is 12 weeks for the first 18 months after randomization, and 16 weeks thereafter.

[c] If there are no adequate post-baseline assessments prior to the PD or death, then the time without adequate assessment should be measured from the date of randomization; if the criteria is met, the censoring will be on the date of randomization.

The treatment effect will be estimated using a Cox's proportional hazards model stratified by randomization strata to calculate the HR for PFS of Arm A vs. Control Arm, and of Arm B vs. Control Arm, along with the corresponding 2-sided CI. The confidence level will match the alpha tested (eg, 97.5% CI when PFS is tested at 1-sided 0.0125). In addition, the 2-sided 95% CIs for the HR will also be reported.

Kaplan-Meier estimates (product-limit estimates) will be presented by treatment arm together with a summary of associated statistics including the median PFS time with 2-sided 95% confidence intervals (CI).  The PFS rate at 3, 6, 9, 12, 15, 18 and 21 months will be estimated with corresponding 2-sided 95% CIs.  The CIs for the median will be calculated according to Brookmeyer and Crowley[3] (conftype=linear option in SAS PROC LIFETEST) and the CIs for the survival function estimates at the timepoints defined above will be derived using the log(-log) method according to Kalbfleisch and Prentice[9] (conftype=loglog default option in SAS Proc LIFETEST). The estimate of the standard error will be computed using Greenwood's formula.

Frequency (number and percentage) of participants with each event type (PD or death) and censoring reasons will be presented by treatment arm along with the overall event and censor rates.

Reasons for censoring will be summarized according to the categories in Table 5. If a participant meets multiple definitions for censoring the list will be used to define the hierarchy.

**Table 5: PFS Censoring Reasons and Hierarchy - Primary Analysis**

| Hierarchy | Condition | Censoring Reason |
|---|---|---|
| 1 | No adequate baseline assessment | No adequate baseline assessment |
| 2 | Start of new anticancer therapy before event or undergo metastasectomy procedures before event | Start of new anticancer therapy/Undergoing metastasectomy procedures |
| 3 | Event >12 (or 16)[a] weeks from last adequate post-baseline tumor assessment/start date | Event after missing assessments or inadequate assessments[a] |
| 4 | No event and [withdrawal of consent date ≥ date of randomization OR End of study (EOS) = Subject refused further follow-up] | Withdrawal of consent |
| 5 | No event and lost to follow-up in any disposition page | Lost to follow-up |
| 6 | No event and [EOS present OR disposition page for any phase after screening says participant will not continue into any subsequent phase of the study] and no adequate post-baseline tumor assessment | No adequate post-baseline tumor assessment |
| 7 | No event and none of the conditions in the prior hierarchy are met | Ongoing without an event |

[a] Durations are equal to 2 times the length of the tumor assessment interval, which is 12 weeks for the first 18 months after date of first dose (SLI) or date of randomization (Phase 3), and 16 weeks thereafter.

The PFS time or censoring time and the reasons for censoring will also be presented in a patient listing.

Schoenfeld residuals for the stratified Cox proportional model may be plotted to investigate graphically violations from the proportional hazards (PH) assumption (see Section 5.2.16 for details).

090177e194e374fe\Approved\Approved On: 10-Sep-2020 19:24 (GMT)

**Time of Follow-Up for PFS by BICR**

A Kaplan-Meier plot for PFS follow-up duration will also be created to assess the follow-up time in each treatment arm using reversed Kaplan-Meier method as shown in Table 6. The Kaplan-Meier estimate of the median PFS follow-up time with its corresponding 2-sided 95% CI will be calculated and presented in the plot.

**Table 6: Reverse Censoring Rules for PFS Follow-up Duration**

| | Date of event/censoring | Censoring |
|---|---|---|
| Participant alive or lost to follow-up | Time from randomization to last date known to be alive | No |
| Death or PD | Time from randomization to date of first PD or death | Yes |

### 6.1.2. Sensitivity/Supplementary Analyses

The following sensitivity and supplementary analyses will be performed to explore the robustness of the primary analysis results and the supportive estimands. These analyses are regarded as purely exploratory.

**Sensitivity Analyses:**

- An stratified analysis will be performed to compare the PFS time by Investigator assessment.
- An unstratified analysis will be performed to compare the PFS time by BICR.
- PFS by BICR will also be analyzed based on RMST differences (see Section 5.2.16 for details).
- Multivariable Cox regression analysis will be performed to explore the potential influences of baseline participant characteristics on PFS. The baseline characteristics variables specified in Section 3.3.2 might be included as covariates in the Cox regression model in addition to the randomization strata which will be included in the model during the selection procedure.

  A stepwise selection procedure will serve to identify these explanatory variables of potential prognostic values. Variables are entered into and removed from the model in such a way that each forward selection step can be followed by one or more backward elimination steps. The stepwise selection process terminates if no further variable can be added to the model or if the variable just entered into the model is the only variable removed in the subsequent backward elimination. The level of significance for an explanatory variable to enter the model is set to 0.15 (p-value of Score test) and the significance level for removing it is set to 0.40 (p-value of Wald test). This analysis will be performed using the stepwise selection method in SAS (PROC

090177e194e374fe\Approved\Approved On: 10-Sep-2020 19:24 (GMT)

PHREG). Once this procedure stops, the variable for treatment arm will be added to the last selected model in order to evaluate the effect of treatment on PFS time when adjusted for the selected explanatory variables. The HRs of all selected explanatory variables and of treatment effect will be reported including 2-sided 95% CIs. No interactions will be considered.

**<u>Supplementary Analyses:</u>**

- PFS by BICR will be analyzed based on Central *BRAF* V600E Positive analysis set.
- PFS by BICR will be analyzed using the treatment policy strategy (Section 2.1.3). The analysis will use the same methodology and summary as the main analysis but will include observations that occur after the intercurrent events of starting new anticancer therapy or undergoing metastasectomy procedures. All other intercurrent events will be addressed in the same approach as the primary estimand of PFS. Table 7 summarizes the details of the intercurrent events and censoring rules for this supplementary analysis.

**Table 7: PFS Outcome and Event Dates – Supplementary Analysis**

<table>
<tr><th>Situation</th><th>Date of Progression/Censoring</th><th>Outcome</th></tr>
<tr><td>No adequate baseline assessment</td><td>Date of randomization[a]</td><td>Censored[a]</td></tr>
<tr><td>PD or death ≤12 (or 16)[b] weeks after last adequate tumor assessment or ≤12 weeks after date of randomization</td><td>Date of PD or death</td><td>Event</td></tr>
<tr><td>PD or death >12 (or 16)[b] weeks after the last adequate tumor assessment[b]</td><td rowspan="2">Date of last adequate tumor assessment[c] documenting no PD prior to missed assessments</td><td rowspan="2">Censored</td></tr>
<tr><td>No PD</td></tr>
</table>

[a] If the participant dies ≤12 weeks after date of randomization, the death is an event with date on death date.
[b] Durations are equal to 2 times the length of the tumor assessment interval, which is 12 weeks for the first 18 months after randomization, and 16 weeks thereafter.
[c] If there are no adequate post- baseline assessments prior to the PD or death, then the time without adequate assessment should be measured from the date of randomization; if the criteria were met the censoring will be on the date of randomization.

**<u>Discordance between BICR and Investigator Assessment on PFS</u>**

A summary of discordances of events (PD or death) between BIRC and Investigator assessment will be provided as shown in Table 8:

090177e194e374fe\Approved\Approved On: 10-Sep-2020 19:24 (GMT)

**Table 8: Discordance Summary for Investigator vs BICR Assessment on PFS**

| | | BICR | |
|---|---|---|---|
| | | **Event** | **No Event** |
| **Investigator** | **Event** | a = a1 + a2 + a3 | b |
| | **No Event** | c | d |

a1: number of agreements on timing and occurrence of event;
a2: number of times agreement on event but INV declares event later than BICR;
a3: number of times agreement on event but INV declares event earlier than BICR;
N= a+b+c+d.
The timing agreement of event is defined as a window of ±7 days.

The following **measures of discordances** will be calculated:

- Total Event Discrepancy Rate: (b+c)/N.
- Early Discrepancy Rate (EDR): (a3+b)/(a+b).
- Late Discrepancy Rate (LDR): (a2+c)/(a2+a3+b+c).
- Overall Discrepancy Rate: (a2+a3+b+c)/N.

The EDR represents the positive predictive value of Investigator assessment and quantifies the frequency with which the Investigator declares PFS event earlier than BICR within each treatment arm as a proportion of the total number of Investigator assessed PFS events.

The LDR quantifies the frequency with which the Investigator declares PFS event later than BICR as a proportion of the total number of participants with discrepancies within the treatment arm.

Total event discrepancy rate represents the frequency with which the Investigator and BICR disagreed on overall censoring/event status as a proportion of the number of participants within the treatment arm (N).

Overall discrepancy rate represents the frequency with which the Investigator and BICR disagreed on overall censoring/event status or the timing of an event as a proportion of the number of participants within the arm (N).

Discordance metrics will be calculated for each treatment arm and, for each metric, the difference in discordance between Arm A and the Control arm, and between Arm B and the Control Arm is used to evaluate potential bias. If the discordance is similar across the treatment arms then it suggests the absence of evaluation bias favoring a particular arm. A negative differential discordance for EDR and/or a positive differential discordance for LDR may be indicative of Investigator devaluation bias in favor of the experimental arm, ie, Arm A or Arm B (Amit et al, 2011).[8]



090177e194e374fe\Approved\Approved On: 10-Sep-2020 19:24 (GMT)

## 6.2. Efficacy Secondary Endpoints

### 6.2.1. Overall Survival

#### 6.2.1.1. Main Analysis

**Safety Lead-in:**

OS will be calculated in months as follows:

OS (months) = (date of death or censoring – date of first dose +1)/30.4375

Kaplan-Meier estimates will be presented by cohort together with a summary of associated statistics including the median OS time with 2-sided 95% CI. The OS rate at 6, 12, 18 and 24 months will be estimated with corresponding 2-sided 95% CIs.

Frequency (number and percentage) of participants with death events and censoring reasons will be presented by cohort along with the overall event and censor rates.

**Phase 3:**

The key secondary efficacy analysis will compare OS time between Arm A and the Control Arm, and between Arm B and the Control Arm using a 1-sided stratified log-rank test at the significance level based on the graphical multiple testing strategy as outlined in Section 5.1.2.2. The analysis will be performed based on the FAS. Strata will be based on those specified in the IRT system as described in Section 3.3.1.

OS will be calculated in months as follows:

OS (months) = (date of death or censoring – date of randomization +1)/30.4375

The treatment effect will be estimated using a Cox's proportional hazards model stratified by randomization strata to calculate the HR for OS of Arm A vs. the Control Arm, and of Arm B vs. the Control Arm.

In order to account for the group sequential design on this endpoint, the repeated CI method[2] will be used to construct the 2-sided repeated CI for the HR at the interim and the final analyses of OS. Based on the Lan-DeMets alpha-spending function[10] that approximates O'Brien-Fleming stopping boundaries, the alpha level will be 0.0028 (1-sided) at the interim analysis, and 0.0116 (1-sided) at the final analysis (Table 15). These alpha values be used to calculate the 2-sided CI for the corresponding analysis. This approach results in a 2-sided 99.1% CI and 97.7% CI at the interim and the final analysis, respectively. The actual alpha spent at the interim and the final analysis will be updated prior to the time of the analysis based on the actual number of events and the information fraction. In addition, the 2-sided 95% CIs for the HR will also be reported.

OS time will be estimated using the same Kaplan-Meier method as described for PFS in Section 6.1.1. The OS rate at 6, 12, 18, 24, 30 and 36 months will be estimated with corresponding 2-sided 95% CIs.



090177e194e374fe\Approved\Approved On: 10-Sep-2020 19:24 (GMT)

Frequency (number and percentage) of participants with death events and censoring reasons will be presented by treatment arm along with the overall event and censor rates. The event and ensoring reasons are as follows:

- Death:
    - Death due to COVID-19;
- Ongoing and no death;
- Withdrawal of consent;
- Lost to follow-up.

The OS time or censoring time and the reasons for censoring will also be presented in a patient listing.

Schoenfeld residuals for the stratified Cox proportional model may be plotted to investigate graphically violations from the proportional hazards (PH) assumption (see Section 5.2.16 for details).

**Time of Follow-Up for OS in Phase 3**

A Kaplan-Meier plot for OS follow-up duration will also be created to assess the follow-up time in each treatment arm using the reversed Kaplan-Meier method as shown in Table 9. The Kaplan-Meier estimate of the median OS follow-up time with its corresponding 2-sided 95% CI will be calculated and presented in the plot.

**Table 9: Reverse Censoring Rules for OS Follow-up Duration**

| | Date of event / censoring | Censoring |
|---|---|---|
| Participant alive or lost to follow-up | Time from randomization to last date known to be alive | No |
| Participant died | Time from randomization to date of death | Yes |

### 6.2.1.2. Sensitivity/Supplementary Analysis

The following sensitivity and supplementary analyses will be performed to explore the robustness of the key secondary endpoint analysis results and the supportive estimands in the Phase 3 portion of the study. These analyses are regarded as purely exploratory.

**Sensitivity Analyses:**

- An unstratified analysis will be performed to compare the OS time.
- OS will also be analyzed based on RMST differences (see Section 5.2.16 for details).



090177e194e374fe\Approved\Approved On: 10-Sep-2020 19:24 (GMT)

- The same multivariable Cox regression analysis that is performed on PFS as desccribed in Section 6.1.2 will also be conducted on OS to assess and adjust the treatment effect for relevant baseline factors of potential prognostic impact.

**Supplementary Analyses:**

- OS will be analyzed based on Central *BRAF* V600E Positive analysis set.

#### 6.2.2. Objective Response Rate

ORR will be calculated by dividing the number of participants with BOR of CR or PR per RECIST v1.1 by the number of participants dosed in each cohort (SLI) or randomized to each treatment arm (Phase 3).

BOR is the best response obtained among all tumor assessment visits after the study start date until documented disease progression, death, start of subsequent anticancer therapy or undergoing metastasectomy procedures. The study start date is date of the first dose for the SLI and date of randomization for the Phase 3 as defined in Section 5.2.5.

Two sets of BOR will be summarized, one based on confirmed responses and one based on confirmed + unconfirmed responses. For BOR based on confirmed responses, the following rules will be applied for the confirmation:

- CR = at least two determinations of CR at least 4 weeks apart and documented before progression, start of new anticancer therapy or undergoing metastasectomy procedures.
- PR = at least two determinations of PR or better (and not qualifying for a CR) at least 4 weeks apart and before progression, start of new anticancer or undergoing metastasectomy procedures.
- SD (for participants with at least one measurable lesion at baseline)= at least one SD assessment (or better and not qualifying for CR or PR) ≥6 weeks after the study start date and before progression, start of new anticancer therapy or undergoing metastasectomy procedures.
- Non-CR/Non-PD (for participants with only non-target disease at baseline) = at least one Non-CR/Non-PD assessment (or better and not qualifying for CR or PR) ≥6 weeks after the study start date and before progression, the start of new anticancer therapy or undergoing metastasectomy procedures.
- PD = progression ≤12 weeks after the study start date not qualifying for CR, PR or SD.
- Not Evaluable (NE) = all other cases.

Clinical deterioration or clinical progression noted on the completion eCRF will not be considered as documented disease progression.

090177e194e374fe\Approved\Approved On: 10-Sep-2020 19:24 (GMT)

The frequency (number and percentage) of participants with BOR of CR, PR, SD, PD, non-CR/non-PD (applicable only to participants with non-measurable disease at baseline), and NE will be tabulated. The ORR will be calculated along with corresponding exact 2-sided Clopper-Pearson 95% CI.

Participants with a BOR of "Not Evaluable" based on confirmed responses will be summarized by reason for having unknown status. The following reasons will be used:

- No adequate baseline assessment.
- No evidence of disease at baseline.
- No post-baseline assessments due to early death (ie, death ≤12 weeks after the start date).
- No post-baseline assessments due to COVID-19 (ie, participants miss tumor assessment visits due to COVID-19 pandemic).
- No post-baseline assessments due to other reasons.
- All post-baseline assessments have overall response NE.
- New anticancer therapy started or undergoing metastasectomy procedures before first post-baseline assessment.
- SD of insufficient duration (<6 weeks after the start date without further evaluable tumor assessments).
- PD too late (>12 weeks after the start date, ie, tumor assessment of PD was >12 weeks after start of treatment and there was no tumor assessment in between).

Special and rare cases where BOR is NE due to both SD of insufficient duration ('too early') and late PD will be classified as "SD of insufficient duration".

For the SLI, ORR based on Investigator assessment will be calculated. For the Phase 3, both ORR by BICR and ORR by Investigator will be calculated. For the Phase 3, a summary of concordance of BOR between BICR and Investigator assessment will also be provided.

### 6.2.3. Duration of Response

DOR will be calculated for participants with a confirmed response (CR or PR) as follows:

DOR (months) = (date of event or censoring – date of first CR or PR + 1)/30.4375

The same censoring rules specified for PFS in Section 6.1.1 apply to DOR. DOR will be estimated using the same Kaplan-Meier method as described for PFS. DOR rates at 3, 6, 9, 12 and 15 months will be estimated with corresponding 2-sided 95% CIs.



090177e194e374fe\Approved\Approved On: 10-Sep-2020 19:24 (GMT)

In addition, a plot of time to and duration of response for participants with a confirmed response (swimmer plot) will be created.

For the SLI, DOR based on Investigator assessment will be calculated. For the Phase 3, both DOR by BICR and DOR by Investigator will be calculated.

### 6.2.4. Time to Response

TTR will be calculated for participants with a confirmed response (CR or PR) in weeks as follows:

TTR (in weeks) = [data of first CR or PR – date of first dose (SLI)/date of randomization (Phase 3) +1]/7

TTR will be summarized using simple descriptive statistics (mean, standard deviation, median, minimum, maximum, first quartile, third quartile).

For the SLI, TTR based on Investigator assessment will be calculated. For the Phase 3, both TTR by BICR and TTR by Investigator will be calculated.

### 6.2.5. Progression-free Survival (Safety Lead-in)

For the SLI, PFS will be calculated in months as follows:

PFS (months) = (date of event or censoring – date of first dose +1)/30.4375

The same censoring rules specified for PFS in the Phase 3 portion in in Section 6.1.1 apply to PFS in the SLI, with the date of randomization replaced by the date of first dose in the SLI. Kaplan-Meier estimates will be presented by cohort together with a summary of associated statistics including the median PFS time with 2-sided 95% CI. The PFS rate at 3, 6, 9, 12, 15, 18 and 21 months will be estimated with corresponding 2-sided 95% CIs.

Frequency (number and percentage) of participants with each event type (PD or death) and censoring reasons will be presented by cohort along with the overall event and censor rates. Reasons for censoring will be summarized according to the categories in Table 4.

### 6.2.6. Progression-free Survival 2

PFS2 will be calculated in months for Phase 3 portion of the study as follows:

PFS2 (months) = [minimum of (date of death, date of second objective PD) or censoring – date of randomization +1]/30.4375

As defined in Section 3.2.2.6, second objective PD is PD after the start of subsequent anticancer therapy based on Investigator assessment. A participant will be considered to have an event if

090177e194e374fe\Approved\Approved On: 10-Sep-2020 19:24 (GMT)

1. the participant had PD by BICR on or prior to start of next-line anticancer treatment, AND started next-line anticancer treatment, AND (had objective PD after start of next-line anticancer treatment OR discontinued next-line anticancer treatment).

   OR

2. the participant died.

The censoring and event date options to be considered for PFS2 analysis are presented in Table 10.

PFS2 time will be estimated using the same Kaplan-Meier method as described for PFS.

**Table 10: Outcome, Event Dates, Censoring Reasons and Hierarchy for PFS2 Analyses**

| **Scenario** | **Date of event/ censoring** | **Event/ Censoring reason/ Censoring hierarchy** |
|---|---|---|
| (No PD[a]) and (no death) | Date of last adequate tumor assessment[b] documenting no PD | Censored/ No PD/ 1 |
| (No PD[a]) and death | Date of death | Event (Death) |
| (PD[a] and no NTX[c]) and death | Date of death | Event (Death) |
| (PD[a] date > NTX[c] start date) and (no death) | Start date of NTX[c] | Censored/ Start of new anticancer treatment before PD/ 2 |
| (PD[a] date > NTX[c] start date) and death | Date of death | Event (Death) |
| (PD[a] date ≤ NTX[c] start date) and (PD2[d] date > NTX[c] start date) | | |
| • If PD2 date ≤ NTX[c] end date | Date of PD2 | Event (PD after start of next line anticancer treatment) |
| • If PD2 date > NTX[c] end date | End date of NTX[c] | Event (Discontinuation of next line anticancer treatment) |
| (PD[a] date ≤ NTX[c] start date) and (no PD2[d]) and (no death) | | |
| • If NTX[c] end date is non-missing | End date of NTX[c] | Event (Discontinuation of next line anticancer treatment) |
| • Else if [withdrawal of consent date ≥ date of randomization OR End of study (EOS) = Participant refused further follow-up] | (Withdrawal of consent date) or (EOS visit date where participant refusal of further follow-up is recorded) | Censored/ Withdrawal of consent/ 3 |
| • Else if [lost to follow-up in any disposition page] | Last contact date | Censored/ Lost to follow-up/ |



090177e194e374fe\Approved\Approved On: 10-Sep-2020 19:24 (GMT)

| Scenario | Date of event/ censoring | Event/ Censoring reason/ Censoring hierarchy |
|---|---|---|
| | | 4 |
| • Else if no prior conditions are met | Last contact date | Censored/ Ongoing without PFS2 event/ 5 |
| (PD[a] and no NTX[c]) and (no death) | Last contact date | Censored/ Ongoing without PFS2 event/ 6 |
| (PD[a] date ≤ NTX[c] start date) and (no PD2[d]) and death | | |
| • If NTX[c] end date is non-missing | End date of NTX[c] | Event (Discontinuation of next line anticancer treatment) |
| • Else if the prior condition is not met | Date of death | Event (Death) |

[a] PD is the first PD by BICR per RECIST v1.1, without considering any censoring rules

[b] If there are no adequate post-baseline assessments, then the censoring date is the date of randomization. If a participant has initiated next-line anticancer treatment, the last adequate post-baseline assessment on or prior to start date of next-line anticancer treatment will be considered.

[c] NTX is the next-line anticancer treatment.

[d] PD2 is the first PD by investigator assessment per RECIST v1.1 after initiation of next-line anticancer treatment, without considering any censoring rules.

### 6.2.7. Efficacy Endpoints of Arm A vs. Arm B Comparison

For the Phase 3 portion of the study, the following efficacy endpoints will also be analyzed between Arm A and Arm B using the same analysis methods as those used for the corresponding endpoint of Arm A/Arm B vs. the Control Arm. No formal statistical testing will be performed on these endpoints between Arm A and Arm B.

- PFS by BICR and by Investigator assessment.
- OS.
- ORR by BICR and by Investigator assessment.
- DOR by BICR and by Investigator assessment.
- PFS2.
- TTR by BICR and by Investigator assessment.



090177e194e374fe\Approved\Approved On: 10-Sep-2020 19:24 (GMT)

## 6.3. Other Secondary Endpoints

### 6.3.1. Patient-Reported Outcomes

The following PRO analyses will be performed to support the CSR development. All other PRO analyses described in the study protocol but are not included in this SAP will be described in details in a separate PRO analysis plan.

#### 6.3.1.1. Scoring

The EORTC QLQ-C30 and EQ-5D-5L will be scored according to their respective validation papers and User's Guides (Fayers et al.)[11] The current version at the time of analysis of UK weights will be used to construct the index values of the EQ-5D-5L. Within-treatment arm and between-treatment arm comparisons to baseline will also be performed in order to assess changes among treatment arms.

PGIS and PGIC questionnaire items will be analyzed separately. Items within each questionnaire will not be combined or scored.

#### 6.3.1.2. Instrument Completion Rates

For each treatment arm and at each time point, the number and percentage of participants who complete each of the EORTC QLQ-C30, EQ-5D-5L and VAS, PGIS, and PGIC will be summarized, as will the reasons for non-completion of these measures.

#### 6.3.1.3. Descriptive Summary

For the Global Health Status/QoL scale, the subscales of the EORTC QLQ-C30 and EQ-5D-5L VAS, a line chart depicting the means along with standard error bars over time will be provided for each scale at each time point by treatment arm.

#### 6.3.1.4. Health Status

For the EQ-5D-5L, the number and percentage of participants reported having "none", "slight", "moderate", "severe", or "extreme/unable" problems at each time point will be calculated along with the 2-sided 95% CI using the Clopper-Pearson method (exact CI for a binomial proportion as computed by default by the FREQ procedure using the EXACT option).

### 6.3.2. Pharmacokinetic Analysis

#### 6.3.2.1. Safety Lead-in

Plasma concentrations and PK parameter estimates will be determined for encorafenib, its metabolite (LHY746), irinotecan and its metabolite SN-38, and oxaliplatin (total platinum in plasma and platinum in ultrafiltrate). PK summaries will be presented by cohort by visit for participants in the PK set. Cycle 1 Day 15 is defined as steady state of encorafenib.

090177e194e374fe\Approved\Approved On: 10-Sep-2020 19:24 (GMT)

#### 6.3.2.1.1. Plasma Concentrations of Encorafenib and LHY746, Irinotecan and SN-38, and Oxaliplatin

In the SLI EC + FOLFIRI cohort, plasma concentrations of encorafenib and its metabolite (LHY746), and irinotecan and its metabolite (SN-38) will be quantified at the time points indicated in Table 11. In the SLI EC + mFOLFOX6 cohort, plasma concentrations of encorafenib and its metabolite (LHY746), and oxaliplatin (total platinum in plasma and platinum in ultrafiltrate) will be quantified at the time points indicated in Table 12.

**Table 11: PK Sampling Schedule for EC + FOLFIRI Cohort in the Safety Lead-in**

| Visit | Sampling Time | Irinotecan PK Sample (3 mL Blood) | Encorafenib PK Sample (2 mL Blood) |
|---|---|---|---|
| **Cycle 1 Day 1** | 0 hr (predose[b]) | X | |
| | 0.75 hr | X | |
| | 1.5 hr | X[c] | |
| | 2.5 hr | X | |
| | 3.5 hr | X | |
| | 5.5 hr | X | |
| | 7.5 hr | X | |
| **Cycle 1 Day 3** | Predose[d] | X | |
| **Cycle 1 Day 15[a]** | 0 hr (predose[d]) | X | X |
| | 0.75 hr | X | X |
| | 1.5 hr | X[c] | X |
| | 2.5 hr | X | X |
| | 3.5 hr | X | X |
| | 5.5 hr | X | X |
| | 7.5 hr | X | X |
| **Cycle 1 Day 17** | Predose[d] | X | X |
| **Day 1 of Cycle 2 – 6** | Predose[d] | X | X |

[a] Encorafenib will be administered within 5 minutes before the start of irinotecan infusion
[b] Predose is within 30 minutes prior to irinotecan infusion
[c] Immediately before irinotecan infusion ends
[d] Predose is within 30 minutes prior to encorafenib



090177e194e374fe\Approved\Approved On: 10-Sep-2020 19:24 (GMT)

## Table 12: PK Sampling Schedule for EC + mFOLFOX6 Cohort in the Safety Lead-in

| **Visit** | **Sampling Time** | **Oxaliplatin PK Sample** (5 mL Blood) | **Encorafenib PK Sample** (2 mL Blood) |
|---|---|---|---|
| **Cycle 1 Day 1[a]** | 0 hr (predose[b]) | X | X |
| | 1 hr | X | X |
| | 2 hr | X[c] | X |
| | 3 hr | X | X |
| | 4 hr | X | X |
| | 6 hr | X | X |
| | 8 hr | X | X |
| **Cycle 1 Day 3** | Predose[b] | X | X |
| **Cycle 1 Day 15[a]** | 0 hr (predose[b]) | X | X |
| | 1 hr | X | X |
| | 2 hr | X[c] | X |
| | 3 hr | X | X |
| | 4 hr | X | X |
| | 6 hr | X | X |
| | 8 hr | X | X |
| **Cycle 1 Day 17** | Predose[b] | X | X |
| **Day 1 of Cycle 2 – 6** | Predose[b] | X | X |

[a] Encorafenib will be administered within 5 minutes before the start of oxaliplatin infusion
[b] Predose is within 30 minutes prior to encorafenib
[c] Immediately before oxaliplatin infusion ends

All plasma concentration values for participants in the PK analysis set will be included in the bioanalytical plasma concentration listings. Individual concentration records will be flagged for the affected visit if any of the following occur:

a. Participant vomits within 4 hours following encorafenib dosing on the day of PK sampling (Cycle 1 Day 1 and Cycle 1 Day 15).

b. Encorafenib plasma levels are not considered to be at steady-state (ie, encorafenib dosing was not performed for at least 3 consecutive days prior to Cycle 1 Day 15).

c. Participant receives a higher or lower dose of encorafenib compared to planned treatment on the day of PK sampling (Cycle 1 Day 1 and Cycle 1 Day 15).

d. Participant receives a higher or lower dose of irinotecan compared to planned treatment on the day of PK sampling (Cycle 1 Day 1 and Cycle 1 Day 15).

e. Participant receives a higher or lower dose of oxaliplatin compared to planned treatment on the day of PK sampling (Cycle 1 Day 1 and Cycle 1 Day 15).

f. PK sampling time deviation >20%



090177e194e374fe\Approved\Approved On: 10-Sep-2020 19:24 (GMT)

The plasma concentrations of encorafenib and LHY746, and irinotecan and SN-38, and oxaliplatin will be summarized for all nominal time points, including predose (trough) concentrations for day 1 of cycle 2 through cycle 6, using the following descriptive statistics: N (number of participants in the population), n (number of participants with non-missing values), arithmetic mean, standard deviation, coefficient of variation (CV), geometric mean, geometric standard deviation, geometric CV, minimum, median and maximum. Exclusion of an individual concentration-time data point from the calculation of summary statistics will follow the rules below:

- For encorafenib and LHY746 on Cycle 1 Day 1, if any of the above flags [a, c, and f] apply; for encorafenib and LHY746 on Cycle 1 Day 15, if any of the above flags [a, b, c, and f] apply.
- For irinotecan and SN-38 on Cycle 1 Day 1, if any of the above flags [a, d and f] apply; for irinotecan and SN-38 on Cycle 1 Day 15, if any of the above flags [a, b, d and f] apply
- For oxaliplatin on Cycle 1 Day 1, if any of above flags [a, e and f] apply; for oxaliplatin on Cycle 1 Day 15, if any of above flags [a, b, e, and f] apply.

Concentrations reported as below the limit of quantification (BLQ) will be set to zero for the calculation of arithmetic mean, standard deviation, CV, minimum, median and maximum. For the calculation of geometric mean and geometric CV, BLQ values will be set as missing. All summary statistics below the limit of quantification will be presented as "< X.XX" where X.XX represents LLOQ.

The median plasma concentration-time profiles on Cycle 1 Day 1 and Cycle 1 Day 15 will be presented graphically for each analyte by cohort, as appropriate, using both linear and semi-logarithmic scales and nominal time. Individual plasma concentration-time profiles will also be presented graphically using linear and semi-logarithmic scales by study day, using actual times. In the median plasma concentration-time profiles of irinotecan and SN-38, or oxaliplatin, the time of the predose concentrations on Cycle 1 Day 3 and Cycle 1 Day 17 will be imputed as nominal time 48 hrs post-start of IV infusion on Cycle 1 Day 1, and Cycle 1 Day 15, respectively; in individual plasma concentration-time profiles, the time will be imputed as the actual time intervals from start of IV infusion. The median and individual trough plasma concentration-visit plots will be presented for each analyte by cohort, as appropriate, using both linear and semi-logarithmic scales.

#### 6.3.2.1.2. Plasma Pharmacokinetic Parameters for Encorafenib and LHY746, Irinotecan and SN-38, and Oxaliplatin

Pharmacokinetic parameters defined in Table 13 for participants in the PK set will be determined, as data permit and when appropriate, with noncompartmental analysis (NCA) using an internally validated software system, oNCA (version 2.1). Actual blood collection times with respect to the actual dose time of corresponding study drug will be used for PK parameter calculation. If actual times are not recorded/available, nominal times will be used. For irinotecan and SN-38, and oxaliplatin, the time of predose samples on Cycle 1 Day 3 and Cycle 1 Day 17 will be imputed as the actual time intervals from start of infusion on Cycle 1



090177e194e374fe\Approved\Approved On: 10-Sep-2020 19:24 (GMT)

Day 1 and Cycle 1 Day 15, respectively, and included in the individual plasma concentration-time data for calculation of PK parameters. All BLQ values before the observed maximum plasma concentration ($C_{max}$) will be set to 0; all BLQ values after $C_{max}$ will be considered as missing.

The AUC parameters will be calculated according to the linear-up log-down trapezoidal rule. Additional PK parameters may be calculated at the discretion of the pharmacokineticist.

All PK parameter values will be presented in data listings by analyte and visit. Each parameter will be summarized in tables by analyte and visit using the following descriptive statistics: N, n, arithmetic mean, standard deviation, CV, geometric mean, geometric CV, minimum, median and maximum. For Tmax values, median, minimum and maximum will be presented. Exclusion of pharmacokinetic parameters from the calculation of summary statistics will follow the rules below:

- For encorafenib and LHY746 on Cycle 1 Day 1, if any of the above flags [a, and c] apply; for encorafenib and LHY746 Cycle 1 Day 15, if any of the above flags [a, b, and c] apply.

- For irinotecan and SN-38, if any of the above flags [a and d] apply; for irinotecan and SN-38 on Cycle 1 Day 15, if any of the above flags [a, b and d] apply.

- For oxaliplatin on Cycle 1 Day 1, if any of above flags [a and e] apply; for oxaliplatin on Cycle 1 Day 15, if any of above flags [a, b and e] apply.

**Table 13: Pharmacokinetic Parameters**

| **Analytes** | **PK Parameters** |
|---|---|
| <u>Oral</u><br>Encorafenib and the metabolite LHY746 | $C_{max}$, $C_{trough}$, $AUC_{last}$, $AUC_{tau}$, $R_{ac,Cmax}$, $R_{ac,AUC}$, $MR_{Cmax}$, $MR_{AUC}$, $T_{max}$, $AUC_6$, CL/F, $V_z$/F |
| <u>IV infusion</u><br>Irinotecan and the metabolite SN-38<br>Oxalipatin (plasma platinum and ultracentrifugual plasma platinum) | $C_{max}$, $C_{trough}$, $AUC_{last}$, $AUC_{48}$, $R_{ac,Cmax}$, $R_{ac,AUC}$, $MR_{Cmax}$, $MR_{AUC}$, $T_{max}$, $AUC_{inf}$, $k_{el}$, $t_{1/2}$, CL, $V_z$ |

Note:

- Pharmacokinetic parameters will be determined, as data permit and when appropriate, with noncompartmental analysis (NCA) using an internally validated software system, oNCA (version 2.1).
- When appropriate, dose normalized PK parameters may be generated.



090177e194e374fe\Approved\Approved On: 10-Sep-2020 19:24 (GMT)

#### 6.3.2.1.3. Drug-drug Interaction

Effect of steady-state encorafenib on irinotecan and SN-38 pharmacokinetics or oxaliplatin PK will be evaluated based on overall assessment of the geometric mean ratios (Cycle 1 Day 15/Cycle 1 Day 1) and associated 90% CI for PK parameter estimates of irinotecan and SN-38 or oxaliplatin, respectively, (ie, $C_{max}$, AUC, CL).

The effect of a single dose of encorafenib on oxaliplatin PK, as appropriate, may be evaluated by comparing exposures of oxaliplatin (ie, $C_{max}$, AUC) on Cycle 1 Day 1 in EC + mFOLFOX6 cohort to historical data. The effect of irinotecan or oxaliplatin on encorafenib and LHY746 may be evaluated by comparison of encorafenib and LHY746 PK results to historical results. Comparisons to historical PK results will be qualitative, exploratory, and only done as data permit.

When appropriate, dose normalized concentrations and PK parameters may be used for the analysis.

### 6.3.2.2. Phase 3

The central laboratory, analytical laboratories, and Pfizer clinical assay group colleagues will be unblinded. If the need arises for early analysis of the PK data (before database lock and release of the randomization codes for the study), a PK unblinding plan will be developed. A PK analyst, who is not associated with the study team, will conduct the analysis to avoid unblinding of the study team.

Trough plasma concentrations of encorafenib and LHY746 on day 1 of Cycle 1 through Cycle 6 will be determined in Arm A and Arm B. The values will be presented in data listings by analyte and visit, and will be summarized descriptively (n, mean, standard deviation, CV, median, minimum, maximum, geometric mean, and its associated CV) by visit and arm. The median and individual trough plasma concentration-visit plots will be presented for each analyte by cohort, as appropriate, using both linear and semi-logarithmic scales.

#### 6.3.2.2.1. Pharmacokinetics of Encorafenib and the Metabolite LHY746 in Chinese Population

Plasma concentration and pharmacokinetic parameters of encorafenib and LHY746 for day 1 of Cycle 1 and steady state (day 15 of Cycle 1, or day 1 or day 15 of Cycle 2 if a dose interruption occurs prior to day 15 of Cycle 1) will be summarized in participants randomized to Arm A in China.

Plasma concentration will be presented in data listings and be summarized descriptively (n, mean, standard deviation, CV, median, minimum, maximum, geometric mean, and its associated CV) by analyte and nominal time for day 1 of Cycle 1 and steady state. The median and individual plasma concentration-time plots will be presented for each analyte, as appropriate, using both linear and semi-logarithmic scales, for day 1 of Cycle 1 and steady-state.



090177e194e374fe\Approved\Approved On: 10-Sep-2020 19:24 (GMT)

Plasma pharmacokinetic parameters listed below, for day 1 of Cycle 1 and steady state, will be estimated using non compartmental analysis if data permits or if considered appropriate:

- $C_{trough}$: trough plasma concentration.
- $C_{max}$: maximum plasma concentrations.
- $T_{max}$: time to maximum plasma concentration.
- $AUC_t$: area under the plasma concentration versus time profile from time 0 to time t.
- $AUC_{tau}$: area under the plasma concentration versus time profile within a dose interval.
- $AUC_{inf}$: area under the plasma concentration versus time curve to infinity.
- $t_{1/2}$: terminal elimination half-life.
- CL/F: oral plasma clearance.
- $V_z/F$: apparent volume of distribution.
- $R_{ac}$: accumulation ratio.
- MR: metabolite ratio.

The values will be presented in data listings by analyte, and be summarized descriptively (n, mean, standard deviation, CV, median, minimum, maximum, geometric mean, and its associated CV).

### 6.3.3. Biomarker Analyses

The secondary biomarker endpoint data will be analyzed for the Phase 3 portion of the study based on the biomarker analysis sets as defined in Section 4. Data will be presented by treatment arm. Summaries of baseline levels, changes from baseline (where appropriate), expression or genetic alterations/status will be reported. For continuous variables, summary statistics may include the mean, ratio to baseline, standard deviation, median, first and third quartiles, %CV, and minimum/maximum levels; for categorical variables, summary may include number and percentage, odds ratio, and frequency statistics, as appropriate.

No duplicate results for biomarker or pharmacodynamic markers are expected. However, if more than one record is received for a particular time point, the duplicate records might be averaged or most recent result will be used for continuous data. For non-continuous data, the results will be reviewed by the study team and a representative sample will be selected. When participants provide both archival and de novo tumor tissue samples at screening, data from the de novo sample will be used in the summary analysis.



090177e194e374fe\Approved\Approved On: 10-Sep-2020 19:24 (GMT)

Data from biomarker assays may be analyzed using graphical methods and descriptive statistics such as Wilcoxon Signed Rank, Wilcoxon Rank Sum Test, Kaplan-Meier estimates of efficacy parameters (eg, PFS, OS) with biomarkers as a covariate, or Box plots. The statistical approach may examine correlations of biomarker results with pharmacokinetic parameters and measures of anti-tumor efficacy.

Regarding exploratory biomarkers, the data analysis will be conducted with the goal of identifying predictive and/or pharmacodynamic (ie, baseline and change from baseline values) biomarkers associated with clinical outcome, encompassing both safety and efficacy. These analyses may not be reported in the Clinical Study Report. Candidate biomarkers will be validated in subsequent trials.

#### 6.3.3.1. Tumor Tissue Analyses

##### 6.3.3.1.1. MSI Status Analyses

Analyses for tumor-based MSI status performed at a central laboratory will be based on the tumor tissue analyses set.

MSI status in tumor tissue is measured at baseline (pre-screening/screening). Participants are expected to be MSI-H, MSS, or MSI-unknown.

A descriptive summary table at baseline for MSI status will be provided. When appropriate, a descriptive summary table comparing results from local MSI/dMMR testing and central MSI testing within the tumor tissue analyses set will be provided. The number and percentage of participants in each category will be tabulated. All data will be listed.

##### 6.3.3.1.2. *BRAF* V600E Analyses

Analyses for tumor-based *BRAF* V600E will be based on the tumor diagnostics concordance analysis set.

Diagnostic concordance assessment of *BRAF* V600E status between local tumor tissue/plasma results (as reported in the eCRF) and the reference, central PCR-based QIAGEN *BRAF* V600E FDA-approved test will be presented. The reference, the central test is the tumor tissue PCR testing performed at screening as part of the enrollment criteria, and all participants are therefore considered *BRAF* V600E positive (ie, detected). Overall concordance will be summarized by treatment arm and in total.

#### 6.3.3.2. cfDNA Analyses

Analyses of cfDNA alterations will be based on either the cfDNA analysis set or the paired cfDNA analysis set.

cfDNA alterations are measured at defined timepoints including baseline, on-treatment and End of Treatment. Biomarkers will be listed and summarized at defined timepoints when appropriate. Participants are expected to have either none or one or more alterations detected (eg, *BRAF* V600E), and participants may have a change in alteration dectected during and/or after treatment.



090177e194e374fe\Approved\Approved On: 10-Sep-2020 19:24 (GMT)

A summary table of *BRAF* V600E mutation at baseline, on-treatment and changes from baseline will be provided. A summary table of mean VAF at baseline, on-treatment and changes from baseline will be provided. All data will be listed.

Summaries of the *BRAF* V600E mutation and mVAF at screening vs. objective response (yes=objective response, and no=no objective response) and changes from baseline vs. objective response will be provided. A summary table of the change in mVAF vs. percent change in tumor volume will be provided when appropriate. Graphical analyses (eg stick plots, box plots, etc) will be provided when appropriate.

PFS and OS analyses will be stratified by mVAF and/or changes in mVAF and will use the same Kaplan-Meier method as described for PFS. Kaplan-Meier curves with appropriate summary statistics including median time and associated 2-sided 95% CIs will be calculated.

Diagnostic concordance assessment of *BRAF* V600E status between the plasma-based *BRAF* V600E test and the reference, central PCR-based QIAGEN *BRAF* V600E FDA-approved test will be presented for cfDNA diagnostics concordance analysis set. The reference, the central test is the tumor tissue PCR testing performed at screening as part of the enrollment criteria, and all participants are therefore considered *BRAF* V600E positive (ie, detected). Overall concordance will be summarized by treatment arm and in total.

### 6.4. Subset Analyses

All the subset analyses will be exploratory; no adjustment for multiplicity will be performed. Analyses will only be performed if there is sufficient sample size. The determination of whether or not there is sufficient sample size will be defined after enrollment is complete and prior to database lock. As a general rule, time to event analyses will not be performed on subgroups unless there are ≥10 events on each treatment arm within the defined subset.

The following subset analyses will be performed for PFS by BICR and OS for Phase 3 FAS participants:

- Age (<65 years vs. ≥65 years).
- Age (<18 years; 18-<65 years; 65-<75 years; ≥75 years).
- Gender (Male vs. Female).
- Race (Caucasian vs. non-Caucasian).
- Race (Asian vs. non-Asian).
- Region based on the IRT system (US/Canada vs. Europe vs. Rest of World).
- ECOG performance status based on the IRT system (0 vs. 1).
- Number of organs involved at baseline (≤2 vs. ≥3).
- Removal status of primary tumor (no resection/partial resection vs. complete resection).



090177e194e374fe\Approved\Approved On: 10-Sep-2020 19:24 (GMT)

- Side of tumor (left vs. right).
- Presence of liver metastases at baseline, based on Target and Non-target lesion assessment (yes vs. no).
- Prior anticancer adjuvant/ neoadjuvant therapy (yes vs. no).
- Duration from initial diagnosis to first metastasis (<12 months vs. >= 12 months).
- *BRAF* V600E mutation per central assessment (Positive vs. Negative/Indeterminate).
- Baseline CEA [≤ upper limit of normal (ULN) vs. > ULN].
- Baseline CRP (≤ ULN vs. > ULN).

In addition, the following subset analyses will be performed for PFS by BICR and OS for Phase 3 FAS participants to support country specific regulatory submission. Results of these subset analyses may not be included in the main CSR.

- Japanese subgroup (participants randomized in Japan).
- Korean subgroup (participants randomized in Korea).
- Chinese subgroup (participants randomized in China).

Subset analyses for PFS, OS will use the censoring rules described in Section 6.1.1. The unstratified HR and its corresponding 95% CI will be calculated per subset level and will be presented in a forest plot.

## 6.5. Baseline and Other Summaries and Analyses

### 6.5.1. Baseline Summaries

The following analyses will be performed by cohort and overall on the FAS for the SLI, and by treatment arm and overall for the Phase 3 on the FAS.

#### 6.5.1.1. Demographic Summary

The following demographic will be summarized by number and percentage:

- Gender (male, female).
- Age (<18; 18-<65; ≥65; ≥75).
- Race (White, Black or Afriacan American, Asian, American Indian or Alaska Native, Native Hawaiian or other Pacific Islander, Other, Not Reported).
- Racial designation for Asian (Japanese, Korean, Chinese, other).
- Ethnicity (Hispanic/Latino, not-Hispanic/Latino).

Age (continuous) will be summarized with descriptive statistics (mean, median, standard deviation, minimum, and maximum).

090177e194e374fe\Approved\Approved On: 10-Sep-2020 19:24 (GMT)

#### 6.5.1.2. Baseline and Disease Characteristics

The following baseline characteristics and disease characteristics will be summarized:

- Body site as collected on the “Primary Diagnosis” eCRF page.
- Stage at initial diagnosis.
- Primary tumor removed prior to study entry (No, Complete resection, Partial resection).
- Side of tumor (left vs. right).
- Number of organs involved based on baseline target and non-target lesion assessment.
- Presence of liver metastases at baseline, based on target and non-target lesion assessment.
- Time since initial diagnosis, defined as [date of first dose (SLI) or date of randomization (Phase 3) – date of initial diagnosis]/30.4375.
- Time since recurrence/metastatic disease (months), defined as [date of first dose (SLI) or date of randomization (Phase 3) – date of recurrence/metastatic disease]/30.4375.
- ECOG Performance status.
- Local *BRAF* V600E status by sample source (tumor tissue vs. plasma)as collected on the “Local BRAF Results” eCRF page.
- KRAS and MSI/MRR results by sample source (tumor tissue vs. plasma) as collected on the “Local KRAS Results” and “Local MSI/MRR Results” eCRF page, respectively.
- Baseline CEA values and CRP values (≤ ULN vs. > ULN) and descriptive summary.
- Baseline MSI status.

#### 6.5.1.3. Stratification Factors

The number of participants per strata will be summarized based on strata reported in the IRT system as described in Section 3.3.1.

#### 6.5.1.4. Medical History

Medical history will be coded using the most current version of MedDRA and summarized by MedDRA’s SOC and PT. Each participant will be counted only once within each PT or SOC. Summaries will be ordered by primary SOC and PT by decreasing frequency based on the EC + mFOLFOX6 treatment arm (SLI) or Arm A (Phase 3).

090177e194e374fe\Approved\Approved On: 10-Sep-2020 19:24 (GMT)

#### 6.5.1.5. Prior Anticancer Treatments

Prior anticancer treatments include systemic therapy, radiation and surgery.

The number and percentage of participants in each of the following anticancer therapy categories will be tabulated:

- Participants with at least one prior anticancer systemic therapy (SLI).
- Participants with at least one prior anticancer adjuvant therapy (Phase 3).
- Participants with at least one prior anticancer radiotherapy.
- Participants with at least one prior anticancer surgery, excluding procedure=biopsy.

All prior anticancer systemic therapy will be presented in a listing.

### 6.5.2. Study Conduct and Participant Disposition

The following analyses will be performed by cohort and overall on the FAS for the SLI, and by treatment arm and overall for the Phase 3 on the FAS.

#### 6.5.2.1. Patient Disposition

The disposition summary will be created for the SLI and the Phase 3 portion of the study separately.

The following disposition categories will be summarized:

- Total number of participants screened.

Number (%) of participants who screened failed overall and grouped by the main reason

- Number (%) of participants who were randomized (Phase 3 only).
- Number (%) of participants who were randomized but not treated (Phase 3 only).
- Number (%) of participants who received at least one dose of study drug.
- Number (%) of participants who are still on treatment.
- Number (%) of participants who discontinued the treatment.
  - Primary reasons for treatment discontinuation.
- Number (%) of participants who discontinued the treatment but are still in long-term follow-up.
- Number (%) of patients who discontinued the study.
  - Primary reasons for study discontinuation.



090177e194e374fe\Approved\Approved On: 10-Sep-2020 19:24 (GMT)

The number and percent of participants enrolled by region, country and site, and the number and percent of participants in each analysis set as described in Section 4 will also be summarized.

**COVID-19 Related Disposition**

A listing of all participants affected by COVID-19 related study disruption will be created. The listing will present subject number identifier by investigational site, and a description of COVID-19 related events including:

- Protocol deviations.
- Adverse events treatment.
- Treatment discontinuation.
- Study discontinuation.
- Death.

#### 6.5.2.2. Protocol Deviations

Potentially important protocol deviations (PIPDs) will be compiled prior to database closure and will be summarized by category and also presented in a listing. Categories will be assigned by the study Clinician.

In addition, PIPDs related to COVID-19 will be presented in a separate listing.

### 6.5.3. Study Treatment Exposure

The following analyses will be performed on the safety analysis set by cohort for the SLI and by treatment arm for the Phase 3.

**Treatment Regimens**

- Safety Lead-in.
  - Participants enrolled in the SLI will receive either a regimen of EC + mFOLFOX6 or a regimen of EC + FOLFIRI in 28 day cycles. Encorafenib is dosed at 300 mg daily; cetuximab, mFOLFOX6 and FOLFIRI are dosed once every 2 weeks with an IV infusion.
- Phase 3.
  - Arm A: Participants randomized to Arm A will receive encorafenib + cetuximab in 28 day cycles. Encorafenib is dosed at 300 mg daily and cetuximab is dosed once every 2 weeks with an IV infusion.

090177e194e374fe\Approved\Approved On: 10-Sep-2020 19:24 (GMT)

- Arm B: Participants randomized to Arm B will receive either a regimen of EC + mFOLFOX6 or a regimen of EC + FOLFIRI in 28-day cycles. The use of mFOLFOX6 or FOLFIRI in Arm B will depend on the results of the SLI of the study. The dosing schedule is the same as the SLI.
- Control Arm: Participants randomized to the Control Arm will receive one of the standard of care chemotherapy per physician choice as descrbied in Table 13 in the protocol. mFOLFOX6, FOLFIRI and FOLFOXIRI are dosed once every 2 weeks with an IV infusion; and CAPOX is dosed once every 3 weeks with an IV infusion.

**Duration of Exposure**

The date of receiving the last non-zero dose is considered as last dose date.

- Encorafenib: daily dosing.

  **duration of exposure** (weeks) = (last dose date – first dose date +1)/7

- Cetuximab, mFOLFOX6, FOLFIRI: once every two weeks on 28-day Cycle

  **duration of exposure** (weeks) = (last dose date – first dose date +14)/7

- CAPOX (oxaliplatin + capecitabline):
  - Oxaliplatin: once every three weeks on 21-day cycle

    **duration of exposure** (weeks) = (last dose date – first dose date + 21)/7

  - Capecitabline: twice daily for 2 weeks followed by a 1-week rest period once every 3 weeks

    **duration of exposure** (weeks) = (last dose date – first dose date + 7)/7

**Cumulative Dose**

Cumulative dose is defined as:

- **Intended cumulative dose** (mg) = sum of all protocol specified doses across each intended day of dosing.
- **Actual cumulative dose** (mg) = sum of all actual doses that the participant received.

For participants who did not take any drug the actual cumulative dose is by definition equal to zero.

**Dose Intensity**

- **Intended dose intensity** (mg/day) = intended cumulative dose/duration of exposure.
- **Actual dose intensity** (mg/day) = actual cumulative dose/duration of exposure.

090177e194e374fe\Approved\Approved On: 10-Sep-2020 19:24 (GMT)

- **Relative dose intensity** (%) = 100* (actual dose intensity/intended dose intensity).

A summary of exposure, including duration of exposure, cumulative dose, actual dose intensity, and relative dose intensity (including categories <50%, 50%-<75%, 75%-<90%, 90%-<110%, and ≥110%, if applicable), will be presented for each study drug. Duration of exposure will also be categorized by time intervals (eg, < 1 months, 1-3 months, etc. as appropriate for the protocol) for which frequency counts and percentages of participants will be provided.

Duration of exposure, cumulative dose, actual dose intensity, and relative dose intensity will also be listed for each participant by treatment arm.

#### 6.5.3.1. Dose Modification

Dose modification will be summarized based on the the dosing data collected on the study treatment CRF page. Dose reduction and interruption will be summarized for each study drug (Leucovorin and 5-FU will be summarized together).

**Dose Reduction**

A dose reduction is defined as a decrease in dose from the protocol-planned dose and a decrease from the previous non-zero dose, even if this decrease has been directly preceded by an interruption. For example, for encorafenib, in the sequence of total daily dose 300 mg – 0 mg – 200 mg, the 200 mg dose will be counted as a reduction.

The number and percentage of participants with at least one dose reduction as well as a breakdown of dose reductions (1, 2, ≥3) will be summarized by treatment arm.

Reasons for dose reductions will also be summarized. Participants can contribute to more than one reason if multiple dose reductions occurred for different reasons, but will only be counted once per reason. Percentages will be calculated based on the total number of participants in the safety analysis set.

**Dose Interruption**

For daily dosing of encorafenib and capecitabine, an interruption is defined a 0 mg dose administered for one or more days. For intermittent dosing of IV drugs, such as cetuximab, Oxaliplatin, Leucovorin+5-FU, Irinotecan and bevacizumab, an interruption is defined a 0 mg dose administered during an infusion session.

The number and percentage of participants with dose interruptions and the corresponding reasons will be summarized. Participants can contribute to more than one reason if multiple dose interruptions occurred for different reasons, but will only be counted once per reason. Percentages will be calculated based on the total number of participants in the safety analysis set.

090177e194e374fe\Approved\Approved On: 10-Sep-2020 19:24 (GMT)

### 6.5.4. Concomitant Medications

Concomitant medications will be summarized by cohort on the safety analysis set for the SLI, and by treatment arm on the FAS for the Phase 3.

Concomitant medications are medications, other than study medications, which started prior to first dose of study treatment and continued during the on-treatment period (see Section 5.2.7) as well as those started during the on-treatment period. Concomitant medications will be coded in the WHO Drug coding dictionary and will be tabulated by ATC classification level 2 and PT by decreasing frequency based on the EC + mFOLFOX6 treatment arm (SLI) or Arm A (Phase 3). In case of equal frequency regarding drug class (respectively drug name), alphabetical order will be used. A participant will be counted only once within a given drug name, even if he/she received the same medication at different times.

### 6.5.5. Subsequent Anticancer Therapies/Procedures

Subsequent anticancer therapies/procedures will be summarized by cohort on the safety analysis set for the SLI, and by treatment arm on the FAS for the Phase 3.

Subsequent anticancer therapies are defined as therapies entered on the “Follow-up Cancer Therapy” CRF page. Subsequent ancicancer radiation and surgery will be derived based on the data collected on the "Radiation Treatment” and “Non-Drug Treatments - Cancer Surgery” CRF page, respectively. The radiation treatments and the surgeries will be considered as the subsequent anticancer procedures if the date of the radiation/surgery is after the last dose date of all study drugs.

The number and percentage of participants within each category (medication therapy, radiation therapy, and surgeries) will be provided by treatment arm.

Medications will be coded using the WHO Drug coding dictionary and will be tabulated by drug category and PT by decreasing frequency based on the EC + mFOLFOX6 treatment arm (SLI) or Arm A (Phase 3). The final list of drug category will be provided upon medical review of all subsequent anti-cancer systemic treatment before database lock.

All subsequent anticancer medication therapy will be presented in a listing.

## 6.6. Safety Summaries and Analyses

All safety analyses will be performed on the safety analysis set by cohort for the SLI and by treatment arm for the Phase 3.

Safety data will be presented in tabular and/or graphical format and summarized descriptively, where appropriate.

090177e194e374fe\Approved\Approved On: 10-Sep-2020 19:24 (GMT)

### 6.6.1. Adverse Events

All analyses will be based on TEAEs as defined in Section 3.4.1 unless otherwise specified. AEs not considered treatment emergent will be flagged in data listings.

Seriousness, toxicity grade, action taken (interruption, reduction, and withdraw) of AEs are as reported by the investigator on the "Adverse Events" CRF.

A high level summary of AEs will include the number and percent of participants with:

- Any TEAEs.
- Serious TEAEs.
- TEAEs with CTCAE Grade ≥3.
- Related TEAEs to:
    - Any study drug.
    - Encorafenib.
    - Cetuximab.
- Related serious TEAEs
    - Any study drug.
    - Encorafenib.
    - Cetuximab.
- TEAEs leading to dose interruption of
    - Any study drug.
    - Encorafenib.
    - Cetuximab.
- TEAEs leading to dose reduction of
    - Any study drug.
    - Encorafenib.
    - Cetuximab.

090177e194e374fe\Approved\Approved On: 10-Sep-2020 19:24 (GMT)

- TEAEs leading to permanent discontinuation from
  - Any study drug.
  - Encorafenib.
  - Cetuximab.
- TEAEs leading to dose reduction of any study drug due to COVID-19.
- TEAEs leading to permanent discontinuation from any study drug due to COVID-19.

Each unique AE at the PT level in each treatment arm of the study for a participant is included in the count.

**Adverse Events by SOC and PT**

The following summaries will be created by SOC and PT by decreasing frequency based on the EC + mFOLFOX6 treatment arm (SLI) or Arm A (Phase 3):

- TEAEs by maximum toxicity grade (all causality).
- TEAEs by maximum toxicity grade (treatment related).
- COVID-19 related TEAEs (all causality).
- Serious TEAEs by maximum toxicity grade (all causality).
- Serious TEAEs by maximum toxicity grade (treatment related).
- TEAEs leading to dose reduction of any study drug due to COVID-19.
- TEAEs leading to permanent discontinuation from any study drug due to COVID-19.

An event will be considered treatment related if the investigator considered the event related to the study drug or this information is unknown.

**Adverse Events by PT Only**

The following summaries will be created by AE PT (ie, summaries will not include SOC) by decreasing frequency based on the EC + mFOLFOX6 treatment arm (SLI) or Arm A (Phase 3):

- TEAEs (all causality) experienced by ≥10% of participants in at least one cohort (SLI) or treatment arm (Phase 3).
- Treatment related TEAEs experienced by ≥10% of participants in at least one cohort (SLI) or treatment arm (Phase 3).



090177e194e374fe\Approved\Approved On: 10-Sep-2020 19:24 (GMT)

- TEAEs with CTCAE Grade ≥3 (all causality) by PT and maximum toxicity grade.
- Serious TEAEs (all causality).
- COVID-19 related serious TEAEs (all causality).

**Adverse Events Leading to Dose Modification by PT**

The following summaries will be created by PT of AEs by decreasing frequency of PTs based on the EC + mFOLFOX6 treatment arm (SLI) or Arm A (Phase 3):

- TEAEs leading to dose interruptions by maximum toxicity grade (all causality).
- TEAEs leading to dose reductions by maximum toxicity grade (all causality).
- TEAEs leading to permanent discontinuation by maximum toxicity grade (all causality).

The AE by PT summary will be created separately for:

- Any study drug.
- Encorafenib.
- Cetuximab.

Each participant will be counted only once within each PT.

All AEs and their attributes will be presented in data listings sorted by participant identifier, AE and date of onset of the AE.

### 6.6.2. Deaths

All deaths, deaths within 30 days after last dose of study drug, deaths within 30 days after first dose of study drug as well as the primary reason for death, will be tabulated based on information from the “Death Details” and “Disposition Long-term Follow-Up” eCRFs.

- Number of deaths.
- Number of deaths within 30 days after *last* dose of study treatment.
- Number of deaths within 30 days after *first* dose of study treatment.
- Primary cause of death.
    - Disease under study.



090177e194e374fe\Approved\Approved On: 10-Sep-2020 19:24 (GMT)

- Study treatment toxicity.
- Unknown.
- Other.

In addition, if there are ≥5 deaths due to COVID-19, a separate death summary will be created.

Date and cause of death will be provided in patient data listing together with selected dosing information (study treatment received, date of first/last administration, dose).

### 6.6.3. Adverse Events of Special Interest

Specific groupings of AEs of special interest (AESI) will be considered and the number of patients with at least one event in each grouping will be reported. Such groups consist of AEs for which there is a specific clinical interest in connection with encorafenib treatment (ie, where encorafenib may influence a common mechanism of action responsible for triggering them) or AEs which are similar in nature (although not identical).

All AESI groupings are defined through the use of PT, High Level Terms (HLT), SOC, Standardized MedDRA Queries (SMQ) or through a combination of these components. The MedDRA terms that define each AESI grouping will be outlined in the Case Retrieval Strategy (CRS), which will be used to map reported AEs to the notable AEs groupings. The list of AESIs may be updated during the course of the trial based on accumulating safety data.

A high level summary of AESIs will include the number and percent of participants with:

- Any AESIs.
- Serious AESIs.
- AESIs with CTCAE Grade ≥3.
- Related AESIs.
- Related serious AESIs.
- AESIs leading to dose interruption of encorafenib.
- AESIs leading to dose reduction of encorafenib.
- AESIs leading to permanent discontinuation of encorafenib.

In addition, AESIs will be summarized by grouping and PT as follows:

- AESIs by maximum toxicity grade (all causality).

090177e194e374fe\Approved\Approved On: 10-Sep-2020 19:24 (GMT)

- AESIs by maximum toxicity grade (treatment related).
- Serious AESIs by maximum toxicity grade (all causality).
- Serious AESIs by maximum toxicity grade (treatment related).
- AESIs leading to dose interruption of encorafenib (all causality).
- AESIs leading to dose reduction of encorafenib (all causality).
- AESIs leading to permanent discontinuation of encorafenib by maximum toxicity grade (all causality).

All AESIs and their attributes will be presented in a data listing including CRS grouping terms and PTs.

### 6.6.4. Laboratory Data

Laboratory results will be converted to International System of Units (SI) units which will be used for applying toxicity grades and for all summaries.

As described in Section 3.3, baseline is defined as the last completed assessment prior to date of first dose for the safety assessments. If there are multiple assessments that meet the baseline definition on the same day without the ability to determine which was truly last, then the worst grade will be assigned as the baseline grade.

Laboratory results will be programmatically classified according to NCI-CTCAE version 4.03 grade. Non-numerical qualifiers will not be taken into consideration in the derivation of grade (eg, hypokalemia Grade 1 and Grade 2 are only distinguished by a non-numerical qualifier and therefore Grade 2 will not be derived). In summary statistics the number and percentage of participants corresponding to grades that only include non-quantitative criteria will be displayed as a blank or NA (not assessed) rather than 0. If there is any overlap between grade criteria (eg, CTCAE grading criteria for Creatinine Increased – a value can fall into one range based on comparison to ULN and another range based on comparison to baseline), the highest (worst) grade would be assigned to that record. Grade 5 is defined in the CTCAE criteria guidance as an event with an outcome of death. Since laboratory data does not collect an outcome, Grade 5 is not used when programmatically grading laboratory data.

Grade 0 or Outside Toxicity Reference (OTR) is not defined specifically in the CTCAE guidance. However, programmatically this is used as a category to represent those participants who did not meet any of the Grades 1 to 4 criteria. If the laboratory value is evaluable for CTCAE criteria grading (numeric value is present, valid units and ranges are present as required to allow conversion to standard units and grading), and does not qualify for any of the Grade 1-4 criteria for a given lab test, then the value is assigned as Grade 0 or OTR.

090177e194e374fe\Approved\Approved On: 10-Sep-2020 19:24 (GMT)

Abnormalities will be described using the worst grade post-baseline. When determining the maximum post-baseline grade for a given participant and CTCAE test, the maximum across all analytes and assessments contributing to that CTCAE test will be used. Several laboratory tests have bi-directional grading criteria defined so that both low (hypo) and high (hyper) values can be graded separately. Each criterion will be summarized separately. In the cases where a value is graded as a Grade 1, 2, 3, or 4 for one of the directions, that value will also be assigned as a Grade 0 for the opposite direction for that test. For example, a value meeting the criteria for Grade 3 Hypercalcemia will be classified as a Grade 0 Hypocalcemia. For CTCAE terms that can be derived using one of several laboratory tests, the maximum post-baseline grade for a given participant and CTCAE term will be the maximum across all possible laboratory tests.

For **WBC differential counts** (total neutrophil [including bands], lymphocyte, monocyte, eosinophil, and basophil counts), the absolute value will be used when reported by the lab. When only percentages are available (this is mainly applicable for neutrophils and lymphocytes, because the CTCAE grading is based on the absolute counts), the absolute value is derived as follows:

Derived differential absolute count = (WBC count) × (Differential %value/100)

If the investigator reports both the absolute and % value for Neutrophils or Lymphocytes from the same laboratory sample date and participant, ONLY the absolute value will be graded. The % value will not be graded in this scenario.

If the % value is converted to the differential absolute count for grading and the LLN for the differential absolute count is not available (only LLN for % is available) then Grade 1 will be assigned if the following conditions are met:

- Lymphocyte count decreased:
    - derived absolute count does not meet Grade 2-4 criteria, and
    - % value < % LLN value, and
    - derived absolute count $\geq 800/mm^3$.
- Neutrophil count decreased
    - derived absolute count does not meet Grade 2-4 criteria, and
    - % value < % LLN value, and
    - derived absolute count $\geq 1500/mm^3$.



090177e194e374fe\Approved\Approved On: 10-Sep-2020 19:24 (GMT)

For **calcium**, CTCAE grading is based on Corrected Calcium and Ionized Calcium. Corrected Calcium is calculated from Albumin and Calcium as follows:

- Corrected Calcium (mg/dL) = measured total calcium (mg/dL) – 0.8 [serum albumin (g/dL)-4].

Laboratory toxicities will be tabulated using descriptive statistics (number of participants and percentages). If both central and local labs are collected for a subject, summaries of worst on-treatment abnormalities will be based on both local and central lab data.

Additional laboratory results that are not part of NCI-CTCAE will be presented according to the categories: below normal limit, within normal limits and above normal limit (according to the laboratory normal ranges). The following summary tables will be created:

- Shift summary of laboratory parameters during the on-treatment period by maximum CTCAE grade.
- Shift summary of laboratory parameters from ≤ Grade 2 at baseline to ≥ Grade 3 post-baseline.
- Shift summary of laboratory test results with no CTCAE criteria by worst on-treatment assessment.

All laboratory test results will be presented in a data listing sorted by patient identifier, laboratory test, and date/time of collection. The CTCAE grades and the classifications relative to the laboratory reference ranges will be presented. Values outside laboratory normal ranges will be flagged where appropriate and the central laboratory data and local laboratory data will be flagged accordingly.

**Drug Induced Liver Toxicity**

Alanine aminotransferase (ALT), aspartate aminotransferase (AST), Alkaline Phosphatase (ALP), and total bilirubin (TBILI) are used to assess possible drug induced liver toxicity. The ratios of test result over the ULN will be calculated and classified for these three parameters during the on-treatment period.

Summary of liver function tests will include the following categories. The number and percentage of participants with each of the following during the on-treatment period will be summarized:

- ALT ≥3×ULN, ALT ≥5xULN, ALT ≥10×ULN, ALT ≥20×ULN.
- AST ≥3×ULN, AST ≥5xULN, AST ≥10×ULN, AST ≥20×ULN.
- (ALT or AST) ≥3×ULN, (ALT or AST) ≥5×ULN, (ALT or AST) ≥10×ULN, (ALT or AST) ≥20×ULN.



090177e194e374fe\Approved\Approved On: 10-Sep-2020 19:24 (GMT)

- TBILI ≥2×ULN.
- Concurrent ALT ≥3×ULN and TBILI ≥2×ULN.
- Concurrent AST ≥3×ULN and TBILI ≥2×ULN.
- Concurrent (ALT or AST) ≥3×ULN and TBILI ≥2×ULN.
- Concurrent (ALT or AST) ≥3×ULN and TBILI ≥2×ULN and ALP >2×ULN.
- Concurrent (ALT or AST) ≥3×ULN and TBILI ≥2×ULN and ALP ≤2×ULN or missing.

Concurrent measurements are those occurring on the same date.

Categories will be cumulative, ie, a participant with an elevation of AST ≥10×ULN will also appear in the categories ≥5×ULN and ≥3×ULN. Liver function elevation and possible Hy's Law cases will be summarized using frequency counts and percentages.

An evaluation of Drug-Induced Serious Hepatotoxicity (eDISH) plot will also be created, with different symbols for different treatment arms, by graphically displaying:

- Peak serum ALT(/ULN) vs peak total bilirubin (/ULN) including reference lines at ALT=3×ULN and total bilirubin=2×ULN.
- Peak serum AST(/ULN) vs peak total bilirubin (/ULN) including reference lines at AST=3×ULN and total bilirubin=2×ULN.

In addition, a listing of all TBILI, ALT, AST and ALP values for participants with a post-baseline TBILI ≥2×ULN, ALT≥3×ULN or AST ≥3×ULN will be provided.

### 6.6.5. Vital Signs

Vital signs data includes weight, pulse, systolic blood pressure (BP), and diastolic BP. Measurements were only to be provided once per timepoint. If multiple assessments are provided per timepoint, the maximum value will be used for reporting.

The following criteria define clinically notable vital sign abnormalities:

- Clinically notable elevated values.
    - Systolic BP: ≥160 mmHg and an increase ≥20 mmHg from baseline.
    - Diastolic BP: ≥100 mmHg and an increase ≥15 mmHg from baseline
    - Heart rate (collected as pulse rate in the vital signs eCRF): ≥120 bpm with increase from baseline of ≥15 bpm



090177e194e374fe\Approved\Approved On: 10-Sep-2020 19:24 (GMT)

  - Weight: increase from baseline of ≥10%
  - Body temperature [C]: ≥37.5 C
- Clinically notable low values
  - Systolic BP: ≤90 mmHg with decrease from baseline of ≥20 mmHg;
  - Diastolic BP: ≤50 mmHg with decrease from baseline of ≥15 mmHg;
  - Heart rate (collected as pulse rate in the vital signs eCRF): ≤50 bpm with decrease from baseline of ≥15 bpm;
  - Weight: ≥20% decrease from baseline;
  - Body temperature [C]: ≤36 C.

All assessments, including unscheduled assessments will be considered. A participant can be included in multiple categories if different criteria are met at different timepoints.

Number and percentage of participants with at least one post-baseline vital sign abnormality will be summarized.

Participants with clinically notable vital sign abnormalities will be presented in a data listing. Clinically notable values will also be flagged in this listing.

### 6.6.6. Electrocardiograms

As per protocol, baseline ECG will be obtained as the mean of the triplicate measurements collected at Cycle 1 Day 1 predose.

Heart rate, QT, PR and QRS data will be collected on the ECG eCRF page. QTcF (Fridericia correction) will be derived as follows:

- Fridericia's formula: QTcF= QT/ $\sqrt[3]{RR}$, where RR = 60/heart rate

Number and percentages of participants with clinically notable ECG values during the on-treatment period will be summarized. The clinically notable ECG values are defined in Table 14.

Participants with clinically notable ECG values will be presented in a data listing. Clinically notable values will also be flagged in this listing.



090177e194e374fe\Approved\Approved On: 10-Sep-2020 19:24 (GMT)

**Table 14: Clinical Notable ECG Criteria**

| Parameter | Criterion |
|---|---|
| QT, QTcF | increase from baseline >30 ms |
| | increase from baseline >60 ms |
| | new >450 ms |
| | new >480 ms |
| | new >500 ms |
| Heart rate | Increase from baseline >25% and to a value >100 bpm |
| | Decrease from baseline >25% and to a value <50 bpm |

# 7. INTERIM ANALYSES

## 7.1. Introduction

There are two interim analyses planned on this study. An interim non-binding futility analysis for the primary endpoint of PFS by BICR and an interim efficacy analysis for the key secondary endpoint of OS.

The interim non-binding futility analysis will be performed by an independent statistician. Unblinded results from this interim analysis will not be communicated to the Sponsor's clinical team nor to any party involved in the study conduct (apart from the independent statistician and E-DMC members) until the E-DMC has determined that (i) PFS analysis has crossed the prespecified boundary for futility or (ii) the study needs to be terminated due to any other cause, including safety reasons. Further details are described in the E-DMC charter.

If one or both of the hypothesis tests of the PFS primary endpoint is statistically significant, an interim analysis for superiority will be conducted at that time on the corresponding OS comparison. This interim analysis will be performed by the Sponsor's statistical team at the time of the PFS analysis and the results will be unblinded to the Sponsor.

All participants will continue to be followed for OS until the final OS analysis (or earlier if OS reaches statistical significance at the interim efficacy analysis).

## 7.2. Interim Analyses and Summaries

At each analysis timepoint, the critical boundaries for the group sequential test will be derived from the predefined spending functions as described in Section 7.2.1 for PFS and Section 7.2.2 for OS. The calculations of boundaries will be performed using EAST software (Cytel).[4]

090177e194e374fe\Approved\Approved On: 10-Sep-2020 19:24 (GMT)

### 7.2.1. Interim Futility Analysis for PFS

The interim non-binding futility analysis will be conducted on both primary endpoint comparisons once there are at least 157 PFS events in Arm A + Control Arm (ie, approximately 40% information) and at least 157 PFS events in Arm B + Control Arm (ie, approximately 50% information). This is anticipated to occur approximately 20 months after the first participant is randomized.

The non-binding futility boundaries will be determined using a Rho family beta-spending function with parameter = 3. For PFS of Arm A vs. the Control Arm, with 157 PFS events, the futility boundary will be crossed if the HR is >1.04. For PFS of Arm B vs. the Control Arm, with 157 PFS events, the futility boundary will be crossed if the HR is >0.96. The exact futility boundaries will be updated prior to the time of the analysis based on the actual number of events and the information fraction.

The HR for PFS of Arm A vs. the Control Arm, and of Arm B vs. the Control Arm will be calculated using the Cox's proportional hazards model stratified by randomization strata as described in Section 6.1.1. The corresponding 2-sided CI at pre-defined alpha level will also be calculated.

### 7.2.2. Interim Efficacy Analysis for OS

If one or both of the hypothesis tests of the PFS primary endpoint is statistically significant, an interim analysis for superiority will be conducted at that time on the corresponding OS comparison. It is anticipated that this will occur at approximately 70% information in the Arm A vs. the Control Arm OS comparison (ie, approximately 282 OS events in these two arms) and at approximately 80% information in the Arm B vs. the Control Arm OS comparison (ie, approximately 275 OS events in these two arms).

A Lan-DeMets alpha-spending function[10] that approximates O'Brien-Fleming stopping boundaries will be used to control the overall type I error rate at 0.0125 level (1-sided).

Table 15 summarizes the operating characteristics and efficacy boundaries for the OS analysis. The exact efficacy boundaries and the actual alpha spent at this interim analysis will be updated prior to the time of the analysis based on the actual number of events and the information fraction.

The interim analysis of OS will be performed using the methodology described in Section 6.2.1.1.

090177e194e374fe\Approved\Approved On: 10-Sep-2020 19:24 (GMT)

## Table 15: OS – Summary of Planned Efficacy Boundaries

| Formal Testing | Analysis | Information Fraction[a] | Cumulative OS Events | Cumulative Type I Error (1-sided)[b] | Efficacy Boundaries | | Cum. Prob of Reject $H_0$ for Efficacy if $H_1$ is True |
|---|---|---|---|---|---|---|---|
| | | | | | P-scale (1-sided) | HR Scale | |
| Arm A vs Control | Interim Efficacy | 70% | 282 | 0.0028 | 0.0028 | 0.72 | 0.496 |
| | Final | 100% | 403 | 0.0125 | 0.0116 | 0.80 | 0.850 |
| Arm B vs Control | Interim Efficacy | 80% | 275 | 0.0052 | 0.0052 | 0.73 | 0.654 |
| | Final | 100% | 344 | 0.0125 | 0.0109 | 0.78 | 0.850 |

The efficacy boundaries are determined using Lan-DeMets alpha spending function that approximates O'Brien-Fleming stopping boundaries.
[a] The exact efficacy boundaries and the actual alpha spent on each analysis will be updated based on the actual number of events observed.
[b] The overall type I error rate was assumed to be 0.0125 (1-sided) for each endpoint. The actual alpha value used during testing will be based on the graphical multiple testing strategy outlined in Section 5.1.2.2.

090177e194e374fe\Approved\Approved On: 10-Sep-2020 19:24 (GMT)

## 8. REFERENCES


1. Maurer W, Bretz FJSiBR. Multiple testing in group sequential trials using graphical approaches. 2013;5(4):311-20.

2. Jennison C, Turnbull BW. Group sequential methods with applications to clinical trials: CRC Press; 1999.

3. Brookmeyer R and Crowley J. A confidence interval for the median survival time. Biometrics 38:29-41, 1982.

4. East® version 6.5 manual (2018). Cytel Inc. Cambridge, MA USA.

5. Schoenfeld D. Chi-squared goodness-of-fit tests for the proportional hazards regression model. Biometrika 1980; 67:145-53.

6. Therneau TM, Grambsch PM. Modeling Survival Data: Extending the Cox Model. Statistics for Biology and Health 2000.

7. Zhang X. Comparison of restricted mean survival times between treatments based on a stratified Cox model. DOI 10.1515/bams-2013-0101 Bio-Algorithms and Med-Systems 2013; 9(4): 183–9.

8. Amit O, et al. Blinded independent central review of progression in cancer clinical trials: Results from a meta-analysis and recommendation from a PhRMA working group. European Journal of Cancer 47:1772-1778, 2011.

9. Kalbfleisch JD, Prentice, RL. Statistical Analysis of Failure Time Data, 2nd Edition.Hoboken, Wiley Interscience.

10. Lan K, DeMets DL. Discrete sequential boundaries for clinical trials. Biometrika. 1983;70(3):659-63.

11. Fayers PM, Aaronson NK, Bjordal K, Groenvold M, Curran D, Bottomley A, on behalf of the EORTC Quality of Life Group. EORTC QLQ-C30 Scoring Manual, 3rd edn. Brussels, EORTC, 2001.


090177e194e374fe\Approved\Approved On: 10-Sep-2020 19:24 (GMT)

# 9. APPENDICES

## Appendix 1. List of Abbreviations

| Abbreviation | Term |
|---|---|
| AE | adverse event |
| AESI | adverse event of special interest |
| ALP | alkaline phosphatase |
| ALT | alanine aminotransferase |
| AST | aspartate aminotransferase |
| ATC | Anatomic Therapeutic Chemical |
| AUC | area under the curve |
| BICR | blinded independent central review |
| BP | blood pressure |
| BLQ | below the limit of quantification |
| BOR | Best overall response |
| BRAF | B-RAF proto-oncogene, serine/threonine-protein kinase |
| CRS | case retrieval strategy |
| cfDNA | circulating free DNA |
| CI | confidence interval |
| $C_{max}$ | maximum observed concentration |
| CR | complete response |
| CSR | clinical study report |
| CV | coefficient of variation |
| CTCAE | Common Terminology Criteria for Adverse Events |
| DLT | dose-limiting toxicity |
| DOR | duration of response |
| EC | encorafenib plus cetuximab |
| ECOG | Eastern Cooperative Oncology Group |
| ECG | electrocardiogram |
| eCRF | electronic case report form |
| E-DMC | external data monitoring committee |
| EDR | early discrepancy rate |
| EORTC QLQ-C30 | European Organisation for Research and Treatment of Cancer Quality of Life Questionnaire for Cancer Patients – 30 Item Core Questionnaire |
| EOT | end of treatment |
| EQ-5D-5L | EuroQol-5D-5L |
| EQ VAS | EuroQol-visual analogue scale |



090177e194e374fe\Approved\Approved On: 10-Sep-2020 19:24 (GMT)

| Abbreviation | Term |
|---|---|
| FAS | full analysis set |
| FOLFIRI | fluorouracil/leucovorin/irinotecan |
| FOLFOX | fluorouracil/leucovorin/oxaliplatin |
| FOLFOXIRI | fluorouracil/leucovorin/oxaliplatin/irinotecan |
| HLT | High level terms |
| HR | hazard ratio |
| IRT | interactive response technology |
| IV | intravenous |
| LDR | late discrepancy rate |
| LLOQ | Lower limit of quantification |
| mCRC | metastatic colorectal cancer |
| MedDRA | Medical Dictionary for Regulatory Activities |
| mFOLFOX6 | modified fluorouracil/leucovorin/oxaliplatin |
| ms | millisecond(s) |
| MMRM | mixed-model repeated measures |
| NA | not applicable |
| NCI | National Cancer Institute |
| NE | non-evaluable |
| ORR | objective response rate |
| OS | overall survival |
| PD | progressive disease |
| PFS | progression-free survival |
| PFS2 | PFS after next line of treatment |
| PIPDs | potentially important protocol deviations |
| PK | pharmacokinetic(s) |
| PR | partial response |
| PRO | patient-reported outcome |
| PT | preferred term |
| Q1 | first quartile |
| Q3 | third quartile |
| QT | QT interval |
| QTcF | corrected QT (Fridericia method) |
| RECIST | Response Evaluation Criteria in Solid Tumors |
| REML | restricted maximum likelihood |
| RMST | restricted mean survival time |
| SAE | serious adverse event |
| SAP | statistical analysis plan |



090177e194e374fe\Approved\Approved On: 10-Sep-2020 19:24 (GMT)

| Abbreviation | Term |
|---|---|
| SD | stable disease |
| SLI | Safety Lead-in |
| SMQ | standardized MedDRA queries |
| SoC | standard of care |
| SOC | system organ class |
| SOP | standard operating procedure |
| TEAE | treatment-emergent adverse event |
| TTR | Time to response |
| ULN | upper limit of normal |
| WBC | white blood cell |
| WHO | World Health Organization |

090177e194e374fe\Approved\Approved On: 10-Sep-2020 19:24 (GMT)

# Protocol C4221015

## AN OPEN-LABEL, MULTICENTER, RANDOMIZED PHASE 3 STUDY OF FIRST-LINE ENCORAFENIB PLUS CETUXIMAB WITH OR WITHOUT CHEMOTHERAPY VERSUS STANDARD OF CARE THERAPY WITH A SAFETY LEAD-IN OF ENCORAFENIB AND CETUXIMAB PLUS CHEMOTHERAPY IN PARTICIPANTS WITH METASTATIC *BRAF* V600E-MUTANT COLORECTAL CANCER

**Statistical Analysis Plan**
**(SAP)**

**Version:** **6.0**

**Date:** **08 Mar 2024**

090177e1a0219102\Approved\Approved On: 08-Mar-2024 15:37 (GMT)

## TABLE OF CONTENTS

LIST OF TABLES ....... 8
LIST OF FIGURES ....... 9
1. VERSION HISTORY ....... 10
2. INTRODUCTION ....... 15
2.1. Study Objectives, Endpoints, and Estimands ....... 15
2.1.1. Primary Estimands ....... 18
2.1.2. Secondary Estimand ....... 21
2.1.3. Additional Estimands ....... 22
2.2. Study Design ....... 23
3. ENDPOINTS AND BASELINE VARIABLES: DEFINITIONS AND CONVENTIONS ....... 24
3.1. Safety Lead-in ....... 24
3.1.1. Primary Endpoint ....... 24
3.1.2. Secondary Endpoints ....... 24
3.1.2.1. Safety Endpoints ....... 24
3.1.2.2. Efficacy Endpoints ....... 24
3.1.2.3. PK Endpoints ....... 25
3.1.3. Exploratory Endpoints ....... 26
3.2. Phase 3 ....... 26
3.2.1. Primary Endpoints ....... 26
3.2.2. Efficacy Secondary Endpoints ....... 26
3.2.2.1. Overall Survival ....... 26
3.2.2.2. Objective Response Rate by Derived Investigator ....... 26
3.2.2.3. Duration of Response ....... 27
3.2.2.4. Progression-free Survival by Derived Investigator ....... 27
3.2.2.5. Time to Response ....... 27
3.2.2.6. PFS after Next Line of Treatment (PFS2) ....... 27
3.2.3. Other Secondary Endpoints ....... 27
3.2.3.1. Patient-Reported Outcomes Endpoints ....... 27
3.2.3.2. Pharmacokinetic Endpoints ....... 28
3.2.3.3. Biomarker Endpoints ....... 28



090177e1a0219102\Approved\Approved On: 08-Mar-2024 15:37 (GMT)

3.2.4. Exploratory Endpoints ....28
3.3. Cohort 3 ....28
3.3.1. Primary Endpoint ....28
3.3.2. Efficacy Secondary Endpoints ....29
3.3.2.1. Progression-free Survival ....29
3.3.2.2. Objective Response Rate by Derived Investigator ....29
3.3.2.3. Duration of Response ....29
3.3.2.4. Time to Response ....29
3.3.2.5. Overall Survival ....29
3.3.3. Other Secondary Endpoints ....29
3.3.3.1. Patient-Reported Outcomes Endpoints ....29
3.3.3.2. Pharmacokinetic Endpoints ....30
3.3.3.3. Biomarker Endpoints ....30
3.3.4. Exploratory Endpoints ....30
3.4. Baseline Variables ....30
3.4.1. Stratification ....31
3.4.2. Covariates ....31
3.5. Safety Endpoints ....32
3.5.1. Adverse Events ....32
3.5.2. Laboratory Data ....32
3.5.3. Vital Signs Data ....32
3.5.4. Electrocardiograms Data ....32
4. ANALYSIS SETS (POPULATIONS FOR ANALYSIS) ....32
5. GENERAL METHODOLOGY AND CONVENTIONS ....34
5.1. Hypotheses and Decision Rules ....34
5.1.1. Hypotheses and Sample Size Calculation ....34
5.1.1.1. Safety Lead-in ....35
5.1.1.2. Phase 3 ....35
5.1.1.3. Cohort 3 ....36
5.1.2. Decision Rules ....37
5.1.2.1. Safety Lead-in ....37
5.1.2.2. Phase 3 ....38



090177e1a0219102\Approved\Approved On: 08-Mar-2024 15:37 (GMT)

5.1.2.3. Cohort 3 ....................40

5.2. General Methods ....................40

5.2.1. Data Handling After the Cut-off Date ....................40

5.2.2. Pooling of Data by Center ....................40

5.2.3. Analyses to Assess the Impact of COVID-19 Pandemic....................40

5.2.4. COVID-19 Anchor Date....................41

5.2.5. Definition of Start Date ....................41

5.2.6. Definition of Study Day....................41

5.2.7. On-treatment Period....................41

5.2.8. Definition of Start of New Anti-cancer Drug Therapy....................42

5.2.9. Definition of Start of New Anti-cancer therapy ....................42

5.2.10. Date of Last Contact ....................42

5.2.11. Unscheduled Assessments ....................43

5.2.12. Tumor Assessment Date ....................43

5.2.13. Adequate Baseline Tumor Assessment ....................43

5.2.14. Adequate Post-baseline Tumor Assessment....................44

5.2.15. Standard Derivations and Reporting Conventions ....................44

5.2.16. Analyses for Binary and Categorical Endpoints....................44

5.2.17. Analyses for Continuous Endpoints ....................44

5.2.18. Analyses for Time-to-Event Endpoints ....................44

5.3. Methods to Manage Missing Data ....................46

5.3.1. Missing Dates ....................46

5.3.2. Missing Patient-Reported Outcomes Data....................50

5.3.3. Missing Pharmacokinetic Data ....................50

5.3.4. Missing Biomarker Data....................51

6. ANALYSES AND SUMMARIES ....................51

6.1. Primary Endpoints....................51

6.1.1. Safety Lead-in....................51

6.1.2. Phase 3 ....................51

6.1.2.1. PFS by BICR....................51

6.1.2.2. ORR by BICR ....................57

6.1.3. Cohort 3 ....................59




090177e1a0219102\Approved\Approved On: 08-Mar-2024 15:37 (GMT)

6.1.3.1. ORR by BICR ....59
6.2. Efficacy Secondary Endpoints ....60
6.2.1. Safety Lead-in....60
6.2.1.1. Objective Response Rate by Investigator....60
6.2.1.2. Duration of Response by Investigator ....60
6.2.1.3. Time to Response by Investigator ....61
6.2.1.4. Progression-free Survival by Investigator....61
6.2.1.5. Overall Survival ....61
6.2.2. Phase 3 ....62
6.2.2.1. Overall Survival ....62
6.2.2.2. Duration of Response ....63
6.2.2.3. Time to Response ....64
6.2.2.4. Progression-free Survival 2 ....64
6.2.2.5. Efficacy Endpoints of Arm A Versus Arm C and Versus Arm B Comparison....66
6.2.3. Cohort 3 ....66
6.2.3.1. Progression-free Survival ....66
6.2.3.2. Duration of Response ....67
6.2.3.3. Time to Response ....67
6.2.3.4. Overall Survival ....67
6.3. Other Secondary Endpoints....67
6.3.1. Patient-Reported Outcomes (PRO)....67
6.3.1.1. Scoring ....67
6.3.1.2. Instrument Completion Rates....68
6.3.1.3. Descriptive Summary ....68
6.3.1.4. Health Status ....68
6.3.2. Pharmacokinetic Analysis ....68
6.3.2.1. Safety Lead-in ....68
6.3.2.2. Phase 3....73
6.3.2.3. Cohort 3 ....75
6.3.3. Biomarker Analyses....75
6.3.3.1. Tumor Tissue Analyses ....76




090177e1a0219102\Approved\Approved On: 08-Mar-2024 15:37 (GMT)

6.3.3.2. ctDNA Analyses ......76
6.3.4. Surgical conversion rate (exploratory endpoint) ......77
6.4. Subset Analyses......78
6.5. Baseline and Other Summaries and Analyses ......79
6.5.1. Baseline Summaries......79
6.5.1.1. Demographic Summary......79
6.5.1.2. Baseline and Disease Characteristics ......79
6.5.1.3. Stratification Factors ......80
6.5.1.4. Medical History......80
6.5.1.5. Prior Anticancer Treatments ......80
6.5.2. Study Conduct and Participant Disposition......81
6.5.2.1. Patient Disposition ......81
6.5.2.2. Protocol Deviations......82
6.5.3. Study Treatment Exposure ......82
6.5.3.1. Dose Modification......85
6.5.4. Concomitant Medications......86
6.5.5. Subsequent Anticancer Therapies/Procedures......86
6.6. Safety Summaries and Analyses ......86
6.6.1. Adverse Events ......86
6.6.2. Deaths ......90
6.6.3. Adverse Events of Special Interest......90
6.6.4. Laboratory Data......91
6.6.5. Vital Signs ......95
6.6.6. Electrocardiograms......96
6.7. Summaries for Korea, Japan and China......96
6.7.1. Efficacy Analyses ......96
6.7.2. Safety Analyses ......97
6.7.3. Other Analyses......97
7. INTERIM ANALYSES......98
7.1. Introduction ......98
7.2. Interim Analyses and Summaries for Efficacy OS ......98
8. REFERENCES ......101



090177e1a0219102\Approved\Approved On: 08-Mar-2024 15:37 (GMT)

9. APPENDIX 1 LIST OF ABBREVIATIONS ....................102
10. APPENDIX 2 SPECIFICATIONS FOR PROGRAMMATIC DERIVATION OF TUMOR RESPONSE USING RECIST 1.1 ....................105
10.1. Study Specific Information ....................105
10.1.1. Adequate Baseline ....................105
10.1.2. Interchangeable Methods of Tumor Assessment....................106
10.1.3. Period for Derivation of Best Overall Response (BOR) ....................106
10.1.4. New Anticancer Therapy....................106
10.2. Lesion Evaluation at Assessment Times....................106
10.2.1. Response Evaluation of Target Lesions ....................106
10.2.2. Response Evaluation of Non-Target Lesions ....................108
10.2.3. New Lesion Evaluation Criteria ....................109
10.3. Objective Response Status at Each Assessment ....................109
10.4. Date of Response at Each Assessment/Overall:....................110
10.4.1. Best Overall Response Evaluation for Each Patient....................110

090177e1a0219102\Approved\Approved On: 08-Mar-2024 15:37 (GMT)

# LIST OF TABLES


Table 1 Summary of Change ....10

Table 2 Characteristics of DLT Probability During the DLT Evaluation Period ....37

Table 3 Probability of Observing ≥ 1 Instance of Toxicity at 10% and 15% Incidence Rate ....38

Table 4 PFS Outcome and Event Dates – Primary Analysis ....52

Table 5. PFS Censoring Reasons and Hierarchy - Primary Analysis ....53

Table 6 Reverse Censoring Rules for PFS Follow-up Duration ....54

Table 7 PFS Outcome and Event Dates – Supplementary Analysis ....55

Table 8 Discordance Summary for Derived Investigator vs BICR Assessment on PFS ....56

Table 9. Definition of Agreement Used to Determine Total Agreement Rates in Response ....59

Table 10 Reverse Censoring Rules for OS Follow-up Duration ....63

Table 11 Outcome, Event Dates, Censoring Reasons and Hierarchy for PFS2 Analyses ....64

Table 12 PK Sampling Schedule for Cohort 1 (EC + FOLFIRI) in the Safety Lead-in ....69

Table 13 PK Sampling Schedule for Cohort 2 (EC + mFOLFOX6) in the Safety Lead-in ....70

Table 14 Pharmacokinetic Parameters ....73

Table 15 Clinical Notable ECG Criteria ....96

Table 16 OS – Summary of Planned Efficacy Boundaries ....99

Table 17. Derivation of Non-Target Lesion Assessment ....109

Table 18. Derivation of Objective Status Based on Target and Non-Target Lesion Response Assuming No New Lesions* ....110

Table 19. Derivation of Best Overall Response Based on Objective Status at Assessment Time Points Assuming That Confirmation of Response is Not Required ....111

Table 20. Best Overall Response When Confirmation of Response is Required ....112

Table 21. Best Overall Response When Confirmation of Response is Required: Further Examples ....113


090177e1a0219102\Approved\Approved On: 08-Mar-2024 15:37 (GMT)

## LIST OF FIGURES

Figure 1 Study Design....................................................................................24
Figure 2 Multiple Testing Strategy ..........................................................................39

090177e1a0219102\Approved\Approved On: 08-Mar-2024 15:37 (GMT)

# 1. VERSION HISTORY

**Table 1 Summary of Change**

| Version/ Date | Associated Protocol Amendment | Rationale | Specific Changes |
|---|---|---|---|
| 1.0/ 09 September 2020 | 1.0/ 15 July 2020 | N/A | N/A |
| 2.0 / 11 May 2021 | 3.0 / 24 February 2021 | To match latest protocol amendment. Also to add analyses for Korea and Japan. | Updated various cross references that did not work in the text.<br><br>Clarified that the safety set in the SLI was in fact the same as the full analysis set (FAS) in the SLI. The FAS was noted for the SLI. Also replaced treatment arm with treatment cohort for the SLI.<br><br>Added in various points of clarity (confirmed and unconfirmed response in sections 3.1.2.2, 3.2.2.2, 3.2.2.3, removed a discrete summary under the continuous section 5.2.15, clarified PFS2 in section 6.2.6, noted the PRO set in section 6.3.1, noted the PK analysis set in section 6.3.2.2, clarified China in section 6.3.3.2).<br><br>Clarified that secondary endpoints PFS and OS were for Arm A versus Arm B, under section 2.1.<br><br>Modified PGIS to 4 categories and PGIC to 5 categories under section 3.2.3.1.<br><br>Clarified that BICR was used for liver covariate, and replaced Caucasian with White in covariate section 3.3.2.<br><br>Noted that the SLI is analyzed according to actual treatment arm received in section 4.<br><br>Clarified the DLT evaluable set in section 4.<br><br>Included new text for possible two-arm trial only (assuming Arm B was not tolerable per the safety lead-in) in section 2.2, section 5.1.1, and section 7.<br><br>Inserted p-values and removed the hazard ratios for the primary analysis decision criteria for both the primary and key secondary endpoints, under section 5.1.1.2.<br><br>Removed the hazard ratio for decision criteria in table 15.<br><br>Removed the RECIST criteria for the secondary endpoint PFS2.<br><br>Removed the plot of RMST versus time in section 5.2.16.<br><br>Clarified missing dates under section 5.3.1.<br><br>Corrected Table 6 to use the last scan rather than the last known alive date.<br><br>Updated Tables 11, 12, and 13 in PK section 6.3.2 to be more consistent with the protocol. |



090177e1a0219102\Approved\Approved On: 08-Mar-2024 15:37 (GMT)

**Table 1 Summary of Change**

| Version/ Date | Associated Protocol Amendment | Rationale | Specific Changes |
|---|---|---|---|
| | | | Clarified PK analyses in sections 6.3.2.1.1, 6.3.2.1.2, 6.3.2.1.3, and 6.3.2.2.1.<br><br>Removed individual PK plots.<br><br>Removed some biomarker listings under section 6.3.3.2.<br><br>Clarified the race subset, the side of tumor subset, and liver metastasis subset, in section 6.4.<br><br>Added in bevacizumab exposure calculation in section 6.5.3. Also changed categorical summary from months to weeks in section 6.5.3.<br><br>Clarified how to handle dosing for days 1 and 2 for patients taking FOLFIRI (either Cohort 1 in the safety lead-in or Arm B in phase 3), under section 6.5.3.1.<br><br>Removed the SLI cohorts from the AE sorting procedure in section 6.6.1.<br><br>Added in new treatment related summaries in section 6.6.1. Also clarified that treatment related is at least one study drug related.<br><br>Changed the inequality signs to strict inequality for the liver function tests, under section 6.6.4, to match the protocol and FDA guidance.<br><br>Removed a listing under section 6.6.5.<br><br>Removed QT parameter and a listing from section 6.6.6.<br><br>Added a new section 6.7 to cover particular summaries needed for Korea and Japan.<br><br>Inserted p-values and removed the hazard ratios for the futility analysis decision criteria for the primary endpoint, under section 7.2.1.<br><br>In the list of abbreviations (appendix 1), added new entries for MWPC, ND, NR, and OTR. Removed entries not used in the text: FOLFOX, MMRM, REML. |
| 3.0 / 26 April 2022 | 4.0 / 28 February 2022 | To match protocol amendment 4: based on SLI results primary and key endpoints were modified. In addition, for Phase 3 portion, efficacy endpoints based on investigator assessment were | Following results from SLI portion, removed reference to FOLFIRI treatment for Arm B of the Phase 3 portion through the whole document.<br><br>For Phase 3 portion moved the comparison of PFS by BICR between Arm A versus the Control Arm from co-primary endpoint to key secondary endpoint and updated primary and secondary estimands in sections 2.1 and 3.2.<br><br>Updated the sample size and study design in sections 2.2 and 5.1.1. |

090177e1a0219102\Approved\Approved On: 08-Mar-2024 15:37 (GMT)

**Table 1 Summary of Change**

| Version/ Date | Associated Protocol Amendment | Rationale | Specific Changes |
|---|---|---|---|
| | | changed to derived investigator tumor assessment. | For Phase 3 portion specified in sections 3.2.2 and 6.2 that endpoints related to tumor assessment by investigator will be derived programmatically.<br><br>Removed PRO analysis set from section 4.<br><br>Substituted graphical gatekeeping procedure with hierarchical testing procedure to preserve overall alpha of the study in section 5.1.2.2.<br><br>Added definitions of start of new anticancer drug therapy and of start of new anticancer therapy in sections 5.2.8 and 5.2.9.<br><br>Substituted Clopper Pearson CIs by Wilson score CIs in sections 5.2.16 and 6.2.3.<br><br>Specified in section 6 that for SLI portion the efficacy analyses will be performed by number of lines of prior therapies.<br><br>In section 6 analyses for primary and secondary endpoints have been aligned with modified endpoints of Phase 3 portion.<br><br>Added discontinuation of next-line treatment after first objective PD by investigator assessment as event in the PFS2 definition in sections 2.1, 3.2.2.6, 6.2.6.<br><br>Added in section 6.6.1 tables for AEs/SAEs related to chemotherapy and AE associated to dose modification for chemotherapy.<br><br>Changed the criteria to show the deaths from 30 days to 28 days after the last dose of study treatment, in section 6.6.2.<br><br>Added criteria for QRS and PR interval to be included in the table for ECG, in section 6.6.6.<br><br>Updated the number of events and criteria for interim analyses for PFS futility and for OS efficacy in section 7.2.<br><br>Added specification for programmatic derivation of tumor response in section 10.<br><br>Throught the document, included editorial changes to enahance clarity. |
| 4.0 / 17 February 2023 | 5.0 / 20 December 2022 | To match protocol amendment 5 dated 20 Dec 2022: based on FDA inputs/discussion: removed Arm A from key secondary endpoints, added ORR as primary endpoint for Phase | Updated primary and secondary estimands in sections 2.1, 3.2 and 3.3: for Phase 3 portion moved the comparison of PFS by BICR between Arm A versus the Control Arm from the key secondary endpoint to secondary endpoints and added ORR by BICR for Arm B versus the Control Arm as a primary endpoint. Included endpoints for Cohort 3.<br>. |



090177e1a0219102\Approved\Approved On: 08-Mar-2024 15:37 (GMT)

## Table 1 Summary of Change

| Version/ Date | Associated Protocol Amendment | Rationale | Specific Changes |
|---|---|---|---|
| | | 3, added Cohort 3 with 2 randomized arms: Arm D and E | Added Cohort 3 to analysis set definitions and the subgroup "Full Analysis Set, ORR subset" for ORR analysi of Phase 3 in section 4.<br><br>Updated the sample size and study design in sections 2.2 and 5.1.1.<br><br>Modified hierarchical testing procedure to preserve overall alpha of Phase 3 and added for Cohort 3 in sections 5.1.2.2 and 5.1.2.3.<br><br>In section 6 analyses for primary and secondary endpoints have been aligned with modified endpoints of Phase 3 and Cohort 3.<br><br>Added some listing in PK section 6.3.2.<br><br>Modified some AE tables in section 6.6.<br><br>Removed interim analysis for PFS for Phase 3 and updated the number of events and criteria for interim analyses for OS efficacy in section 7. |
| 5.0 / 15 December 2023 | 5.0 / 20 December 2022 | Reviewed after BDR to align with requested minor changes and manage particular cases | Added a few descriptive outputs: included overall exposure for SLI, Phase 3 and Cohort 3, a table for regimens in arm C in Phase 3, a swimmer plot for subsequent therapies for Phase 3, a disposition for end of screening, a plot for OS/PFS in SLI, time to first dose modification, threshold could be applied to some in-text AE tables.<br><br>In Table 16 specified that in case ORR and PFS or only PFS analyses results are statistically significant the nominal alpha level for OS analysis will be 0.023.<br><br>Changed CEA, CRP and MSI categories/criteria for reporting, sections 6.4 and 6.5.1.2.<br><br>Modified efficacy baseline definition and adequate baseline tumor assessment definition to consider assessments performed up to first dose date, sections 3.4, 5.2.13, 10.1.1.<br><br>Specified the NE BOR includes cases with no evidence of disease at baseline (ND), section 6.1.2.2.1.<br><br>Modified the endpoint definition for Phase 3 and Cohort 3, and some analyses for ctDNA biomarkers, sections 2.1, 3.2.3.3, 3.3.3.3, 6.3.3.2.<br><br>Minor editorial changes. |
| 6.0 / 08 March 2024 | 5.0 / 20 December 2022 | Given the slower than expected number of PFS events, the required number per original study design cannot | Updated the required number of PFS events for the final analysis and as a result, decreased the power for the sample size calculation for PFS of Phase 3 portion, sections 5.1.1.2, 5.1.2.2, 6.1.2.1.1. |



090177e1a0219102\Approved\Approved On: 08-Mar-2024 15:37 (GMT)

**Table 1 Summary of Change**

| Version/ Date | Associated Protocol Amendment | Rationale | Specific Changes |
|---|---|---|---|
| | | be achieved. Therefore, the required number of PFS events for final analysis has been reduced to what could be observed in a timely manner. As a result, the power of the study has been reduced from 95% to 85% still acceptable for a Phase 3 study.<br><br>Modified the definition of adequate baseline tumor assessment to reduce the number of not evaluable participants due to missing baseline tumor assessments.<br><br>ctDNA biomarker changes were implemented to incorporate recent findings from ctMoniTR project.<br><br>Added exploratory surgical conversion rate, to assess if study intervention enables patients to have surgical resections with curative intent.<br><br>Added supplementary analysis of PFS to consider events even if they occur after long gap in assessments. | Modified the definition of adequate baseline tumor assessment to accept exams older than 35 days prior randomization for Phase 3 and Cohort 3, sections 5.2.13, 10.1.1.<br><br>Modified the endpoint definition for ctDNA biomarkers for Phase 3 and Cohort 3, and some related analyses, sections 2.1, 3.2.3.3, 3.3.3.3, 6.3.3.2.<br><br>Added exploratory endpoint of surgical conversion rate, sections 2.1, 6.3.4.<br><br>Specified that in Cohort 3, if ORR results are not statistically significant, PFS will be analyzed descriptively, section 5.1.2.3<br><br>Added a supplementary analysis for PFS including observations/events that occur after >12 (or 16) weeks from last adequate post-baseline tumor assessment/start date, section 6.1.2.1.2.<br><br>Minor editorial changes and re-wording for clarity. |

090177e1a0219102\Approved\Approved On: 08-Mar-2024 15:37 (GMT)

# 2. INTRODUCTION

This statistical analysis plan (SAP) provides the detailed methodology for summary and statistical analyses of the data collected in Study C4221015. This document may modify the plans outlined in the protocol; however, any major modifications of the primary endpoint and key secondary endpoint definition or corresponding analyses will also be reflected in a protocol amendment.

As this is an open-label study, the treatment is unblinded on participant level due to different treatment regimens in each treatment arm. However, the aggregate/cumulative data summary by actual treatment arm should remain blinded to the Sponsor and the external investigators until the database lock for the primary analysis. Before the database release date for the ORR analysis of Phase 3 and Cohort 3 of the study, PK concentration and safety data will be early unblinded and provided to a restricted group of pharmacometricians/programmers in order to support preliminary modeling and simulation work ahead of formal analysis of ORR for the planned sNDA. Final exposure-safety analyses will be conducted based on the formal database snapshot for the ORR analysis.

## 2.1. Study Objectives, Endpoints, and Estimands

| Safety Lead-in | |
|---|---|
| **Objectives** | **Endpoints** |
| **Primary** | |
| • To determine the safety and tolerability of EC + mFOLFOX6 and EC + FOLFIRI | • Incidence of DLTs |
| **Secondary** | |
| • To assess the overall safety and tolerability of EC + mFOLFOX6 and EC + FOLFIRI | • Incidence and severity of AEs graded according to the NCI CTCAE v4.03 and changes in clinical laboratory parameters, vital signs and ECGs<br>• Incidence of dose interruptions, dose modifications and discontinuations due to AEs |
| • To estimate the efficacy of EC + mFOLFOX6 and EC + FOLFIRI | • ORR by investigator, defined as the proportion of participants who have achieved a confirmed BOR (CR or PR) per RECIST v1.1<br>• DOR by investigator, defined as the time from the date of first radiographic evidence of response (CR or PR) to the earliest documented disease progression per RECIST v1.1, or death due to any cause<br>• PFS by investigator, defined as the time from the first dose to the earliest documented disease progression per RECIST v1.1, or death due to any cause<br>• TTR by investigator, defined as the time from first dose to first radiographic evidence of response (CR or PR) per RECIST v1.1 |



090177e1a0219102\Approved\Approved On: 08-Mar-2024 15:37 (GMT)

| | |
|---|---|
| • To estimate the efficacy of EC + mFOLFOX6 and EC + FOLFIRI | • OS defined as the time from the first dose to death due to any cause |
| • To characterize the PK of encorafenib, irinotecan, oxaliplatin and relevant metabolites | • PK parameters of encorafenib, irinotecan, oxaliplatin and relevant metabolites |
| • To assess drug-drug interaction of encorafenib with irinotecan or oxaliplatin | • Changes in exposures of irinotecan and its metabolite (SN-38) on Cycle 1 Day 15 compared to Cycle 1 Day 1 in Cohort 1 (EC + FOLFIRI)<br>• Changes in exposures of oxaliplatin on Cycle 1 Day 15 compared to Cycle 1 Day 1 in Cohort 2 (EC + mFOLFOX6) |
| **Tertiary/Exploratory** | |
| • To understand the relationship between the therapeutic intervention(s) being studied and the biology of the participant's disease | • Measurements of biomarkers, consisting of DNA, RNA, protein or defined cell types, resulting from analyses of peripheral blood and/or tumor tissue biospecimen obtained at baseline, on treatment and/or at end-of-study |
| **Phase 3** | |
| **Objectives** | **Endpoints** |
| **Primary** | |
| • To compare the efficacy of EC + mFOLFOX6 (Arm B) vs SOC (Control Arm [Arm C]) as measured by PFS and by ORR | • PFS by BICR, defined as the time from the date of randomization to the earliest documented disease progression per RECIST v1.1, or death due to any cause<br>• ORR by BICR |
| **Key Secondary** | |
| • To further compare the efficacy of Arm B vs the Control Arm as measured by OS | • OS, defined as the time from the date of randomization to death due to any cause |
| **Secondary (Descriptive Statistics Only)** | |
| • To further evaluate the efficacy of Arm B vs the Control Arm as measured by ORR, DOR, PFS, PFS2 and TTR<br>• To evaluate the efficacy of EC (Arm A) vs the Control Arm as measured by ORR, DOR, PFS, PFS2, TTR and OS<br>• To evaluate the efficacy of Arm A vs Arm B as measured by OS, PFS, PFS2, ORR, DOR and TTR | • ORR by investigator<br>• ORR by BICR (Arm A vs Control Arm, Arm A versus Arm B)<br>• DOR by BICR and by investigator<br>• PFS by BICR (Arm A vs Control Arm, Arm A versus Arm B)<br>• OS (Arm A vs Control Arm, Arm A versus Arm B)<br>• PFS by Investigator<br>• TTR (by BICR and by investigator), defined as the time from the date of randomization to first radiographic evidence of response (CR or PR) per RECIST v1.1<br>• PFS2, defined as the time from the date of randomization to the date of discontinuation of next-line treatment after first objective PD by |



090177e1a0219102\Approved\Approved On: 08-Mar-2024 15:37 (GMT)

| | |
|---|---|
| | investigator assessment, the second objective disease progression, or death from any cause, whichever occurs first |
| • To determine the safety and tolerability of EC<br>• To determine the safety and tolerability of EC + mFOLFOX6 | • Incidence and severity of AEs graded according to the NCI CTCAE v4.03 and changes in clinical laboratory parameters, vital signs, and ECGs |
| • To evaluate quality of life and health states, captured by PRO measures | • PRO scores as measured by the EORTC QLQ-C30, EQ-5D-5L, and anchoring instruments PGIS and PGIC |
| • To evaluate trough concentrations of encorafenib and its metabolite LHY746 in Arm A and Arm B | • Trough plasma concentrations of encorafenib and the metabolite LHY746 in Arm A and Arm B |
| • To characterize the PK of encorafenib and its metabolite LHY746 in participants randomized in mainland China (Arm A and Arm B) | • PK parameters of encorafenib and its metabolite LHY746 |
| • To confirm the MSI-status in tumor tissue | • Summarize MSI-status as determined by retrospective central testing of baseline tumor tissue |
| • To determine the correlation between ctDNA, BRAF V600 alterations, and clinical outcome | • ctDNA levels and *BRAF* V600 VAF from ctDNA analysis of plasma samples collected at baseline and on treatment |
| **Tertiary/Exploratory** | |
| • To understand the relationship between the therapeutic intervention(s) being studied and the biology of the participant's disease | • Measurements of biomarkers, consisting of DNA, RNA, protein or defined cell types, resulting from analyses of peripheral blood and/or tumor tissue biospecimen obtained at baseline, on treatment and/or at end-of-study |
| • To understand the surgical conversion rate | • Surgical conversion rate, defined as the rate of participants who become eligible for surgery and undergo surgery with curative intent as a result of study intervention. |
| **Cohort 3** | |
| **Objectives** | **Endpoints** |
| **Primary** | |
| • To compare the efficacy of EC + FOLFIRI (Arm D) vs FOLFIRI with or without bevacizumab (Control Arm [Arm E]) as measured by ORR | • ORR by BICR |
| **Key Secondary** | |
| • To further compare the efficacy of Arm D vs Arm E as measured by PFS | • PFS by BICR, defined as the time from the date of randomization to the earliest documented disease progression per RECIST v1.1, or death due to any cause |



090177e1a0219102\Approved\Approved On: 08-Mar-2024 15:37 (GMT)

| Secondary (Descriptive Statistics Only) | |
|---|---|
| • To further evaluate the efficacy of Arm D vs Arm E as measured by ORR, DOR, PFS, TTR and OS | • ORR by Investigator<br>• DOR by BICR and by Investigator, defined as the time from the date of first radiographic evidence of response (CR or PR) to the earliest documented disease progression per RECIST v1.1, or death due to any cause<br>• PFS by Investigator, defined as the time from the date of randomization to the earliest documented disease progression per RECIST v1.1, or death due to any cause<br>• OS, defined as the time from the date of randomization to death due to any cause<br>• TTR (by BICR and by Investigator), defined as the time from the date of randomization to first radiographic evidence of response (CR or PR) per RECIST v1.1 |
| • To determine the safety and tolerability of EC + FOLFIRI | • Incidence and severity of AEs graded according to the NCI CTCAE v4.03 and changes in clinical laboratory parameters, vital signs, and ECGs |
| • To evaluate quality of life and health states, captured by PRO measures | • PRO scores as measured by the EORTC QLQ-C30, EQ-5D-5L, and anchoring instruments PGIS and PGIC |
| • To evaluate trough concentrations of encorafenib and its metabolite LHY746 in Arm D | • Trough plasma concentrations of encorafenib and the metabolite LHY746 in Arm D |
| • To confirm the MSI status in tumor tissue | • Summarize MSI-status as determined by retrospective central testing of baseline tumor tissue |
| • To determine the correlation between ctDNA levels, BRAF V600 alterations, and clinical outcome | • ctDNA levels and BRAF V600 VAF from ctDNA analysis of plasma samples collected at baseline and on treatment<br>• |
| **Tertiary/Exploratory** | |
| • To understand the relationship between the therapeutic intervention(s) being studied and the biology of the participant's disease | • Measurements of biomarkers, consisting of DNA, RNA, protein or defined cell types, resulting from analyses of peripheral blood and/or tumor tissue biospecimen obtained at baseline, on treatment and/or at end-of-study |
| • To understand the surgical conversion rate | • Surgical conversion rate, defined as the rate of participants who become eligible for surgery and undergo surgery with curative intent as a result of study intervention. |

### 2.1.1. Primary Estimands

Estimands are defined below for the primary endpoint of incidence of dose-limiting toxicities (DLTs) in the Safety Lead-in (SLI) as well as the primary and key secondary endpoints in the Phase 3 portion and Cohort 3 of the study.



090177e1a0219102\Approved\Approved On: 08-Mar-2024 15:37 (GMT)

090177e1a0219102\Approved\Approved On: 08-Mar-2024 15:37 (GMT)

**Safety Lead-in**

The primary estimand for the SLI portion of the study is the DLT rate estimated based on data from DLT-evaluable participants during the DLT-evaluation period, which is the first 28 days after the first dose of study intervention in the SLI. It includes the following attributes:

- Population: all participants with local or central laboratory confirmed *BRAF* V600E- metastatic colorectal cancer (mCRC), as defined by the screening inclusion/exclusion criteria in Section 5.1.2 and Section 5.2.2 in the protocol to reflect the targeted population of the SLI portion of the study.

- Variable: DLT rate during the DLT-evaluation period, which is the first 28 days after the first dose of study intervention in the SLI. DLTs are defined in Section 6.1.1.1 in the study protocol.

- Intercurrent events: hypothetical strategy will be applied for the intercurrent events. The intercurrent event is treatment discontinuation for reasons other than treatment-related toxicity that leads to <75% of the planned dose of each study intervention during the DLT evaluation period. Participants without DLTs and with the intercurrent event will not be included in the DLT rate calculation. See Section 4 for more details on the DLT evaluable definition.

- Population-level summary measure: DLT rate defined as the number of DLT-evaluable participants with DLTs in the DLT-evaluation period divided by the number of DLT-evaluable participants.

**Phase 3, Two Primary Estimands**

The primary estimands in the Phase 3 portion of the study are the treatment effect in progression-free survival (PFS) by blinded independent central review (BICR) and in ORR by BICR per Response Evaluation Criteria in Solid Tumors version 1.1 (RECIST v1.1) for Arm B vs Arm C. They are the hypothetical estimands and include the following 4 attributes.

PFS for Arm B vs Arm C:

- Population: all participants who are randomized in the Phase 3 portion of the study in Arm B and Arm C. This analysis population will be based on FAS defined in Section 4.

- Variable: PFS by BICR, defined as the time from the date of randomization to the earliest documented disease progression per RECIST v1.1, or death due to any cause.

- Intercurrent events: Details of the intercurrent events and censoring rules for the primary analysis are summarized in Table 4.

- Population-level summary measure: HR for PFS and corresponding 2-sided 95.4% CI based on Cox proportional hazard model stratified by randomization strata. PFS will be compared between the two treatment arms using a 1-sided stratified log-rank test.

OR for Arm B vs Arm C:

- Population: the first 110 participants who are randomized in the Phase 3 portion of the study into each of Arm B and Arm C (Full Analysis Set, ORR Subset defined in Section 4).

- Variable: Objective response defined as complete response (CR), or partial response (PR) according to RECIST v1.1 based on BICR assessment, from the date of randomization until the date of the first documentation of progression of disease (PD). Both CR and PR must be confirmed by repeat assessments performed no less than 4 weeks after the criteria for response are first met. Only tumor assessments performed on or before the start date of subsequent anticancer therapies will be considered in the assessment.

- Intercurrent events: All data collected after an intercurrent event of subsequent anticancer therapy or first PD will be excluded.

- Population-level summary measure: stratified (by the randomization strata) odds ratio in terms of OR defined as the odds of OR with Arm B divided by the odds of OR with Arm C, with its 2-sided 99.8% CI. ORR treatment effect will be compared using Cochran-Mantel-Haenszel test stratified by the randomization strata.

**Cohort 3**

The primary estimand in the Cohort 3 portion of the study is the treatment effect in OR by BICR per RECIST v1.1 for Arm D vs Arm E. It includes the following 4 attributes:

- Population: all participants who are randomized in Cohort 3. This analysis population will be based on FAS defined in Section 4.

- Variable: Objective response defined as CR, or PR according to RECIST v1.1 based on BICR assessment, from the date of randomization until the date of the first documentation of PD, death or start of new anticancer therapy. Both CR and PR must be confirmed by repeat assessments performed no less than 4 weeks after the criteria for response are first met. Only tumor assessments performed on or before the start date of subsequent anticancer therapies will be considered in the assessment.

- Intercurrent events: All data collected after an intercurrent event of subsequent anticancer therapy or first PD will be excluded.

- Population-level summary measure: stratified (by the randomization strata) odds ratio in terms of OR defined as the odds of OR with Arm D divided by the odds of OR

090177e1a0219102\Approved\Approved On: 08-Mar-2024 15:37 (GMT)

with Arm E, with its 2-sided 95% CI. ORR treatment effect will be compared using Cochran-Mantel-Haenszel test stratified by the randomization strata.

### 2.1.2. Secondary Estimand

**Phase 3**

The key secondary estimand in the Phase 3 portion of the study is the treatment effect in overall survival (OS) for Arm B vs Arm C. It is the hypothetical estimand and includes the following 4 attributes:

- Population: all participants who are randomized in the Phase 3 portion of the study in Arm B and Arm C. This analysis population will be based on FAS defined in Section 4.
- Variable: OS, defined as the time from date of randomization to death due to any cause.
- Intercurrent events: Participants not known to have died are censored at the date of last contact when participant was known to be alive.
- Population-level summary measure: HR for OS and corresponding 2-sided CI based on Cox proportional hazard model stratified by randomization strata. Level of CI will depend on alpha spent for the analysis. OS will be compared between the two treatment arms using a 1-sided stratified log-rank test.

**Cohort 3**

The key secondary estimand in the Cohort 3 portion of the study is the treatment effect in PFS by BICR. It is the hypothetical estimand and includes the following 4 attributes:

- Population: all participants who are randomized in the Cohort 3. This analysis population will be based on FAS defined in Section 4.
- Variable: PFS by BICR, defined as the time from the date of randomization to the earliest documented disease progression per RECIST v1.1, or death due to any cause.
- Intercurrent events: details of the intercurrent events and censoring rules are summarized in Table 4.
- Population-level summary measure: HR for PFS and corresponding 2-sided 95% CI based on Cox proportional hazard model stratified by the randomization strata. PFS will be compared between the two treatment arms using a 1-sided stratified log-rank test.

090177e1a0219102\Approved\Approved On: 08-Mar-2024 15:37 (GMT)

### 2.1.3. Additional Estimands

The following supportive estimands are defined for the Phase 3 and for the Cohort 3 portion of the study:

**Supportive Estimand 1 (OR):**

- Population: all participants with a central laboratory confirmed result of *BRAF* V600E-mutant mCRC, as defined by the screening inclusion/exclusion criteria in Section 5.1.2 and Section 5.2.2 in the study protocol to reflect the targeted population of the Phase 3 portion and Cohort 3 of the study.
- Variable: same as primary estimand of OR.
- Intercurrent events: same as primary estimand of OR.
- Population-level summary measure: same as primary estimand of OR.

**Supportive Estimand 1 (PFS):**

- Population: all participants with a central laboratory confirmed result of *BRAF* V600E-mutant mCRC, as defined by the screening inclusion/exclusion criteria in Section 5.1.2 and Section 5.2.2 in the study protocol to reflect the targeted population of the Phase 3 portion and Cohort 3 of the study.
- Variable: same as primary estimand of PFS.
- Intercurrent events: same as primary estimand of PFS.
- Population-level summary measure: same as primary estimand of PFS.

**Supportive Estimand 2 (PFS):**

- Population: same as primary estimand of PFS.
- Variable: same as primary estimand of PFS.
- Intercurrent events: treatment policy strategy will be applied for the intercurrent event of starting new anticancer therapy. All other intercurrent events will be addressed in the same approach as the primary estimand of PFS. Table 7 summarizes the details of the intercurrent events and censoring rules for this supportive analysis.
- Population-level summary measure: same as primary estimand of PFS.

**Supportive Estimand 3 (OS):**

- Population: all participants with a central laboratory confirmed result of *BRAF* V600E-mutant mCRC, as defined by the screening inclusion/exclusion criteria in Section 5.1.2 and Section 5.2.2 in the study protocol to reflect the targeted population of the Phase 3 portion and Cohort 3 of the study.
- Variable: same as key secondary estimand of OS.
- Intercurrent events: same as key secondary estimand of OS.



090177e1a0219102\Approved\Approved On: 08-Mar-2024 15:37 (GMT)

- Population-level summary measure: same as key secondary estimand of OS.

## 2.2. Study Design

C4221015 study is an open-label, multicenter, randomized Phase 3 study of encorafenib plus cetuximab (EC) with or without chemotherapy vs standard of care chemotherapy in participants with previously untreated *BRAF* V600E-mutant mCRC. It includes a SLI portion, a randomized Phase 3 portion, and a randomized Cohort 3.

Prior to the Phase 3 portion, a SLI will be conducted at a limited number of sites to evaluate the safety/tolerability and PK of EC in combination with either mFOLFOX6 or FOLFIRI. Approximately 60 participants will be enrolled in the SLI portion (up to 30 per regimen). The results of the SLI will inform which chemotherapy regimen is used in Arm B of the Phase 3 portion of the study.

After reviewing the totality of the SLI data, the E-DMC and SC determined that there were no new safety signals and that the study can proceed as planned. As a result of the SLI data review, Arm B will consist of EC in combination with mFOLFOX6. In addition, Phase 3 treatment Arm C will consist of investigator's choice of mFOLFOX6 ± bevacizumab, FOLFOXIRI ± bevacizumab or CAPOX ± bevacizumab.

In the Phase 3 portion, approximately 620 participants will be randomized overall, initially at a ratio of 1:1:1 to receive EC (Arm A), EC + chemotherapy (Arm B) or standard of care (SOC) chemotherapy (Arm C), and then 1:1 to receive EC + chemotherapy (Arm B) or SOC chemotherapy (Arm C) after the approval of Protocol Amendment 5, with a total of approximately 150 participants for Arm A and approximately 235 participants each for Arm B and Arm C. The primary objective in the Phase 3 portion of the study is to compare the efficacy, as measured by the primary endpoints of PFS by BICR and ORR by BICR, of Arm B versus Arm C. Phase 3 portion will be considered positive (ie, demonstrated evidence of effectiveness) if the hypothesis test of either primary endpoint is statistically significant.

With protocol amendment 5, a Cohort 3 with 2 randomized arms will be included. Approximately 135 participants will be enrolled in Cohort 3 at a ratio of 2:1 to receive EC + FOLFIRI (Arm D, n=90) or FOLFIRI with or without bevacizumab (Arm E, n=45). Cohort 3 will start after the enrollment of the Phase 3 portion is complete. The primary objective in the Cohort 3 portion of the study is to compare the efficacy, as measured by the primary endpoint of ORR by BICR, of Arm D versus Arm E. Cohort 3 portion will be considered positive (ie, demonstrated evidence of effectiveness) if the hypothesis test of the primary endpoint is statistically significant.

A schema of the study design is presented in Figure 1.

090177e1a0219102\Approved\Approved On: 08-Mar-2024 15:37 (GMT)

**Figure 1 Study Design**

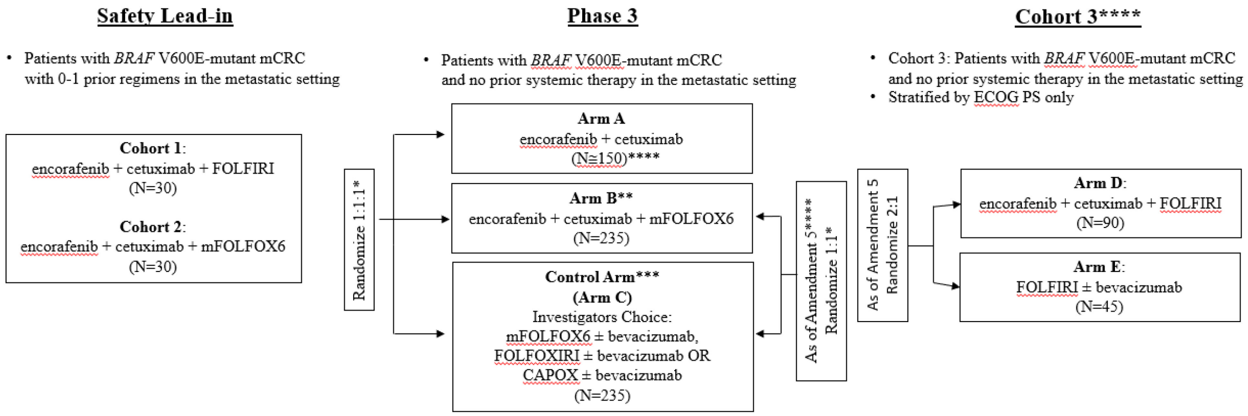


* Stratified by: ECOG PS (0 vs 1) and Region (US/Canada vs Europe vs Rest of World).
** mFOLFOX6 will be used in Arm B based on results of the SLI portion of the study.
*** Since mFOLFOX6 is used in Arm B, the Control Arm will consist of investigator's choice of mFOLFOX6 ± bevacizumab, FOLFOXIRI ± bevacizumab or CAPOX ± bevacizumab.
**** Upon Protocol Amendment 5 approval by site EC/IRB: randomization to Arm A is discontinued; Cohort 3 to begin after Phase 3 enrollment is complete.

# 3. ENDPOINTS AND BASELINE VARIABLES: DEFINITIONS AND CONVENTIONS

## 3.1. Safety Lead-in

### 3.1.1. Primary Endpoint

The primary endpoint for the SLI is incidence of DLTs estimated based on data from DLT-evaluable participants during the DLT-evaluation period, which is the first 28 days after the first dose of study intervention in the SLI. The criteria of DLTs are specified in Section 6.1.1.1 in the protocol.

### 3.1.2. Secondary Endpoints

#### 3.1.2.1. Safety Endpoints

- Incidence and severity of adverse events (AEs) graded according to the National Cancer Institute Common Terminology Criteria for Adverse Events version 4.03 (NCI CTCAE v4.03) and changes in clinical laboratory parameters, vital signs and ECGs.
- Incidence of dose interruptions, dose modifications and discontinuations due to AEs.

#### 3.1.2.2. Efficacy Endpoints

**Objective Response Rate**

Objective response rate (ORR) is defined as the proportion of participants who have achieved a best overall confirmed response (BOR) of complete response (CR) or partial response (PR) per RECIST v1.1. The BOR is the best response obtained among all tumor assessment visits after the date of first dose until documented disease progression, death, or start of subsequent anticancer therapy. Clinical deterioration or clinical progression noted on the completion eCRF will not be considered as documented disease progression.



090177e1a0219102\Approved\Approved On: 08-Mar-2024 15:37 (GMT)

ORR based on investigator assessment will be calculated for the SLI participants.

**Duration of Response**

Duration of response (DOR) is defined as the time from the date of first radiographic evidence of response (CR or PR) to the earliest documented disease progression per RECIST v1.1, or death due to any cause.

DOR based on investigator assessment will be calculated for the SLI participants with a confirmed response (CR or PR).

**Progression-free Survival**

PFS is defined as the time from date of the first dose to the earliest documented disease progression per RECIST v1.1, or death due to any cause.

The censoring and event date options to be considered for the PFS analysis are described in details in Section 6.1.2.1.1.

PFS based on investigator assessment will be calculated for the SLI participants.

**Time to Response**

Time to response (TTR) is defined as the time from the date of first dose to first radiographic evidence of response (CR or PR) per RECIST v1.1. TTR will be calculated for participants with a confirmed response (CR or PR).

TTR based on investigator assessment will be calculated for the SLI participants.

**Overall Survival**

OS is defined as the time from the date of first dose to death due to any cause. If a participant is not known to have died at the time of the cutoff for analysis, then OS will be censored at the date of last contact.

### 3.1.2.3. PK Endpoints

- PK parameters of encorafenib, irinotecan, oxaliplatin and relevant metabolites.
- Changes in exposures of irinotecan and its metabolite (SN-38) on Cycle 1 Day 15 compared to Cycle 1 Day 1 in Cohort 1 (EC + FOLFIRI).
- Changes in exposures of oxaliplatin on Cycle 1 Day 15 compared to Cycle 1 Day 1 in Cohort 2 (EC + mFOLFOX6).

090177e1a0219102\Approved\Approved On: 08-Mar-2024 15:37 (GMT)

#### 3.1.3. Exploratory Endpoints

Measurements of biomarkers, consisting of DNA, RNA, protein or defined cell types, resulting from analyses of peripheral blood and/or tumor tissue biospecimen obtained at baseline, on treatment and/or at end-of-study.

### 3.2. Phase 3

#### 3.2.1. Primary Endpoints

- PFS by BICR is defined as the time from date of randomization to the earliest documented disease progression per RECIST v1.1 based on BICR assessment, or death due to any cause.
  The censoring and event date options to be considered for the PFS primary analysis are described in details in Section 6.1.2.1.1.

- ORR by BICR is defined as the proportion of participants who have achieved a BOR of confirmed (by repeat assessments performed no less than 4 weeks after the criteria for response are first met) CR or PR per RECIST v1.1. The BOR, as assessed by BICR, is the best response obtained among all tumor assessment visits after the date of randomization until documented disease progression, death, or start of subsequent anticancer therapy. Participants who do not have a post-baseline tumor assessment due to early progression of disease, who receive anti-cancer therapies other than the study treatments prior to reaching a CR or PR, or who die, have PD, or stop tumor assessments for any reason prior to reaching a CR or PR will be counted as non-responders in the assessment of OR. Each participant will have an objective response status (0: no OR; 1: OR). Clinical deterioration or clinical progression noted on the completion eCRF will not be considered as documented disease progression.

#### 3.2.2. Efficacy Secondary Endpoints

##### 3.2.2.1. Overall Survival

The key secondary endpoint of OS is defined as the time from the date of randomization to death due to any cause. If a participant is not known to have died at the time of the cutoff for analysis, then OS will be censored at the date of last contact.

##### 3.2.2.2. Objective Response Rate by Derived Investigator

ORR by derived investigator assessment (response will be derived programmatically from the target lesion measurements, non-target lesion status, and new lesions recorded on the CRF, see APPENDIX 2 for details) is defined as the proportion of participants who have achieved a BOR of confirmed (by repeat assessments performed no less than 4 weeks after the criteria for response are first met) CR or PR per RECIST v1.1. The BOR is the best response obtained among all tumor assessment visits after the date of randomization until documented disease progression, death, or start of subsequent anticancer therapy. Clinical deterioration or clinical progression noted on the completion eCRF will not be considered as documented disease progression.

090177e1a0219102\Approved\Approved On: 08-Mar-2024 15:37 (GMT)

#### 3.2.2.3. Duration of Response

DOR in the Phase 3 portion of the study has the same definition of DOR as described in Section 3.1.2.2. Confirmed responses will be used.

Both DOR by BICR and DOR by derived investigator assessment will be calculated.

#### 3.2.2.4. Progression-free Survival by Derived Investigator

PFS by derived investigator assessment is defined as the time from date of randomization to the earliest documented disease progression per RECIST v1.1 based on derived investigator assessment (PD will be derived programmatically from the target lesion measurements, non-target lesion status, and new lesions recorded on the case report form (CRF)), or death due to any cause.

#### 3.2.2.5. Time to Response

TTR is defined as the time from the date of randomization to first radiographic evidence of response (CR or PR) per RECIST v1.1. TTR will be calculated for participants with a confirmed response (CR or PR).

Both TTR by BICR and TTR by derived investigator assessment will be calculated.

#### 3.2.2.6. PFS after Next Line of Treatment (PFS2)

PFS after next line of treatment (PFS2) is defined as the time from the date of randomization to the date of discontinuation of next-line treatment after first objective PD by investigator assessment, to second objective disease progression, or death from any cause, whichever occurs first. Second objective disease progression is progressive disease (PD2) after the start of subsequent anticancer therapy based on investigator assessment.

### 3.2.3. Other Secondary Endpoints

#### 3.2.3.1. Patient-Reported Outcomes Endpoints

Patient-reported outcomes (PROs) as measured by the following instruments: European Organization for Treatment of Cancer Quality of Life Questionnaire (EORTC QLQ-C30), the EuroQol EQ-5D-5L (EQ-5D-5L) and visual analogue scale (VAS), patient global impression of disease severity (PGIS), and patient global impression of change (PGIC).

The EORTC QLQ-C30 is a 30-question survey, which can be grouped into 5 functional domain subscales, including a physical functioning subscale, a role functioning subscale, an emotional functioning subscale, a cognitive functioning subscale and a social functioning subscale. Higher scores on the functional domains are indicative of higher levels of functioning. Oncology related symptoms assessed by the EORTC QLQ-C30 include fatigue (3 items), pain (2 items), nausea and vomiting (2 items), and dyspnea, insomnia, appetite loss, constipation, diarrhea, and financial impact (1 item each). Higher scores are reflective of a greater presence of symptoms.



090177e1a0219102\Approved\Approved On: 08-Mar-2024 15:37 (GMT)

The EQ-5D-5L is a 6-item patient-completed questionnaire designed to assess health status in terms of a single index value or utility score. There are 2 components, a Health State Profile which has individuals rate their level of problems (none, slight, moderate, severe, extreme/unable) in 5 areas (mobility, self-care, usual activities, pain/discomfort, and anxiety/depression), and a VAS in which participants rate their overall health status from 0 (worst imaginable) to 100 (best imaginable). Published weights are available that allow for the creation of a single summary score. Overall scores range from 0 to 1, with lower scores representing a higher level of dysfunction.

The PGIS is a single-item PRO designed to facilitate an anchor-based methodology for establishing meaningful within person change (MWPC) of other PRO-based endpoints such as the EORTC QLQ-C30. There are 4 response options, ranging from “none” to “severe”.

The PGIC is a single-item PRO designed to assess the participant’s overall impression of the degree of change they have experienced since the start of study treatment. There are 5 response options, ranging from “much better” to “much worse”.

#### 3.2.3.2. Pharmacokinetic Endpoints

- Trough plasma concentrations of encorafenib and the metabolite LHY746 in Arm A and Arm B.
- PK parameters of encorafenib and its metabolite LHY746 in participants randomized in mainland China (Arm A and Arm B, as appropriate).

#### 3.2.3.3. Biomarker Endpoints

- Microsatellite instability (MSI)-status as determined by retrospective central testing of baseline tumor tissue.
- ctDNA levels
- BRAF V600 VAF in ctDNA

### 3.2.4. Exploratory Endpoints

Measurements of biomarkers, consisting of DNA, RNA, protein or defined cell types, resulting from analyses of peripheral blood and/or tumor tissue biospecimen obtained at baseline, on treatment and/or at end-of-study.

## 3.3. Cohort 3

### 3.3.1. Primary Endpoint

ORR by BICR is defined as the proportion of participants who have achieved a BOR of confirmed (by repeat assessments performed no less than 4 weeks after the criteria for response are first met) CR or PR per RECIST v1.1. The BOR, as assessed by BICR, is the best response obtained among all tumor assessment visits after the date of randomization until documented disease progression, death, or start of subsequent anticancer therapy.



090177e1a0219102\Approved\Approved On: 08-Mar-2024 15:37 (GMT)

Participants who do not have a post-baseline tumor assessment due to early progression of disease, who receive anti-cancer therapies other than the study treatments prior to reaching a CR or PR, or who die, have PD, or stop tumor assessments for any reason prior to reaching a CR or PR will be counted as non-responders in the assessment of OR. Each participant will have an objective response status (0: no OR; 1: OR). Clinical deterioration or clinical progression noted on the completion eCRF will not be considered as documented disease progression.

### 3.3.2. Efficacy Secondary Endpoints

#### 3.3.2.1. Progression-free Survival

The key secondary endpoint of PFS by BICR is defined as the time from date of randomization to the earliest documented disease progression per RECIST v1.1 based on BICR assessment, or death due to any cause.

PFS by derived investigator assessment is defined as the time from date of randomization to the earliest documented disease progression per RECIST v1.1 based on derived investigator assessment (PD will be derived programmatically from the target lesion measurements, non-target lesion status, and new lesions recorded on the case report form (CRF)), or death due to any cause.

The censoring and event date options to be considered for the PFS primary analysis are described in detail in Section 6.1.2.1.1.

#### 3.3.2.2. Objective Response Rate by Derived Investigator

ORR by derived investigator assessment is defined as described in Section 3.2.2.2.

#### 3.3.2.3. Duration of Response

DOR in the Cohort 3 portion of the study has the same definition of DOR as described in Section 3.1.2.2. Confirmed responses will be used.

Both DOR by BICR and DOR by derived investigator assessment will be calculated.

#### 3.3.2.4. Time to Response

TTR is defined as described in Section 3.2.2.5.

Both TTR by BICR and TTR by derived investigator will be calculated.

#### 3.3.2.5. Overall Survival

OS is defined as described in Section 3.2.2.1.

### 3.3.3. Other Secondary Endpoints

#### 3.3.3.1. Patient-Reported Outcomes Endpoints

Patient-reported outcomes (PROs) as measured by the following instruments: European Organization for Treatment of Cancer Quality of Life Questionnaire (EORTC QLQ-C30), the



090177e1a0219102\Approved\Approved On: 08-Mar-2024 15:37 (GMT)

EuroQol EQ-5D-5L (EQ-5D-5L) and visual analogue scale (VAS), patient global impression of disease severity (PGIS), and patient global impression of change (PGIC), see Section 3.2.3.1 for additional details.

#### 3.3.3.2. Pharmacokinetic Endpoints

Trough plasma concentrations of encorafenib and the metabolite LHY746 in Arm D.

#### 3.3.3.3. Biomarker Endpoints

- Microsatellite instability (MSI)-status as determined by retrospective central testing of baseline tumor tissue.
- ctDNA levels
- BRAF V600 VAF in ctDNA

### 3.3.4. Exploratory Endpoints

Measurements of biomarkers, consisting of DNA, RNA, protein or defined cell types, resulting from analyses of peripheral blood and/or tumor tissue biospecimen obtained at baseline, on treatment and/or at end-of-study.

## 3.4. Baseline Variables

The date of first dose of study treatment is the earliest date of non-zero dosing of any study drug. The date of last dose of study treatment is the latest date of non-zero dosing of any study drug.

For all endpoint assessments in the SLI and all safety assessments in the Phase 3 portion and in Cohort 3 of the study, baseline is defined as the last completed assessment prior to date of first dose of study treatment in the SLI, the Phase 3, and Cohort 3, respectively. If an assessment that is planned to be performed prior to the first dose of study treatment in the protocol is performed on the same day as the first dose of study treatment and the time is unknown, it will be assumed that it was performed prior to study treatment administration and will be considered as baseline assessment.

For efficacy assessments in the Phase 3 portion and in Cohort 3 of the study, baseline is defined as the last assessment prior to randomization. If the assessment that is planned to be performed before randomization per the protocol is performed on the same day as the date of randomization and assessment time point is missing, it will be assumed that it was performed prior to randomization and will be considered as baseline assessment. If no tumor assessment was performed prior to randomization, any tumor assessment conducted between randomization and prior or on the first study intervention date will be considered as baseline tumor assessment.

For PRO the last measurement prior to or on the date of first dose of study treatment will be used as the baseline measurement. If there are no measurements meeting these criteria, then baseline is considered missing.



090177e1a0219102\Approved\Approved On: 08-Mar-2024 15:37 (GMT)

Unscheduled assessments will be used in the determination of baseline. Data reported at the End of Treatment visit are not eligible for baseline selection.

### 3.4.1. Stratification

Randomization for Phase 3 is stratified by the following factors as recorded in the Interactive Response Technology (IRT) system:

- ECOG performance status: 0 vs 1.
- Region: US/Canada vs Europe vs Rest of World.

Randomization for Cohort 3 is stratified by ECOG performance status (0 vs 1) as recorded in the IRT system.

All stratified analysis will utilize the strata defined in the IRT system.

### 3.4.2. Covariates

In addition to the randomization strata, the following baseline characteristics variables will be included as covariates in the Cox regression model to explore the potential prognostic impact on PFS per BICR and OS:

- Age (<65 years vs ≥65 years).
- Gender (Male vs Female).
- Race (White vs non-White).
- Race (Asian vs non-Asian).
- Number of organs involved at baseline (≤2 vs ≥3) by BICR.
- *BRAF* V600E mutation per central assessment (Detected vs Not Detected/Indeterminate).
- Prior anticancer adjuvant/neoadjuvant therapy (yes vs no).
- Removal status of primary tumor (no resection/partial resection vs complete resection).
- Side of tumor (left/right vs left vs right).
- Presence of liver metastases at baseline, based on target and non-target lesion assessment (yes vs no) (based on BICR).



090177e1a0219102\Approved\Approved On: 08-Mar-2024 15:37 (GMT)

090177e1a0219102\Approved\Approved On: 08-Mar-2024 15:37 (GMT)

### 3.5. Safety Endpoints

Safety endpoints will be summarized based on the on-treatment period (defined in Section 5.2.7) unless otherwise specified. Safety data collected outside the on-treatment period will be listed but not summarized.

#### 3.5.1. Adverse Events

An AE is considered a treatment-emergent adverse event (TEAE) if the event starts on or after the first dosing day and time/start time, if collected, but before the last dose +28 days, or start of subsequent anticancer therapy minus 1 day, whichever occurs first. Any events that started prior to the first dose date are not considered TEAEs. If an AE starts on the same day as the first dose date, it will be considered treatment emergent unless the CRF data indicates otherwise via explicitly recording time for AE onset and treatment dosing.

Adverse events will be coded using current Medical Dictionary for Regulatory Activities (MedDRA). Severity of AEs will be graded using the NCI-CTCAE v 4.03 toxicity grading scale.

#### 3.5.2. Laboratory Data

Hematology, chemistry and coagulation tests results will be programmatically graded according to the NCI CTCAE version 4.03 for relevant parameters. Additional details are provided in Section 6.6.4.

#### 3.5.3. Vital Signs Data

Vital signs data includes weight, pulse, systolic blood pressure (BP), and diastolic BP will be summarized. Additional details are provided in Section 6.6.5.

#### 3.5.4. Electrocardiograms Data

Clinically notable ECG values during the on-treatment period will be summarized. Additional details are provided in Section 6.6.6.

## 4. ANALYSIS SETS (POPULATIONS FOR ANALYSIS)

Data for all participants will be assessed to determine if participants meet the criteria for inclusion in each analysis population prior to unblinding and releasing the database and classifications will be documented per standard operating procedures.

| Participant Analysis Set | Description |
|---|---|
| **Enrolled** | All participants who sign the Screening informed consent/assent document. |
| **DLT-Evaluable Analysis Set** | All participants who receive at least 1 dose of study drug in the SLI and either experience DLT during the DLT evaluation period or complete the DLT evaluation period without DLT. |

| Participant Analysis Set | Description |
|---|---|
| | Participants who complete the DLT evaluation period without DLTs before receiving at least 75% of the planned dose of each of the three study drugs (encorafenib, cetuximab, and mFOLFOX6 or FOLFIRI) during the DLT evaluation period for reasons other than treatment-related toxicity are not evaluable for DLT. Examples of that include non-compliance, protocol deviations, and other issues (e.g. car accident, COVID issues). Patients who complete the DLT evaluation period without DLTs and received less than 75% of the planned dose on one or two study drugs (not all three) due to an unrelated toxicity are DLT evaluable.<br><br>Patients who complete the DLT evaluation period and without DLTs who receive less than 75% of the planned dose for one or two study drugs, due to treatment-related toxicity, are DLT evaluable. Also such patients who receive less than 75% of the planned dose for all three study drugs, due to treatment-related toxicity, are also DLT evaluable (since this event would constitute an actual DLT).<br><br>The DLT evaluation period is the first 28 days after the first dose of study intervention.<br><br>Participants in the SLI will be analyzed according to the actual study treatment received. |
| **Full Analysis Set (FAS)** | For participants in the SLI, the FAS includes all participants who receive at least 1 dose of study drug. Note for the SLI, this set plays the role of the safety analysis set. Participants in the SLI will be analyzed according to the actual study treatment received.<br><br>For participants in the Phase 3 portion and Cohort 3, the FAS includes all participants who are randomized in the Phase 3 portion and Cohort 3 of the study, respectively. Participants will be analyzed according to the study treatment assigned at randomization. |
| **Full Analysis Set, ORR Subset** | For primary analysis of ORR/DOR/TTR in the Phase 3 portion of the study, "Full Analysis Set, ORR Subset" comprises the first 110 participants randomized in each Arm B and Arm C. |
| **Central *BRAF* V600E Positive Analysis Set** | All participants who are randomized in the Phase 3 portion and in Cohort 3 of the study with a confirmed central laboratory result of *BRAF* V600E mutation. Participants will be analyzed according to the study treatment assigned at randomization. |
| **Safety Analysis Set** | All participants who receive at least 1 dose of study drug.<br><br>For the Phase 3 portion and Cohort 3 of the study, participants will be analyzed according to the study treatment assigned at |



090177e1a0219102\Approved\Approved On: 08-Mar-2024 15:37 (GMT)

| Participant Analysis Set | Description |
|---|---|
| | randomization unless the incorrect treatment(s) was/were received throughout the dosing period in which case participants will be analyzed according to the actual study treatment received in the first cycle. |
| **PK Analysis Set** | The PK concentration set is defined as all enrolled participants who are treated and have at least 1 analyte concentration.<br><br>The PK parameter analysis set is defined as all enrolled participants treated who have sufficient information to estimate at least 1 of the PK parameters of interest and have no major protocol deviations affecting PK assessment.<br><br>Participants will be analyzed according to the actual study treatment received. |
| **Biomarker Analysis Set** | All participants who are in the safety set and who had at least 1 of the pharmacodynamics or biomarkers evaluated at pre and/or post dose. Analysis sets will be defined separately for tumor tissue-based and blood-based biomarkers and will also be defined separately for non-paired and paired biomarkers.<br><br>The tumor tissue analysis set is defined as participants from the safety analysis set who had a tumor biomarker assessment at baseline.<br><br>The cfDNA analysis set is defined as participants from the safety analysis set who have a blood-based biomarker assessment at baseline.<br><br>The paired cfDNA analysis set is defined as all patients in the safety analysis set who have blood-based biomarker data at baseline and a corresponding post-treatment timepoint (eg, C1D15, C2D1, C2D15, EOT).<br><br>The cfDNA diagnostics concordance analysis set is defined as participants who have both a central tumor tissue result and a central plasma result. |

# 5. GENERAL METHODOLOGY AND CONVENTIONS

## 5.1. Hypotheses and Decision Rules

### 5.1.1. Hypotheses and Sample Size Calculation

The planned sample size of the study is approximately 815 participants including approximately 60 participants in the SLI, approximately 470 participants in the Phase 3



090177e1a0219102\Approved\Approved On: 08-Mar-2024 15:37 (GMT)

portion of the study (Arm B and Arm C), and approximately 135 participants in Cohort 3. In addition, up to the time of Protocol Amendment 5 approval, approximately 150 participants are estimated to be randomized in Arm A of the Phase 3 portion of the study.

#### 5.1.1.1. Safety Lead-in

There is no statistical hypothesis for the SLI portion of the study.

The primary endpoint of the SLI is incidence of DLTs. Up to 60 participants will be enrolled on a rolling basis in an alternating manner to receive either EC + mFOLFOX6 or EC + FOLFIRI (up to 30 participants per cohort). The safety data will be evaluated by the Steering Committee and External Data Monitor Committee (E-DMC) after the first 9 evaluable participants in each cohort have been followed for a minimum of 28 days with EC + mFOLFOX6 or EC + FOLFIRI. The committees will also review the cumulative safety and PK data at the end of the SLI when all evaluable participants in each of the two cohorts have been followed for a minimum of 28 days.

#### 5.1.1.2. Phase 3

A total of approximately 620 participants will be randomized, initially in a 1:1:1 ratio, stratified by ECOG performance status (0 vs 1) and region (US/Canada vs Europe vs Rest of World), to one of the three treatment arms, and then 1:1 to receive EC + chemotherapy (Arm B) or SOC chemotherapy (Arm C) after the approval of Protocol Amendment 5, with a total of approximately 150 participants for Arm A and 235 participants each for Arm B and Arm C. It is assumed that participant accrual will reach a maximum of 36 participants per month for an accrual duration of approximately 26 months.

**Primary Endpoint**

The primary objective in the Phase 3 portion of the study is to compare the efficacy, as measured by the primary endpoints of PFS by BICR and ORR by BICR, of Arm B versus Arm C. The following statistical hypotheses will be tested to address the primary objective:

$H_{01}$: $HR_{PFS\ (B\ versus\ Control)} \geq 1$ vs $H_{11}$: $HR_{PFS\ (B\ versus\ Control)} < 1$

$H_{02}$: $OR_{OR\ (B\ versus\ Control)} \leq 1$ versus $H_{12}$: $OR_{OR\ (B\ versus\ Control)} > 1$

where $HR_{PFS\ (B\ versus\ Control)}$ is the HR for PFS of Arm B versus the Control Arm and $OR_{OR\ (B\ versus\ Control)}$ is the odds ratio for objective response of Arm B versus the Control Arm.

Approximately 230 PFS events by BICR will be required to have at least 85% power to detect a hazard ratio of 0.67 using a one-sided stratified log-rank test at a significance level of 0.023.

The sample size of 235 participants per arm was determined based on the assumption of a hazard ratio of 0.67 under the exponential model assumptions with median PFS of 7 months on the control arm and 10.4 months on Arm B. Further assumptions include a drop-out hazard rate of 0.07 for each treatment arm and a non-uniform participants accrual over 26 months (for 2 arms: 6 participants per month for the first 5 months, 11 participants a month



090177e1a0219102\Approved\Approved On: 08-Mar-2024 15:37 (GMT)

for months 6 through 7, 16 participants a month for months 8 through 11, 23 participants a month for months 12 through 16, and 24 participants per month thereafter).

The sample size of 220 participants (110 per arm) will provide 90% power for test of odds ratio of 2 proportions between the Arm B and Arm C using a 1-sided chi-square test at a significance level of 0.001 assuming an ORR by BICR of 35% and 65% for Arm C and Arm B, respectively.

**Key Secondary Endpoint**

The key secondary objective is to compare the efficacy, as measured by the key secondary endpoint of OS, of Arm B versus Arm C. The following statistical hypothesis will be tested to address the key secondary objectives:

$H_{03}$: $HR_{OS\ (B\ versus\ Control)} \geq 1$ vs $H_{13}$: $HR_{OS\ (B\ versus\ Control)} < 1$

where $HR_{OS\ (B\ versus\ Control)}$ is the HR for OS of Arm B versus the Control Arm.

For the comparison of OS for Arm B versus the Control Arm, 297 OS events will achieve 85% power to detect an OS HR of 0.70 using a 1-sided stratified log-rank test at a significance level of 0.023, and a 2-look group-sequential design with a Lan-DeMets (O'Brien-Fleming) spending function to determine the efficacy boundary[1]. An exponential distribution for OS was assumed, that corresponds to an improvement in the median OS of 6.4 months (21.4 months in Arm B versus 15 months in the Control Arm). The sample size further assumes a dropout hazard rate of 0.011 within each treatment arm, a non-uniform participants accrual over 26 months (same accrual over time as assumed for the primary endpoint), interim analyses after 80% of OS events, and follow-up after the last participant is randomized of about 24 months.

### 5.1.1.3. Cohort 3

A total of approximately 135 participants will be randomized in a 2:1 ratio, stratified by ECOG performance status (0 vs 1), to receive EC + FOLFIRI (Arm D, n=90) or FOLFIRI with or without bevacizumab (Arm E, n=45). It is assumed that participant accrual will reach a maximum of 20 participants per month for an accrual duration of approximately 7 months.

**Primary Endpoint**

The primary objective in Cohort 3 is to compare the efficacy, as measured by ORR by BICR, of Arm D versus Arm E. The following statistical hypothesis will be tested:

$H_{01}$: $OR_{OR\ (D\ versus\ E)} \leq 1$ versus $H_{11}$: $OR_{OR\ (D\ versus\ E)} > 1$

where $OR_{OR\ (D\ versus\ E)}$ is the odds ratio for objective response of Arm D versus Arm E.

The sample size of 135 participants (90 in Arm D and 45 in Arm E) will provide 90% power for test of odds ratio of 2 proportions between the Arm D and Arm E using a 1-sided chi-square test at a significance level of 0.025 assuming an ORR by BICR of 35% and 65% for Arm E and Arm D, respectively.



090177e1a0219102\Approved\Approved On: 08-Mar-2024 15:37 (GMT)

090177e1a0219102\Approved\Approved On: 08-Mar-2024 15:37 (GMT)

**Key Secondary Endpoint**

The key secondary objective is to compare the efficacy, as measured by the key secondary endpoint of PFS by BICR, of Arm D versus Arm E. The following statistical hypothesis will be tested to address the key secondary objective:

$H_{02}$: $HR_{PFS\ (D\ versus\ E)} \geq 1$ versus $H_{12}$: $HR_{PFS\ (D\ versus\ E)} < 1$

where $HR_{PFS\ (D\ versus\ E)}$ is the hazard ratio for PFS of Arm D versus Arm E

For the comparison of Arm D versus Arm E PFS, 135 participants and 82 PFS by BICR events in Arm D + Arm E will achieve 80% power to detect a PFS HR of 0.519 using a 1-sided stratified log-rank test at a significance level of 0.025. An exponential distribution for PFS was assumed, that corresponds to an improvement in the median PFS of 6.5 months (13.5 months in Arm D versus 7 months in Arm E). The sample size further assumes a dropout hazard rate of 0.015 within each treatment arm, uniform participants accrual of 20 participants a month, and follow-up after the last participant is randomized of about 14 months.

### 5.1.2. Decision Rules

#### 5.1.2.1. Safety Lead-in

The target DLT rate in the first 28 days in the SLI is <33% (ie, <3 out of 9 evaluable participants with DLTs).

Table 2 provides a comparison of the characteristics of this DLT evaluation rule and the traditional 3 + 3 rules based on 9 participants.

**Table 2 Characteristics of DLT Probability During the DLT Evaluation Period**

| True DLT Rate During DLT Evaluation Period | Probability of Dose Declared Toxic Using 3 + 3 Rules | Probability of Observed DLT Rate ≥33% in 9 Participants During DLT Evaluation Period |
|---|---|---|
| 10% | 0.094 | 0.053 |
| 20% | 0.291 | 0.262 |
| 30% | 0.506 | 0.537 |
| 40% | 0.691 | 0.768 |
| 50% | 0.828 | 0.910 |

The DLT evaluation period is the first 28 days after the first dose of study intervention in the SLI, and the DLT-evaluable is defined in Section 4.

If the doses are determined to be tolerable in the first 9 evaluable participants in either or both regimens based on the observed DLT rate (<33%) and evaluation of the overall toxicity profile, the SLI will be expanded for a total of up to 30 evaluable participants in each cohort. If the DLT rate is ≥33% in either cohort, that combination regimen will not be evaluated further in the SLI and will not be used in the Phase 3. Table 3 summarizes the probability of

observing at least one instance of a toxicity that has a true incidence rate of 10% and 15% for the given number of participants.

**Table 3 Probability of Observing ≥ 1 Instance of Toxicity at 10% and 15% Incidence Rate**

| True Toxicity Incidence Rate | Number of Participants | Probability of Observing ≥1 Instance of Toxicity |
|---|---|---|
| 10% | 15 | 0.79 |
| | 20 | 0.88 |
| | 30 | 0.96 |
| 15% | 15 | 0.91 |
| | 20 | 0.96 |
| | 30 | 0.99 |

If any participant is deemed non-evaluable for DLT, additional participants may be enrolled to ensure there are a sufficient number of evaluable participants in the SLI.

#### 5.1.2.2. Phase 3

For the Phase 3 portion of the study a hierarchical testing procedure will be used to control the family-wise type I error rate[2].

Eight months after randomization of the first 110 participants each in Arm B and in Arm C, or after the completion of enrollment of the Phase 3 portion of the study, whichever occurs later, the ORR by BICR will be compared for Arm B versus Arm C, on these first 220 participants, using a 1-sided alpha of 0.001.

If the results of ORR by BICR analysis are statistically significant, an interim analysis for OS on all participants will be conducted at this time using a portion of nominal 1-sided alpha of 0.001.

Once at least 230 PFS events by BICR will be observed for Arm B + Arm C and at least 12 months after the completion of enrollment of the Phase 3 portion of the study, the PFS analysis for the comparison of Arm B versus Arm C will be tested using a 1-sided alpha of 0.023.

If the results of PFS by BICR analysis are statistically significant, an interim analysis for efficacy OS will be conducted at this time using a portion of nominal 1-sided alpha of 0.023. The boundaries for the efficacy analysis will be derived using a Lan-DeMets alpha-spending function[1] that approximates O'Brien-Fleming stopping boundaries for overall type I error rate at 0.023 level (1-sided).

If the results of PFS by BICR analysis are not statistically significant, but the ORR by BICR on 220 participants resulted statistically significant, an interim analysis for efficacy OS will be conducted at the time of PFS analysis using a portion of nominal 1-sided alpha of 0.001.



090177e1a0219102\Approved\Approved On: 08-Mar-2024 15:37 (GMT)

If the value of the test statistic for interim OS of Arm B versus Arm C at the time of PFS analysis exceeds the efficacy boundary no further test will be performed.

If the results of the interim analysis of OS are not statistically significant, a final OS analysis will be performed once 297 events are observed for Arm B + Arm C. Details of the OS interim efficacy analysis are described in Section 7.2. Figure 2 outlines the steps of multiple testing strategy.

**Figure 2 Multiple Testing Strategy**

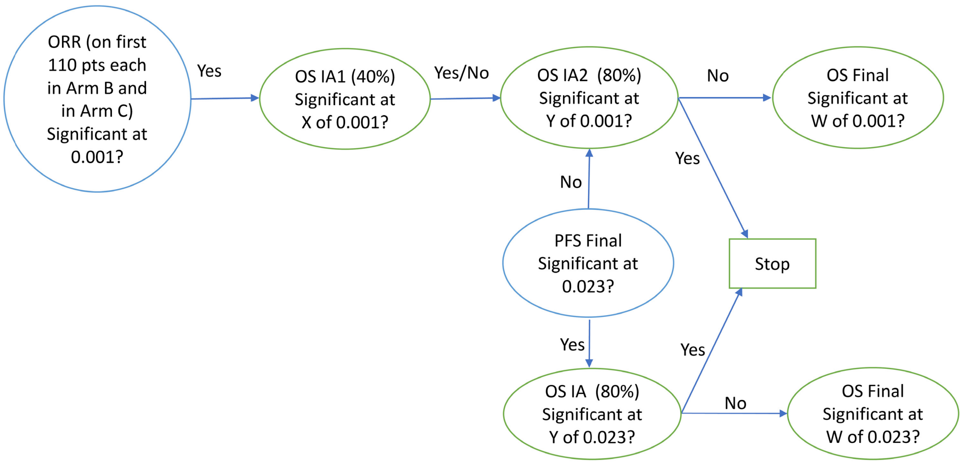


Proportions of events for OS Interim Analyses are shown only for illustration purposes, since the observed number of events at IAs may be different from planned;
X, Y and W will be determined based on the proportion of information fraction at the time of OS Interim Analyses
STOP means no additional test for OS

The overall significance level for Phase 3 portion of the study is 0.025 (1-sided) and will be used as follows:

- 1-sided alpha of 0.001 for the comparison of ORR by BICR of Arm B versus Arm C;
- 1-sided alpha of 0.023 for the comparison of PFS by BICR of Arm B versus Arm C;
- alpha for the comparison of OS of Arm B versus Arm C will depend on the outcome of ORR and PFS;
- 1-sided alpha of 0.001 for the comparison of PFS by BICR of Arm A versus Arm C, even if there is no plan for formal testing.

No formal statistical testing will be performed on the secondary and exploratory endpoints (with the exception of the key secondary endpoint described above), sensitivity analyses and subgroup analyses. These results will be reported with descriptive statistics only.



090177e1a0219102\Approved\Approved On: 08-Mar-2024 15:37 (GMT)

Confidence intervals will be 2-sided with a confidence level of 95%, if not otherwise specified.

#### 5.1.2.3. Cohort 3

In Cohort 3, the analysis of ORR by BICR comparing Arm D versus Arm E will be performed at least 8 months after the randomization of the last participant using 1-sided alpha of 0.025. A hierarchical testing procedure will be used to control the family-wise type I error rate at 0.025 (1-sided) level for ORR and PFS. PFS by BICR will be tested when at least 82 events will be observed, only if the analysis for ORR is statistically significant. If ORR results are not statistically significant, PFS will be analyzed descriptively.

No formal statistical testing will be performed on the secondary and exploratory endpoints (with the exception for key endpoint described above), sensitivity analyses and subgroup analyses. These results will be reported with descriptive statistics only.

Confidence intervals will be 2-sided with a confidence level of 95%, if not otherwise specified.

## 5.2. General Methods

Unless otherwise specified, all analyses will be performed separately for the SLI, for the Phase 3 portion, and for the Cohort 3 of the study.

In general, descriptive summaries will be presented for the efficacy and safety variables collected. Continuous variables will be summarized using mean, standard deviation, minimum, median, and maximum. Categorical variables will be summarized using frequency counts and percentages.

All statistical analyses will be conducted using SAS®, version 9.4 or higher (SAS Institute, Inc.). Noncompartmental PK analyses will be performed using an internally validated software system, openNCA. Sample size calculation for the primary and key secondary efficacy endpoints were performed with East version 6.5 (Cytel)[3].

### 5.2.1. Data Handling After the Cut-off Date

Data after the cut-off date may not undergo the cleaning process and will not be displayed in any listings or used for summary statistics, statistical analyses or imputations.

### 5.2.2. Pooling of Data by Center

In order to provide overall estimates of treatment effects, data will be pooled across trial centers. The “center” factor will not be considered in statistical models or for subgroup analyses due to the high number of participating centers in contrast to the anticipated small number of participants randomized at each center.

### 5.2.3. Analyses to Assess the Impact of COVID-19 Pandemic

The study enrollment started during the COVID-19 pandemic period. The following data summaries and analyses may be performed to assess the impact of COVID-19 on the trial population and study data. Additional analyses may be added in a SAP amendment if they



090177e1a0219102\Approved\Approved On: 08-Mar-2024 15:37 (GMT)

are considered necessary to evaluate the outcome of the trial. Details of these summaries and analyses are included in the respective sections.

- A listing of all participants affected by COVID-19 related study disruption.
- A listing of protocol deviations related to COVID-19.
- COVID-19 related AEs and deaths.
- Summary of missing tumor assessments due to COVID-19.

### 5.2.4. COVID-19 Anchor Date

An anchor date will be used as a start date for COVID-19 pandemic related periods based on Pfizer guidance and standard operating procedure (SOP):

- For global pandemic reference date: Use the date the World Health Organization designated COVID-19 as a global pandemic - March 11, 2020.
- For China reference date: Use the date COVID-19 was identified as the causative agent of outbreak in Wuhan by the China Center for Disease Control and Prevention - January 9, 2020.

A different anchor date may be used for purposes of regulatory submission should the regulatory authority requests.

### 5.2.5. Definition of Start Date

The start date is defined as date of the first dose of any study drug for the SLI and date of randomization for the Phase 3 portion and for the Cohort 3 of the study.

### 5.2.6. Definition of Study Day

The study day for assessments occurring on or after the first dose of study treatment (eg, adverse event onset, laboratory date) will be calculated as:

Study day = Date of the assessment/event - start date of study treatment +1.

The study day for assessments occurring prior to the first dose of study treatment (eg, baseline characteristics, medical history) will be negative and calculated as:

Study day = Date of the assessment/event - start date of study treatment.

For efficacy endpoints and for tumor assessments in the Phase 3 portion and in the Cohort 3 the study day will be calculated respect to the randomization date and not to the date of first dose of study treatment.

The study day will be displayed in all relevant data listings.

### 5.2.7. On-treatment Period

The on-treatment period is defined as the time from the first dose of study treatment through minimum of (last dose of study treatment +28 days, start day of subsequent anticancer systemic therapy - 1 day).



090177e1a0219102\Approved\Approved On: 08-Mar-2024 15:37 (GMT)

### 5.2.8. Definition of Start of New Anti-cancer Drug Therapy

Start date of new anti-cancer systemic therapy is used to determine the end of the on-treatment period (see Section 3.5).

The start date of new anti-cancer drug therapy is the earliest start date of anti-cancer drug therapy recorded in the 'Follow-up Cancer Therapy' eCRF pages that is after the first dose of study treatment. When start date of anti-cancer drug therapy is missing or partially missing, the imputation rules described in Section 5.3.1 should be applied using only data from the 'Follow-up Cancer Therapy' eCRF pages.

### 5.2.9. Definition of Start of New Anti-cancer therapy

Start date of new anti-cancer therapy (drug, radiation, surgery, including metastasectomy) is used for censoring in efficacy analyses (see Section 6.1.2.1.1).

The start date of new anti-cancer therapy is the earliest date after randomization amongst the following:

- Start date of anti-cancer drug therapy recorded in the 'Follow-up Cancer Therapy' eCRF pages
- Start date of radiation therapy recorded in 'RADIATION TREATMENT', 'RADIATION TREATMENT FOR PHASE 3' eCRF pages with 'Subcategory Intent' = 'Curative in intent'
- Surgery date recorded in 'NON-DRUG TREATMENTS - CANCER SURGERY', 'NON-DRUG TREATMENTS - CANCER SURGERY FOR PHASE 3', eCRF pages when 'Outcome of Procedure' = 'Resected' or 'Partially Resected'.

When start date of anti-cancer therapy is missing or partially missing, the imputation rules described in Section 5.3.1 should be applied using 'Follow-up Cancer Therapy', 'RADIATION TREATMENT', 'RADIATION TREATMENT FOR PHASE 3, 'NON-DRUG TREATMENTS - CANCER SURGERY', 'NON-DRUG TREATMENTS - CANCER SURGERY FOR PHASE 3' eCRF pages.

### 5.2.10. Date of Last Contact

The date of last contact will be derived for participants not known to have died at the data cutoff date using the latest complete date (ie, imputed dates will not be used in the derivation) among the following:

- All participant assessment dates (eg, blood draws (laboratory, Pharmacokinetics (PK), vital signs, performance status, ECG, tumor assessments);
- Start and stop dates of concomitant therapies including non-drug treatments or procedures;
- Completion dates for PRO Questionnaires;



090177e1a0219102\Approved\Approved On: 08-Mar-2024 15:37 (GMT)

- Start and end dates of anticancer therapies administered after study treatment discontinuation including systemic therapy, radiation, and surgeries;
- AE start and end dates;
- Last date of contact collected on the 'Survival Follow-up' eCRF (do not use date of survival follow-up assessment unless status is 'alive' or 'subject remains in follow-up');
- Study treatment start and end dates;
- Randomization and enrollment date; and
- Date of discontinuation on disposition eCRF pages (do not use if reason for discontinuation is lost to follow-up or death).

Only dates associated with actual examinations of the participant will be used in the derivation. Dates associated with a technical operation unrelated to participant status such as the date a blood sample was processed or dates data were entered into the eCRF will not be used. For participants with assessment dates after the data cutoff date the last contact date will be derived as the cutoff date for the analysis.

### 5.2.11. Unscheduled Assessments

Unless otherwise specified, unscheduled assessments will not be displayed in summary tables by nominal visit/timepoint. Unscheduled assessments will be used when deriving baseline and worst case on-treatment for safety and PRO analyses (except where noted for baseline ECGs). Additionally, unscheduled tumor assessments will be used for efficacy analyses (eg, defining date of progression/censoring, best overall response, date of last contact).

### 5.2.12. Tumor Assessment Date

If there are multiple scan dates associated with a tumor evaluation, ie, radiological assessments occur over a series of days rather than the same day, the earliest scan date associated with the evaluation will be used as the date of the assessment. The same convention should be used for investigator, derived investigator and BICR assessments, noting however that in the event that the BICR provides the date of PD, that date should be used instead of deriving the date of PD.

### 5.2.13. Adequate Baseline Tumor Assessment

An adequate baseline tumor assessment is defined as follows (see additional details in Section 10.1.1):

- Baseline assessments of all lesions (target, and non-target) must be prior to first dose date (SLI) or date of randomization (Phase 3 and Cohort 3) per Schedule of Activities in Section 1.3 of the protocol. For Phase 3 and Cohort 3 lesions assessed after randomization, but before or on the first dose date will also be considered adequate.



090177e1a0219102\Approved\Approved On: 08-Mar-2024 15:37 (GMT)

- All lesions (target and non-target) must have non-missing assessments.

  **Note:** for target lesions, an actual measurement should be recorded and it should meet the criteria for being measurable and for non-target lesions the actual status at baseline should indicate that the lesion was present.

### 5.2.14. Adequate Post-baseline Tumor Assessment

An adequate post-baseline tumor assessment is defined as an assessment where a response of CR, PR, SD, non-CR/non-PD, or PD can be determined. Time points where the response is NE, ND (no disease at baseline) or no assessment was performed will not be used for determining the censoring date.

### 5.2.15. Standard Derivations and Reporting Conventions

The following conversion factors will be used to convert days into weeks, months or years: 1 week = 7 days, 1 month = 30.4375 days, 1 year = 365.25 days.

Percentages will be reported to one decimal place. The rounding will be performed to closest integer/first decimal using the common mid-point between the two consecutive values. Eg, 5.1 to 5.4 will be rounded to an integer of 5, and 5.5 to 5.9 will be rounded to an integer of 6.

### 5.2.16. Analyses for Binary and Categorical Endpoints

Binary and categorical endpoints will be summarized by frequency counts and percentages along with corresponding 2-sided Wilson score[4] 95% CI. Unless otherwise specified, the calculation of proportions will include the missing category. Therefore counts of missing observations will be included in the denominator and presented as a separate category.

In case the analysis refers only to certain visits, percentages will be based on the number of participants with an assessment at that visit, unless otherwise specified.

### 5.2.17. Analyses for Continuous Endpoints

Continuous endpoints will be summarized using descriptive statistics ie, number of non-missing values and number of missing values [ie, n (missing)], mean, median, standard deviation, minimum, maximum and first and third quartile (Q1 and Q3). PK summaries will also include coefficient of variation percent (%CV).

### 5.2.18. Analyses for Time-to-Event Endpoints

The stratified log-rank test will be used for comparing treatments. Hazard ratios and the associated 2-sided 95% confidence intervals (CIs) are estimated by stratified Cox proportional hazards model, including treatment as a covariate. To account for the required alpha spending of the group sequential design, 2-sided repeated CIs[5] will also be provided.

Time to event endpoints will be summarized using the Kaplan-Meier method and estimated survival curves will be displayed graphically when appropriate. Graphs will describe the number of participants at risk over time. The median, quartiles, and probabilities of an event at particular points in time will be estimated. CIs for medians and quartiles are based on the



090177e1a0219102\Approved\Approved On: 08-Mar-2024 15:37 (GMT)

Brookmeyer-Crowley[6] method. CIs for the estimated probability of event at a particular timepoint will be generated using the log(-log) method with back transformation to a confidence interval on the untransformed scale. Summaries of the number and percentages of participants with an event and reason for censoring will also be provided on summary tables and/or figures.

**Assessment of proportional hazards**

Schoenfeld residuals for the stratified Cox proportional regression model may be plotted to investigate graphically violations from the proportional hazards (PH) assumption; a non-zero slope is evidence of departure from PH. The PH assumption may be formally tested using Schoenfeld's residual test (Schoenfeld, 1980[7], Therneau & Grambsch, 2000[8]). Large departures from PH will be evidenced by a p-value <0.05. Note however that the test will not be sensitive to detect non-linear deviations from PH.

In addition, the PH assumption will be checked visually within each stratum by plotting

$$\log(-\log(S(t))) \text{ vs } \log(t),$$

where S(t) is the estimated survival function at time t.

**Restricted Mean Survival Time**

The restricted mean survival time (RMST) methodology (Zhang, 2013)[9] is applicable independently of the PH assumption and can be used, at a minimum, as a sensitivity analysis to explore the robustness of the primary analysis result on time-to event endpoint.

In particular, as it pertains to the **cut-off point (τ)** to evaluate the RMST it is noted that the cut-off point should not exceed the minimum of the largest observed time for both treatment arms so that the RMST of all treatment arms being evaluated can be adequately estimated and comparison between treatments is feasible; τ should be clinically meaningful and closer to the end of the study follow-up so that the majority of survival outcomes will be covered by the time interval. The RMST up to time τ can be interpreted as the expected survival time restricted to the common follow-up time τ among all participants. Analyses will be repeated using the following criteria to define τ:

- $\tau_1$ = minimum of (largest observed survival time for experimental arm, largest observed survival time for control arm);
- $\tau_2$ = minimum of (largest survival event time for experimental arm, largest survival event time for control arm);
- $\tau 3$ = midpoint between $\tau 1$ and $\tau 2$.

RMST can be estimated consistently by the area under the Kaplan-Meier curve over [0, τ].

The treatment effect between the experimental arm and the control arm will be assessed based on the difference in RMST. The associated 95% CI for the difference in means and 1-sided p-value will be generated.



090177e1a0219102\Approved\Approved On: 08-Mar-2024 15:37 (GMT)

## 5.3. Methods to Manage Missing Data

Unless otherwise specified, all data will be evaluated as observed, and no imputation method for missing values will be used.

Any imputations will occur at the analysis dataset level. Additionally, in all participant data listings imputed values will be presented and flagged as imputed.

Missing statistics, eg, when they cannot be calculated, should be presented as 'ND' for not done, 'NR' for not reached or 'NA' for not applicable. For example, if N=1, the measure of variability cannot be computed and should be presented as 'ND' or 'NA'.

### 5.3.1. Missing Dates

For purposes of data listings, dates will reflect only the information provided by the investigator on the eCRF.

If start dates for adverse events or concomitant medications are completely missing a worst case approach will be taken whereby the events will be considered treatment emergent and the medications will be considered concomitant. If only partial information are available (eg, only a month and year or only a year) and the partial information provide sufficient information to indicate the dates are prior to the start of study treatment (eg, month/year less than month/year of first dose) then these will be considered to have started prior to treatment; otherwise a similar worst case approach will apply and these will be considered to have started after treatment.

**Missing or Partial Death Dates**

Missing or partial death dates will be imputed based on the last contact date:

- If the entire date is missing it will be imputed as the day after the date of last contact (see derivation of date of last contact in Section 5.2.10).
- If the day or month is missing, death will be imputed to the maximum of the full (non-imputed) day after the date of last contact and the following:
    - Missing day: 1st day of the month and year of death, or
    - Missing day and month: January 1st of the year of death.

**Date of First and Last Dose of Study Treatment**

No imputation will be done for first dose date.

Date of last dose of study drug for encorafenib and capecitabine, if unknown or partially unknown, will be imputed as follows:

- If the last date of study drug is completely missing and there is no End of Treatment eCRF page and no death date, the participant should be considered to be ongoing and



090177e1a0219102\Approved\Approved On: 08-Mar-2024 15:37 (GMT)

use the cutoff date for the analysis as the last dosing date. **Note:** the study team should confirm that the participant is actively receiving dose at the time of the data cutoff.

- If the last date of study drug is completely or partially missing and there is EITHER an End of Treatment eCRF page OR a death date available (within the data cutoff date), then impute as follows:
    - If only Year is available and Year < Year of min (EOT date, death date), impute the date as 31DECYYYY.
    - If both Year and Month are available and Year = Year of min (EOT date, death date) and Month < Month of min (EOT date, death date), impute as last day of the month.
    - For all other cases, impute as min (EOT date, death date).

For 5-FU infusion, if unknown, the date of last dose is assumed equal to the last dose start date plus 2 days. For all other study drugs administered by infusion or bolus the date of last dose is assumed equal to the last dose start date.

**Date of Start of New Anticancer Therapy**

Incomplete dates for start date of new anticancer therapy (systemic therapy, radiation and surgery, etc.) will be imputed as follows and will be used for determining censoring dates for efficacy analyses. PD date below refers to PD date by investigator assessment (as reported on Investigator Overall Tumor Assessment CRF page). If the imputation results in an end date prior to the imputed start date then the imputed start date should be set to the end date.

- The end date of new anticancer therapy will be included in the imputations for start date of new anticancer therapy. If the end date of new anticancer therapy is:
    - completely missing then it will be ignored in the imputations below;
    - partially missing with only year (YYYY) available then the imputations below will consider 31DECYYYY as the end date of the new anticancer therapy;
    - partially missing with only month and year available then the imputations below will consider the last day of the month for MMMYYYY as the end date of the new anticancer therapy.
- For participants who have not discontinued study treatment at the analysis cut-off date, last dose of study treatment is set to the analysis cut-off date in the imputations below.



090177e1a0219102\Approved\Approved On: 08-Mar-2024 15:37 (GMT)

- If the start date of new anticancer therapy is completely or partially missing then the imputed start date of new anticancer therapy is derived as follows:

  - Start date of new anticancer therapy is completely missing:

    Imputed start date = min [max(PD date +1, last dose of study treatment +1), end date of new anticancer therapy]

  - Only year (YYYY) for start of anticancer therapy is available:

    IF YYYY < Year of min [max(PD date +1, last dose of study treatment +1), end date of new anticancer therapy] THEN imputed start date = 31DECYYYY;

    ELSE IF YYYY = Year of min [max(PD date +1, last dose of study treatment +1), end date of new anticancer therapy]

    THEN imputed start date = min [max(PD date +1, last dose of study treatment +1), end date of new anticancer therapy]

    ELSE IF YYYY > Year of min [max(PD date +1, last dose of study treatment +1), end date of new anticancer therapy]

    THEN imputed start date = 01JANYYYY

  - Both Year (YYYY) and Month (MMM) for start of anticancer therapy are available

    IF

      YYYY = Year of min [max(PD date +1, last dose of study treatment +1), end date of new anticancer therapy], AND

      MMM < Month of min [max(PD date +1 day, last dose of study treatment +1 day), end date of new anticancer therapy]

    THEN

      imputed start date = DAY (Last day of MMM) MMM YYYY ;

    ELSE IF

      YYYY = Year of min [max(PD date +1, last dose of study treatment +1), end date of new anticancer therapy], AND

      MMM = Month of min [max(PD date +1 day, last dose of study treatment +1 day), end date of new anticancer therapy]

    THEN

      imputed start date = min [max(PD date +1 day, last dose of study treatment +1 day), end date of new anticancer therapy]);

    ELSE IF

      YYYY = Year of min [max(PD date +1, last dose of study treatment +1), end date of new anticancer therapy], AND



090177e1a0219102\Approved\Approved On: 08-Mar-2024 15:37 (GMT)

MMM > Month of min [max(PD date +1 day, last dose of study treatment +1 day), end date of new anticancer therapy]

THEN

imputed start date = 01 MMM YYYY;

ELSE IF

YYYY < Year of min [max(PD date +1, last dose of study treatment +1), end date of new anticancer therapy]

THEN

imputed start date = DAY (Last day of MMM) MMM YYYY;

ELSE IF

YYYY > Year of min [max(PD date +1, last dose of study treatment +1), end date of new anticancer therapy]

THEN

imputed start date = 01 MMM YYYY.

**AE Dates**

AE Onset Date:

If completely missing, the onset date will be set to first dose date if the first dose date is less than AE stop date. Otherwise if the first dose date is after AE stop date, then set the onset date to the earliest of non-missing AE stop date or informed consent date.

AE Stop Date:

If completely missing, the stop date will be imputed as the latest of the end of study date, death date, last dose date of the study treatment, or onset date.

Partial AE Date:

Partial AE date will be imputed bases on the imputation rule for "Other Missing or Partial Dates". If the AE onset date is imputed from a partial AE date and the first dose date falls in the same month as the AE onset date, the following will be done:

- The AE onset date is reset to the first dose date.

If AE stop date is imputed, and less than the first dose date, set the AE stop date to the first dose date.

090177e1a0219102\Approved\Approved On: 08-Mar-2024 15:37 (GMT)

**Partial Date of Progression after Subsequent Therapy**

Partial date of progression after subsequent therapy will be imputed as follows:

- If the entire date is missing or if only year is provided, no imputation will be performed.
- If month and year are provided and only the day is missing, the date will be imputed as the 15th of the month.

**Other Missing or Partial Dates**

Imputation methods for other partial dates are as follows:

- If the day of the month is missing for a start date used in a calculation, the 1st of the month will be used to replace the missing date.
- If both the day and month are missing, the first day of the year is used.
- For stop dates, the last day of the month, or last day of the year is used if the day or day and month are missing, respectively.
- If the date is completely missing, no imputation will be performed.

These rules are used unless the calculations result in negative time durations (eg, date of resolution cannot be prior to date of onset). In these cases, the resolution and onset dates will be the same and the duration will be set to 1 day.

### 5.3.2. Missing Patient-Reported Outcomes Data

For the EORTC QLQ-C30 and EQ-5D-5L, missing items will be handled per the respective scoring manuals of each questionnaire. For the PGIS and PGIC, there will be no adjustments for missing data.

### 5.3.3. Missing Pharmacokinetic Data

**Deviations, Missing Concentrations and Anomalous Values**

In summary tables and plots of median profiles, concentrations will be set to missing if one of the following cases is true:

- A concentration has been reported as ND (ie, not done) or NS (ie, no sample);
- A deviation in sampling time is of sufficient concern or a concentration has been flagged as anomalous by the clinical pharmacologist.

Summary statistics will not be presented at a particular timepoint if more than 50% of the data are missing. For analysis of pharmacokinetic concentrations, no values will be imputed for missing data.



090177e1a0219102\Approved\Approved On: 08-Mar-2024 15:37 (GMT)

090177e1a0219102\Approved\Approved On: 08-Mar-2024 15:37 (GMT)

#### 5.3.4. Missing Biomarker Data

No missing data will be imputed.

## 6. ANALYSES AND SUMMARIES

All efficacy analyses will be performed by cohort and number of lines of prior therapies on the FAS for the SLI, and by treatment arm on the FAS for the Phase 3 and for Cohort 3.

### 6.1. Primary Endpoints

#### 6.1.1. Safety Lead-in

The number and proportion of participants experiencing DLTs during the DLT-evaluation period will be summarized by System Organ Class (SOC) and preferred term (PT) based on maximum toxicity grade by treatment cohort. Analyses of DLT will be performed on DLT-Evaluable Set.

All DLTs and their attributes will be presented in data listings sorted by participant identifier, AE and date of onset of the AE.

#### 6.1.2. Phase 3

The primary objective is to compare the efficacy, as measured by the primary endpoints of PFS by BICR and ORR by BICR, of Arm B versus Arm C.

##### 6.1.2.1. PFS by BICR

###### 6.1.2.1.1. Main Analysis

The primary efficacy analysis will compare PFS time by BICR between Arm B and Arm C using a 1-sided stratified log-rank test at the significance level of 0.023. The analysis will be performed based on the FAS when at least 230 PFS events by BICR will be observed in Arms B + C and at least 12 months after the completion of enrollment of the Phase 3 portion of the study. This will allow for adequate follow-up based on PFS assumptions in Arm B. Strata will be based on those specified in the IRT system as described in Section 3.4.1.

PFS will be calculated in months as follows:

PFS (months) = (date of event or censoring – date of randomization +1)/30.4375

Participants without an event or with an event more than 12 weeks (for the first 18 months after randomization) or 16 weeks (after the first 18 months of randomization) after the last adequate tumor assessment that documented no progression will be censored on the date of the last adequate tumor assessment that documented no progression. In addition, if a new anticancer therapy is started prior to an event, the participant will be censored on the date of the last adequate tumor assessment that documented no progression prior to the start of the new anticancer therapy.

Adequate baseline assessment and adequate post-baseline assessment are defined in Section 5.2.13 and Section 5.2.14, respectively.

Participants with no baseline tumor assessment (including participants with an inadequate baseline assessment) or with no adequate post-baseline tumor assessments within 12 weeks after the date of randomization will be censored on the day of randomization, unless the participant dies within 12 weeks of randomization, in which case, death will be an event on date of death.

The censoring and event date options to be considered for the PFS primary analysis are presented in Table 4.

**Table 4 PFS Outcome and Event Dates – Primary Analysis**

| Situation | Date of Progression/Censoring | Outcome |
|---|---|---|
| No adequate baseline assessment, including no disease at baseline | Date of randomization[a] | Censored[a] |
| PD or death ≤12 (or 16)[b] weeks after last adequate tumor assessment or ≤12 weeks after date of randomization | Date of PD or death | Event |
| PD or death >12 (or 16)[b] weeks after the last adequate tumor assessment[c] | Date of last adequate tumor assessment[c] documenting no PD prior to new anticancer therapy, or missed assessments | Censored |
| No PD | | |
| New anticancer therapy given[d] | | |

a If the participant dies ≤12 weeks after date of randomization, the death is an event with date on death date.

b Durations are equal to 2 times the length of the tumor assessment interval, which is 12 weeks for the first 18 months after randomization, and 16 weeks thereafter.

c If there are no adequate post-baseline assessments prior to the PD or death, then the time without adequate assessment should be measured from the date of randomization; if the criteria is met, the censoring will be on the date of randomization.

d New anticancer therapy includes systemic therapy, radiation and surgery

The treatment effect will be estimated using a Cox's proportional hazards model stratified by randomization strata to calculate the HR for PFS of Arm B versus Arm C, along with the corresponding 2-sided 95% CI and 2-sided 95.4% CI.

Kaplan-Meier estimates (product-limit estimates) will be presented by treatment arm together with a summary of associated statistics including the median PFS time with 2-sided 95% CI. The PFS at 3, 6, 9, 12, 15, 18 and 21 months will be estimated with corresponding 2-sided 95% CIs. The CIs for the median will be calculated according to Brookmeyer and Crowley[6] (conftype=linear option in SAS PROC LIFETEST) and the CIs for the survival function estimates at the timepoints defined above will be derived using the log(-log) method according to Kalbfleisch and Prentice[10] (conftype=loglog default option in SAS Proc LIFETEST). The estimate of the standard error will be computed using Greenwood's formula.

090177e1a0219102\Approved\Approved On: 08-Mar-2024 15:37 (GMT)

Frequency (number and percentage) of participants with each event type (PD or death) and censoring reasons will be presented by treatment arm along with the overall event and censor rates.

Reasons for censoring will be summarized according to the categories in Table 5. If a participant meets multiple definitions for censoring the list will be used to define the hierarchy.

**Table 5. PFS Censoring Reasons and Hierarchy - Primary Analysis**

| Hierarchy | Condition | Censoring Reason |
|---|---|---|
| 1 | No adequate baseline assessment, including no disease at baseline | No adequate baseline assessment |
| 2 | Start of new anticancer therapy[a] before event | Start of new anticancer therapy |
| 3 | Event >12 (or 16)[b] weeks from last adequate post-baseline tumor assessment/start date | Event after missing assessments or inadequate assessments[c] |
| 4 | No event and [withdrawal of consent date ≥ date of randomization OR End of study (EOS) = Subject refused further follow-up] | Withdrawal of consent |
| 5 | No event and lost to follow-up in any disposition page | Lost to follow-up |
| 6 | No event and [EOS present OR disposition page for any phase after screening says participant will not continue into any subsequent phase of the study] and no adequate post-baseline tumor assessment | No adequate post-baseline tumor assessment |
| 7 | No event and none of the conditions in the prior hierarchy are met | Ongoing without an event |

a New anticancer therapy includes systemic therapy, radiation and surgery
b Durations are equal to 2 times the length of the tumor assessment interval, which is 12 weeks for the first 18 months after date of first dose (SLI) or date of randomization (Phase 3 and Cohort 3), and 16 weeks thereafter.
c see section 5.2.14 for definition of adequate post baseline tumor assessment

The PFS time or censoring time and the reasons for censoring will also be presented in a patient listing.

Schoenfeld residuals for the stratified Cox proportional model may be plotted to investigate graphically violations from the proportional hazards (PH) assumption (see Section 5.2.18 for details).



090177e1a0219102\Approved\Approved On: 08-Mar-2024 15:37 (GMT)

090177e1a0219102\Approved\Approved On: 08-Mar-2024 15:37 (GMT)

**<u>Time of Follow-Up for PFS by BICR</u>**

A Kaplan-Meier plot for PFS follow-up duration will also be created to assess the follow-up time in each treatment arm using reversed Kaplan-Meier method as shown in Table 6. The Kaplan-Meier estimate of the median PFS follow-up time with its corresponding 2-sided 95% CI will be calculated and presented in the plot.

**Table 6 Reverse Censoring Rules for PFS Follow-up Duration**

| | Date of event/censoring | Censoring |
|---|---|---|
| Participant without a PFS event by BICR | Time from randomization to last tumor assessment scan | No |
| Death or PD | Time from randomization to date of first PD or death | Yes |

### 6.1.2.1.2. Sensitivity/Supplementary Analyses

The following sensitivity and supplementary analyses will be performed to explore the robustness of the primary analysis results and the supportive estimands. These analyses are regarded as purely exploratory.

**<u>Sensitivity Analyses:</u>**

- A stratified analysis will be performed to compare the PFS time by derived investigator assessment.
- An unstratified analysis will be performed to compare the PFS time by BICR.
- PFS by BICR will be analyzed based on Central *BRAF* V600E Positive analysis set.
- PFS by BICR will also be analyzed based on RMST differences (see Section 5.2.18 for details).
- Multivariable Cox regression analysis will be performed to explore the potential influences of baseline participant or disease characteristics on PFS. The baseline characteristics variables specified in Section 3.4.2 will be included as covariates in the Cox regression model in addition to the randomization strata which will be included in the model during the selection procedure.

  A stepwise selection procedure will serve to identify these explanatory variables of potential prognostic values. Variables are entered into and removed from the model in such a way that each forward selection step can be followed by one or more backward elimination steps. The stepwise selection process terminates if no further variable can be added to the model or if the variable just entered into the model is the only variable removed in the subsequent backward elimination. The level of significance for an explanatory variable to enter the model is set to 0.15 (p-value of Score test) and the significance level for removing it is set to 0.40 (p-value of Wald

test). This analysis will be performed using the stepwise selection method in SAS (PROC PHREG). Once this procedure stops, the variable for treatment arm will be added to the last selected model in order to evaluate the effect of treatment on PFS time when adjusted for the selected explanatory variables. The HRs of all selected explanatory variables and of treatment effect will be reported including 2-sided 95% CIs. No interactions will be considered.

**Supplementary Analyses:**

- PFS by BICR will be analyzed using the treatment policy strategy (Section 2.1.3). The analysis will use the same methodology and summary as the main analysis but will include observations that occur after the intercurrent events of starting new anticancer therapy. Reasons for censoring will be summarized according to the categories in Table 5, excluding number 2 - start of new anticancer therapy. All other intercurrent events will be addressed in the same approach as the primary estimand of PFS. Table 7 summarizes the details of the intercurrent events and censoring rules for this supplementary analysis.

**Table 7 PFS Outcome and Event Dates – Supplementary Analysis**

| Situation | Date of Progression/Censoring | Outcome |
|---|---|---|
| No adequate baseline assessment, including no disease at baseline | Date of randomization[a] | Censored[a] |
| PD or death ≤12 (or 16)[b] weeks after last adequate tumor assessment or ≤12 weeks after date of randomization | Date of PD or death | Event |
| PD or death >12 (or 16)[b] weeks after the last adequate tumor assessment[b] | Date of last adequate tumor assessment[c] documenting no PD prior to missed assessments | Censored |
| No PD | | |

a If the participant dies ≤12 weeks after date of randomization, the death is an event with date on death date.

b Durations are equal to 2 times the length of the tumor assessment interval, which is 12 weeks for the first 18 months after randomization, and 16 weeks thereafter.

c If there are no adequate post- baseline assessments prior to the PD or death, then the time without adequate assessment should be measured from the date of randomization; if the criteria were met the censoring will be on the date of randomization.

- PFS by BICR will be analyzed using the same methodology and summary as the main analysis, but will include observations/events that occur after >12 (or 16) weeks from last adequate post-baseline tumor assessment/start date. Reasons for censoring will be summarized according to the categories in Table 5, excluding number 3 – "Event >12 (or 16) weeks from last adequate post-baseline tumor assessment/start date". All other intercurrent events will be addressed in the same approach as the primary estimand of PFS.

090177e1a0219102\Approved\Approved On: 08-Mar-2024 15:37 (GMT)

**Discordance between BICR and Derived Investigator Assessment on PFS**

A summary of discordances of events (PD or death) between BIRC and derived investigator assessment will be provided as shown in Table 8:

**Table 8 Discordance Summary for Derived Investigator vs BICR Assessment on PFS**

| | | BICR | |
|---|---|---|---|
| **Derived Investigator** | | **Event** | **No Event** |
| | **Event** | a = a1 + a2 + a3 | b |
| | **No Event** | c | d |

a1: number of agreements on timing and occurrence of event;
a2: number of times agreement on event but INV declares event later than BICR;
a3: number of times agreement on event but INV declares event earlier than BICR;
N= a+b+c+d.
The timing agreement of event is defined as a window of ±7 days.

The following **measures of discordances** will be calculated:

- Total Event Discrepancy Rate: (b+c)/N.
- Early Discrepancy Rate (EDR): (a3+b)/(a+b).
- Late Discrepancy Rate (LDR): (a2+c)/(a2+a3+b+c).
- Overall Discrepancy Rate: (a2+a3+b+c)/N.

The EDR represents the positive predictive value of investigator assessment and quantifies the frequency with which the Investigator declares PFS event earlier than BICR within each treatment arm as a proportion of the total number of investigator-assessed PFS events.

The LDR quantifies the frequency with which the investigator declares PFS event later than BICR as a proportion of the total number of participants with discrepancies within the treatment arm.

Total event discrepancy rate represents the frequency with which the investigator and BICR disagreed on overall censoring/event status as a proportion of the number of participants within the treatment arm (N).

Overall discrepancy rate represents the frequency with which the investigator and BICR disagreed on overall censoring/event status or the timing of an event as a proportion of the number of participants within the arm (N).

Discordance metrics will be calculated for each treatment arm and, for each metric, the difference in discordance between Arm B and Arm C is used to evaluate potential bias. If the



090177e1a0219102\Approved\Approved On: 08-Mar-2024 15:37 (GMT)

discordance is similar across the treatment arms then it suggests the absence of evaluation bias favoring a particular arm. A negative differential discordance for EDR and/or a positive differential discordance for LDR may be indicative of investigator evaluation bias in favor of the experimental arm, ie, Arm B (Amit et al, 2011)[11].

### 6.1.2.2. ORR by BICR

#### 6.1.2.2.1. Main Analysis

ORR is defined as the proportion of participants who have achieved a BOR of confirmed CR or PR per RECIST v1.1 as assessed by BICR. The BOR is the best response obtained among all tumor assessment visits after date of randomization until documented PD by BICR, or start of subsequent anticancer therapy, or death. Confirmation of the response will be no sooner than 4 weeks after the initial documentation of CR or PR.

The following rules will be applied for the confirmation:

- CR = at least two determinations of CR at least 4 weeks apart and documented before progression, or start of new anticancer therapy.
- PR = at least two determinations of PR or better (and not qualifying for a CR) at least 4 weeks apart and before progression, or start of new anticancer therapy.
- SD (for participants with at least one measurable lesion at baseline)= at least one SD assessment (or better and not qualifying for CR or PR) ≥6 weeks after the study start date and before progression, or start of new anticancer therapy .
- Non-CR/Non-PD (for participants with only non-target disease at baseline) = at least one Non-CR/Non-PD assessment (or better and not qualifying for CR) ≥6 weeks after the study start date and before progression, or the start of new anticancer therapy.
- PD = progression ≤12 weeks after the study start date not qualifying for CR, PR or SD.
- Not Evaluable (NE) = all other cases, including ND (no evidence of disease at baseline).

Clinical deterioration or clinical progression noted on the completion eCRF will not be considered as documented disease progression.

The frequency (number and percentage) of participants with BOR of CR, PR, SD, PD, non-CR/non-PD (applicable only to participants with non-measurable disease at baseline), and NE will be tabulated.

Participants with a BOR of “Not Evaluable” based on confirmed responses will be summarized by reason for having unknown status. The following reasons will be used:

- No adequate baseline assessment.
- No evidence of disease at baseline.

090177e1a0219102\Approved\Approved On: 08-Mar-2024 15:37 (GMT)

- No post-baseline assessments due to early death (ie, death ≤12 weeks after the start date).
- No post-baseline assessments due to COVID-19 (ie, participants miss tumor assessment visits due to COVID-19 pandemic).
- No post-baseline assessments due to other reasons.
- All post-baseline assessments have overall response NE.
- New anticancer therapy started before first post-baseline assessment.
- SD of insufficient duration (<6 weeks after the start date without further evaluable tumor assessments).
- PD too late (>12 weeks after the start date, ie, tumor assessment of PD was >12 weeks after start of treatment and there was no tumor assessment in between).

Special and rare cases where BOR is NE due to both SD of insufficient duration ('too early') and late PD will be classified as "SD of insufficient duration".

The ORR will be calculated along with corresponding 2-sided Wilson Score[4] 95% CI.

The treatment effect of the Arm B compared to Arm C, as measured by stratified (by the randomization strata) odds ratio and its 95% CI and its 99.8% CI in terms of OR defined as the odds of OR with Arm B divided by the odds of OR with Arm C will be tested using Cochran-Mantel-Haenszel statistics stratified by the randomization strata at 1-sided 0.001 level of significance considering the first 110 randomized participants each in Arm B and in Arm C. This analysis will be performed eight months after randomization of the first 110 participants each in Arm B and in Arm C, or after the completion of enrollment of the Phase 3 portion of the study, whichever occurs later.
ORR may be evaluated descriptively only at the time of PFS analysis on all participants randomized in the Phase 3.

#### 6.1.2.2.2. Sensitivity/Supplementary Analyses

The following sensitivity and supplementary analyses will be performed to explore the robustness of the primary analysis results. These analyses are regarded as purely exploratory.

**Sensitivity Analyses:**

- An updated OR by BICR analysis at the time of PFS analysis may be performed including all randomized participants.

090177e1a0219102\Approved\Approved On: 08-Mar-2024 15:37 (GMT)

- OR by BICR will be analyzed based on Central *BRAF* V600E Positive analysis set. A stratified analysis will be performed to compare the OR by derived investigator assessment.

Concordance between BICR and derived investigator assessment on OR will be presented.

**Table 9. Definition of Agreement Used to Determine Total Agreement Rates in Response**

| | Derived Investigator | BICR | Variable Name |
|---|---|---|---|
| Agreement | No response | No response | a |
| | Response | Response | b |
| Disagreement | No response | Response | c |
| | Response | No response | d |

N = (a + b + c + d)

The total agreement rate measures the proportion of participants for whom there is a concordance between the BICR and derived investigator as to whether the participant was a responder or a non-responder with both the BICR and the derived investigator, among all participants who are evaluated by both BICR and investigator.

- Total Agreement Rate = [(a+b) / N] × 100%.

Concordance metrics will be calculated for each treatment arm and the difference in concordance between Arm B and Arm C is used to evaluate potential bias. If the concordance is similar across the treatment arms then it suggests the absence of evaluation bias favoring a particular arm.

### 6.1.3. Cohort 3

The primary objective is to compare the efficacy, as measured by the primary endpoint of ORR by BICR, of Arm D versus Arm E.

#### 6.1.3.1. ORR by BICR

##### 6.1.3.1.1. Main Analysis

ORR is defined as in Section 6.1.2.2.1 and will be calculated along with corresponding 2-sided Wilson Score[4] 95% CI.

The treatment effect of the Arm D compared to Arm E, as measured by stratified (by the randomization strata) odds ratio and its 95% CI in terms of OR defined as the odds of OR with Arm D divided by the odds of OR with Arm E will be tested using Cochran-Mantel-Haenszel statistics stratified by the randomization strata at 1-sided 0.025 level of significance. This analysis will be performed at least eight months after randomization of the last participant in Cohort 3.



090177e1a0219102\Approved\Approved On: 08-Mar-2024 15:37 (GMT)

090177e1a0219102\Approved\Approved On: 08-Mar-2024 15:37 (GMT)

#### 6.1.3.1.2. Sensitivity/Supplementary Analyses

The following sensitivity and supplementary analyses will be performed to explore the robustness of the primary analysis results. These analyses are regarded as purely exploratory.

**Sensitivity Analyses:**

- OR by BICR will be analyzed based on Central *BRAF* V600E Positive analysis set.
- A stratified analysis will be performed to compare the OR by derived investigator assessment.

Concordance between BICR and Derived Investigator Assessment on OR will be presented as described in Section 6.1.2.2.2.

## 6.2. Efficacy Secondary Endpoints

### 6.2.1. Safety Lead-in

#### 6.2.1.1. Objective Response Rate by Investigator

The OR is defined as described in Section 6.1.2.2.1, but the study start date is date of the first dose and tumor assessment is based on response reported by investigator on eCRF. ORR will be calculated by dividing the number of participants with BOR of CR or PR per RECIST v1.1 by the number of participants dosed in each cohort and number of lines of prior therapy.

The ORR will be calculated along with corresponding 2-sided Wilson Score[4] 95% CI.

#### 6.2.1.2. Duration of Response by Investigator

DOR will be calculated for participants with a confirmed response (CR or PR) as follows:

DOR (months) = (date of event or censoring – date of first CR or PR + 1)/30.4375

The same censoring rules specified for PFS in Section 6.1.2.1.1 apply to DOR. DOR will be estimated using the same Kaplan-Meier method as described for PFS. DOR rates at 3, 6, 9, 12 and 15 months will be estimated with corresponding 2-sided 95% CIs.

Frequency of participant with DOR ≥ 6 months, ≥ 9 months, ≥ 12 months will be reported.

In addition, a plot of time to and duration of response for participants with a confirmed response (swimmer plot) will be created.

DOR will be calculated and presented by cohort and number of lines of prior therapies.

### 6.2.1.3. Time to Response by Investigator

TTR will be calculated for participants with a confirmed response (CR or PR) in weeks as follows:

TTR (in weeks) = [date of first CR or PR – date of first dose +1]/7

TTR will be summarized using simple descriptive statistics (mean, standard deviation, median, minimum, maximum, first quartile, third quartile).

TTR will be calculated and presented by cohort and number of lines of prior therapies.

### 6.2.1.4. Progression-free Survival by Investigator

For the SLI, PFS will be calculated in months as follows:

PFS (months) = (date of event or censoring – date of first dose +1)/30.4375

The same censoring rules specified for PFS in the Phase 3 portion in Section 6.1.2.1.1 apply to PFS in the SLI, with the date of randomization replaced by the date of first dose in the SLI and tumor response as assessed by the investigator. Kaplan-Meier estimates will be presented in a table and a plot by cohort and number of lines for prior therapies together with a summary of associated statistics including the median PFS time with 2-sided 95% CI. The PFS at 3, 6, 9, 12, 15, 18 and 21 months will be estimated with corresponding 2-sided 95% CIs.

Frequency (number and percentage) of participants with each event type (PD or death) and censoring reasons will be presented by cohort and number of lines for prior therapies along with the overall event and censor rates. Reasons for censoring will be summarized according to the categories in Table 5.

### 6.2.1.5. Overall Survival

OS will be calculated in months as follows:

OS (months) = (date of death or censoring – date of first dose +1)/30.4375

Kaplan-Meier estimates will be presented in a table and a plot by cohort and by line of prior therapies together with a summary of associated statistics including the median OS time with 2-sided 95% CI. The OS at 6, 12, 18 and 24 months will be estimated with corresponding 2-sided 95% CIs.

Frequency (number and percentage) of participants with death events and censoring reasons will be presented by cohort and by line of prior therapies along with the overall event and censor rates.

090177e1a0219102\Approved\Approved On: 08-Mar-2024 15:37 (GMT)

### 6.2.2. Phase 3

#### 6.2.2.1. Overall Survival

##### 6.2.2.1.1. Main Analysis

The key secondary efficacy analysis will compare OS time between Arm B and Arm C using a 1-sided stratified log-rank test. The nominal significance level (0.001 or 0.023) will depend on significance of ORR/PFS analyses. The actual alpha spent at the interim and final analysis will be updated based on the actual number of events and the information fraction (see details in Section 7.2). The analysis will be performed based on the FAS. Strata will be based on those specified in the IRT system as described in Section 3.4.1.

OS will be calculated in months as follows:

OS (months) = (date of death or censoring – date of randomization +1)/30.4375

The treatment effect will be estimated using a Cox's proportional hazards model stratified by randomization strata to calculate the HR for OS of Arm B versus Arm C.

In order to account for the group sequential design on this endpoint, the repeated CI method[5] will be used to construct the 2-sided repeated CI for the HR at the interim and the final analyses of OS, in addition to the unadjusted 2-sided 95% CI.

OS time will be estimated using the same Kaplan-Meier method as described for PFS in Section 6.1.2.1.1. The OS at 6, 12, 18, 24, 30 and 36 months will be estimated with corresponding 2-sided 95% CIs.

Frequency (number and percentage) of participants with death events and censoring reasons will be presented by treatment arm along with the overall event and censor rates. The event and censoring reasons are as follows:

- Death:
  - Death due to COVID-19;
- Ongoing and no death;
- Withdrawal of consent;
- Lost to follow-up.

The OS time or censoring time and the reasons for censoring will also be presented in a patient listing.

Schoenfeld residuals for the stratified Cox proportional model may be plotted to investigate graphically violations from the proportional hazards (PH) assumption (see Section 5.2.18 for details).



090177e1a0219102\Approved\Approved On: 08-Mar-2024 15:37 (GMT)

**Time of Follow-Up for OS in Phase 3**

A Kaplan-Meier plot for OS follow-up duration will also be created to assess the follow-up time in each treatment arm using the reversed Kaplan-Meier method as shown in Table 10. The Kaplan-Meier estimate of the median OS follow-up time with its corresponding 2-sided 95% CI will be calculated and presented in the plot.

**Table 10 Reverse Censoring Rules for OS Follow-up Duration**

| | Date of event / censoring | Censoring |
|---|---|---|
| Participant didn't die | Time from randomization to last date known to be alive | No |
| Participant died | Time from randomization to date of death | Yes |

### 6.2.2.1.2. Sensitivity/Supplementary Analysis

The following sensitivity and supplementary analyses will be performed to explore the robustness of the key secondary endpoint analysis results in the Phase 3 portion of the study. These analyses are regarded as purely exploratory.

**Sensitivity Analyses:**

- An unstratified analysis will be performed to compare the OS time.
- OS will be analyzed based on Central *BRAF* V600E Positive analysis set.
- OS will also be analyzed based on RMST differences (see Section 5.2.18 for details).
- The same multivariable Cox regression analysis that is performed on PFS as described in Section 6.1.2.1.2 will also be conducted on OS to assess and adjust the treatment effect for relevant baseline factors of potential prognostic impact.

**Supplementary Analyses:**

Since majority of the patients enrolled in this study will likely receive additional anticancer systemic therapy upon PD by BICR, it is unclear if/how this additional therapy may impact the treatment effect estimate on OS. Based on type/frequency/distribution of follow-up therapy, analyses to correct for follow-up systemic therapy on treatment effect estimate for OS (eg 2-stage or other appropriate method) may be implemented. A swimmer plot may be produced to show OS time course from randomization, for each patient, denoting duration of randomized treatment and duration of subsequent anti-cancer systemic therapies.

### 6.2.2.2. Duration of Response

DOR will be calculated as described in Section 6.2.1.2 at the time of ORR analysis and at the time of PFS analysis.

Both DOR by BICR and DOR by derived investigator will be calculated and presented by arm.



090177e1a0219102\Approved\Approved On: 08-Mar-2024 15:37 (GMT)

#### 6.2.2.3. Time to Response

TTR will be calculated at the time of ORR analysis for participants with a confirmed response (CR or PR) in weeks as follows:

TTR (in weeks) = [date of first CR or PR – date of randomization +1]/7

TTR will be summarized using simple descriptive statistics (mean, standard deviation, median, minimum, maximum, first quartile, third quartile).

Both TTR by BICR and TTR by derived investigator will be calculated and presented by arm.

#### 6.2.2.4. Progression-free Survival 2

PFS2 will be calculated in months for Phase 3 portion of the study as follows at the time of PFS analysis and final OS analysis:

PFS2 (months) = [minimum of (date of death, date of second objective PD, date of discontinuation of next-line treatment after first objective PD by investigator assessment) or censoring – date of randomization +1]/30.4375

As defined in Section 3.2.2.6, PD2 is PD after the start of subsequent anticancer therapy based on investigator assessment. A participant will be considered to have an event if

1. the participant had PD by investigator on or prior to start of next-line anticancer treatment, AND started next-line anticancer treatment, AND (had objective PD after start of next-line anticancer treatment OR discontinued next-line anticancer treatment).

   OR

2. the participant died.

The censoring and event date options to be considered for PFS2 analysis are presented in Table 11.

PFS2 time will be estimated using the same Kaplan-Meier method as described for PFS.

**Table 11 Outcome, Event Dates, Censoring Reasons and Hierarchy for PFS2 Analyses**

| Scenario | Date of event/ censoring | Event/<br>Censoring reason/<br>Censoring hierarchy |
|---|---|---|
| (No PD[a]) and (no death) | Date of last adequate tumor assessment[b] documenting no PD | Censored/<br>No PD/<br>1 |



090177e1a0219102\Approved\Approved On: 08-Mar-2024 15:37 (GMT)

## Table 11 Outcome, Event Dates, Censoring Reasons and Hierarchy for PFS2 Analyses

| Scenario | Date of event/ censoring | Event/ Censoring reason/ Censoring hierarchy |
|---|---|---|
| (No PD[a]) and death | Date of death | Event (Death) |
| (PD[a] and no NTX[c]) and death | Date of death | Event (Death) |
| (PD[a] date > NTX[c] start date) and (no death) | Start date of NTX[c] | Censored/<br>Start of new anticancer treatment before PD/<br>2 |
| (PD[a] date > NTX[c] start date) and death | Date of death | Event (Death) |
| (PD[a] date ≤ NTX[c] start date) and (PD2[d] date > NTX[c] start date) | | |
| • If PD2 date ≤ NTX[c] end date | Date of PD2 | Event (PD after start of next line anticancer treatment) |
| • If PD2 date > NTX[c] end date | End date of NTX[c] | Event (Discontinuation of next line anticancer treatment) |
| (PD[a] date ≤ NTX[c] start date) and (no PD2[d]) and (no death) | | |
| • If NTX[c] end date is non-missing | End date of NTX[c] | Event (Discontinuation of next line anticancer treatment) |
| • Else if [withdrawal of consent date ≥ date of randomization OR End of study (EOS) = Participant refused further follow-up] | (Withdrawal of consent date) or (EOS visit date where participant refusal of further follow-up is recorded) | Censored/<br>Withdrawal of consent/<br>3 |
| • Else if [lost to follow-up in any disposition page] | Last contact date | Censored/<br>Lost to follow-up/<br>4 |
| • Else if no prior conditions are met | Last contact date | Censored/<br>Ongoing without PFS2 event/<br>5 |
| (PD[a] and no NTX[c]) and (no death) | Last contact date | Censored/<br>Ongoing without PFS2 event/<br>5 |
| (PD[a] date ≤ NTX[c] start date) and (no PD2[d]) and death | | |
| • If NTX[c] end date is non-missing | End date of NTX[c] | Event (Discontinuation of next line anticancer treatment) |
| • Else if the prior condition is not met | Date of death | Event (Death) |

a. PD is the first PD by investigator, without considering any censoring rules



090177e1a0219102\Approved\Approved On: 08-Mar-2024 15:37 (GMT)

**Table 11 Outcome, Event Dates, Censoring Reasons and Hierarchy for PFS2 Analyses**

| Scenario | Date of event/ censoring | Event/ Censoring reason/ Censoring hierarchy |
|---|---|---|

b. If there are no adequate post-baseline assessments, then the censoring date is the date of randomization. If a participant has initiated next-line anticancer treatment, the last adequate post-baseline assessment on or prior to start date of next-line anticancer treatment will be considered.

c. NTX is the next-line anticancer treatment.

d. PD2 is the first PD by investigator assessment after initiation of next-line anticancer treatment, without considering any censoring rules.

#### 6.2.2.5. Efficacy Endpoints of Arm A Versus Arm C and Versus Arm B Comparison

For the Phase 3 portion of the study, the following efficacy endpoints will also be analyzed between Arm A and Arm C/Arm B using the same analysis methods as those used for the corresponding endpoint of Arm B versus Arm C at the time of PFS analysis. As these comparisons are not part of the multiple testing strategy as outlined in Section 5.1.2.2, no formal statistical testing will be performed on these endpoints between Arm A and Arm C/Arm B:

- PFS by BICR and by derived investigator assessment.
- OS.
- ORR by BICR and by derived investigator assessment.
- DOR by BICR and by derived investigator assessment.
- TTR by BICR and by derived investigator assessment.
- PFS2.

### 6.2.3. Cohort 3

#### 6.2.3.1. Progression-free Survival

##### 6.2.3.1.1. Main Analysis

The key secondary efficacy analysis will compare PFS time by BICR between Arm D and Arm E using a 1-sided stratified log-rank test at the significance level of 0.025. The analysis will be performed based on the FAS when approximately 82 PFS events by BICR will be observed only if the results for ORR by BICR analysis are statistically significant. Strata will be based on those specified in the IRT system as described in Section 3.4.1.

PFS will be calculated as described in Section 6.1.2.1.1.

090177e1a0219102\Approved\Approved On: 08-Mar-2024 15:37 (GMT)

The treatment effect will be estimated using a Cox's proportional hazards model stratified by randomization strata to calculate the HR for PFS of Arm D vs. Arm E, along with the corresponding 2-sided 95% CI.

Kaplan Meier estimates will be presented as described in Section 6.1.2.1.1, including the time to follow-up for PFS by BICR.

#### 6.2.3.1.2. Sensitivity/Supplementary Analyses

Sensitivity and supplementary analyses will be conducted as described in Section 6.1.2.1.2.

### 6.2.3.2. Duration of Response

DOR will be calculated as described in Section 6.2.1.2 at the time of ORR analysis and at the time of PFS analysis.

Both DOR by BICR and DOR by derived investigator assessment will be calculated and presented by arm.

### 6.2.3.3. Time to Response

TTR will be calculated and summarized at the time of ORR analysis as described in Section 6.2.2.3.

Both TTR by BICR and DOR by derived investigator assessment will be calculated and presented by arm.

### 6.2.3.4. Overall Survival

OS for Cohort 3 will be calculated at the time of ORR and PFS analysis as described in Section 6.2.2.1.1, but it will be reported descriptively only.

## 6.3. Other Secondary Endpoints

### 6.3.1. Patient-Reported Outcomes (PRO)

The following PRO analyses will be performed on the full analysis set at the time of PFS analysis to support the CSR development for Phase 3 portion and for Cohort 3 of the study. All other PRO analyses that are not included in this SAP will be described in detail in a separate PRO analysis plan.

#### 6.3.1.1. Scoring

The EORTC QLQ-C30 and EQ-5D-5L will be scored according to their respective validation papers and User's Guides (Fayers et al.)[12]. The current version at the time of analysis of UK weights will be used to construct the index values of the EQ-5D-5L.

PGIS and PGIC questionnaire items will be analyzed separately. Items within each questionnaire will not be combined or scored.

090177e1a0219102\Approved\Approved On: 08-Mar-2024 15:37 (GMT)

#### 6.3.1.2. Instrument Completion Rates

For each treatment arm and at each time point, the number and percentage of participants who complete each of the EORTC QLQ-C30, EQ-5D-5L, PGIS, and PGIC will be summarized, as will the reasons for non-completion of these measures.

#### 6.3.1.3. Descriptive Summary

For the Global Health Status/QoL scale, the subscales of the EORTC QLQ-C30 and EQ-5D-5L VAS, summary tables including the mean, median, SD, range and 95% CIs, and line charts depicting the means along with standard error bars over time will be provided for each scale value and change from baseline at each time point by treatment arm.

#### 6.3.1.4. Health Status

For the EQ-5D-5L, the number and percentage of participants reported having "none", "slight", "moderate", "severe", or "extreme/unable" problems at each time point will be summarized.

### 6.3.2. Pharmacokinetic Analysis

#### 6.3.2.1. Safety Lead-in

Plasma concentrations and PK parameter estimates will be determined for encorafenib, its metabolite (LHY746), irinotecan and its metabolite SN-38, and oxaliplatin (total platinum in plasma and platinum in plasma ultrafiltrate). PK summaries will be presented by cohort by visit for participants in the PK analysis set. Cycle 1 Day 15 is defined as steady state of encorafenib.

##### 6.3.2.1.1. Plasma Concentrations of Encorafenib and LHY746, Irinotecan and SN-38, and Oxaliplatin

In the SLI EC + FOLFIRI cohort, plasma concentrations of encorafenib and its metabolite (LHY746), and irinotecan and its metabolite (SN-38) will be quantified at the time points indicated in Table 12. In the SLI EC + mFOLFOX6 cohort, plasma concentrations of encorafenib and its metabolite (LHY746), and oxaliplatin (total platinum in plasma and platinum in ultrafiltrate) will be quantified at the time points indicated in Table 13.

090177e1a0219102\Approved\Approved On: 08-Mar-2024 15:37 (GMT)

**Table 12 PK Sampling Schedule for Cohort 1 (EC + FOLFIRI) in the Safety Lead-in**

| Visit | Sampling Time | Irinotecan PK Sample (3 mL Blood) | Encorafenib PK Sample (2 mL Blood) |
|---|---|---|---|
| **Cycle 1 Day 1** | 0 hr (predose[b]) | X | |
| | 0.75 hr | X | |
| | 1.5 hr | X[c] | |
| | 2.5 hr | X | |
| | 3.5 hr | X | |
| | 5.5 hr | X | |
| | 7.5 hr | X | |
| **Cycle 1 Day 3** | 0 hr (predose[d]) | X | |
| **Cycle 1 Day 15[a]** | 0 hr (predose[d]) | X | X |
| | 0.75 hr | X | X |
| | 1.5 hr | X[c] | X |
| | 2.5 hr | X | X |
| | 3.5 hr | X | X |
| | 5.5 hr | X | X |
| | 7.5 hr | X | X |
| **Cycle 1 Day 17** | 0 hr (predose[d]) | X | X |
| **Day 1 of Cycle 2 – 6** | 0 hr (predose[d]) | X | X |

a. Encorafenib will be administered within 5 minutes before the start of irinotecan infusion
b. Predose is within 30 minutes prior to irinotecan infusion
c. Immediately before irinotecan infusion ends
d. Predose is within 30 minutes prior to encorafenib

Note: the time points are related to the start time of administration of respective drugs. Collection of samples up to and including 8 hours after dose administration should be obtained within ~ 10% of the nominal time relative to dosing (eg, within 6 minutes of a 1-hour sample)

090177e1a0219102\Approved\Approved On: 08-Mar-2024 15:37 (GMT)

**Table 13 PK Sampling Schedule for Cohort 2 (EC + mFOLFOX6) in the Safety Lead-in**

| Visit | Sampling Time | Oxaliplatin PK Sample (5 mL Blood) | Encorafenib PK Sample (2 mL Blood) |
|---|---|---|---|
| **Cycle 1 Day 1[a]** | 0 hr (predose[b]) | X | X |
| | 1 hr | X | X |
| | 2 hr | X[c] | X |
| | 3 hr | X | X |
| | 4 hr | X | X |
| | 6 hr | X | X |
| | 8 hr | X | X |
| **Cycle 1 Day 3** | 0 hr (predose[b]) | X | X |
| **Cycle 1 Day 15[a]** | 0 hr (predose[b]) | X | X |
| | 1 hr | X | X |
| | 2 hr | X[c] | X |
| | 3 hr | X | X |
| | 4 hr | X | X |
| | 6 hr | X | X |
| | 8 hr | X | X |
| **Cycle 1 Day 17** | 0 hr (predose[b]) | X | X |
| **Day 1 of Cycle 2 – 6** | 0 hr (predose[b]) | X | X |

a. Encorafenib will be administered within 5 minutes before the start of oxaliplatin infusion
b. Predose is within 30 minutes prior to encorafenib
c. Immediately before oxaliplatin infusion ends

Note: the time points are related to the start time of administration of respective drugs. Collection of samples up to and including 8 hours after dose administration should be obtained within ~ 10% of the nominal time relative to dosing (eg, within 6 minutes of a 1 hour sample)

All plasma concentration values for each participant in the safety set will be included in the bioanalytical plasma concentration listings, and participants will be identified as being in the PK set, as applicable. Individual concentration records will be flagged for the affected visit if any of the following occur:

a. Participant vomits within 4 hours following encorafenib dosing on the day of PK sampling (Cycle 1 Day 1 and Cycle 1 Day 15).

b. Encorafenib plasma levels are not considered to be at steady-state (ie, encorafenib dosing was not performed for at least 3 consecutive days prior to Cycle 1 Day 15).

c. Participant receives a higher or lower dose of encorafenib compared to planned treatment on the day of PK sampling (Cycle 1 Day 1 and Cycle 1 Day 15).

d. Participant receives a higher or lower dose of irinotecan compared to planned treatment on the day of PK sampling (Cycle 1 Day 1 and Cycle 1 Day 15).

e. Participant receives a higher or lower dose of oxaliplatin compared to planned treatment on the day of PK sampling (Cycle 1 Day 1 and Cycle 1 Day 15).



090177e1a0219102\Approved\Approved On: 08-Mar-2024 15:37 (GMT)

f. PK sampling time deviation >10%

The plasma concentrations of encorafenib and LHY746, and irinotecan and SN-38, and oxaliplatin will be summarized for all nominal time points, including predose (trough) concentrations for Day 1 of Cycle 2 through Cycle 6, using the following descriptive statistics: N (number of participants in the population), n (number of participants with non-missing values), arithmetic mean, standard deviation, coefficient of variation (CV), geometric mean, geometric standard deviation, geometric CV, minimum, median and maximum. Exclusion of an individual concentration-time data point from the calculation of summary statistics will follow the rules below:

- For encorafenib and LHY746 on Cycle 1 Day 1, if any of the above flags [a, c, and f] apply; for encorafenib and LHY746 on Cycle 1 Day 15, if any of the above flags [a, b, c, and f] apply.
- For irinotecan and SN-38 on Cycle 1 Day 1, if any of the above flags [d and f] apply; for irinotecan and SN-38 on Cycle 1 Day 15, if any of the above flags [a, b, d and f] apply
- For oxaliplatin on Cycle 1 Day 1, if any of above flags [a, e and f] apply; for oxaliplatin on Cycle 1 Day 15, if any of above flags [a, b, e, and f] apply.

Concentrations reported as below the limit of quantification (BLQ) will be set to zero for the calculation of arithmetic mean, standard deviation, CV, minimum, median and maximum. For the calculation of geometric mean and geometric CV, BLQ values will be set as missing. All summary statistics below the limit of quantification will be presented as "< X.XX" where X.XX represents LLOQ.

The median plasma concentration-time profiles on Cycle 1 Day 1 and Cycle 1 Day 15 will be presented graphically for each analyte by cohort, as appropriate, using both linear and semi-logarithmic scales and nominal time. In the median plasma concentration-time profiles of irinotecan and SN-38, or oxaliplatin, the time of the predose concentrations on Cycle 1 Day 3 and Cycle 1 Day 17 will be imputed as nominal time 48 hrs post-start of IV infusion on Cycle 1 Day 1, and Cycle 1 Day 15, respectively. Only patients who have at least 1 matched pair of estimated pharmacokinetic parameters available on both Cycle 1 Day 1 and Cycle 1 Day 15 will be included in the median concentration profiles except for encorafenib and LHY746 in the cohort 1 because the first dose of encorafenib will not be administered till Cycle 1 Day 3 in the cohort 1 (EC + FOLFIRI). The median trough plasma concentration-visit plots will be presented for each analyte by cohort, as appropriate, using both linear and semi-logarithmic scales.

#### 6.3.2.1.2. Plasma Pharmacokinetic Parameters for Encorafenib and LHY746, Irinotecan and SN-38, and Oxaliplatin

Pharmacokinetic parameters defined in Table 14 for participants in the PK set will be determined, as data permit and when appropriate, with noncompartmental analysis (NCA) using an internally validated software system, open NCA. Actual blood collection times with respect to the actual dose time of corresponding study drug will be used for PK parameter



090177e1a0219102\Approved\Approved On: 08-Mar-2024 15:37 (GMT)

calculation. If actual times are not recorded/available, nominal times will be used. For irinotecan and SN-38, and oxaliplatin, the time of predose samples on Cycle 1 Day 3 and Cycle 1 Day 17 will be imputed as the actual time intervals from start of infusion on Cycle 1 Day 1 and Cycle 1 Day 15, respectively, and included for calculation of PK parameters. All BLQ values before the observed maximum plasma concentration ($C_{max}$) will be set to 0; all BLQ values after $C_{max}$ will be considered as missing.

The AUC parameters will be calculated according to the linear-up log-down trapezoidal rule. Additional PK parameters may be calculated at the discretion of the pharmacokineticist.

All PK parameter values will be presented in data listings by analyte and visit and cohort. Pharmacokinetic parameters (and if appropriate, dose-normalized exposure parameters) (Table 14) will be summarized in tables by analyte and visit and cohort using the following descriptive statistics: N, n, arithmetic mean, standard deviation, CV, geometric mean, geometric CV, minimum, median and maximum. For $T_{max}$ values, median, minimum and maximum will be presented.

Exclusion of pharmacokinetic parameters from the calculation of summary statistics will follow the rules below:

- For encorafenib and LHY746 on Cycle 1 Day 1 (only for the cohort 2, EC + oxaliplatin), if any of the above flags [a and c] apply; for encorafenib and LHY746 Cycle 1 Day 15, if any of the above flags [a, b, and c] apply.

- For irinotecan and SN-38 on Cycle 1 Day 1, if any of the above flags [d] apply; for irinotecan and SN-38 on Cycle 1 Day 15, if any of the above flags [a, b and d] apply.

- For oxaliplatin on Cycle 1 Day 1, if any of above flags [a and e] apply; for oxaliplatin on Cycle 1 Day 15, if any of above flags [a, b and e] apply.

When dose-normalized pharmacokinetic parameters are calculated, exclusion of dose-normalized pharmacokinetic parameters from the calculation of summary statistics will follow the rules below:

- For encorafenib and LHY746 on Cycle 1 Day 1 (only for the cohort 2, EC + oxaliplatin), if any of the above flags [a] apply; for encorafenib and LHY746 Cycle 1 Day 15, if any of the above flags [a and b] apply.

- For irinotecan and SN-38 on Cycle 1 Day 15, if any of the above flags [a and b] apply.

- For oxaliplatin on Cycle 1 Day 1, if any of above flags [a] apply; for oxaliplatin on Cycle 1 Day 15, if any of above flags [a and b] apply.

In addition, some samples may be excluded from the summary analysis based on open NCA group assessment.



090177e1a0219102\Approved\Approved On: 08-Mar-2024 15:37 (GMT)

**Table 14 Pharmacokinetic Parameters**

| Analytes | PK Parameters |
|---|---|
| Oral<br>Encorafenib and the metabolite LHY746 | $C_{max}$, $C_{min}$, $C_{trough}$, $AUC_{last}$, $AUC_{tau}$, $R_{ac,Cmax}$, $R_{ac,AUC}$, $MR_{Cmax}$, $MR_{AUC}$, $T_{max}$, $AUC_x$, CL/F, $V_z$/F<br>If appropriate, dose normalized exposure parameters to the planned dose: $C_{max}$, $C_{min}$, $C_{trough}$, $AUC_{last}$, $AUC_{tau}$, and $AUC_x$ |
| IV infusion<br>Irinotecan and the metabolite SN-38<br>Oxalipatin (platinum in plasma and platinum in plasma ultrafiltrate) | $C_{max}$, $C_{min}$, $C_{trough}$, $AUC_{last}$, $AUC_x$, $R_{ac,Cmax}$, $R_{ac,AUC}$,<br>$MR_{Cmax}$, $MR_{AUC}$, $T_{max}$, $AUC_{inf}$, $k_{el}$, $t_{1/2}$, CL, $V_z$<br>If appropriate, dose normalized exposure parameters to the planned dose: $C_{max}$, $C_{min}$, $C_{trough}$, $AUC_{last}$, $AUC_x$, and $AUC_{inf}$ |

Note: Pharmacokinetic parameters will be determined, as data permit and when appropriate, with noncompartmental analysis (NCA) using an internally validated software system, openNCA.

#### 6.3.2.1.3. Drug-drug Interaction

Effect of steady-state encorafenib on irinotecan and SN-38 or oxaliplatin PK will be evaluated based on overall assessment of the geometric mean ratios (Cycle 1 Day 15/Cycle 1 Day 1) and associated 90% CI for PK parameter estimates of irinotecan and SN-38 or oxaliplatin, respectively, (ie, $C_{max}$ and AUC, CL). Only participants who have a matched pair of estimated pharmacokinetic parameters available Cycle 1 Day 1 and Cycle 1 Day 15 will be included in this analysis. Exclusion is described as in Section 6.3.2.1.2.

The effect of a single dose of encorafenib on oxaliplatin PK, as appropriate, may be evaluated by comparing exposures of oxaliplatin (ie, $C_{max}$, AUC) on Cycle 1 Day 1 in EC + mFOLFOX6 cohort to historical data. The effect of irinotecan or oxaliplatin on encorafenib and LHY746 may be evaluated by comparison of encorafenib and LHY746 PK results to historical results. Comparisons to historical PK results will be qualitative, exploratory, and only done as data permit.

The final PK analysis may vary at the discretion of the pharmacokineticist.

#### 6.3.2.2. Phase 3

The central laboratory, analytical laboratories, and Pfizer clinical assay group colleagues will be unblinded. If the need arises for early analysis of the PK data (before database lock and release of the randomization codes for the study), a PK unblinding plan will be developed. A PK analyst, who is not associated with the study team, will conduct the analysis to avoid unblinding of the study team. The PK analysis set will be used.



090177e1a0219102\Approved\Approved On: 08-Mar-2024 15:37 (GMT)

Trough plasma concentrations of encorafenib and LHY746 on Day 1 of Cycle 1 through Cycle 6 will be determined in Arm A and Arm B. Individual concentration records will be flagged as in Section 6.3.2.1.1. All plasma concentration values for each participant in the safety set will be included in the bioanalytical plasma concentration listings, and participants will be identified as being in the PK set, as applicable. The values will be summarized by analyte and visit and arms descriptively (n, mean, standard deviation, CV, median, minimum, maximum, geometric mean, and its associated CV). The median trough plasma concentration-visit plots will be presented for each analyte by arm, as appropriate, using both linear and semi-logarithmic scales.

#### 6.3.2.2.1. Pharmacokinetics of Encorafenib and the Metabolite LHY746 in Mainland Chinese Population

Plasma concentration and pharmacokinetic parameters of encorafenib and LHY746 for Day 1 of Cycle 1 and steady state (Day 15 of Cycle 1, or Day 1 or Day 15 of Cycle 2 if a dose interruption occurs prior to Day 15 of Cycle 1) will be summarized for participants randomized to Arm A or Arm B in China who underwent intensive PK.

Individual concentration records will be flagged as in Section 6.3.2.1.1. All plasma concentration values for each participant in the safety set will be included in the bioanalytical plasma concentration listings, and participants will be identified as being in the PK set, as applicable. Plasma concentration will be summarized descriptively (n, mean, standard deviation, CV, median, minimum, maximum, geometric mean, and its associated CV) by analyte and nominal time for Day 1 of Cycle 1 and steady state. The median plasma concentration-time plots will be presented for each analyte, as appropriate, using both linear and semi-logarithmic scales, for Day 1 of Cycle 1 and steady-state.

Plasma pharmacokinetic parameters listed below, for Cycle 1 Day 1 and steady state, will be estimated using noncompartmental analysis if data permits or if considered appropriate:

- $C_{trough}$: trough plasma concentration.
- $C_{max}$: maximum plasma concentrations.
- $T_{max}$: time to maximum plasma concentration.
- $AUC_t$: area under the plasma concentration versus time profile from time 0 to time t.
- $AUC_{tau}$: area under the plasma concentration versus time profile within a dose interval.
- $AUC_{inf}$: area under the plasma concentration versus time curve to infinity.
- $t_{1/2}$: terminal elimination half-life.
- CL/F: oral plasma clearance.



090177e1a0219102\Approved\Approved On: 08-Mar-2024 15:37 (GMT)

- $V_z/F$: apparent volume of distribution.
- $R_{ac}$: accumulation ratio.
- MR: metabolite ratio.

All PK parameter values will be presented in data listings by analyte for Cycle 1 Day 1 and steady state. The PK parameters will be summarized by analyte, descriptively (n, mean, standard deviation, CV, median, minimum, maximum, geometric mean, and its associated CV).

Participants who vomit within 4 hours following encorafenib dosing on the day of PK sampling (Cycle 1 Day 1 and steady state) or who receive a higher or lower dose of encorafenib compared to planned treatment on the day of PK sampling (Cycle 1 Day 1 and steady state) will be excluded from the summary of plasma concentration and pharmacokinetic parameters and median plasma concentration-time plots. When appropriate, dose normalized concentrations and PK parameters may be used for the analysis. The final PK analysis may vary at the discretion of the pharmacokineticist.

#### 6.3.2.3. Cohort 3

Trough plasma concentrations of encorafenib and LHY746 on Day 1 of Cycle 1 through Cycle 6 will be determined in Arm D. Individual concentration records will be flagged as in Section 6.3.2.1.1. All plasma concentration values for each participant in the safety set will be included in the bioanalytical plasma concentration listings, and participants will be identified as being in the PK set, as applicable. The values will be summarized by analyte and visit descriptively (n, mean, standard deviation, CV, median, minimum, maximum, geometric mean, and its associated CV). The median trough plasma concentration-visit plots will be presented for each analyte by arm, as appropriate, using both linear and semi-logarithmic scales.

### 6.3.3. Biomarker Analyses

The secondary biomarker endpoint data will be analyzed for the Phase 3 and Cohort 3 portion of the study based on the biomarker analysis sets as defined in Section 4. Data will be presented by treatment arm. Summaries of baseline levels, changes from baseline (where appropriate), expression or genetic alterations/status will be reported. For continuous variables, summary statistics may include the mean, ratio to baseline, standard deviation, median, first and third quartiles, %CV, and minimum/maximum levels; for categorical variables, summary may include number and percentage, odds ratio, and frequency statistics, as appropriate.

No duplicate results for biomarker or pharmacodynamic markers are expected. However, if more than one record is received for a particular time point, the duplicate records might be averaged or most recent result will be used for continuous data. For non-continuous data, the results will be reviewed by the study team and a representative sample will be selected. When participants provide both archival and de novo tumor tissue samples at screening, data from the de novo sample will be used in the summary analysis.



090177e1a0219102\Approved\Approved On: 08-Mar-2024 15:37 (GMT)

Data from biomarker assays may be analyzed using graphical methods and descriptive statistics such as Kaplan-Meier estimates of efficacy parameters (eg, PFS, OS) with biomarkers as a covariate, or Box plots. The statistical approach may examine correlations of biomarker results with pharmacokinetic parameters and measures of anti-tumor efficacy.

Regarding exploratory biomarkers, the data analysis will be conducted with the goal of identifying predictive and/or pharmacodynamic (ie, baseline and change from baseline values) biomarkers associated with clinical outcome, encompassing both safety and efficacy. These analyses may not be reported in the Clinical Study Report. Candidate biomarkers will be validated in subsequent trials.

#### 6.3.3.1. Tumor Tissue Analyses

##### 6.3.3.1.1. MSI Status Analyses

Retrospective analyses for tumor-based MSI status performed at a central laboratory will be based on the tumor tissue analyses set.

MSI status in tumor tissue is measured at baseline (pre-screening/screening). Participants are expected to be MSI-H, MSS/MSI-L (presented combined in the tables) or MSI-unknown.

A descriptive summary table at baseline for MSI status will be provided. When appropriate, a descriptive summary table comparing results from local MSI/dMMR testing and central MSI testing within the tumor tissue analyses set will be provided. The number and percentage of participants in each category will be tabulated. All data will be listed.

##### 6.3.3.1.2. *BRAF* V600E Analyses

Analyses for tumor-based *BRAF* V600E will be based on the full analysis set.

Diagnostic concordance assessment of *BRAF* V600E status between local tumor tissue/plasma results (as reported in the eCRF) and the reference, central PCR-based QIAGEN *BRAF* V600E FDA-approved test will be presented. The reference, the central test is the tumor tissue PCR testing performed at screening as part of the enrollment criteria, and all participants are therefore considered *BRAF* V600E positive (ie, detected). Overall concordance will be summarized by treatment arm and in total.

#### 6.3.3.2. ctDNA Analyses

Retrospective analyses of ctDNA alterations will be based on either the cfDNA analysis set or the paired cfDNA analysis set. Note that mainland China uses a different vendor than the rest of the world for this biomarker; hence data from mainland China will be reported separately.

ctDNA alterations will be measured at defined timepoints including baseline, on-treatment and End of Treatment. Biomarkers will be summarized at defined timepoints when appropriate. Participants may have detectable ctDNA before, during and/or after treatment. Participants with detectable ctDNA are expected to have either none or one or more measurable tumor biomarkers including *BRAF* V600 alterations.



090177e1a0219102\Approved\Approved On: 08-Mar-2024 15:37 (GMT)

The following definitions will be used for subgroups based on whether ctDNA was detected or not at C1D1 and C1D15:

- • "ND/ND" = ND ctDNA at C1D1 and C2D15
- • "D/ND" = D at C1D1 and ND at C2D15
- • "ND/D" = ND at C1D1 and D at C2D15
- • "D/D" = D at C1D1 and C2D15

PFS and OS analyses will be stratified by ctDNA status at baseline (detected or not-detected) and/or changes in ctDNA status (ND/ND, D/ND, ND/D, D/D [C1D1/C2D15]) and will use the same Kaplan-Meier method as described for PFS. Kaplan-Meier curves with appropriate summary statistics including median time and associated 2-sided 95% CIs will be calculated.

A summary table of measurable *BRAF* V600 VAF (mean, maximum, minimum and standard deviation) at available timepoints will be provided. A summary table will be provided for subgroups defined by BRAF V600 VAF at baseline as measurable or not-measurable, or as ND/ND, D/ND, ND/D, D/D based on ctDNA detection at C1D1 vs C2D15.

Summaries of objective response (yes=objective response, and no=no objective response) will be provided for subgroups defined by the *BRAF* V600 alteration status (measurable or not-measurable) at baseline or as ND/ND, D/ND, ND/D, D/D based on ctDNA detection at C1D1 vs C2D15. Graphical analyses (eg stick plots, box plots, etc) will be provided when appropriate.

PFS and OS analyses will be stratified by BRAF V600 status at baseline (measurable or not-measurable) and/or changes in ctDNA detection status ND/ND, D/ND, ND/D, D/D [C1D1/C2D15])) and will use the same Kaplan-Meier method as described for PFS. Kaplan-Meier curves with appropriate summary statistics including median time and associated 2-sided 95% CIs will be calculated.

Diagnostic concordance assessment of *BRAF* V600E status between the plasma-based *BRAF* V600E test and the reference, central PCR-based QIAGEN *BRAF* V600E FDA-approved test will be presented for cfDNA diagnostics concordance analysis set. The reference, the central test is the tumor tissue PCR testing performed at screening as part of the enrollment criteria, and all participants are therefore considered *BRAF* V600E positive (ie, detected). Overall concordance will be summarized by treatment arm and in total.

### 6.3.4. Surgical conversion rate (exploratory endpoint)

For the Phase 3 and Cohort 3 portion of the study the surgical conversion rate, defined as the rate of participants who become eligible for surgery and undergo surgery with curative intent as a result of study intervention, will be evaluated in the safety analysis set. Summary data will be presented by treatment arm.



090177e1a0219102\Approved\Approved On: 08-Mar-2024 15:37 (GMT)

## 6.4. Subset Analyses

All the subset analyses will be exploratory and thus descriptive in nature; no adjustment for multiplicity will be performed. Analyses will only be performed if there is sufficient sample size. The determination of whether or not there is sufficient sample size will be defined after enrollment is complete and prior to database lock. As a general rule, time to event analyses will not be performed on subgroups unless there are at least 10 events or 10% of total events, the largest, within the defined subset. Analyses of response rates and safety will only be performed if there are at least 10 participants or 10% of total participants within the defined subset.

The following subset analyses will be performed for ORR by BICR, PFS by BICR and OS for Phase 3 FAS participants (Arm B vs. Arm C) and for ORR by BICR and PFS by BICR for Cohort 3 FAS participants:

- Age (<65 years vs ≥65 years).
- Gender (Male vs Female).
- ECOG performance status based on the IRT system (0 vs 1).
- Number of organs involved at baseline (≤2 vs ≥3) according to BICR.
- Side of tumor (left vs right). Classifications are below:
    - Descending (Left)
    - Rectosigmoid (Left)
    - Sigmoid (Left)
    - Rectum (Left)
    - Splenic Flexure (Left)
    - Cecum (Right)
    - Ascending (Right)
    - Transverse (Right)
    - Hepatic Flexure (Right)
- Presence of liver metastases at baseline, based on Target and Non-target lesion assessment (yes vs no), according to BICR.

In addition, the following subset analyses may be performed for ORR by BICR, PFS by BICR and OS for Phase 3 and Cohort 3 FAS participants to support country specific regulatory submission, if required. PK will also be analyzed (see Section 6.7). Results of these subset analyses may not be included in the main CSR.



090177e1a0219102\Approved\Approved On: 08-Mar-2024 15:37 (GMT)

- Japanese subgroup (participants randomized in Japan).
- Korean subgroup (participants randomized in Korea).
- Chinese subgroup (participants randomized in China).

Subset analyses for PFS, OS will use the censoring rules described in Section 6.1.2.1.1. The unstratified HR and its corresponding 95% CI will be calculated per subset level and will be presented in a forest plot.

## 6.5. Baseline and Other Summaries and Analyses

### 6.5.1. Baseline Summaries

The following analyses will be performed by cohort on the FAS for the SLI, and by treatment arm for the Phase 3 and for the Cohort 3 on the FAS.

#### 6.5.1.1. Demographic Summary

The following demographic parameters will be listed and summarized by number and percentage:

- Gender (male, female).
- Age (<18; 18-<65; ≥65; ≥75).
- Race (White, Black or African American, Asian, American Indian or Alaska Native, Native Hawaiian or other Pacific Islander, Other, Not Reported).
- Racial designation for Asian (Japanese, Korean, Chinese, other).
- Ethnicity (Hispanic/Latino, not-Hispanic/Latino).

Age (continuous) will be summarized with descriptive statistics (mean, median, standard deviation, minimum, and maximum).

#### 6.5.1.2. Baseline and Disease Characteristics

The following baseline characteristics and disease characteristics will be listed and summarized:

- Body site as collected on the “Primary Diagnosis” eCRF page.
- Stage at initial diagnosis.
- Primary tumor removed prior to study entry (No, Complete resection, Partial resection).
- Side of tumor (left vs right).
- Number of organs involved based on baseline target and non-target lesion assessment by investigator (SLI) or BICR (Phase 3 and Cohort 3).
- Presence of liver metastases at baseline, based on target and non-target lesion assessment by investigator (SLI) or BICR (Phase 3 and Cohort 3).



090177e1a0219102\Approved\Approved On: 08-Mar-2024 15:37 (GMT)

- Time since initial diagnosis, defined as [date of first dose (SLI) or date of randomization (Phase 3 and Cohort 3) – date of initial diagnosis]/30.4375.
- Time since recurrence/metastatic disease (months), defined as [date of first dose (SLI) or date of randomization (Phase 3 and Cohort 3) – date of recurrence/metastatic disease]/30.4375.
- ECOG Performance status.
- Line of therapy (for SLI only): first line if no prior treatment; second line if participant received a prior treatment defined as advanced/metastatic or locoregional disease or maintenance or if neoadjuvant/adjuvant with a disease recurrence occurred during or within 6 months of the last dose of therapy.
- Local *BRAF* V600E status as collected on the "Local BRAF Results" eCRF page combining results from tumor tissue and plasma sample.
- Central *BRAF* V600E status based on tumor tissue.
- *KRAS* and *NRAS* results as collected on the "Local KRAS Results" eCRF page combining results from tumor tissue and plasma sample.
- MSI/MRR results as collected on the "Local MSI/MRR Results" eCRF page combining results from tumor tissue and plasma sample.
- Baseline CEA values (≤ 5 ug/L vs. > 5 ug/L) and CRP values (≤ 10 mg/L vs. > 10 mg/L) and descriptive summary.

#### 6.5.1.3. Stratification Factors

The number of participants per strata will be summarized based on strata reported in the IRT system and strata reported in CRF as described in Section 3.4.1.

#### 6.5.1.4. Medical History

Medical history will be coded using the most current version of MedDRA and summarized by MedDRA's SOC and PT. Each participant will be counted only once within each PT or SOC. Summaries will be ordered by primary SOC and PT by decreasing frequency based on mFOLFOX6 Cohort for SLI, the Arm B for Phase 3, and Arm D for Cohort 3.

#### 6.5.1.5. Prior Anticancer Treatments

Prior anticancer treatments include systemic therapy, radiation, and surgery.

The number and percentage of participants in each of the following anticancer therapy categories will be tabulated:

- Participants with at least one prior anticancer systemic therapy (SLI).
- Participants with at least one prior anticancer neoadjuvant/adjuvant therapy (Phase 3 and Cohort 3).
- Participants with at least one prior anticancer radiotherapy.



090177e1a0219102\Approved\Approved On: 08-Mar-2024 15:37 (GMT)

- Participants with at least one prior anticancer surgery, excluding procedure=biopsy.

All prior anticancer therapy will be presented in a listing.

### 6.5.2. Study Conduct and Participant Disposition

The following analyses will be performed by cohort on the FAS for the SLI, and by treatment arm for the Phase 3 and for the Cohort 3 on the FAS.

#### 6.5.2.1. Patient Disposition

The disposition summary will be created for the SLI, the Phase 3 portion and the Cohort 3 of the study. A listing will also be presented.

The following disposition categories will be summarized:

- Total number of participants screened.
- Primary reasons for screening discontinuation.

Number (%) of participants who screened failed overall and grouped by the main reason

- Number (%) of participants who were randomized (Phase 3 and Cohort 3 only).
- Number (%) of participants who were randomized but not treated (Phase 3 and Cohort 3 only).
- Number (%) of participants who received at least one dose of study drug.
- Number (%) of participants who are still on treatment.
- Number (%) of participants who discontinued the treatment.
  - Primary reasons for treatment discontinuation.
- Number (%) of participants who discontinued the treatment but are still in long-term follow-up.
- Number (%) of patients who discontinued the study.
  - Primary reasons for study discontinuation.

The number and percent of participants enrolled by region, country and site, and the number and percent of participants in each analysis set as described in Section 4 will also be summarized. A listing of analysis sets will be provided. A listing will show the randomized/assigned treatment along with actual treatment received.

090177e1a0219102\Approved\Approved On: 08-Mar-2024 15:37 (GMT)

**COVID-19 Related Disposition**

A listing of all participants affected by COVID-19 related study disruption will be created. The listing will present subject number identifier by investigational site, and a description of COVID-19 related events including:

- Protocol deviations.
- Adverse events treatment.
- Treatment discontinuation.
- Study discontinuation.
- Death.

**Ukraine/Russia War Related Disposition**

A listing of all participants affected by Ukraine/Russia war related study disruption may be created.

#### 6.5.2.2. Protocol Deviations

Potentially important protocol deviations (PIPDs) will be compiled prior to database closure and will be summarized by category and also presented in a listing. Categories will be assigned by the study Clinician.

In addition, PIPDs related to COVID-19 will be presented in a separate table and listing.

### 6.5.3. Study Treatment Exposure

The following analyses will be performed by cohort for the SLI and by treatment arm for the Phase 3 and the Cohort 3 on safety analysis set.

**Treatment Regimens**

- Safety Lead-in.
  - Participants enrolled in the SLI will receive either a regimen of EC + mFOLFOX6 or a regimen of EC + FOLFIRI in 28 day cycles. Encorafenib is dosed at 300 mg daily; cetuximab, mFOLFOX6 and FOLFIRI are dosed once every 2 weeks with an IV infusion.
- Phase 3.
  - Arm A: Participants randomized to Arm A will receive encorafenib + cetuximab in 28 day cycles. Encorafenib is dosed at 300 mg daily and cetuximab is dosed once every 2 weeks with an IV infusion.



090177e1a0219102\Approved\Approved On: 08-Mar-2024 15:37 (GMT)

- Arm B: Participants randomized to Arm B will receive a regimen of EC + mFOLFOX6 in 28-day cycles. The dosing schedule is the same as the SLI.
- Arm C: Participants randomized to the Arm C will receive one of the standard-of-care chemotherapy per physician choice as described in Table 13 in the protocol. mFOLFOX6, and FOLFOXIRI with or without bevacizumab are dosed once every 2 weeks with an IV infusion; and CAPOX with or without bevacizumab is dosed once every 3 weeks with an IV infusion.
  Within Arm C, the number of participants receiving different regimens (mFOLFOX6, FOLFOXIRI, or CAPOX with or without bevacizumab) will be summarized.

- Cohort 3.
  - Arm D: Participants randomized to Arm D will receive a regimen of EC + FOLFIRI in 28-day cycles. The dosing schedule is the same as the SLI.
  - Arm E: Participants randomized to Arm E will receive FOLFIRI with or without bevacizumab and are dosed once every 2 weeks with an IV infusion, see Table 15 in the protocol.

**Duration of Exposure**

The date of receiving the last non-zero dose is considered as last dose date.

- Encorafenib: daily dosing.

  **duration of exposure** (weeks) = (last dose date – first dose date +1)/7

- Cetuximab, mFOLFOX6, FOLFIRI, bevacizumab: once every two weeks on 28-day Cycle for all drugs except 5-FU:

  **duration of exposure** (weeks) = (last dose date – first dose date +14)/7

  - 5-FU if the last dose is a bolus:

    **duration of exposure** (weeks) = (last dose date – first dose date +14)/7

  - 5-FU if the last dose is an infusion:

    **duration of exposure** (weeks) = (last dose date – first dose date +12)/7

- CAPOX (oxaliplatin + capecitabine):
  - Oxaliplatin: once every three weeks on 21-day cycle

    **duration of exposure** (weeks) = (last dose date – first dose date + 21)/7



090177e1a0219102\Approved\Approved On: 08-Mar-2024 15:37 (GMT)

- Capecitabine: twice daily for 2 weeks followed by a 1-week rest period once every 3 weeks

  **duration of exposure** (weeks) = (last dose date – first dose date + 7)/7

- Bevacizumab: once every three weeks on 21-day cycle

  **duration of exposure** (weeks) = (last dose date – first dose date +21)/7

- Overall regimen:

  **duration of exposure (weeks) =** (last dose date of the last drug (including + 1 or 7 or 14 or 12 or 21 depending on last drug type) – first dose date of the first drug)/7

**Cumulative Dose**

Cumulative dose is defined as:

- **Intended cumulative dose** (mg or mg/kg or mg/m$^2$) = sum of all protocol specified doses across each intended day of dosing, as per protocol.
- **Actual cumulative dose** (mg or mg/kg or mg/m$^2$) = sum of all actual doses that the participant received. The actual doses reported in CRF are converted to mg/kg or mg/m$^2$ based on height and weight at most recent collection on or before dosing. BSA is derived using the DuBois & DuBois formula

$$BSA = 0.007184 * Height^{0.725} * Weight^{0.425}.$$

For participants who did not take any drug the actual cumulative dose is by definition equal to zero.

**Dose Intensity**

- **Intended dose intensity** (mg/day or mg/kg/day or mg/m$^2$/day) = intended cumulative dose/duration of exposure.
- **Actual dose intensity** (mg/day or mg/kg/day or mg/m$^2$/day) = actual cumulative dose/duration of exposure.
- **Relative dose intensity** (%) = 100* (actual dose intensity/intended dose intensity).

A summary of exposure, including duration of exposure, cumulative dose, actual dose intensity, and relative dose intensity (including categories <50%, 50%-<75%, 75%-<90%, 90%-<110%, and ≥110%, if applicable), will be presented for each study drug. Duration of exposure will also be categorized by time intervals (eg, < 4 weeks, 4-12 weeks, 12-48 weeks, etc. as appropriate for the protocol) for which frequency counts and percentages of participants will be provided.

Actual doses administered will also be listed for each participant by treatment arm.



090177e1a0219102\Approved\Approved On: 08-Mar-2024 15:37 (GMT)

#### 6.5.3.1. Dose Modification

Dose modification will be summarized based on the dosing data collected on the study treatment CRF page. Dose reduction and interruption will be summarized for each study drug (Leucovorin and 5-FU will be summarized together). Time to first dose modification (reduction and/or interruption) will be calculated and summarized with descriptive statistics for participant with at least one dose modification.

**Dose Reduction**

A dose reduction is defined as a decrease in dose of at least 10%, from the protocol-planned dose and a decrease from the previous non-zero dose, even if this decrease has been directly preceded by an interruption. For encorafenib and capecitabine, to qualify as a dose reduction, it should last for 2 or more days. For example, for encorafenib, in the sequence of total daily dose 300 mg – 0 mg – 200 mg (for at least 2 days), the 200 mg dose will be counted as a reduction. Also, for patients taking FOLFIRI in Cohort 1 in the SLI, the encorafenib dose is not given for Day 1 and Day 2, with the first encorafenib dose planned on Day 3. Days 1 and 2 will not count toward any reductions. A day 3 encorafenib dose of 200 mg for example would constitute a dose reduction since 300 mg was planned at Day 3.

The number and percentage of participants with at least one dose reduction as well as a breakdown of dose reductions (1, 2, ≥3) will be summarized by cohort or treatment arm.

Reasons for dose reductions will also be summarized. Participants can contribute to more than one reason if multiple dose reductions occurred for different reasons, but will only be counted once per reason. Percentages will be calculated based on the total number of participants in the safety analysis set.

**Dose Interruption**

For daily dosing of encorafenib and capecitabine, an interruption is defined a 0 mg dose administered for one or more days. For intermittent dosing of IV drugs, such as cetuximab, Oxaliplatin, Leucovorin+5-FU, Irinotecan and bevacizumab, an interruption is defined as more than 20 days (delay of at least 7 days) between successive start dates with non-zero actual doses for regimens on 28-day cycles (treatment on D1 and D15 of every cycle) or as more than 27 days (delay of at least 7 days) between successive start dates with non-zero actual doses for regimens on 21-day cycles (treatment on D1 of every cycle). For capecitabine the off-week, that is part of the regimen, will not be counted as an interruption. For patients taking FOLFIRI in Cohort 1 of the safety lead-in, the planned encorafenib doses of 0 mg on Days 1 and 2 will not constitute a dose interruption.

The number and percentage of participants with dose interruptions will be summarized. Percentages will be calculated based on the total number of participants in the safety analysis set.

090177e1a0219102\Approved\Approved On: 08-Mar-2024 15:37 (GMT)

### 6.5.4. Concomitant Medications

Concomitant medications will be summarized by cohort on the FAS set for the SLI, and by treatment arm on the FAS for the Phase 3 and for the Cohort 3.

Concomitant medications are medications, other than study medications, which started prior to first dose of study treatment and continued during the on-treatment period (see Section 5.2.7) as well as those started during the on-treatment period. Concomitant medications will be coded in the WHO Drug coding dictionary and will be tabulated by ATC classification level 2 and PT by decreasing frequency based on mFOLFOX6 Cohort for SLI, the Arm B for Phase 3, and Arm D for Cohort 3. In case of equal frequency regarding drug class (respectively drug name), alphabetical order will be used. A participant will be counted only once within a given drug name, even if he/she received the same medication at different times.

### 6.5.5. Subsequent Anticancer Therapies/Procedures

Subsequent anticancer therapies/procedures will be summarized by cohort on the FAS set for the SLI, and by treatment arm on the FAS for the Phase 3 and for the Cohort 3.

Subsequent anticancer therapies are defined as therapies entered on the “Follow-up Cancer Therapy” CRF page. Subsequent anticancer radiation and surgery will be derived based on the data collected on the "Radiation Treatment” and “Non-Drug Treatments - Cancer Surgery” CRF page, respectively. The radiation treatments and the surgeries will be considered as the subsequent anticancer procedures if the date of the radiation/surgery is after the last dose date of all study drugs.

The number and percentage of participants within each category (medication therapy, radiation therapy, and surgeries) will be provided.

Medications will be coded using the WHO Drug coding dictionary and will be tabulated by drug category and PT by decreasing frequency based on mFOLFOX6 Cohort for SLI, the Arm B for Phase 3, and Arm D for Cohort 3. The final list of drug category will be provided upon medical review of all subsequent anti-cancer systemic treatment before database lock.

All subsequent anticancer therapy will be presented in a listing.

## 6.6. Safety Summaries and Analyses

All safety analyses will be performed on the safety analysis set by cohort for the SLI and by treatment arm for the Phase 3 and for the Cohort 3.

Safety data will be presented in tabular and/or graphical format and summarized descriptively, where appropriate.

### 6.6.1. Adverse Events

All analyses will be based on TEAEs as defined in Section 3.5.1 unless otherwise specified. AEs not considered treatment emergent will be flagged in data listings.



090177e1a0219102\Approved\Approved On: 08-Mar-2024 15:37 (GMT)

Seriousness, toxicity grade, action taken (interruption, reduction, and withdraw) of AEs are as reported by the investigator on the “Adverse Events” CRF.

A high level summary of AEs will include the number and percent of participants with:

- Any TEAEs.
- Serious TEAEs.
- TEAEs with CTCAE Grade 3 or 4.
- TEAEs with CTCAE Grade 5.
- Related TEAEs to:
    - Any study drug.
    - Encorafenib.
    - Cetuximab.
    - Other study intervention (i.e capecitabine, 5-FU, irinotecan, leucovorin, oxaliplatin, and bevacizumab).
- Related TEAEs to any study drug with CTCAE Grade 3 or 4.
- Related TEAEs to any study drug with CTCAE Grade 5
- Related serious TEAEs to:
    - Any study drug.
    - Encorafenib.
    - Cetuximab.
    - Other study intervention.
- TEAEs leading to dose interruption of:
    - Any study drug.
    - Encorafenib.
    - Cetuximab.
    - Other study intervention.

090177e1a0219102\Approved\Approved On: 08-Mar-2024 15:37 (GMT)

- TEAEs leading to dose reduction of:
    - Any study drug.
    - Encorafenib.
    - Cetuximab.
    - Other study intervention.
- TEAEs leading to permanent discontinuation from:
    - Any study drug.
    - Encorafenib.
    - Cetuximab.
    - Other study intervention.
- TEAEs leading to dose reduction of any study drug due to COVID-19.
- TEAEs leading to permanent discontinuation from any study drug due to COVID-19.

Each unique AE at the PT level in each cohort/treatment arm of the study for a participant is included in the count.

**<u>Adverse Events by SOC and PT</u>**

The following summaries will be created by SOC and PT by decreasing frequency based on mFOLFOX6 Cohort for SLI, the Arm B for Phase 3, and Arm D for Cohort 3:

- TEAEs by maximum toxicity grade (all causality).
- TEAEs by maximum toxicity grade (treatment related).
- COVID-19 related TEAEs (all causality).
- Serious TEAEs by maximum toxicity grade (all causality).
- Serious TEAEs by maximum toxicity grade (treatment related).
- TEAEs leading to dose reduction of any study drug due to COVID-19.
- TEAEs leading to permanent discontinuation from any study drug due to COVID-19.

An event will be considered treatment related if the investigator considered the event related to at least one study drug or this information is unknown.

090177e1a0219102\Approved\Approved On: 08-Mar-2024 15:37 (GMT)

**Adverse Events by PT Only**

The following summaries will be created by AE PT (ie, summaries will not include SOC) by decreasing frequency based on mFOLFOX6 Cohort for SLI, the Arm B Phase 3, and Arm D for Cohort 3:

- TEAEs (all causality) experienced by ≥10% of participants in at least one cohort (SLI) or treatment arm (Phase 3 and Cohort 3).
- Treatment related TEAEs experienced by ≥10% of participants in at least one cohort (SLI) or treatment arm (Phase 3 and Cohort 3).
- TEAEs with CTCAE Grade ≥3 (all causality) by PT and maximum toxicity grade.
- Serious TEAEs (all causality).
- COVID-19 related serious TEAEs (all causality).

A cutoff may be applied to some of the above AE tables for intext display.

**Adverse Events Leading to Dose Modification by PT**

The following summaries will be created by PT of AEs by decreasing frequency of PTs based on mFOLFOX6 Cohort for SLI, the Arm B for Phase 3, and Arm D for Cohort 3:

- TEAEs leading to dose interruptions by maximum toxicity grade (all causality).
- TEAEs leading to dose reductions by maximum toxicity grade (all causality).
- TEAEs leading to permanent discontinuation by maximum toxicity grade (all causality).
- TEAEs leading to dose interruptions by maximum toxicity grade (treatment related).
- TEAEs leading to dose reductions by maximum toxicity grade (treatment related).
- TEAEs leading to permanent discontinuation by maximum toxicity grade (treatment related).

The AE by PT summary will be created separately for:

- Any study drug.
- Encorafenib.
- Cetuximab.



090177e1a0219102\Approved\Approved On: 08-Mar-2024 15:37 (GMT)

- Other study intervention.

A cutoff may be applied to the tables for AE leading to dose modification. The treatment related tables, for AE leading to dose modification, will include AEs treatment related to a specific drug for which the dose modification was implemented (i.e. treatment related to encorafenib for AEs leading to dose reduction of encorafenib). Each participant will be counted only once within each PT.

All AEs and their attributes will be presented in data listings sorted by participant identifier, AE and date of onset of the AE.

### 6.6.2. Deaths

All deaths, deaths within 28 days after last dose of study drug, deaths within 30 days after first dose of study drug as well as the primary reason for death, will be tabulated based on information from the “Death Details” and “Disposition Long-term Follow-Up” eCRFs.

- Number of deaths.
- Number of deaths within 28 days after *last* dose of study treatment.
- Number of deaths within 30 days after *first* dose of study treatment.
- Primary cause of death.
  - Disease under study.
  - Study treatment toxicity.
  - Unknown.
  - Other.

In addition, if there are ≥5 deaths due to COVID-19, a separate death summary will be created.

Date and cause of death will be provided in patient data listing together with selected dosing information (study treatment received, date of first/last administration, dose).

### 6.6.3. Adverse Events of Special Interest

Specific groupings of AEs of special interest (AESI) will be considered and the number of patients with at least one event in each grouping will be reported. Such groups consist of AEs for which there is a specific clinical interest in connection with encorafenib treatment (ie, where encorafenib may influence a common mechanism of action responsible for triggering them) or AEs which are similar in nature (although not identical), and there is potential overlap with the safety profile of cetuximab and/or chemotherapy.



090177e1a0219102\Approved\Approved On: 08-Mar-2024 15:37 (GMT)

090177e1a0219102\Approved\Approved On: 08-Mar-2024 15:37 (GMT)

All AESI groupings are defined through the use of PT, High Level Terms (HLT), SOC, Standardized MedDRA Queries (SMQ) or through a combination of these components. The MedDRA terms that define each AESI grouping will be outlined in the Case Retrieval Strategy (CRS), which will be used to map reported AEs to the notable AEs groupings and will be maintained by the Sponsor. The list of AESIs may be updated during the course of the trial based on accumulating safety data.

A high level summary of AESIs will include the number and percent of participants with:

- Any AESIs.
- Serious AESIs.
- AESIs with CTCAE Grade 3 or 4.
- AESIs with CTCAE Grade 5.
- Related AESIs.
- Related AESIs with CTCAE Grade 3 or 4.
- Related AESIs with CTCAE Grade 5.
- Related serious AESIs.

In addition, AESIs will be summarized by grouping and PT as follows:

- AESIs by maximum toxicity grade (all grade, Grade 3, Grade 4, Grade 5, all causality).
- AESIs by maximum toxicity grade (all grade, Grade 3, Grade 4, Grade 5, treatment related).
- Serious AESIs all grade (all causality).
- Serious AESIs all grade (treatment related).

A cutoff may be applied to some of the above AESI tables, for intext display.

### 6.6.4. Laboratory Data

Laboratory results will be converted to International System of Units (SI) units which will be used for applying toxicity grades and for all summaries.

As described in Section 3.4, baseline is defined as the last completed assessment prior to date of first dose for the safety assessments. If there are multiple assessments that meet the baseline definition on the same day without the ability to determine which was truly last, then the worst grade will be assigned as the baseline grade.

Laboratory results will be programmatically classified according to NCI-CTCAE version 4.03 grade. Non-numerical qualifiers will not be taken into consideration in the derivation of

grade (eg, hypokalemia Grade 1 and Grade 2 are only distinguished by a non-numerical qualifier and therefore Grade 2 will not be derived). In summary statistics the number and percentage of participants corresponding to grades that only include non-quantitative criteria will be displayed as a blank or NA (not assessed) rather than 0. If there is any overlap between grade criteria (eg, CTCAE grading criteria for Creatinine Increased – a value can fall into one range based on comparison to ULN and another range based on comparison to baseline), the highest (worst) grade would be assigned to that record. Grade 5 is defined in the CTCAE criteria guidance as an event with an outcome of death. Since laboratory data does not collect an outcome, Grade 5 is not used when programmatically grading laboratory data.

Grade 0 or Outside Toxicity Reference (OTR) is not defined specifically in the CTCAE guidance. However, programmatically this is used as a category to represent those participants who did not meet any of the Grades 1 to 4 criteria. If the laboratory value is evaluable for CTCAE criteria grading (numeric value is present, valid units and ranges are present as required to allow conversion to standard units and grading), and does not qualify for any of the Grade 1-4 criteria for a given lab test, then the value is assigned as Grade 0 or OTR.

Abnormalities will be described using the worst grade post-baseline. When determining the maximum post-baseline grade for a given participant and CTCAE test, the maximum across all analytes and assessments contributing to that CTCAE test will be used. Several laboratory tests have bi-directional grading criteria defined so that both low (hypo) and high (hyper) values can be graded separately. Each criterion will be summarized separately. In the cases where a value is graded as a Grade 1, 2, 3, or 4 for one of the directions, that value will also be assigned as a Grade 0 for the opposite direction for that test. For example, a value meeting the criteria for Grade 3 Hypercalcemia will be classified as a Grade 0 Hypocalcemia. For CTCAE terms that can be derived using one of several laboratory tests, the maximum post-baseline grade for a given participant and CTCAE term will be the maximum across all possible laboratory tests.

For **WBC differential counts** (total neutrophil [including bands], lymphocyte, monocyte, eosinophil, and basophil counts), the absolute value will be used when reported by the lab. When only percentages are available (this is mainly applicable for neutrophils and lymphocytes, because the CTCAE grading is based on the absolute counts), the absolute value is derived as follows:

Derived differential absolute count = (WBC count) × (Differential %value/100)

If the investigator reports both the absolute and % value for Neutrophils or Lymphocytes from the same laboratory sample date and participant, ONLY the absolute value will be graded. The % value will not be graded in this scenario.

090177e1a0219102\Approved\Approved On: 08-Mar-2024 15:37 (GMT)

If the % value is converted to the differential absolute count for grading and the LLN for the differential absolute count is not available (only LLN for % is available) then Grade 1 will be assigned if the following conditions are met:

- Lymphocyte count decreased:
  - derived absolute count does not meet Grade 2-4 criteria, and
  - % value < % LLN value, and
  - derived absolute count ≥800/$mm^3$.
- Neutrophil count decreased
  - derived absolute count does not meet Grade 2-4 criteria, and
  - % value < % LLN value, and
  - derived absolute count ≥1500/$mm^3$.

For **calcium**, CTCAE grading is based on Corrected Calcium and Ionized Calcium. Corrected Calcium is calculated from Albumin and Calcium as follows:

- Corrected Calcium (mg/dL) = measured total calcium (mg/dL) – 0.8 [serum albumin (g/dL)-4].

Laboratory toxicities will be tabulated using descriptive statistics (number of participants and percentages). If both central and local labs are collected for a subject, summaries of worst on-treatment abnormalities will be based on both local and central lab data.

Additional laboratory results that are not part of NCI-CTCAE will be presented according to the categories: below normal limit, within normal limits and above normal limit (according to the laboratory normal ranges). The following summary tables will be created:

- Shift summary of laboratory parameters during the on-treatment period by maximum CTCAE grade.
- Shift summary of laboratory parameters from ≤ Grade 2 at baseline to ≥ Grade 3 post-baseline.
- Shift summary of laboratory test results with no CTCAE criteria by worst (below, within or above normal range) on-treatment assessment.

All laboratory test results will be presented in a data listing sorted by patient identifier, laboratory test, and date/time of collection. The CTCAE grades and the classifications relative to the laboratory reference ranges will be presented. Values outside laboratory



090177e1a0219102\Approved\Approved On: 08-Mar-2024 15:37 (GMT)

normal ranges will be flagged where appropriate and the central laboratory data and local laboratory data will be flagged accordingly.

**Drug Induced Liver Toxicity**

Alanine aminotransferase (ALT), aspartate aminotransferase (AST), Alkaline Phosphatase (ALP), and total bilirubin (TBILI) are used to assess possible drug induced liver toxicity. The ratios of test result over the ULN will be calculated and classified for these three parameters during the on-treatment period.

Summary of liver function tests will include the following categories. The number and percentage of participants with each of the following during the on-treatment period will be summarized:

- ALT ≥3×ULN, ALT ≥5xULN, ALT ≥10×ULN, ALT ≥20×ULN.
- AST ≥3×ULN, AST ≥5xULN, AST ≥10×ULN, AST ≥20×ULN.
- (ALT or AST) ≥3×ULN, (ALT or AST) ≥5×ULN, (ALT or AST) ≥10×ULN, (ALT or AST) ≥20×ULN.
- TBILI ≥2×ULN.
- Concurrent ALT ≥3×ULN and TBILI ≥2×ULN.
- Concurrent AST ≥3×ULN and TBILI ≥2×ULN.
- Concurrent (ALT or AST) ≥3×ULN and TBILI ≥2×ULN.
- Concurrent (ALT or AST) ≥3×ULN and TBILI ≥2×ULN and ALP ≥2×ULN.
- Concurrent (ALT or AST) ≥3×ULN and TBILI ≥2×ULN and ALP <2×ULN or missing.

Concurrent measurements are those occurring on the same date.

Categories will be cumulative, ie, a participant with an elevation of AST ≥10×ULN will also appear in the categories ≥5×ULN and ≥3×ULN. Liver function elevation and possible Hy's Law cases will be summarized using frequency counts and percentages.

An evaluation of Drug-Induced Serious Hepatotoxicity (eDISH) plot will also be created, with different symbols for different treatment arms, by graphically displaying:

- Peak serum ALT (/ULN) vs peak total bilirubin (/ULN) including reference lines at ALT=3×ULN and total bilirubin=2×ULN.

090177e1a0219102\Approved\Approved On: 08-Mar-2024 15:37 (GMT)

- Peak serum AST(/ULN) vs peak total bilirubin (/ULN) including reference lines at AST=3×ULN and total bilirubin=2×ULN.

In addition, a listing of all TBILI, ALT, AST and ALP values for participants with a post-baseline TBILI ≥2×ULN, ALT ≥3×ULN or AST ≥3×ULN will be provided.

### 6.6.5. Vital Signs

Vital signs data includes weight, pulse, systolic blood pressure (BP), and diastolic BP. Measurements were only to be provided once per timepoint. If multiple assessments are provided per timepoint, the maximum value will be used for reporting.

The following criteria define clinically notable vital sign abnormalities:

- Clinically notable elevated values.
  - Systolic BP: ≥160 mmHg and an increase ≥20 mmHg from baseline.
  - Diastolic BP: ≥100 mmHg and an increase ≥15 mmHg from baseline
  - Heart rate (collected as pulse rate in the vital signs eCRF): ≥120 bpm with increase from baseline of ≥15 bpm
  - Weight: increase from baseline of ≥10%
  - Body temperature [C]: ≥37.5 C
- Clinically notable low values
  - Systolic BP: ≤90 mmHg with decrease from baseline of ≥20 mmHg;
  - Diastolic BP: ≤50 mmHg with decrease from baseline of ≥15 mmHg;
  - Heart rate (collected as pulse rate in the vital signs eCRF): ≤50 bpm with decrease from baseline of ≥15 bpm;
  - Weight: ≥20% decrease from baseline;
  - Body temperature [C]: ≤36 C.

All assessments, including unscheduled assessments will be considered. A participant can be included in multiple categories if different criteria are met at different timepoints.

Number and percentage of participants with at least one post-baseline vital sign abnormality will be summarized.

090177e1a0219102\Approved\Approved On: 08-Mar-2024 15:37 (GMT)

### 6.6.6. Electrocardiograms

As per protocol, baseline ECG will be obtained as the mean of the triplicate measurements collected at Cycle 1 Day 1 predose.

Heart rate, QT, PR and QRS data will be collected on the ECG eCRF page. QTcF (Fridericia correction) will be derived as follows:

- Fridericia's formula: QTcF= QT/ $\sqrt[3]{RR}$, where RR = 60/heart rate

Number and percentages of participants with clinically notable ECG values during the on-treatment period will be summarized. All participants reporting a clinical notable ECG value will be presented in data listings sorted by participant identifier and ECG date. The clinically notable ECG values are defined in Table 15.

**Table 15 Clinical Notable ECG Criteria**

| Parameter | Criterion |
|---|---|
| QTcF | increase from baseline >30 ms |
| | increase from baseline >60 ms |
| | new >450 ms |
| | new >480 ms |
| | new >500 ms |
| PR Interval | new > 280 ms |
| QRS | new > 120 ms |
| Heart rate | increase from baseline >25% and to a value >100 bpm |
| | decrease from baseline >25% and to a value <50 bpm |

ms: milliseconds

## 6.7. Summaries for Korea, Japan and China

The following analyses by country (Korea, Japan, China) may be performed to support country specific regulatory submission, if required. Results of these analyses may not be included in the main CSR. The appropriate SLI, Phase 3 and Cohort 3 portions will be noted below.

### 6.7.1. Efficacy Analyses

ORR by BICR, PFS by BICR and OS will be analyzed by country for Phase 3 and for Cohort 3, and potentially for SLI (if required for submission in Japan). The full analysis set or full analysis set, ORR subset will be used. See Section 6.4 for details.

090177e1a0219102\Approved\Approved On: 08-Mar-2024 15:37 (GMT)

Additionally, ORR and PFS by derived investigator, DOR, TTR (by BICR and by derived investigator assessment) and PFS2 will also be analyzed by country for Phase 3 and Cohort 3 (except PFS2), and potentially for SLI (if required for submission in Japan), utilizing the full analysis set or full analysis set, ORR subset.

### 6.7.2. Safety Analyses

The following analyses will be performed. See Sections 6.6.1, 6.6.2, 6.6.4, and 6.6.5 for details. The SLI, the Phase 3 and Cohort 3 utilizing the safety analysis set will be reported.

- A high-level summary of AEs (see Section 6.6.1)
- AEs by SOC and PT (all causalities)
- AEs leading to dose modification by PT
  - Dose interruption summary (all causalities), done for any drug, for encorafenib, for cetuximab, and for other study intervention
  - Dose reduction summary (all causalities), done for any drug, for encorafenib, for cetuximab, and for other study intervention
  - Dose permanently discontinued summary (all causalities), done for any drug, for encorafenib, for cetuximab, and for other study intervention
- Deaths
- Laboratory shift tables
- Vital signs

### 6.7.3. Other Analyses

The following other analyses will be performed for SLI, Phase 3 and Cohort 3:

- Demographic summary (full analysis set)
- Baseline and disease characteristics (full analysis set)
- Patient disposition (full analysis set)
- Protocol deviations (full analysis set)
- Study treatment exposure (safety analysis set)
- Subsequent anticancer therapies/procedures will be summarized (full analysis set)



090177e1a0219102\Approved\Approved On: 08-Mar-2024 15:37 (GMT)

- Pharmacokinetic analyses (PK analysis set). See Sections 6.3.2.2 and 6.3.2.3 for details of Phase 3 and Cohort 3, respectively.

The following other analyses will be performed for the SLI when data are appropriate:

- Pharmacokinetic analyses (PK analysis set). See Section 6.3.2.1 for details.

# 7. INTERIM ANALYSES

## 7.1. Introduction

Depending on the results of ORR and PFS analysis for the Phase 3, there will be two, one or no interim for efficacy analyses of OS.

## 7.2. Interim Analyses and Summaries for Efficacy OS

At each analysis timepoint, the critical boundaries for the group sequential test will be derived from the predefined spending functions as described below for OS of the Phase 3 portion of the study. The calculations of boundaries will be performed using EAST software (Cytel).[3]

No futility analysis will be performed for OS. Let u(t1) and u(tF) denote the upper critical boundaries based on the test statistics $Z_1$ and $Z_F$ for efficacy at the interim and the final analysis, respectively.

In what follows $P_0$ denotes the probability under the null hypothesis, and $\alpha(t_1)$ denotes the α spent based on the predefined α-spending function at information fraction t1 (t1 is calculated as the ratio of the number of OS events observed at the time of the data cutoff for the interim analysis and the total number of OS events targeted for the final analysis).

The critical value $u(t_1)$ for the interim analysis of OS is determined such as

$$P_0(Z_1 \geq u(t_1)) = \alpha(t_1).$$

The boundary for the final efficacy analysis is derived such that

$$\alpha(t_2) + P_0(Z_1 < u(t_1), Z_F \geq u_F) = 0.001 \ or \ 0.023.$$

As described below, if the number of OS events in the final analysis deviates from the target number of OS events, the final analysis criteria will be determined as above taking into account the actual alpha spent at the interim analysis and the actual correlation between the 2 test statistics $Z_1$ and $Z_F$, so that the overall 1-sided significance level across all analyses and comparisons is preserved at 0.001 or 0.023.

If the hypothesis test of the ORR by BICR Arm B versus Arm C (on 110 participants for each arm) is statistically significant, an interim analysis for superiority will be conducted at that time on the corresponding OS comparison (on all participants of the 2 arms) using a portion of overall nominal alpha=0.001. It is anticipated that this will occur at approximately



090177e1a0219102\Approved\Approved On: 08-Mar-2024 15:37 (GMT)

40% information in the Arm B versus Arm C OS comparison (ie, approximately 119 OS events in these two arms).

If the hypothesis test of the PFS Arm B versus Arm C is statistically significant, an interim analysis for superiority will be conducted at that time on the corresponding OS comparison using a portion of overall nominal alpha=0.023. If the results of PFS Arm B versus Arm C is not statistically significant, but results for ORR are statistically significant, an interim analysis for superiority will be conducted at that time of PFS analysis on the corresponding OS comparison using a portion of overall nominal alpha=0.001. It is anticipated that this will occur at approximately 80% information in the Arm B versus Arm C OS comparison (ie, approximately 238 OS events in these two arms).

A Lan-DeMets alpha-spending function[1] that approximates O'Brien-Fleming stopping boundaries will be used to control the overall type I error rate at 0.001/0.023 level (1-sided).

Table 16 summarizes the operating characteristics and efficacy boundaries for the OS analysis. The exact efficacy boundaries and the actual alpha spent at the interim analyses will be updated at the time of the analyses based on the actual number of events and the information fraction.

The interim analysis of OS will be performed using the methodology described in Section 6.2.2.1.1.

### Table 16 OS – Summary of Planned Efficacy Boundaries

| Formal Testing | Analysis | Information Fraction[a] | Cumulative OS Events | Efficacy Boundaries on P-scale (1-sided) |
|---|---|---|---|---|
| **In case only ORR analysis results are statistically significant** | | | | |
| Arm B vs Arm C | Interim Efficacy 1 | 40% | 119 | <0.0001 |
| | Interim Efficacy 2 | 80% | 238 | 0.0002 |
| | Final | 100% | 297 | 0.0009 |
| **In case ORR and PFS[b] or only PFS analyses results are statistically significant** | | | | |
| Arm B vs Arm C | Interim Efficacy | 80% | 238 | 0.0111 |
| | Final | 100% | 297 | 0.0198 |

The efficacy boundaries are determined using Lan-DeMets alpha spending function that approximates O'Brien-Fleming stopping boundaries.

a. The exact efficacy boundaries and the actual alpha spent on each analysis will be updated based on the actual number of events observed.
b. In case ORR is significant, interim efficacy 1 is conducted at p-value <0.0001



090177e1a0219102\Approved\Approved On: 08-Mar-2024 15:37 (GMT)

Interim analysis results may be used for decisions regarding stopping for early success in OS. Participants may be discontinued from the study as a result of the interim analysis.

090177e1a0219102\Approved\Approved On: 08-Mar-2024 15:37 (GMT)

# 8. REFERENCES


1. Lan K, DeMets DL. Discrete sequential boundaries for clinical trials. Biometrika. 1983;70(3):659-63.

2. Bretz F, Maurer W, Brannath W, Posch M. A graphical approach to sequentially rejective multiple test procedures. Statistics in Medicine 2009; 28: 586-604.

3. East® version 6.5 manual (2018). Cytel Inc. Cambridge, MA USA.

4. Wilson EB. Probable Inference, the Law of Succession and Statistical Inference. J Amstat Assoc. 1927;22(158):209-12.

5. Jennison C, Turnbull BW. Group sequential methods with applications to clinical trials: CRC Press; 1999.

6. Brookmeyer R and Crowley J. A confidence interval for the median survival time. Biometrics 38:29-41, 1982.

7. Schoenfeld D. Chi-squared goodness-of-fit tests for the proportional hazards regression model. Biometrika 1980; 67:145-53.

8. Therneau TM, Grambsch PM. Modeling Survival Data: Extending the Cox Model. Statistics for Biology and Health 2000.

9. Zhang X. Comparison of restricted mean survival times between treatments based on a stratified Cox model. DOI 10.1515/bams-2013-0101 Bio-Algorithms and Med-Systems 2013; 9(4): 183–9.

10. Kalbfleisch JD, Prentice, RL. Statistical Analysis of Failure Time Data, 2nd Edition.Hoboken, Wiley Interscience.

11. Amit O, et al. Blinded independent central review of progression in cancer clinical trials: Results from a meta-analysis and recommendation from a PhRMA working group. European Journal of Cancer 47:1772-1778, 2011.

12. Fayers PM, Aaronson NK, Bjordal K, Groenvold M, Curran D, Bottomley A, on behalf of the EORTC Quality of Life Group. EORTC QLQ-C30 Scoring Manual, 3rd edn. Brussels, EORTC, 2001.


090177e1a0219102\Approved\Approved On: 08-Mar-2024 15:37 (GMT)

# 9. APPENDIX 1 LIST OF ABBREVIATIONS

| Abbreviation | Term |
|---|---|
| AE | adverse event |
| AESI | adverse event of special interest |
| ALP | alkaline phosphatase |
| ALT | alanine aminotransferase |
| AST | aspartate aminotransferase |
| ATC | Anatomic Therapeutic Chemical |
| AUC | area under the curve |
| BICR | blinded independent central review |
| BP | blood pressure |
| BLQ | below the limit of quantification |
| BOR | Best overall response |
| BRAF | B-RAF proto-oncogene, serine/threonine-protein kinase |
| CRS | case retrieval strategy |
| cfDNA | circulating free DNA |
| ctDNA | circulating tumor DNA |
| CI | confidence interval |
| $C_{max}$ | maximum observed concentration |
| CR | complete response |
| CSR | clinical study report |
| CV | coefficient of variation |
| CTCAE | Common Terminology Criteria for Adverse Events |
| DLT | dose-limiting toxicity |
| DOR | duration of response |
| EC | encorafenib plus cetuximab |
| ECOG | Eastern Cooperative Oncology Group |
| ECG | electrocardiogram |
| eCRF | electronic case report form |
| E-DMC | external data monitoring committee |
| EDR | early discrepancy rate |
| EORTC QLQ-C30 | European Organisation for Research and Treatment of Cancer Quality of Life Questionnaire for Cancer Patients – 30 Item Core Questionnaire |
| EOT | end of treatment |
| EQ-5D-5L | EuroQol-5D-5L |
| EQ VAS | EuroQol-visual analogue scale |
| FAS | full analysis set |
| FOLFIRI | fluorouracil/leucovorin/irinotecan |



090177e1a0219102\Approved\Approved On: 08-Mar-2024 15:37 (GMT)

| Abbreviation | Term |
|---|---|
| FOLFOXIRI | fluorouracil/leucovorin/oxaliplatin/irinotecan |
| HLT | High level terms |
| HR | hazard ratio |
| IRT | interactive response technology |
| IV | intravenous |
| LDR | late discrepancy rate |
| LLOQ | Lower limit of quantification |
| mCRC | metastatic colorectal cancer |
| MedDRA | Medical Dictionary for Regulatory Activities |
| mFOLFOX6 | modified fluorouracil/leucovorin/oxaliplatin |
| MWPC | meaningful within person change |
| NA | not applicable |
| NCI | National Cancer Institute |
| ND | not done or no disease at baseline |
| NE | non-evaluable |
| NR | not reached |
| ORR | objective response rate |
| OS | overall survival |
| OTR | outside toxicity reference |
| PD | progressive disease |
| PD2 | second objective disease progression |
| PFS | progression-free survival |
| PFS2 | PFS after next line of treatment |
| PIPDs | potentially important protocol deviations |
| PK | pharmacokinetic(s) |
| PR | partial response |
| PRO | patient-reported outcome |
| PT | preferred term |
| Q1 | first quartile |
| Q3 | third quartile |
| QT | QT interval |
| QTcF | corrected QT (Fridericia method) |
| RECIST | Response Evaluation Criteria in Solid Tumors |
| RMST | restricted mean survival time |
| SAE | serious adverse event |
| SAP | statistical analysis plan |
| SD | stable disease |
| SLI | Safety Lead-in |



090177e1a0219102\Approved\Approved On: 08-Mar-2024 15:37 (GMT)

| Abbreviation | Term |
|---|---|
| SMQ | standardized MedDRA queries |
| SOC | standard of care or system organ class |
| SOP | standard operating procedure |
| TEAE | treatment-emergent adverse event |
| TTR | Time to response |
| ULN | upper limit of normal |
| vs | versus |
| WBC | white blood cell |
| WHO | World Health Organization |

090177e1a0219102\Approved\Approved On: 08-Mar-2024 15:37 (GMT)

# 10. APPENDIX 2 SPECIFICATIONS FOR PROGRAMMATIC DERIVATION OF TUMOR RESPONSE USING RECIST 1.1

For Phase 3 portion and Cohort 3, the primary efficacy analysis will be based on blinded independent central review (BICR) interpretation of tumor assessment scans from study sites, performed by Calyx. The details of this process are described in the study specific Calyx charter. Analyses based on BICR will use data as transferred from BICR without any further derivation (i.e. using the response at each timepoint provided by Calyx) except to account for censoring.

For SLI portion, the efficacy analysis will be based on investigator tumor assessment reported in the eCRF. Analyses based on investigator will use data as reported without any further derivation (i.e. using the response at each timepoint provided by the investigator) except to account for censoring.

For Phase 3 portion and Cohort 3, a secondary efficacy analysis will be based on a programmatic approach to derive tumor response/progression, using RECIST 1.1 and the investigator tumor assessment data recorded on the target, non-target, and new lesion electronic case report forms (eCRF), as described in this appendix.

The tumor response criteria are adapted from RECIST 1.1 (Eisenhauer EA, Therasse P, Bogaerts J, et al. New response evaluation criteria in solid tumours: Revised RECIST guideline [Version 1.1]. Eur J Cancer. 2009;45(2):228-47).

## 10.1. Study Specific Information

### 10.1.1. Adequate Baseline

The following must be met to qualify for "Adequate Baseline" assessment:

- Baseline assessments of all lesions (target, and non-target) must be prior to the first dose date (SLI) or date of randomization (Phase 3 and Cohort 3) per Schedule of Activities in Section 1.3 of the protocol. For Phase 3 and Cohort 3 lesions assessed after randomization, but before or on the first dose date will also be considered adequate.

- All lesions recorded at baseline must have an associated status recorded on the eCRF. For target lesions, an actual measurement should be recorded and it should meet the criterion for being measurable; for non-target lesions the actual status at baseline should be checked on the eCRF. For example, if 3 target lesion sites of disease are recorded all 3 must have associated measurements recorded; otherwise, the sum of longest dimensions (SOD) cannot be calculated.

- Baseline lesions must be assessed with an acceptable method of tumor assessment as specified in the protocol and could include: Conventional CT Scan, Spiral CT Scan, X-ray, MRI, PET/CT, physical examination and Bone Scan.



090177e1a0219102\Approved\Approved On: 08-Mar-2024 15:37 (GMT)

### 10.1.2. Interchangeable Methods of Tumor Assessment

Following tumor assessment methods are considered “interchangeable”: Conventional CT, Spiral CT, Enhanced CT, MRI, PET/CT.

### 10.1.3. Period for Derivation of Best Overall Response (BOR)

Is defined as the time from the start date until progressive disease (PD) or the day before initiation of new anticancer therapy in the absence of PD or death, whichever occurs first.

Assessments done after PD or starting with the day of new anticancer therapy will not be considered for evaluation of BOR.

### 10.1.4. New Anticancer Therapy

Includes any systemic anticancer therapy (other than study medication), radiation therapy to target or non-target lesions with curative intent, or surgery (including metastasectomy) for target or non-target lesions with outcome of “resected” or “partially resected” provided on or after the start date, see also Section 5.2.9.

## 10.2. Lesion Evaluation at Assessment Times

Often, at pre-planned and unplanned tumor assessment times (visits or weeks), there are multiple dates associated with evaluation of the same target, non-target and new lesions. In addition, tumor evaluations are not always recorded on the eCRF under a specific visit (week) that would allow identifying the lesions/investigator overall tumor assessment (IOTA) that were evaluated at that particular visit (week). In order to address this and group lesion (target, non-target and new) evaluations and IOTA assessment to the corresponding actual post-baseline tumor assessment visit (week), a clustering algorithm is applied. Each cluster represents an actual unique assessment date. For each patient, the number of clusters is equal to the maximum number of unique assessment dates available among all lesions (target, non-target, and new) and IOTA assessments. The SAS procedure, Proc Fastclus, is applied to a variable that represents the days from the start date to the unique date of the evaluation/assessment for each lesion/IOTA (date of scan – start date +1). Then the evaluations/assessments of lesions/IOTA that occurred “close to each other in time” will be assigned to the same cluster.

### 10.2.1. Response Evaluation of Target Lesions

Complete Response (CR) is defined by the disappearance of all non-lymph node target lesions (where all target lesions are recorded with a length of 0 mm on the “Target Lesions” eCRF). Any pathological lymph nodes (recorded as target lesion) must have reduction in short axis to <10 mm.

- Note: the SOD may not be zero if lymph nodes are included as target lesions.

Partial Response (PR) is defined by a 30% or more decrease in SOD of target lesions, taking as reference the baseline SOD.



090177e1a0219102\Approved\Approved On: 08-Mar-2024 15:37 (GMT)

PD is defined by a 20% or more increase in the SOD of target lesions relative to nadir (smallest SOD considering baseline and all assessments prior to the time point under evaluation), with a minimum absolute increase of 5 mm relative to nadir.

- Note: if only a subset of target lesions are assessed and the sum of the non-missing lesion diameters results in an increase above of at least 5 mm and at least 20% above the nadir, then the SOD will still be calculated and the progression criteria will have been otherwise met. If the sum of non-missing lesions does not indicate PD, then the SOD will be left as missing.

Stable Disease (SD) is assigned when neither sufficient shrinkage to qualify for CR or PR, nor sufficient increase to qualify for PD is observed, taking as reference the nadir.

No Target Lesion at Baseline (NB) is assigned if "No Target Lesion" is checked, on the "Target Lesions" eCRF at baseline.

Not All Evaluated (NAE) is assigned if, in the absence of PD based on evaluated target lesions:

- Any individual target lesion is evaluated as "Indeterminate" on the "Target Lesions" eCRF.
- Inconsistent methods (unless considered "interchangeable") are used for any target lesions after start date.
- One or more target lesions are not assessed.
- One or more target lesions were excised or irradiated and have not reappeared or increased.

Determination of target lesion response in case of reappearance of one or more target lesion(s) that have previously disappeared:

- If the previous target lesion response was CR, and a non-lymph node target lesion reappears, then the response is always PD.
- If the previous target lesion response was CR, and a lymph node target lesion reappears, then the response (whether CR or PD) is assessed based on PD criteria noted above. The response will be PD only if SOD criterion for PD is met and if the lymph node returns to pathologic size (≥10 mm) and meets the absolute requirement of 5 mm increase over nadir for the reappearing lesion. Otherwise, the response is CR.
- If the previous target lesion response was PR, then the response should be evaluated based on the SOD.

090177e1a0219102\Approved\Approved On: 08-Mar-2024 15:37 (GMT)

**Notes:**

- The SOD is only considered if the methods of assessment are consistent with baseline. The "interchangeable" methods noted in Section 10.1.2 above are all considered consistent methods.

- In the SOD, the longest diameter will be used for non-nodal lesions and the short axis dimension will be used for each lymph node included in the sum.

### 10.2.2. Response Evaluation of Non-Target Lesions

CR is defined by the complete disappearance of all non-target lesions (where all non-target lesions are marked "Absent" on the "Non-Target Lesion" eCRF). All lymph nodes must be non-pathological in size (<10 mm in short axis).

Non-CR/Non-PD is defined by persistence of one or more non-target lesions (ie, if any non-target lesions are marked "Present/Not Increased" on the "Non-Target Lesion" eCRF).

No NB is assigned if "No Non-Target Lesions" is marked on the "Non-Target Lesions" eCRF at baseline.

NAE is assigned if, in the absence of PD based on evaluated non-target lesions:

- Any individual non-target lesion is evaluated as "Indeterminate" (marked as "Indeterminate" on the "Non-Target Lesions" eCRF).

- Inconsistent methods (unless considered "interchangeable" as noted in Section 10.1.2) are used for any non-target lesions after start date.

- One or more non-target lesions are not assessed.

PD is assigned if any non-target lesion is marked "Increased" on the "Non-Target Lesion" eCRF. However, in an effort to programmatically define "unequivocal progression" of non-target lesions, the derived non-target lesion response will also take into account the "Non-Target Lesions" assessment from the IOTA eCRF, as noted in Table 17.

- Note: the lesions assessed are only considered for CR, Non-CR/Non-PD and PD if the methods of assessments are consistent with baseline. The "interchangeable" methods noted in Section 10.1.2 above are all considered consistent methods.

090177e1a0219102\Approved\Approved On: 08-Mar-2024 15:37 (GMT)

**Table 17. Derivation of Non-Target Lesion Assessment**

| Non-Target lesion assessment status | "Non-Target lesions" assessment status on IOTA eCRF | Derived non-target lesion response |
|---|---|---|
| PD ("Increased" is marked for ≥1 lesion) | PD | PD |
| | Non-CR/Non-PD | Non-CR/Non-PD |
| | CR | Non-CR/Non-PD |
| | Not Assessed (NA) | PD |
| | No Baseline (NB) | PD |
| | Indeterminate (IND) | NAE |
| | Missing | PD |

*Instruction: Relevant discrepancies between the non-target lesion assessment status and information on the IOTA page must be queried.*
Abbreviations: IOTA = investigator overall tumor assessment; eCRF = electronic case report forms; CR = Complete Response; PD = Progressive Disease; NA = Not Assessed; NB = No Baseline; IND = Indeterminate; NAE= Not All Evaluated.

### 10.2.3. New Lesion Evaluation Criteria

A new lesion is defined by the appearance of 1 or more new lesions (where any lesion is marked "New" on the "New Lesions" eCRF).

Any lesion that is recorded for the first time after the start date without being marked as "New" on the "New Lesions" eCRF must be queried. In case the inconsistency is not resolved at time of database snapshot/lock, the lesion will be considered as new and the patient in progression at that time point.

The requirement for consistent methods of assessment with baseline does not apply for new lesions.

If a new lesion is equivocal at one assessment (classified as "Equivocal") and verified at the next assessment, (classified as "Unequivocal"), the earlier date is used as the progression date.

## 10.3. Objective Response Status at Each Assessment

Objective response status is determined from the derived target and derived non-target lesion data using the conventions in Table 18 under the assumption that there are no new lesions identified at the visit.

Objective status after a change in modality that is not considered interchangeable is classified as Not Evaluable (NE).

If there are any new lesions at a time point, then the response is PD at that time point regardless of target or non-target lesion response.



090177e1a0219102\Approved\Approved On: 08-Mar-2024 15:37 (GMT)

**Table 18. Derivation of Objective Status Based on Target and Non-Target Lesion Response Assuming No New Lesions***

| Target lesion response | Non-Target lesion response | Objective response status |
|---|---|---|
| CR | CR | CR |
| CR | Non-CR/Non-PD | PR |
| CR | PD | PD |
| CR | NAE | PR |
| PR | CR | PR |
| PR | Non-CR/Non-PD | PR |
| PR | PD | PD |
| PR | NAE | PR |
| SD | CR | SD |
| SD | Non-CR/Non-PD | SD |
| SD | PD | PD |
| SD | NAE | SD |
| PD | Any | PD |
| NAE | PD | PD |
| NAE | CR<br>Non-CR/Non-PD<br>NAE | NE |
| NB | CR | CR |
| | Non-CR/Non-PD | Non-CR/Non-PD |
| | PD | PD |
| | NAE | NE |
| CR | NB | CR |
| PR | | PR |
| SD | | SD |
| PD | | PD |
| NAE | | NE |

*If there are any new lesions at a time point, then the response is PD at that time point regardless of target or non-target lesion response.

Abbreviations: CR = Complete Response; PR = Partial Response; SD = Stable Disease; PD = Progressive Disease; NAE = Not All Evaluated; NB = No Baseline; NE = Not Evaluable.

Note: If non-target (or target) lesions are not collected at baseline, then the overall response is equivalent to the target (or non-target) lesions response, respectively.

## 10.4. Date of Response at Each Assessment/Overall:

The date of CR, PR, PD, non-CR/non-PD, SD, NE is derived as the date of the first radiographic evaluation included in the cluster.

The date of first response for purposes of calculating duration of response and time to response is defined as the first date a CR or PR was documented.

### 10.4.1. Best Overall Response Evaluation for Each Patient

BOR is derived from the sequence of objective responses reported during the “Period for Derivation of Best Overall Response” according to the rules specified in Section 4.2.1.5 of the Oncology Rulebook.

090177e1a0219102\Approved\Approved On: 08-Mar-2024 15:37 (GMT)

Table 19 presents derivation of BOR for specific cases assuming that confirmation of response is not required.

**Table 19. Derivation of Best Overall Response Based on Objective Status at Assessment Time Points Assuming That Confirmation of Response is Not Required**

| Week 6 | Week 12 | Week 18 | Best overall response |
|---|---|---|---|
| Early Death | | | NE |
| CR | PD | | uCR |
| CR | NE | | uCR |
| CR | PR | | uCR |
| PR | PD | | uPR |
| PR | CR | PD | uCR |
| PR | SD | | uPR |
| PR | NE | | uPR |
| SD | PD | | SD[a] |
| SD | NE | | SD[b] |
| Non-CR/non-PD | PD | | Non-CR/non-PD[c] |
| Non-CR/non-PD | PD | | PD[d] |
| NE | PD | | PD |
| NE | SD | PD | SD |
| NE | NE | PD | NE |

Abbreviations: CR = Confirmed Response; PR = Partial Response; SD = Stable Disease; PD = Progressive Disease; NE = Not Evaluable; uCR = unconfirmed CR; uPR = unconfirmed PR.

a. If SD was documented less than 42 days after start date, the best overall response will be PD.

b. If SD was documented less than 42 days after start date, the best overall response will be NE.

c. Applicable when only non-target lesions are present at baseline. If non-CR/non-PD documented more than 41 days after start date.

d. Applicable when only non-target lesions are present at baseline. If non-CR/non-PD documented less than 42 days after start date.

Table 20 presents derivation of BOR for specific cases assuming that confirmation of response is required.

090177e1a0219102\Approved\Approved On: 08-Mar-2024 15:37 (GMT)

### Table 20. Best Overall Response When Confirmation of Response is Required

| Overall response first time point | Overall response subsequent time point | Best overall response |
|---|---|---|
| CR | CR | CR |
| CR | PR | SD, PD, or PR[a] |
| CR | SD | SD provided minimum criteria for SD duration met, otherwise PD |
| CR | PD | SD provided minimum criteria for SD duration met, otherwise PD |
| CR | NE | SD provided minimum criteria for SD duration met, otherwise NE |
| PR | CR | PR |
| PR | PR | PR |
| PR | SD | SD |
| PR | PD | SD provided minimum criteria for SD duration met, otherwise PD |
| PR | NE | SD provided minimum criteria for SD duration met, otherwise NE |
| NE | NE | NE |

*Note that best overall response of CR or PR requires confirmation ≥4 weeks after response is first observed. Minimum duration criteria for SD must be met for a best overall response of SD.*

Abbreviations: CR = complete response; PR = partial response; SD = stable disease; PD = progressive disease; NE = not evaluable.

a. If a CR is *truly* met at first time point, then any disease seen at a subsequent time point, even disease meeting PR criteria relative to baseline, makes the disease PD at that point (since disease must have reappeared after CR). Best response would depend on whether minimum duration for SD was met. However, sometimes "CR" may be claimed when subsequent scans suggest small lesions were likely still present and in fact the patient had PR, not CR at the first time point. Under these circumstances, the original CR should be changed to PR and the best response is PR.

090177e1a0219102\Approved\Approved On: 08-Mar-2024 15:37 (GMT)

Table 21 presents derivation of BOR for specific examples of overall response at sequential time points.

**Table 21. Best Overall Response When Confirmation of Response is Required: Further Examples**

| Overall response sequential time points | Best overall response |
|---|---|
| SD - CR - CR - PR | CR if the two CRs were ≥4 weeks apart; else, SD if at any time on or before the 3rd time point evaluation the minimum criteria for SD duration was met; else PD |
| SD - PR - CR - PR | PR if the 2nd and 3rd evaluations (PR, CR) were ≥4 weeks apart; else SD if at any time on or before the 3rd time point evaluation the minimum criteria for SD duration was met; else PD |
| SD - CR - SD - CR | SD if at any time on or before the 2nd time point evaluation the minimum criteria for SD duration was met; else PD |
| PR - CR - NA - CR - PR | CR if the 2nd and 4th evaluations (CR, CR) were ≥4 weeks apart; else PR if the 1st and 2nd evaluations (PR, CR) or the 1st and 4th evaluations (PR, CR) were ≥4 weeks apart; else SD if at any time on or before the 4th time point evaluation the minimum criteria for SD duration was met; else PD |
| PR - CR - CR - PR | CR if the 2nd and 3rd evaluations (CR, CR) were ≥4 weeks apart; else PR if the 1st and 2nd evaluations (PR, CR) or the 1st and 3rd evaluations (PR, CR) were ≥4 weeks apart; else SD if at any time on or before the 3rd time point evaluation the minimum criteria for SD duration was met; else PD |
| PR - CR - CR - SD | CR if the 2nd and 3rd evaluations (CR, CR) were ≥4 weeks apart; else PR if the 1st and 2nd evaluations (PR, CR) or the 1st and 3rd evaluations (PR, CR) were ≥4 weeks apart; else SD if at any time on or before the 3rd time point evaluation the minimum criteria for SD duration was met; else PD |
| CR - NE - CR - SD | CR if the 1st and 3rd evaluations (CR, CR) were ≥4 weeks apart; else SD if at any time on or before the 3rd time point evaluation the minimum criteria for SD duration was met; else PD |
| PR - PR - CR - PR | PR if 2 of the first 3 evaluations (1st to 2nd, or 1st to 3rd, or, 2nd to 3rd) were ≥4 weeks apart; else SD if at any time on or before the 3rd time point evaluation the minimum criteria for SD duration was met; else PD |
| PR - PR - CR - SD | PR if 2 of the first 3 evaluations were ≥4 weeks apart (1st to 2nd, or 1st to 3rd, or, 2nd to 3rd); else SD if at any time on or before the 3rd time point evaluation the minimum criteria for SD duration was met; else PD |
| CR - SD - SD - CR | SD if at the 1st time point evaluation the minimum criteria for SD duration was met; else PD |
| PR - CR - PR - CR | PR if the 1st and 2nd time point evaluations (PR, CR) were ≥4 weeks apart; else SD if at any time on or before the 2nd time point evaluation the minimum criteria for SD duration was met; else PD |

If a CR is truly met at first time point, then any disease seen at a subsequent time point, even disease meeting PR criteria relative to baseline, makes the disease PD at that point (since disease must have reappeared after CR). Best response would depend on whether minimum duration for SD was met.
Abbreviations: CR = complete response; PR = partial response; SD = stable disease; PD = progressive disease; NE = not evaluable; NA = not assessed.

090177e1a0219102\Approved\Approved On: 08-Mar-2024 15:37 (GMT)
