# Supplementary material for: Encorafenib, cetuximab and chemotherapy in BRAF-mutant colorectal cancer: a randomized phase 3 trial
Source: Nat Med. 2025 Jan 25;31(3):901–8. doi: 10.1038/s41591-024-03443-3 (PMC11922750; doi:10.1038/s41591-024-03443-3)
Supplement: Supplementary file 2 — Reporting Summary [file 41591_2024_3443_MOESM2_ESM.pdf]

nature portfolio

Corresponding author(s): Scott Kopetz

Last updated by author(s): Nov 5, 2024

Reporting Summary

Nature Portfolio wishes to improve the reproducibility of the work that we publish. This form provides structure for consistency and transparency in reporting. For further information on Nature Portfolio policies, see our Editorial Policies and the Editorial Policy Checklist.

Statistics

For all statistical analyses, confirm that the following items are present in the figure legend, table legend, main text, or Methods section.

n/a	Confirmed	
☐	☒	The exact sample size (*n*) for each experimental group/condition, given as a discrete number and unit of measurement
☒	☐	A statement on whether measurements were taken from distinct samples or whether the same sample was measured repeatedly
☐	☒	The statistical test(s) used AND whether they are one- or two-sided<br>*Only common tests should be described solely by name; describe more complex techniques in the Methods section.*
☐	☒	A description of all covariates tested
☐	☒	A description of any assumptions or corrections, such as tests of normality and adjustment for multiple comparisons
☐	☒	A full description of the statistical parameters including central tendency (e.g. means) or other basic estimates (e.g. regression coefficient) AND variation (e.g. standard deviation) or associated estimates of uncertainty (e.g. confidence intervals)
☐	☒	For null hypothesis testing, the test statistic (e.g. *F*, *t*, *r*) with confidence intervals, effect sizes, degrees of freedom and *P* value noted<br>*Give P values as exact values whenever suitable.*
☒	☐	For Bayesian analysis, information on the choice of priors and Markov chain Monte Carlo settings
☐	☒	For hierarchical and complex designs, identification of the appropriate level for tests and full reporting of outcomes
☐	☒	Estimates of effect sizes (e.g. Cohen's *d*, Pearson's *r*), indicating how they were calculated

Our web collection on statistics for biologists contains articles on many of the points above.

Software and code

Policy information about availability of computer code

Data collection	Inform database
Data analysis	Statistical analyses were performed using SAS version 9.4 or higher

For manuscripts utilizing custom algorithms or software that are central to the research but not yet described in published literature, software must be made available to editors and reviewers. We strongly encourage code deposition in a community repository (e.g. GitHub). See the Nature Portfolio guidelines for submitting code & software for further information.

Data

Policy information about availability of data

All manuscripts must include a data availability statement. This statement should provide the following information, where applicable:

- Accession codes, unique identifiers, or web links for publicly available datasets
- A description of any restrictions on data availability
- For clinical datasets or third party data, please ensure that the statement adheres to our policy

The analyses in this paper were based on a data cutoff of December 22, 2023.
Upon reasonable request and subject to review, Pfizer will provide the data that support the findings of this study. Subject to certain criteria, conditions, and exceptions, Pfizer may also provide access to the related individual de-identified participant data from Pfizer-sponsored global interventional clinical studies conducted for medicines, vaccines, and medical devices (1) for indications that have been approved in the United States and/or European Union or (2) in programs

that have been terminated (that is, development for all indications has been discontinued). Pfizer will also consider requests for the protocol, data dictionary, and statistical analysis plan. See https://www.pfizer.com/science/clinical-trials/trial-data-and-results for more information.
Data may be requested from Pfizer trials 24 months after study completion. The de-identified participant data will be made available to researchers whose proposals meet the research criteria and other conditions, and for which an exception does not apply, via a secure portal. To gain access, data requestors must enter into a data access agreement with Pfizer.

Human research participants

Policy information about studies involving human research participants and Sex and Gender in Research.

Reporting on sex and gender	The self-reported sex of patients was collected along with other demographics categories; gender was not collected. 50.5% were male and 49.5% were female. Objective response rates in male and female patients were prespecified and reported in Figure 2.
Population characteristics	Patients with histologically or cytologically confirmed, metastatic CRC with the BRAF V600E mutation who had not received treatment systemic treatment for metastatic disease. The patient population was mostly male (50.5%) and White (59.5%), with a median age of 61.0 years. Most patients' tumors were located in the colon (80.0%) and were right-sided (61.0%), other characteristics are reported in a table in the manuscript.
Recruitment	Participants were recruited by investigators at each participating study site. Sites typically present the study to the oncology physicians at the site who then offer participation of the study to patients that they believe may be eligible. Although there may be some selection bias on the part of the physician and/or the patient, this is difficult to control, is not expected to be higher on this study than on other similar clinical trials, and is unlikely to substantially impact results. Participants were not compensated for study participation, although certain trial-related expenses (e.g., hotel rooms, transportation) were reimbursed for some patients.
Ethics oversight	The trial was approved by the institutional review board or independent ethics committee at each participating center.

Note that full information on the approval of the study protocol must also be provided in the manuscript.

Field-specific reporting

Please select the one below that is the best fit for your research. If you are not sure, read the appropriate sections before making your selection.

☒ Life sciences ☐ Behavioural & social sciences ☐ Ecological, evolutionary & environmental sciences

For a reference copy of the document with all sections, see nature.com/documents/nr-reporting-summary-flat.pdf

Life sciences study design

All studies must disclose on these points even when the disclosure is negative.

Sample size	637 patients were randomized to the phase 3 portion of the study. This manuscript reports data from 479 patients in the EC+FOLFOX and SOC arms.
Data exclusions	No data were exclused
Replication	Not applicable as this is a clinical trial.
Randomization	Patients were assigned using a Interactive Response Technology; randomization was stratified by ECOG and region.
Blinding	Not applicable as this was an open-label study.

Reporting for specific materials, systems and methods

We require information from authors about some types of materials, experimental systems and methods used in many studies. Here, indicate whether each material, system or method listed is relevant to your study. If you are not sure if a list item applies to your research, read the appropriate section before selecting a response.

Materials & experimental systems

n/a	Involved in the study
☒	☐ Antibodies
☒	☐ Eukaryotic cell lines
☒	☐ Palaeontology and archaeology
☒	☐ Animals and other organisms
☐	☒ Clinical data
☒	☐ Dual use research of concern

Methods

n/a	Involved in the study
☒	☐ ChIP-seq
☒	☐ Flow cytometry
☒	☐ MRI-based neuroimaging

Clinical data

Policy information about clinical studies

All manuscripts should comply with the ICMJE guidelines for publication of clinical research and a completed CONSORT checklist must be included with all submissions.

Clinical trial registration	NCT04607421
Study protocol	The latest version of the protocol is included in the submission as supplementary information.
Data collection	Data were collected at various sites in 28 countries. Patients were enrolled between November 16, 2021 and December 22, 2023. The data cut off was December 22, 2023.
Outcomes	This analysis reports objective response, response duration and time to response (by blinded independent central review and by investigator), overall survival, and safety.
